# A Photocatalytic Approach to Radical 1-(Trifluoromethyl)cyclopropanation

**DOI:** 10.1021/acscatal.5c01642

**Published:** 2025-04-17

**Authors:** Sven Timmann, Moritz T. H. Dilchert, Jörg Dietzel, Verena S. Pöltl, Marc R. Wennekamp, Christopher Golz, Manuel Alcarazo

**Affiliations:** Institut für Organische und Biomolekulare Chemie, Georg-August-Universität Göttingen, Tammannstr 2, Göttingen 37077, Germany

**Keywords:** bioisosteric replacement, photoredox catalysis, radical alkylation, sulfonium salts, 1-(trifluoromethyl)cyclopropane

## Abstract

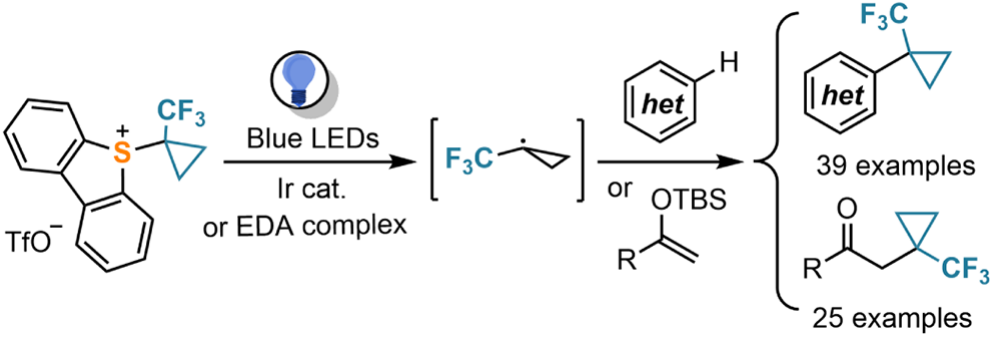

A simple protocol for the multigram-scale synthesis of 5-(1-(trifluoromethyl)cyclopropyl)dibenzothiophenium triflate is reported. This benchtop-stable reagent efficiently releases 1-(trifluoromethyl)cyclopropyl radicals under mild photochemical conditions, enabling the straightforward incorporation of that privileged chemotype at previously nonfunctionalized positions of (hetero)arenes and silyl enol ethers. The trifluoromethylcyclopropanation protocols herein reported are associated with an exceedingly broad substrate scope, remarkable functional group compatibility, high regioselectivity, and synthetically useful yields, making this reagent especially suitable for the preparation of medicinally relevant building blocks.

## Introduction

**Scheme 1:**
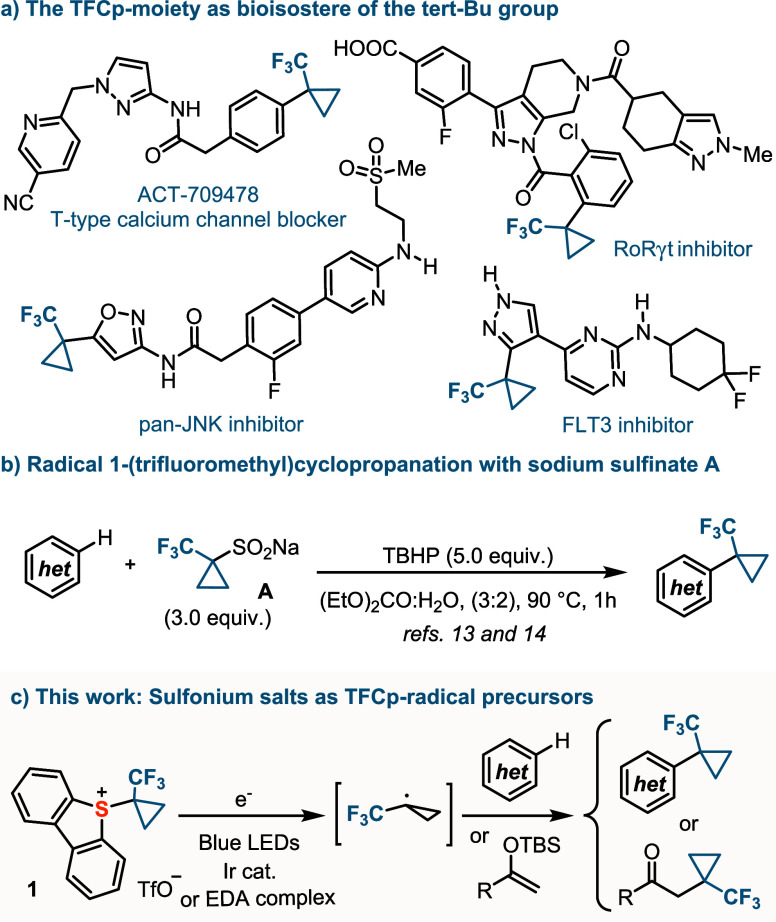
1-(Trifluoromethyl)cyclopropane Unit: (a) Representative Bioactive Compounds Containing the TFCp Moiety; (b) Generation of TFCp Radicals from the Corresponding Sodium Sulfinate; (c) Our Approach to Generate TFCp Radicals via Single-Electron Reduction of Sulfonium Salts

The optimization of the metabolic stability of lead compounds during the drug discovery process is often challenging due, among others factors, to the opposing requirements for absorption and metabolism.^1^ A rational approach to address this dichotomy requires the identification and conservation of the structural core and functional groups that are essential for the desired biological activity and the subtle modification of those molecular regions that having little or no impact on activity may contribute to the overall improvement of metabolic parameters. Thus, the introduction of substituents that cap metabolically vulnerable sites or the replacement of metabolically labile groups with appropriate isosteres are common practices at the development stage in drug discovery.^2^ This is actually the case for the *tert*-butyl substituent, whose incorporation into structures of interest tends to be avoided because of its known lability.^3^ Alternatives to that moiety include the pentafluorosulfanyl-,^4^ bicyclo[1.1.1]pentanyl-,^5^ 1-(trifluoromethyl)cyclopropyl (TFCp),^6^ and more recently also the 1-(trifluoromethyl)cyclobutyl groups.^7^ Specifically, the use of the TFCp fragment has rapidly gained prominence in drug discovery campaigns (Scheme 1a),^8^ and this growing demand has encouraged the development of efficient synthetic routes for the assembly of this group from a variety of precursors, including appropriately substituted alkenes,^6,9^ 1-trimethylsilyl-3-tosyl-3-trifluoromethylpropanes,^10^ and cyclopropane carboxylic acids,^11^ among others.^12^ Yet, for the expansion of developing pharmaceutical libraries, operationally simple protocols able to directly append the TFCp unit onto advanced intermediates or even the actual lead compounds are particularly desirable.

Baran pioneered this approach with the preparation of the corresponding diversinate,^13^ sodium trifluorocyclopropylsulfinate (**A**), a salt that under oxidative conditions delivers the electrophilic TFCp radical, thus enabling its attachment onto heteroarenes via a Minisci-type mechanism (Scheme 1b).^14^ However, the temperatures required for the TFCp moiety to be transferred are elevated compared to other sulfinates, and the reported substrate scope is still surprisingly narrow,^14a,15^ reflecting the difficulty to generate the tertiary TFCp radical through this method and/or incompatibility between the oxidative reaction conditions and electron-rich substrates. More recently, Qin and coworkers have achieved the coupling between (hetero)arenes and TFCp by reaction of **A** with aryl Grignard reagents.^16^ The method is highly modular and site-programmable, but it requires prehalogenation of substrates and suffers from the limited functional group tolerance usually associated with the use of organomagnesium reagents.^17^

Being aware of the ability of sulfonium salts to act as precursors for radicals of different structures, including (poly)fluoroalkyl ones,^18^ we envisioned a new reagent, 5-(1-(trifluoromethyl)cyclopropyl)dibenzothiophenium triflate **1**, which is expected to deliver TFCp radicals under reductive photocatalytic conditions (Scheme 1c). Trapping such species with moderately nucleophilic substrates should provide convenient access to previously unavailable TFCp-substituted scaffolds of potential value in drug discovery.

## Results and Discussion

**Scheme 2:**
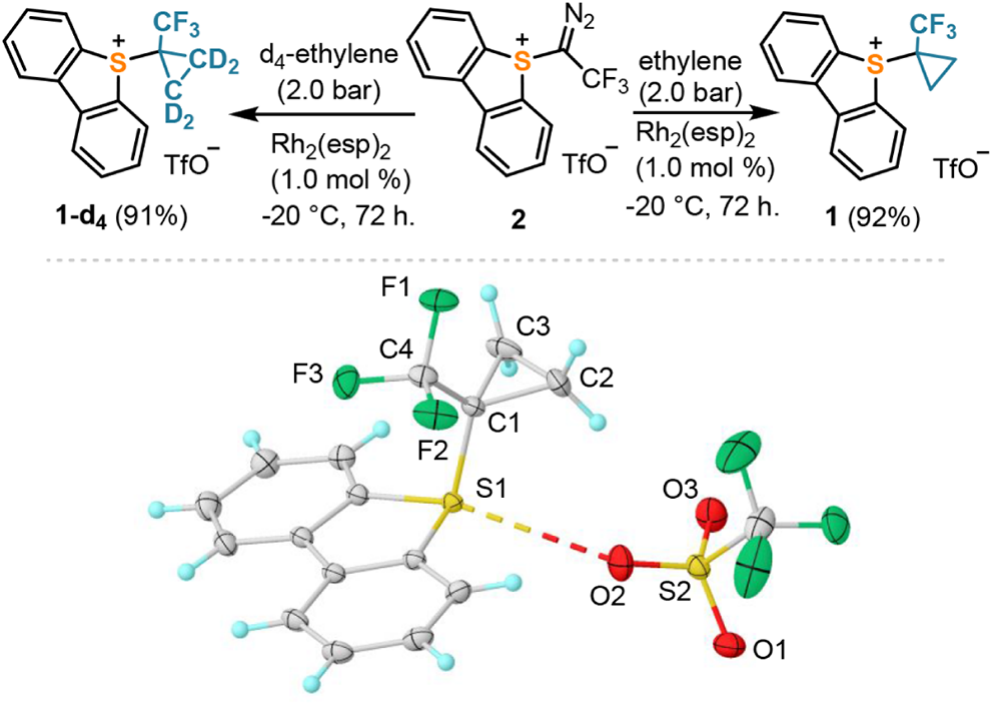
Synthesis of **1** and **1-d**_**4**_ and Structure of **1** in the Solid State Anisotropic displacements shown at 50% probability level. Selected bond lengths [Å]: S1–C1, 1.7914(10); C1–C2, 1.5081(14); C1–C3, 1.5127(14); C2–C3, 1.495(2); C1–C4, 1.5038(14); S1–O2, 3.2505(10).^25^

Having recently developed a multigram-scale synthesis for α-diazo sulfonium salt **2**,^19^ which serves as a precursor of sulfoniocarbenes under Rh-catalysis,^20^ we envisioned that its reaction with ethylene might be a straightforward entry to **1**. To our delight, the cyclopropanation reaction works particularly well, provided that the reaction is carried out under a slight overpressure of ethylene (2.0 bar), and the mixture is kept at −20 °C until complete consumption of **2**. Addition of diethyl ether to the crude product causes the precipitation of **1**, which is obtained as an analytically pure white powder in 92% isolated yield (Scheme 2). An identical procedure, but employing *d*_4_-ethylene instead, delivers the tetradeuterated analogue reagent **1-d**_**4**_.

In the solid state, the central sulfur atom (S1) of **1** adopts a trigonal-pyramidal coordination environment, with the sum of the bond angles around this atom being 304.4°. The S1–C1 bond distance is typical for a S–C(sp^3^) single bond (1.7914(10) Å), and a weak interaction between the S atom and the triflate counteranion is detected. However, the S1···O1 distance (3.2505(10) Å is clearly longer than that found in structurally related 5-aryl,^21^ 5-alkynyl,^22^ or 5-(pentafluorocyclopropyl) dibenzothiophenium triflates,^23^ suggesting only moderate electrophilic character at S1 in that salt. The thermal decomposition of **1** was studied by differential scanning calorimetry-thermogravimetric analysis (DSC-TGA). An exothermic event was detected just after reaching the melting point at 170 °C, leading to moderate/low energy release (226 J g^–1^). From these results, the probability of a thermal runaway of **1** under adiabatic conditions (30 °C) is classified as low.^24^

The reduction potential (*E*_red_) of **1** has been determined by cyclic voltammetry to be −1.45 V vs. Fc^+/0^ (irrev.; CH_3_CN); a value in the same range as those measured for 5-aryl dibenzothiophenium salts,^22^ and considerably less negative than the one characteristic for the excited state of Ir(ppy)_3_ (*E*_red_* = −2.13 V vs Fc^+/0^ in CH_3_CN),^26^ indicating the feasibility of the TFCp radical to be generated through a single-electron transfer from the Ir-photocatalyst to **1**.

Considering the ambiphilic character of the TFCp radical (predicted global electrophilicity and nucleophilicity indices: ω = 1.58; ω^–^ = 0.28, respectively),^27^ the radical transfer process was initially tested using electron-rich silyl enol ether **3a** as a substrate to produce the corresponding α-(TFCp) ketone **4a**.^28^ Pleasingly, when the experiment was run in the presence of Ir(ppy)_3_ (1.0 mol %) and under blue-light irradiation (462 nm), the reaction delivered the desired ketone **4a** in an already promising 74% NMR yield (Table 1; entry 1). The use of an excess of **3a** (2.0 equiv) had no beneficial effect (entry 2), whereas a slight excess of **1** (1.3 equiv) substantially increased the yield of **4a** (80% isolated, entry 3). Formal exchange of the TBS group in **2a** by TMS (substrate **2b**) was counterproductive and resulted in a significant drop in yield (entry 4). Control experiments highlighted that both light and the Ir-photocatalyst are required to trigger the radical reactivity (entries 5 and 6), while the addition of TEMPO inhibited the reaction completely (entry 7).

**Table 1:**
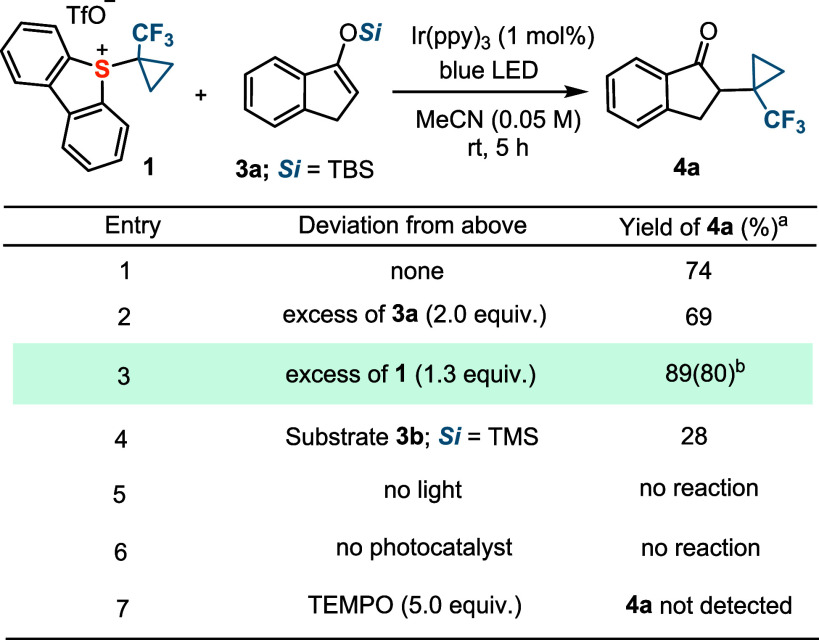
Photochemical Transfer of the TFCp Radical to Silyl Enol Ethers^a^^b^

a Yields were determined by ^19^F NMR spectroscopy using the triflate signal as an internal standard; isolated yields are given in parentheses.

b Reaction conducted on a 0.2 mmol scale. All reactions were performed on a 0.05 mmol scale.

**Scheme 3:**
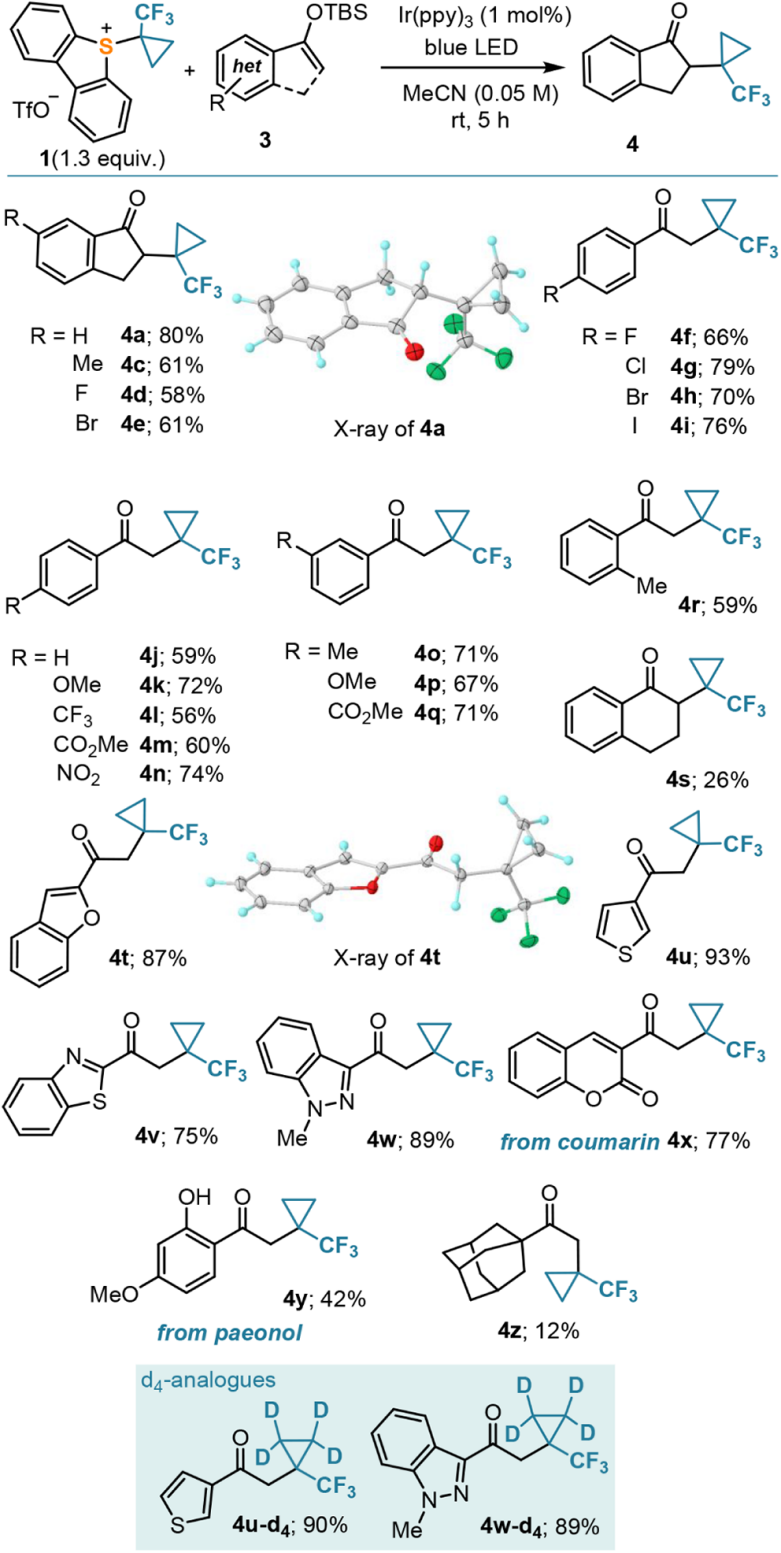
Scope of the (Trifluoromethyl)cyclopropanation of Silyl Enol Ethers

With conditions already optimized for the radical alkylation, a complete series of silyl enol ethers were evaluated as substrates. Indanone derivatives bearing alkyl or halogen substituents (**3c**–**e**) proved to be adequate reaction partners producing the TFCp-substituted derivatives in good yields (**4c**–**e**). Similarly, acetophenones (**3f**–**r**) performed well and delivered the corresponding ketones regardless of their substitution pattern, which included halogens (**4e**–**i**), common electron-donating groups (**4j**–**k**; **4p**–**o**; **4r**–**s**), and also electron-withdrawing groups (**4l**–**n**; **4q**) attached to the aromatic ring. The efficient formation of ketones **4t**–**w** also indicated that heterocycles are perfectly tolerated and showcased the compatibility of our method with a broad array of structures (Scheme 3). Natural products such as the coumarin derivative **3x** and paeonol (**3y**) also got involved in the radical cyclopropanation reaction delivering ketones **4x** and **4y**, respectively. On the other hand, silyl enol ethers derived from aliphatic ketones did not afford the desired products. In most of these assays, trifluoromethylcyclopropane was the sole fluorine-containing product detected; only the 1-adamantyl-substituted silyl enol ether **3z** delivered some (12% yield) of the corresponding cyclopropanated ketone **4z**.

Substrates **3u** and **3w** were additionally submitted to the action of reagent **1-d**_**4**_ to obtain the corresponding tetra-deuterated products **4u-d**_**4**_ and **4w-d**_**4**_ in yields identical to those of the unlabeled analogues (Scheme 3). No scrambling of deuterium to positions other than those of the cyclopropyl moiety was detected by ^2^H NMR spectroscopy, showcasing the utility of this methodology for the selective incorporation of the tetra-deuterated *tert*-butyl bioisostere into organic building blocks. Finally, the atom connectivity of **4a** and **4t** was confirmed by X-ray crystallography.^25^

In an attempt to further evaluate the scope of the radical process just set out, the C–H alkylation of quinoxalinones was attempted following the same protocol; *N*-methylquinoxalinone **5a** was chosen as the model substrate for the optimization studies.^29^ To our delight, the initial control experiment already revealed that the reaction proceeds with moderate yield under conditions very similar to those optimized for **3a**; only the addition of K_3_PO_4_ was necessary to quench the triflic acid formed (Table 2, entry 1). However, further screening demonstrated that the operating mechanism was different for substrate **5a**. The Ir-catalyst plays no role (entry 2), whereas the nature of the base employed seems to be crucial; only a trace of **6a** is detected when no K_3_PO_4_ is added to the reaction mixture (entry 3), or even if it is replaced by Na_2_CO_3_ (entry 4). Light is required in any case (entry 5), and the yield of **6a** increases when an excess of **5a** (2.0 equiv) is used (entries 6 and 7).

**Table 2:**
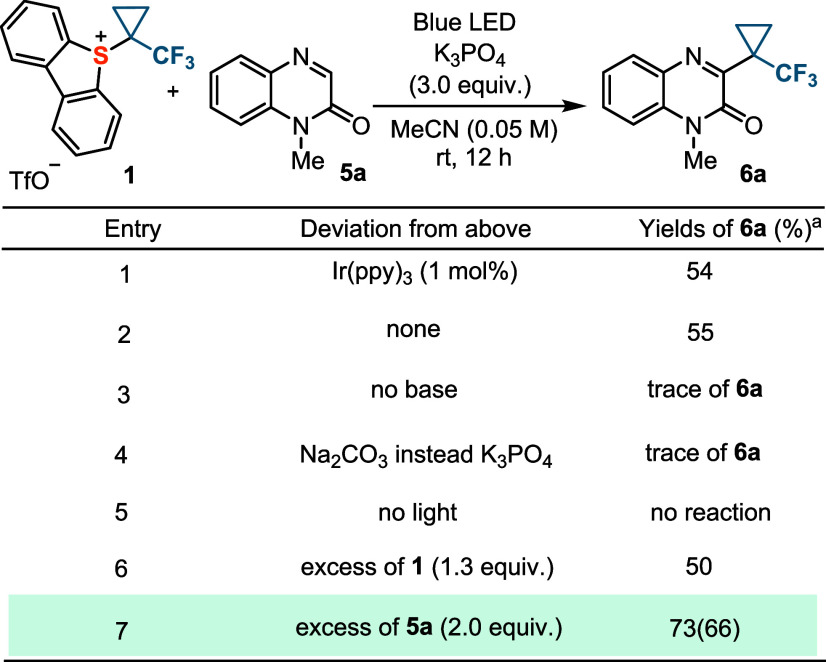
Photochemical Cyclopropanation of *N*-Methylquinoxalinone **5a**^a^

a Yields were determined by ^19^F NMR spectroscopy using the triflate signal as an internal standard; isolated yields are given in parentheses. All reactions were performed on a 0.05 mmol scale.

**Scheme 4:**
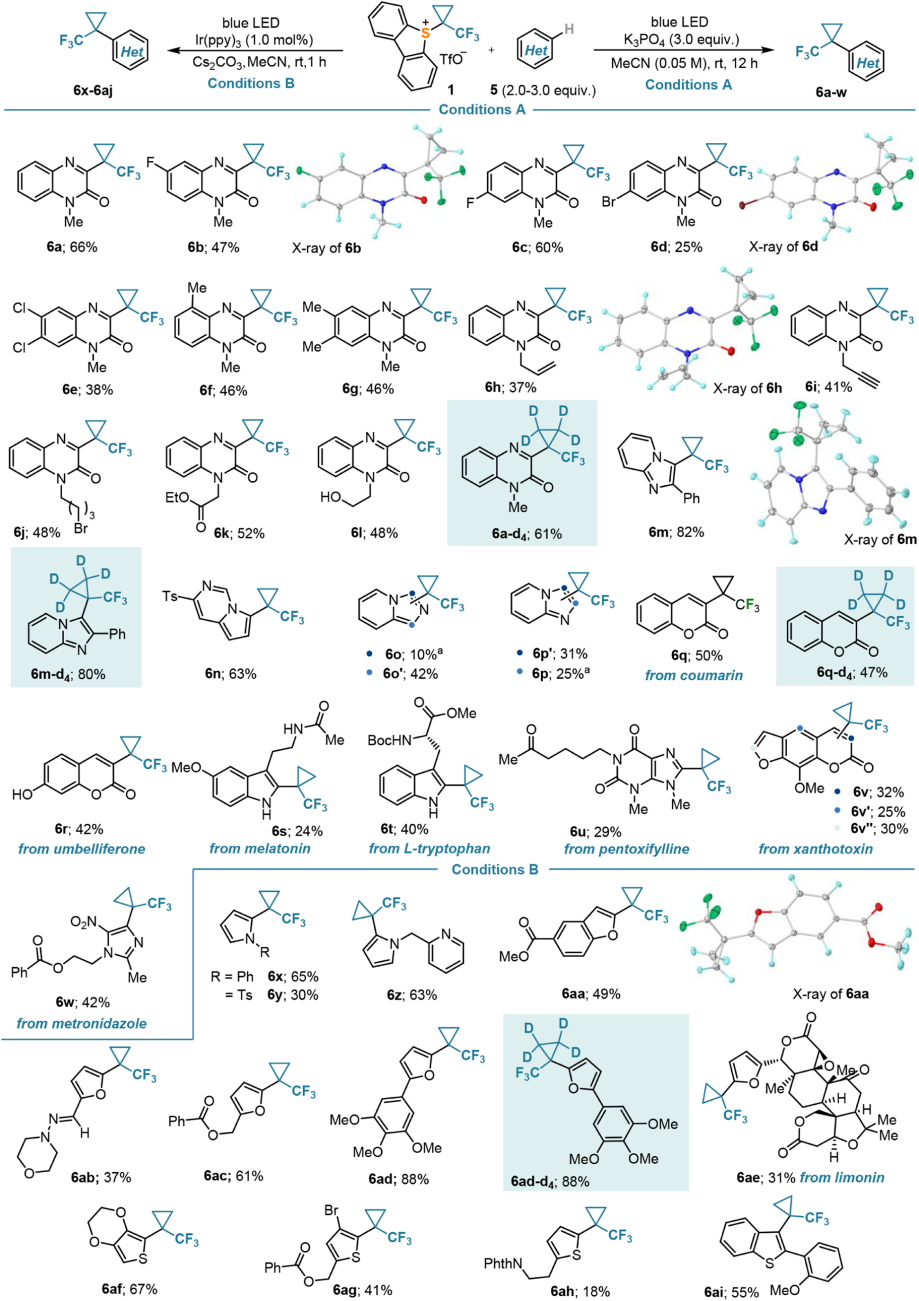
(Trifluoromethyl)cyclopropanation of Heteroarenes NMR yields.

An initial evaluation of the compatibility of this C–H functionalization with common substituents was performed on quinoxalinone substrates. Halogens (**6b**–**e**,**j**), terminal olefins (**6h**), alkynes (**6i**), esters (**6k**), and alcohols (**6l**) were tolerated. Moreover, the transfer of the TFCp radical to other heterocyclic structures often encountered in medicinal chemistry, such as pyrrolo[1,2-*c*]pyrimidine (**6n**), imidazo[1,5-*a*]pyridine (**6o**), imidazo[1,2-*a*]pyridine (**6p**), and imidazole (**6u**,**w**), or naturally occurring coumarins (**6q**,**r**,**v**) and indoles (**6s**,**t**), was equally satisfactory and delivered the substituted heterocycles with moderate to good yields (Scheme 4, conditions A). On the other hand, the method seemed to be incompetent for the functionalization of pyrroles, (benzo)furans, and (benzo)thiophenes. Yields were often low even after extended reaction times, and specifically, furan and thiophene derivatives completely failed to react. For these heterocycles, the use of the Ir-photocatalyst (Ir(ppy)_3_; 1.0 mol %) was required, as in the case of silyl enol ethers **3**. After this modification of the protocol, the corresponding TFCp-substituted products (**6x**–**6ai**) were obtained in moderate to good yields (Scheme 4, conditions B). Pyridines and other electron-deficient heteroarenes, which would be typical substrates for Minisci-type chemistry, did not function as appropriate reaction partners regardless of the conditions applied. Most of the building blocks **6** were virtually delivered as single regioisomers with incorporation of the TFCp moiety at the innately electron-rich site(s) of their heteroaromatic core. Control experiments by ^2^H NMR spectroscopy did not detect scrambling of deuterium during the installation of the tetradeuterated TFCp moiety to heterocycles. The connectivity of products **6b**, **6d**, **6h**, **6m**, and **6aa** has been unambiguously confirmed by X-ray diffraction analyses (see the Supporting Information). Inspection of these structures revealed the preference of the F_3_*C*–*C*(Cp) bond to stand nearly orthogonal to the plane defined by the π-system (Φ = 85.06(9)° (**6b**), 82.99(13)° (**6d**), 84.13(9)° (**6h**), 83.22(5)° (**6m**), and 57.40(3)° (**6aa**); this conformation minimizes steric hindrance and additionally, it facilitates stabilizing hyperconjugative π→σ*(*C*(Cp)–*C*F_3_) interactions.

**Scheme 5:**
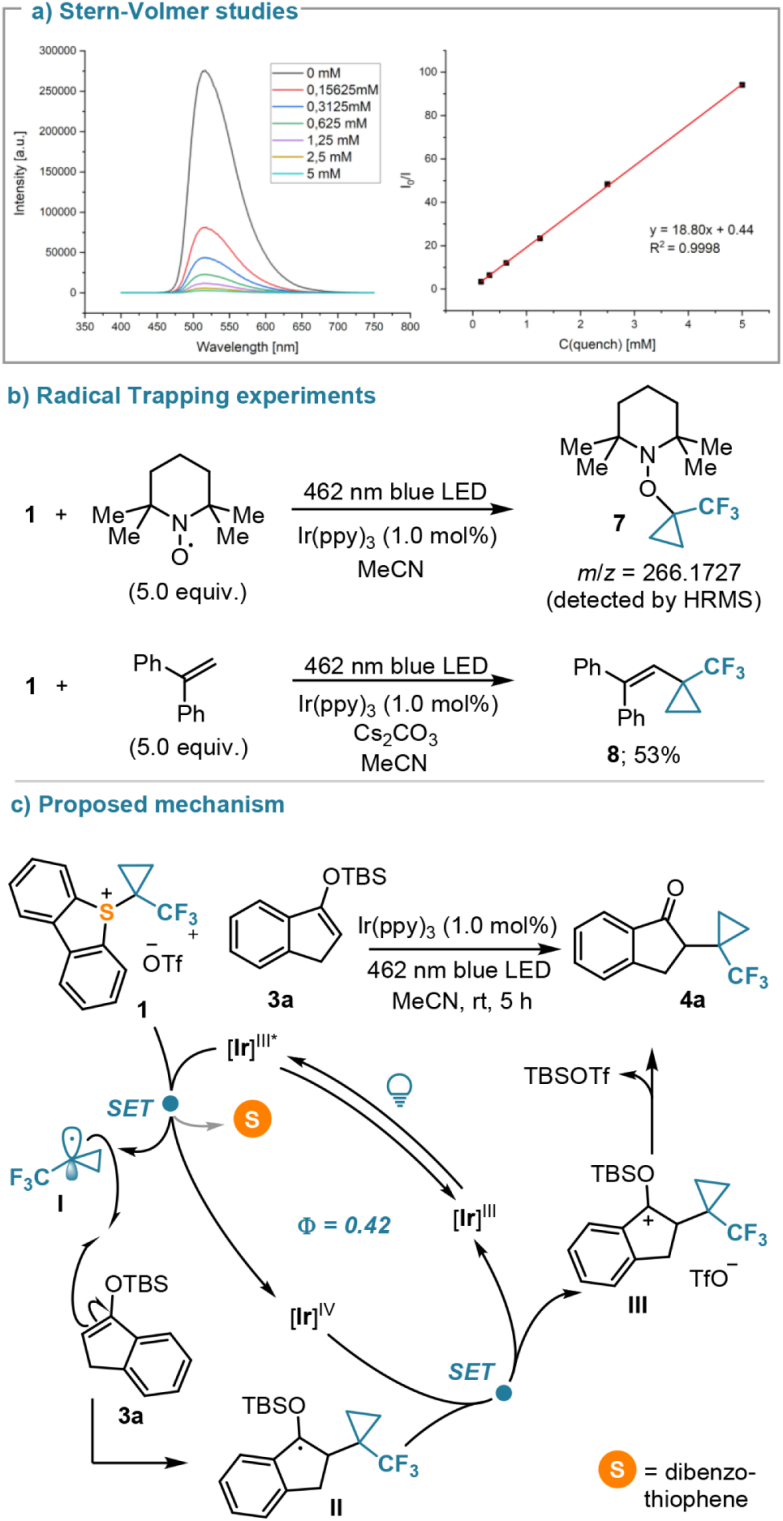
Mechanistic Proposal for the TFCp Transfer to Silyl Enol Ethers: (a) Stern–Volmer Studies of **1** and Determination of KSV; (b) Radical Trapping Experiments; (c) Proposed Mechanism

The results just described point toward two operating mechanistic scenarios, which have been studied in detail using silyl enol ether **3a** and quinoxalinone **5a** as model substrates. We initially conducted a fluorescence quenching analysis (Stern–Volmer plot), which revealed efficient quenching of the excited Ir-photocatalyst in the presence of **1** (Scheme 5a). Repeating the same experiments using **3a** as a potential quencher indicated no involvement of such species in the quenching process (see the Supporting Information). The generation of TFCp radicals under the reaction conditions was also confirmed using TEMPO and 1,1-diphenylethylene as radical trapping reagents (Scheme 5b). Thus, we propose that the SET reduction of **1** results in the formation of the TFCp radical **I**, which reacts with **3a**. Oxidation of the thus formed intermediate **II** by the oxidized photocatalyst results in the formation of carbocation **III**, which collapses to the observed product with the concomitant formation of TBSOTf. The quantum yield of this functionalization has been determined through the ferrioxalate actinometry method^30^ (Φ = 0.42; see the Supporting Information for details) suggesting the reduction of **1** to be performed by the photocatalyst, with no considerable involvement of the alternative radical chain manifold. A similar mechanism operates when *N*-(phenyl)pyrrole is employed as a substrate, albeit with a slightly lower quantum yield (Φ = 0.27; see Supporting Information for details).

**Scheme 6:**
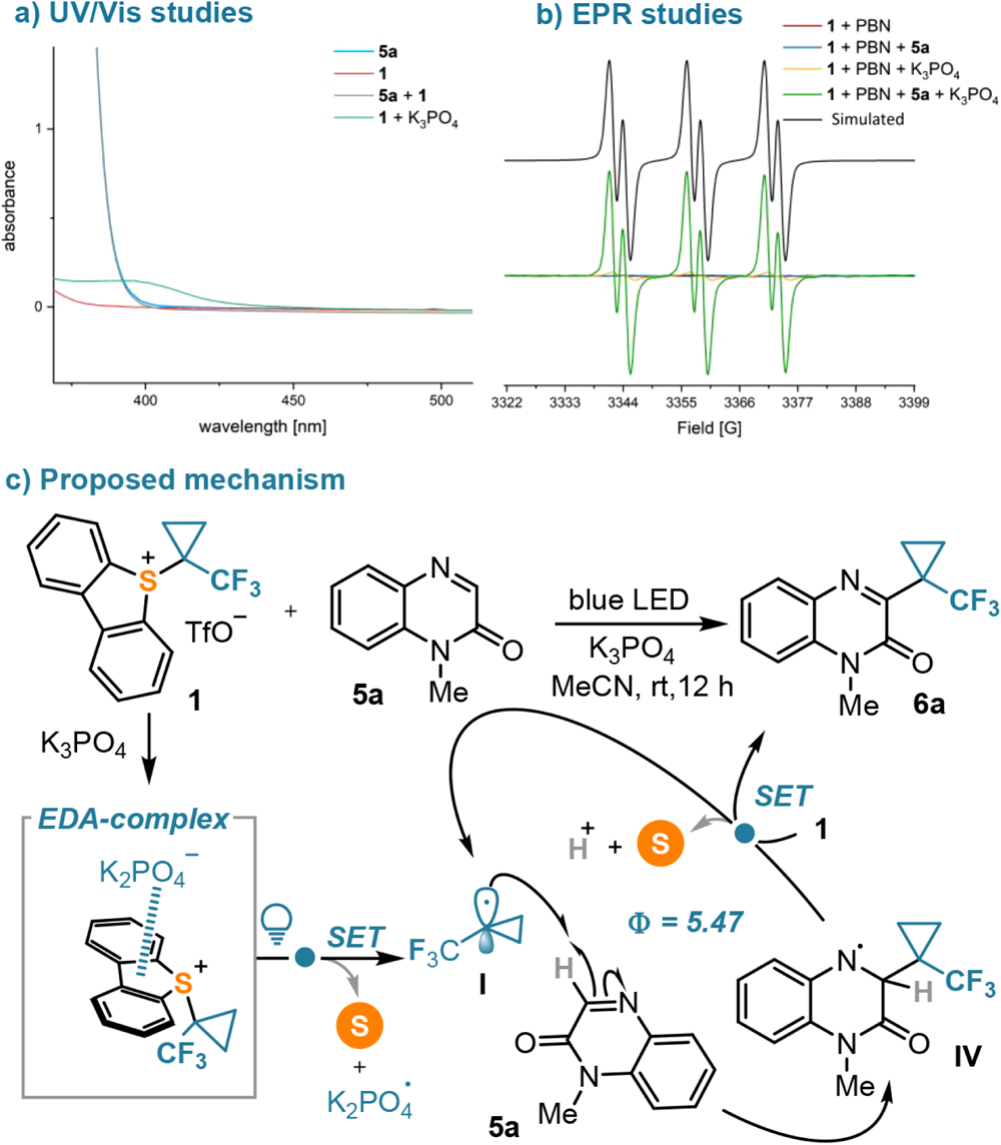
Mechanistic Proposal for the TFCp Transfer to Quinoxalinone **3a**: (a) UV–Vis Spectra; (b) EPR Studies; (c) Proposed Mechanism

The fact that no photocatalyst is necessary for the efficient installation of the TFCp onto quinoxalinones suggests the involvement of electron donor–acceptor (EDA) interactions in this reaction.^31^ For this reason, the UV–vis absorption spectra of **1**, **5a**, and their 1:1 mixture were recorded, but neither the individual partners depicted absorption bands at the irradiation wavelength nor a red shift was observed in the absorption spectra of the mixture. Unexpectedly, a new absorption band centered at 396 nm, with significant tailing in the blue region raised after the addition of K_3_PO_4_ to the sample containing **1**, hinting the potential formation of an EDA complex after triflate/phosphate anion exchange, in which the phosphate anion takes the role of the electron donor (Scheme 6a).

Further evidence supporting the formation of a photoactive EDA complex was harvested via electron paramagnetic resonance (EPR) spectroscopy. The spectra recorded upon irradiation of samples containing **1** and phenyl *N*-*t-*butylnitrone (PBN), or **1**, **5a**, and PBN remained silent (Scheme 6b, red and blue lines, respectively). However, when K_3_PO_4_ was added to the first sample and subsequently irradiated, a weak spectrum centered at *g* = 2.00643 was recorded, which showed hyperfine splitting as a result of hyperfine coupling to one N atom (*a*_N_ = 14.7 G) and one proton (*a*_H_ = 2.1 G) (Scheme 6b, yellow line). Addition of K_3_PO_4_ to the second sample, followed by irradiation, had a much more marked effect. The recorded spectrum had a structure identical to that of the previous one, but it was significantly more intense (Scheme 6b, green line). Putting together this spectroscopic evidence with the information obtained from the substrate scope evaluation studies makes us believe that the generation of TFCp radicals through the photoactivation of the sulfonium-phosphate EDA complex is a rather inefficient process, but enough to initiate a self-propagating radical chain. Actually, the quantum yield for the trifluorocyclopropanation of quinoxalinone **3a** has been determined through the ferrioxalate actinometry method to be 5.47 (see the Supporting Information).^30^

Based on the above-mentioned experiments, we propose the following operating mechanism (Scheme 6c): triflate/phosphate anion exchange in sulfonium salt **1** forms a photoactive EDA complex, which upon irradiation triggers a SET from the phosphate to the sulfonium moiety, delivering the desired TFCp radical **I** and a phosphate radical. Trapping of **I** by **5a** generates radical **IV**, which finally reduces **1** delivering the product **6a** and regenerating **I**. Hydrogen atom transfer from **IV** to the phosphate radical is probably the final step of the radical chain.

## Conclusions

In conclusion, sulfonium salt **1** has been synthesized in multigram scale and identified as a convenient source of TFCp radicals under reductive conditions. Subsequently, two photocatalytic protocols have been implemented to attach the TFCp unit to both aliphatic chains and a broad array of (hetero)arenes via C–H functionalization. Regardless of the method used, the radical cyclopropanation tolerates a range of functional groups, including halogens, alkenes, alkynes, alcohols, ketones, amides, and esters, as well as a broad variety of heterocycles often used in medicinal chemistry. This broad scope, together with the operational ease of the experimental procedures and the existing pressure to replace *tert*-butyl moieties by more metabolically resistant bioisosteres, makes us anticipate broad utility for **1** in future drug development campaigns

## Methods

### Synthesis of **1**

A pressure Schlenk flask was charged with 5-(1-diazo-2,2,2-trifluoroethyl)-5*H*-dibenzo[*b,d*]thiophen-5-ium **2** (3.00 g, 4.36 mmol, 1.0 equiv)^19^ and Rh_2_(esp)_2_ (33.1 mg, 43.6 μmol, 1 mol %). The flask was cooled to −20 °C, and DCM (40 mL) was added slowly. Subsequently, the N_2_ atmosphere was exchanged for ethylene (2.0 bar) using freeze–pump–thaw techniques, and the reaction mixture was kept at −20 °C for 3 d. After consumption of **2**, Et_2_O was added at −20 °C for precipitation. The obtained solid was washed with Et_2_O (2 × 20 mL) and finally dried under high vacuum. Sulfonium salt **1** was obtained as a white solid (2.66 g, 4.03 mmol, 92%).

### General Procedure for the Synthesis of α-Substituted Ketones (**4a**–y)

In a glovebox, a Schlenk flask equipped with a magnetic stir bar was charged with Ir(ppy)_3_ (1.3 mg, 0.2 μmol, 1 mol %), sulfonium salt **1** (0.26 mmol, 1.3 equiv), and the desired silyl enol ether (0.2 mmol, 1.0 equiv). Subsequently, MeCN (4 mL) was added, the flask was sealed, transferred to a photoreactor equipped with blue LED strips (maximum wavelength: 462 nm) and irradiated at 50% intensity for 5 h. Finally, the reaction mixture was diluted with DCM (3 mL) and purified by silica gel column chromatography.

### General Procedure for the Synthesis of (**6a**–w)

In a glovebox, a Schlenk flask equipped with a magnetic stir bar was charged with sulfonium salt **1** (0.2 mmol, 1.0 equiv), the desired substrate (0.4 mmol, 2.0 equiv), and K_3_PO_4_ (0.6 mmol, 3.0 equiv). Subsequently, MeCN (4 mL) was added, and the flask was sealed, transferred to a photoreactor equipped with blue LED strips (maximum wavelength: 462 nm) and irradiated at 50% intensity for 12 h. Finally, the reaction mixture was diluted with DCM (3 mL) and purified by silica gel column chromatography.

## Supporting Information Available

All experimental procedures, optimization studies, mechanistic experiments, characterization of the new compounds, NMR spectra, and crystal data are provided (PDF)
X-ray structure of compound **1** (CIF)
X-ray structure of compound **4a** (CIF)
X-ray structure of compound **6aa** (CIF)
X-ray structure of compound **6b** (CIF)
X-ray structure of compound **6d** (CIF)
X-ray structure of compound **6h** (CIF)
X-ray structure of compound **6m** (CIF)
X-ray structure of compound **4t** (CIF)

## Supplementary Material

cs5c01642_si_001.pdf

cs5c01642_si_002.cif

cs5c01642_si_003.cif

cs5c01642_si_004.cif

cs5c01642_si_005.cif

cs5c01642_si_006.cif

cs5c01642_si_007.cif

cs5c01642_si_008.cif

cs5c01642_si_009.cif

## Dedication

Dedicated to Prof. Franc Meyer on the occasion of his 60th birthday.
