# Supplementary material for: A Photocatalytic Approach to Radical 1-(Trifluoromethyl)cyclopropanation
Source: ACS Catal. 2025 Apr 17;15(9):7232–40. doi: 10.1021/acscatal.5c01642 (PMC12053945; doi:10.1021/acscatal.5c01642)
Supplement: Supplementary file 1 — cs5c01642_si_001.pdf [file cs5c01642_si_001.pdf]

# A Photocatalytic Approach to Radical 1-(Trifluromethyl)cyclopropanation

*Sven Timmann, Moritz T. H. Dilchert, Jörg Dietzel, Verena S. Pöltl, Marc R. Wennekamp, Christopher Golz, and Manuel Alcarazo**

Institut für Organische und Biomolekulare Chemie

Georg-August-Universität Göttingen, Tammannstr 2, 37077 Göttingen (Germany)

Email: manuel.alcarazo@chemie.uni-goettingen.de

# TABLE OF CONTENTS

MATERIALS AND METHODS ... 4

SYNTHESES OF SULFONIUM SALTS 1 and 1-$d_4$ ... 6

OPTIMIZATION OF REACTION CONDITIONS: SILYL-ENOL-ETHERS ... 7

OPTIMIZATION OF REACTION CONDITIONS FOR HETEROARENES: CATALYST-FREE TRANSFORMATION ... 11

OPTIMIZATION OF REACTION CONDITIONS FOR (HETERO)ARENES: Ir-CATALIZED PROCESS ... 15

SYNTHESIS OF SUBSTRATES ... 18

General procedure **A** for the synthesis of silyl-enol ethers (**3d**, **3e**, **3f**, **3i**, **3m**, **3n**, **3q**, **3u**, **3v**, **3w**, **3x**, **3y**, **3z**): ... 19

SYNTHESIS OF α-TFCp SUBSTITUTED KETONES ... 31

General procedure **B** for the synthesis of α-substituted ketones (**4a** – **4y**): ... 31

CATALYST-FREE TRANSFER OF TFCp ... 44

General procedure **C** for the synthesis of (**6a**-**w**): ... 44

PHOTOREDOX CATALYTIC TRANSFER TO (HETERO)AROMATIC COMPOUNDS ... 57

General procedure **D** for the synthesis of (**6x**-**6ai**): ... 57

TRANSFER OF $D_4$-LABELLED TFCp ... 64

CYCLIC VOLTAMMETRY MEASUREMENT ... 68

STERN-VOLMER QUENCHING EXPERIMENTS[25] ... 69

Stern-Volmer Quenching Experiments of Sulfonium salt **1** ... 69

Stern-Volmer Quenching Experiments of Substrate **3a** ... 70

Stern-Volmer Quenching Experiments of substrate **6x** ... 72

QUANTUM YIELD DETERMINATIONS ... 74

Determination of ferrioxalate actinometry ... 74

Quantum yield determination for silyl-enol-ether substrates: ... 77

Quantum yield determination for the catalyst-free transformation of heteroaromatic substrates: 78

Quantum yield determination for the photoredox-catalytic transformation of heteroaromatic substrates: ... 79

RADICAL TRAPPING EXPERIMENTS ... 80

UV/Vis STUDIES ... 82

ELECTRON PARAMAGNETIC RESONANCE (EPR) STUDIES ... 83

DIFFERENTIAL SCANNING CALORIMETRY (DSC) OF SULFONIUM SALTS ... 90

X-RAY CRYSTALLOGRAPHIC ANALYSIS ... 92

Refinement details for **1** ... 93

Refinement details for **4a** ... 94

Refinement details for **4t** ... 95

Refinement details for **6b** .......... 96
Refinement details for **6d** .......... 97
Refinement details for **6h** .......... 98
Refinement details for **6m** .......... 99
Refinement details for **6aa** .......... 100
SPECTROSCOPIC DATA .......... 101
COMPUTATIONAL STUDIES .......... 230
REFERENCES .......... 231

## MATERIALS AND METHODS

Unless stated otherwise, all reactions were carried out using pre-dried glassware under an inert atmosphere (nitrogen or argon) using standard Schlenk techniques, or in a MBraun UNIlab plus glovebox. After quenching the reaction mixtures were concentrated under reduced pressure performed by rotary evaporation at 25–40 °C at an appropriate pressure. Purified compounds were further dried under high vacuum if appropriate. Yields refer to purified and spectroscopically pure compounds, unless otherwise stated.

**Solvents:** Dry and degassed solvents (THF, dichloromethane, dichloroethane, toluene, diethyl ether, pentane, acetonitrile) were obtained from a MBraun Solvent Purification System (MB-SPS-800) or by distillation over the appropriate drying agent and stored under a protective gas atmosphere.

**Chromatography:** Thin layer chromatography (TLC) was performed using polygram SIL G/UV254 TLC plates from Macherey Nagel and visualized by UV irradiation and/or phosphomolybdic acid or $KMnO_4$ dip. Flash column chromatography was performed using Macherey Nagel 60 (40-63 µm) silica gel.

**Starting materials:** Commercially available reagents were purchased from *Acros Organics, ABCR, Alfa Aesar, BLD Pharmatech, Sigma Aldrich* and *TCI*, and used as received.

Starting materials already described were synthesized according to literature procedure: 5-(1-diazo-2,2,2-trifluoroethyl)-5*H*-dibenzo[*b,d*]thiophen-5-ium **2**[1]; ((1H-inden-3-yl)oxy)trimethylsilane **3b**[2];1-(benzo[*d*]thiazol-2-yl)ethan-1-one **S1**[3]; 1-(1-methyl-1*H*-indazol-3-yl)ethan-1-one **S2**[4].

**NMR:** Spectra were recorded on Bruker Avance Neo 600, Avance Neo 400, Avance III HD 400, Avance III 400 or Avance III HD 300 spectrometers. $^1$H and $^{13}$C chemical shifts (δ) are reported in ppm relative to TMS using the solvent signals as reference in $CDCl_3$ ($^1$H: 7.26 ppm, $^{13}$C: 77.16 ppm) or $CD_3CN$ ($^1$H: 1.94 ppm, $^{13}$C: 118.26 ppm). Coupling constants (*J*) are given in Hertz (Hz). Data is reported as follows: s = singlet, d = doublet, t = triplet, q = quartet, m = multiplet, br = broad; coupling constants in Hz; integration.

**HRMS:** Spectra were recorded using *Bruker Daltonik maXis Q-TOF* (ESI), *Bruker Daltonik micrOTOF* (ESI), *Thermo Scietific LTQ Orbitrap XL* (ESI), *Thermo Scietific Exactive GC-Orbitrap-MS* (EI) or *Jeol AccuTOF* (EI) instruments. Dimensionless mass-to-charge ratios (*m*/*z*) are given.

**IR:** Infrared spectra were recorded on a FT/IR-4600 spectrometer and reported in wavenumbers ($cm^{-1}$).

**UV/Vis:** Recorded on a Jasco V-630 spectrometer.

**Fluorescence spectra:** Recorded on an Edinburgh Instrument FS5 Spectrofluorometer.

**EPR:** EPR-spectra were measured on a Bruker EMX mikro X-Band EPR from BRUKER Biospin with the Bruker Xenon Software. The spectra were measured in dry and degassed solvents at room temperature unless stated otherwise.

**Melting point:** Melting points were measured with a Büchi M-560 apparatus with a heating rate of 5°C/min.

**Differential Scanning Calorimetry (DSC):** Recorded on a Mettler Toledo TGA/DSC 3+ STARe System.

Method for the DSC measurement of sulfonium salts **1** and **1-d$_4$**: from 25.0 to 500.0 °C, 15.0 °C/min., working gas as indicated for each measurement (50.0 mL/min.).

**Specific rotations:** Specific rotations were collected using Jasco P-2000 polarimeter at the stated temperature under a Na/Hg lamp, $\lambda$ = 589 nm ($c$ in g/mL).

**Single crystal X-ray diffraction analysis:** Data collection was done on two dual source equipped *Bruker D8 Venture* four-circle-diffractometer from *Bruker AXS GmbH*; used X-ray sources: microfocus *IµS 2.0* Cu/Mo and microfocus *IµS 3.0* Ag/Mo from *Incoatec GmbH* with mirror optics *HELIOS* and single-hole collimator from *Bruker AXS GmbH;* used detector: *Photon III CE14* (Cu/Mo) and *Photon III HE* (Ag/Mo) from *Bruker AXS GmbH*.

Used programs: *APEX4 Suite* (v2022.1-1) for data collection and therein integrated programs *SAINT* V8.40A (Integration) und *SADABS* 2016/2 (Absorption correction) from *Bruker AXS GmbH*; structure solution was done with *SHELXT*, refinement with *SHELXL*-2018/3[5] or with *olex.refine* and therein the *NoSpherA2* plugin to obtain the aspherical atomic structure factors.[6] OLEX2[7] and FinalCif were used for data finalization (D. Kratzert, *FinalCif*, *V113*, https://dkratzert.de/finalcif.html).

Special Utilities: *SMZ1270* stereomicroscope from *Nikon Metrology GmbH* was used for sample preparation; crystals were mounted on *MicroMounts* or *MicroLoops* from *MiTeGen* in NVH oil; for sensitive samples the *X-TEMP 2 System* was used for picking of crystals.[8]; crystals were cooled to given temperature with *Cryostream 800* from *Oxford Cryosystems.*

**Photoreactor:** 5m dimmable LED strip, 460-465nm wavelength (blue), maximum rated power 72W, dimmed to 28W (dimmer, 24V transformer). Supplier: revoArt GmbH.

LED strip was mounted on a cylindrical metal case (23 cm diameter) to control diffuse light scattering. Stirring was achieved by magnetic stirrer. Constant temperature was ensured by ventilation slots and a 20cm computer fan.

## SYNTHESES OF SULFONIUM SALTS 1 and 1-d$_4$

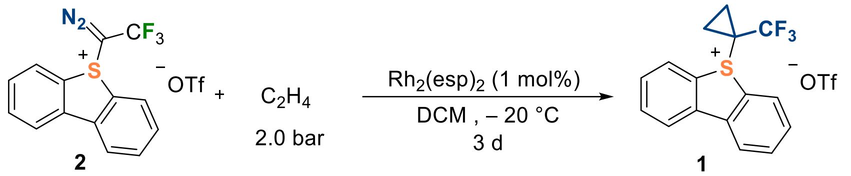


A pressure Schlenk flask was charged with 5-(1-diazo-2,2,2-trifluoroethyl)-5H-dibenzo[b,d]thiophen-5-ium **2** (3.00 g, 4.36 mmol, 1.0 equiv) and $Rh_2(esp)_2$ (33.1 mg, 43.6 µmol, 1 mol%). The flask was cooled to -20 °C and DCM (40 mL) was added slowly. Subsequently, the $N_2$ atmosphere was exchanged to ethylene (2.0 bar) using freeze-pump-thaw techniques, and the reaction mixture kept at -20 °C for 3 d. After consumption of 2, $Et_2O$ was added at -20 °C for precipitation. The obtained solid was washed with $Et_2O$ (2 × 20 mL) and finally dried under high vacuum. Sulfonium salt **1** was obtained as a white solid (2.66 g, 4.03 mmol, 92%).

Single crystals suitable for X-ray diffraction were obtained by slow diffusion of $Et_2O$ into a solution of **1** in MeCN.

**$^1$H NMR** (400 MHz, $CD_3CN$): $\delta$ = 8.33−8.26 (m, 4H), 8.00 (td, $J$ = 7.7, 1.0 Hz, 2H), 7.86−7.79 (m, 2H), 2.10 (m, 2H), 2.02−1.97 (m, 2H) ppm.

**$^{13}$C{$^1$H} NMR** (101 MHz, $CD_3CN$) δ = 140.9, 136.3, 132.5, 129.9, 127.0, 125.7, 124.0 (q, $J$ = 277.1 Hz), 121.7 (q, $J$ = 322.2 Hz), 38.2 (q, $J$ = 36.8 Hz), 13.0 (q, $J$ = 1.4 Hz) ppm.

**$^{19}$F NMR** (377 MHz, $CD_3CN$) δ = − 64.8, −79.3 ppm.

**IR** (ATR, neat) $\tilde{\nu}$ = 1453, 1354, 1260, 1224, 1155, 1125, 1065, 1029, 960, 818, 756, 731, 708, 635, 613, 573, 517, 503, 493, 486, 465, 449, 440, 431, 420, 404 cm$^{-1}$.

**HRMS-ESI (m/z)** calculated for $C_{16}H_{12}F_3S$ [M−OTf]$^+$: 293.0606; found: 293.0608.

**Melting point:** 170 °C decomp.

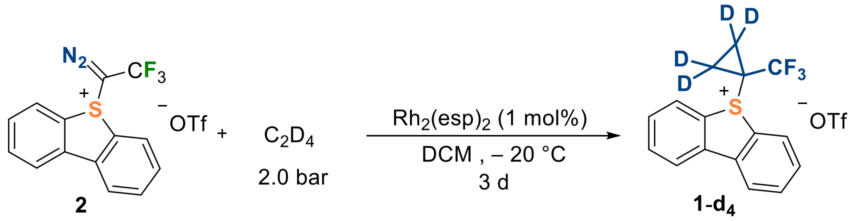


Prepared from **2** (1.63 g, 3.68 mmol, 1.0 equiv) and $Rh_2(esp)_2$ (27.9 g, 36.9 µmol, 1 mol%) in identical way as **1** but employing d$_4$-ethylene. White solid (1.49 g, 3.34 mmol, 91%).

**$^1$H NMR** (500 MHz, $CD_3CN$): $\delta$ = 8.34−8.27 (m, 4H), 8.00 (td, $J$ = 7.7, 1.1 Hz, 2H), 7.82 (ddd, $J$ = 8.5, 7.5, 1.2 Hz, 2H) ppm.

**$^{13}$C{$^1$H} NMR** (101 MHz, $CD_3CN$) δ = 140.8, 136.3, 132.5, 129.9, 127.0, 125.7, 123.9 (q, $J$ = 277.8 Hz), 122.1 (q, $J$ = 321.7 Hz), 37.7 (q, $J$ = 36.9 Hz), 12.5 (p, $J$ = 26.2 Hz) ppm.

**$^{19}$F NMR** (377 MHz, $CD_3CN$) δ = − 64.8, −79.3 ppm.

**$^{2}$H NMR** (92 MHz, $CH_3CN$): $\delta$ = 2.12 (brs, 2D), 1.99 (brs, 2D) ppm.

**IR** (ATR, neat) $\tilde{\nu}$ = 1453, 1335, 1260, 1223, 1157, 1029, 960, 881, 758, 705, 635, 572, 514, 487, 462, 435, 420 $cm^{-1}$.

**HRMS-ESI (m/z)** calculated for $C_{16}H_8D_4F_3S$ $[M-OTf]^+$: 297.0857; found: 297.0885.

**Melting point:** 167 °C decomp.

## OPTIMIZATION OF REACTION CONDITIONS: SILYL-ENOL-ETHERS

A Schlenk flask equipped with a magnetic stir bar was charged with silyl-enol-ether **3a**, sulfonium salt **1**, base and photocatalyst inside a nitrogen-filled glovebox. Solvent was added and the flask was sealed. The reaction mixture was transferred to a photoreactor equipped with blue LED strips (maximum wavelength: 462 nm) and irradiated at 50% intensity. Samples of the reaction mixture were taken, diluted with deuterated acetonitrile and analyzed by $^{19}$F NMR. The NMR-yields were determined by integration of the peak corresponding to the $CF_3$-signal of the product ((282 MHz, $CD_3CN$) δ = − 68.3 ppm) and the triflate signal ((282 MHz, $CD_3CN$) δ = −79.3 ppm), which was used as internal standard. All reactions were carried out at 0.05 mmol scale.

### Screening of silyl-protecting groups:

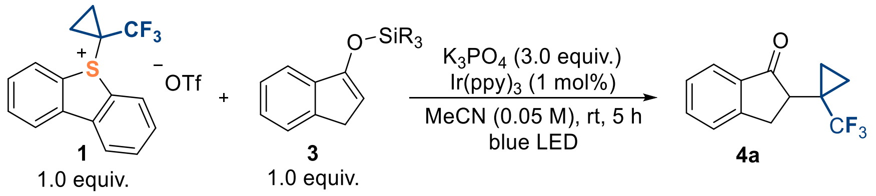


| Entry | $SiR_3$ Protecting group | $^{19}$F NMR yield[a] |
|---|---|---|
| 1 | TBS | 60% |
| 2 | TMS | 28% |
| 3 | TES | 42% |

[a]Yield determined by $^{19}$F NMR using the triflate signal as internal standard.

**Screening of photocatalysts:**

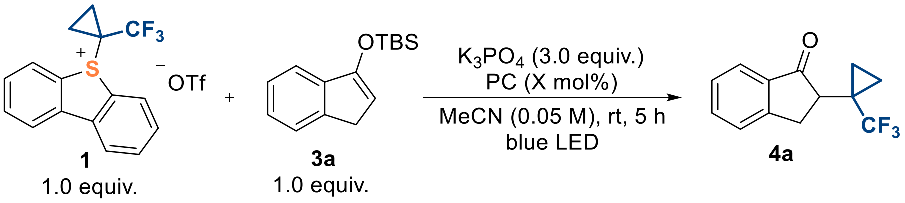


| Entry | Photocatalyst (x mol%) | $^{19}$F NMR yield[a] |
|---|---|---|
| 1 | $Ir(ppy)_3$ (1) | 60% |
| 2 | 4CzIPN (5) | 22% |
| 3 | PTH (5) | 16% |
| 4 | $(C_6H_4Br)_3N$ (10) | n.d. |
| 5 | - | no reaction[b] |

n.d. = not detected; [a]Yield determined by $^{19}$F NMR using the triflate signal as internal standard; [b]Reaction time of 20 h.

**Screening of bases:**

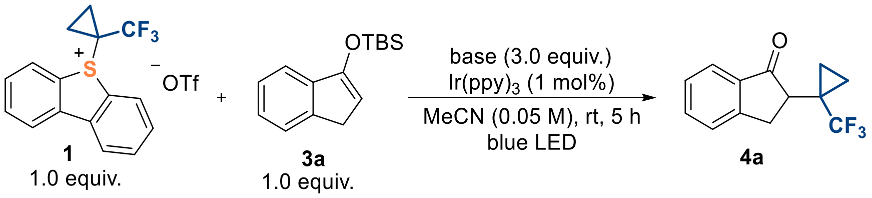


| Entry | Base (3.0 equiv.) | $^{19}$F NMR yield[a] |
|---|---|---|
| 1 | $Na_2CO_3$ | 38% |
| 2 | $K_2CO_3$ | 64% |
| 3 | $Cs_2CO_3$ | 28% |
| 4 | $K_3PO_4$ | 60% |
| 5 | $NaHCO_3$ | 62% |
| 6 | Pyridine | 48% |
| 7 | - | 74% |
| 8 | $K_2CO_3$ (3.0 equiv.)/2,6-Lutidine (1.5equiv.) | 62% |

[a]Yield determined by $^{19}$F NMR using the triflate signal as internal standard.

**Screening of reagent excesses:**

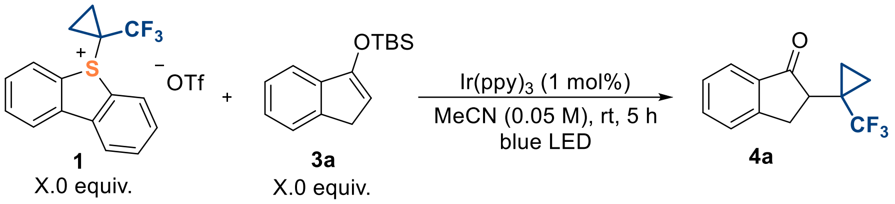


| Entry | Sulfonium salt 1 (equiv.) | Substrate (equiv.) | $^{19}F$ NMR yield[a] |
|---|---|---|---|
| 1 | 1.0 | 2.0 | 69% |
| 2 | 1.0 | 1.5 | 72% |
| 3 | 1.0 | 1.0 | 74% |
| 4 | 1.3 | 1.0 | 89% |

[a]Yield determined by $^{19}F$ NMR using the triflate signal as internal standard.

**Screening of solvents:**

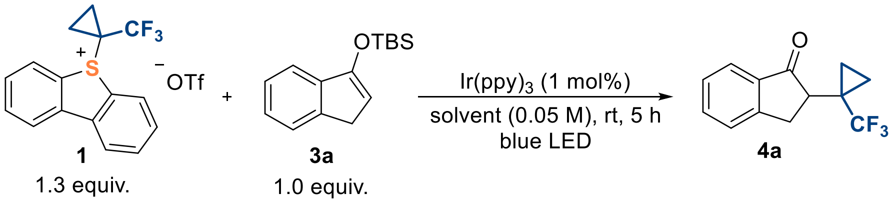


| Entry | Solvent | $^{19}F$ NMR yield[a] |
|---|---|---|
| 1 | MeCN | 89% |
| 2 | DCM | 35% |
| 3 | DCE | 37% |
| 4 | THF | 9% |

[a]Yield determined by $^{19}F$ NMR using the triflate signal as internal standard.

**Screening of substrate concentration:**

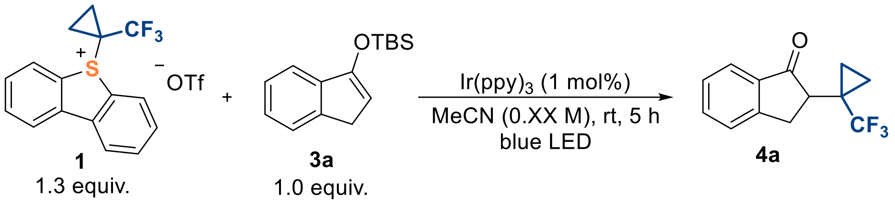


| Entry | Concentration | $^{19}F$ NMR yield[a] |
|---|---|---|
| 1 | 0.05 M | 89%(80%)[b] |
| 2 | 0.1 M | 51% |
| 3 | 0.025 M | 68% |

[a]Yield determined by $^{19}F$ NMR using the triflate signal as internal standard; [b]Isolated yield in parenthesis at 0.2 mmol scale.

**Other control experiments:**

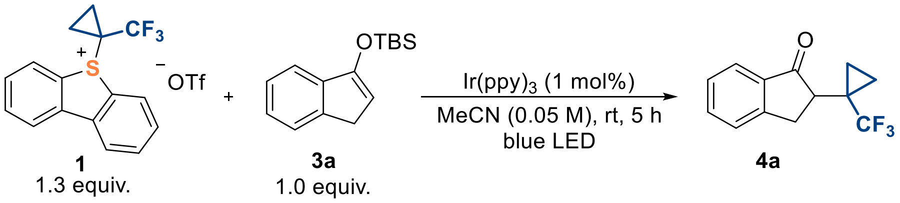


| Entry | Deviation from standard conditions | $^{19}F$ NMR yield[a] |
|---|---|---|
| 1 | No light | No reaction |
| 2 | No photocatalyst | No reaction[b] |
| 3 | TEMPO (5.0 equiv.) | n.d. |

n.d. = not detected; [a]Yield determined by $^{19}F$ NMR using the triflate signal as internal standard; Reaction time 20 h.

## OPTIMIZATION OF REACTION CONDITIONS FOR HETEROARENES: CATALYST-FREE TRANSFORMATION

A Schlenk flask equipped with a magnetic stir bar was charged with 1-methylquinoxalin-2(1*H*)-one **5a**, sulfonium salt **1** and base inside a nitrogen-filled glovebox. Solvent was added and the flask was sealed. The reaction mixture was transferred to a photoreactor equipped with blue LED strips (maximum wavelength: 462 nm) and irradiated at 50% intensity until completion. A sample of the reaction mixture was taken, diluted with deuterated acetonitrile and analyzed by $^{19}F$ NMR. The NMR-yield obtained was determined by integration of the peak corresponding to the $CF_3$-signal of the product ((282 MHz, $CD_3CN$) δ = − 67.7 ppm) and the triflate signal ((282 MHz, $CD_3CN$) δ = − 79.3 ppm) which is used as internal standard. All reactions were carried out at 0.05 mmol scale.

**Screening of bases:**

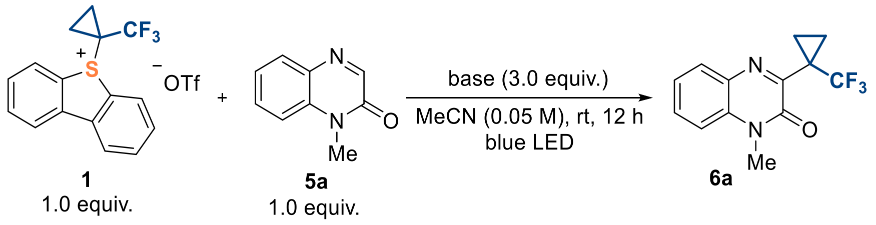


| Entry | Base (3.0 equiv.) | $^{19}F$ NMR yield[a] |
|---|---|---|
| 1 | $Na_2CO_3$ | traces |
| 2 | $K_2CO_3$ | traces |
| 3 | $Cs_2CO_3$ | 43% |
| 4 | $K_3PO_4$ | 54% |
| 5 | $NaHCO_3$ | traces |
| 6 | $KHCO_3$ | traces |
| 7 | *N*-methyl- morpholine | traces |
| 8 | - | traces |

[a]Yield determined by $^{19}F$ NMR using the triflate signal as internal standard.

**Screening of reagent excesses:**

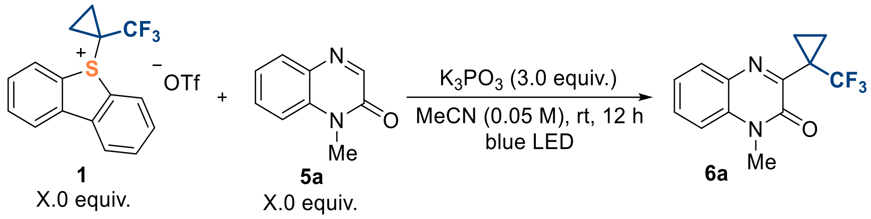


| Entry | Sulfonium salt 1 (equiv.) | Substrate (equiv.) | $^{19}F$ NMR yield[a] |
|---|---|---|---|
| 1 | 1.0 | 3.0 | 67% |
| 2 | 1.0 | 5.0 | 63% |
| 3 | 1.0 | 2.0 | 73% |
| 4 | 1.0 | 1.0 | 54% |
| 5 | 1.3 | 1.0 | 50% |

[a]Yield determined by $^{19}F$ NMR using the triflate signal as internal standard.

**Screening of solvents:**

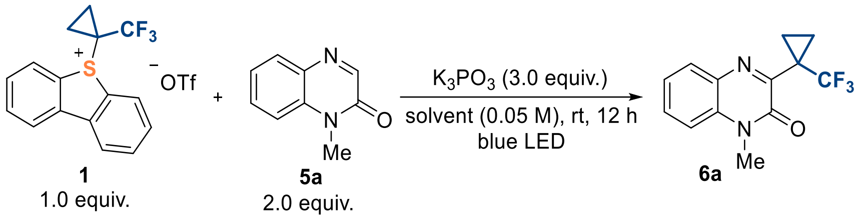


| Entry | Solvent | $^{19}F$ NMR yield[a] |
|---|---|---|
| 1 | MeCN | 73% |
| 2 | DCM | 26% |
| 3 | DMSO | n.d. |
| 4 | THF | 5%[b] |
| 5 | 1,4-dioxane | 11%[c] |
| 6 | 1,2-dichlorobenzene | 35% |

n.d. = not detected; [a]Yield determined by $^{19}F$ NMR using the triflate signal as internal standard; [b]86% cyclopropane; [c]62% cyclopropane.

**Screening of concentration:**

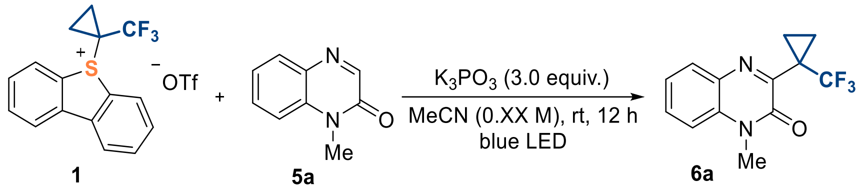


**1** 1.0 equiv. + **5a** 2.0 equiv. → **6a**

| Entry | Concentration | $^{19}$F NMR yield[a] |
|---|---|---|
| 1 | 0.05 M | 73% |
| 2 | 0.1 M | 64% |
| 3 | 0.025 M | 47% |

[a]Yield determined by $^{19}$F NMR using the triflate signal as internal standard.

**Screening of base equivalents:**

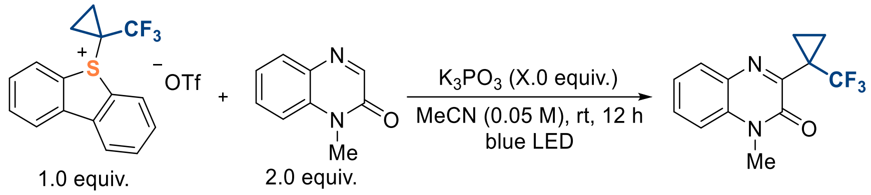


1.0 equiv. 2.0 equiv.

| Entry | $K_3PO_4$ (X.0 equiv.) | $^{19}$F NMR yield[a] |
|---|---|---|
| 1 | 1.0 | 65% |
| 2 | 2.0 | 61% |
| 3 | 3.0 | 73%(66%)[b] |
| 4 | 5.0 | 61% |

[a]Yield determined by $^{19}$F NMR using the triflate signal as internal standard; [b]isolated yield in parenthesis at 0.2 mmol scale.

**Other control experiments:**

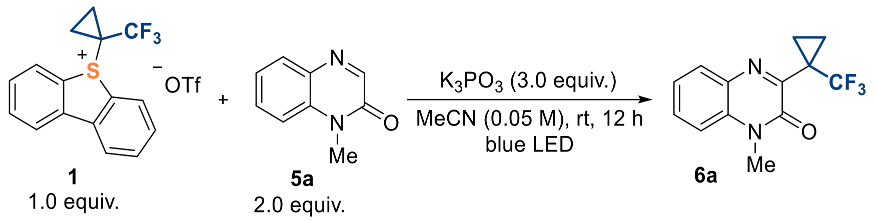


| Entry | Deviation from standard conditions | $^{19}$F NMR yield[a] |
|---|---|---|
| 1 | none | 73% |
| 2 | No light | no reaction |
| 3 | Ir(ppy)$_3$ (1 mol%); 1.0 equiv. **5a** | 55% |
| 6 | TEMPO (5.0 equiv.) | n.d. |

n.d. = not detected; [a]Yield determined by $^{19}$F NMR using the triflate signal as internal standard.

## OPTIMIZATION OF REACTION CONDITIONS FOR (HETERO)ARENES: Ir-CATALIZED PROCESS

A Schlenk flask equipped with a magnetic stir bar was charged with 1-phenyl-1*H*-pyrrole **5x**, sulfonium salt **1**, base and the photocatalyst inside a nitrogen-filled glovebox. Solvent was added and the flask was sealed. The reaction mixture was transferred to a photoreactor equipped with blue LED strips (maximum wavelength: 462 nm) and irradiated at 50% intensity. A sample of the reaction mixture was taken, diluted with deuterated acetonitrile and analyzed by $^{19}F$ NMR. The NMR-yield obtained was determined by integration of the peak corresponding to the $CF_3$-signal of the product ((282 MHz, $CD_3CN$) δ = − 69.1 ppm) and the triflate signal ((282 MHz, $CD_3CN$) δ = − 79.3 ppm) which is used as internal standard. All reactions were carried out at 0.05 mmol scale.

**Screening of bases:**

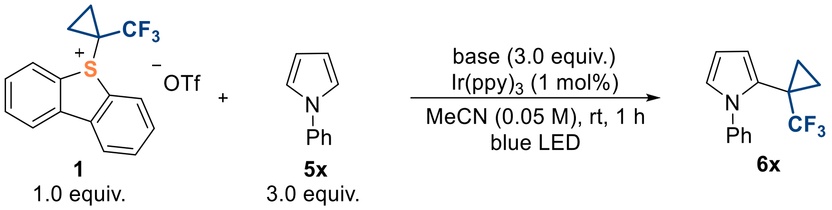


| Entry | Base (3.0 equiv.) | $^{19}F$ NMR yield[a] |
|---|---|---|
| 1 | $Na_2CO_3$ | 52% |
| 2 | $K_2CO_3$ | 43% |
| 3 | $Cs_2CO_3$ | 70% |
| 4 | $K_3PO_4$ | 62% |
| 5 | $NaHCO_3$ | 58% |
| 6 | $K_2HPO_4$ | 60% |
| 7 | - | 25%[b] |

[a]Yield determined by $^{19}F$ NMR using the triflate signal as internal standard; [b] 50% conversion.

**Screening of substrate excesses:**

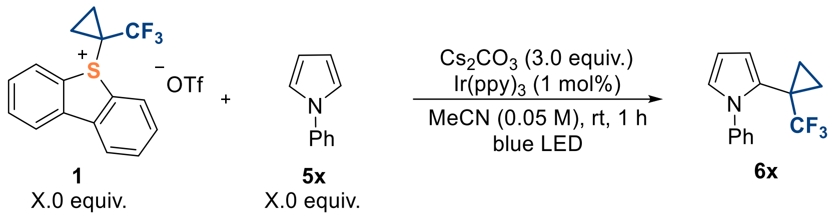

| Entry | Sulfonium salt 1 (equiv.) | Substrate (equiv.) | $^{19}$F NMR yield[a] |
|---|---|---|---|
| 1 | 1.0 | 3.0 | 70% |
| 2 | 1.0 | 5.0 | 70% |
| 3 | 1.0 | 2.0 | 52% |
| 4 | 1.0 | 1.5 | 40% |
| 5 | 1.3 | 1.0 | 32% |

[a]Yield determined by $^{19}$F NMR using the triflate signal as internal standard.

**Screening of reaction concentration:**

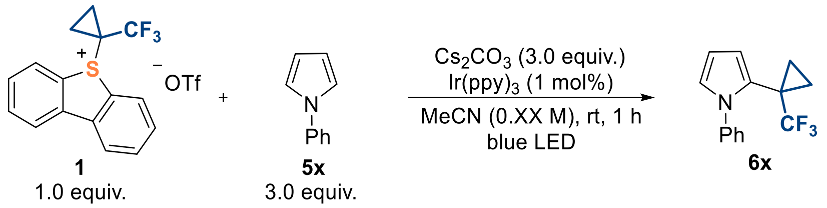

| Entry | Concentration | $^{19}$F NMR yield[a] |
|---|---|---|
| 1 | 0.05 M | 70% |
| 2 | 0.1 M | 77%(65%)[b] |
| 3 | 0.025 M | 64% |

[a]Yield determined by $^{19}$F NMR using the triflate signal as internal standard; [b]Isolated yield in parenthesis at 0.2 mmol scale.

**Other control experiments:**

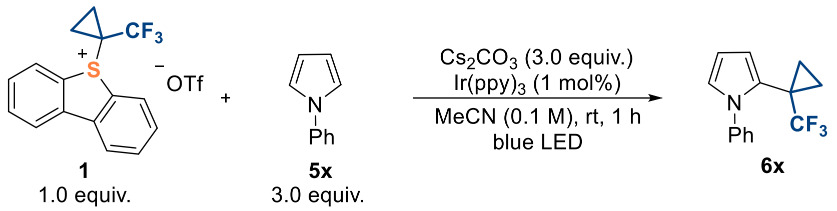


| Entry | Deviation from standard conditions | $^{19}$F NMR yield[a] |
|---|---|---|
| 1 | No light | No reaction |
| 2 | No photocatalyst | 9%[b] |
| 3 | TEMPO (5.0 equiv.) | n.d. |

n.d. = not detected; [a]Yield determined by $^{19}$F NMR using the triflate signal as internal standard; [b]Elongated reaction time of 20 h.

## SYNTHESIS OF SUBSTRATES

**Silyl-enol-ether substrates:**

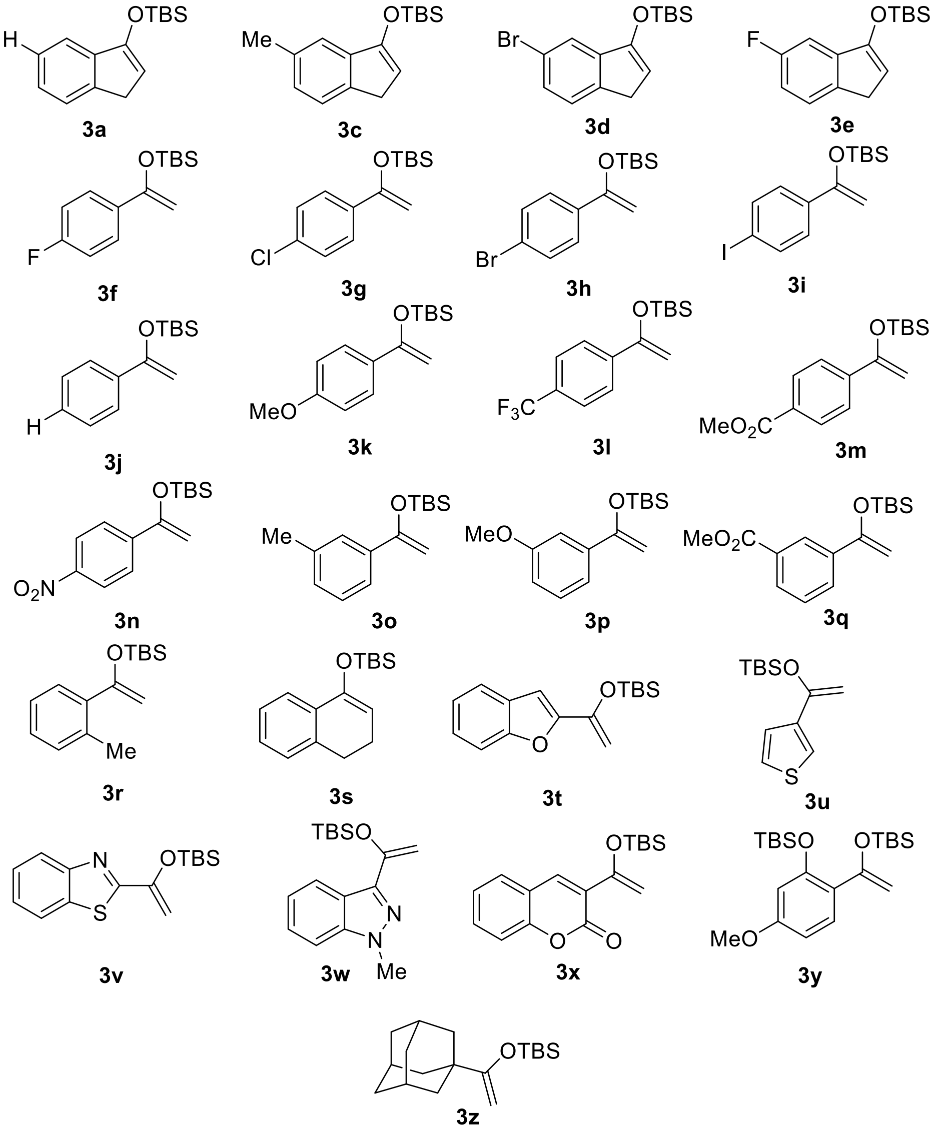


**Figure S1**: Scope of substrates used for the photoredox-catalyzed transfer of the TFCp to silyl-enol-ethers.

Substrates **3a**[9], **3c**[9], **3g**[10], **3h**[11], **3j**[12], **3k**[10], **3l**[12], **3o**[12], **3p**[13], **3r**[12], **3s**[9], **3t**[12] were prepared according to literature procedure.

General procedure **A** for the synthesis of silyl-enol ethers (**3d**, **3e**, **3f**, **3i**, **3m**, **3n**, **3q**, **3u**, **3v**, **3w**, **3x**, **3y**, **3z**):

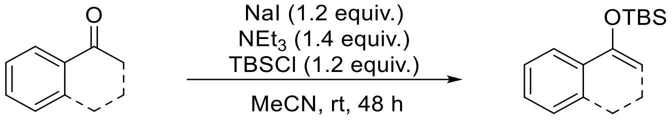


A Schlenk flask equipped with a magnetic stir bar was charged with NaI (1.2 equiv.), TBSCl (1.2 equiv.) and the desired ketone (1.0 equiv.). MeCN (0.2 M) and $NEt_3$ (1.4 equiv.) were subsequently added, and the reaction mixture stirred at ambient temperature for 48 h. Finally, the solvent was removed under reduced pressure, and the resulting residue extracted with hexane. The hexane extracts were concentrated, and when needed subjected to column chromatography on passivated silica gel. Otherwise, they were used without further purification.

Synthesis of **3d**:

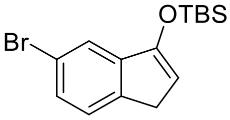


Prepared following general procedure A from 6-bromo-2,3-dihydro-1*H*-inden-1-one (350.1 mg, 1.66 mmol, 1.0 equiv.), NaI (298.3 mg, 1.99 mmol, 1.2 equiv.), $Et_3N$ (322 µL, 234.9 mg, 2.32 mmol, 1.4 equiv.) and *tert*-butylchlorodimethylsilane (299.9 mg, 1.99 mmol, 1.2 equiv.). The desired compound was purified by column chromatography on passivated silica gel eluting with hexane and was obtained as a colorless liquid (381.5 mg, 1.17 mmol, 71%).

**$^1$H NMR** (300 MHz, $CDCl_3$): δ = 7.47 (d, *J* = 1.8 Hz, 1H), 7.32 (dd, *J* = 7.9, 1.9 Hz, 1H), 7.24 (t, *J* = 7.9 Hz, 2H), 5.43 (t, *J* = 2.5 Hz, 1H), 3.22 (d, *J* = 2.4 Hz, 2H), 1.02 (s, 9H), 0.25 (s, 6H) ppm.

**$^{13}$C{$^1$H} NMR** (101 MHz, $CDCl_3$) δ = 153.0, 144.3, 141.5, 128.0, 125.3, 121.5, 120.4, 107.3, 33.8, 25.9, 18.4, − 4.6 ppm.

**HRMS-ESI (m/z)** calculated for $C_{15}H_{21}BrOSi$ $[M+H]^+$: 325.0618; found: 325.0611.

**IR** (ATR, neat) $\tilde{\nu}$ = 2955, 2928, 2884, 2857, 1769, 1598, 1568, 1471, 1460, 1415, 1389, 1361, 1336, 1300, 1252, 1240, 1216, 1205, 1176, 1135, 1089, 1051, 1005, 975, 939, 868, 838, 781, 755, 685, 664, 628, 587, 554, 525, 516, 508, 497, 472, 455, 445, 440, 430, 422, 417 $cm^{-1}$.

Synthesis of **3e**:

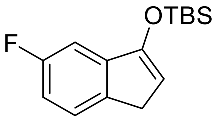


Prepared following general procedure A from 6-bromo-2,3-dihydro-1*H*-inden-1-one (250.2 mg, 1.66 mmol, 1.0 equiv.), NaI (300.1 mg, 2.00 mmol, 1.2 equiv.), $Et_3N$ (324 µL, 235.9 mg, 2.33 mmol, 1.4 equiv.) and *tert*-butylchlorodimethylsilane (301.2 mg, 2.00 mmol, 1.2 equiv.). The desired compound was purified by column chromatography on passivated silica gel eluting with hexane and was obtained as a colorless liquid (340.6 mg, 1.29 mmol, 77%).

**$^{1}$H NMR** (400 MHz, $CDCl_3$): δ = 7.29 (dd, *J* = 8.1, 4.9 Hz, 1H), 7.04 (dd, *J* = 8.7, 2.5 Hz, 1H), 6.89 (ddd, *J* = 9.5, 8.1, 2.5 Hz, 1H), 5.48 (t, *J* = 2.4 Hz, 1H), 3.37−3.09 (m, 2H), 1.02 (s, 9H), 0.25 (s, 6H) ppm.

**$^{13}$C{$^{1}$H} NMR** (101 MHz, $CDCl_3$) δ = 162.5 (d, *J* = 241.9 Hz), 153.3 (d, *J* = 3.3 Hz), 144.1 (d, *J* = 9.1 Hz), 138.0 (d, *J* = 2.5 Hz), 124.6 (d, *J* = 9.0 Hz), 111.9 (d, *J* = 23.3 Hz), 108.0, 105.5 (d, *J* = 23.6 Hz), 33.5, 25.8, 18.4, − 4.6 ppm.

**$^{19}$F NMR** (377 MHz, $CDCl_3$) δ = − 117.5 (td, *J* = 9.1, 4.8 Hz) ppm.

**HRMS-EI (m/z)** calculated for $C_{15}H_{21}FOSi$ [M]$^{\cdot}$: 264.1340; found: 264.1336.

**IR** (ATR, neat) $\tilde{\nu}$ = 2955, 2929, 2888, 2857, 1771, 1610, 1581, 1507, 1471, 1443, 1404, 1390, 1362, 1347, 1311, 1270, 1252, 1207, 1161, 1111, 1059, 1006, 975, 927, 857, 838, 801, 781, 732, 677, 641, 616, 574, 516, 504, 496, 488, 476, 469, 460, 451, 445, 437, 432, 425, 417, 412, 406 $cm^{-1}$.

Synthesis of **3f**:

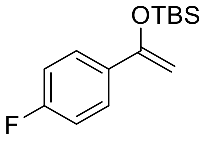


Prepared following general procedure A from 1-(4-fluorophenyl)ethan-1-one (250.1 mg, 1.81 mmol, 1.0 equiv.), NaI (325.5 mg, 2.17 mmol, 1.2 equiv.), $Et_3N$ (351 µL, 256.4 mg, 2.53 mmol, 1.4 equiv.) and *tert*-butylchlorodimethylsilane (327.3 mg, 2.17 mmol, 1.2 equiv.). The desired compound was purified by column chromatography on passivated silica gel eluting with hexane and was obtained as a colorless liquid (318.1 mg, 1.26 mmol, 70%).

**$^{1}$H NMR** (300 MHz, $CDCl_3$): δ = 7.61−7.54 (m, 2H), 7.04−6.95 (m, 2H), 4.81 (d, *J* = 1.8 Hz, 1H), 4.39 (d, *J* = 1.8 Hz, 1H), 1.00 (s, 9H), 0.21 (s, 6H) ppm.

**$^{13}$C{$^{1}$H} NMR** (101 MHz, $CDCl_3$) δ = 162.9 (d, *J* = 247.2 Hz), 155.3, 134.1 (d, *J* = 3.3 Hz), 127.2 (d, *J* = 8.1 Hz), 115.0 (d, *J* = 21.5 Hz), 90.7 (d, *J* = 1.7 Hz), 26.0, 18.5, − 4.5 ppm.

**$^{19}$F NMR** (282 MHz, $CDCl_3$) δ = − 114.1 ppm.

**HRMS-ESI (m/z)** calculated for $C_{14}H_{21}FOSi$ [M-H]$^{\cdot}$: 251.1262; found: 251.1257.

**IR** (ATR, neat) $\tilde{\nu}$ = 2956, 2929, 2885, 2858, 1771, 1616, 1604, 1559, 1507, 1471, 1463, 1404, 1390, 1361, 1311, 1294, 1281, 1253, 1231, 1156, 1114, 1097, 1011, 1002, 938, 829, 809, 779, 727, 685, 661, 612, 573, 537, 507, 499, 495, 488, 475, 468, 458, 451, 447, 437, 426, 417, 412, 407 $cm^{-1}$.

Synthesis of **3i**:

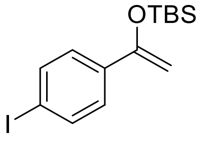


Prepared following general procedure A from 1-(4-iodophenyl)ethan-1-one (400.0 mg, 1.63 mmol, 1.0 equiv.), NaI (292.4 mg, 1.95 mmol, 1.2 equiv.), $Et_3N$ (316 µL, 230.3 mg, 2.28 mmol, 1.4 equiv.) and *tert*-butylchlorodimethylsilane (294.1 mg, 1.95 mmol, 1.2 equiv.). The desired compound was purified by column chromatography on passivated silica gel eluting with hexane and was obtained as a colorless liquid (513.8 mg, 1.43 mmol, 88%).

**$^1$H NMR** (300 MHz, $CDCl_3$): δ = 7.68−7.62 (m, 2H), 7.36−7.31 (m, 2H), 4.88 (d, *J* = 1.9 Hz, 1H), 4.42 (d, *J* = 1.9 Hz, 1H), 0.99 (s, 9H), 0.20 (s, 6H) ppm.

**$^{13}$C{$^1$H} NMR** (101 MHz, $CDCl_3$) δ = 155.2, 137.5, 137.3, 127.2, 94.0, 91.6, 26.0, 18.5, − 4.5 ppm.

**HRMS-ESI (m/z)** calculated for $C_{14}H_{21}IOSi$ $[M+H]^+$: 361.0479; found: 361.0486.

**IR** (ATR, neat) $\tilde{\nu}$ = 2955, 2927, 2884, 2856, 1769, 1614, 1585, 1556, 1482, 1471, 1462, 1388, 1361, 1310, 1294, 1252, 1183, 1112, 1102, 1060, 1011, 1001, 938, 827, 779, 731, 717, 687, 660, 630, 615, 570, 543, 536, 521, 508, 503, 497, 487, 484, 477, 471, 459, 455, 435, 428, 421, 416, 412, 404 $cm^{-1}$.

Synthesis of **3m**:

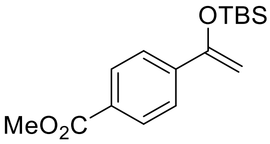


Prepared following general procedure A from methyl 4-acetylbenzoate (356.4 mg, 2.00 mmol, 1.0 equiv.), NaI (359.8 mg, 2.40 mmol, 1.2 equiv.), $Et_3N$ (390 µL, 283.3 mg, 2.80 mmol, 1.4 equiv.) and *tert*-butylchlorodimethylsilane (361.7 mg, 2.40 mmol, 1.2 equiv.). The desired compound was obtained as a colorless oil without the need for further purification (332.1 mg, 1.10 mmol, 55%).

**$^1$H NMR** (300 MHz, $CDCl_3$): δ = 7.99 (dt, *J* = 8.4, 1.9 Hz, 2H), 7.66 (dt, *J* = 8.4, 1.9 Hz, 2H), 4.99 (d, *J* = 1.9 Hz, 1H), 4.52 (d, *J* = 1.9 Hz, 1H), 3.91 (s, 3H), 1.00 (s, 9H), 0.21 (s, 6H) ppm.

**$^{13}$C{$^1$H} NMR** (101 MHz, $CDCl_3$) δ = 167.0, 155.2, 142.3, 129.8, 129.6, 125.3, 93.2, 52.2, 26.0, 18.5, − 4.5 ppm.

**HRMS-ESI (m/z)** calculated for $C_{16}H_{24}O_3Si$ $[M+Na]^+$: 315.1387; found: 315.1382.

**IR** (ATR, neat) $\tilde{\nu}$ = 2953, 2930, 2885, 2858, 2552, 1721, 1610, 1570, 1507, 1472, 1463, 1435, 1408, 1362, 1310, 1274, 1193, 1180, 1104, 1018, 1003, 980, 939, 876, 863, 835, 779, 729, 709, 695, 664, 552, 521, 509, 500, 485, 471, 447, 439, 430, 423 $cm^{-1}$.

Synthesis of **3n**:

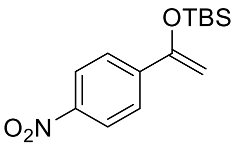


Prepared following general procedure A from 1-(4-nitrophenyl)ethan-1-one (267 µL, 270.2 mg, 1.63 mmol, 1.0 equiv.), NaI (294.1 mg, 1.96 mmol, 1.2 equiv.), $Et_3N$ (317 µL, 231.7 mg, 2.29 mmol, 1.4 equiv.) and *tert*-butylchlorodimethylsilane (295.7 mg, 1.96 mmol, 1.2 equiv.). The desired compound was purified by column chromatography on passivated silica gel eluting with hexane and was obtained as a yellow oil (401.9 mg, 1.44 mmol, 88%).

**$^1$H NMR** (300 MHz, $CDCl_3$): δ = 8.22−8.15 (m, 2H), 7.78−7.71 (m, 2H), 5.05 (d, *J* = 2.3 Hz, 1H), 4.61 (d, *J* = 2.3 Hz, 1H), 1.01 (s, 9H), 0.23 (s, 6H) ppm.

**$^{13}$C{$^1$H} NMR** (101 MHz, $CDCl_3$) δ = 154.2, 147.6, 144.1, 126.0, 123.6, 94.6, 25.9, 18.4, −4.5 ppm.

**HRMS-ESI (m/z)** calculated for $C_{14}H_{21}NO_3Si$ $[M+H]^+$: 280.1363; found: 280.1369.

**IR** (ATR, neat) $\tilde{\nu}$ = 3117, 2958, 2926, 2884, 2857, 2361, 1926, 1789, 1619, 1593, 1518, 1490, 1469, 1445, 1404, 1344, 1319, 1304, 1289, 1250, 1182, 1101, 1000, 937, 853, 833, 811, 781, 761, 745, 701, 660, 636, 625, 603, 570, 533, 457, 424, 404 $cm^{-1}$.

Synthesis of **3q**:

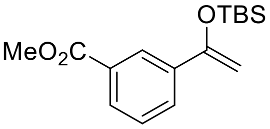


Prepared following general procedure A from methyl 3-acetylbenzoate (290.2 mg, 1.63 mmol, 1.0 equiv.), NaI (292.7 mg, 1.95 mmol, 1.2 equiv.), $Et_3N$ (316 µL, 230.6 mg, 2.28 mmol, 1.4 equiv.) and *tert*-butylchlorodimethylsilane (294.4 mg, 1.95 mmol, 1.2 equiv.). The desired compound was obtained as colorless oil without the need for further purification (352.1 mg, 1.20 mmol, 74%).

**$^1$H NMR** (400 MHz, $CDCl_3$): δ = 8.30 (t, *J* = 1.6 Hz, 1H), 7.96 (dt, *J* = 7.8, 1.4 Hz, 1H), 7.79 (ddd, *J* = 7.9, 1.9, 1.3 Hz, 1H), 7.40 (t, *J* = 7.7 Hz, 1H), 4.97 (d, *J* = 1.9 Hz, 1H), 4.49 (d, *J* = 1.9 Hz, 1H), 3.92 (s, 3H), 1.01 (s, 9H), 0.22 (s, 6H) ppm.

**$^{13}$C{$^1$H} NMR** (101 MHz, $CDCl_3$) δ = 167.2, 155.1, 138.3, 130.2, 129.7, 129.3, 128.3, 126.7, 91.9, 52.3, 26.0, 18.5, − 4.50 ppm.

**HRMS-ESI (m/z)** calculated for $C_{16}H_{24}O_3Si$ $[M+H]^+$: 293.1567; found: 293.1560.

**IR** (ATR, neat) $\tilde{\nu}$ = 2952, 2930, 2885, 2857, 1725, 1618, 1579, 1471, 1462, 1437, 1390, 1362, 1320, 1285, 1250, 1191, 1168, 1132, 1083, 1013, 938, 860, 827, 812, 780, 760, 701, 670, 587, 512, 464, 439, 409, 402 $cm^{-1}$.

Synthesis of **3u**:

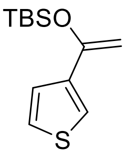


Prepared following general procedure A from 1-(thiophen-3-yl)ethan-1-one (230.0 mg, 1.82 mmol, 1.0 equiv.), NaI (327.9 mg, 2.19 mmol, 1.2 equiv.), $Et_3N$ (354 µL, 258.3 mg, 2.55 mmol, 1.4 equiv.) and *tert*-butylchlorodimethylsilane (329.7 mg, 2.19 mmol, 1.2 equiv.). The desired compound was purified by column chromatography on passivated silica gel eluting with hexane and was obtained as a colorless liquid (390.2 mg, 1.62 mmol, 89%).

**$^1$H NMR** (300 MHz, $CDCl_3$): δ = 7.39 (dd, *J* = 3.0, 1.4 Hz, 1H), 7.25−7.20 (m, 2H), 4.74 (d, *J* = 1.6 Hz, 1H), 4.36 (d, *J* = 1.7 Hz, 1H), 1.01 (s, 9H), 0.22 (s, 6H) ppm.

**$^{13}$C{$^1$H} NMR** (101 MHz, $CDCl_3$) δ = 152.6, 140.7, 125.7, 125.5, 121.9, 90.8, 26.0, 18.4, − 4.5 ppm.

**HRMS-ESI (m/z)** calculated for $C_{12}H_{20}OSSi$ $[M+H]^+$: 241.1077; found: 241.1086.

**IR** (ATR, neat) $\tilde{\nu}$ = 2955, 2929, 2885, 2857, 2360, 2342, 1615, 1520, 1471, 1462, 1393, 1361, 1287, 1253, 1197, 1101, 1078, 1012, 1002, 938, 917, 867, 834, 810, 789, 674, 583, 530, 437, 413 $cm^{-1}$.

Synthesis of **3v**:

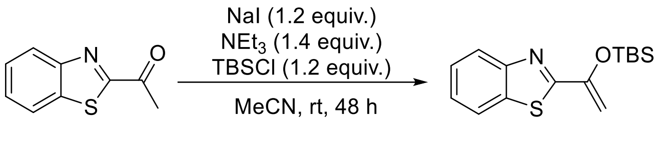


**S1** **3v**

Prepared following general procedure A from **S1** (250.2 mg, 1.41 mmol, 1.0 equiv.), NaI (253.7 mg, 1.69 mmol, 1.2 equiv.), $Et_3N$ (273.8 µL, 199.9 mg, 1.97 mmol, 1.4 equiv.) and *tert*-butylchlorodimethylsilane (255.2 mg, 1.69 mmol, 1.2 equiv.). The desired compound was obtained as a colorless liquid without the need for further purification (387.7 mg, 1.33 mmol, 94%).

**$^1$H NMR** (300 MHz, $CDCl_3$): δ = 8.01 (d, *J* = 7.9 Hz, 1H), 7.88 (d, *J* = 7.9 Hz, 1H), 7.47 (ddd, *J* = 8.3, 7.2, 1.3 Hz, 1H), 7.37 (ddd, *J* = 8.2, 7.2, 1.2 Hz, 1H), 5.69 (d, *J* = 1.9 Hz, 1H), 4.64 (d, *J* = 1.9 Hz, 1H), 1.06 (s, 9H), 0.30 (s, 6H) ppm.

**$^{13}$C{$^1$H} NMR** (101 MHz, $CDCl_3$) δ = 167.9, 154.0, 150.1, 135.6, 126.3, 125.1, 123.3, 121.8, 94.3, 25.8, 18.4, − 4.6 ppm.

**HRMS-ESI (m/z)** calculated for $C_{15}H_{21}NOSi$ $[M+H]^+$: 292.1186; found: 292.1189.

**IR** (ATR, neat) $\tilde{\nu}$ = 3063, 2954, 2928, 2884, 2857, 1611, 1557, 1498, 1471, 1461, 1434, 1409, 1390, 1363, 1314, 1300, 1268, 1254, 1092, 1046, 1000, 938, 825, 811, 781, 756, 727, 686, 657, 631, 575, 543, 479, 463, 426, 404 $cm^{-1}$.

Synthesis of **3w**:

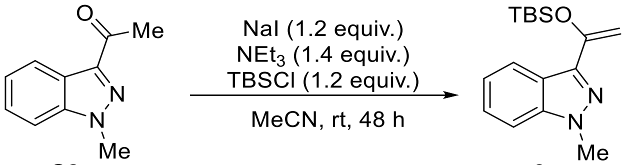


**S2** **3w**

Prepared following general procedure A from **S2** (300.1 mg, 1.72 mmol, 1.0 equiv.), NaI (309.8 mg, 2.07 mmol, 1.2 equiv.), $Et_3N$ (334 µL, 243.9 mg, 2.41 mmol, 1.4 equiv.) and *tert*-butylchlorodimethylsilane (311.5 mg, 2.07 mmol, 1.2 equiv.). The desired compound was purified by column chromatography on passivated silica gel eluting with pentane/EtOAc (100/0→50/1 (v/v)) and was obtained as a colorless oil (432.6 mg, 1.50 mmol, 87%).

**$^1$H NMR** (300 MHz, $CDCl_3$): δ = 8.00 (dt, *J* = 8.2, 1.0 Hz, 1H), 7.40−7.33 (m, 2H), 7.15 (ddd, *J* = 8.2, 6.0, 1.8 Hz, 1H), 5.15 (d, *J* = 1.3 Hz, 1H), 4.59 (d, *J* = 1.3 Hz, 1H), 4.06 (s, 3H), 1.01 (s, 9H), 0.25 (s, 6H) ppm.

**$^{13}$C{$^1$H} NMR** (101 MHz, $CDCl_3$) δ = 151.7, 142.0, 141.2, 126.2, 122.5, 121.7, 120.8, 109.1, 93.3, 35.7, 26.1, 18.7, − 4.3 ppm.

**HRMS-ESI (m/z)** calculated for $C_{16}H_{24}N_2OSi$ $[M+H]^+$: 289.1731; found: 289.1737.

**IR** (ATR, neat) $\tilde{\nu}$ = 2957, 2928, 2854, 1608, 1497, 1469, 1435, 1408, 1339, 1295, 1253, 1215, 1165, 1118, 1044, 995, 941, 833, 815, 781, 754, 733, 689, 657, 578, 507, 486, 446, 432, 420 cm$^{-1}$.

Synthesis of **3x**:

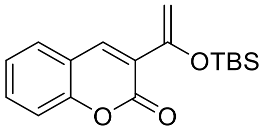


Prepared following general procedure A from 3-acetyl-2*H*-chromen-2-one (350.0 mg, 1.86 mmol, 1.0 equiv.), NaI (334.6 mg, 2.23 mmol, 1.2 equiv.), $Et_3N$ (360 µL, 263.5 mg, 2.60 mmol, 1.4 equiv.) and *tert*-butylchlorodimethylsilane (336.4 mg, 2.23 mmol, 1.2 equiv.). The desired compound was purified by column chromatography on passivated silica gel eluting with hexane and was obtained as a colorless liquid (449.2 mg, 1.49 mmol, 80%).

**$^1$H NMR** (300 MHz, $CDCl_3$): δ = 8.10 (s, 1H), 7.58−7.46 (m, 2H), 7.39−7.20 (m, 2H), 5.97 (d, J = 0.7 Hz, 1H), 4.80 (d, *J* = 0.7 Hz, 1H), 1.03 (s, 9H), 0.26 (s, 6H) ppm.

**$^{13}$C{$^1$H} NMR** (101 MHz, $CDCl_3$) δ = 159.1, 153.3, 149.1, 138.3, 131.8, 128.4, 124.5, 123.6, 119.3, 116.4, 99.5, 26.1, 18.5, − 4.4ppm.

**HRMS-ESI (m/z)** calculated for $C_{17}H_{22}O_3Si$ $[M+H]^+$: 303.1411; found: 303.1415.

**IR** (ATR, neat) $\tilde{\nu}$ = 3046, 2953, 2929, 2884, 2857, 1731, 1609, 1563, 1488, 1471, 1455, 1366, 1331, 1303, 1251, 1217, 1157, 1132, 1119, 1019, 1002, 986, 946, 923, 827, 812, 781, 753, 700, 670, 611, 576, 527, 456 cm$^{-1}$.

Synthesis of **3y**:

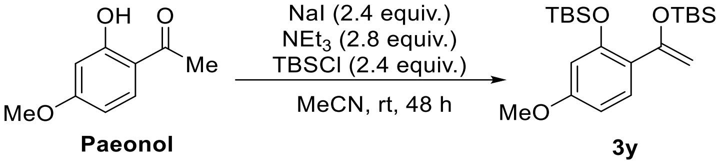


A Schlenk flask equipped with a magnetic stir bar was charged with 1-(2-hydroxy-4-methoxyphenyl)-ethanone (332.0 mg, 2.00 mmol, 1.0 equiv.), NaI (719.6 mg, 4.80 mmol, 2.4 equiv.) and *tert*-butylchlorodimethylsilane (723.4 mg, 4.80 mmol, 2.4 equiv.). MeCN (10 mL) and subsequently $NEt_3$ (780 µL, 566.6 mg, 5.60 mmol, 2.8 equiv.) were added and the reaction mixture was stirred at ambient temperature for 48 h. The solvent was removed under reduced pressure and the resulting residue was extracted with hexane (2×50 mL). The hexane extracts were combined and concentrated under reduced pressure yielding **3y** as a yellow oil without the need for further purification (726.0 mg, 1.84 mmol, 92%).

**$^1$H NMR** (300 MHz, $CDCl_3$): δ = 7.41 (d, *J* = 8.7 Hz, 1H), 6.49 (dd, J = 8.7, 2.5 Hz, 1H), 6.37 (d, *J* = 2.5 Hz, 1H), 4.89 (s, 1H), 4.57 (s, 1H), 3.77 (s, 3H), 1.00 (s, 9H), 0.95 (s, 9H), 0.23 (s, 6H), 0.12 (s, 6H) ppm.

**$^{13}$C{$^1$H} NMR** (101 MHz, $CDCl_3$) δ = 159.9, 154.0, 152.7, 129.8, 122.9, 106.4, 105.8, 95.8, 55.4, 26.0, 26.0, 18.5, 18.4, -3.8, − 4.4 ppm.

**HRMS-ESI (m/z)** calculated for $C_{21}H_{38}O_3Si_2$ [M+Na]$^+$: 417.2252; found: 417.2267 .

**IR** (ATR, neat) $\tilde{\nu}$ = 2955, 2929, 2885, 2858, 1604, 1569, 1501, 1472, 1464, 1442, 1424, 1390, 1362, 1324, 1295, 1251, 1199, 1165, 1148, 1136, 1090, 1038, 1016, 1004, 983, 939, 829, 778, 757, 697, 668, 574, 522, 506, 497, 489, 484, 469, 462, 454, 444, 433, 424, 416, 408, 402 cm$^{-1}$.

Synthesis of **3x**:

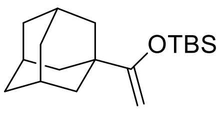


Prepared following general procedure A from 1-((3*r*,5*r*,7*r*)-adamantan-1-yl)ethan-1-one (350.0 mg, 1.96 mmol, 1.0 equiv.), NaI (353.1 mg, 2.36 mmol, 1.2 equiv.), $Et_3N$ (380 µL, 278.1 mg, 2.75 mmol, 1.4 equiv.) and *tert*-butylchlorodimethylsilane (355.1 mg, 2.36 mmol, 1.2 equiv.). The desired compound was purified by column chromatography on passivated silica gel eluting with hexane and was obtained as a colorless liquid (501.8 mg, 1.72 mmol, 87%).

**$^1$H NMR** (300 MHz, $CDCl_3$): δ = 3.96 (d, *J* = 1.3 Hz, 1H), 3.91 (d, *J* = 1.3 Hz, 1H), 2.02−1.95 (m, 3H), 1.75−1.61 (m, 12H), 0.95 (s, 9H), 0.17 (s, 6H) ppm.

**$^{13}$C{$^1$H} NMR** (101 MHz, $CDCl_3$) δ = 167.7, 85.1, 40.2, 38.6, 37.2, 28.6, 26.1, 18.5, −4.5 ppm.

**HRMS-ESI (m/z)** calculated for $C_{18}H_{32}OSi$ [M+H]$^+$: 293.2295; found: 293.2298.

**IR** (ATR, neat) $\tilde{\nu}$ = 2903, 2851, 1652, 1612, 1471, 1453, 1389, 1360, 1344, 1324, 1272, 1250, 1215, 1183, 1105, 1080, 1047, 1015, 1003, 976, 938, 894, 829, 810, 777, 758, 696, 659, 630, 593, 475, 458, 433, 414 cm$^{-1}$.

**Substrates used for catalyst-free transformations:**

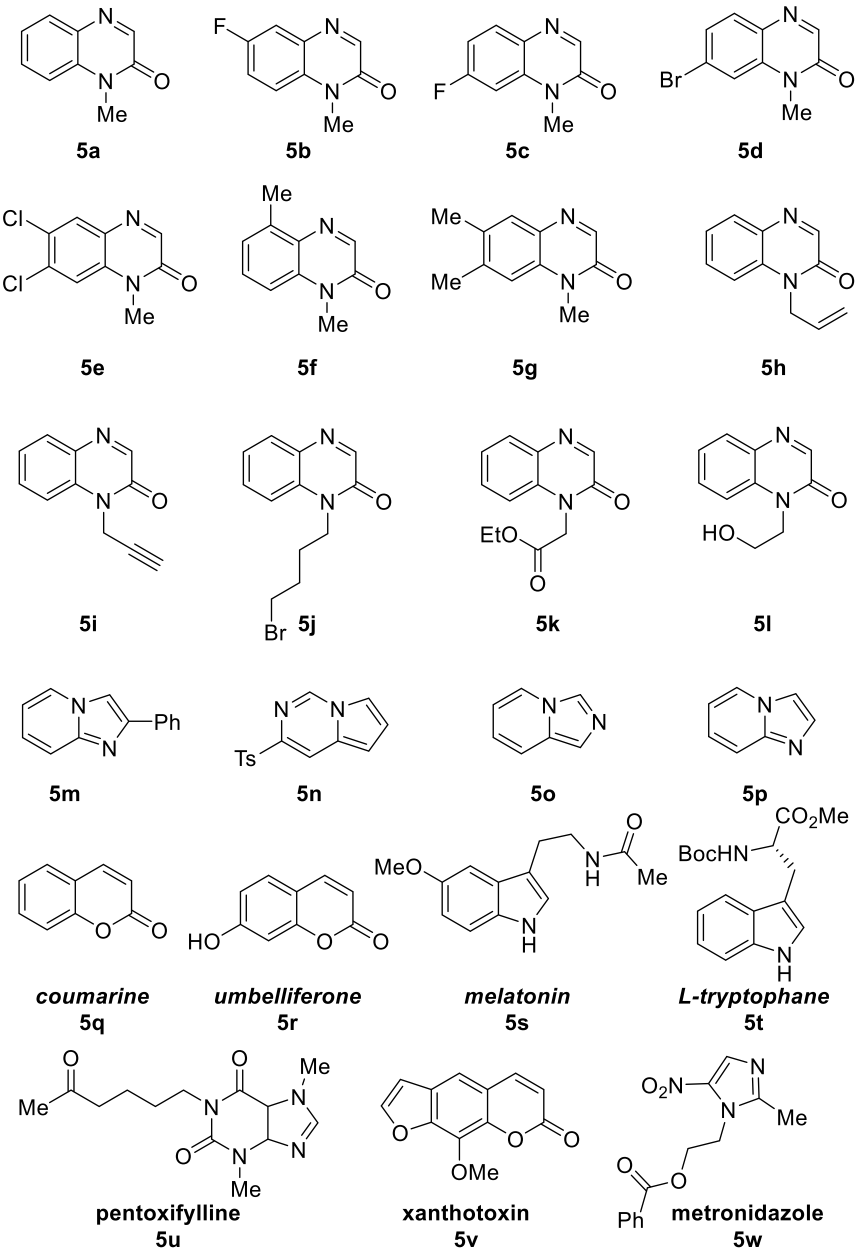


**Figure S2**: Scope of substrates used for the photochemical catalyst-free transfer of TFCp.

Substrates **5m**, **5o**, **5p**, **5q**, **5r**, **5s**, **5u**, **5v** are commercially available and were used as received from the corresponding supplier.

Substrates **5a**[14], **5b**[14], **5c**[14], **5d**[15], **5e**[14], **5f**[15], **5g**[15], **5h**[16], **5i**[16], **5j**[17], **5k**[14], **5l**[18], **5n**[19] **5t**[20] were prepared according to literature procedure.

Synthesis of **5w**:

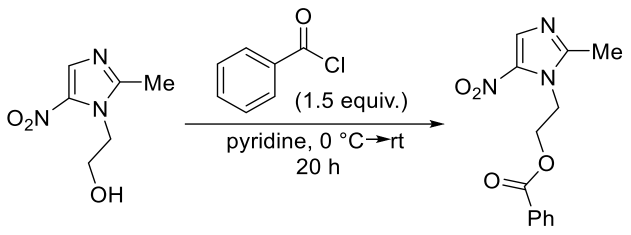

A Schlenk flask equipped with a magnetic stir bar was charged with metronidazole (600 mg, 3.51 mmol, 1.0 equiv.). The reaction flask was cooled to 0 °C and pyridine (5 mL) was added, followed by dropwise addition of benzoyl chloride (600 µL, 739 mg, 3.51 mmol, 1.5 equiv.). The reaction mixture was stirred at ambient temperature for 20 h. Finally, the reaction was quenched with HCl (2.0 mL, 1 M), and neutralized with sat. $NaHCO_3$ solution. The aqueous phase was extracted with DCM (3 × 40 mL), and the combined organic phases were dried over $Na_2SO_4$. The solvent was removed under reduced pressure, and the resulting residue purified by column chromatography on silica gel eluting with pentane/EtOAc (90/10→40/60 (v/v)). Compound **5w** was obtained as an off-white solid (882 mg, 3.21 mmol, 91%).

**$^1$H NMR** (400 MHz, $CDCl_3$): δ = 7.94 (s, 1H), 7.89 (dt, *J* = 8.5, 1.5 Hz, 3H), 7.60−7.51 (m, 1H), 7.46−7.37 (m, 2H), 4.75−4.61 (m, 7H), 2.46 (s, 3H) ppm.

**$^{13}$C{$^1$H} NMR** (101 MHz, $CDCl_3$) δ = 166.1, 150.9, 138.6, 133.6, 133.3, 129.6, 129.1, 128.7, 62.9, 45.3, 14.4 ppm.

**HRMS-ESI (m/z)** calculated for $C_{13}H_{13}N_3O_3$ $[M+H]^+$: 276.0979; found: 276.0982.

**IR** (ATR, neat) $\tilde{\nu}$ = 2360, 1698, 1602, 1527, 1469, 1451, 1427, 1379, 1361, 1313, 1272, 1257, 1186, 1144, 1114, 1071, 1040, 882, 822, 743, 709, 678, 645, 554, 520, 474, 451, 419, 409 cm$^{-1}$.

**Melting point:** 91-92 °C.

**(Hetero)aromatic substrates used for catalyst-free transformations:**

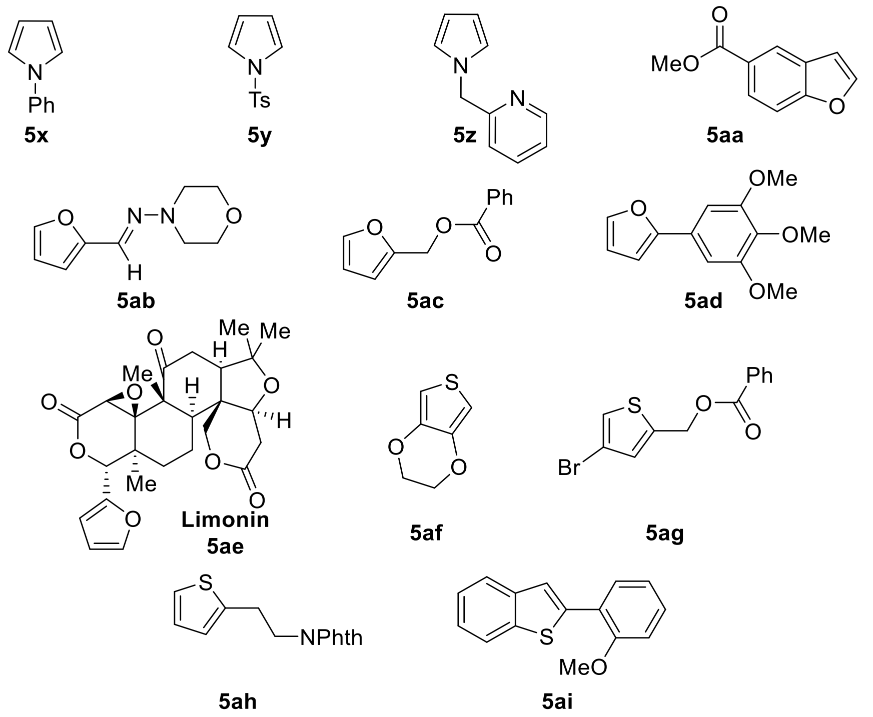


**Figure S3**: Scope of substrates used for the photoredox-catalyzed transfer of TFCp to heteroaromatic substrates.

Substrates **5x**, **5y**, **5z**, **5ae**, **5af** are commercially available and were used as received from the corresponding supplier.

Substrates **5aa**[21], **5ab**[22], **5ac**[23], **5ah**[24] were prepared according to literature procedure.

Synthesis of **5ad**:

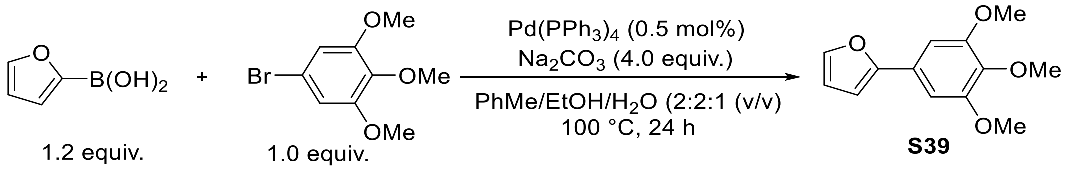


A Schlenk flask equipped with a magnetic stir bar was charged with furan-2-ylboronic acid (0.54 g, 4.86 mmol, 1.2 equiv.), 5-bromo-1,2,3-trimethoxybenzene (1.00 g, 4.05 mmol, 1.0 equiv.), $Pd(PPh_3)_4$ (23.4 mg, 20.0 µmol, 0.5 mol%) and $Na_2CO_3$ (1.72 g, 16.2 mmol, 4.0 equiv.). Subsequently, toluene (4 mL), EtOH (4 mL) and $H_2O$ (2 mL) were added to the flask, and the mixture was degassed by bubbling with nitrogen. The reaction mixture was heated to 100 °C for 24 h and after this diluted with aq. sat. $NH_4Cl$ solution, and extracted with EtOAc (3 × 50 mL). The combined organic phases were dried over $Na_2SO_4$, and the solvent was removed under reduced pressure. Finally, the resulting residue was purified by column chromatography on silica gel eluting with pentane/EtOAc (100/0→10/1 (v/v)) delivering **5ad** as an orange oil (923.3 mg, 3.94 mmol, 97%).

$^{1}$**H NMR** (400 MHz, $CDCl_3$): δ = 7.45 (dd, *J* = 1.8, 0.7 Hz, 1H), 6.90 (s, 2H), 6.58 (dd, *J* = 3.4, 0.8 Hz, 1H), 6.47 (dd, *J* = 3.4, 1.8 Hz, 1H), 3.92 (s, 6H), 3.87 (s, 3H) ppm.

$^{13}$**C{$^{1}$H} NMR** (101 MHz, $CDCl_3$) δ = 153.9, 153.7, 142.0, 137.8, 126.8, 111.9, 104.8, 101.3, 61.1, 56.3 ppm.

**HRMS-ESI (m/z)** calculated for $C_{13}H_{14}O_4$ $[M+H]^+$: 235.0965; found: 235.0971.

**IR** (ATR, neat) $\tilde{\nu}$ = 3114, 2937, 2836, 1589, 1571, 1509, 1487, 1455, 1417, 1375, 1335, 1239, 1173, 1124, 1084, 1005, 937, 884, 833, 802, 765, 732, 683, 593, 532, 464, 438, 422, 408 $cm^{-1}$.

Synthesis of **5ag**:

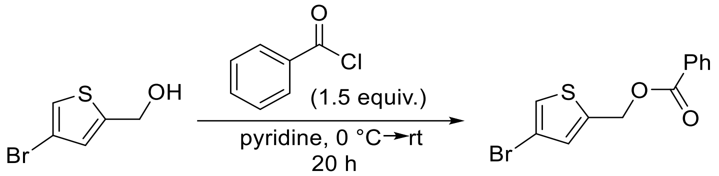

**S35**

A Schlenk flask equipped with a magnetic stir bar was charged with (4-bromothiophen-2-yl)methanol (1.00 g, 5.18 mmol, 1.0 equiv.). The reaction flask was cooled to 0 °C, and pyridine (5 mL) was added. To the cooled solution, benzoyl chloride (900 µL, 1.09 g, 7.77 mmol, 1.5 equiv.) was added dropwise and the reaction mixture was stirred at r.t. for 20 h. After this, the mixture was quenched with HCl (2.5 mL, 1 M), and neutralized with sat. $NaHCO_3$ solution. The aqueous phase was extracted with $Et_2O$ (3 × 40 mL), and the combined organic phases dried over $Na_2SO_4$. Finally, the solvent was removed under reduced pressure and the resulting residue purified by column chromatography on silica gel eluting with pentane/EtOAc (100/0→10/1 (v/v)). Compound **5ag** was delivered as a colorless oil (1.44 mg, 4.85 mmol, 94%).

$^{1}$**H NMR** (300 MHz, $CDCl_3$): δ = 8.09−8.01 (m, 2H), 7.62−7.53 (m, 1H), 7.50−7.40 (m, 2H), 7.23 (d, *J* = 1.5 Hz, 1H), 7.10 (dt, *J* = 1.5, 0.8 Hz, 1H), 5.45 (s, 2H) ppm.

$^{13}$**C{$^{1}$H} NMR** (101 MHz, $CDCl_3$) δ = 166.3, 139.6, 133.4, 130.7, 129.9, 129.7, 128.6, 124.1, 109.6, 60.6 ppm.

**HRMS-ESI (m/z)** calculated for $C_{12}H_9BrO_2S$ $[M+Na]^+$: 318.9399; found: 318.9400.

**IR** (ATR, neat) $\tilde{\nu}$ = 3109, 3086, 2956, 1973, 1706, 1597, 1580, 1521, 1489, 1444, 1421, 1378, 1349, 1308, 1266, 1189, 1171, 1153, 1092, 1068, 1023, 1000, 988, 937, 924, 871, 853, 825, 750, 716, 702, 687, 670, 634, 578, 518, 484, 439, 423, 405 $cm^{-1}$.

Synthesis of **5ai**:

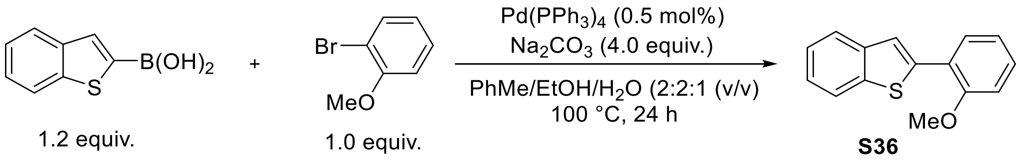


A Schlenk flask equipped with a magnetic stir bar was charged with benzo[*b*]thiophen-2-ylboronic acid (0.57 g, 3.21 mmol, 1.2 equiv.), $Pd(PPh_3)_4$ (15.5 mg, 13.4 µmol, 0.5 mol%) and $Na_2CO_3$ (1.13 g, 10.7 mmol, 4.0 equiv.). Subsequently toluene (4 mL), EtOH (4 mL), $H_2O$ (2 mL) and 1-bromo-2-methoxybenzene (0.50 g, 333 µL, 2.67 mmol, 1.0 equiv.) were added. The reaction mixture was then degassed and heated to 100 °C for 24 h. After this aq. sat. $NH_4Cl$ solution was added and the mixture extracted with EtOAc (3 × 50 mL). The combined organic phases were dried over $Na_2SO_4$, the solvent removed under reduced pressure and the resulting residue purified by column chromatography on silica gel eluting with pentane/EtOAc (100/0→100/1 (v/v)). Compound **5ai** was obtained as a colorless solid (621.8 mg, 2.59 mmol, 97%).

**$^1$H NMR** (300 MHz, $CDCl_3$): δ = 7.87−7.68 (m, 4H), 7.38−7.27 (m, 3H), 7.04 (t, J = 8.1 Hz, 2H), 3.97 (s, 3H) ppm.

**$^{13}$C{$^1$H} NMR** (101 MHz, $CDCl_3$) δ = 156.5, 140.3, 140.2, 139.9, 129.7, 129.4, 124.3, 124.2, 123.6, 123.4, 122.7, 121.9, 121.1, 111.9, 55.8 ppm.

**HRMS-ESI (m/z)** calculated for $C_{15}H_{12}OS$ $[M+H]^+$: 241.0682; found: 241.0682.

**IR** (ATR, neat) $\tilde{\nu}$ = 3051, 2996, 2939, 2839, 2504, 2323, 2277, 2043, 1934, 1903, 1872, 1823, 1785, 1690, 1590, 1576, 1518, 1482, 1446, 1434, 1336, 1313, 1297, 1251, 1185, 1163, 1128, 1114, 1074, 1051, 1023, 969, 935, 863, 828, 789, 751, 742, 732, 687, 646, 584, 549, 512, 493, 455, 427, 406 $cm^{-1}$.

**Melting point:** 60.5 °C.

## SYNTHESIS OF α-TFCp SUBSTITUTED KETONES

General procedure **B** for the synthesis of α-substituted ketones (**4a** – **4y**):

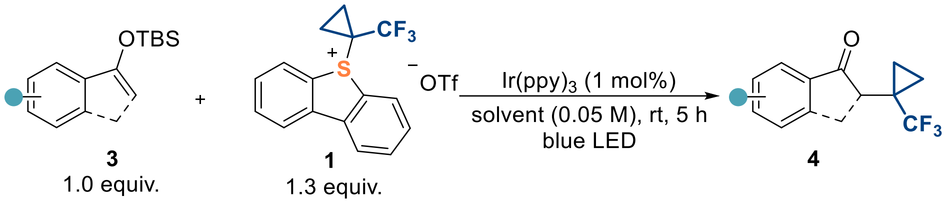

In a glove box, a Schlenk flask equipped with a magnetic stir bar was charged with Ir(ppy)$_3$ (1.3 mg, 0.2 µmol, 1 mol%), sulfonium salt **1** (0.26 mmol, 1.3 equiv.) and the desired silyl-enol ether (0.2 mmol, 1.0 equiv.). Subsequently, MeCN (4 mL) was added, the flask was sealed, transferred to a photoreactor equipped with blue LED strips (maximum wavelength: 462 nm), and irradiated at 50% intensity for 5 h. Finally, the reaction mixture was diluted with $CH_2Cl_2$ (3 mL), and purified by silica gel column chromatography.

Synthesis of **4a**:

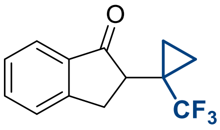

Prepared following general procedure B from ((1*H*-inden-3-yl)oxy)(*tert*-butyl)dimethylsilane **3a** ( 50.1 mg, 0.20 mmol, 1.0 equiv.) and sulfonium salt **1** (116.7 mg, 0.26 mmol, 1.3 equiv.). Compound **4a** was purified by chromatography on silica gel eluting with pentane/EtOAc (100/0→100/1 (v/v)) and obtained as a colorless oil (39.2 mg, 0.16 mmol, 80%).

**$^1$H NMR** (300 MHz, $CDCl_3$): δ = 7.75 (d, *J* = 7.7 Hz, 1H), 7.63 (td, *J* = 7.4, 1.2 Hz, 1H), 7.50 (d, *J* = 7.8 Hz, 1H), 7.39 (t, *J* = 7.5 Hz, 1H), 3.52 (dd, *J* = 17.6, 8.1 Hz, 1H), 3.32 (dd, *J* = 17.7, 4.5 Hz, 1H), 2.89 (dd, *J* = 8.1, 4.4 Hz, 1H), 1.53−1.40 (m, 1H), 1.16−1.01 (m, 2H), 0.65−0.58 (m, 1H) ppm.

**$^{13}$C{$^1$H} NMR** (101 MHz, $CDCl_3$) δ = 203.6, 153.4, 136.6, 135.4, 127.7,127.3 (q, *J* = 274.8 Hz), 126.5, 124.2, 46.1, 33.0 (d, *J* = 1.5 Hz), 24.4 (q, *J* = 32.0 Hz), 7.7 (dq, *J* = 16.5, 2.6 Hz) ppm.

**$^{19}$F NMR** (282 MHz, $CDCl_3$) δ = − 68.9 ppm.

**HRMS-ESI (m/z)** calculated for $C_{13}H_{11}OF_3$ $[M+H]^+$: 241.0835; found: 241.0836.

**IR** (ATR, neat) $\tilde{\nu}$ = 2902, 1704, 1610, 1586, 1465, 1437, 1397, 1357, 1323, 1297, 1274, 1257, 1216, 1208, 1133, 1115, 1072, 1059, 1037, 976, 963, 946, 926, 889, 833, 799, 747, 720, 686, 640, 614, 586, 560, 480, 456, 412 $cm^{-1}$.

**Melting point:** 52.0 °C

Synthesis of **4c**:

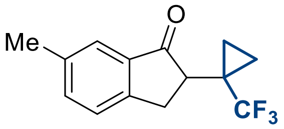

Prepared following general procedure B from *tert*-butyldimethyl((5-methyl-1*H*-inden-3-yl)oxy)silane **3c** ( 52.0 mg, 0.20 mmol, 1.0 equiv.) and sulfonium salt **1** (114.8 mg, 0.26 mmol, 1.3 equiv.). Compound **4c** was purified by chromatography on silica gel eluting with pentane/EtOAc (100/0→50/1 (v/v)) and obtained as a yellowish oil (31.1 mg, 0.12 mmol, 61%).

**$^1$H NMR** (400 MHz, $CDCl_3$): δ = 7.35 (d, *J* = 8.3 Hz, 1H), 7.20 (dd, *J* = 8.4, 2.6 Hz, 1H), 7.14 (d, *J* = 2.6 Hz, 1H), 3.82 (s, 3H), 3.41 (dd, *J* = 17.3, 7.9 Hz, 1H), 3.21 (dd, *J* = 17.3, 4.2 Hz, 1H), 2.88 (dd, *J* = 7.9, 4.1 Hz, 1H), 1.45−1.38 (m, 1H), 1.11−1.00 (m, 2H), 0.61−0.56 (m, 1H) ppm.

**$^{13}$C{$^1$H} NMR** (101 MHz, $CDCl_3$) δ = 203.9, 159.7, 146.3, 137.7, 127.3 (q, *J* = 274.5 Hz), 127.2, 124.9, 105.2, 55.7, 46.9, 32.4, 24.4 (q, *J* = 32.0 Hz), 7.7 (dq, *J* = 18.9, 2.7 Hz) ppm.

**$^{19}$F NMR** (377 MHz, $CDCl_3$) δ = − 68.9 ppm.

**HRMS-ESI (m/z)** calculated for $C_{14}H_{13}F_3O$ [M+H]$^+$: 255.0991; found: 255.0986.

**IR** (ATR, neat) $\tilde{\nu}$ = 3061, 2949, 2844, 1703, 1664, 1613, 1491, 1464, 1451, 1434, 1391, 1336, 1293, 1277, 1250, 1232, 1205, 1193, 1175, 1136, 1115, 1074, 1041, 1024, 979, 946, 929, 911, 896, 864, 827, 795, 775, 750, 706, 687, 667, 640, 620, 585, 557, 538, 521, 499, 486, 467, 447, 437, 427, 418, 411, 402 cm$^{-1}$.

Synthesis of **4d**:

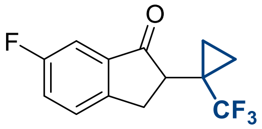

Prepared following general procedure B from *tert*-butyl((5-fluoro-1*H*-inden-3-yl)oxy)dimethylsilane **3d** ( 53.0 mg, 0.20 mmol, 1.0 equiv.) and sulfonium salt **1** (115.0 mg, 0.26 mmol, 1.3 equiv.). Compound **4d** was purified by chromatography on silica gel eluting with pentane/EtOAc (100/0→100/1 (v/v)) and obtained as a colorless oil (32.3 mg, 0.12 mmol, 58%).

**$^1$H NMR** (400 MHz, $CDCl_3$): δ 7.49−7.39 (m, 1H), 7.39−7.28 (m, 2H), 3.46 (dd, *J* = 17.4, 8.0 Hz, 1H), 3.27 (dd, *J* = 17.5, 4.4 Hz, 1H), 2.87 (dd, *J* = 8.0, 4.4 Hz, 1H), 1.39 (dddt, *J* = 10.9, 6.5, 3.1, 1.5 Hz, 1H), 1.15−1.00 (m, 2H), 0.63 (dddd, *J* = 11.5, 5.1, 3.5, 1.8 Hz, 1H) ppm.

**$^{13}$C{$^1$H} NMR** (101 MHz, $CDCl_3$) δ = = 203.0 (d, *J* = 3.0 Hz), 162.5 (d, *J* = 248.3 Hz), 148.7 (d, *J* = 2.1 Hz), 138.2 (d, *J* = 7.3 Hz), 127.9 (d, *J* = 7.9 Hz), 127.2 (q, *J* = 272.7 Hz) 123.1 (d, *J* = 23.8 Hz), 110.0 (d, *J* = 22.0 Hz), 47.3, 32.3 (q, *J* = 1.6 Hz), 24.3 (q, *J* = 32.1 Hz), 7.8 (dq, *J* = 5.3, 2.6 Hz) ppm.

**$^{19}$F NMR** (377 MHz, $CDCl_3$) δ = −68.64, −114.06 (dddd, *J* = 10.6, 6.4, 4.0, 1.7 Hz) ppm.

**HRMS-ESI (m/z)** calculated for $C_{13}H_{10}F_4O$ [M]$^{\cdot+}$: 258.0662; found: 258.0659.

**IR** (ATR, neat) $\tilde{\nu}$ = 3404, 3069, 2896, 1706, 1671, 1615, 1488, 1446, 1432, 1399, 1360, 1325, 1286, 1267, 1226, 1215, 1178, 1162, 1141, 1117, 1074, 1062, 1038, 980, 954, 948, 928, 886, 878, 864, 822, 787, 772, 756, 707, 682, 669, 637, 617, 587, 576, 558, 527, 513, 505, 499, 490, 477, 463, 446, 440, 435, 411, 402 cm$^{-1}$.

Synthesis of **4e**:

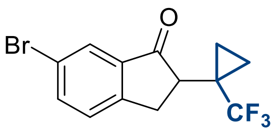

Prepared following general procedure B from ((5-bromo-1*H*-inden-3-yl)oxy)(*tert*-butyl)dimethylsilane **3e** ( 66.1 mg, 0.20 mmol, 1.0 equiv.) and sulfonium salt **1** (116.7 mg, 0.26 mmol, 1.3 equiv.). Compound **4e** was purified by chromatography on silica gel eluting with pentane/EtOAc (100/0→100/1 (v/v)) and obtained as a yellowish oil (38.8 mg, 0.12 mmol, 61%).

**$^{1}$H NMR** (300 MHz, $CDCl_3$): δ = 7.85 (d, J = 1.9 Hz, 1H), 7.71 (dd, *J* = 8.1, 2.0 Hz, 1H), 7.38−7.33 (m, 1H), 3.44 (dd, *J* = 17.8, 8.1 Hz, 1H), 3.24 (dd, *J* = 17.8, 4.5 Hz, 1H), 2.85 (dd, *J* = 8.1, 4.5 Hz, 1H), 1.42−1.32 (m, 1H), 1.16−1.00 (m, 2H), 0.68−0.57 (m, 1H) ppm.

**$^{13}$C{$^{1}$H} NMR** (101 MHz, $CDCl_3$) δ = 202.5, 151.8, 138.3, 138.1, 128.1, 127.2, 127.1 (q, *J* = 274.5 Hz), 120.0, 46.8, 32.6, 24.3 (q, *J* = 32.0 Hz), 7.9 (dq, *J* = 7.6, 2.6 Hz) ppm.

**$^{19}$F NMR** (377 MHz, $CDCl_3$) δ = − 68.6 ppm.

**HRMS-ESI (m/z)** calculated for $C_{13}H_{10}BrF_3O$ $[M+Na]^+$: 340.9759; found: 340.9773.

**IR** (ATR, neat) $\tilde{\nu}$ = 3057, 2360, 2341, 1713, 1596, 1469, 1439, 1393, 1347, 1316, 1261, 1211, 1195, 1163, 1145, 1133, 1116, 1072, 1057, 1038, 975, 943, 924, 891, 842, 818, 793, 781, 760, 727, 682, 647, 621, 609, 585, 502, 458, 418 $cm^{-1}$.

Synthesis of **4f**:

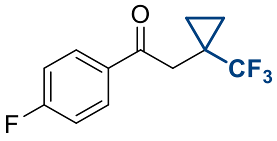

Prepared following general procedure B from *tert*-butyl((1-(4-fluorophenyl)vinyl)oxy)dimethylsilane **3f** (51.2 mg, 0.20 mmol, 1.0 equiv.) and sulfonium salt **1** (116.2 mg, 0.26 mmol, 1.3 equiv.). Compound **4f** was purified by chromatography on silica gel eluting with pentane/EtOAc (100/0→200/1 (v/v)) and obtained as a colorless oil (32.6 mg, 0.13 mmol, 66%).

**$^{1}$H NMR** (400 MHz, $CDCl_3$): δ = 8.00−7.94 (m, 2H), 7.18−7.10 (m, 2H), 3.21 (s, 2H), 1.15−1.09 (m, 2H), 0.86−0.82 (m, 2H) ppm.

**$^{13}$C{$^{1}$H} NMR** (101 MHz, $CDCl_3$) δ = 195.0, 166.1 (d, *J* = 255.4 Hz), 133.4 (d, *J* = 3.0 Hz), 131.0 (d, *J* = 9.4 Hz), 127.1 (q, *J* = 273.6 Hz), 116.0 (d, *J* = 22.0 Hz), 38.3, 20.1 (q, *J* = 33.7 Hz), 8.8 (q, *J* = 2.6 Hz) ppm.

**$^{19}$F NMR** (377 MHz, $CDCl_3$) δ = − 70.9, − 104.6 ppm.

**HRMS-EI (m/z)** calculated for $C_{12}H_{10}F_3IO$ $[M]^{\cdot}$: 353.9723; found: 353.9724.

**IR** (ATR, neat) $\tilde{\nu}$ = 3025, 2168, 2013, 2007, 1695, 1597, 1507, 1402, 1354, 1308, 1214, 1155, 1122, 1069, 1038, 1012, 978, 937, 924, 832, 788, 623, 580, 533, 519, 502, 495, 473, 465, 457, 449, 431, 426, 417, 407, 403 $cm^{-1}$.

Synthesis of **4g**:

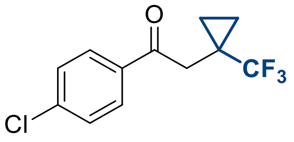


Prepared following general procedure B from *tert*-butyl((1-(4-chlorophenyl)vinyl)oxy)dimethylsilane **3g** ( 45.5 mg, 0.20 mmol, 1.0 equiv.) and sulfonium salt **1** (115.2 mg, 0.26 mmol, 1.3 equiv.). Compound **4g** was purified by chromatography on silica gel eluting with pentane/EtOAc (100/0→100/1 (v/v)) and obtained as a colorless oil (42.0 mg, 0.16 mmol, 79%).

**$^{1}$H NMR** (400 MHz, $CDCl_3$): δ = 7.91−7.85 (m, 2H), 7.48−7.42 (m, 2H), 3.20 (s, 2H), 1.16−1.09 (m, 2H), 0.86−0.81 (m, 2H) ppm.

**$^{13}$C{$^{1}$H} NMR** (101 MHz, $CDCl_3$) δ = 195.4, 140.1, 135.3, 129.7, 129.2, 127.1 (q, *J* = 273.9 Hz)., 38.3, 20.1 (q, *J* = 33.7 Hz), 8.9 (q, *J* = 2.5 Hz) ppm.

**$^{19}$F NMR** (377 MHz, $CDCl_3$) δ = − 70.9 ppm.

**HRMS-ESI (m/z)** calculated for $C_{12}H_{10}ClF_3O$ $[M+Na]^+$: 285.0264; found: 285.0268.

**IR** (ATR, neat) $\tilde{\nu}$ = 2924, 1691, 1604, 1586, 1399, 1352, 1304, 1248, 1207, 1153, 1121, 1069, 1037, 977, 938, 865, 777, 736, 690, 608, 590, 539, 520, 459, 421 $cm^{-1}$.

Synthesis of **4h**:

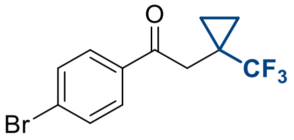


Prepared following general procedure B from ((1-(4-bromophenyl)vinyl)oxy)(*tert*-butyl)dimethylsilane **3h** (63.0 mg, 0.20 mmol, 1.0 equiv.) and sulfonium salt **1** (115.7 mg, 0.26 mmol, 1.3 equiv.). Compound **4h** was purified by chromatography on silica gel eluting with pentane/EtOAc (100/0→200/1 (v/v)) and obtained as a yellowish oil (43.3 mg, 0.14 mmol, 70%).

**$^{1}$H NMR** (400 MHz, $CDCl_3$): δ = 7.82−7.77 (m, 2H), 7.64−7.59 (m, 2H), 3.19 (s, 2H), 1.15−1.10 (m, 2H), 0.87−0.82 (m, 2H) ppm.

**$^{13}$C{$^{1}$H} NMR** (101 MHz, $CDCl_3$) δ = 195.6, 135.7, 132.2, 129.8, 128.8, 127.0 (q, *J* = 273.6 Hz)., 38.3, 20.1 (q, *J* = 33.7 Hz), 8.9 (q, *J* = 2.5 Hz) ppm.

**$^{19}$F NMR** (377 MHz, $CDCl_3$) δ = − 70.9 ppm.

**HRMS-ESI (m/z)** calculated for $C_{12}H_{10}BrF_3O$ $[M+Na]^+$: 328.9759; found: 328.9767.

**IR** (ATR, neat) $\tilde{\nu}$ = 2922, 1695, 1584, 1568, 1485, 1431, 1396, 1355, 1309, 1296, 1206, 1153, 1122, 1071, 1037, 1008, 976, 953, 937, 923, 812, 796, 749, 734, 696, 618, 608, 593, 574, 529, 502, 481, 463, 458, 453, 441, 435, 431, 425, 410, 404 $cm^{-1}$.

Synthesis of **4i**:

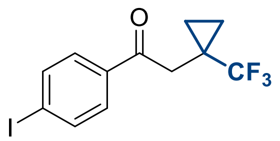

Prepared following general procedure B from *tert*-butyl((1-(4-iodophenyl)vinyl)oxy)dimethylsilane **3i** (73.0 mg, 0.20 mmol, 1.0 equiv.) and sulfonium salt **1** (116.5 mg, 0.26 mmol, 1.3 equiv.). Compound **4i** was purified by chromatography on silica gel eluting with pentane/EtOAc (100/0→100/1 (v/v)) and obtained as a colorless oil (54.8 mg, 0.16 mmol, 76%).

**$^1$H NMR** (400 MHz, $CDCl_3$): δ = 7.86−7.82 (m, 2H), 7.66−7.62 (m, 2H), 3.18 (s, 2H), 1.15−1.09 (m, 2H), 0.86−0.80 (m, 2H) ppm.

**$^{13}$C{$^1$H} NMR** (101 MHz, $CDCl_3$) δ = 195.9, 138.2, 136.2, 129.7, 127.0 (d, *J* = 273.7 Hz), 101.6, 38.2, 20.0 (q, *J* = 33.8 Hz), 8.9 (q, *J* = 2.6 Hz) ppm.

**$^{19}$F NMR** (377 MHz, $CDCl_3$) δ = − 70.9 ppm.

**HRMS-ESI (m/z)** calculated for $C_{12}H_{10}F_3IO$ $[M+Na]^+$: 376.9621; found: 376.9636.

**IR** (ATR, neat) $\tilde{\nu}$ = 3025, 2912, 1685, 1579, 1562, 1481, 1437, 1407, 1391, 1361, 1312, 1298, 1272, 1219, 1209, 1178, 1159, 1120, 1068, 1060, 1038, 1004, 972, 954, 937, 927, 870, 851, 811, 797, 748, 732, 704, 689, 625, 593, 575, 525, 506, 501, 483, 471, 460, 443, 424, 418, 411, 406 $cm^{-1}$.

Synthesis of **4j**:

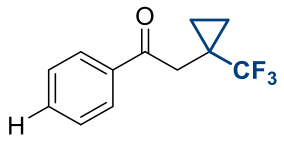

Prepared following general procedure B from *tert*-butyldimethyl((1-phenylvinyl)oxy)silane **3j** ( 55.3 mg, 0.20 mmol, 1.0 equiv.) and sulfonium salt **1** (115.1 mg, 0.26 mmol, 1.3 equiv.). Compound **4j** was purified by chromatography on silica gel eluting with pentane/EtOAc (100/0→150/1 (v/v)) and obtained as a yellow oil (26.9 mg, 0.12 mmol, 59%).

**$^1$H NMR** (400 MHz, $CDCl_3$): δ = 8.00−7.91 (m, 2H), 7.61−7.56 (m, 1H), 7.51−7.44 (m, 2H), 3.25 (s, 2H), 1.16−1.09 (m, 2H), 0.87−0.82 (m, 2H) ppm.

**$^{13}$C{$^1$H} NMR** (101 MHz, $CDCl_3$) δ = 196.5, 137.0, 133.5, 128.9, 128.3, 127.2 (q, *J* = 274.7 Hz), 38.3, 20.0 (q, *J* = 34.3 Hz), 8.8 (q, *J* = 2.5 Hz) ppm.

**$^{19}$F NMR** (377 MHz, $CDCl_3$) δ = − 71.0 ppm.

**HRMS-ESI (m/z)** calculated for $C_{12}H_{11}F_3O$ $[M+Na]^+$: 251.0654; found: 251.0655.

**IR** (ATR, neat) $\tilde{\nu}$ = 1770, 1759, 1716, 1687, 1653, 1596, 1578, 1558, 1541, 1522, 1507, 1489, 1471, 1449, 1435, 1402, 1386, 1360, 1321, 1304, 1246, 1222, 1202, 1175, 1151, 1131, 1117, 1067, 1038, 1016, 1000, 972, 963, 936, 919, 864, 799, 753, 689, 670, 616, 587, 579, 524, 456, 418, 402 $cm^{-1}$.

Synthesis of **4k**:

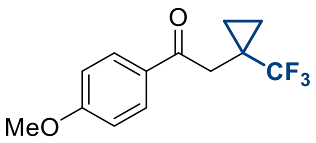

Prepared following general procedure B from *tert*-butyl((1-(4-methoxyphenyl)vinyl)oxy)dimethylsilane **3k** (53.1 mg, 0.20 mmol, 1.0 equiv.) and sulfonium salt **1** (115.3 mg, 0.26 mmol, 1.3 equiv.). Compound **4k** was purified by chromatography on silica gel eluting with pentane/EtOAc (100/0→100/1 (v/v)) and obtained as a colorless oil (37.4 mg, 0.14 mmol, 72%).

**$^1$H NMR** (400 MHz, $CDCl_3$): δ = 7.95−7.89 (m, 2H), 6.98−6.91 (m, 2H), 3.87 (s, 3H), 3.19 (s, 2H), 1.13−1.06 (m, 2H), 0.88−0.80 (m, 2H) ppm.

**$^{13}$C{$^1$H} NMR** (101 MHz, $CDCl_3$) δ = 195.07, 163.88, 130.69, 130.10, 127.2 (q, *J* = 273.6 Hz), 114.00, 55.62, 37.64, 20.1 (q, *J* = 33.6 Hz), 8.67 (q, *J* = 2.7 Hz) ppm.

**$^{19}$F NMR** (377 MHz, $CDCl_3$) δ = − 71.1 ppm.

**HRMS-ESI (m/z)** calculated for $C_{13}H_{13}F_3O_2$ $[M+H]^+$: 259.0940; found: 259.0953.

**IR** (ATR, neat) $\tilde{\nu}$ = 2974, 2938, 2906, 2844, 1680, 1602, 1575, 1508, 1460, 1438, 1410, 1395, 1356, 1308, 1254, 1228, 1206, 1183, 1172, 1149, 1130, 1114, 1076, 1045, 1027, 1016, 978, 969, 939, 924, 870, 849, 814, 784, 637, 623, 584, 576, 533, 503, 498, 492, 481, 473, 462, 446, 441, 431, 426, 417, 410, 404 $cm^{-1}$.

Synthesis of **4l**:

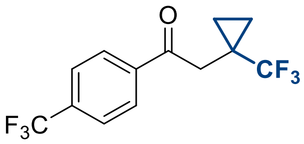

Prepared following general procedure B from *tert*-butyldimethyl((1-(4-(trifluoromethyl)phenyl)vinyl)oxy)silane **3l** (61.0 mg, 0.20 mmol, 1.0 equiv.) and sulfonium salt **1** (116.0 mg, 0.26 mmol, 1.3 equiv.). Compound **4l** was purified by chromatography on silica gel eluting with pentane/EtOAc (100/0→100/1 (v/v)) and obtained as a yellow liquid (33.7 mg, 0.11 mmol, 56%).

**$^1$H NMR** (400 MHz, $CDCl_3$): δ = 8.04 (d, *J* = 7.9 Hz, 1H), 7.75 (d, *J* = 8.4 Hz, 2H), 3.25 (s, 2H), 1.18−1.12 (m, 2H), 0.90−0.84 (m, 2H) ppm.

**$^{13}$C{$^1$H} NMR** (101 MHz, $CDCl_3$) δ = 195.69, 139.61, 134.8 (q, *J* = 32.8 Hz), 128.6, 127.0 (q, *J* = 273.8 Hz), 126.0 (q, *J* = 3.7 Hz), 123.7 (q, *J* = 272.6 Hz), 38.84, 20.1 (q, *J* = 33.9 Hz)., 8.9 (q, *J* = 2.5 Hz) ppm.

**$^{19}$F NMR** (377 MHz, $CDCl_3$) δ = − 63.2, − 70.8 ppm.

**HRMS-EI (m/z)** calculated for $C_{13}H_{10}F_6O$ $[M]^{\cdot}$: 296.0630; found: 296.0626.

**IR** (ATR, neat) $\tilde{\nu}$ = 2915, 1769, 1696, 1582, 1512, 1461, 1408, 1330, 1313, 1246, 1208, 1107, 1067, 1040, 1014, 976, 962, 939, 930, 871, 855, 831, 807, 769, 743, 734, 685, 633, 605, 594, 566, 523, 509, 499, 476, 467, 458, 451, 436, 429, 423, 418, 409, 402 $cm^{-1}$.

Synthesis of **4m**:

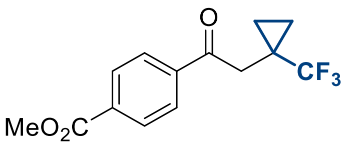


Prepared following general procedure B from methyl 4-(1-((*tert*-butyldimethylsilyl)oxy)vinyl)benzoate **3m** (58.5 mg, 0.20 mmol, 1.0 equiv.) and sulfonium salt **1** (115.7 mg, 0.26 mmol, 1.3 equiv.). Compound **4m** was purified by chromatography on silica gel eluting with pentane/EtOAc (100/0→50/1 (v/v)) and obtained as a colorless oil (34.3 mg, 0.12 mmol, 60%).

**$^{1}$H NMR** (400 MHz, $CDCl_3$): δ = 8.13 (td, *J* = 8.2, 1.6 Hz, 2H), 7.98 (td, *J* = 8.2, 1.6 Hz, 2H), 3.95 (s, 3H), 3.26 (s, 2H), 1.18−1.06 (m, 2H), 0.90−0.81 (m, 2H) ppm ppm.

**$^{13}$C{$^{1}$H} NMR** (101 MHz, $CDCl_3$) δ = 196.1, 166.3, 140.1, 134.3, 130.1, 128.2, 127.0 (q, *J* = 274.72 Hz), 52.7, 38.9, 20.0 (q, *J* = 33.8 Hz), 8.9 (q, *J* = 2.6 Hz) ppm.

**$^{19}$F NMR** (377 MHz, $CDCl_3$) δ = − 70.9 ppm.

**HRMS-ESI (m/z)** calculated for $C_{14}H_{13}O_3F_3$ $[M+Na]^+$: 309.0709; found: 309.0708.

**IR** (ATR, neat) $\tilde{\nu}$ = 2958, 2922, 2361, 2100, 1717, 1691, 1574, 1505, 1432, 1408, 1360, 1311, 1276, 1207, 1173, 1112, 1067, 1038, 1013, 978, 962, 937, 925, 876, 861, 841, 796, 782, 760, 692, 594, 527, 460, 410 $cm^{-1}$.

Synthesis of **4n**:

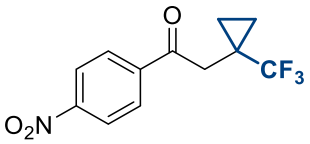


Prepared following general procedure B from *tert*-butyldimethyl((1-(4-nitrophenyl)vinyl)oxy)silane **3n** (56.1 mg, 0.20 mmol, 1.0 equiv.) and sulfonium salt **1** (116.3 mg, 0.26 mmol, 1.3 equiv.). Compound **4n** was purified by chromatography on silica gel eluting with pentane/EtOAc (100/0→50/1 (v/v)) and obtained as a yellow oil (40.6 mg, 0.15 mmol, 74%).

**$^{1}$H NMR** (300 MHz, $CDCl_3$): δ = 8.35−8.29 (m, 2H), 8.12−8.06 (m, 2H), 3.26 (s, 2H), 1.20−1.13 (m, 2H), 0.91−0.84 (m, 2H) ppm.

**$^{13}$C{$^{1}$H} NMR** (101 MHz, $CDCl_3$) δ = 195.19, 150.59, 141.32, 129.30, 126.9 (q, *J* = 273.6 Hz), 124.10, 39.25, 20.10 (q, *J* = 33.9 Hz), 9.0 (q, *J* = 2.5 Hz) ppm.

**$^{19}$F NMR** (377 MHz, $CDCl_3$) δ = − 70.7 ppm.

**HRMS-ESI (m/z)** calculated for $C_{12}H_{10}F_3NO_3$ $[M+Na]^+$: 296.0505; found: 296.0504.

**IR** (ATR, neat) $\tilde{\nu}$ = 3115, 2909, 1699, 1653, 1604, 1558, 1519, 1438, 1410, 1393, 1361, 1343, 1321, 1303, 1293, 1207, 1174, 1149, 1122, 1076, 1048, 1039, 1023, 1011, 986, 966, 928, 876, 856, 843, 798, 744, 700, 686, 629, 591, 566, 532, 511, 503, 492, 486, 481, 457, 442, 431, 427, 421, 415, 410 $cm^{-1}$.

Synthesis of **4o**:

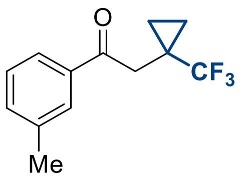


Prepared following general procedure B from *tert*-butyldimethyl((1-(m-tolyl)vinyl)oxy)silane **3o** (50.0 mg, 0.20 mmol, 1.0 equiv.) and sulfonium salt **1** (115.2 mg, 0.26 mmol, 1.3 equiv.). Compound **4o** was purified by chromatography on silica gel eluting with pentane/EtOAc (100/0→100/1 (v/v)) and obtained as a colorless oil (34.2 mg, 0.14 mmol, 71%).

**$^{1}$H NMR** (400 MHz, $CDCl_3$): δ = 7.80−7.70 (m, 2H), 7.45−7.31 (m, 2H), 3.24 (s, 2H), 2.42 (s, 3H), 1.15−1.07 (m, 2H), 0.88−0.79 (m, 2H) ppm.

**$^{13}$C{$^{1}$H} NMR** (101 MHz, $CDCl_3$) δ = 196.7, 138.7, 137.1, 134.3, 128.8, 128.7, 127.2 (q, *J* = 274.7Hz) 125.6, 38.3, 21.5, 20.0 (q, *J* = 33.6 Hz), 8.8 (q, *J* = 2.6 Hz) ppm.

**$^{19}$F NMR** (377 MHz, $CDCl_3$) δ = − 71.0 ppm.

**HRMS-ESI (m/z)** calculated for $C_{13}H_{13}F_3O$ $[M+H]^+$: 243.0991; found: 243.0991.

**IR** (ATR, neat) $\tilde{\nu}$ = 2925, 1691, 1604, 1586, 1399, 1353, 1305, 1249, 1207, 1153, 1121, 1069, 1038, 978, 938, 866, 777, 737, 690, 608, 590, 540, 521, 460, 421 $cm^{-1}$.

Synthesis of **4p**:

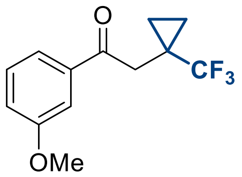


Prepared following general procedure B from *tert*-butyl((1-(3-methoxyphenyl)vinyl)oxy)dimethylsilane **3p** (53.0 mg, 0.20 mmol, 1.0 equiv.) and sulfonium salt **1** (115.9 mg, 0.26 mmol, 1.3 equiv.). Compound **4p** was purified by chromatography on silica gel eluting with pentane/EtOAc (100/0→50/1 (v/v)) and obtained as a colorless oil (34.5 mg, 0.13 mmol, 67%).

**$^{1}$H NMR** (400 MHz, $CDCl_3$): δ = 7.51 (dt, *J* = 7.6, 1.1 Hz, 1H), 7.47 (dd, *J* = 2.7, 1.6 Hz, 1H), 7.38 (t, *J* = 7.9 Hz, 1H), 7.12 (ddd, *J* = 8.2, 2.7, 1.0 Hz, 1H), 3.85 (s, 3H), 3.23 (s, 2H), 1.14−1.09 (m, 2H), 0.87−0.81 (m, 2H) ppm.

**$^{13}$C{$^{1}$H} NMR** (101 MHz, $CDCl_3$) δ = 196.3, 160.1, 138.4, 129.8, 127.1 (q, *J* = 273.8 Hz), 120.9, 120.1, 112.5, 55.6, 38.4, 20.0 (q, *J* = 33.6 Hz), 8.8 (q, *J* = 2.6 Hz) ppm.

**$^{19}$F NMR** (377 MHz, $CDCl_3$) δ = − 71.0 ppm.

**HRMS-ESI (m/z)** calculated for $C_{13}H_{13}O_2F_3$ $[M+H]^+$: 259.0940 ; found: 259.0951.

**IR** (ATR, neat) $\tilde{\nu}$ = 2357, 1692, 1597, 1582, 1486, 1465, 1451, 1428, 1396, 1304, 1290, 1256, 1195, 1151, 1118, 1036, 994, 981, 939, 879, 862, 770, 737, 685, 589, 561, 540, 524, 512, 504, 496, 479, 471, 454, 447, 432, 428, 422, 415, 404 $cm^{-1}$.

Synthesis of **4q**:

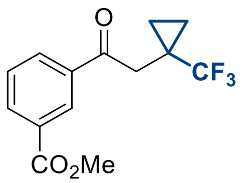


Prepared following general procedure B from methyl 3-(1-((*tert*-butyldimethylsilyl)oxy)vinyl)benzoate **3q** (58.1 mg, 0.20 mmol, 1.0 equiv.) and sulfonium salt **1** (116.0 mg, 0.26 mmol, 1.3 equiv.). Compound **4q** was purified by chromatography on silica gel eluting with pentane/EtOAc (100/0→100/1 (v/v)) and obtained as a yellow oil (41.1 mg, 0.14 mmol, 71%).

**$^1$H NMR** (400 MHz, $CDCl_3$): δ = 8.57 (t, *J* = 1.6 Hz, 1H), 8.25 (dt, *J* = 7.7, 1.4 Hz, 1H), 8.14 (ddd, *J* = 7.8, 1.9, 1.2 Hz, 1H), 7.57 (td, *J* = 7.8, 0.6 Hz, 1H), 3.96 (s, 3H), 3.28 (s, 2H), 1.19−1.11 (m, 2H), 0.89−0.83 (m, 2H) ppm.

**$^{13}$C{$^1$H} NMR** (101 MHz, $CDCl_3$) δ = 195.7, 166.3, 137.20, 134.27, 132.37, 130.97, 129.36, 129.18, 127.0 (q, *J* = 273.7 Hz), 52.60, 38.79, 20.0 (q, *J* = 33.7 Hz), 9.0 (q, *J* = 2.5 Hz).  ppm.

**$^{19}$F NMR** (377 MHz, $CDCl_3$) δ = − 70.8 ppm.

**HRMS-ESI (m/z)** calculated for $C_{14}H_{13}O_3F_3$ $[M+H]^+$: 287.0890; found: 287.0899.

**IR** (ATR, neat) $\tilde{\nu}$ = 1724, 1701, 1604, 1511, 1431, 1402, 1357, 1280, 1196, 1154, 1122, 1067, 989, 967, 936, 816, 752, 704, 682, 655, 616, 582, 530, 511, 503, 491, 479, 474, 465, 460, 453, 447, 440, 431, 423, 415, 405 $cm^{-1}$.

Synthesis of **4r**:

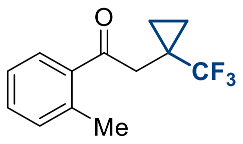


Prepared following general procedure B from *tert*-butyldimethyl((1-(o-tolyl)vinyl)oxy)silane **3r** (50.0 mg, 0.20 mmol, 1.0 equiv.) and sulfonium salt **1** (115.6 mg, 0.26 mmol, 1.3 equiv.). Compound **4r** was purified by chromatography on silica gel eluting with pentane/EtOAc (100/0→100/1 (v/v)) and obtained as a colorless oil (28.6 mg, 0.12 mmol, 59%).

**$^1$H NMR** (300 MHz, $CDCl_3$): δ = 7.59 (d, *J* = 7.4 Hz, 1H), 7.38 (td, *J* = 7.2, 1.4 Hz, 1H), 7.27 (t, *J* = 7.3 Hz, 2H), 3.14 (s, 2H), 2.48 (s, 3H), 1.18−1.07 (m, 2H), 0.94−0.79 (m, 2H) ppm.

**$^{13}$C{$^1$H} NMR** (101 MHz, $CDCl_3$) δ = 200.4, 138.5, 138.0, 132.2, 131.7, 127.1 (q, *J* = 273.8 Hz), 128.5, 125.9, 41.6, 21.3, 20.2 (q, *J* = 33.4 Hz), 8.9 (q, *J* = 2.5 Hz) ppm.

**$^{19}$F NMR** (377 MHz, $CDCl_3$) δ = − 70.8 ppm.

**HRMS-ESI (m/z)** calculated for $C_{13}H_{13}OF_3$ $[M+Na]^+$: 265.0811 ; found: 265.0812.

**IR** (ATR, neat) $\tilde{\nu}$ = 2929, 1693, 1601, 1571, 1487, 1456, 1398, 1349, 1306, 1263, 1200, 1152, 1121, 1069, 1036, 936, 859, 752, 719, 664, 613, 594, 539, 489, 450 $cm^{-1}$.

Synthesis of **4s**:

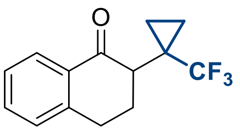

Prepared following general procedure B from *tert*-butyl((3,4-dihydronaphthalen-1-yl)oxy)dimethylsilane **3s** (52.1 mg, 0.20 mmol, 1.0 equiv.) and sulfonium salt **1** (115.7 mg, 0.26 mmol, 1.3 equiv.). Compound **4s** was purified by chromatography on silica gel eluting with pentane/EtOAc (100/0→200/1 (v/v)) and obtained as a colorless oil (13.5 mg, 0.05 mmol, 26%).

**$^1$H NMR** (300 MHz, $CDCl_3$): δ = 8.07 (dd, *J* = 7.8, 1.5 Hz, 1H), 7.48 (td, *J* = 7.4, 1.5 Hz, 1H), 7.38−7.20 (m, 2H), 3.13−2.91 (m, 2H), 2.43−2.14 (m, 3H), 1.39 (ddd, *J* = 9.9, 6.8, 5.4 Hz, 1H), 1.09−0.83 (m, 2H), 0.70−0.56 (m, 1H) ppm.

**$^{13}$C{$^1$H} NMR** (101 MHz, $CDCl_3$) δ = 195.7, 143.5, 133.6, 132.6, 128.7, 127.9, 127.1 (q, *J* = 275.7 Hz), 127.0, 52.0, 29.9, 27.6, 22.9 (q, *J* = 32.2 Hz), 10.6 (q, *J* = 3.1 Hz), 7.8 (q, *J* = 2.6 Hz) ppm.

**$^{19}$F NMR** (282 MHz, $CDCl_3$) δ = − 65.8 ppm.

**HRMS-ESI (m/z)** calculated for $C_{14}H_{13}OF_3$ [M+Na]$^+$: 277.0811; found: 277.0816.

**IR** (ATR, neat) $\tilde{\nu}$ = 2941, 2360, 2029, 1770, 1759, 1685, 1601, 1558, 1541, 1522, 1507, 1473, 1456, 1435, 1389, 1304, 1271, 1245, 1226, 1204, 1169, 1142, 1121, 1067, 1037, 958, 945, 904, 818, 791, 758, 742, 668, 614, 586, 538, 504, 492, 472, 446, 410 cm$^{-1}$.

Synthesis of **4t**:

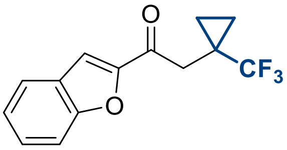

Prepared following general procedure B from ((1-(benzofuran-2-yl)vinyl)oxy)(*tert*-butyl)dimethylsilane **3t** (55.2 mg, 0.20 mmol, 1.0 equiv.) and sulfonium salt **1** (116.1 mg, 0.26 mmol, 1.3 equiv.). Compound **4t** was purified by chromatography on silica gel eluting with pentane/EtOAc (100/0→50/1 (v/v)). Obtained as a yellowish oil (46.8 mg, 0.17 mmol, 87%).

**$^1$H NMR** (400 MHz, $CDCl_3$): δ = 7.72 (dt, *J* = 7.9, 1.0 Hz, 1H), 7.58 (dq, *J* = 8.4, 1.0 Hz, 1H), 7.55 (d, *J* = 1.0 Hz, 1H), 7.49 (ddd, *J* = 8.5, 7.1, 1.3 Hz, 1H), 7.32 (ddd, *J* = 8.0, 7.1, 1.0 Hz, 1H), 3.22 (s, 2H), 1.15−1.10 (m, 2H), 0.95−0.90 (m, 2H) ppm.

**$^{13}$C{$^1$H} NMR** (101 MHz, $CDCl_3$) δ = 187.5, 155.9, 152.4, 128.7, 127.0 (q, *J* = 273.8 Hz), 127.1, 124.2, 123.6, 113.6, 112.6, 38.8, 20.2 (q, *J* = 33.4 Hz), 8.8 (q, *J* = 2.5 Hz) ppm.

**$^{19}$F NMR** (377 MHz, $CDCl_3$) δ = − 70.9 ppm.

**HRMS-ESI (m/z)** calculated for $C_{14}H_{11}F_3O_2$ [M+Na]$^+$: 291.0603; found: 291.0608.

**IR** (ATR, neat) $\tilde{\nu}$ = 2963, 2360, 1683, 1613, 1555, 1476, 1397, 1362, 1324, 1306, 1275, 1260, 1203, 1156, 1111, 1072, 1032, 980, 940, 916, 879, 828, 812, 753, 641, 613, 602, 590, 571, 553, 483, 458, 429, 402 cm$^{-1}$.

**Melting point:** 66.5 °C.

Synthesis of **4u**:

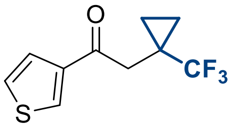


Prepared following general procedure B from *tert*-butyldimethyl((1-(thiophen-3-yl)vinyl)oxy)silane **3u** (48.0 mg, 0.20 mmol, 1.0 equiv.) and sulfonium salt **1** (115.0 mg, 0.26 mmol, 1.3 equiv.). Compound **4u** was purified by chromatography on silica gel eluting with pentane/EtOAc (100/0→100/1 (v/v)) and obtained as a colorless oil (43.5 mg, 0.19 mmol, 93%).

**$^1$H NMR** (400 MHz, $CDCl_3$): δ = 8.06 (dd, *J* = 2.9, 1.3 Hz, 1H), 7.54 (dd, *J* = 5.1, 1.3 Hz, 1H), 7.33 (dd, *J* = 5.1, 2.9 Hz, 1H), 3.14 (s, 2H), 1.13−1.07 (m, 2H), 0.89−0.83 (m, 2H) ppm.

**$^{13}$C{$^1$H} NMR** (101 MHz, $CDCl_3$) δ = 190.7, 142.4, 132.7, 127.1 (q, *J* = 273.3 Hz), 127.08, 126.81, 39.6, 20.1 (q, *J* = 33.4 Hz), 8.7 (q, *J* = 2.5 Hz) ppm.

**$^{19}$F NMR** (377 MHz, $CDCl_3$) δ = − 71.0 ppm.

**HRMS-ESI (m/z)** calculated for $C_{10}H_9F_3OS$ $[M+H]^+$: 235.0399; found: 235.0386.

**IR** (ATR, neat) $\tilde{\nu}$ = 3104, 2360, 1674, 1509, 1393, 1349, 1300, 1233, 1207, 1152, 1121, 1069, 1037, 980, 939, 895, 872, 781, 764, 727, 703, 637, 589, 546, 507, 446 $cm^{-1}$.

Synthesis of **4v**:

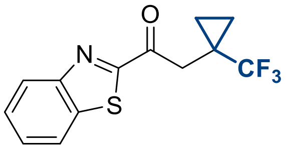


Prepared following general procedure B from 2-(1-((*tert*-butyldimethylsilyl)oxy)vinyl)benzo[*d*]thiazole **3v** (58.0 mg, 0.20 mmol, 1.0 equiv.) and sulfonium salt **1** (115.2 mg, 0.26 mmol, 1.3 equiv.). Compound **4v** was purified by chromatography on silica gel eluting with pentane/EtOAc (100/0→10/1 (v/v)) and obtained as a yellowish oil (42.9 mg, 0.15 mmol, 75%).

**$^1$H NMR** (400 MHz, $CDCl_3$): δ = 8.20−8.17 (m, 1H), 8.00−7.96 (m, 1H), 7.61−7.51 (m, 2H), 3.56 (s, 2H), 1.20−1.15 (m, 2H), 0.97−0.92 (m, 2H) ppm.

**$^{13}$C{$^1$H} NMR** (101 MHz, $CDCl_3$) δ = 191.8, 166.1, 153.6, 137.6, 126.9 (q, *J* = 273.3 Hz), 128.0, 127.2, 125.7, 122.6, 39.3, 19.9 (q, *J* = 34.3 Hz), 9.2 (q, *J* = 2.5 Hz) ppm.

**$^{19}$F NMR** (377 MHz, $CDCl_3$) δ = − 70.6 ppm.

**HRMS-ESI (m/z)** calculated for $C_{13}H_{10}F_3NOS$ $[M+H]^+$: 286.0508; found: 286.0514.

**IR** (ATR, neat) $\tilde{\nu}$ = 2939, 2357, 1691, 1550, 1486, 1429, 1398, 1319, 1298, 1235, 1198, 1169, 1148, 1117, 1068, 1042, 1000, 952, 931, 905, 856, 765, 736, 727, 702, 616, 602, 579, 566, 526, 475, 464, 436, 426 $cm^{-1}$.

Synthesis of **4w**:

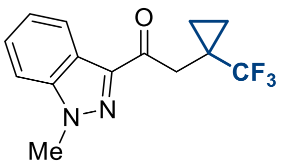


Prepared following general procedure B from 3-(1-((*tert*-butyldimethylsilyl)oxy)vinyl)-1-methyl-1*H*-indazole **3w** (58.1 mg, 0.20 mmol, 1.0 equiv.) and sulfonium salt **1** (114.3 mg, 0.26 mmol, 1.3 equiv.). Compound **4w** was purified by chromatography on silica gel eluting with pentane/EtOAc (100/0→10/1 (v/v)) and obtained as a yellowish oil (50.2 mg, 0.18 mmol, 89%).

**$^1$H NMR** (300 MHz, $CDCl_3$): δ = 8.36 (dt, *J* = 8.1, 1.1 Hz, 1H), 7.48−7.41 (m, 2H), 7.33 (ddd, *J* = 8.0, 5.2, 2.7 Hz, 1H), 4.14 (s, 3H), 3.44 (s, 2H), 1.16−1.09 (m, 2H), 0.97−0.90 (m, 2H) ppm.

**$^{13}$C{$^1$H} NMR** (101 MHz, $CDCl_3$) δ = 193.1, 141.9, 141.4, 127.2 (q, *J* = 273.3 Hz), 127.2, 123.9, 123.0, 123.0, 109.4, 38.9, 36.5, 20.0 (q, *J* = 33.6 Hz), 8.9 (q, *J* = 2.5 Hz) ppm.

**$^{19}$F NMR** (282 MHz, $CDCl_3$) δ = − 70.7 ppm.

**HRMS-ESI (m/z)** calculated for $C_{14}H_{13}F_3N_2O$ $[M+H]^+$: 283.1053; found: 283.1066.

**IR** (ATR, neat) $\tilde{\nu}$ = 3025, 2950, 2361, 1680, 1618, 1578, 1497, 1474, 1426, 1390, 1344, 1300, 1253, 1195, 1170, 1146, 1116, 1076, 1061, 1030, 1003, 950, 931, 915, 859, 846, 803, 772, 754, 734, 661, 615, 574, 548, 534, 506, 458, 431, 412 $cm^{-1}$.

Synthesis of **4x**:

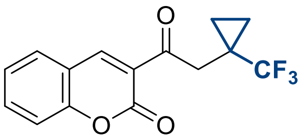


Prepared following general procedure B from 3-(1-((*tert*-butyldimethylsilyl)oxy)vinyl)-2*H*-chromen-2-one **3x** (60.5 mg, 0.20 mmol, 1.0 equiv.) and sulfonium salt **1** (115.3 mg, 0.26 mmol, 1.3 equiv.). Compound **4x** was purified by chromatography on silica gel eluting with pentane/EtOAc (100/0→5/1 (v/v)) and obtained as a yellowish oil (45.9 mg, 0.16 mmol, 77%).

**$^1$H NMR** (300 MHz, $CDCl_3$): δ = 8.48 (s, 1H), 7.71−7.62 (m, 2H), 7.40−7.32 (m, 2H), 3.42 (s, 2H), 1.17−1.11 (m, 2H), 0.87−0.80 (m, 2H) ppm.

**$^{13}$C{$^1$H} NMR** (101 MHz, $CDCl_3$) δ = 194.5, 159.4, 155.3, 148.0, 134.7, 130.4, 127.1 (q, *J* = 273.3 Hz), 125.3, 124.7, 118.4, 116.9, 43.6, 19.6 (q, *J* = 34.1 Hz), 9.3 (q, *J* = 2.5 Hz) ppm.

**$^{19}$F NMR** (282 MHz, $CDCl_3$) δ = − 70.4 ppm.

**HRMS-ESI (m/z)** calculated for $C_{15}H_{11}F_3O_3$ $[M+H]^+$: 297.0733; found: 297.0745.

**IR** (ATR, neat) $\tilde{\nu}$ = 3056, 1727, 1691, 1608, 1558, 1446, 1399, 1365, 1312, 1290, 1224, 1189, 1159, 1111, 1063, 1030, 976, 951, 922, 883, 856, 758, 724, 639, 612, 580, 539, 518, 455, 429, 412 $cm^{-1}$.

Synthesis of **4y**:

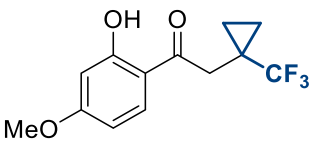


Prepared following general procedure B from *tert*-butyl((1-(2-((*tert*-butyldimethylsilyl)oxy)-4-methoxyphenyl)vinyl)oxy)dimethylsilane **3y** (48.0 mg, 0.20 mmol, 1.0 equiv.) and sulfonium salt **1** (115.1 mg, 0.26 mmol, 1.3 equiv.). Compound **4y** was purified by chromatography on silica gel eluting with pentane/EtOAc (100/0→100/1 (v/v)) and obtained as a colorless oil (23.0 mg, 0.08 mmol, 42%).

**$^{1}$H NMR** (400 MHz, $CDCl_3$): δ = 12.68 (s, 1H), 7.63 (d, *J* = 8.9 Hz, 1H), 6.50−6.40 (m, 2H), 3.85 (s, 3H), 3.18 (s, 2H), 1.15−1.07 (m, 2H), 0.90−0.79 (m, 2H) ppm.

**$^{13}$C{$^{1}$H} NMR** (101 MHz, $CDCl_3$) δ = 200.7, 166.6, 166.0, 131.9, 127.0 (q, *J* = 273.6 Hz), 113.7, 108.2, 101.2, 55.8, 37.5, 20.3 (q, *J* = 33.7 Hz), 8.8 (q, *J* = 2.5 Hz) ppm.

**$^{19}$F NMR** (377 MHz, $CDCl_3$) δ = − 71.0 ppm.

**HRMS-ESI (m/z)** calculated for $C_{13}H_{13}O_3F_3$ $[M+Na]^+$: 297.0709; found: 297.0711.

**IR** (ATR, neat) $\tilde{\nu}$ = 3734, 2962, 2360, 2341, 2101, 1634, 1574, 1508, 1444, 1401, 1365, 1290, 1258, 1228, 1207, 1153, 1123, 1068, 1012, 862, 791, 702, 590, 575, 458 $cm^{-1}$.

Synthesis of **4z**:

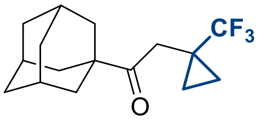


Prepared following general procedure B from ((1-((3*r*,5*r*,7*r*)-adamantan-1-yl)vinyl)oxy)(*tert*-butyl)dimethylsilane **3z** (59.0 mg, 0.20 mmol, 1.0 equiv.) and sulfonium salt **1** (116.0 mg, 0.26 mmol, 1.3 equiv.). Compound **4z** was purified by chromatography on silica gel eluting with pentane/EtOAc (100/0→200/1 (v/v)) and obtained as a colorless solid (6.9 mg, 0.024 mmol, 12%).

**$^{1}$H NMR** (400 MHz, $CDCl_3$): δ = 2.69 (s, 2H), 2.08−2.02 (m, 3H), 1.82−1.62 (m, 12H), 1.10−1.02 (m, 2H), 0.81−0.72 (m, 2H) ppm.

**$^{13}$C{$^{1}$H} NMR** (101 MHz, $CDCl_3$) δ = 211.3, 127.2 (q, *J* = 273.6 Hz), 46.9, 37.9, 36.6, 36.4, 27.9, 19.4 (q, *J* = 33.4 Hz), 8.8 (q, *J* = 2.6 Hz) ppm.

**$^{19}$F NMR** (377 MHz, $CDCl_3$) δ = − 71.0 ppm.

**HRMS-ESI (m/z)** calculated for $C_{16}H_{21}OF_3$ $[M+Na]^+$: 309.1437; found: 309.1437.

**IR** (ATR, neat) $\tilde{\nu}$ = 2911, 2885, 2853, 2682, 2085, 1701, 1567, 1450, 1398, 1362, 1350, 1303, 1267, 1207, 1166, 1148, 1112, 1063, 1052, 1033, 992, 959, 938, 927, 854, 811, 782, 764, 730, 674, 647, 624, 587, 523, 485, 458, 426, 414 $cm^{-1}$.

**Melting point:** 75 °C.

## CATALYST-FREE TRANSFER OF TFCp

General procedure **C** for the synthesis of (**6a**-**w**):

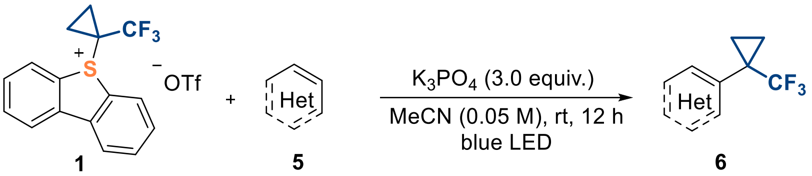


A Schlenk flask equipped with a magnetic stir bar was charged with sulfonium salt **1** (0.2 mmol, 1.0 equiv.), the desired substrate (0.4 mmol, 2.0 equiv.) and $K_3PO_4$ (0.6 mmol, 3.0 equiv.). Subsequently, MeCN (4 mL) was added, the flask was sealed ,transferred to a photoreactor equipped with blue LED strips (maximum wavelength: 462 nm), and irradiated at 50% intensity for 12 h. The reaction mixture was diluted with DCM (3 mL) and purified by silica gel column chromatography with the indicated solvent mixtures.

Synthesis of **6a**:

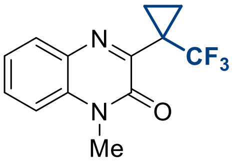


Prepared following general procedure C from 1-methylquinoxalin-2(1*H*)-one **5a** (64.1 mg, 0.40 mmol, 2.0 equiv.), $K_3PO_4$ (127.4 mg, 0.60 mmol, 3.0 equiv.) and **1** (88.5 mg, 0.20 mmol, 1.0 equiv.). Compound **6a** was purified by chromatography on silica gel eluting with pentane/EtOAc (100/0→80/20 (v/v)) and obtained as a light-yellow solid (35.2 mg, 0.13 mmol, 66%).

**$^1$H NMR** (300 MHz, $CDCl_3$): δ = 7.91 (ddd, *J* = 8.0, 1.5, 0.5 Hz, 1H), 7.60 (ddd, *J* = 8.7, 7.3, 1.5 Hz, 1H), 7.40−7.29 (m, 2H), 3.72 (s, 3H), 1.56−1.51 (m, 2H), 1.36−1.30 (m, 2H) ppm.

**$^{13}$C{$^1$H} NMR** (101 MHz, $CDCl_3$) δ = 154.4, 153.3, 133.9, 132.5, 131.5, 130.8, 125.9 (q, *J* = 274.8 Hz), 123.9, 113.8, 29.4, 28.1 (q, *J* = 34.5 Hz), 10.4 (q, *J* = 2.3 Hz) ppm.

**$^{19}$F NMR** (288 MHz, $CDCl_3$) δ = − 67.2 ppm.

**HRMS-ESI (m/z)** calculated for $C_{13}H_{11}F_3N_2O$ $[M+Na]^+$: 291.0716; found: 291.0723.

**IR** (ATR, neat) $\tilde{\nu}$ = 1654, 1601, 1588, 1554, 1470, 1434, 1416, 1365, 1350, 1329, 1316, 1266, 1186, 1164, 1148, 1128, 1078, 1065, 1037, 995, 965, 951, 932, 867, 788, 767, 757, 715, 668, 650, 592, 580, 568, 561, 525, 512, 503, 500, 494, 482, 473, 459, 443, 432, 403 $cm^{-1}$.

**Melting point:** 138 – 139 °C.

Synthesis of **6b**:

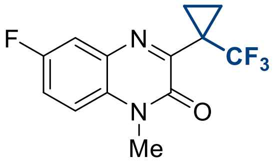


Prepared following general procedure C from 6-fluoro-1-methylquinoxalin-2(1*H*)-one **5b** (71.0 mg, 0.40 mmol, 2.0 equiv.), $K_3PO_4$ (127.4 mg, 0.60 mmol, 3.0 equiv.) and **1** (88.6 mg, 0.20 mmol, 1.0 equiv.). Compound **6b** was purified by chromatography on silica gel eluting with pentane/$Et_2O$ (100/0→60/40 (v/v)), and obtained as a yellow solid (26.9 mg, 0.09 mmol, 47%).

**$^1$H NMR** (300 MHz, $CDCl_3$): δ = 7.60 (dd, *J* = 8.6, 2.8 Hz, 1H), 7.41−7.23 (m, 2H), 3.70 (s, 3H), 1.59−1.48 (m, 2H), 1.39−1.34 (m, 2H) ppm.

**$^{13}$C{$^1$H} NMR** (101 MHz, $CDCl_3$) δ = 159.9, 157.6, 154.9, 153.9, 132.9 (d, *J* = 11.3 Hz), 130.5 (d, *J* = 2.3 Hz), 125.8 (q, *J* = 275.0 Hz), 119.3 (d, *J* = 24.2 Hz), 116.1 (d, *J* = 22.5 Hz), 114.9 (d, *J* = 8.7 Hz), 29.7, 28.3 (q, *J* = 34.4 Hz), 10.5 (q, *J* = 2.2 Hz) ppm.

**$^{19}$F NMR** (288 MHz, $CDCl_3$) δ = − 67.1, − 118.60 (td, *J* = 8.1, 4.7 Hz) ppm.

**HRMS-ESI (m/z)** calculated for $C_{13}H_{10}F_4N_2O$ $[M+H]^+$: 287.0802; found: 287.0808.

**IR** (ATR, neat) $\tilde{\nu}$ = 3094, 3073, 3047, 2952, 2922, 2851, 1661, 1618, 1589, 1350, 1334, 1313, 1176, 1151, 1111, 1067, 1038, 1002, 849, 834, 815 $cm^{-1}$.

**Melting point:** 158 – 163 °C.

Synthesis of **6c**:

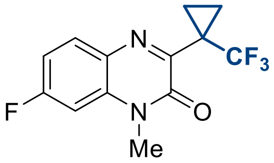


Prepared following general procedure C from 7-fluoro-1-methylquinoxalin-2(1*H*)-one **5c** (71.1 mg, 0.40 mmol, 2.0 equiv.), $K_3PO_4$ (127.3 mg, 0.60 mmol, 3.0 equiv.) and **1** (88.5 mg, 0.20 mmol, 1.0 equiv.). Compound **6c** was purified by chromatography on silica gel eluting with pentane/EtOAc (100/0→85/15 (v/v)) and obtained as a light-yellow solid (35.0 mg, 0.12 mmol, 60%).

**$^1$H NMR** (300 MHz, $CDCl_3$): δ = 7.88 (dd, *J* = 8.9, 5.9 Hz, 1H), 7.07 (ddd, *J* = 8.9, 8.1, 2.6 Hz, 1H), 6.99 (dd, *J* = 10.0, 2.6 Hz, 1H), 3.66 (s, 3H), 1.55−1.49 (m, 2H), 1.34−1.28 (m, 2H) ppm.

**$^{13}$C{$^1$H} NMR** (101 MHz, $CDCl_3$) δ = 165.5, 162.9, 154.2, 152.1 (d, *J* = 3.6 Hz), 135.5 (d, *J* = 12.0 Hz), 132.9 (d, *J* = 10.6 Hz), 129.3 (d, *J* = 2.4 Hz), 125.9 (q, *J* = 274.7 Hz), 111.9 (d, *J* = 23.5 Hz), 100.7 (d, *J* = 28.0 Hz), 29.6, 27.9 (q, *J* = 34.6 Hz), 10.4 (q, *J* = 2.3 Hz) ppm.

**$^{19}$F NMR** (288 MHz, $CDCl_3$) δ = − 67.3, − 105.2 (ddd, *J* = 10.2, 8.1, 5.9 Hz) ppm.

**HRMS-ESI (m/z)** calculated for $C_{13}H_{10}F_4N_2O$ $[M+H]^+$: 287.0802; found: 287.0814.

**IR** (ATR, neat) $\tilde{\nu}$ = 3094, 3073, 3047, 2952, 2922, 2851, 1661, 1618, 1589, 1350, 1334, 1313, 1176, 1151, 1111, 1067, 1038, 1002, 849, 834, 815 $cm^{-1}$.

**Melting point:** 158 – 160 °C.

Synthesis of **6d**:

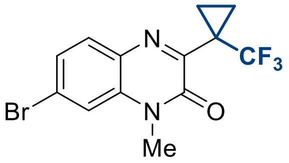

Prepared following general procedure C from 7-bromo-1-methylquinoxalin-2(1*H*)-one **5d** (96.0 mg, 0.40 mmol, 2.0 equiv.), $K_3PO_4$ (127.5 mg, 0.60 mmol, 3.0 equiv.) and **1** (88.5 mg, 0.20 mmol, 1.0 equiv.). Compound **6d** was purified by chromatography on silica gel eluting with pentane/EtOAc (100/0→80/20 (v/v)) and obtained as a white solid (18.1 mg, 0.05 mmol, 25%).

**$^1$H NMR** (300 MHz, $CDCl_3$): δ = 7.74 (d, *J* = 9.0 Hz, 1H), 7.50−7.43 (m, 2H), 3.67 (s, 3H), 1.56−1.48 (m, 2H), 1.38−1.29 (m, 2H) ppm.

**$^{13}$C{$^1$H} NMR** (101 MHz, $CDCl_3$) δ = 153.9, 153.6, 134.8, 131.9, 131.3, 127.24, 125.83, 125.7 (q, *J* = 275.0 Hz), 116.9, 29.5, 28.2 (q, *J* = 34.5 Hz), 10.4 (q, *J* = 2.2 Hz) ppm.

**$^{19}$F NMR** (288 MHz, $CDCl_3$) δ = − 67.2 ppm.

**HRMS-ESI (m/z)** calculated for $C_{13}H_{10}BrF_3N_2O$ $[M+Na]^+$: 368.9821; found: 368.9838.

**IR** (ATR, neat) $\tilde{\nu}$ = 1660, 1591, 1554, 1431, 1341, 1313, 1174, 1149, 1134, 1117, 1074, 1038, 995, 936, 848, 835, 795, 787, 682 $cm^{-1}$.

**Melting point:** 210 – 213 °C.

Synthesis of **6e**:

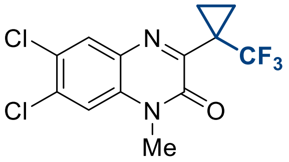

Prepared following general procedure C from 6,7-dichloro-1-methylquinoxalin-2(1*H*)-one **5e** (92.2 mg, 0.40 mmol, 2.0 equiv.), $K_3PO_4$ (127.4 mg, 0.60 mmol, 3.0 equiv.) and **1** (88.5 mg, 0.20 mmol, 1.0 equiv.). Compound **6e** was purified by chromatography on silica gel eluting with pentane/EtOAc (100/0→80/20 (v/v)) and obtained as a white solid (24.9 mg, 0.08 mmol, 38%).

**$^1$H NMR** (300 MHz, $CDCl_3$): δ = 7.98 (s, 1H), 7.40 (s, 1H), 3.66 (s, 3H), 1.56−1.48 (m, 2H), 1.42−1.32 (m, 2H) ppm.

**$^{13}$C{$^1$H} NMR** (101 MHz, $CDCl_3$) δ = 154.8, 153.6, 135.7, 133.1, 131.5, 127.8, 125.7 (d, *J* = 274.9 Hz), 115.3, 29.7, 28.3 (q, *J* = 34.5 Hz), 10.5 (q, *J* = 2.2 Hz) ppm.

**$^{19}$F NMR** (288 MHz, $CDCl_3$) δ = -67.1 ppm.

**HRMS-ESI (m/z)** calculated for $C_{13}H_9Cl_2F_3N_2O$ $[M+H]^+$: 337.0117; found: 337.0118.

**IR** (ATR, neat) $\tilde{\nu}$ = 1673, 1654, 1593, 1460, 1359, 1338, 1173, 1154, 1128, 1086, 1072, 728, 691, 584, 518, 507, 454, 423, 418, 404 $cm^{-1}$.

**Melting point:** 139- 141 °C.

Synthesis of **6f**:

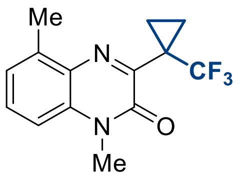

Prepared following general procedure C from 1,5-dimethylquinoxalin-2(1*H*)-one **5f** (69.6 mg, 0.40 mmol, 2.0 equiv.), $K_3PO_4$ (127.6 mg, 0.60 mmol, 3.0 equiv.) and **1** (88.5 mg, 0.20 mmol, 1.0 equiv.). Compound **6f** was purified by chromatography on silica gel eluting with pentane/EtOAc (100/0→80/20 (v/v)) and obtained as a white solid (25.7 mg, 0.09 mmol, 46%).

**$^1$H NMR** (300 MHz, $CDCl_3$): δ = 7.47 (t, *J* = 7.9 Hz, 1H), 7.21 (d, *J* = 7.5 Hz, 1H), 7.14 (d, *J* = 8.4 Hz, 1H), 3.69 (s, 3H), 2.69 (s, 3H), 1.52−1.41 (m, 4H) ppm.

**$^{13}$C{$^1$H} NMR** (101 MHz, $CDCl_3$) δ = 154.2, 151.1, 139.7, 133.8, 131.1, 131.1, 126.0 (q, *J* = 274.7 Hz), 125.1, 111.6, 29.4, 28.5 (q, *J* = 33.8 Hz), 17.5, 10.4 (q, *J* = 2.3 Hz) ppm.

**$^{19}$F NMR** (288 MHz, $CDCl_3$) δ = − 67.2 ppm.

**HRMS-ESI (m/z)** calculated for $C_{14}H_{13}F_3N_2O$ $[M+H]^+$: 283.1053; found: 283.1053.

**IR** (ATR, neat) $\tilde{\nu}$ = 2918, 2851, 2360, 2337, 1650, 1621, 1576, 1543, 1471, 1354, 1311, 1173, 1151, 1113, 1062, 1038, 907, 842, 785, 742, 667, 617, 521, 504, 484, 461, 435, 416 $cm^{-1}$.

**Melting point:** 121 °C.

Synthesis of **6g**:

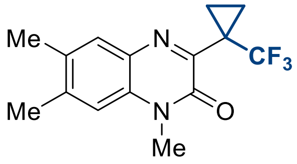

Prepared following general procedure C from 1,6,7-trimethylquinoxalin-2(1*H*)-one **5g** (75.3 mg, 0.40 mmol, 2.0 equiv.), $K_3PO_4$ (127.4 mg, 0.60 mmol, 3.0 equiv.) and **1** (88.5 mg, 0.20 mmol, 1.0 equiv.). Compound **6g** was purified by chromatography on silica gel eluting with pentane/EtOAc (100/0→90/10 (v/v)) and obtained as a white solid (27.2 mg, 0.09 mmol, 46%).

**$^1$H NMR** (300 MHz, $CDCl_3$): δ = 7.65 (s, 1H), 7.07 (s, 1H), 3.68 (s, 3H), 2.42 (s, 3H), 2.34 (s, 3H), 1.56−1.46 (m, 2H), 1.34−1.25 (m, 2H) ppm.

**$^{13}$C{$^1$H} NMR** (101 MHz, $CDCl_3$) δ = 154.5, 151.7, 141.7, 132.9, 131.9, 131.0, 130.7, 126.0 (q, *J* = 274.8 Hz), 114.3, 29.3, 27.9 (q, *J* = 34.4 Hz), 20.8, 19.2, 10.3 (q, *J* = 2.3 Hz) ppm.

**$^{19}$F NMR** (288 MHz, $CDCl_3$) δ = − 67.4 ppm.

**HRMS-ESI (m/z)** calculated for $C_{15}H_{15}F_3N_2O$ $[M+H]^+$: 297.1209; found: 297.1207.

**IR** (ATR, neat) $\tilde{\nu}$ = 2360, 2340, 1650, 1595, 1481, 1337, 1176, 1150, 1110, 1027, 783, 742, 666, 623, 524, 489, 460, 406 $cm^{-1}$.

**Melting point:** 120 °C.

Synthesis of **6h**:

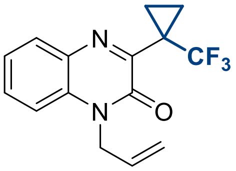


Prepared following general procedure C from 1-allylquinoxalin-2(1*H*)-one **5h** (74.2 mg, 0.40 mmol, 2.0 equiv.), $K_3PO_4$ (127.4 mg, 0.60 mmol, 3.0 equiv.) and **1** (88.6 mg, 0.20 mmol, 1.0 equiv.). Compound **6h** was purified by chromatography on silica gel eluting with pentane/EtOAc (100/0→80/20 (v/v)) and obtained as a yellow solid (21.9 mg, 0.07 mmol, 37%).

**$^1$H NMR** (300 MHz, $CDCl_3$): δ = 7.91 (dd, *J* = 8.0, 1.5 Hz, 1H), 7.56 (ddd, *J* = 8.5, 7.2, 1.6 Hz, 1H), 7.41−7.26 (m, 2H), 6.02−5.84 (m, 1H), 5.28 (ddd, *J* = 10.5, 1.7, 0.9 Hz, 1H), 5.18 (ddd, *J* = 17.2, 1.8, 1.0 Hz, 1H), 4.91 (dt, *J* = 5.3, 1.8 Hz, 2H), 1.57−1.50 (m, 2H), 1.41−1.33 (m, 2H) ppm.

**$^{13}$C{$^1$H} NMR** (101 MHz, $CDCl_3$) δ = 153.9, 153.3, 133.1, 132.7, 131.4, 130.9, 130.6, 125.9 (q, *J* = 274.9 Hz), 123.9, 118.5, 114.3, 44.9, 28.1 (q, *J* = 34.4 Hz), 10.4 (q, *J* = 2.7 Hz) ppm.

**$^{19}$F NMR** (288 MHz, $CDCl_3$) δ = − 67.2 ppm.

**HRMS-ESI (m/z)** calculated for $C_{15}H_{13}F_3N_2O$ $[M+H]^+$: 295.1053; found: 295.1060.

**IR** (ATR, neat) $\tilde{\nu}$ = 1653, 1641, 1602, 1436, 1362, 1333, 1174, 1150, 1127, 1081, 1073, 1039, 1009, 947, 756, 741 $cm^{-1}$.

**Melting point:** 86 − 87 °C.

Synthesis of **6i**:

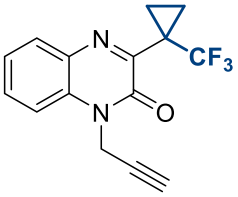


Prepared following general procedure C from methyl 1-(prop-2-yn-1-yl)-quinoxalin-2(1H)-one **5i** (73.7 mg, 0.40 mmol, 2.0 equiv.), $K_3PO_4$ (127.4 mg, 0.60 mmol, 3.0 equiv.) and **1** (88.6 mg, 0.20 mmol, 1.0 equiv.). Compound **6i** was purified by chromatography on silica gel eluting with pentane/EtOAc (100/0→85/15 (v/v)) and obtained as an off-white solid (23.8 mg, 0.08 mmol, 41%).

**$^1$H NMR** (300 MHz, $CDCl_3$): δ = 7.92 (dd, *J* = 8.0, 1.5 Hz, 1H), 7.63 (ddd, *J* = 8.6, 7.2, 1.5 Hz, 1H), 7.48 (dd, *J* = 8.5, 1.2 Hz, 1H), 7.39 (ddd, *J* = 8.4, 7.3, 1.2 Hz, 1H), 5.06 (d, *J* = 2.5 Hz, 2H), 2.30 (t, *J* = 2.6 Hz, 1H), 1.56−1.50 (m, 2H), 1.41−1.33 (m, 2H) ppm.

**$^{13}$C{$^1$H} NMR** (101 MHz, $CDCl_3$) δ = 153.3, 153.2, 132.7, 132.3, 131.6, 130.9, 125.8 (q, *J* = 274.7 Hz), 124.3, 114.3, 76.7, 73.6, 31.8, 28.1 (q, *J* = 34.5 Hz), 10.4 (q, *J* = 2.3 Hz) ppm.

**$^{19}$F NMR** (288 MHz, $CDCl_3$) δ = − 67.2 ppm.

**HRMS-ESI (m/z)** calculated for $C_{15}H_{11}F_3N_2O$ $[M+H]^+$: 293.0896; found: 293.0892.

**IR** (ATR, neat) $\tilde{\nu}$ = 3249, 2967, 2126, 1979, 1656, 1602, 1561, 1469, 1433, 1362, 1332, 1314, 1267, 1213, 1160, 1149, 1131, 1115, 1084, 1067, 1044, 1024, 983, 963, 943, 916, 875, 800, 787, 766, 753, 734, 692, 678, 647, 619, 588, 566, 554, 507, 481, 463, 437, 417 $cm^{-1}$.

**Melting point:** 148 – 149 °C.

Synthesis of **6j**:

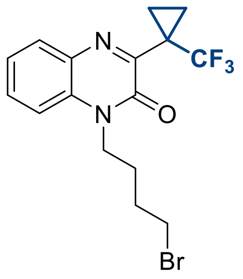

Prepared following general procedure C from 1-(4-bromobutyl)quinoxalin-2(1*H*)-one **5j** (112.5 mg, 0.40 mmol, 2.0 equiv.), $K_3PO_4$ (127.4 mg, 0.60 mmol, 3.0 equiv.) and **1** (88.6 mg, 0.20 mmol, 1.0 equiv.). Compound **6j** was purified by chromatography on silica gel eluting with pentane/EtOAc (100/0→80/20 (v/v)) and obtained as an off-white solid (37.5 mg, 0.10 mmol, 48%).

**$^1$H NMR** (400 MHz, $CDCl_3$): δ = 7.92 (dd, *J* = 7.9, 1.6 Hz, 1H), 7.61 (ddd, *J* = 8.6, 7.3, 1.6 Hz, 1H), 7.41−7.31 (m, 2H), 4.30 (t, *J* = 7.1 Hz, 2H), 3.49 (t, *J* = 6.2 Hz, 2H), 2.07−1.90 (m, 4H), 1.57−1.50 (m, 2H), 1.37−1.31 (m, 2H) ppm.

**$^{13}$C{$^1$H} NMR** (101 MHz, $CDCl_3$) δ = 154.1, 153.2, 132.9, 132.8, 131.6, 131.1, 125.9 (q, *J* = 275.0 Hz), 123.9, 113.6, 41.6, 33.0, 29.9, 28.0 (q, *J* = 34.4 Hz), 25.9, 10.4 (q, *J* = 2.4 Hz) ppm.

**$^{19}$F NMR** (377 MHz, $CDCl_3$) δ = − 67.2 ppm.

**HRMS-ESI (m/z)** calculated for $C_{16}H_{16}BrF_3N_2O$ $[M+H]^+$: 389.0471; found: 389.0467.

**IR** (ATR, neat) $\tilde{\nu}$ = 2959, 2359, 1654, 1603, 1589, 1560, 1469, 1432, 1362, 1342, 1307, 1254, 1222, 1153, 1128, 1083, 1037, 949, 910, 800, 753, 668, 649, 627, 593, 562, 484, 463, 440, 404 $cm^{-1}$.

**Melting point:** 99 – 100 °C.

Synthesis of **6k**:

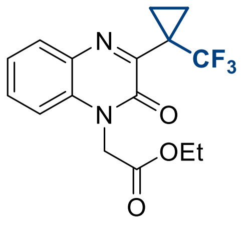

Prepared following general procedure C from ethyl 2-(2-oxoquinoxalin-1(2*H*)-yl)acetate **5k** (92.9 mg, 0.40 mmol, 2.0 equiv.), $K_3PO_4$ (127.4 mg, 0.60 mmol, 3.0 equiv.) and **1** (88.6 mg, 0.20 mmol, 1.0 equiv.). Compound **6k** was purified by chromatography on silica gel eluting with pentane/EtOAc (100/0→75/25 (v/v)) and obtained as an off-white solid (36.0 mg, 0.10 mmol, 52%).

**$^1$H NMR** (300 MHz, $CDCl_3$): δ = 7.92 (dd, *J* = 8.0, 1.5 Hz, 1H), 7.56 (ddd, *J* = 8.6, 7.3, 1.6 Hz, 1H), 7.36 (ddd, *J* = 8.3, 7.3, 1.2 Hz, 1H), 7.07 (dd, *J* = 8.4, 1.2 Hz, 1H), 5.02 (s, 2H), 4.24 (q, *J* = 7.2 Hz, 2H), 1.56−1.48 (m, 2H), 1.42−1.33 (m, 2H), 1.27 (t, *J* = 7.1 Hz, 3H) ppm.

**$^{13}$C{$^1$H} NMR** (101 MHz, $CDCl_3$) δ = 167.0, 153.9, 153.1, 133.0, 132.6, 131.6, 131.1, 125.8 (q, *J* = 274.7 Hz), 124.2, 113.2, 62.3, 43.8, 28.1 (q, *J* = 34.5 Hz), 14.2, 10.4 (q, *J* = 2.3 Hz) ppm.

**$^{19}$F NMR** (288 MHz, $CDCl_3$) δ = − 67.3 ppm.

**HRMS-ESI (m/z)** calculated for $C_{16}H_{15}F_3N_2O_3$ $[M+H]^+$: 341.1108; found: 341.1107.

**IR** (ATR, neat) $\tilde{\nu}$ = 405, 465, 500, 554, 566, 596, 654, 675, 712, 762, 801, 860, 889, 925, 951, 966, 992, 1018, 1043, 1074, 1091, 1132, 1171, 1204, 1219, 1267, 1319, 1346, 1363, 1416, 1467, 1562, 1604, 1666, 1742, 2360, 2954 $cm^{-1}$.

**Melting point:** 135 – 136 °C.

Synthesis of **6l**:

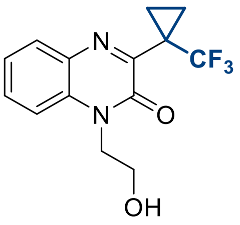

Prepared following general procedure C from 1-(2-hydroxyethyl)quinoxalin-2(1*H*)-one **5l** (76.0 mg, 0.40 mmol, 2.0 equiv.), $K_3PO_4$ (127.4 mg, 0.60 mmol, 3.0 equiv.) and **1** (88.6 mg, 0.20 mmol, 1.0 equiv.). Compound **6l** was purified by chromatography on silica gel eluting with pentane/EtOAc (100/0→33/67 (v/v)) and obtained as an off-white solid (29.2 mg, 0.10 mmol, 48%).

**$^1$H NMR** (300 MHz, $CDCl_3$): δ = 7.93 (dd, *J* = 8.0, 1.6 Hz, 1H), 7.60 (ddd, *J* = 8.6, 7.2, 1.6 Hz, 1H), 7.50−7.30 (m, 2H), 4.51 (t, *J* = 5.6 Hz, 2H), 4.05 (q, *J* = 5.3 Hz, 2H), 2.43 (t, *J* = 5.0 Hz, 1H), 1.60−1.48 (m, 2H), 1.43−1.30 (m, 2H) ppm.

**$^{13}$C{$^1$H} NMR** (101 MHz, $CDCl_3$) δ = 155.3, 153.0, 133.4, 132.9, 131.6, 131.1, 125.9 (q, *J* = 274.8 Hz), 124.1, 113.9, 60.8, 45.3, 28.0 (q, *J* = 34.5 Hz), 10.4 (q, *J* = 2.3 Hz) ppm.

**$^{19}$F NMR** (282 MHz, $CDCl_3$) δ = − 67.2 ppm.

**HRMS-ESI (m/z)** calculated for $C_{14}H_{13}F_3N_2O_2$ $[M+H]^+$: 299.1002; found: 299.1001.

**IR** (ATR, neat) $\tilde{\nu}$ = 404, 426, 459, 485, 553, 566, 656, 669, 712, 763, 788, 800, 850, 881, 951, 1010, 1034, 1075, 1092, 1126, 1173, 1201, 1275, 1314, 1332, 1347, 1361, 1428, 1467, 1561, 1601, 1648, 1743, 2341, 2360, 2958, 3447 $cm^{-1}$.

**Melting point:** 129 – 130 °C.

Synthesis of **6m**:

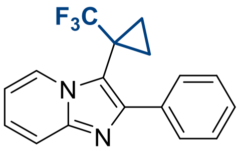

Prepared following general procedure C from 2-phenylimidazo[1,2-*a*]pyridine **5m** (116.6 mg, 0.60 mmol, 3.0 equiv.), $K_3PO_4$ (127.4 mg, 0.60 mmol, 3.0 equiv.) and **1** (88.5 mg, 0.20 mmol, 1.0 equiv.). Compound **5m** was purified by chromatography on silica gel eluting with pentane/EtOAc (100/0→9/1 (v/v)) and obtained as a yellow solid (49.3 mg, 0.16 mmol, 82%).

**$^1$H NMR** (400 MHz, $CDCl_3$): δ = 8.32 (dd, *J* = 7.0, 1.1 Hz, 1H), 8.03−7.94 (m, 2H), 7.68 (dt, *J* = 9.0, 1.2 Hz, 1H), 7.52−7.43 (m, 2H), 7.42−7.35 (m, 1H), 7.32−7.23 (m, 1H), 6.90 (td, *J* = 6.9, 1.2 Hz, 1H), 1.63 (d, *J* = 33.9 Hz, 2H), 0.97−0.86 (m, 2H) ppm.

**$^{13}$C{$^1$H} NMR** (101 MHz, $CDCl_3$) δ = 146.8, 145.2, 133.7, 128.8 (q, *J* = 1.6 Hz), 128.4, 128.3, 126.8 (q, *J* = 275.3 Hz), 125.5, 124.5 (q, *J* = 2.5 Hz), 117.9, 114.2, 112.7, 18.4 (q, *J* = 35.4 Hz), 12.6 (bd, *J* = 61.1 Hz) ppm.

**$^{19}$F NMR** (377 MHz, $CDCl_3$) δ = − 67.1 ppm.

**HRMS-ESI (m/z)** calculated for $C_{17}H_{13}F_3N_2$ $[M+H]^+$: 303.1104; found: 303.1110.

**IR** (ATR, neat) $\tilde{\nu}$ = 3041, 2360, 1717, 1636, 1558, 1540, 1507, 1447, 1426, 1398, 1364, 1341, 1282, 1260, 1235, 1177, 1145, 1125, 1110, 1075, 1035, 1024, 960, 912, 845, 832, 777, 755, 740, 716, 702, 691, 638, 619, 586, 559, 513, 469, 453, 433, 404 $cm^{-1}$.

**Melting point:** 143 – 144 °C.

Synthesis of **6n**:

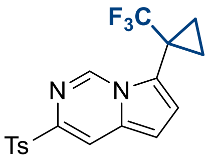


Prepared following general procedure C from 3-tosylpyrrolo[1,2-*c*]pyrimidine **5n** (163.4 mg, 0.60 mmol, 3.0 equiv.), $K_3PO_4$ (127.4 mg, 0.60 mmol, 3.0 equiv.) and **1** (88.5 mg, 0.20 mmol, 1.0 equiv.). Compound **6n** was purified by chromatography on silica gel eluting with pentane/EtOAc (100/0→70/30 (v/v)) and obtained as a yellow oil (47.6 mg, 0.13 mmol, 63%).

**$^1$H NMR** (300 MHz, $CDCl_3$): δ = 8.97 (s, 1H), 8.25 (d, *J* = 1.4 Hz, 1H), 8.00−7.92 (m, 2H), 7.33 (d, *J* = 8.1 Hz, 2H), 7.05 (d, *J* = 4.0 Hz, 2H), 6.79 (dd, *J* = 4.0, 0.9 Hz, 1H), 2.42 (s, 3H), 1.61−1.54 (m, 2H), 1.16 −1.10 (m, 2H) ppm.

**$^{13}$C{$^1$H} NMR** (101 MHz, $CDCl_3$) δ = δ 144.8, 141.9, 137.2 (q, *J* = 2.4 Hz), 136.4, 131.1, 129.9, 128.9, 125.8 (q, *J* = 274.4 Hz), 121.8, 121.4, 115.2, 105.9, 21.8, 19.7 (q, *J* = 36.2 Hz), 10.8 (q, *J* = 1.7 Hz) ppm.

**$^{19}$F NMR** (288 MHz, $CDCl_3$) δ = − 69.3 ppm.

**HRMS-ESI (m/z)** calculated for $C_{18}H_{15}F_3N_2O_2S$ $[M+H]^+$: 381.0879; found: 381.0886.

**IR** (ATR, neat) $\tilde{\nu}$ = 3021, 2360, 1596, 1522, 1493, 1428, 1402, 1342, 1319, 1303, 1263, 1201, 1150, 1126, 1091, 1051, 1014, 962, 928, 908, 812, 798, 747, 725, 703, 681, 652, 623, 595, 571, 533, 482, 435, 418 $cm^{-1}$.

Synthesis of **6o**:

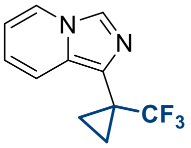


Prepared following general procedure C from imidazo[1,5-*a*]pyridine **5o** (70.9 mg, 0.60 mmol, 3.0 equiv.), $K_3PO_4$ (127.4 mg, 0.60 mmol, 3.0 equiv.) and **1** (88.5 mg, 0.20 mmol, 1.0 equiv.). Compound **6o** was purified by chromatography on silica gel eluting with pentane/EtOAc (100/0→70/30 (v/v)) and obtained as a yellow oil (19.1 mg, 0.08 mmol, 42%).

**$^1$H NMR** (300 MHz, $CDCl_3$): δ = 8.10 (d, *J* = 7.0 Hz, 1H), 7.51−7.41 (m, 2H), 6.79 (ddd, *J* = 9.1, 6.4, 1.0 Hz, 1H), 6.65 (ddd, *J* = 7.5, 6.4, 1.2 Hz, 1H), 1.64−1.57 (m, 2H), 1.35−1.27 (m, 2H) ppm.

**$^{13}$C{$^1$H} NMR** (101 MHz, $CDCl_3$) δ = 132.1, 131.8, 126.1 (q, *J* = 274.7 Hz)), 121.5, 120.0, 119.4, 118.7, 113.2, 21.0 (q, *J* = 35.6 Hz), 10.3 (q, *J* = 1.8 Hz) ppm.

**$^{19}$F NMR** (288 MHz, $CDCl_3$) δ = − 68.6 ppm.

**HRMS-ESI (m/z)** calculated for $C_{11}H_9F_3N_2$ $[M+H]^+$: 227.0791; found: 227.0791.

**IR** (ATR, neat) $\tilde{\nu}$ = 2925, 1761, 1690, 1635, 1592, 1491, 1432, 1376, 1346, 1312, 1268, 1233, 1155, 1116, 1031, 997, 931, 905, 826, 794, 762, 735, 701, 687, 637, 620, 572, 504, 438, 421, 403 $cm^{-1}$.

Synthesis of **6p**:

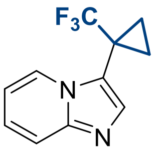


Prepared following general procedure C from imidazo[1,2-*a*]pyridine **5p** (70.8 mg, 0.60 mmol, 3.0 equiv.), $K_3PO_4$ (127.4 mg, 0.60 mmol, 3.0 equiv.) and **1** (88.6 mg, 0.20 mmol, 1.0 equiv.). Compound **6p** was purified by chromatography on silica gel eluting with pentane/EtOAc (100/0→20/1 (v/v)) and obtained as a yellow oil (13.9 mg, 0.06 mmol, 31%).

**$^1$H NMR** (400 MHz, $CDCl_3$): δ = 8.26 (dq, *J* = 6.9, 1.0 Hz, 1H), 7.67 (s, 1H), 7.64 (dt, *J* = 9.1, 1.2 Hz, 1H), 7.28−7.19 (m, 1H), 6.89 (td, *J* = 6.8, 1.2 Hz, 1H), 1.62−1.53 (m, 2H), 1.19−1.10 (m, 2H) ppm.

**$^{13}$C{$^1$H} NMR** (101 MHz, $CDCl_3$) δ = 146.4, 136.4, 126.1 (d, *J* = 274.5 Hz) 124.97, 124.17, 119.6, 118.3, 112.8, 18.1 (q, *J* = 36.2 Hz), 10.2 (q, *J* = 1.9 Hz) ppm.

**$^{19}$F NMR** (377 MHz, $CDCl_3$) δ = − 69.5 ppm.

**HRMS-ESI (m/z)** calculated for $C_{11}H_9F_3N_2$ $[M+H]^+$: 227.0791; found: 227.0793.

**IR** (ATR, neat) $\tilde{\nu}$ = 3089, 1722, 1636, 1498, 1428, 1369, 1343, 1321, 1300, 1269, 1220, 1154, 1130, 1036, 963, 929, 895, 869, 822, 756, 738, 696, 662, 626, 580, 516, 478, 444, 426 $cm^{-1}$.

Synthesis of **6q**:

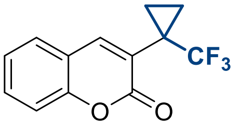


Prepared following general procedure C from coumarin **5q** (58.4 mg, 0.40 mmol, 2.0 equiv.), $K_3PO_4$ (127.5 mg, 0.60 mmol, 3.0 equiv.) and **1** (88.5 mg, 0.20 mmol, 1.0 equiv.). Compound **6q** was purified by chromatography on silica gel eluting with pentane/EtOAc (100/0→20/1 (v/v)) and obtained as a white solid (26.7 mg, 0.10 mmol, 50%).

**$^1$H NMR** (300 MHz, $CDCl_3$): δ = 7.88 (s, 1H), 7.60−7.48 (m, 2H), 7.38−7.26 (m, 2H), 1.51−1.44 (m, 2H), 1.10−1.06 (m, 2H) ppm.

**$^{13}$C{$^1$H} NMR** (101 MHz, $CDCl_3$) δ = 160.3, 154.1, 147.1, 132.5, 128.2, 125.9 (q, *J* = 275.3 Hz), 124.7, 124.6, 118.8, 116.8, 24.5 (q, *J* = 35.0 Hz), 10.7 (q, *J* = 2.2 Hz) ppm.

**$^{19}$F NMR** (288 MHz, $CDCl_3$) δ = − 70.2 ppm.

**HRMS-ESI (m/z)** calculated for $C_{13}H_9F_3O_2$ $[M+Na]^+$: 277.0447; found: 277.0450.

**IR** (ATR, neat) $\tilde{\nu}$ = 3101, 1718, 1633, 1608, 1577, 1492, 1458, 1431, 1361, 1338, 1274, 1251, 1225, 1166, 1148, 1114, 1081, 1050, 1022, 970, 955, 936, 927, 858, 794, 782, 756, 735, 680, 635, 599, 577, 566, 557, 548, 503, 472, 459 $cm^{-1}$.

**Melting point:** 136 °C.

Synthesis of **6r**:

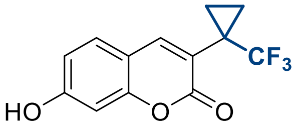


Prepared following general procedure C from umbelliferone **5r** (64.9 mg, 0.40 mmol, 2.0 equiv.), $K_3PO_4$ (127.4 mg, 0.60 mmol, 3.0 equiv.) and **1** (88.6 mg, 0.20 mmol, 1.0 equiv.). Compound **6r** was purified by chromatography on silica gel eluting with pentane/EtOAc (100/0→80/20 (v/v)) and obtained as a white solid (22.9 mg, 0.08 mmol, 42%).

**$^{1}$H NMR** (400 MHz, $CD_3CN$): δ = 7.96 (s, 1H), 7.46 (d, *J* = 8.5 Hz, 1H), 6.81 (dd, *J* = 8.5, 2.3 Hz, 1H), 6.75 (d, *J* = 2.3 Hz, 1H), 1.40−1.34 (m, 2H), 1.10−1.04 (m, 2H) ppm.

**$^{13}$C{$^{1}$H} NMR** (101 MHz, $CD_3CN$) δ = 162.1, 161.4, 156.8, 148.4, 130.8, 127.5 (q, *J* = 273.5 Hz), 119.7, 114.1, 113.1, 103.2, 24.8 (q, *J* = 34.7 Hz), 10.9 (q, *J* = 2.3 Hz) ppm.

**$^{19}$F NMR** (377 MHz, $CD_3CN$) δ = − 70.7 ppm.

**HRMS-ESI (m/z)** calculated for $C_{13}H_9F_3O_3$ $[M+H]^+$: 271.0577; found: 271.0564.

**IR** (ATR, neat) $\tilde{\nu}$ = 3166, 3132, 3026, 2360, 1687, 1593, 1456, 1359, 1267, 1227, 1177, 1149, 1119, 1076, 1026, 954, 851, 777, 730, 670, 639, 563, 516, 470, 458, 443 $cm^{-1}$.

**Melting point:** 214 – 215 °C.

Synthesis of **6s**:

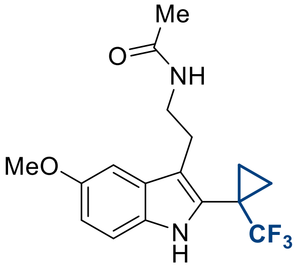


Prepared following general procedure C from coumarin 1 **5s** (136.1 mg, 0.40 mmol, 2.0 equiv.), $K_3PO_4$ (127.4 mg, 0.60 mmol, 3.0 equiv.) and **1** (88.6 mg, 0.20 mmol, 1.0 equiv.). Compound **6s** was purified by chromatography on silica gel eluting with pentane/EtOAc (100/0→15/85 (v/v)) and obtained as an off-white solid (16.1 mg, 0.05 mmol, 24%).

**$^{1}$H NMR** (300 MHz, $CDCl_3$): δ = 8.17 (s, 1H), 7.22 (d, *J* = 8.8 Hz, 1H), 7.09 (d, *J* = 2.4 Hz, 1H), 6.89 (dd, *J* = 8.8, 2.4 Hz, 1H), 5.66 (t, *J* = 4.5 Hz, 1H), 3.85 (s, 3H), 3.60 (q, *J* = 6.4 Hz, 2H), 3.02 (t, *J* = 7.1 Hz, 2H), 1.94 (s, 3H), 1.48−1.43 (m, 2H), 1.19−1.14 (m, 2H) ppm.

**$^{13}$C{$^{1}$H} NMR** (101 MHz, $CDCl_3$) δ = 170.3, 154.4, 130.9, 129.5, 128.2, 126.1 (q, *J* = 273.8 Hz), 114.1, 113.7, 111.9, 101.1, 56.1, 39.5, 24.9, 23.5, 20.9 (q, *J* = 35.2 Hz), 10.2 (q, *J* = 1.8 Hz) ppm.

**$^{19}$F NMR** (288 MHz, $CDCl_3$) δ = − 69.2 ppm.

**HRMS-ESI (m/z)** calculated for $C_{17}H_{19}F_3N_2O_2$ $[M+H]^+$: 341.1471; found: 341.1481.

**IR** (ATR, neat) $\tilde{\nu}$ = 3853, 3838, 3733, 3709, 3675, 3648, 3627, 3598, 3566, 3273, 2938, 2360, 2341, 1732, 1716, 1698, 1651, 1591, 1540, 1487, 1455, 1433, 1352, 1298, 1246, 1214, 1174, 1152, 1081, 1029, 944, 928, 828, 801, 751, 668, 525, 479, 456, 417, 404 $cm^{-1}$.

**Melting point:** 66-68 °C.

Synthesis of **6t**:

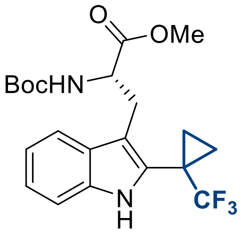


Prepared following general procedure C from coumarin 1 **5t** (127.4 mg, 0.40 mmol, 2.0 equiv.), $K_3PO_4$ (127.4 mg, 0.60 mmol, 3.0 equiv.) and **1** (88.6 mg, 0.20 mmol, 1.0 equiv.). Compound **6t** was purified by chromatography on silica gel eluting with pentane/EtOAc (100/0→70/30 (v/v)) and obtained as an off-white solid (28.4 mg, 0.08 mmol, 40%).

**$^1$H NMR** (300 MHz, $CDCl_3$): δ = 8.19 (s, 1H), 7.61 (d, *J* = 7.9 Hz, 1H), 7.31 (d, *J* = 8.1 Hz, 1H), 7.24−7.17 (m, 1H), 7.16−7.06 (m, 1H), 5.08 (d, *J* = 7.9 Hz, 1H), 4.69 (q, *J* = 7.7 Hz, 1H), 3.63 (s, 3H), 3.32 (dd, *J* = 14.5, 6.4 Hz, 1H), 3.23 (dd, *J* = 14.6, 7.9 Hz, 1H), 1.52−1.40 (m, 2H), 1.35 (s, 9H), 1.25−1.10 (m, 2H) ppm.

**$^{13}$C{$^1$H} NMR** (101 MHz, $CDCl_3$) δ = 173.4, 155.2, 135.6, 129.3, 127.9, 126.0 (q, *J* = 273.9 Hz), 123.2, 120.1, 119.7, 112.3, 110.9, 79.9, 53.8, 52.4, 28.4, 28.3, 21.0 (q, *J* = 35.3 Hz), 10.1 (d, *J* = 25.5 Hz) ppm.

**$^{19}$F NMR** (377 MHz, $CDCl_3$) δ = − 69.1 ppm.

**HRMS-ESI (m/z)** calculated for $C_{21}H_{25}F_3N_2O_4$ $[M+Na]^+$: 449.1659; found: 449.1656.

**IR** (ATR, neat) $\tilde{\nu}$ = 3278, 2983, 2358, 1735, 1683, 1526, 1457, 1438, 1363, 1319, 1273, 1224, 1137, 1090, 1060, 1015, 943, 853, 746, 698, 539, 498, 481, 470, 458, 426, 409 $cm^{-1}$.

**Melting point:** 155 – 156 °C.

$[\alpha]_{25}^{D}$: − 25° (c = 0.1 in acetone).

Synthesis of **6u**:

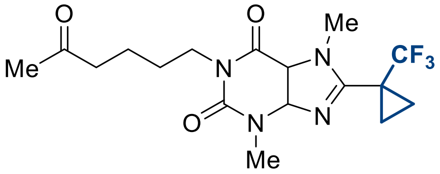


Prepared following general procedure C from pentoxifylline **5u** (167.1 mg, 0.60 mmol, 3.0 equiv.), $K_3PO_4$ (127.4 mg, 0.60 mmol, 3.0 equiv.) and **1** (88.4 mg, 0.20 mmol, 1.0 equiv.). Compound **6u** was purified by chromatography on silica gel eluting with pentane/EtOAc (100/0→50/50 (v/v)) and obtained as a yellow oil (22.1 mg, 0.06 mmol, 29%).

**$^1$H NMR** (400 MHz, $CDCl_3$): δ = 4.03 (s, 3H), 3.99 (t, *J* = 6.9 Hz, 2H), 3.54 (s, 3H), 2.48 (t, *J* = 7.0 Hz, 3H), 2.13 (s, 3H), 1.66−1.57 (m, 4H), 1.61−1.56 (m, 2H), 1.32−1.27 (m, 2H) ppm.

**$^{13}$C{$^1$H} NMR** (101 MHz, $CDCl_3$) δ = 208.8, 155.3, 151.5, 147.6, 146.7, 125.2 (q, *J* = 274.3 Hz)., 108.8, 43.3, 40.9, 33.0 (q, *J* = 2.0 Hz), 30.1, 29.9, 27.5, 21.3 (d, *J* = 36.0 Hz), 21.1, 10.6 (q, *J* = 1.6 Hz) ppm.

**$^{19}$F NMR** (377 MHz, $CDCl_3$) δ = − 67.9 ppm.

**HRMS-ESI (m/z)** calculated for $C_{17}H_{21}F_3N_4O_3$ $[M+H]^+$: 387.1639; found: 387.1650.

**IR** (ATR, neat) $\tilde{\nu}$ = 2950, 2360, 2341, 1710, 1654, 1607, 1548, 1503, 1442, 1393, 1349, 1289, 1249, 1221, 1136, 1101, 1051, 968, 938, 844, 765, 748, 710, 670, 605, 585, 552, 485, 471, 447, 431, 414 $cm^{-1}$.

Synthesis of **6v**:

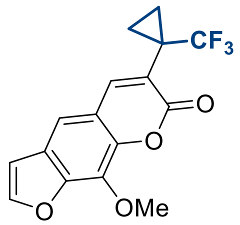


Prepared following general procedure C from xanthotoxin **5v** (129.8 mg, 0.60 mmol, 3.0 equiv.), $K_3PO_4$ (127.4 mg, 0.60 mmol, 3.0 equiv.) and **1** (88.5 mg, 0.20 mmol, 1.0 equiv.). The desired compound was purified by chromatography on silica gel eluting with pentane/EtOAc (100/0→90/10 (v/v)) and obtained as a colorless solid (20.6 mg, 0.06 mmol, 32%).

**$^1$H NMR** (300 MHz, $CDCl_3$): δ = 7.93 (s, 1H), 7.69 (d, *J* = 2.2 Hz, 1H), 7.35 (s, 1H), 6.82 (d, *J* = 2.2 Hz, 1H), 4.29 (s, 3H), 1.56−1.42 (m, 2H), 1.15−1.04 (m, 2H) ppm.

**$^{13}$C{$^1$H} NMR** (101 MHz, $CDCl_3$) δ = 159.9, 148.0, 147.9, 146.9, 142.9, 132.8, 126.4, 126.0 (q, *J* = 274.3 Hz), 121.6, 116.5, 113.2, 106.9, 61.5, 24.4 (q, *J* = 35.0 Hz), 10.7 (q, *J* = 2.0 Hz) ppm.

**$^{19}$F NMR** (288 MHz, $CDCl_3$) δ = − 70.2 ppm.

**HRMS-ESI (m/z)** calculated for $C_{16}H_{11}F_3O_4$ $[M+H]^+$: 325.0682; found: 325.0679.

**IR** (ATR, neat) $\tilde{\nu}$ = 2958, 1714, 1630, 1591, 1472, 1432, 1392, 1360, 1337, 1281, 1200, 1176, 1121, 1095, 1045, 1022, 957, 909, 873, 844, 809, 761, 737, 693, 682, 639, 594, 560, 523, 485, 468, 446, 425 $cm^{-1}$.

**Melting point:** 156 – 158 °C.

Synthesis of **6v'**:

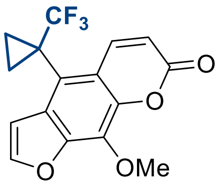


Obtained as a minor regioisomer in the synthesis of **6v**. Compound **6v'** was purified by chromatography on silica gel eluting with pentane/EtOAc (100/0→90/10 (v/v)) and obtained as a white solid (16.2 mg, 0.05 mmol, 25%).

**$^1$H NMR** (300 MHz, $CDCl_3$): δ = 8.28 (d, *J* = 10.0 Hz, 1H), 7.72 (d, *J* = 2.2 Hz, 2H), 6.98 (d, *J* = 2.2 Hz, 3H), 6.45 (d, *J* = 10.0 Hz, 2H), 4.30 (s, 3H), 1.79−1.69 (m, 1H), 1.68−1.57 (m, 1H), 1.20−1.08 (m, 2H) ppm.

**$^{13}$C{$^1$H} NMR** (101 MHz, $CDCl_3$) δ = 159.9, 146.9, 146.7, 143.5, 141.3, 133.5, 129.0, 126.4 (q, *J* = 274.6 Hz), 117.7, 117.3, 115.4, 106.2, 61.5, 22.9 (d, *J* = 34.7 Hz), 12.4 (d, *J* = 2.3 Hz), 10.5 (d, *J* = 2.5 Hz) ppm.

**$^{19}$F NMR** (288 MHz, $CDCl_3$) δ = − 69.4 ppm.

**HRMS-ESI (m/z)** calculated for $C_{16}H_{11}F_3O_4$ $[M+H]^+$: 325.0682; found: 325.0674.

**IR** (ATR, neat) $\tilde{\nu}$ = 2951, 1715, 1622, 1589, 1481, 1454, 1421, 1373, 1347, 1315, 1278, 1247, 1209, 1186, 1137, 1121, 1105, 1051, 1037, 1018, 943, 919, 877, 835, 808, 779, 754, 725, 688, 643, 617, 591, 552, 517, 502, 487, 473, 457, 418 $cm^{-1}$.

**Melting point:** 154 – 155 °C.

Synthesis of **6v''**:

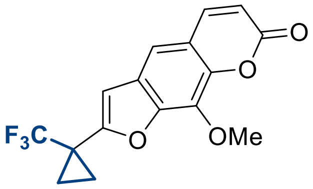


Obtained third regiosomer in the synthesis of **6v**. Compound **6v''** was purified after a second column chromatography on silica gel eluting with pentane/DCM (100/0→30/70 (v/v)) and obtained as a colorless solid (19.4 mg, 0.06 mmol, 30%).

**$^1$H NMR** (300 MHz, $CDCl_3$): δ = 7.74 (d, *J* = 9.6 Hz, 1H), 7.27 (s, 1H), 6.77 (s, 1H), 6.37 (d, *J* = 9.6 Hz, 1H), 4.27 (s, 3H), 1.55−1.50 (m, 2H), 1.48−1.40 (m, 2H) ppm.

**$^{13}$C{$^1$H} NMR** (101 MHz, $CDCl_3$) δ = 160.5, 154.6, 147.2, 144.4, 143.3, 132.6, 126.9, 125.3 (q, *J* = 273.8 Hz), 116.7, 115.1, 112.6, 105.7, 61.4, 23.0 (q, *J* = 35.7 Hz), 11.5 (q, *J* = 2.0 Hz) ppm.

**$^{19}$F NMR** (288 MHz, $CDCl_3$) δ = − 69.2 ppm.

**HRMS-ESI (m/z)** calculated for $C_{16}H_{11}F_3O_4$ $[M+H]^+$: 325.0682; found: 325.0680.

**IR** (ATR, neat) $\tilde{\nu}$ = 3051, 2952, 2924, 2118, 1997, 1717, 1624, 1605, 1585, 1470, 1455, 1433, 1399, 1317, 1268, 1244, 1221, 1200, 1170, 1132, 1109, 1082, 1071, 1010, 969, 919, 890, 827, 807, 791, 753, 730, 706, 690, 671, 633, 611, 590, 568, 555, 512, 498, 465, 438, 422 $cm^{-1}$.

**Melting point:** 119 – 120 °C.

Synthesis of **6w**:

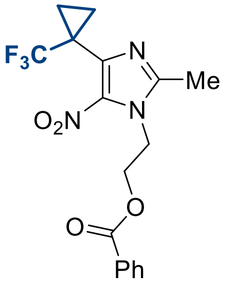


Prepared following general procedure C from metronidazole **5w** (165.2 mg, 0.60 mmol, 3.0 equiv.), $K_3PO_4$ (127.4 mg, 0.60 mmol, 3.0 equiv.) and **1** (88.5 mg, 0.20 mmol, 1.0 equiv.). Compound **6w** was purified by chromatography on silica gel eluting with pentane/EtOAc (100/0→60/40 (v/v)) and obtained as a yellow oil (31.9 mg, 0.09 mmol, 42%).

**$^1$H NMR** (400 MHz, $CDCl_3$): δ = 7.95−7.88 (m, 2H), 7.61−7.56 (m, 1H), 7.47−7.41 (m, 2H), 4.75−4.64 (m, 4H), 2.47 (s, 3H), 1.58−1.48 (m, 2H), 1.20−1.15 (m, 2H) ppm.

**$^{13}$C{$^1$H} NMR** (101 MHz, $CDCl_3$) δ = 166.1, 148.4, 139.3, 133.8, 133.5, 129.7, 129.1, 128.8, 125.7 (q, *J* = 274.3 Hz), 62.8, 45.9, 22.8 (q, *J* = 35.3 Hz), 14.3, 11.4 (q, *J* = 2.2 Hz) ppm.

**$^{19}$F NMR** (377 MHz, $CDCl_3$) δ = − 69.1 ppm.

**HRMS-ESI (m/z)** calculated for $C_{17}H_{16}F_3N_3O_4$ $[M+H]^+$: 384.1166; found: 384.1167.

**IR** (ATR, neat) $\tilde{\nu}$ = 2922, 2853, 1720, 1601, 1562, 1501, 1463, 1420, 1355, 1334, 1266, 1155, 1130, 1095, 1070, 1027, 948, 879, 820, 753, 710, 667, 481, 470, 436, 428, 412 $cm^{-1}$.

# PHOTOREDOX CATALYTIC TRANSFER TO (HETERO)AROMATIC COMPOUNDS

General procedure **D** for the synthesis of (**6x**-**6ai**):

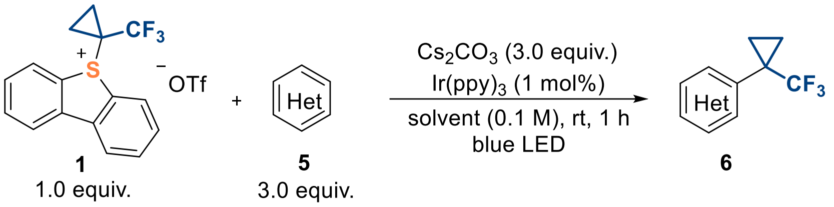


A Schlenk flask equipped with a magnetic stir bar was charged with Ir(ppy)$_3$ (1.3 mg, 0.2 µmol, 1 mol%), sulfonium salt **1** (0.2 mmol, 1.0 equiv.), the desired heteroaromatic compound (0.6 mmol, 3.0 equiv.) and $Cs_2CO_3$ (0.6 mmol, 3.0 equiv.). Subsequently, acetonitrile was added (2 mL), the flask was sealed, transferred to a photoreactor equipped with blue LED strips (maximum wavelength: 462 nm) and irradiated at 50% intensity for 1 h. Finally, the reaction mixture was diluted with DCM (3 mL), and purified by silica gel column chromatography using the indicated solvent mixtures.

Synthesis of **6x**:

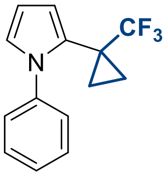


Prepared following general procedure D from 1-phenyl-1*H*-pyrrole **5x** (86.1 mg, 0.60 mmol, 3.0 equiv.), $Cs_2CO_3$ (195.5 mg, 0.60 mmol, 3.0 equiv.) and **1** (88.6 mg, 0.20 mmol, 1.0 equiv.). Compound **6x** was purified by chromatography on silica gel eluting with pentane/EtOAc (100/0→100/1 (v/v)). Analytically pure **6x** was obtained after preparative HPLC as a colorless oil (38.7 mg, 0.13 mmol, 65%).

**$^1$H NMR** (400 MHz, $CDCl_3$): δ = 7.50−7.34 (m, 5H), 6.76 (dd, *J* = 2.9, 1.9 Hz, 1H), 6.44 (dd, *J* = 3.6, 1.9 Hz, 1H), 6.24 (dd, *J* = 3.6, 2.9 Hz, 1H), 1.20−1.13 (m, 2H), 0.88−0.82 (m, 2H) ppm.

**$^{13}$C{$^1$H} NMR** (101 MHz, $CDCl_3$) δ = 140.7, 129.0, 127.8, 127.8, 126.1 (q, *J* = 273.9 Hz), 127.0, 126.9, 124.2, 114.1, 108.3, 20.6 (q, *J* = 34.9 Hz), 11.8 (q, *J* = 2.2 Hz) ppm.

**$^{19}$F NMR** (377 MHz, $CDCl_3$) δ = − 68.8 ppm.

**HRMS-ESI (m/z)** calculated for $C_{14}H_{12}F_3N$ $[M+H]^+$: 252.0995; found: 252.0997.

**IR** (ATR, neat) $\tilde{\nu}$ = 1641, 1599, 1499, 1467, 1424, 1358, 1333, 1208, 1150, 1132, 1103, 1086, 1074, 1036, 1003, 980, 916, 884, 820, 797, 764, 718, 694, 679, 660, 626, 606, 553, 497, 447, 406 cm$^{-1}$.

Synthesis of **6y**:

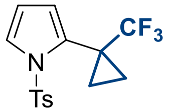


Prepared following general procedure D from 1-phenyl-1*H*-pyrrole **5y** (132.8 mg, 0.60 mmol, 3.0 equiv.), $Cs_2CO_3$ (195.5 mg, 0.60 mmol, 3.0 equiv.) and **1** (88.6 mg, 0.20 mmol, 1.0 equiv.). Compound **6y** was purified by chromatography on silica gel eluting with pentane/EtOAc (100/0→100/1 (v/v)). Analytically pure **6y** was obtained after preparative HPLC as a colorless oil (19.8 mg, 0.06 mmol, 30%).

**$^1$H NMR** (400 MHz, $CDCl_3$): δ = 7.66−7.60 (m, 2H), 7.31−7.27 (m, 3H), 6.41 (dd, *J* = 3.4, 1.8 Hz, 1H), 6.25 (t, *J* = 3.4 Hz, 1H), 2.41 (s, 3H), 1.46−1.40 (m, 2H), 1.12−1.05 (m, 2H) ppm.

**$^{13}$C{$^1$H} NMR** (101 MHz, $CDCl_3$) δ = 144.9, 137.2, 130.7, 130.0, 125.5 (q, *J* = 274.5 Hz), 125.5, 124.2, 119.8, 111.4, 21.8, 21.1 (q, *J* = 35.6 Hz), 12.9 (q, *J* = 2.2 Hz) ppm.

**$^{19}$F NMR** (377 MHz, $CDCl_3$) δ = − 68.6 ppm.

**HRMS-ESI (m/z)** calculated for $C_{15}H_{14}F_3NO_2S$ $[M+H]^+$: 330.0770; found: 330.0775.

**IR** (ATR, neat) $\tilde{\nu}$ = 2926, 2360, 1596, 1482, 1370, 1349, 1322, 1173, 1143, 1081, 1055, 943, 923, 872, 812, 730, 703, 689, 668, 612, 590, 543, 473, 432, 404 $cm^{-1}$.

Synthesis of **6z**:

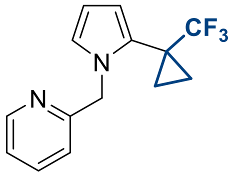


Prepared following general procedure D from 2-((1*H*-pyrrol-1-yl)methyl)pyridine **5z** (94.9 mg, 0.60 mmol, 3.0 equiv.), $Cs_2CO_3$ (195.4 mg, 0.60 mmol, 3.0 equiv.) and **1** (88.5 mg, 0.20 mmol, 1.0 equiv.). Compound **6z** was purified by chromatography on silica gel eluting with pentane/EtOAc (100/0→70/30 (v/v)) and obtained as a yellow oil (33.7 mg, 0.13 mmol, 63%).

**$^1$H NMR** (400 MHz, $CDCl_3$): δ = 8.54 (s, 1H), 8.40 (s, 1H), 7.30−7.22 (m, 2H), 6.52 (dd, *J* = 2.8, 1.8 Hz, 1H), 6.28 (dd, *J* = 3.6, 1.8 Hz, 1H), 6.15 (dd, *J* = 3.6, 2.9 Hz, 1H), 5.24 (s, 2H), 1.37−1.31 (m, 2H), 0.98−0.93 (m, 2H) ppm.

**$^{13}$C{$^1$H} NMR** (101 MHz, $CDCl_3$) δ = 149.3, 148.6, 134.6, 133.9, 127.7, 126.1 (d, *J* = 273.7 Hz), 123.8, 122.5, 113.0, 108.5, 48.1 (q, *J* = 1.8 Hz), 20.1 (q, *J* = 35.0 Hz), 10.6 (q, *J* = 2.0 Hz) ppm.

**$^{19}$F NMR** (377 MHz, $CDCl_3$) δ = − 69.4 ppm.

**HRMS-ESI (m/z)** calculated for $C_{14}H_{13}F_3N_2$ $[M+H]^+$: 267.1104; found: 267.1109.

**IR** (ATR, neat) $\tilde{\nu}$ = 1703, 1578, 1480, 1425, 1390, 1348, 1325, 1271, 1176, 1130, 1090, 1072, 1042, 1026, 928, 788, 710, 680, 657, 640, 619, 558, 438 $cm^{-1}$.

Synthesis of **6aa**:

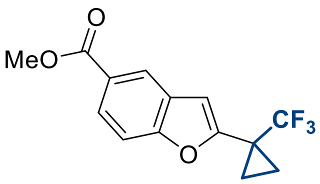


Prepared following general procedure D from methyl benzofuran-5-carboxylate **5aa** (105.8 mg, 0.60 mmol, 3.0 equiv.), $Cs_2CO_3$ (195.5 mg, 0.60 mmol, 3.0 equiv.) and **1** (88.5 mg, 0.20 mmol, 1.0 equiv.). Compound **6aa** was purified by chromatography on silica gel eluting with pentane/EtOAc (100/0→100/1 (v/v)). Analytically pure **6aa** was obtained after preparative HPLC as a colorless oil (28.1 mg, 0.1 mmol, 49%).

**$^1$H NMR** (400 MHz, $CDCl_3$): δ = 8.28−8.25 (m, 1H), 8.00 (dd, *J* = 8.7, 1.7 Hz, 1H), 7.44 (dt, *J* = 8.7, 0.8 Hz, 1H), 6.80 (s, 1H), 3.93 (s, 3H), 1.53−1.46 (m, 2H), 1.44−1.38 (m, 2H) ppm.

**$^{13}$C{$^1$H} NMR** (101 MHz, $CDCl_3$) δ = 167.3, 157.0, 154.1, 128.4, 126.3, 125.5, 125.4 (q, *J* = 273.3 Hz), 123.4, 111.1, 106.2, 52.3, 23.0 (q, *J* = 35.7 Hz), 11.3 (q, *J* = 2.0 Hz) ppm.

**$^{19}$F NMR** (377 MHz, $CDCl_3$) δ = − 69.2 ppm.

**HRMS-EI (m/z)** calculated for $C_{14}H_{11}F_3O_3$ $[M]^{\cdot}$: 284.0655; found: 284.0656.

**IR** (ATR, neat) $\tilde{\nu}$ = 3123, 2958, 2360, 2020, 1900, 1806, 1705, 1605, 1591, 1437, 1357, 1327, 1305, 1267, 1250, 1230, 1179, 1134, 1111, 1079, 969, 936, 901, 844, 820, 811, 797, 765, 738, 687, 671, 609, 589, 565, 522, 494, 480, 445, 430, 415 $cm^{-1}$.

Synthesis of **6ab**:

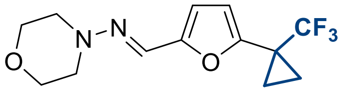


Prepared following general procedure D from 1-(furan-2-yl)-*N*-morpholinomethanimine **5ab** (108.2 mg, 0.60 mmol, 3.0 equiv.), $Cs_2CO_3$ (195.5 mg, 0.60 mmol, 3.0 equiv.) and **1** (88.5 mg, 0.20 mmol, 1.0 equiv.). Compound **6ab** was purified by chromatography on silica gel eluting with pentane/EtOAc (100/0→100/1 (v/v)). Analytically pure **6ab** was obtained after preparative HPLC as a yellowish oil (21.6 mg, 0.07 mmol, 37%).

**$^1$H NMR** (400 MHz, $CDCl_3$): δ = 7.40 (s, 1H), 6.47 (d, *J* = 3.4 Hz, 1H), 6.38 (d, *J* = 3.4 Hz, 1H), 3.90−3.83 (m, 4H), 3.16−3.09 (m, 4H), 1.39−1.34 (m, 2H), 1.27−1.23 (m, 2H) ppm.

**$^{13}$C{$^1$H} NMR** (101 MHz, $CDCl_3$) δ = 151.3, 150.0, 126.6, 125.6 (q, *J* = 273.6 Hz), 111.4, 109.1, 66.5, 51.7, 22.4 (q, *J* = 35.4 Hz), 10.9 (q, *J* = 2.0 Hz) ppm.

**$^{19}$F NMR** (377 MHz, $CDCl_3$) δ = − 69.5 ppm.

**HRMS-ESI (m/z)** calculated for $C_{13}H_{15}F_3N_2O_2$ $[M+H]^+$: 289.1158; found: 289.1158.

**IR** (ATR, neat) $\tilde{\nu}$ = 2964, 2856, 2360, 2109, 1732, 1583, 1542, 1524, 1452, 1355, 1273, 1171, 1133, 1116, 1095, 1002, 967, 930, 886, 863, 828, 789, 757, 709, 692, 675, 653, 632, 566, 529, 500, 470, 434, 407 $cm^{-1}$.

Synthesis of **6ac**:

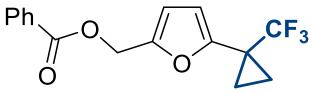


Prepared following general procedure D from furan-2-ylmethyl benzoate **5ac** (121.5 mg, 0.60 mmol, 3.0 equiv.), $Cs_2CO_3$ (195.4 mg, 0.60 mol, 3.0 equiv.) and **1** (88.5 mg, 0.20 mmol, 1.0 equiv.). Compound **6ac** was purified by chromatography on silica gel eluting with pentane/EtOAc (100/0→100/1 (v/v)). Analytically pure **6ac** was obtained after preparative HPLC as a colorless oil (37.9 mg, 0.12 mmol, 61%).

**$^1$H NMR** (400 MHz, $CDCl_3$): δ = 8.10−8.01 (m, 2H), 7.62−7.52 (m, 1H), 7.43 (dd, *J* = 8.3, 7.1 Hz, 2H), 6.44 (d, *J* = 3.3 Hz, 1H), 6.35 (d, *J* = 3.3 Hz, 1H), 5.26 (s, 2H), 1.39−1.34 (m, 2H), 1.28−1.21 (m, 2H) ppm.

**$^{13}$C{$^1$H} NMR** (101 MHz, $CDCl_3$) δ = 166.3, 150.6, 149.2, 133.3, 130.0, 129.9, 128.5, 125.6 (d, *J* = 273.3 Hz), 111.9, 110.4, 58.6, 22.4 (q, *J* = 35.4 Hz), 10.7 (q, *J* = 2.1 Hz) ppm.

**$^{19}$F NMR** (377 MHz, $CDCl_3$) δ = − 69.6 ppm.

**HRMS-ESI (m/z)** calculated for $C_{16}H_{13}F_3O_3$ $[M+Na]^+$: 333.0709; found: 333.0712.

**IR** (ATR, neat) $\tilde{\nu}$ = 1719, 1601, 1585, 1563, 1451, 1431, 1403, 1353, 1315, 1266, 1203, 1173, 1136, 1093, 1069, 1024, 982, 923, 876, 795, 749, 708, 688, 634, 565, 509, 479, 456, 434, 415, 406 $cm^{-1}$.

Synthesis of **6ad**:

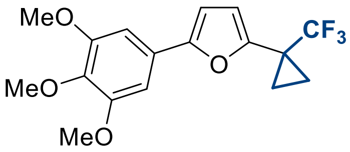


Prepared following general procedure D from 2-(3,4,5-trimethoxyphenyl)furan **5ad** (140.6 mg, 0.60 mmol, 3.0 equiv.), $Cs_2CO_3$ (195.5 mg, 0.60 mmol, 3.0 equiv.) and **1** (88.5 mg, 0.20 mmol, 1.0 equiv.). Compound **6ad** was purified by chromatography on silica gel eluting with pentane/EtOAc (100/0→40/1 (v/v)). Analytically pure **6ad** was obtained after preparative HPLC as a white solid (60.1 mg, 0.18 mmol, 88%).

**$^1$H NMR** (400 MHz, $CDCl_3$): δ = 6.85 (s, 2H), 6.51 (d, *J* = 3.4 Hz, 1H), 6.41 (d, *J* = 3.3 Hz, 1H), 3.92 (s, 6H), 3.86 (s, 3H), 1.45−1.38 (m, 2H), 1.28−1.23 (m, 2H) ppm.

**$^{13}$C{$^1$H} NMR** (101 MHz, $CDCl_3$) δ = 153.7, 153.5, 149.4, 138.1, 126.41, 125.7 (q, *J* = 273.4 Hz), 111.6, 105.7, 101.4, 61.1, 56.4, 22.4 (d, *J* = 35.5 Hz), 10.7 (q, *J* = 2.1 Hz) ppm.

**$^{19}$F NMR** (377 MHz, $CDCl_3$) δ = − 69.5 ppm.

**HRMS-ESI (m/z)** calculated for $C_{17}H_{17}F_3O_4$ $[M+H]^+$: 343.1152; found: 343.1160.

**IR** (ATR, neat) $\tilde{\nu}$ = 3127, 2938, 2827, 2359, 1717, 1582, 1548, 1498, 1470, 1455, 1419, 1384, 1341, 1307, 1248, 1238, 1219, 1176, 1157, 1122, 1097, 1076, 1049, 1029, 1008, 975, 932, 835, 819, 797, 789, 762, 734, 686, 658, 606, 564, 534, 510, 499, 454, 442, 416, 404 $cm^{-1}$.

**Melting point:** 61 °C.

Synthesis of **6ae**:

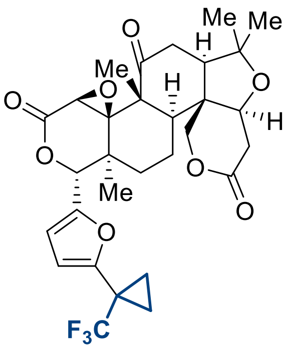


Prepared following general procedure D from limonin **5ae** (282.4 mg, 0.60 mmol, 3.0 equiv.), $Cs_2CO_3$ (195.5 mg, 0.60 mmol, 3.0 equiv.) and **1** (88.5 mg, 0.20 mmol, 1.0 equiv.). Compound **6ae** was purified by chromatography on silica gel eluting with pentane/EtOAc (100/0→70/30 (v/v)) and obtained as an off white solid (35.8 mg, 0.06 mmol, 31%).

**$^1$H NMR** (300 MHz, $CDCl_3$): δ = 7.30 (s, 1H), 6.30 (s, 1H), 5.40 (s, 1H), 4.76 (d, *J* = 13.1 Hz, 1H), 4.46 (d, *J* = 13.1 Hz, 1H), 4.03 (s, 2H), 2.97 (dd, *J* = 16.8, 3.8 Hz, 1H), 2.85 (t, *J* = 15.2 Hz, 1H), 2.68 (dd, *J* = 16.8, 1.9 Hz, 1H), 2.55 (dd, *J* = 12.2, 1.9 Hz, 1H), 2.46 (dd, *J* = 14.5, 3.2 Hz, 1H), 2.22 (dd, *J* = 15.8, 3.4 Hz, 1H), 1.85−1.71 (m, 2H), 1.61−1.44 (m, 2H), 1.37−1.32 (m, 2H), 1.29 (s, 3H), 1.17 (s, 8H), 1.06 (s, 3H).ppm.

**$^{13}C\{^1H\}$ NMR** (101 MHz, $CDCl_3$) δ = 206.3, 169.3, 166.7, 150.7, 140.7, 125.5 (q, *J* = 274.7 Hz), 121.1, 109.3, 80.5, 79.3, 77.8, 65.8, 65.5, 60.7, 53.9, 51.5, 48.2, 38.0, 36.5, 35.8, 30.9, 30.3, 22.3 (q, *J* = 35.6 Hz), 21.5, 20.9, 19.0, 17.7, 10.6 (q, *J* = 1.9 Hz) ppm.

**$^{19}F$ NMR** (288 MHz, $CDCl_3$) δ = − 69.7 ppm.

**HRMS-ESI (m/z)** calculated for $C_{30}H_{33}F_3O_8$ $[M+Na]^+$: 601.2020; found: 601.2016.

**IR** (ATR, neat) $\tilde{\nu}$ = 2971, 2358, 1744, 1696, 1548, 1393, 1359, 1261, 1132, 1115, 1078, 1053, 1031, 948, 915, 888, 863, 808, 763, 699, 659, 620, 596, 546, 506, 485, 437, 411 $cm^{-1}$.

**Melting point:** 279 °C.

$[\alpha]_{25}^{D}$: − 93° (c = 0.1 in acetone).

Synthesis of **6af**:

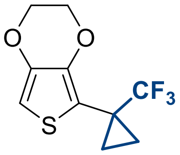


Prepared following general procedure D from 2,3-dihydrothieno[3,4-*b*][1,4]dioxine **5af** (85.3 mg, 0.60 mmol, 3.0 equiv.), $Cs_2CO_3$ (195.5 mg, 0.60 mmol, 3.0 equiv.) and **1** (88.6 mg, 0.20 mmol, 1.0 equiv.). Compound **6af** was purified by chromatography on silica gel eluting with pentane/EtOAc (100/0→100/1 (v/v)). Analytically pure **6af** was obtained after preparative HPLC as a colorless oil (33.7 mg, 0.13 mmol, 67%).

**$^1$H NMR** (400 MHz, $CDCl_3$): δ = 6.28 (s, 1H), 4.25−4.21 (m, 2H), 4.20−4.16 (m, 2H), 1.38−1.33 (m, 2H), 1.16−1.12 (m, 2H) ppm.

**$^{13}C\{^1H\}$ NMR** (101 MHz, $CDCl_3$) δ = 142.0, 141.1, 126.0 (q, *J* = 274.1 Hz), 111.5, 99.6, 64.8, 64.6, 20.1 (q, *J* = 36.0 Hz), 11.3 (q, *J* = 2.2 Hz) ppm.

**$^{19}F$ NMR** (377 MHz, $CDCl_3$) δ = − 70.4 ppm.

**HRMS-EI (m/z)** calculated for $C_{10}H_9F_3O_2S$ $[M]^{\cdot}$: 250.0270; found: 250.0268.

**IR** (ATR, neat) $\tilde{\nu}$ = 3663, 3514, 2986, 2932, 2879, 2360, 1903, 1691, 1594, 1547, 1504, 1435, 1358, 1314, 1246, 1189, 1155, 1129, 1079, 1069, 1055, 1006, 983, 950, 926, 900, 859, 835, 755, 725, 706, 690, 651, 575, 560, 532, 482, 455 cm$^{-1}$.

Synthesis of **6ag**:

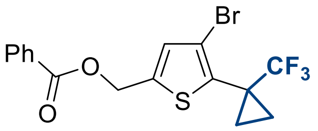


Prepared following general procedure D from (4-bromothiophen-2-yl)methyl benzoate **5ag** (178.3 mg, 0.60 mmol, 3.0 equiv.), $Cs_2CO_3$ (195.6 mg, 0.60 mmol, 3.0 equiv.) and **1** (88.5 mg, 0.20 mmol, 1.0 equiv.). Compound **6ag** was purified by chromatography on silica gel eluting with pentane/EtOAc (100/0→50/1 (v/v)). Analytically pure **6ag** was obtained after preparative HPLC as a colorless oil (33.4 mg, 0.08 mmol, 41%).

**$^1$H NMR** (400 MHz, $CDCl_3$): δ = 8.10−8.03 (m, 2H), 7.62−7.54 (m, 1H), 7.50−7.42 (m, 2H), 5.40 (d, *J* = 0.8 Hz, 2H), 1.55−1.48 (m, 2H), 1.26−1.19 (m, 2H) ppm.

**$^{13}$C{$^1$H} NMR** (101 MHz, $CDCl_3$) δ = 166.3, 139.6, 134.1, 133.5, 131.0, 130.0, 129.6, 128.62, 125.5 (q, *J* = 274.7 Hz)., 114.0, 60.7, 22.4 (q, *J* = 36.0 Hz), 12.4 (q, *J* = 2.0 Hz) ppm.

**$^{19}$F NMR** (377 MHz, $CDCl_3$) δ = − 69.7 ppm.

**HRMS-ESI (m/z)** calculated for $C_{16}H_{12}BrF_3O_2S$ $[M+Na]^+$: 426.9586; found: 426.9596.

**IR** (ATR, neat) $\tilde{\nu}$ = 2956, 2360, 1718, 1601, 1584, 1545, 1491, 1452, 1350, 1315, 1264, 1138, 1092, 1068, 1049, 1025, 938, 908, 833, 754, 732, 709, 686, 669, 628, 555, 497, 455, 431, 405 cm$^{-1}$.

Synthesis of **6ah**:

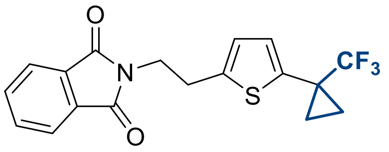


Prepared following general procedure D from 2-(2-(thiophen-2-yl)ethyl)isoindoline-1,3-dione **5ah** (154.4 mg, 0.60 mmol, 3.0 equiv.), $Cs_2CO_3$ (195.5 mg, 0.60 mmol, 3.0 equiv.) and **1** (88.5 mg, 0.20 mmol, 1.0 equiv.). Compound was purified by chromatography on silica gel eluting with pentane/EtOAc (100/0→40/1 (v/v)). Analytically pure **6ah** was obtained after preparative HPLC as colorless oil (13.4 mg, 0.04 mmol, 18%).

**$^1$H NMR** (400 MHz, $CDCl_3$): δ = 7.89−7.80 (m, 2H), 7.76−7.67 (m, 2H), 6.91 (d, *J* = 3.5 Hz, 1H), 6.73 (d, *J* = 3.5 Hz, 1H), 3.99−3.90 (m, 2H), 3.19−3.12 (m, 2H), 1.40−1.33 (m, 2H), 1.13−1.06 (m, 2H) ppm.

**$^{13}$C{$^1$H} NMR** (101 MHz, $CDCl_3$) δ = 168.2, 140.9, 137.9, 134.2, 132.2, 129.0, 125.8 (q, *J* = 273.7 Hz), 125.3, 123.4, 39.2, 28.9, 23.3 (q, *J* = 34.7 Hz), 12.0 (q, *J* = 2.3 Hz) ppm.

**$^{19}$F NMR** (377 MHz, $CDCl_3$) δ = − 70.4 ppm.

**HRMS-ESI (m/z)** calculated for $C_{18}H_{14}F_3NO_2S$ $[M+H]^+$: 366.0770; found: 366.0769.

**IR** (ATR, neat) $\tilde{\nu}$ = 3471, 2939, 2359, 1772, 1709, 1613, 1483, 1467, 1432, 1394, 1360, 1306, 1133, 1085, 1066, 987, 925, 868, 806, 716, 691, 636, 594, 530, 456, 429, 412 cm$^{-1}$.

Synthesis of **6ai**:

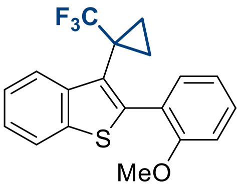

Prepared following general procedure D from 2-(2-methoxyphenyl)benzo[*b*]thiophene **5ai** (144.2 mg, 0.60 mmol, 3.0 equiv.), $Cs_2CO_3$ (195.4 mg, 0.60 mmol, 3.0 equiv.) and **1** (88.5 mg, 0.20 mmol, 1.0 equiv.). Compound **6ai** was purified by chromatography on silica gel eluting with pentane/EtOAc (100/0→50/1 (v/v)). Analytically pure **6ai** was obtained after preparative HPLC as a colorless oil (38.2 mg, 0.11 mmol, 55%).

**$^1$H NMR** (400 MHz, $CDCl_3$): δ = 8.01 (dq, *J* = 8.1, 1.0 Hz, 1H), 7.81 (dt, *J* = 7.9, 1.0 Hz, 1H), 7.50−7.40 (m, 3H), 7.36 (ddd, *J* = 8.2, 7.1, 1.3 Hz, 1H), 7.05 (td, *J* = 7.6, 1.1 Hz, 1H), 6.98 (d, *J* = 7.9 Hz, 1H), 3.77 (s, 3H), 1.49−1.02 (m, 2H), 1.03−0.77 (m, 2H) ppm.

**$^{13}$C{$^1$H} NMR** (101 MHz, $CDCl_3$) δ = 157.2, 142.9, 140.3, 139.4, 133.0 (q, *J* = 2.1 Hz), 130.6, 126.9, 126.5 (q, *J* = 274.5 Hz), 124.4, 124.2, 123.4 (q, *J* = 1.8 Hz), 122.5, 122.1, 120.4, 110.8, 55.6, 21.1 (q, *J* = 34.9 Hz), 10.0 (brs) ppm.

**$^{19}$F NMR** (282 MHz, $CDCl_3$) δ = − 67.6 ppm.

**HRMS-ESI (m/z)** calculated for $C_{19}H_{15}F_3O_1S$ $[M+H]^+$: 349.0868; found: 349.0864.

**IR** (ATR, neat) $\tilde{\nu}$ = 3060, 2939, 2835, 2360, 1905, 1722, 1601, 1580, 1545, 1485, 1459, 1433, 1375, 1343, 1275, 1247, 1207, 1176, 1151, 1125, 1074, 1049, 1023, 968, 953, 937, 907, 840, 796, 781, 752, 732, 679, 667, 635, 615, 561, 493, 481, 431, 412 $cm^{-1}$.

## TRANSFER OF $D_4$-LABELLED TFCp

Synthesis of **4u-d$_4$**:

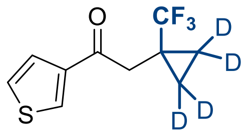


Prepared following general procedure B from *tert*-butyldimethyl((1-(thiophen-2-yl)vinyl)oxy)silane **3u** (48.1 mg, 0.20 mmol, 1.0 equiv.) and **1-d$_4$** (116.1 mg, 0.26 mmol, 1.3 equiv.). Compound **4u-d$_4$** was purified by chromatography on silica gel eluting with pentane/EtOAc (100/0→95/5 (v/v)) and obtained as a colorless oil (40.3 mg, 0.18 mmol, 90%).

**$^1$H NMR** (300 MHz, $CDCl_3$): δ = 8.06 (dd, *J* = 2.9, 1.3 Hz, 1H), 7.53 (dd, *J* = 5.1, 1.3 Hz, 1H), 7.32 (dd, *J* = 5.1, 2.8 Hz, 1H), 3.13 (s, 2H) ppm.

**$^{13}$C{$^1$H} NMR** (101 MHz, $CDCl_3$) δ = 190.7, 142.3, 132.6, 127.1, 127.1 (q, *J* = 273.8 Hz), 126.8, 39.4, 19.7 (q, *J* = 33.7 Hz), 8.0 (p, *J* = 50.1, 25.3 Hz) ppm.

**$^{19}$F NMR** (282 MHz, $CDCl_3$) δ = − 71.0 ppm.

**$^2$H NMR** (92 MHz, $CHCl_3$): δ = 1.08 (brs, 2D), 0.84 (brs, 2D) ppm.

**HRMS-ESI (m/z)** calculated for $C_{10}H_5D_4F_3SO$ $[M+H]^+$: 239.0650; found: 239.0646.

**IR** (ATR, neat) $\tilde{\nu}$ = 3106, 1676, 1509, 1391, 1349, 1312, 1232, 1209, 1155, 1124, 1041, 1012, 928, 899, 872, 783, 768, 749, 733, 708, 634, 585, 556, 497, 438 cm$^{-1}$.

Synthesis of **4w-d$_4$**:

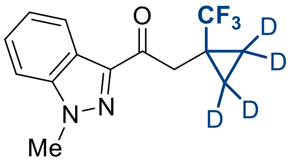


Prepared following general procedure B from 3-(1-((*tert*-butyldimethylsilyl)oxy)vinyl)-1-methyl-1*H*-indazole **3w** (58.1 mg, 0.20 mmol, 1.0 equiv.) and **1-d$_4$** (116.1 mg, 0.26 mmol, 1.3 equiv.). Compound **4w-d$_4$** was purified by chromatography on silica gel eluting with pentane/EtOAc (100/0→10/1 (v/v)) and obtained as a colorless oil (48.7 mg, 0.18 mmol, 89%).

**$^1$H NMR** (300 MHz, $CDCl_3$): δ = 8.35 (dt, *J* = 8.1, 1.0 Hz, 1H), 7.48−7.39 (m, 2H), 7.32 (ddd, *J* = 7.9, 5.6, 2.2 Hz, 1H), 4.13 (s, 3H), 3.44 (s, 2H) ppm.

**$^{13}$C{$^1$H} NMR** (101 MHz, $CDCl_3$) δ = 193.1, 141.9, 141.4, 127.3 (q, *J* = 273.6 Hz), 127.2, 123.9, 122.9, 109.4, 38.7, 36.5, 19.7 (q, *J* = 33.7 Hz), 8.2 (p, *J* = 25.1 Hz) ppm.

**$^{19}$F NMR** (282 MHz, $CDCl_3$) δ = − 70.7 ppm.

**$^2$H NMR** (92 MHz, $CHCl_3$): δ = 1.10 (brs, 2D), 0.91 (brs, 2D) ppm.

**HRMS-ESI (m/z)** calculated for $C_{14}H_9D_4F_3N_2O$ $[M+H]^+$: 287.1304; found: 287.1293.

**IR** (ATR, neat) $\tilde{\nu}$ = 3061, 2950, 2339, 1981, 1680, 1618, 1578, 1498, 1474, 1425, 1387, 1344, 1303, 1253, 1185, 1153, 1120, 1075, 1039, 1004, 955, 941, 924, 881, 863, 784, 770, 735, 712, 659, 606, 572, 535, 486, 444, 431, 409 cm$^{-1}$.

Synthesis of **6a-d$_4$**:

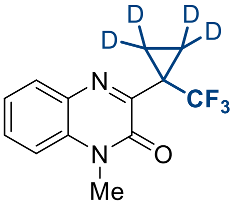


Prepared following general procedure C from 1-methylquinoxalin-2(1*H*)-one **5a** (64.6 mg, 0.40 mmol, 2.0 equiv.), $K_3PO_4$ (128.4 mg, 0.60 mmol, 3.0 equiv.) and **1-d$_4$** (90.0 mg, 0.20 mmol, 1.0 equiv.). Compound **6a-d$_4$** was purified by chromatography on silica gel eluting with pentane/EtOAc (100/0→80/20 (v/v)), and obtained as a light-yellow solid (33.1 mg, 0.12 mmol, 61%).

**$^1$H NMR** (400 MHz, $CDCl_3$): δ = 7.90 (dd, *J* = 8.0, 1.5 Hz, 1H), 7.60 (ddd, *J* = 8.7, 7.3, 1.5 Hz, 1H), 7.36 (ddd, *J* = 8.3, 7.4, 1.2 Hz, 1H), 7.31 (dd, *J* = 8.4, 1.2 Hz, 1H), 3.71 (s, 3H) ppm.

**$^{13}$C{$^1$H} NMR** (101 MHz, $CDCl_3$) δ = 154.4, 153.2, 133.8, 132.5, 131.5, 130.8, 125.9 (q, *J* = 274.8 Hz), 123.9, 113.7, 29.4, 27.8 (q, *J* = 34.5 Hz), 9.7 (p, *J* = 24.9 Hz) ppm.

**$^{19}$F NMR** (377 MHz, $CDCl_3$) δ = − 67.2 ppm.

**$^2$H NMR** (92 MHz, $CHCl_3$): δ = 1.51 (brs, 2D), 1.32 (brs, 2D) ppm.

**HRMS-ESI (m/z)** calculated for $C_{13}H_7D_4F_3N_2O$ $[M+H]^+$: 273.1147; found: 273.1151.

**IR** (ATR, neat) $\tilde{\nu}$ = 3080, 1980, 1935, 1652, 1600, 1585, 1554, 1470, 1417, 1340, 1316, 1270, 1203, 1151, 1130, 1066, 1050, 1034, 972, 934, 878, 860, 825, 798, 759, 731, 718, 634, 583, 565, 530, 509, 492, 481, 460, 450, 427, 416 $cm^{-1}$.

**Melting point:** 142 °C.

Synthesis of **6m-d$_4$**:

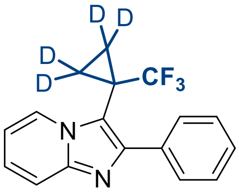


Prepared following general procedure C from 2-phenylimidazo[1,2-*a*]pyridine **5m** (117.5 mg, 0.60 mmol, 3.0 equiv.), $Cs_2CO_3$ (195.5 mg, 0.60 mmol, 3.0 equiv.) and **1-d$_4$** (90.0 mg, 0.20 mmol, 1.0 equiv.). Compound **6m-d$_4$** was purified by chromatography on silica gel eluting with pentane/EtOAc (100/0→9/1 (v/v)) and obtained as a yellow solid (49.6 mg, 0.16 mmol, 80%).

**$^1$H NMR** (300 MHz, $CDCl_3$): δ = 8.32 (dq, *J* = 6.9, 1.1 Hz, 1H), 8.06−7.94 (m, 2H), 7.68 (dt, *J* = 9.1, 1.2 Hz, 1H), 7.52−7.43 (m, 2H), 7.43−7.34 (m, 1H), 7.34−7.23 (m, 1H), 6.90 (td, *J* = 6.9, 1.3 Hz, 1H) ppm.

**$^{13}$C{$^1$H} NMR** (101 MHz, $CDCl_3$) δ = 146.8, 145.2, 133.7, 128.7 (q, *J* = 1.6 Hz), 128.4, 128.3, 126.8 (q, *J* = 275.1 Hz), 125.6, 124.5 (q, *J* = 2.6 Hz), 117.8, 114.2, 112.7, 18.1 (q, *J* = 35.4 Hz), 12.0 (brs) ppm.

**$^{19}$F NMR** (282 MHz, $CDCl_3$) δ = − 67.1 ppm.

**$^2$H NMR** (92 MHz, $CHCl_3$): δ = 1.62 (brs, 2D), 0.90 (brs, 2D) ppm.

**HRMS-ESI (m/z)** calculated for $C_{17}H_9D_4F_3N_2$ $[M+H]^+$: 307.1355; found: 307.1356.

**IR** (ATR, neat) $\tilde{\nu}$ = 3042, 2359, 2079, 1638, 1604, 1506, 1447, 1396, 1347, 1327, 1283, 1268, 1238, 1193, 1149, 1126, 1092, 1025, 978, 959, 938, 927, 917, 907, 869, 845, 778, 749, 738, 713, 692, 616, 604, 582, 545, 513, 471, 457, 424, 403 $cm^{-1}$.

**Melting point:** 146 °C.

Synthesis of **6q-d**$_4$:

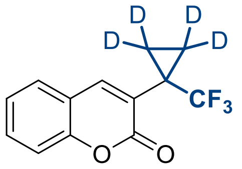


Prepared following general procedure C from coumarin **5q** (58.9 mg, 0.40 mmol, 2.0 equiv.), $K_3PO_4$ (128.5 mg, 0.60 mmol, 3.0 equiv.) and **1-d$_4$** (90.0 mg, 0.20 mmol, 1.0 equiv.). Compound **6q-d$_4$** was purified by chromatography on silica gel eluting with pentane/EtOAc (100/0→9/1 (v/v)) and obtained as a white solid (25.6 mg, 0.10 mmol, 47%).

**$^1$H NMR** (300 MHz, $CDCl_3$): δ = 7.88 (s, 1H), 7.55 (ddd, *J* = 8.4, 7.3, 1.6 Hz, 1H), 7.50 (dd, *J* = 7.8, 1.5 Hz, 1H), 7.33 (ddt, *J* = 8.4, 1.1, 0.6 Hz, 1H), 7.32−7.27 (m, 1H) ppm.

**$^{13}$C{$^1$H} NMR** (101 MHz, $CDCl_3$) δ = 160.3, 154.1, 147.1, 132.5, 128.2, 126.0 (q, *J* = 274.3 Hz), 124.7, 123.7, 118.8, 116.8, 24.2 (q, *J* = 35.1 Hz), 10.7−9.3 (m) ppm.

**$^{19}$F NMR** (288 MHz, $CDCl_3$) δ = − 70.1 ppm.

**$^2$H NMR** (92 MHz, $CHCl_3$): δ = 1.46 (brs, 2D), 1.07 (brs, 2D) ppm.

**HRMS-ESI (m/z)** calculated for $C_{13}H_5D_4F_3O_2$ $[M+H]^+$: 259.0878; found: 259.0882.

**IR** (ATR, neat) $\tilde{\nu}$ = 2343, 1722, 1631, 1607, 1576, 1491, 1457, 1341, 1278, 1251, 1226, 1184, 1157, 1142, 1119, 1065, 1036, 964, 953, 927, 908, 865, 822, 785, 771, 756, 731, 649, 624, 586, 563, 524, 471, 457, 418, 404 $cm^{-1}$.

**Melting point:** 134.5 °C.

Synthesis of **6ad-d**$_4$:

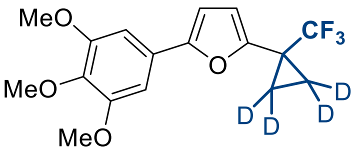


Prepared following general procedure D from 2-(3,4,5-trimethoxyphenyl)furan **5ad** (141.6 mg, 0.60 mmol, 3.0 equiv.), $Cs_2CO_3$ (195.5 mg, 0.60 mmol, 3.0 equiv.) and **1-d$_4$** (90.1 mg, 0.20 mmol, 1.0 equiv.). Compound **6ad-d$_4$** was purified by chromatography on silica gel eluting with pentane/EtOAc (100/0→40/1 (v/v)). Analytically pure **6ad-d$_4$** was obtained after preparative HPLC as colorless oil (61.3 mg, 0.18 mmol, 88%).

**$^1$H NMR** (400 MHz, $CDCl_3$): δ = 6.85 (s, 2H), 6.51 (d, *J* = 3.4 Hz, 1H), 6.40 (d, *J* = 3.4 Hz, 1H), 3.91 (s, 6H), 3.86 (s, 3H) ppm.

**$^{13}$C{$^1$H} NMR** (101 MHz, $CDCl_3$) δ = 153.7, 153.5, 149.3, 138.0, 126.4, 125.6 (q, *J* = 273.5 Hz), 111.6, 105.6, 101.3, 61.1, 56.3, 22.0 (q, *J* = 35.6 Hz), 10.0 (p, *J* = 25.4 Hz) ppm.

**$^{19}$F NMR** (377 MHz, $CDCl_3$) δ = − 69.5 ppm.

**$^2$H NMR** (92 MHz, $CHCl_3$): δ = 1.37 (brs, 2D), 1.23 (brs, 2D) ppm.

**HRMS-ESI (m/z)** calculated for $C_{17}H_{13}D_4F_3O_4$ $[M+H]^+$: 347.1403; found: 347.1411.

**IR** (ATR, neat) $\tilde{\nu}$ = 2938, 2838, 1977, 1743, 1584, 1549, 1497, 1455, 1417, 1339, 1240, 1203, 1124, 1063, 1027, 1004, 970, 925, 908, 867, 834, 786, 760, 733, 659, 630, 605, 533, 505, 478, 437, 420 $cm^{-1}$.

## CYCLIC VOLTAMMETRY MEASUREMENT

**Apparatus**: VersaSTAT 4 potentiostat (Princeton Applied Research)
**Software**: VersaStudio (version 2.44.4)
**Electrodes**: Glassy carbon (working electrode), platinum (counter electrode), Ag/AgCl (pseudo-reference electrode)
**Electrolyte**: 0.1 M $TBAPF_6$ in dry acetonitrile.
**Sweep rate**: 100 mV/s.
**Internal standard**: Ferrocene.
**Procedure:** Before the electrolyte solution had been added, the set-up was purged with nitrogen. Then the electrolyte solution was introduced under nitrogen flow and de-gassed by nitrogen bubbling. A spatula tip's worth of sulfonium salt **1** was put into the solution and mixed by a magnetic stirring bar. The stirring was stopped before measurement. The same procedure was repeated in the presence of a spatula tip's worth of ferrocene as internal standard.
**Potential of 1:** −1.45 V vs. $Fc/Fc^+$

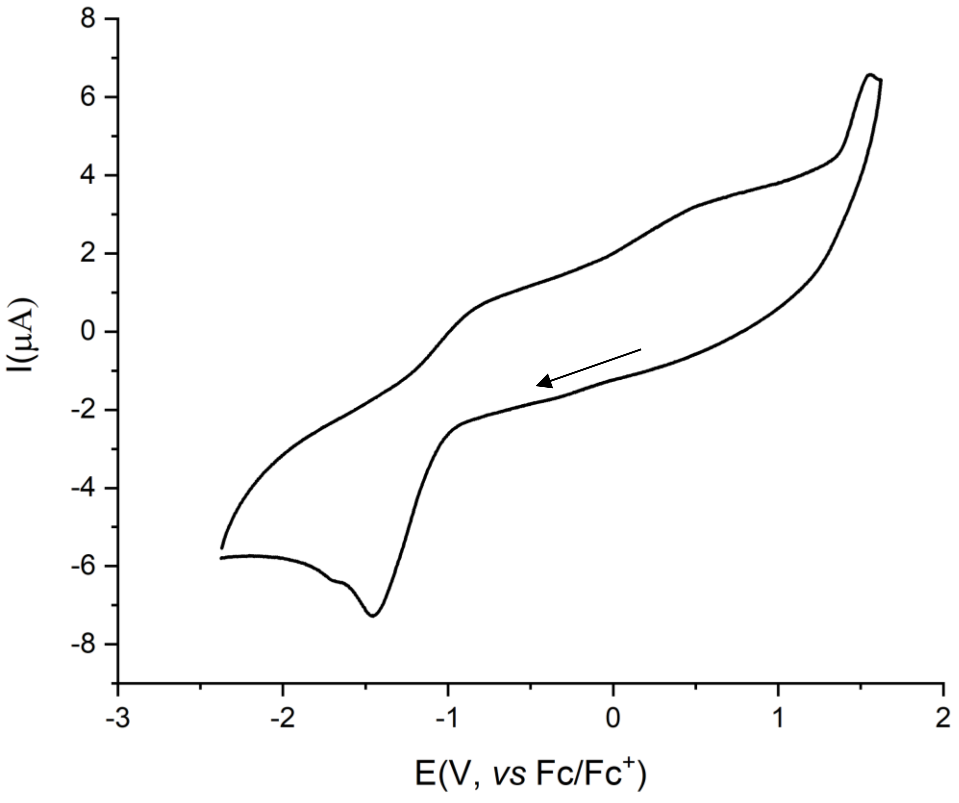


**Figure S4**: Cyclic Voltammetry of sulfonium salt **1** normalized by redox potential of ferrocene.

## STERN-VOLMER QUENCHING EXPERIMENTS[25]

Stern-Volmer Quenching Experiments of Sulfonium salt **1**

A stock solution of the photocatalyst Ir(ppy)$_3$ (0.2 mM, 3,260 mg in 25.0 mL acetonitrile) was prepared. Another stock solution of quencher Sulfonium salt **1** (10 mM, 44.2 mg in 10.0 mL acetonitrile) was prepared. Samples for measuring fluorescence emission were prepared in quartz cuvettes (QS 10.00 mm) by mixing equivalent volume of photo catalyst solution and quencher solution. After each measurement, the stock solution of **1** was diluted to half concentration. All solutions and samples were prepared in glovebox under nitrogen atmosphere.

The sample cell was first irradiated at 373.0 nm, then the emission wavelength with the range from 400 to 750 nm was recorded (shown in Figure **S5**).

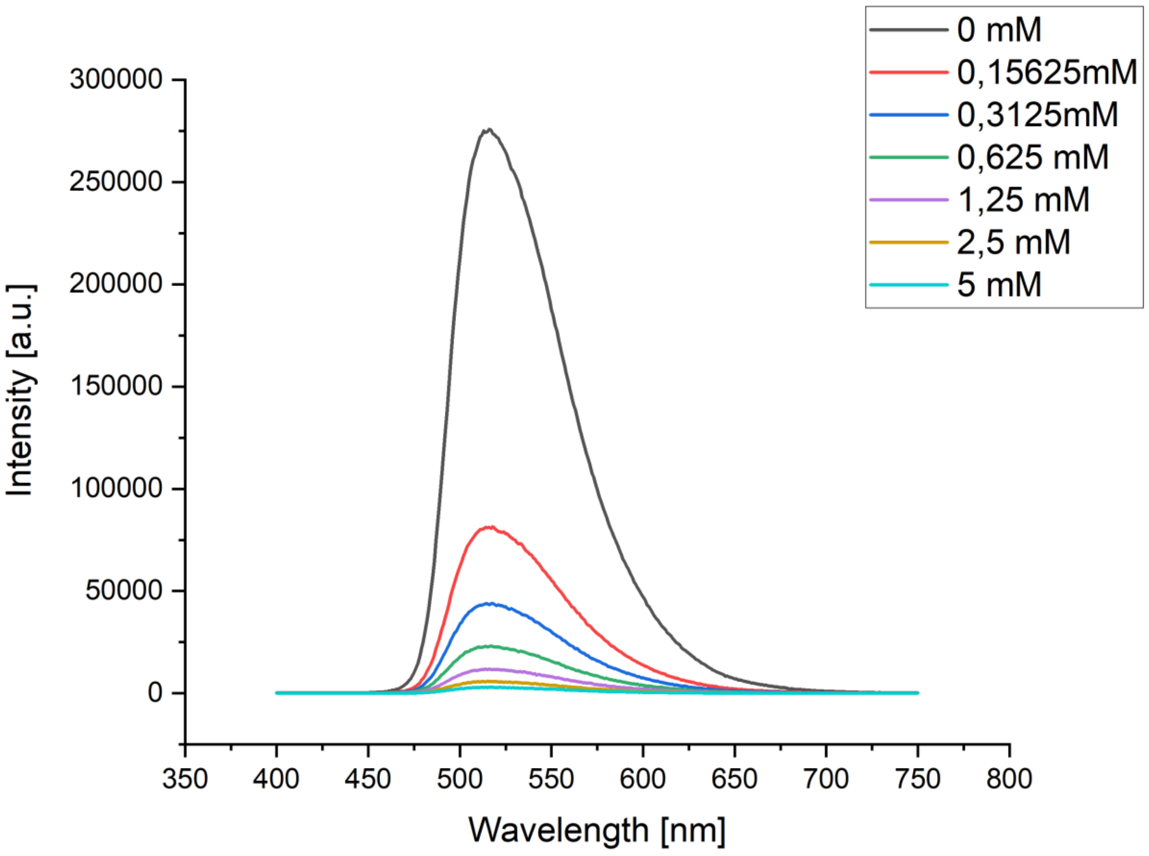


**Figure S5**: Emission spectrum of excited Ir(ppy)$_3$ with increasing concentration of **1**.

The intensity at 516 nm of each emission was extracted and plotted against quencher concentration according to Stern-Volmer equation:

$$I_0/I = 1 + K_{SV}[Q]$$

where $I_0$ is the emission intensity of excited $Ir(ppy)_3$ in the absence of quencher. $I$ is the emission intensity in the presence of quencher with various concentration. $K_{SV}$ is Stern-Volmer constant. $[Q]$ is the concentration of the quencher.

Deducing from the slope of Stern-Volmer plot, $K_{SV}$ is 18.80 $mM^{-1}$.

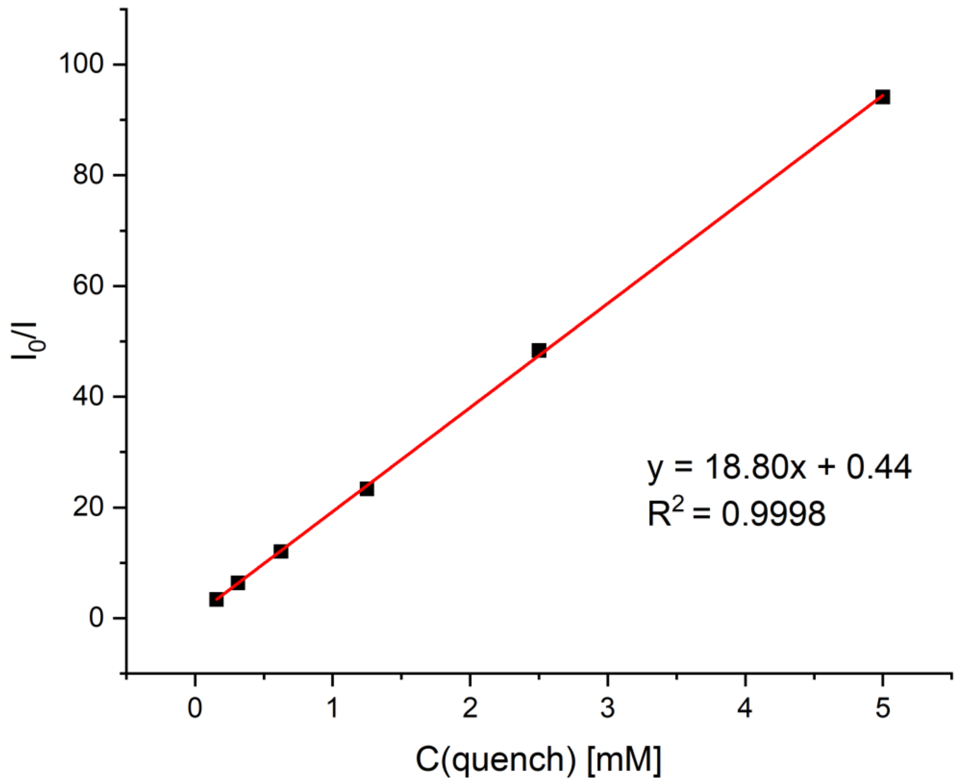


**Figure S6**: Stern-Volmer plot of $Ir(ppy)_3$ with varying concentration of **1**.

Stern-Volmer Quenching Experiments of Substrate **3a**

A stock solution of the photocatalyst $Ir(ppy)_3$ (0.2 mM, 3,260 mg in 25.0 mL acetonitrile) was prepared. Another stock solution of quencher **3a** (10 mM, 24.4 mg in 10.0 mL acetonitrile) was prepared. Samples for measuring fluorescence emission were prepared in quartz cuvettes (QS 10.00 mm) by mixing equivalent volume of photo catalyst solution and quencher solution. After each measurement, the stock solution of **3a** was diluted to half concentration. All solutions and samples were prepared in glovebox under nitrogen atmosphere.

The sample cell was first irradiated at 373.0 nm, then the emission wavelength with the range from 400 to 750 nm was recorded (shown in Figure **S7**).

Figure **S5**: Emission spectrum of excited Ir(ppy)3 with increasing concentration of

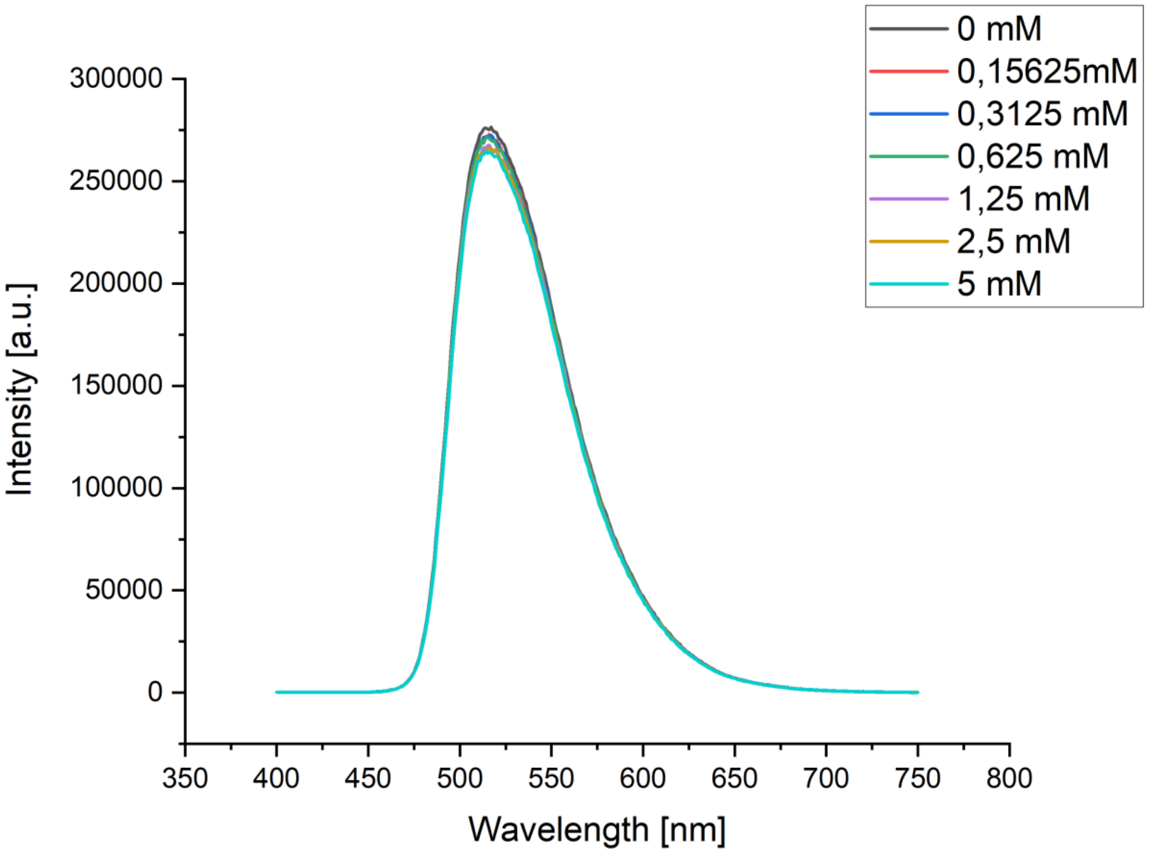


**Figure S7**: Emission spectrum of excited Ir(ppy)3 with increasing concentration of **3a**.

The intensity at 516 nm of each emission was extracted and plotted against quencher concentration according to Stern-Volmer equation:

$$I_0/I = 1 + K_{SV}[Q]$$

where $I_0$ is the emission intensity of excited Ir(ppy)3 in the absence of a quencher. $I$ is the emission intensity in the presence of quencher with various concentration. $K$ is Stern-Volmer constant. $[Q]$ is the concentration of the quencher.

Deducing from the slope of Stern-Volmer plot (Figure**S8**), $K_{SV}$ is 0.0063 $mM^{-1}$.

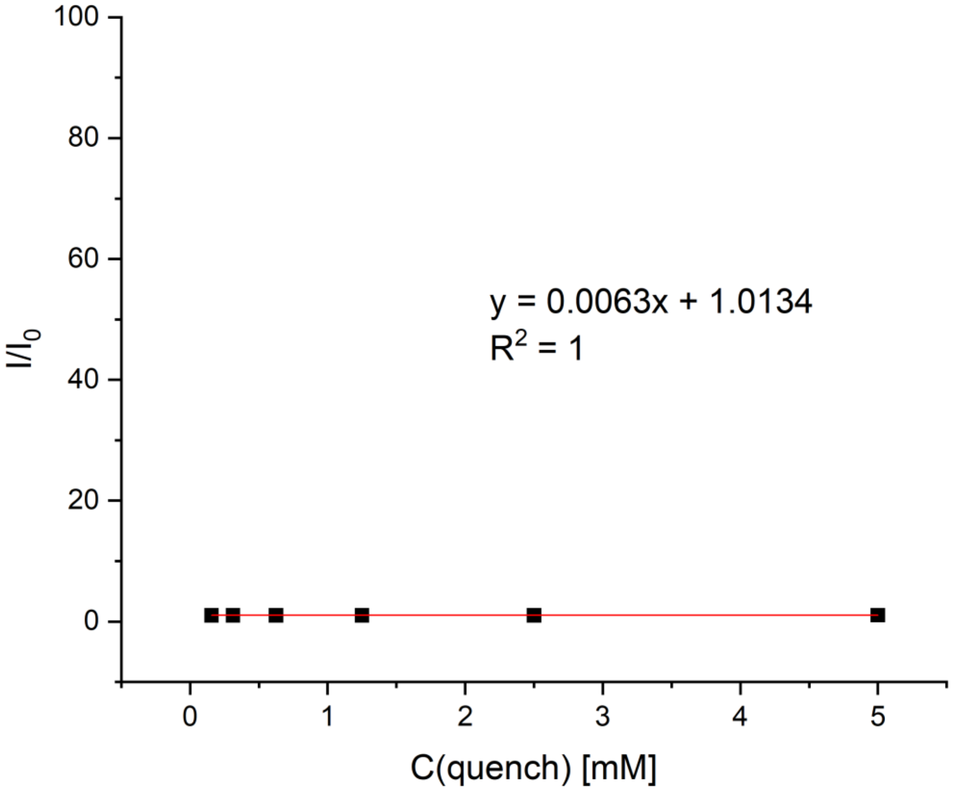


**Figure S8**: Stern-Volmer plot of $Ir(ppy)_3$ with varying concentration of **3a**.

Stern-Volmer Quenching Experiments of substrate **6x**

A stock solution of the photocatalyst $Ir(ppy)_3$ (0.2 mM, 3,260 mg in 25.0 mL acetonitrile) was prepared. Another stock solution of quencher **6x** (10 mM, 14.3 mg in 10.0 mL acetonitrile) was prepared. Samples for measuring fluorescence emission were prepared in quartz cuvettes (QS 10.00 mm) by mixing equivalent volume of photo catalyst solution and quencher solution. After each measurement, the stock solution of **6x** was diluted to half concentration. All solutions and samples were prepared in glovebox under nitrogen atmosphere.

The sample cell was first irradiated at 373.0 nm, then the emission wavelength with the range from 400 to 750 nm was recorded (shown in Figure **S9**).

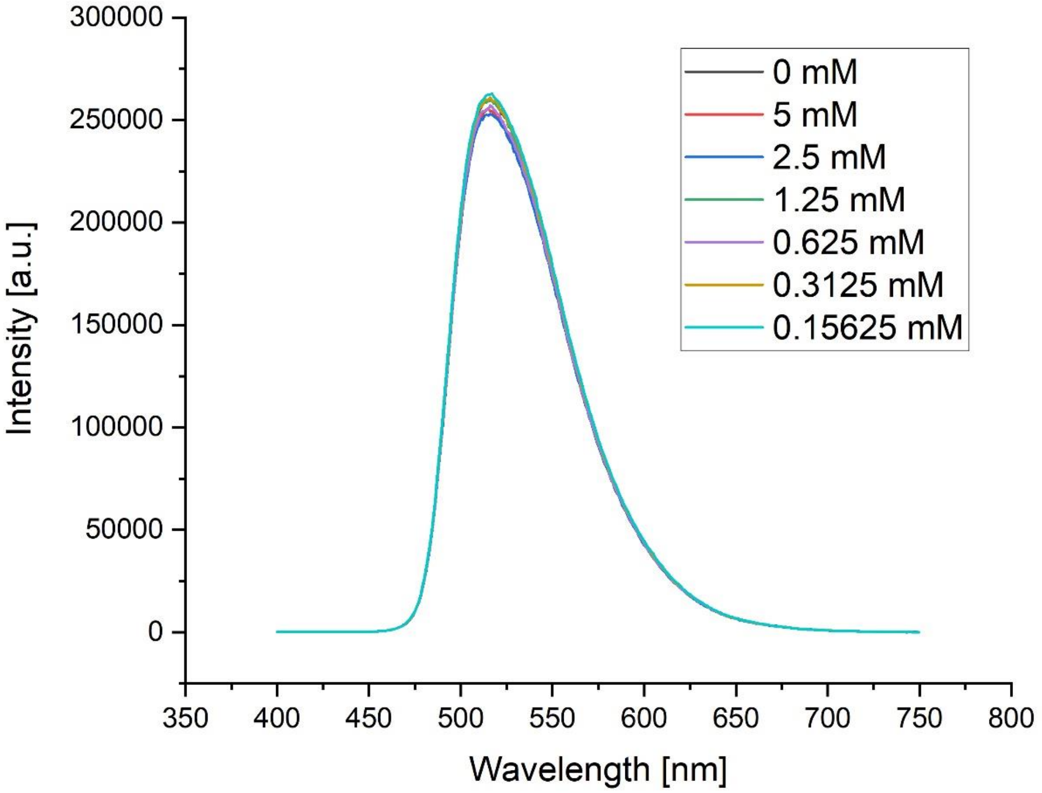


**Figure S9**: Emission spectrum of excited $Ir(ppy)_3$ with increasing concentration of **5x**.

The intensity at 516 nm of each emission was extracted and plotted against quencher concentration according to Stern-Volmer equation:

$$I_0/I = 1 + K_{SV}[Q]$$

where $I_0$ is the emission intensity of excited $Ir(ppy)_3$ in the absence of a quencher. $I$ is the emission intensity in the presence of quencher with various concentration. $K$ is Stern-Volmer constant. $[Q]$ is the concentration of the quencher.

Deducing from the slope of Stern-Volmer plot (Figure**S10**), $K_{SV}$ is 0.0059 $mM^{-1}$.

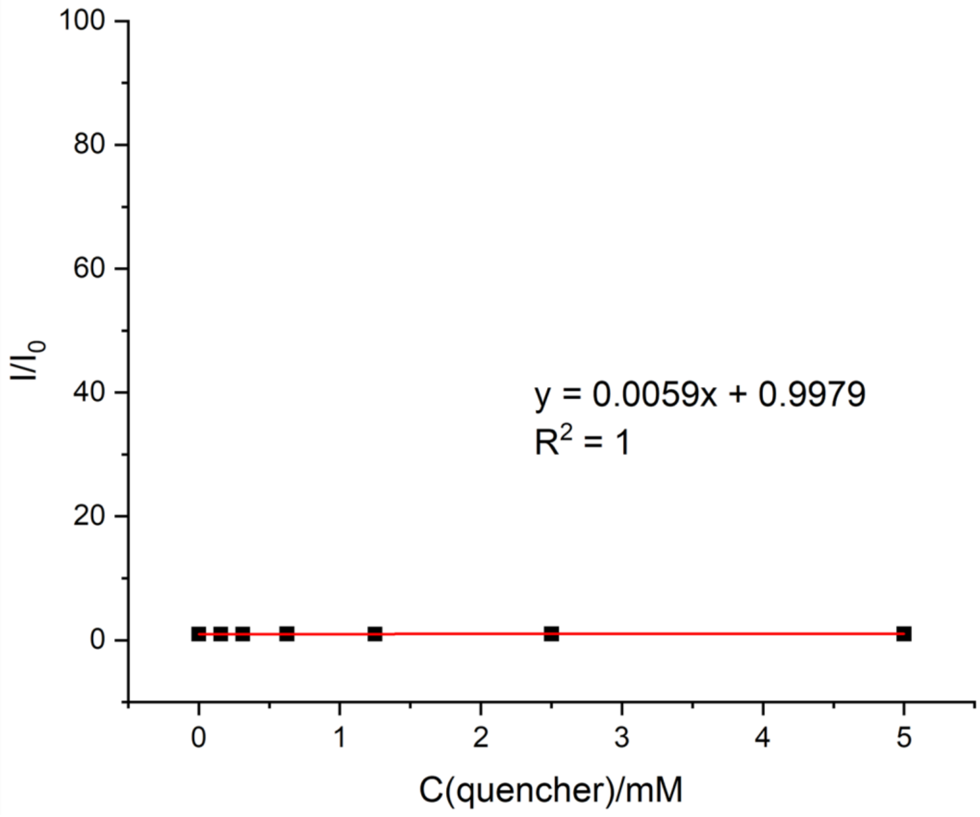


**Figure S10**: Stern-Volmer plot of Ir(ppy)$_3$ with varying concentration of **5x**.

## QUANTUM YIELD DETERMINATIONS

Determination of ferrioxalate actinometry

The photo flux of set-up was determined via a procedure (ferrioxalate actinometry) from literature.[26,27]

Solution preparation:

**Solution A** (0.05 M $H_2SO_4$ aqueous solution):

1.40 mL conc. $H_2SO_4$ (95% w/w, 17.8 M) was slowly added to a 500 mL volumetric flask charged with 200 mL deionized water. Then more deionized water was added until the 500 mL graduation mark was reached.

**Solution B** (0.006 M ferrioxalate solution):

A 250 mL volumetric flask was charged with potassium ferrioxalate ($K_3FeC_2O_4 \cdot 3H_2O$, 737 mg). Aforementioned solution A was added until the 250 mL graduation mark was reached. The solution was sonicated for 1 min to ensure that potassium ferrioxalate was completely dissolved. Note: Light should be avoided during the whole procedure.

**Solution C** (buffer solution):

Sodium acetate NaOAc (7.30 g, 89.0 mmol) and 50 mL deionized water were added into a 100 mL volumetric flask. Then 1.0 mL conc. $H_2SO_4$ (95% w/w, 17.8 M) was slowly added into the

mixture. Deionized water was added until the 100 mL graduation mark was reached. The solution was sonicated for 1 min to ensure the homogeneity.

**Measurement:**

Following procedure was finished while trying to avoid any background light.

A 25 mL Schlenk flask was fixed 5 cm away from a light source (PR160L-400 nm Kessil LED lamp, λmax= 440 nm, 25% of the maximum power). Then 4.0 mL solution B was transferred into the Schlenk flask and irradiated for 10 s. After the irradiation, 0.5 mL of the irradiated solution was transferred into a 10 mL foil-covered volumetric flask which had been charged with 10 mg 1,10-phenanthroline and 0.50 mL solution C. Deionized water was then added until the 10 mL graduation mark was reached. The flask was shaken for a while and stored in a dark room for ca. 1 h. Then around 0.4 mL solution was transferred to a quartz cuvette (path length 0.20 cm). The absorbance of the resulting solution was measured with UV/Vis spectroscopy at the range from 440 nm to 600 nm. Samples without irradiation and with irradiation for 20 s and 30 s were prepared and measured in this same method as well (Figure **S11**).

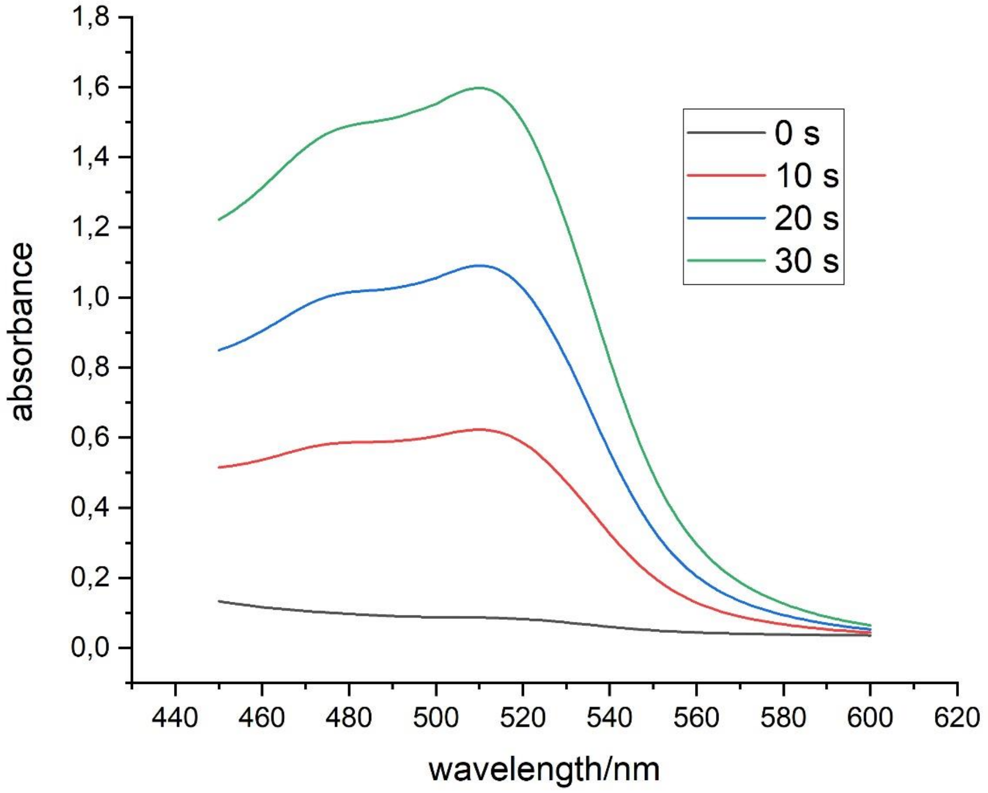


**Figure S11**: Actinometry: UV/Vis spectra of ferrioxalate/1,10-phenanthroline solutions.

The moles of ferrous ions produced via the irradiation can be derived from the equation below:

$$moles\ of\ Fe^{2+} = \frac{V_1 \times V_3 \times \Delta A(510\ nm)}{V_2 \times l \times \varepsilon(510\ nm)}$$

where $V_1$ is the irradiated volume (4 mL), $V_2$ is the aliquot of irradiated solution used for the measurement (0.5 mL), $V_3$ is the final volume of the solution in which ferrous ion was complexed with 1,10-phenanthroline (10 mL), l is the optical pathlength of quartz cuvette (1 cm), Δ =A(510 nm) is the difference of absorbance at 510 nm between the irradiated and non-irradiated ferrioxalate/1,10-phenanthroline solutions and ε(510 nm) is the molar absorptivity of $Fe(phen)_3^{2+}$ complex at 510 nm (11100 $L\cdot mol^{-1}\cdot cm^{-1}$).

The fraction of light absorbed at 440 nm $f$ can be calculated with the equation:

$$f = 1 - 10^{-A}$$

where $A$ is the measured absorbance (0.2217) of **solution B** at 440 nm.

A fitting plot of moles of $Fe^{2+}$ against irradiation time (s) is shown in Figure **S12.**

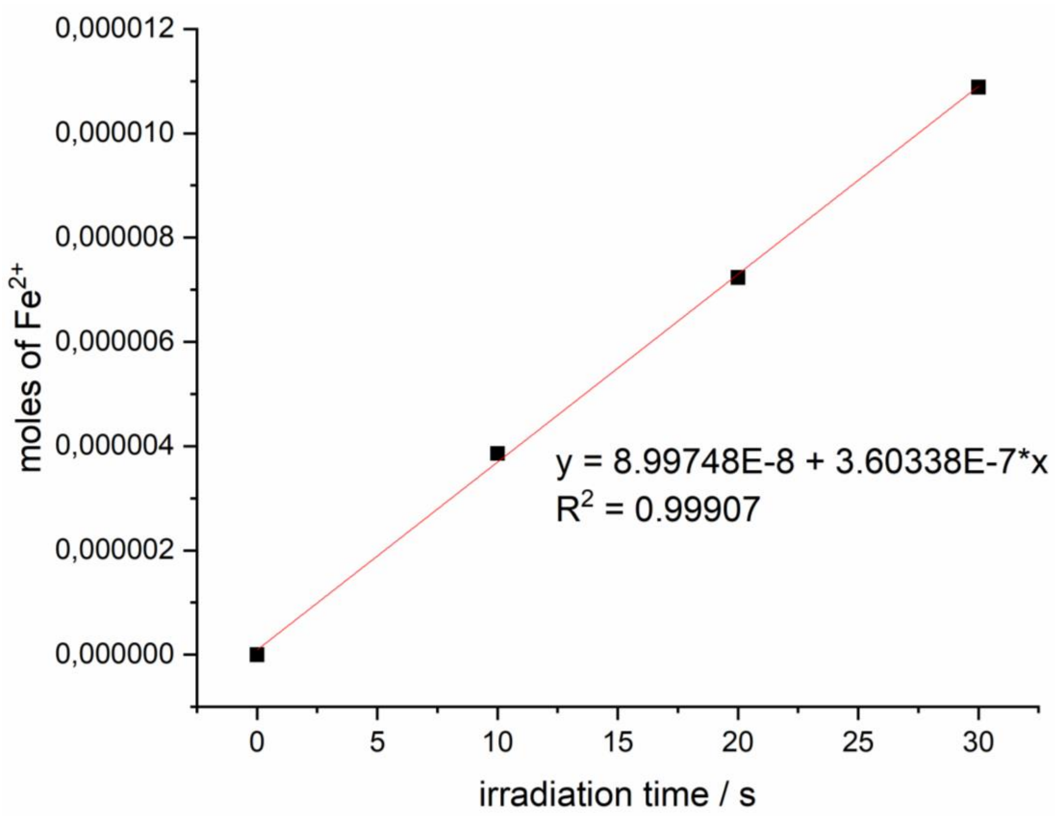


**Figure S12**: Moles of $Fe^{2+}$ against irradiation time.

Deriving from the slope of Figure **S12**:

$$\frac{d(moles\ Fe^{2+})}{dt} = 3.60338 \times 10^{-7}\ mol \cdot s^{-1}$$

And the relationship of amount of ion and time can be formulated as below:

$$moles\ Fe^{2+} = \Phi f \cdot photon\ flux \cdot t$$

where $\Phi$ is the quantum yield of the ferrioxalate (ca. 1.11, which was reported for a **solution B** at 436 nm)[27], $t$ is the irradiation time and $f$ is the fraction of light absorbed at 440 nm (0.3998).

Combine two equations with known terms:

$$photon\ flux = 8.1198 \times 10^{-7}\ Einstein/s$$

Quantum yield determination for silyl-enol-ether substrates:

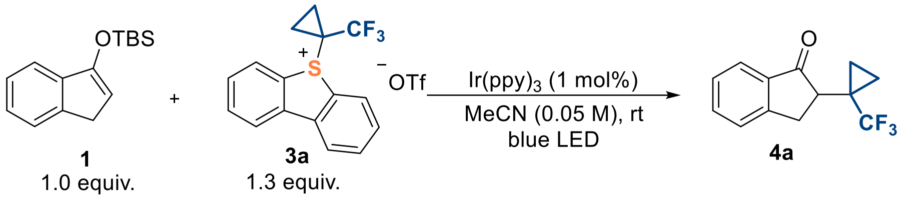


A Schlenk flask (the same flask used for ferrioxalate actinometry) equipped with a magnetic stir bar was charged with $Ir(pppy)_3$ (1.3 mg, 0.2 µmol, 1 mol%) and the flask was transferred to a nitrogen filled glovebox. Sulfonium salt **1** (116.6 mg, 0.26 mmol, 1.3 equiv.) and ((1*H*-inden-3-yl)oxy)(*tert*-butyl)dimethylsilane **3a** (50.1 mg, 0.2 mmol, 1.0 equiv.) were added and dissolved in MeCN (4 mL) under exclusion of light. The flask was sealed and covered with aluminum foil to avoid any background light and positioned at exactly the same place where ferrioxalate actinometry had been measured, *i.e.* 5.0 cm away from the light source (PR160L-400 nm Kessil LED lamp, $\lambda_{max}$= 440 nm, 25% of the maximum power). Once the aluminum foil covering the Schlenk flask was removed, the lamp is switched on to trigger the photo reaction. The lamp is switched off and on for every 60 s. During the off interval, 0.1 mL of the reaction mixture was taken by a syringe under nitrogen flow and transferred to a vial charged with ca. 0.2 mL $CD_3CN$ and a droplet of deionized water. $^{19}F$-NMR spectra were recorded and yields of this reaction at different time were obtained by taking the signal of the triflate anion from **1** as internal standard. The obtained values are listed in Table **S1.**

A $5.0\ \times 10^{-4}$ M solution of *fac*-$Ir(ppy)_3$ in acetonitrile was prepared, and a sample with pure acetonitrile was also prepared as blank sample. The absorbance of this solution at 440 nm was measured to be 1.86489, with which the fraction of light for *fac*-$Ir(ppy)_3$ was derived:

$$f = 1 - 10^{-A} = 1 - 10^{-1.86489} = 0.98635$$

The quantum yield $\Phi$ can be calculated with

$$\Phi = \frac{amount\ of\ products}{photon\ flux \cdot t \cdot f}$$

where photon flux is determined by ferrioxalate actinometry ($8.1198 \times 10^{-7}\ Einstein/s$), $f$ is the fraction of light absorbed by $Ir(ppy)_3$ at 440 nm (0.98635).

**Table S1**: Quantum yield determination for silyl-enol-ether substrates.

| reaction time | conversion | moles of product | Quantum yield |
|---|---|---|---|
| 60 s | 11% | 0.022 mmol | 0.458 |
| 120 s | 21% | 0.042 mmol | 0.437 |
| 180 s | 29% | 0.058 mmol | 0.402 |
| 240 s | 38% | 0.076 mmol | 0.395 |

The average quantum yield is **0.423**.

Quantum yield determination for the catalyst-free transformation of heteroaromatic substrates:

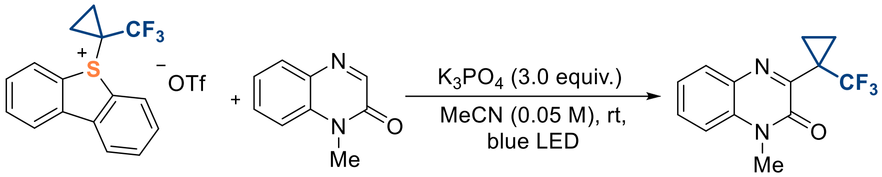


A Schlenk flask (the same flask used for ferrioxalate actinometry) equipped with a magnetic stir bar was transferred to a nitrogen filled glovebox. Sulfonium salt **1** (88.5 mg, 0.26 mmol, 1.0 equiv.), $K_3PO_4$ (127.4 mg. 0.6 mmol, 3.0 equiv.) and 1-methylquinoxalin-2(1*H*)-one **6a** (64.6 mg, 0.4 mmol, 2.0 equiv.) were added and dissolved in MeCN (4 mL) under exclusion of light. The flask was sealed and covered with aluminum foil to avoid any background light and positioned at exactly the same place where ferrioxalate actinometry had been measured, *i.e.* 5.0 cm away from the light source (PR160L-400 nm Kessil LED lamp, $\lambda_{max}$= 440 nm, 25% of the maximum power). The reaction mixture was stirred in the dark for 30 min. to ensure EDA complex formation. Once the aluminum foil covering the Schlenk flask was removed, the lamp is switched on to trigger the photo reaction. The lamp is switched off and on for every 10 min. During the off interval, 0.1 mL of the reaction mixture was taken by a syringe under nitrogen flow and transferred to a vial charged with ca. 0.2 mL $CD_3CN$ and a droplet of deionized water. $^{19}F$ NMR spectra were recorded and consumption of **1** at different times were obtained by taking the signal of the triflate anion from **1** as internal standard. The obtained values are listed in Table **S2**.

The absorbance of the reaction mixture at 440 nm was measured to be 0.00154, with which the fraction of light for the EDA complex was derived:

$$f = 1 - 10^{-A} = 1 - 10^{-0.00154} = 0.00355$$

The quantum yield $\Phi$ can be calculated with

$$\Phi = \frac{amount\ of\ products}{photon\ flux \cdot t \cdot f}$$

where photon flux is the determined by ferrioxalate actinometry ($8.1198 \times 10^{-7}\ Einstein/s$), $f$ is the fraction of light absorbed by the EDA complex at 440 nm (0.00355).

**Table S2**: Quantum yield determination for the catalyst-free transformation of heteroaromatic substrates.

| reaction time | conversion | moles of 1 consumed | Quantum yield |
|---|---|---|---|
| 600 s | 5% | 0.012 mmol | 5.782 |
| 1200 s | 9% | 0.018 mmol | 5.203 |
| 1800 s | 14% | 0.028 mmol | 5.396 |
| 2400 s | 18% | 0.036 mmol | 5.493 |

The average quantum yield $\Phi$ is **5.469**.

Quantum yield determination for the photoredox-catalytic transformation of heteroaromatic substrates:

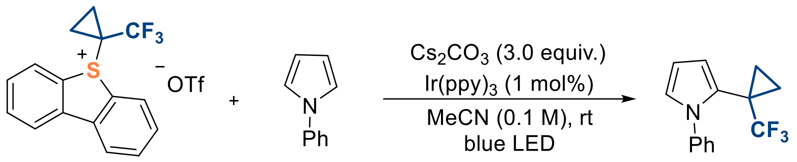


A Schlenk flask (the same flask used for ferrioxalate actinometry) equipped with a magnetic stir bar was charged with Ir(ppy)$_3$ (1.3 mg, 0.2 µmol, 1 mol%) and the flask was transferred to a nitrogen filled glovebox. Sulfonium salt **1** (88.5 mg, 0.26 mmol, 1.0 equiv.), $Cs_2CO_3$ (195.5 mg. 0.6 mmol, 3.0 equiv.) and 1-phenyl-1*H*-pyrrole **6x** (86.1 mg, 0.6 mmol, 3.0 equiv.) were added and dissolved in MeCN (2 mL) under exclusion of light. The flask was sealed and covered with aluminum foil to avoid any background light and positioned at exactly the same place where ferrioxalate actinometry had been measured, *i.e.* 5.0 cm away from the light source (PR160L-400 nm Kessil LED lamp, $\lambda_{max}$= 440 nm, 25% of the maximum power). Once the aluminum foil covering the Schlenk flask was removed, the lamp is switched on to trigger the photo reaction. The lamp is switched off and on for every 180 s. During the off interval, 0.1 mL of the reaction mixture was taken by a syringe under nitrogen flow and transferred to a vial charged with ca. 0.2 mL $CD_3CN$ and a droplet of deionized water. $^{19}$F NMR spectra were recorded and consumption of **1** at different time were obtained by taking the signal of the triflate anion from **1** as internal standard. The obtained values are listed in Table **S3**.

A $1.0 \times 10^{-3}$ M solution of *fac*-Ir(ppy)$_3$ in acetonitrile was prepared, and a sample with pure acetonitrile was also prepared as blank sample. The absorbance of this solution at 440 nm was measured to be 2.42128, with which the fraction of light for *fac*-Ir(ppy)$_3$ was derived:

$$f = 1 - 10^{-A} = 1 - 10^{-2.42128} = 0.99621$$

The quantum yield Φ can be calculated with

$$\Phi = \frac{amount\ of\ products}{photon\ flux \cdot t \cdot f}$$

where photon flux is the determined by ferrioxalate actinometry ($8.1198 \times 10^{-7}\ Einstein/s$), $f$ is the fraction of light absorbed by Ir(ppy)$_3$ at 440 nm (0.99621).

**Table S3**: Quantum yield determination for heteroaromatic substrates.

| reaction time | conversion | moles of 1 consumed | Quantum yield |
|---|---|---|---|
| 180 s | 24% | 0.048 mmol | 0.329 |
| 360 s | 38% | 0.076 mmol | 0.261 |
| 540 s | 52% | 0.10 mmol | 0.238 |
| 720 s | 68% | 0.13 mmol | 0.234 |

The average quantum yield Φ is **0.266**.

## RADICAL TRAPPING EXPERIMENTS

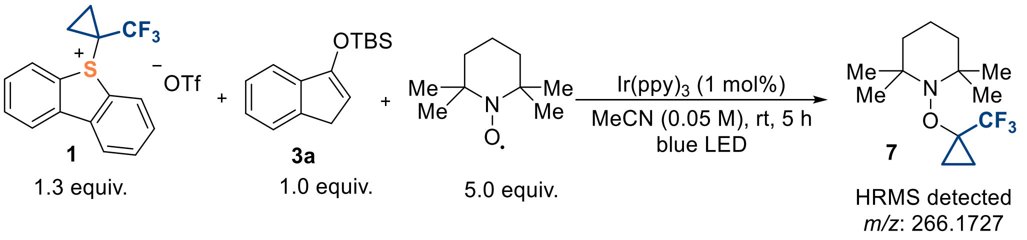


Sulfonium salt **1** (115.0 mg, 0.26 mmol, 1.3 equiv.), **3a** (49.0 mg, 0.2 mmol, 1.0 equiv.), TEMPO (156.0 mg, 1.0 mmol, 5.0 equiv.), $Ir(ppy)_3$ (1.3 mg, 2.0 µmol, 1 mol%) and Acetonitrile (4 mL) were added to a Schlenk flask equipped with a magnetic stir bar inside a nitrogen filled glovebox. The sealed flask was transferred to a photoreactor equipped with blue LED strips (wavelength: 460-465 nm). The mixture was irradiated at 50% intensity and stirred for 5 hours. A sample of the mixture was taken and a crude $^{19}F$ NMR was measured.

Isolation of trapped radical **7** failed via silica gel column chromatography.

**$^{19}$F NMR** (283 MHz, $CD_3CN$): δ = – 74.3 ppm.

**HRMS-ESI (m/z)** calculated for $C_{13}H_{22}F_3NO$ $[M+H]^+$: 266.1726; found: 266.1727

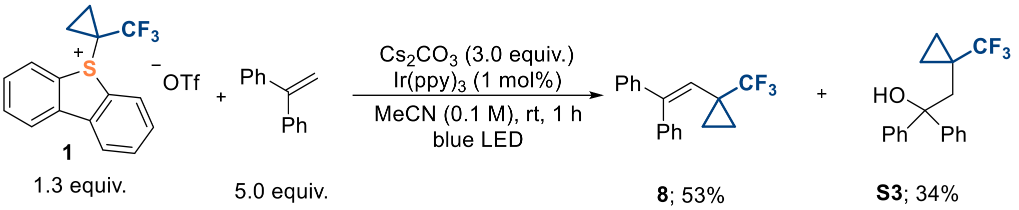


Sulfonium salt **1** (88.5 mg, 0.20 mmol, 1.0 equiv.), $Cs_2CO_3$ (195.5 mg, 0.6 mmol, 3.0 equiv.) and $Ir(ppy)_3$ (1.3 mg, 2.0 µmol, 1 mol%) were added to a Schlenk flask inside a nitrogen filled glovebox. MeCN (2 mL) were added and the flask was transferred to a Schlenk line, 1,1-diphenylethylene (180.3 mg, 176.3 µL, 1.0 mmol, 5.0 equiv.) was added under nitrogen flow and the reaction mixture was degassed by freeze-pump-thaw technique. Afterwards the reaction mixture was transferred to a photoreactor equipped with blue LED strips (wavelength: 460-465 nm). The reaction was irradiated at 50% intensity and stirred for 1 hour. The reaction mixture was diluted with DCM (3 mL) and washed with water (5 mL). The organic phase was dried over $Na_2SO_4$ and the solvent was removed under reduced pressure. The residue obtained was purified by silica gel column chromatography eluting with pure pentane. Compound **8** was obtained analytically pure after preparative HPLC as colorless oil (30.8 mg, 0.11 mmol, 53%).

**$^{1}$H NMR** (300 MHz, CDCl3): δ = 7.38−7.30 (m, 3H), 7.29−7.15 (m, 7H), 6.25 (s, 1H), 1.01−0.92 (m, 2H), 0.51−0.41 (m, 2H) ppm.

**$^{13}$C{$^{1}$H} NMR** (101 MHz, CDCl3) δ = 147.4, 142.8, 139.3, 130.1, 128.3, 128.2, 128.0, 127.9, 127.8, 126.9 (q, *J* = 273.6 Hz), 121.7 (q, *J* = 1.9 Hz), 24.1 (q, *J* = 33.2 Hz), 12.3 (q, *J* = 2.1 Hz) ppm.

**$^{19}$F NMR** (288 MHz, $CD_3CN$) δ = − 69.5 ppm.

**HRMS-EI (m/z)** calculated for $C_{18}H_{15}F_3$ [M]$^{\cdot}$: 288.1120; found: 288.1125.

**IR** (ATR, neat) $\tilde{\nu}$ = 3056, 3024, 1599, 1494, 1444, 1368, 1348, 1255, 1160, 1124, 1073, 1032, 1017, 957, 935, 917, 888, 862, 765, 745, 726, 697, 667, 647, 612, 593, 550, 493, 462, 436, 418, 408 cm$^{-1}$.

Compound **S3** was obtained as a second fraction of the same column chromatography eluting with pentane/EtOAc (100/0→90/10 (v/v)) as a colorless oil (20.9 mg, 0.07 mmol, 34%).

$^{1}$**H NMR** (400 MHz, CDCl3): δ = 7.47−7.40 (m, 4H), 7.36−7.29 (m, 4H), 7.28−7.20 (m, 2H), 2.82 (s, 2H), 2.37 (br s, 1H), 0.84−0.79 (m, 2H), 0.74−0.63 (m, 2H) ppm.

$^{13}$**C{$^{1}$H} NMR** (101 MHz, CDCl3) δ = 147.1, 128.4, 127.6 (q, *J* = 273.5 Hz), 127.2, 125.8, 77.9, 40.3, 20.2 (q, *J* = 32.1 Hz), 9.3 (q, *J* = 2.9 Hz) ppm.

$^{19}$**F NMR** (288 MHz, $CD_3CN$) δ = − 70.3 ppm.

**HRMS-ESI (m/z)** calculated for $C_{18}H_{17}F_3O$ [M+Na]$^{+}$: 329.1124; found: 329.1119.

**IR** (ATR, neat) $\tilde{\nu}$ = 3558, 3480, 3026, 2930, 2125, 1715, 1598, 1493, 1447, 1392, 1305, 1150, 1119, 1057, 1033, 1000, 956, 914, 876, 836, 750, 697, 661, 607, 593, 554, 486, 462, 446, 430, 404 cm$^{-1}$.

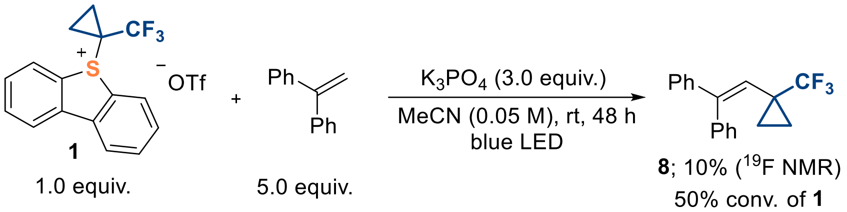


1.0 equiv. 5.0 equiv.

**8**; 10% ($^{19}$F NMR)
50% conv. of **1**

A Schlenk flask equipped with a magnetic stir bar was charged with Sulfonium salt **1** (88.5 mg. 0.2 mmol, 1.0 equiv.) and $K_3PO_4$ (127.4 mg, 0.6 mmol, 3.0 equiv.) inside a nitrogen filled glovebox. Subsequently MeCN (4 mL) was added and the flask was sealed and transferred to a Schlenk line, 1,1-diphenylethylene (180.3 mg, 176.3 µL, 1.0 mmol, 5.0 equiv.) was added under nitrogen flow and the reaction mixture was degassed by freeze-pump-thaw technique. Afterwards the reaction mixture was transferred to a photoreactor equipped with blue LED strips (wavelength: 460-465 nm). The reaction was irradiated at 50% intensity and stirred for 48 h. After that time a sample was taken, diluted with MeCN-$d_3$ and analyzed by $^{19}$F NMR using the triflate signal as internal standard.

## UV/Vis STUDIES

UV/Vis absorption spectra were measured in 10 mm quartz cuvettes using a Jasco V-630 spectrometer to confirm the formation of an EDA complex. All samples were prepared in MeCN:

Sample a): **5a** (0.01 M);

Sample b): **1** (0.005 M);

Sample c): **1** (0.005 M) + **5a** (0.01 M);

Sample d): **1** (0.005 M) + $K_3PO_4$ (0.015 M).

Samples d) was kept under exclusion of light for 15 h after mixing to ensure enough $K_3PO_4$ had been dissolved before measurement.

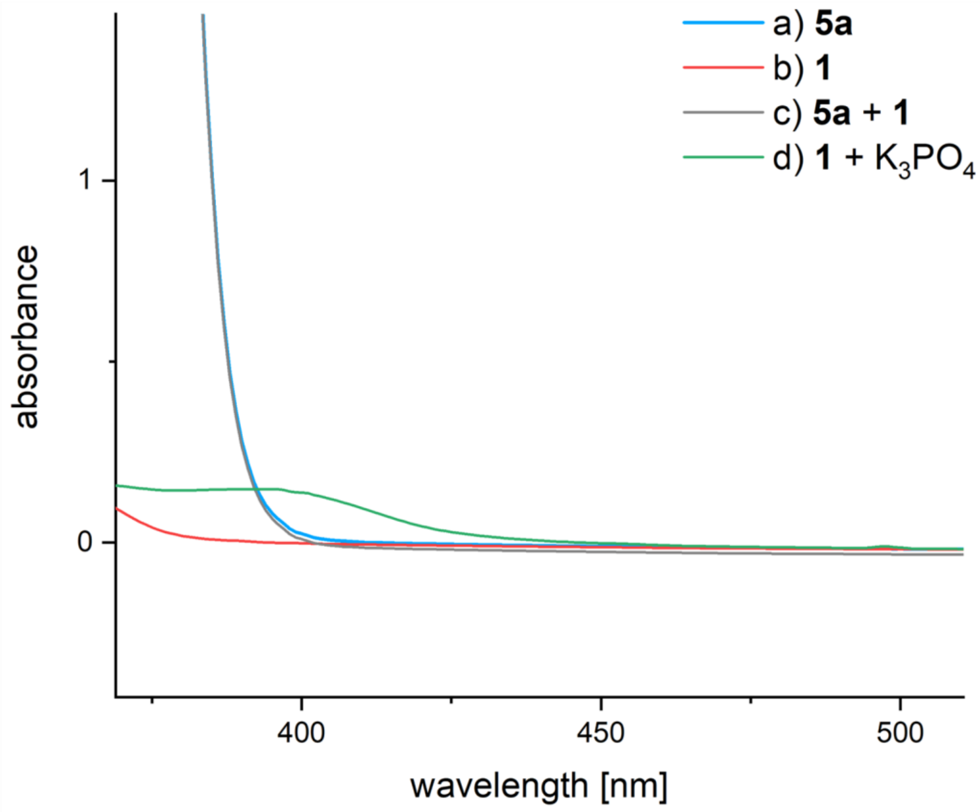


**Figure S13**: UV/Vis absorption spectra of individual reaction components and mixture, measured directly after mixing.

From the UV/Vis absorption spectra obtained (Figure **S13**), an evident red shift can be observed for sample d).

## ELECTRON PARAMAGNETIC RESONANCE (EPR) STUDIES

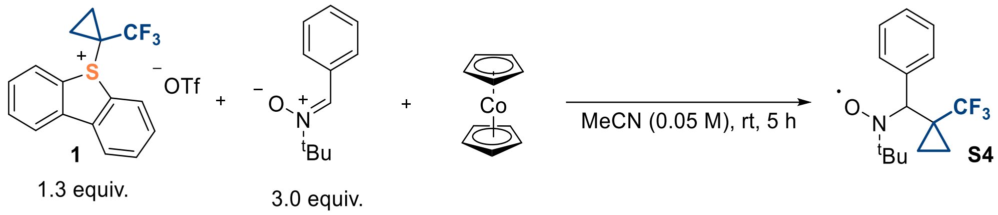


In a nitrogen filled glovebox, sulfonium salt **1** (22.5 mg, 0.05 mmol, 1.0 equiv.), phenyl *N*-tertbutylnitrone (PBN) (27.3 mg, 0.15 mmol, 3.0 equiv.), $CoCp_2$ (10.4 mg, 0.055 mmol, 1.1 equiv.) and acetonitrile (1 mL) were added to a Schlenk flask equipped with a magnetic stir bar. The reaction mixture was stirred at ambient temperature for 5 hours. After that time the reaction mixture was transferred back to a nitrogen filled glovebox and an aliquot was taken, diluted with MeCN and analyzed by EPR spectroscopy.

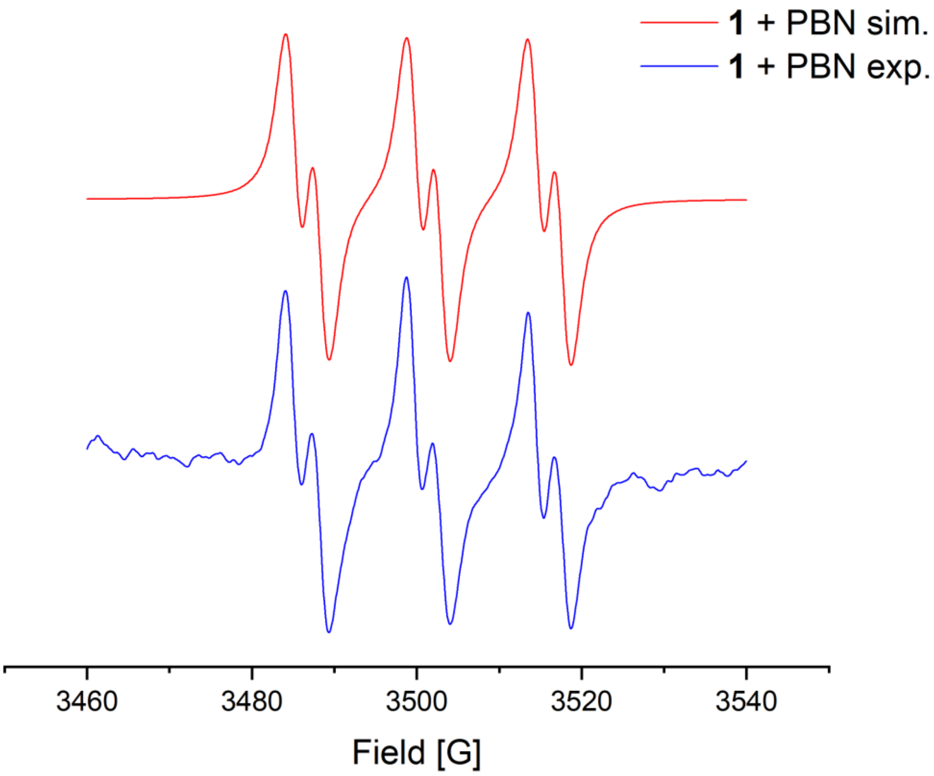


**Figure S14**: X-band EPR spectrum (MeCN, 298 K): Experimental signals of **1** mixed with $CoCp_2$ and PBN (in blue); Simulated signals of product S4 (in red); Fitting Parameters: g = 2.00644, a(1x$^{14}$N) = 14.7 G, a(1x$^{1}$H) = 3.0 G.

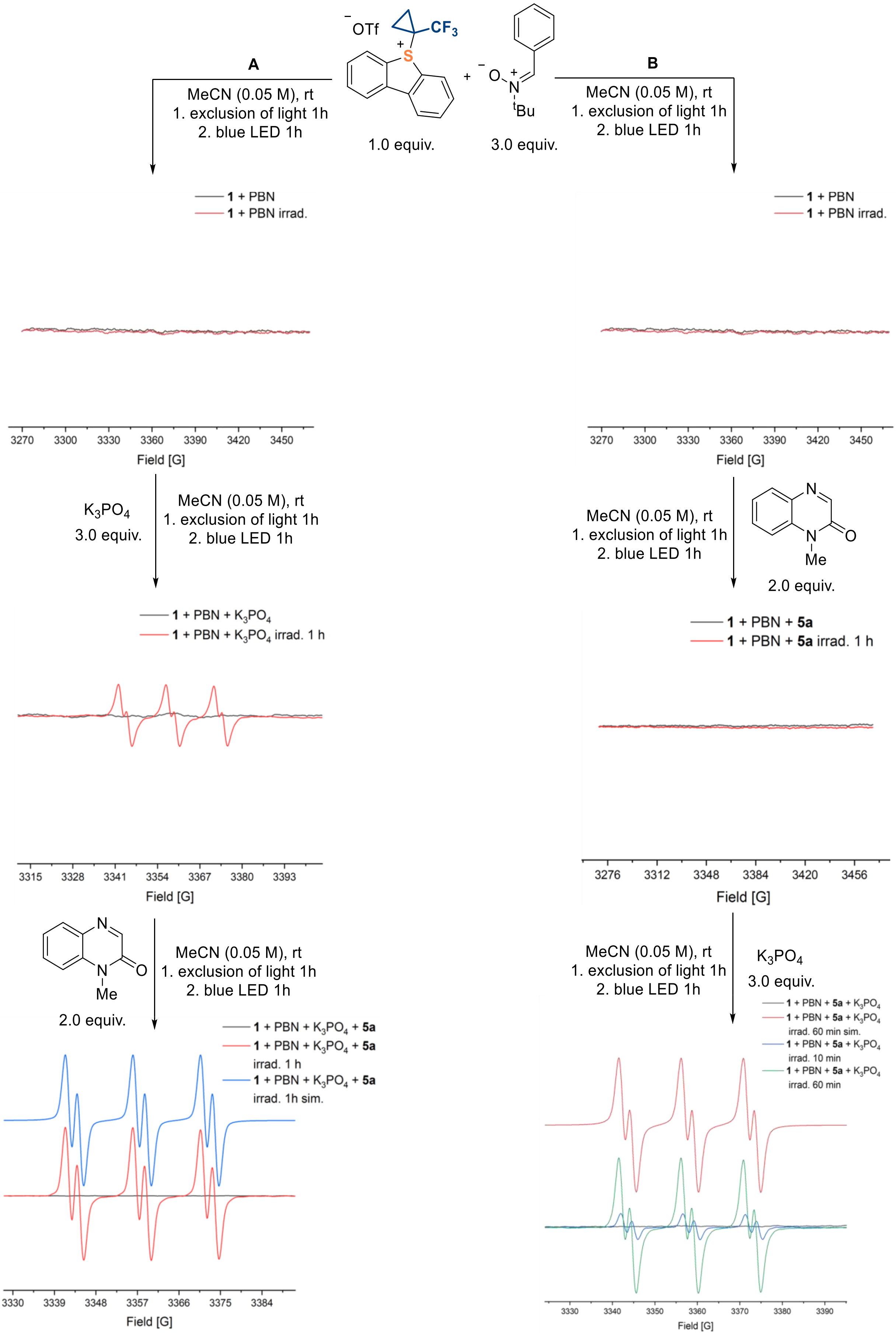


**Scheme S1**: Stepwise reaction tracking of the catalyst-free TFCp transfer.

In a nitrogen filled glovebox, a Schlenk flask equipped with a magnetic stir bar was charged with sulfonium salt **1** (22.5 mg, 0.05 mmol, 1.0 equiv.) and phenyl *N*-tertbutylnitrone (PBN) (27.3 mg, 0.15 mmol, 3.0 equiv.). The flask was wrapped in aluminum foil and acetonitrile (1 mL) was added. The flask was sealed and stirred at ambient temperature for 1 h in the dark. Afterwards, the flask was transferred back to the nitrogen filled glovebox and an aliquot was taken, diluted with MeCN, and analyzed by EPR spectroscopy, while maintaining the exclusion of light. The same sample was transferred to a photoreactor equipped with blue LED strips (wavelength: 460-465 nm) and irradiated at 50% intensity for 1 hours and analyzed by EPR spectroscopy (Scheme **S1**; Figure **S15**).

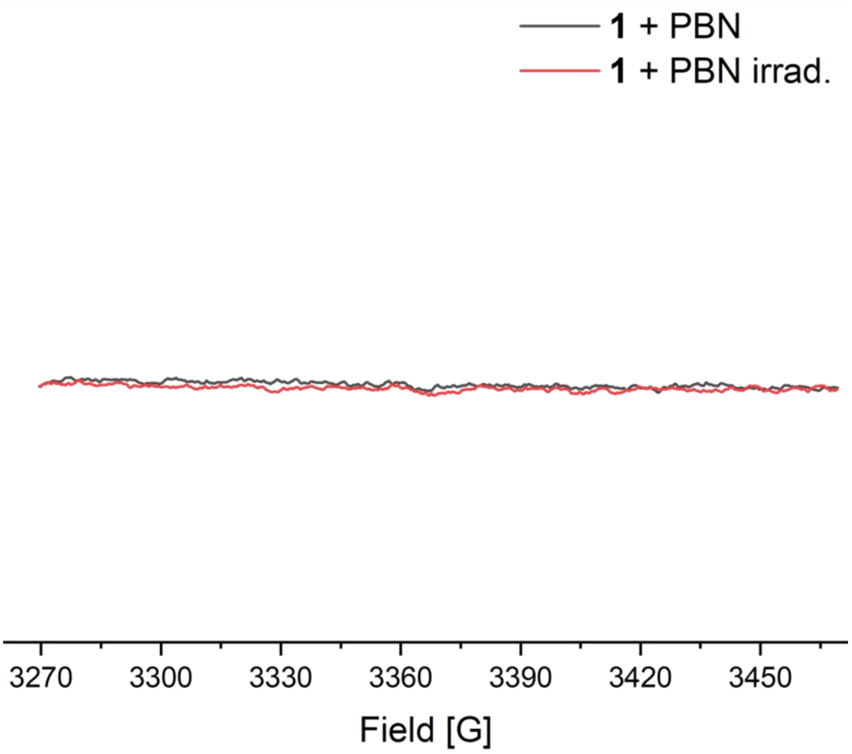


**Figure S15**: X-band EPR spectrum (MeCN, 298 K): Experimental signals of **1** mixed with PBN in the dark (grey) and after irradiation (red).

The reaction mixture was then divided in two equal parts, reaction mixture A and B which were treated differently as described below:

**Reaction mixture A**:

$K_3PO_4$ (16.2 mg, 0.075 mmol, 3.0 equiv.)was added to reaction mixture A and stirred under ambient temperature for 1 h in the dark. Afterwards, the flask was transferred back to the nitrogen filled glovebox and an aliquot was taken, diluted with MeCN, and analyzed by EPR spectroscopy, while maintaining the exclusion of light. The same sample was later transferred to a photoreactor equipped with blue LED strips (wavelength: 460-465 nm) and irradiated at 50% intensity for 1 hours and analyzed by EPR spectroscopy (Scheme **S1**; Figure **S16**).

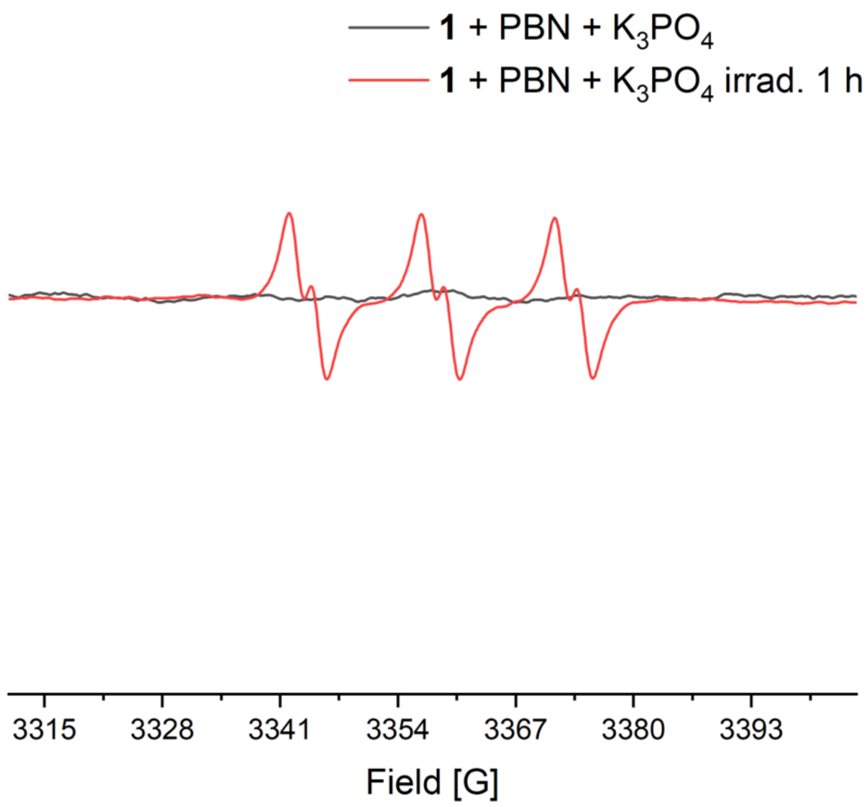


**Figure S16**: X-band EPR spectrum (MeCN, 298 K): Experimental signals of **1** mixed with PBN $K_3PO_4$ and in the dark (grey) and after irradiation (red).

Quinoxalinone (8.0 mg, 0.050 mmol, 2.0 equiv.) was then added to reaction mixture A and stirred under ambient temperature for 1 h in the dark. Afterwards, the flask was transferred back to the nitrogen filled glovebox and an aliquot was taken, diluted with MeCN and analyzed by EPR spectroscopy while maintaining the exclusion of light. The same sample was later transferred to a photoreactor equipped with blue LED strips (wavelength: 460-465 nm) and irradiated at 50% intensity for 1 hours and analyzed by EPR spectroscopy (Scheme **S1**; Figure **S17**).

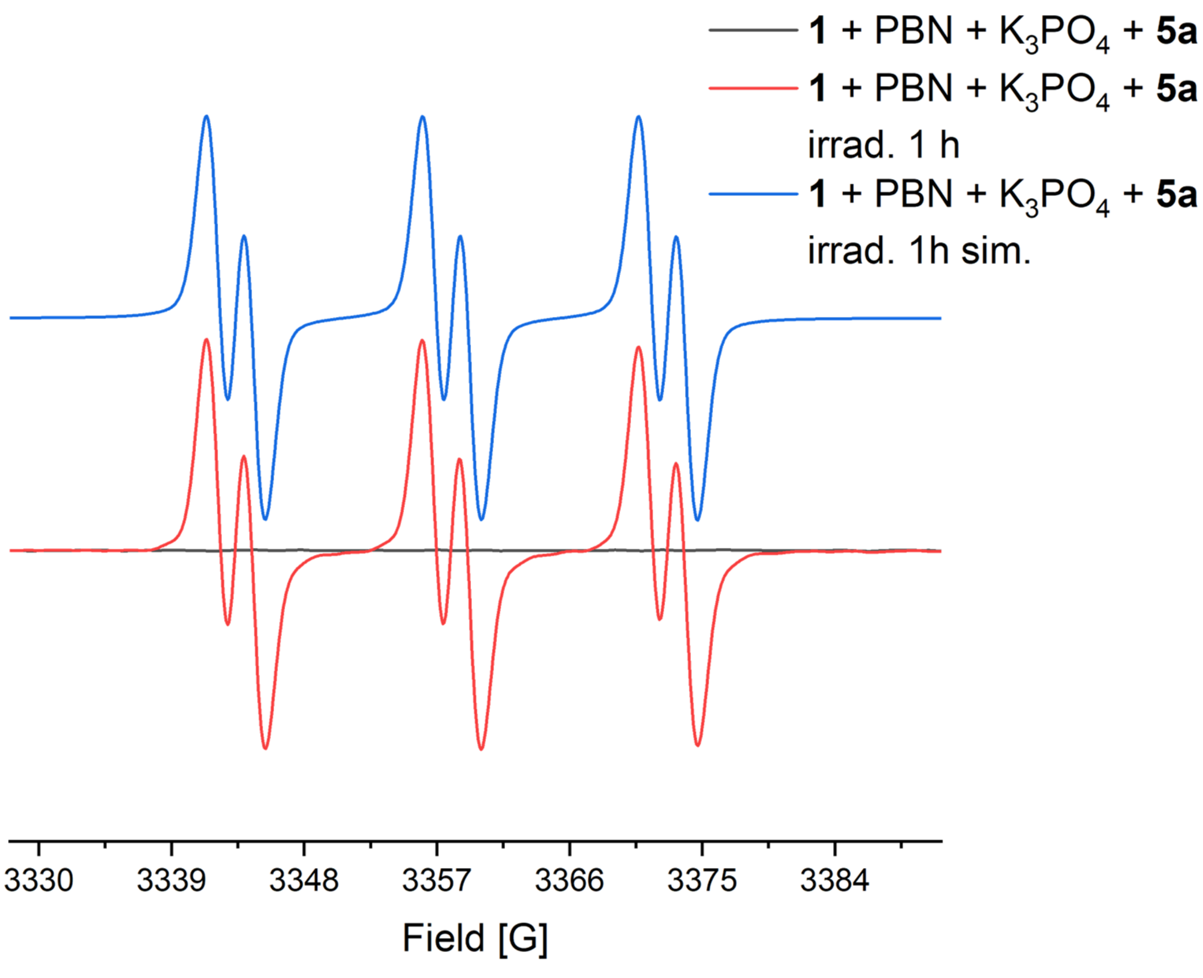


**Figure S17**: X-band EPR spectrum (MeCN, 298 K): Experimental signals of **1** mixed with PBN, $K_3PO_4$ and quinoxalinone C in the dark (grey) and after irradiation (red); Simulated signals of product **S4** (blue); Fitting Parameters: g = 2.00643, a(1x$^{14}$N) = 14.7 G, a(1x$^{1}$H) = 2.3 G.

**Reaction mixture B**:

Quinoxalinone C (8.0 mg, 0.050 mmol, 2.0 equiv.) was added to reaction mixture B. The reaction mixture was stirred under ambient temperature for 1 h in the dark. Afterwards, the flask was transferred back to the nitrogen filled glovebox and an aliquot was taken, diluted with MeCN and analyzed by EPR spectroscopy while maintaining the exclusion of light. The same sample was later transferred to a photoreactor equipped with blue LED strips (wavelength: 460-465 nm) and irradiated at 50% intensity for 1 hours and analyzed by EPR spectroscopy (Scheme **S1**; Figure **S18**).

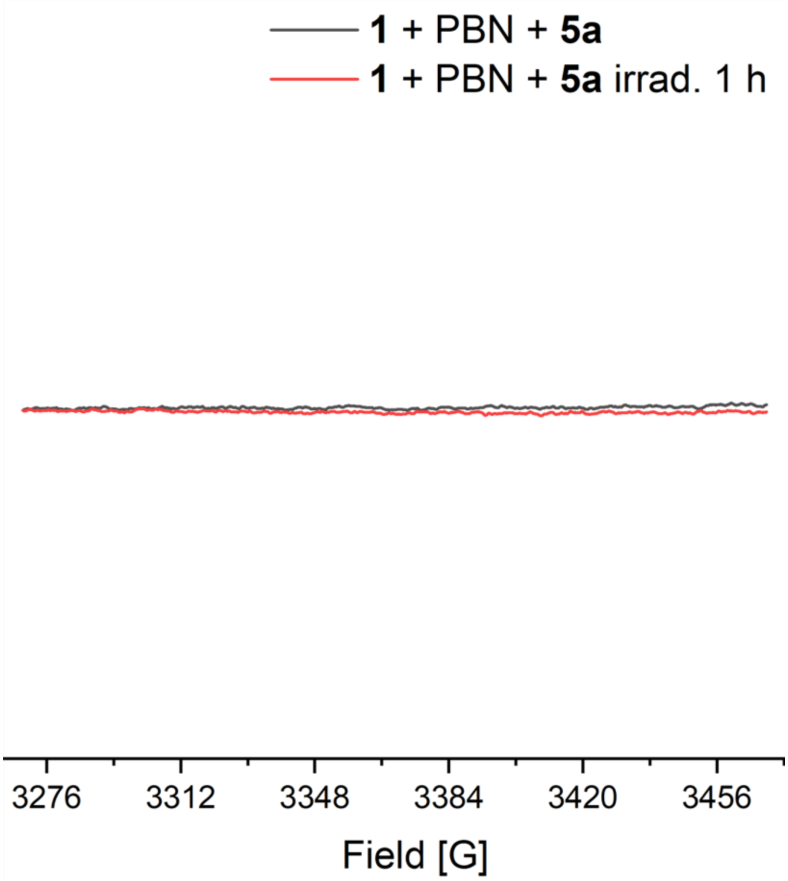


**Figure S18**: X-band EPR spectrum (MeCN, 298 K): Experimental signals of **1** mixed with PBN and quinoxalinone C in the dark (grey) and after irradiation (red).

$K_3PO_4$ (16.2 mg, 0.075 mmol, 3.0 equiv.) was added to reaction mixture B and stirred under ambient temperature for 1 h in the dark. Afterwards, the flask was transferred back to the nitrogen filled glovebox and an aliquot was taken, diluted with MeCN and analyzed by EPR spectroscopy, while maintaining the exclusion of light. The same sample was later transferred to a photoreactor equipped with blue LED strips (wavelength: 460-465 nm) and irradiated at 50% intensity for 1 hours and analyzed by EPR spectroscopy (Scheme **S1**; Figure **S19**).

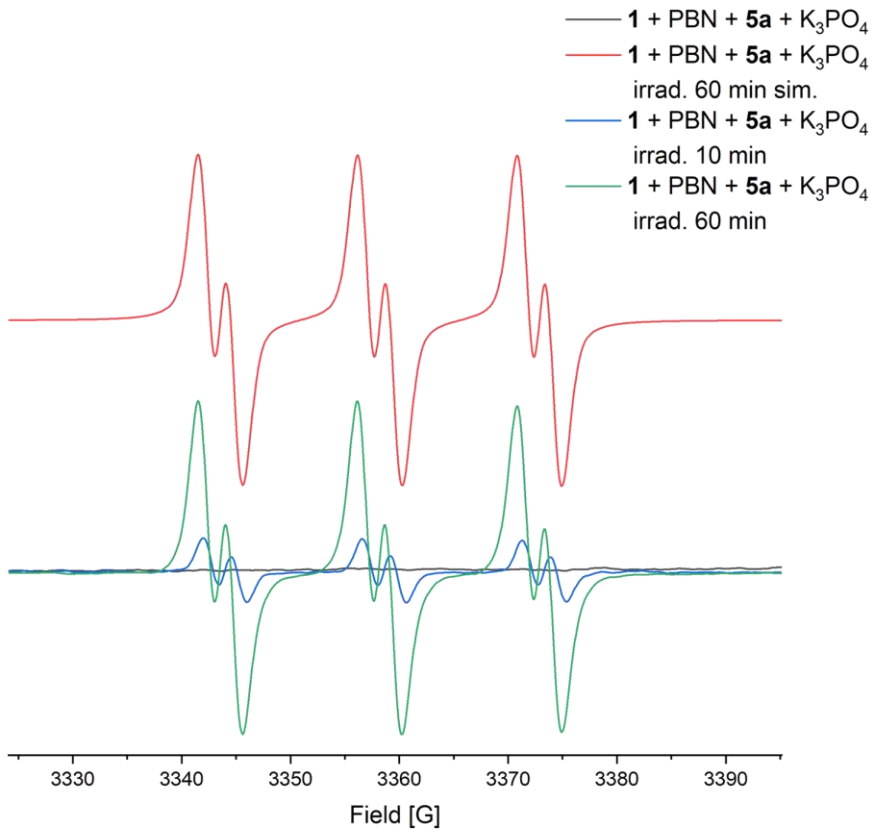


**Figure S19**: X-band EPR spectrum (MeCN, 298 K): Experimental signals of **1** mixed with PBN, quinoxalinone C and $K_3PO_4$ in the dark (grey) and after irradiation (blue and green); Simulated signals of **S4** (red); Fitting Parameters: g = 2.00641, a(1x$^{14}$N) = 14.7 G, a(1x$^{1}$H) = 2.3 G.

# DIFFERENTIAL SCANNING CALORIMETRY (DSC) OF SULFONIUM SALTS

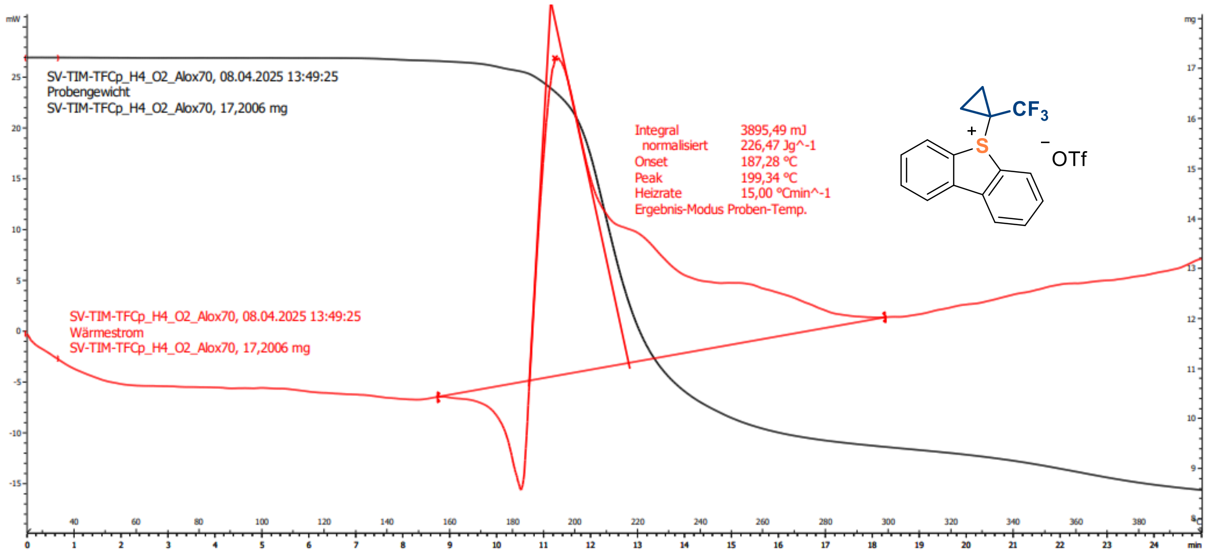


**Figure S20**: DSC measurement sulfonium salt **1** in air.

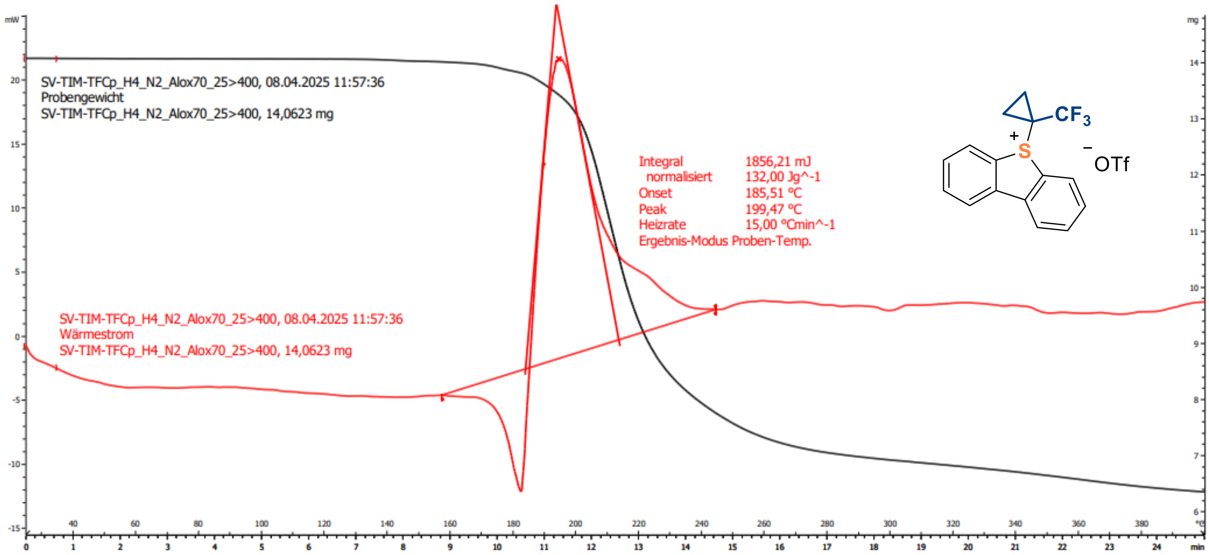


**Figure S21**: DSC measurement sulfonium salt **1** under nitrogen.

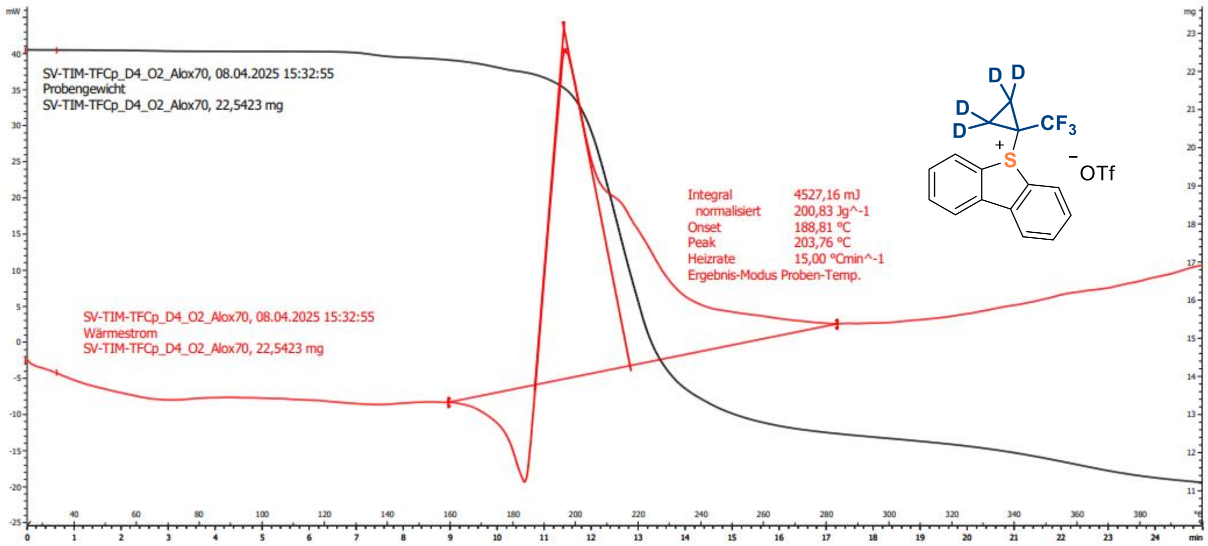


**Figure S22**: DSC measurement sulfonium salt **1-d₄** in air.

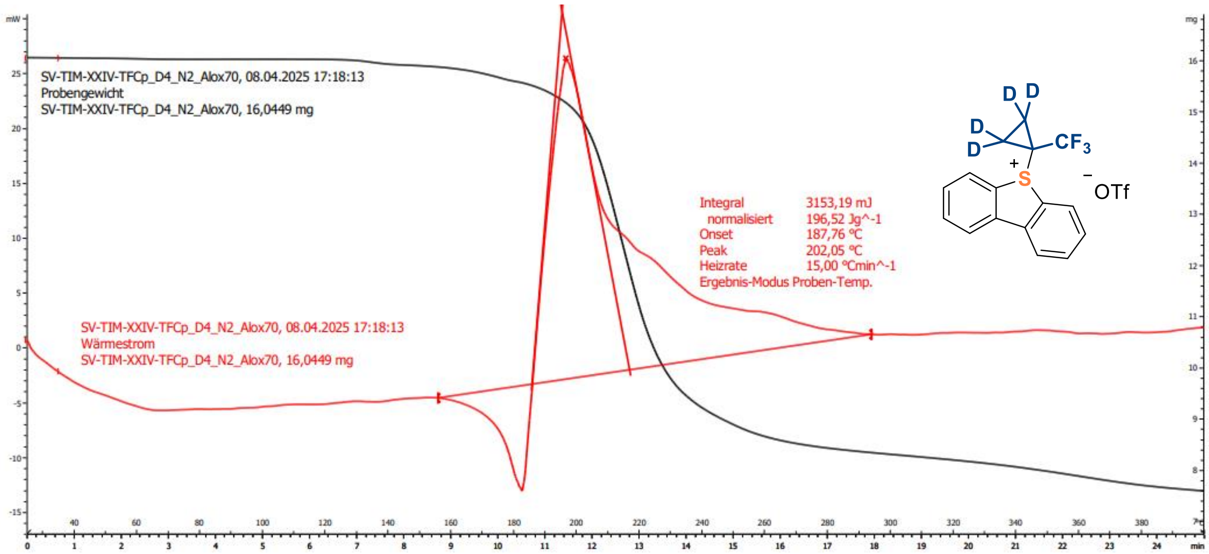


**Figure S23**: DSC measurement sulfonium salt **1-d₄** under nitrogen.

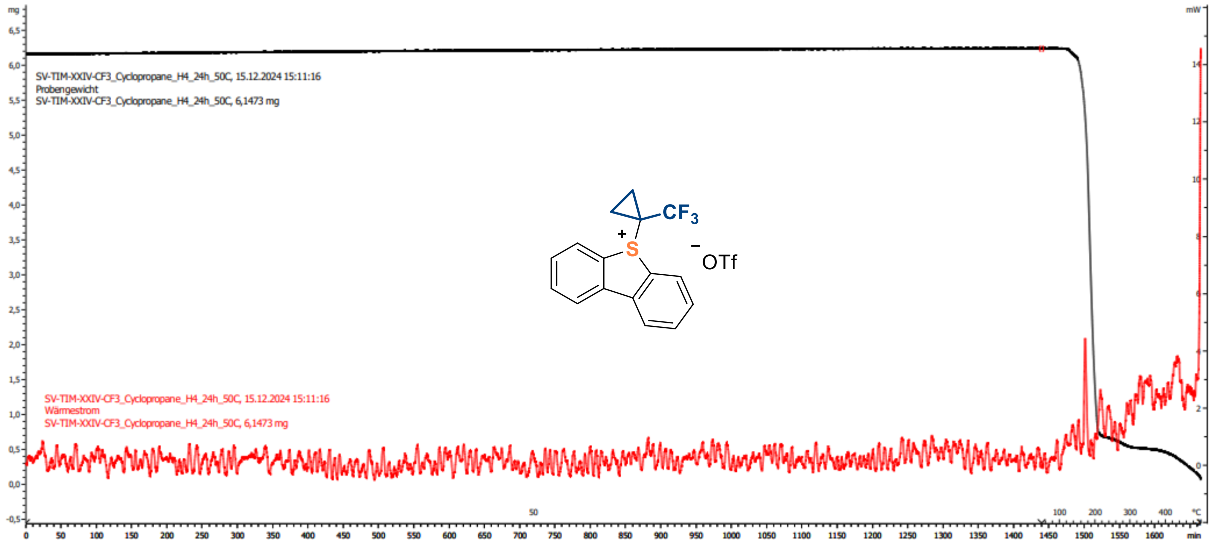


**Figure S24**: Isoage analysis of sulfonium salt **1** at 50 °C under in air.

# X-RAY CRYSTALLOGRAPHIC ANALYSIS

## General

The crystallization conditions are individually stated for each compound. The solvent vapor diffusion method refers to the methodology describes in the literature.[28]

This supplement contains in the following the refinement detail tables, a figure of the complete asymmetric unit and a microscope picture of the crystal used for data collection. The picture of the single crystals was cropped and auto-adjusted for brightness, graduation curve and white balance in Adobe Photoshop (Version 24.1.0). Further crystallographic details can be obtained from the crystallographic information files (CIFs) uploaded to the *Cambridge Crystallographic Data Centre* (CCDC), where they can be obtained free of charge.

**Table S4**: CCDC deposition numbers.

| Identifier | CCDC number | Identifier | CCDC number |
|---|---|---|---|
| **1** | 2427498 | **6d** | 2427503 |
| **4a** | 2427500 | **6h** | 2427504 |
| **4t** | 2427501 | **6m** | 2427505 |
| **6b** | 2427502 | **6aa** | 2427499 |

Refinement details for **1**

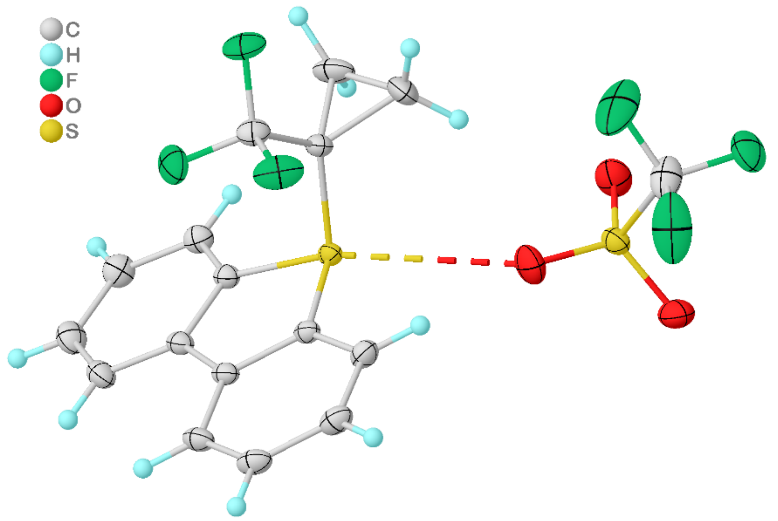


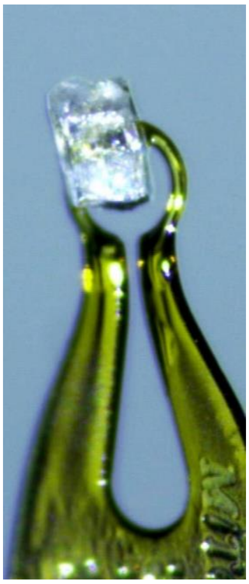

**Figure S25**: Full asymmetric unit of **1**. Anisotropic displacement ellipsoids drawn at 50% probability level. The contact between cationic sulfur and anionic triflate is drawn as stippled bond. Single crystals were obtained from acetonitrile and diethyl ether mixtures by solvent vapor diffusion method.

| | |
|---|---|
| CCDC number | 2427498 |
| Empirical formula | $C_{17}H_{12}F_6O_3S_2$ |
| Formula weight | 442.39 |
| Temperature [K] | 100.00 |
| Crystal system | Monoclinic |
| Space group (number) | $P2_1/n$ (14) |
| *a* [Å] | 10.0614(4) |
| *b* [Å] | 13.5333(9) |
| *c* [Å] | 13.7882(9) |
| α [°] | 90 |
| β [°] | 107.714(2) |
| γ [°] | 90 |
| Volume [Å$^3$] | 1788.44(18) |
| *Z* | 4 |
| $\rho_{calc}$ [gcm$^{-3}$] | 1.643 |
| $\mu$ [mm$^{-1}$] | 0.374 |
| *F*(000) | 896 |
| Crystal size [mm$^3$] | 0.296×0.18×0.163 |
| Crystal color | Colorless |
| Crystal shape | Block |
| Radiation | Mo$K_\alpha$ (λ=0.71073 Å) |
| 2θ range [°] | 4.32 to 65.18 (0.66 Å) |

| | |
|---|---|
| Index ranges | −15 ≤ h ≤ 12<br>−20 ≤ k ≤ 20<br>−20 ≤ l ≤ 20 |
| Reflections collected | 94981 |
| Independent reflections | 6519<br>$R_{int}$ = 0.0213<br>$R_{sigma}$ = 0.0088 |
| Completeness to θ = 25.242° | 100.0 % |
| Data / Restraints / Parameters | 6519/0/253 |
| Absorption correction $T_{min}/T_{max}$ (method) | 0.9147/0.9715 (numerical) |
| Goodness-of-fit on $F^2$ | 1.048 |
| Final *R* indexes [$I \geq 2\sigma(I)$] | $R_1$ = 0.0331<br>w$R_2$ = 0.0940 |
| Final *R* indexes [all data] | $R_1$ = 0.0349<br>w$R_2$ = 0.0958 |
| Largest peak/hole [eÅ$^{-3}$] | 0.95/−0.48 |

Refinement details for **4a**

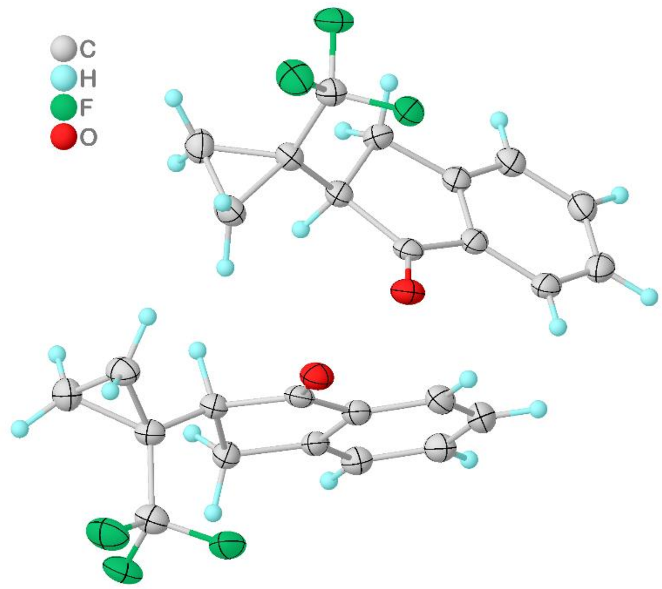


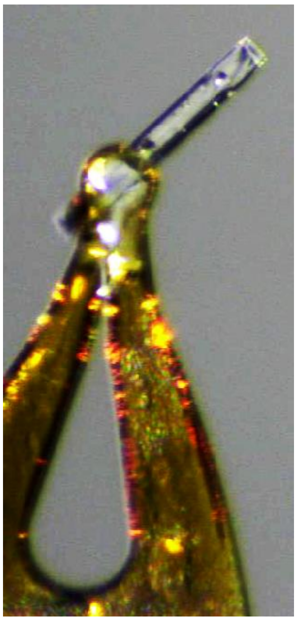

**Figure S26**: Full asymmetric unit of **4a** (Z‘=2). Anisotropic displacement ellipsoids drawn at 50% probability level. Single crystals were obtained from chloroform (NMR tube).

| | |
|---|---|
| CCDC number | 2427500 |
| Empirical formula | $C_{13}H_{11}F_3O$ |
| Formula weight | 240.22 |
| Temperature [K] | 100.00 |
| Crystal system | Monoclinic |
| Space group (number) | $P2_1/n$ (14) |
| *a* [Å] | 5.379(4) |
| *b* [Å] | 20.079(16) |
| *c* [Å] | 19.946(16) |
| α [°] | 90 |
| β [°] | 92.176(19) |
| γ [°] | 90 |
| Volume [Å$^3$] | 2153(3) |
| *Z* | 8 |
| $\rho_{calc}$ [gcm$^{-3}$] | 1.482 |
| $\mu$ [mm$^{-1}$] | 0.128 |
| *F*(000) | 992 |
| Crystal size [mm$^3$] | 0.035×0.037×0.645 |
| Crystal color | Colorless |
| Crystal shape | Needle |

| | |
|---|---|
| Radiation | Mo$K_\alpha$ (λ=0.71073 Å) |
| 2θ range [°] | 4.54 to 55.79 (0.76 Å) |
| Index ranges | −7 ≤ h ≤ 7<br>−25 ≤ k ≤ 26<br>−26 ≤ l ≤ 26 |
| Reflections collected | 35791 |
| Independent reflections | 5113<br>$R_{int}$ = 0.0915<br>$R_{sigma}$ = 0.0796 |
| Completeness to θ = 25.242° | 99.9 % |
| Data / Restraints / Parameters | 5113 / 0 / 307 |
| Absorption correction $T_{min}/T_{max}$ (method) | 0.6975 / 1.0000 (numerical) |
| Goodness-of-fit on $F^2$ | 1.032 |
| Final *R* indexes [*I*≥2σ(*I*)] | $R_1$ = 0.0576<br>w$R_2$ = 0.1415 |
| Final *R* indexes [all data] | $R_1$ = 0.0952<br>w$R_2$ = 0.1650 |
| Largest peak/hole [eÅ$^{-3}$] | 0.30/−0.36 |

Refinement details for **4t**

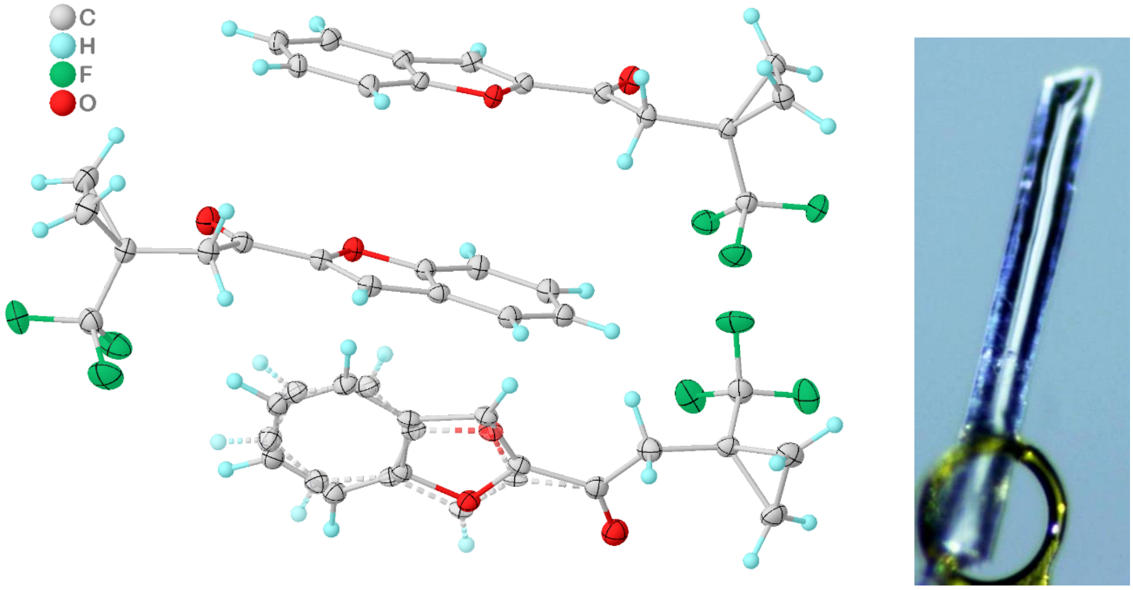


**Figure S27:** Full asymmetric unit of **4t** (Z‘=3). The third independent molecule features a disorder of the benzofuran moiety with a refined occupancy ratio of 0.786(4):0.214(4). Anisotropic displacement ellipsoids drawn at 50% probability level; minor disorder part is drawn translucent with stippled bonds. Single crystals were obtained from chloroform (NMR tube).

| | |
|---|---|
| CCDC number | 2427501 |
| Empirical formula | $C_{14}H_{11}F_3O_2$ |
| Formula weight | 268.23 |
| Temperature [K] | 100.00 |
| Crystal system | Triclinic |
| Space group (number) | $P\bar{1}$ (2) |
| $a$ [Å] | 5.5105(2) |
| $b$ [Å] | 11.3496(5) |
| $c$ [Å] | 28.9545(13) |
| α [°] | 96.938(2) |
| β [°] | 95.4390(10) |
| γ [°] | 93.3970(10) |
| Volume [Å$^3$] | 1784.93(13) |
| $Z$ | 6 |
| $\rho_{calc}$ [gcm$^{-3}$] | 1.497 |
| $\mu$ [mm$^{-1}$] | 0.130 |
| $F$(000) | 828 |
| Crystal size [mm$^3$] | 0.101×0.11×1.717 |
| Crystal color | Colorless |
| Crystal shape | Needle |
| Radiation | Mo$K_\alpha$ (λ=0.71073 Å) |

| | |
|---|---|
| 2θ range [°] | 3.72 to 65.20 (0.66 Å) |
| Index ranges | −6 ≤ h ≤ 8<br>−17 ≤ k ≤ 17<br>−43 ≤ l ≤ 43 |
| Reflections collected | 110960 |
| Independent reflections | 12989<br>$R_{int}$ = 0.0283<br>$R_{sigma}$ = 0.0168 |
| Completeness to θ = 25.242° | 100.0 % |
| Data / Restraints / Parameters | 12989 / 157 / 596 |
| Absorption correction $T_{min}/T_{max}$ (method) | 0.9161 / 1.0000 (numerical) |
| Goodness-of-fit on $F^2$ | 1.036 |
| Final $R$ indexes [$I$≥2σ($I$)] | $R_1$ = 0.0365<br>w$R_2$ = 0.0905 |
| Final $R$ indexes [all data] | $R_1$ = 0.0468<br>w$R_2$ = 0.0981 |
| Largest peak/hole [eÅ$^{-3}$] | 0.45/−0.29 |

Refinement details for **6b**

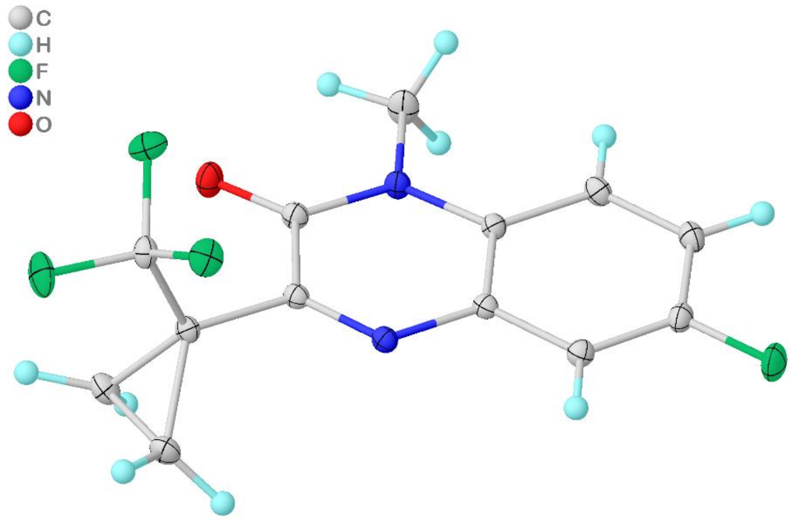


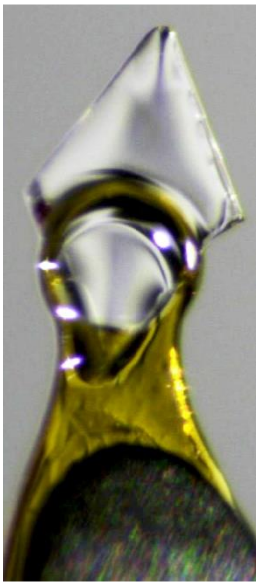

**Figure S28:** Full asymmetric unit of **6b**. Anisotropic displacement ellipsoids drawn at 50% probability level. Single crystals were obtained from a mixture of ethyl acetate and hexane by solvent vapor diffusion method.

| | |
|---|---|
| CCDC number | 2427502 |
| Empirical formula | $C_{13}H_{10}F_4N_2O$ |
| Formula weight | 286.23 |
| Temperature [K] | 100.00 |
| Crystal system | Triclinic |
| Space group (number) | $P\overline{1}$ (2) |
| $a$ [Å] | 6.6351(6) |
| $b$ [Å] | 9.3881(11) |
| $c$ [Å] | 9.9617(10) |
| α [°] | 88.880(4) |
| β [°] | 71.628(3) |
| γ [°] | 82.074(4) |
| Volume [Å$^3$] | 583.08(10) |
| $Z$ | 2 |
| $\rho_{calc}$ [gcm$^{-3}$] | 1.630 |
| $\mu$ [mm$^{-1}$] | 0.149 |
| $F$(000) | 292 |
| Crystal size [mm$^3$] | 0.753×0.568×0.104 |
| Crystal colour | Colorless |
| Crystal shape | Plate |
| Radiation | Mo$K_\alpha$ (λ=0.71073 Å) |
| 2θ range [°] | 4.31 to 61.10 (0.70 Å) |

| | |
|---|---|
| Index ranges | −9 ≤ h ≤ 8<br>−13 ≤ k ≤ 13<br>−14 ≤ l ≤ 14 |
| Reflections collected | 25458 |
| Independent reflections | 3556<br>$R_{int}$ = 0.0349<br>$R_{sigma}$ = 0.0193 |
| Completeness to θ = 25.242° | 100.0 % |
| Data / Restraints / Parameters | 3556/0/182 |
| Absorption correction $T_{min}/T_{max}$ (method) | 0.8203/1.0000 (numerical) |
| Goodness-of-fit on $F^2$ | 1.041 |
| Final $R$ indexes [$I$≥2σ($I$)] | $R_1$ = 0.0357<br>w$R_2$ = 0.1014 |
| Final $R$ indexes [all data] | $R_1$ = 0.0386<br>w$R_2$ = 0.1045 |
| Largest peak/hole [eÅ$^{-3}$] | 0.51/−0.36 |

Refinement details for **6d**

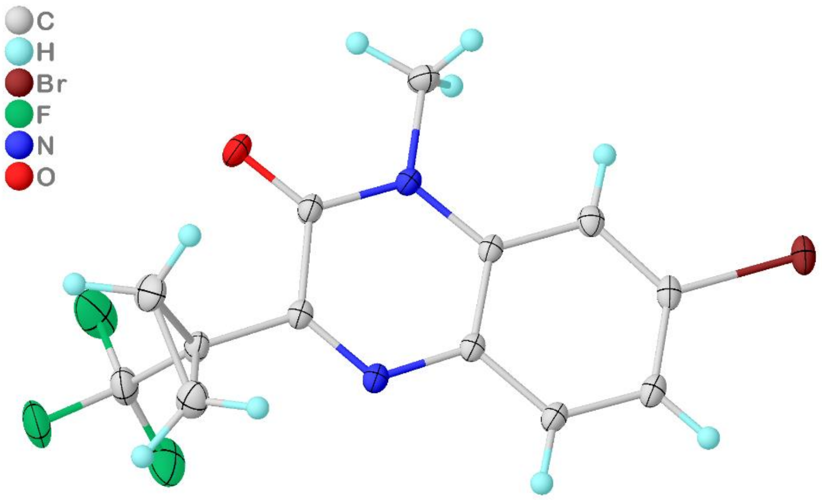


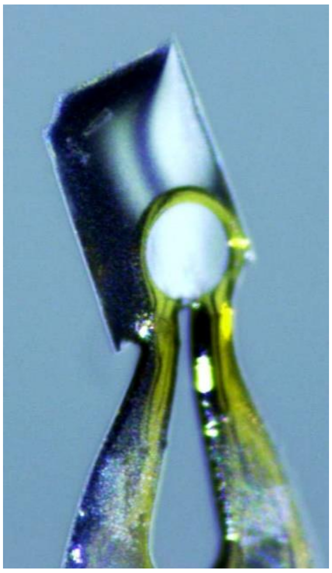

**Figure S29:** Full asymmetric unit of **6d**. Anisotropic displacement ellipsoids drawn at 50% probability level. Single crystals were obtained from a mixture of ethyl acetate and hexane by solvent vapor diffusion method.

| | |
|---|---|
| CCDC number | 2427503 |
| Empirical formula | $C_{13}H_{10}BrF_3N_2O$ |
| Formula weight | 347.14 |
| Temperature [K] | 100.00 |
| Crystal system | Monoclinic |
| Space group (number) | $P2_1/n$ (14) |
| *a* [Å] | 9.436(2) |
| *b* [Å] | 6.8668(15) |
| *c* [Å] | 19.858(4) |
| α [°] | 90 |
| β [°] | 98.071(6) |
| γ [°] | 90 |
| Volume [$Å^3$] | 1274.0(5) |
| *Z* | 4 |
| $\rho_{calc}$ [$gcm^{-3}$] | 1.810 |
| $\mu$ [$mm^{-1}$] | 3.260 |
| *F*(000) | 688 |
| Crystal size [$mm^3$] | 0.691×0.393×0.038 |
| Crystal color | Colorless |
| Crystal shape | plate |
| Radiation | Mo$K_\alpha$ (λ=0.71073 Å) |
| 2θ range [°] | 4.14 to 65.19 (0.66 Å) |

| | |
|---|---|
| Index ranges | $-14 \leq h \leq 14$<br>$-8 \leq k \leq 10$<br>$-30 \leq l \leq 29$ |
| Reflections collected | 46564 |
| Independent reflections | 4640<br>$R_{int}$ = 0.0289<br>$R_{sigma}$ = 0.0170 |
| Completeness to θ = 25.242° | 100.0 % |
| Data / Restraints / Parameters | 4640/0/182 |
| Absorption correction $T_{min}/T_{max}$ (method) | 0.3747/0.9306 (multi-scan) |
| Goodness-of-fit on $F^2$ | 1.063 |
| Final *R* indexes [$I \geq 2\sigma(I)$] | $R_1$ = 0.0245<br>$wR_2$ = 0.0583 |
| Final *R* indexes [all data] | $R_1$ = 0.0309<br>$wR_2$ = 0.0612 |
| Largest peak/hole [$eÅ^{-3}$] | 0.57/−0.74 |

Refinement details for **6h**

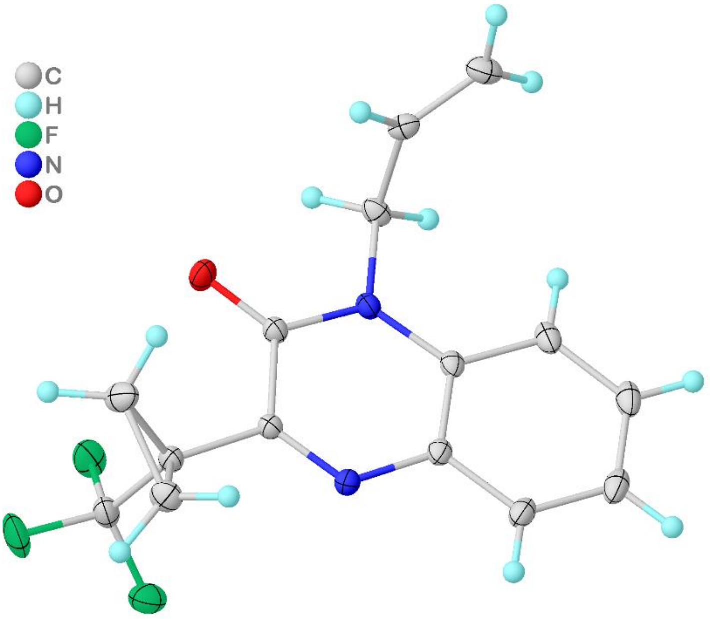


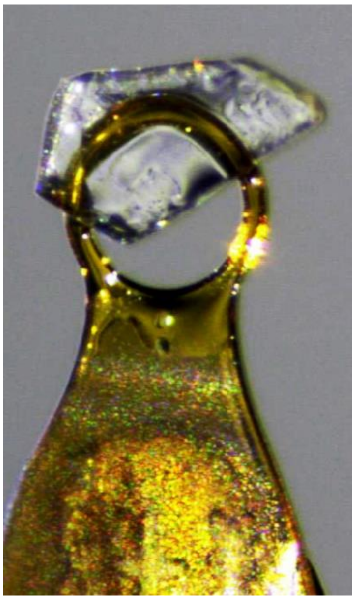

**Figure S30:** Full asymmetric unit of **6h**. Anisotropic displacement ellipsoids drawn at 50% probability level. Single crystals were obtained from a mixture of ethyl acetate and hexane by solvent vapor diffusion method.

| | |
|---|---|
| CCDC number | 2427504 |
| Empirical formula | $C_{15}H_{13}F_3N_2O$ |
| Formula weight | 294.27 |
| Temperature [K] | 100.00 |
| Crystal system | Orthorhombic |
| Space group (number) | $Pbca$ (61) |
| $a$ [Å] | 13.6528(6) |
| $b$ [Å] | 7.3022(4) |
| $c$ [Å] | 26.7572(11) |
| α [°] | 90 |
| β [°] | 90 |
| γ [°] | 90 |
| Volume [Å$^3$] | 2667.6(2) |
| $Z$ | 8 |
| $\rho_{calc}$ [gcm$^{-3}$] | 1.465 |
| $\mu$ [mm$^{-1}$] | 0.122 |
| $F$(000) | 1216 |
| Crystal size [mm$^3$] | 0.583×0.291×0.11 |
| Crystal color | Colorless |
| Crystal shape | Plate |
| Radiation | Mo$K_\alpha$ (λ=0.71073 Å) |
| 2θ range [°] | 4.26 to 65.20 (0.66 Å) |

| | |
|---|---|
| Index ranges | −20 ≤ h ≤ 20<br>−11 ≤ k ≤ 11<br>−40 ≤ l ≤ 39 |
| Reflections collected | 78725 |
| Independent reflections | 4858<br>$R_{int}$ = 0.0426<br>$R_{sigma}$ = 0.0147 |
| Completeness to θ = 25.242° | 100.0 % |
| Data / Restraints / Parameters | 4858/0/198 |
| Absorption correction $T_{min}/T_{max}$ (method) | 0.7849/1.0000 (numerical) |
| Goodness-of-fit on $F^2$ | 1.063 |
| Final $R$ indexes [$I$≥2σ($I$)] | $R_1$ = 0.0373<br>w$R_2$ = 0.1028 |
| Final $R$ indexes [all data] | $R_1$ = 0.0424<br>w$R_2$ = 0.1075 |
| Largest peak/hole [eÅ$^{-3}$] | 0.53/−0.26 |

Refinement details for **6m**

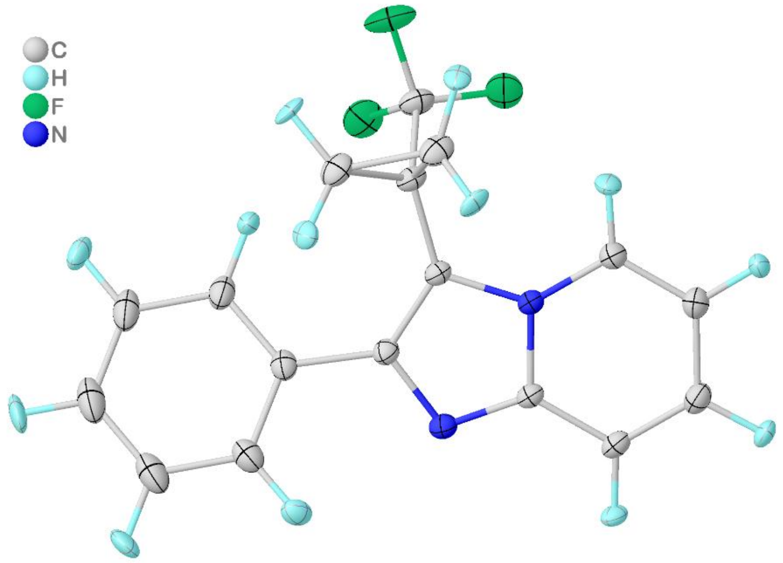


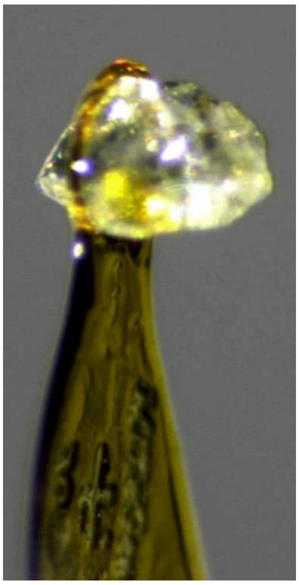

**Figure S31:** Full asymmetric unit of **6m**. Fluorine atoms were refined with anharmonic corrections using the 3rd and 4th order Gram-Charlier formalism. Aspherical atomic form factors used for refinement are based on calculated in NoSpherA2 at R2SCAN/x2c-TZVPP level of theory. Anisotropic displacement ellipsoids drawn at 50% probability level. Single crystals were obtained from chloroform (NMR tube).

| | |
|---|---|
| CCDC number | 2427505 |
| Empirical formula | $C_{17}H_{13}F_3N_2$ |
| Formula weight | 302.301 |
| Temperature [K] | 100.00 |
| Crystal system | Monoclinic |
| Space group (number) | $P2_1/c$ (14) |
| *a* [Å] | 6.8796(6) |
| *b* [Å] | 14.5463(11) |
| *c* [Å] | 14.3107(14) |
| α [°] | 90 |
| β [°] | 91.356(3) |
| γ [°] | 90 |
| Volume [Å$^3$] | 1431.7(2) |
| *Z* | 4 |
| $\rho_{calc}$ [gcm$^{-3}$] | 1.402 |
| $\mu$ [mm$^{-1}$] | 0.111 |
| *F*(000) | 624.502 |
| Crystal size [mm$^3$] | 0.239×0.358×0.43 |
| Crystal color | Colorless |
| Crystal shape | Block |
| Radiation | Mo $K_\alpha$ (λ=0.71073 Å) |

| | |
|---|---|
| 2θ range [°] | 4.00 to 65.30 (0.66 Å) |
| Index ranges | −10 ≤ h ≤ 10<br>−15 ≤ k ≤ 22<br>−21 ≤ l ≤ 21 |
| Reflections collected | 55119 |
| Independent reflections | 5249<br>$R_{int}$ = 0.0399<br>$R_{sigma}$ = 0.0172 |
| Completeness to θ = 25.2417° | 100.0 % |
| Data / Restraints / Parameters | 5249 / 0 / 391 |
| Absorption correction $T_{min}/T_{max}$ (method) | 0.6550 / 0.8499 (multi-scan) |
| Goodness-of-fit on $F^2$ | 1.1041 |
| Final *R* indexes [$I \geq 2\sigma(I)$] | $R_1$ = 0.0209<br>w$R_2$ = 0.0466 |
| Final *R* indexes [all data] | $R_1$ = 0.0265<br>w$R_2$ = 0.0500 |
| Largest peak/hole [eÅ$^{-3}$] | 0.14/−0.15 |

Refinement details for **6aa**

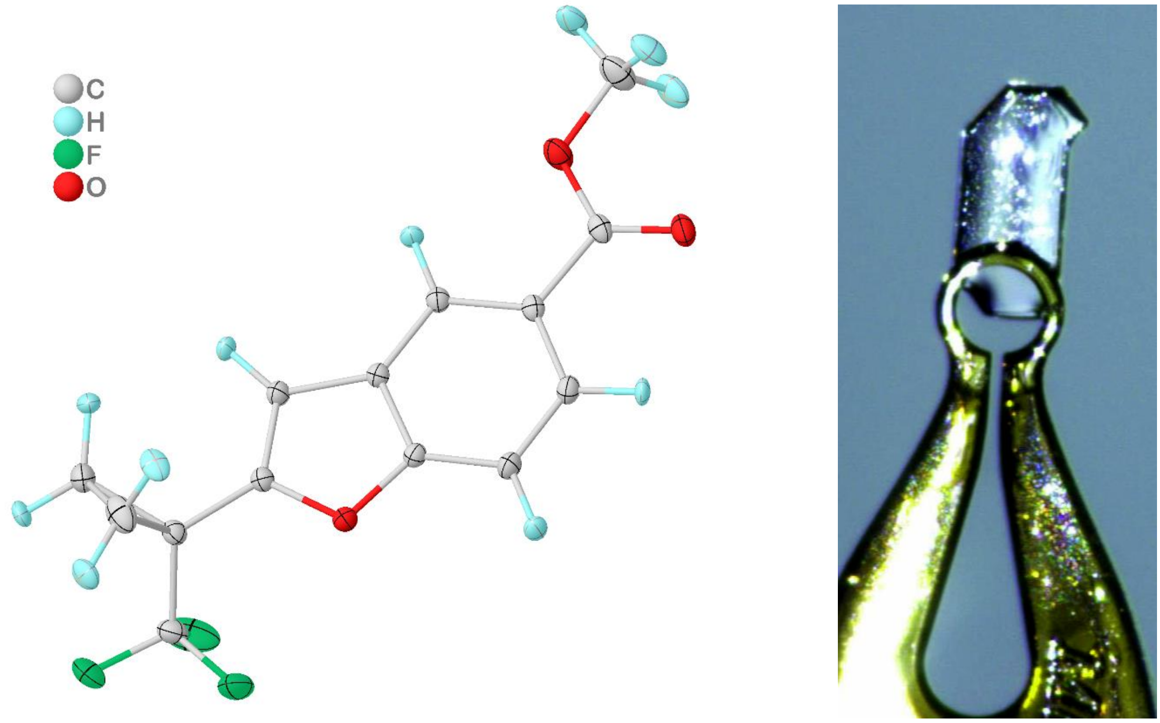


**Figure S32:** Full asymmetric unit of **6aa**. Fluorine atoms were refined with anharmonic corrections using the 3rd and 4th order Gram-Charlier formalism. Aspherical atomic form factors used for refinement are based on calculated in NoSpherA2 at R2SCAN/x2c-TZVPP level of theory. Anisotropic displacement ellipsoids drawn at 50% probability level. Single crystals were obtained from chloroform (NMR tube).

| | |
|---|---|
| CCDC number | 2427499 |
| Empirical formula | $C_{14}H_{11}F_3O_3$ |
| Formula weight | 284.237 |
| Temperature [K] | 100.00 |
| Crystal system | Monoclinic |
| Space group (number) | $P2_1/c$ (14) |
| $a$ [Å] | 14.4613(7) |
| $b$ [Å] | 10.3101(5) |
| $c$ [Å] | 8.3655(4) |
| α [°] | 90 |
| β [°] | 91.105(2) |
| γ [°] | 90 |
| Volume [Å$^3$] | 1247.04(10) |
| $Z$ | 4 |
| $\rho_{calc}$ [gcm$^{-3}$] | 1.514 |
| $\mu$ [mm$^{-1}$] | 0.135 |
| $F$(000) | 584.547 |
| Crystal size [mm$^3$] | 0.042×0.186×0.386 |
| Crystal color | Colorless |
| Crystal shape | plate |

| | |
|---|---|
| Radiation | Mo $K_\alpha$ (λ=0.71073 Å) |
| 2θ range [°] | 4.86 to 84.26 (0.53 Å) |
| Index ranges | −27 ≤ h ≤ 27<br>−19 ≤ k ≤ 19<br>−15 ≤ l ≤ 15 |
| Reflections collected | 128455 |
| Independent reflections | 8782<br>$R_{int}$ = 0.0319<br>$R_{sigma}$ = 0.0144 |
| Completeness to θ = 25.2417° | 100.0 % |
| Data / Restraints / Parameters | 8782 / 0 / 355 |
| Absorption correction $T_{min}/T_{max}$ (method) | 0.7908 / 0.8264 (multi-scan) |
| Goodness-of-fit on $F^2$ | 1.2868 |
| Final $R$ indexes [$I$≥2σ($I$)] | $R_1$ = 0.0222<br>w$R_2$ = 0.0345 |
| Final $R$ indexes [all data] | $R_1$ = 0.0347<br>w$R_2$ = 0.0371 |
| Largest peak/hole [eÅ$^{-3}$] | 0.20/−0.20 |

# SPECTROSCOPIC DATA

Compound **1**:

$^{1}$H NMR (400 MHz, $CD_3CN$)

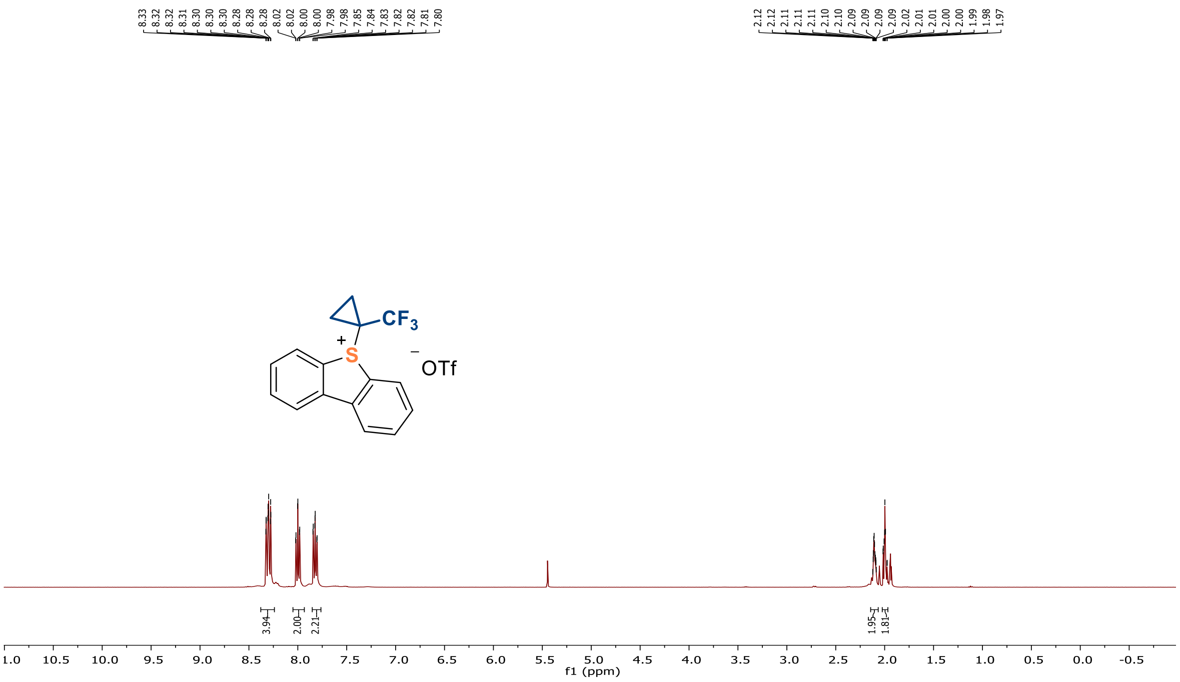


$^{13}$C{1H} NMR (101 MHz, $CD_3CN$)

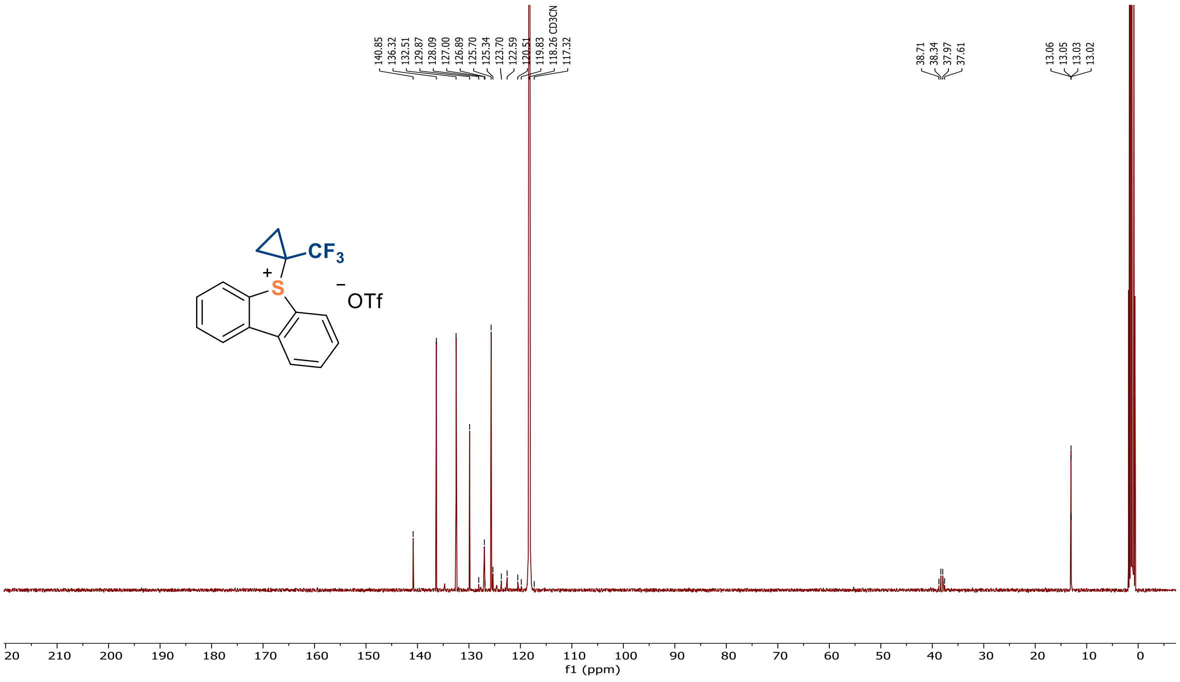

$^{19}F$ NMR (377 MHz, $CD_3CN$)

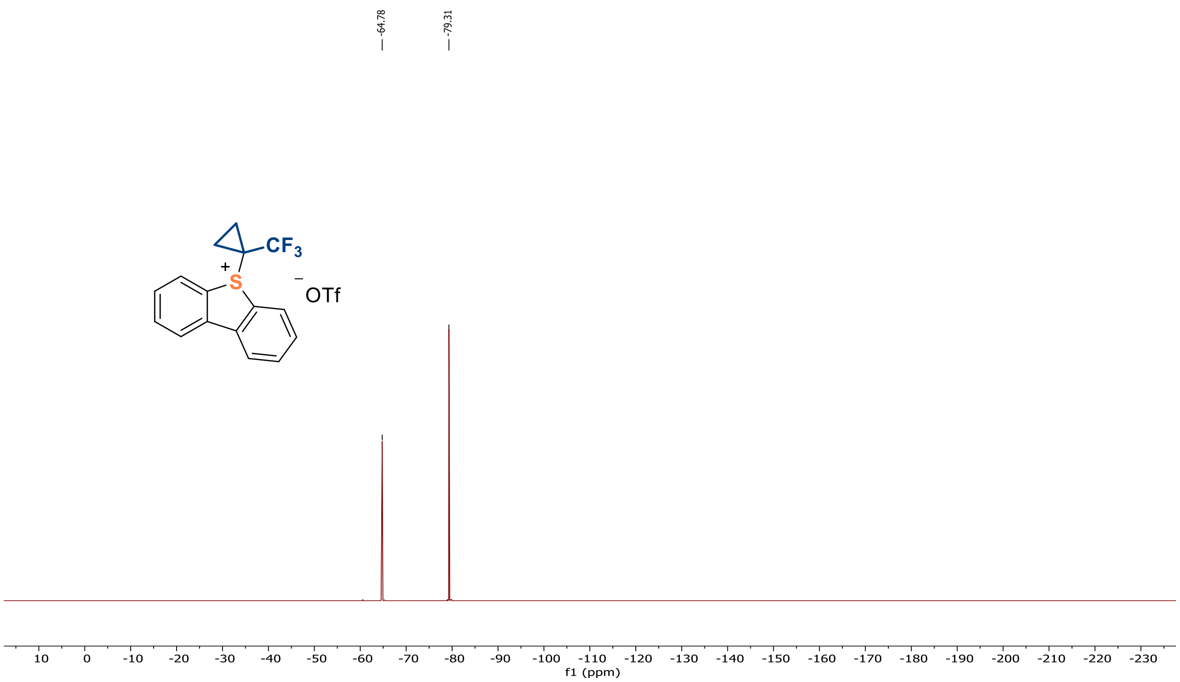


Compound **1-d$_4$**:

$^1H$ NMR (500 MHz, $CD_3CN$)

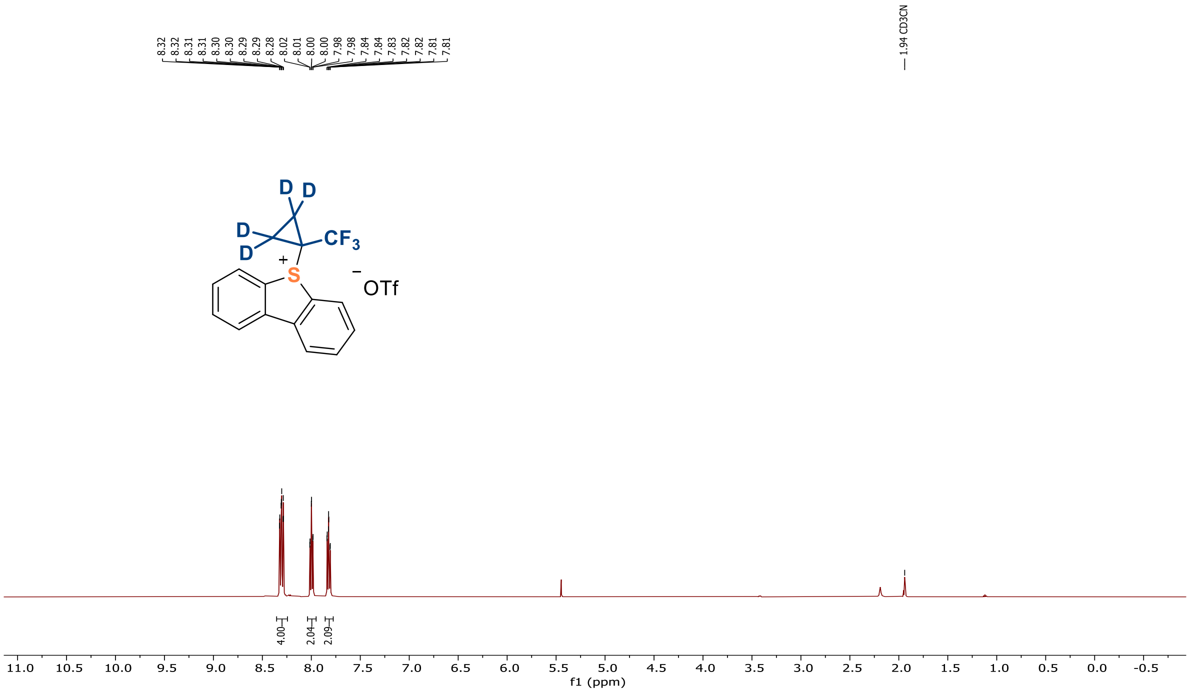

$^{13}C\{1H\}$ NMR (126 MHz, $CD_3CN$)

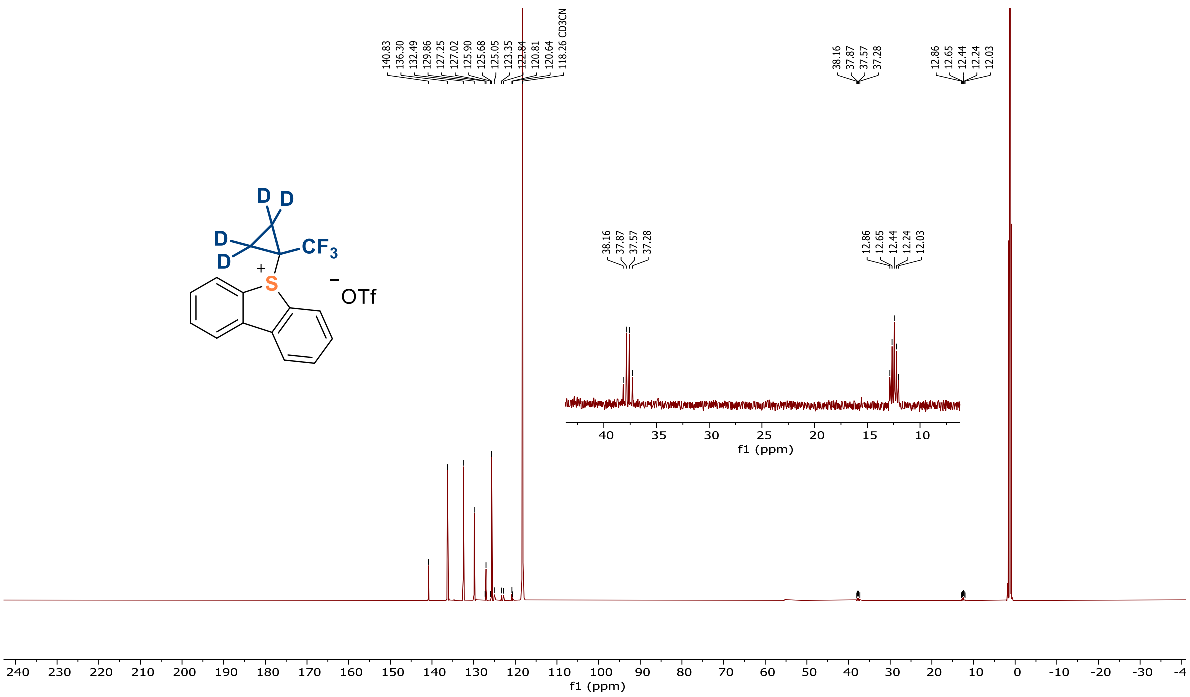

$^{19}F$ NMR (377 MHz, $CD_3CN$)

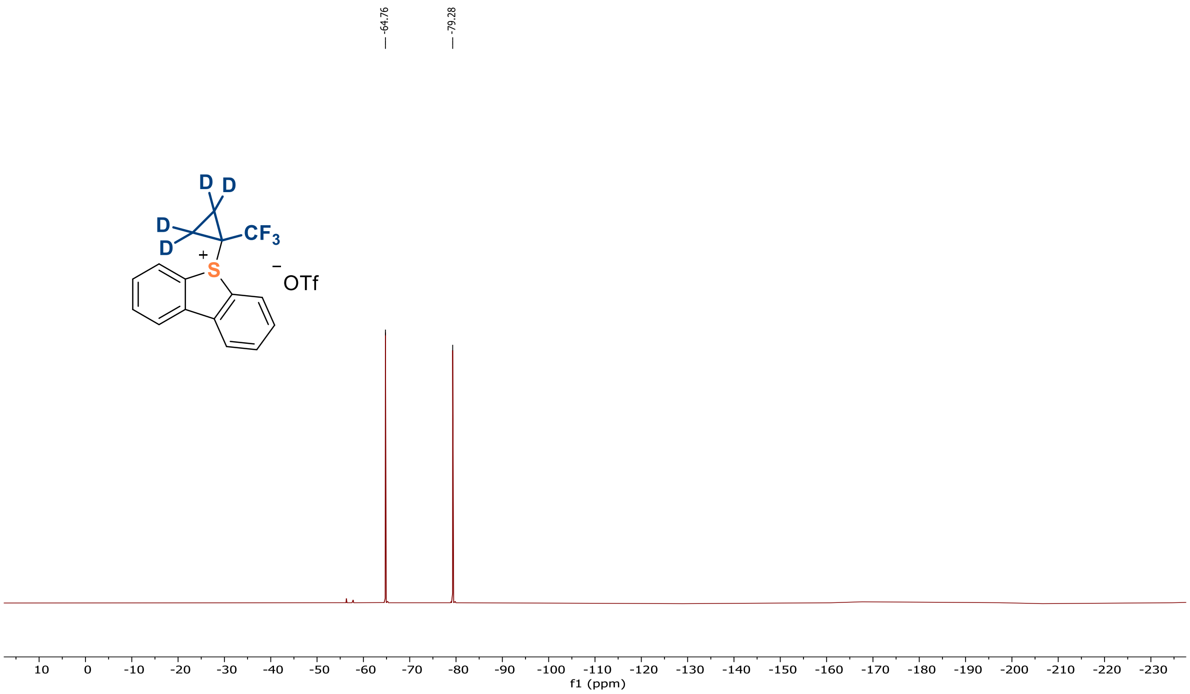

$^{2}H$ NMR (92 MHz, $CH_3CN$)

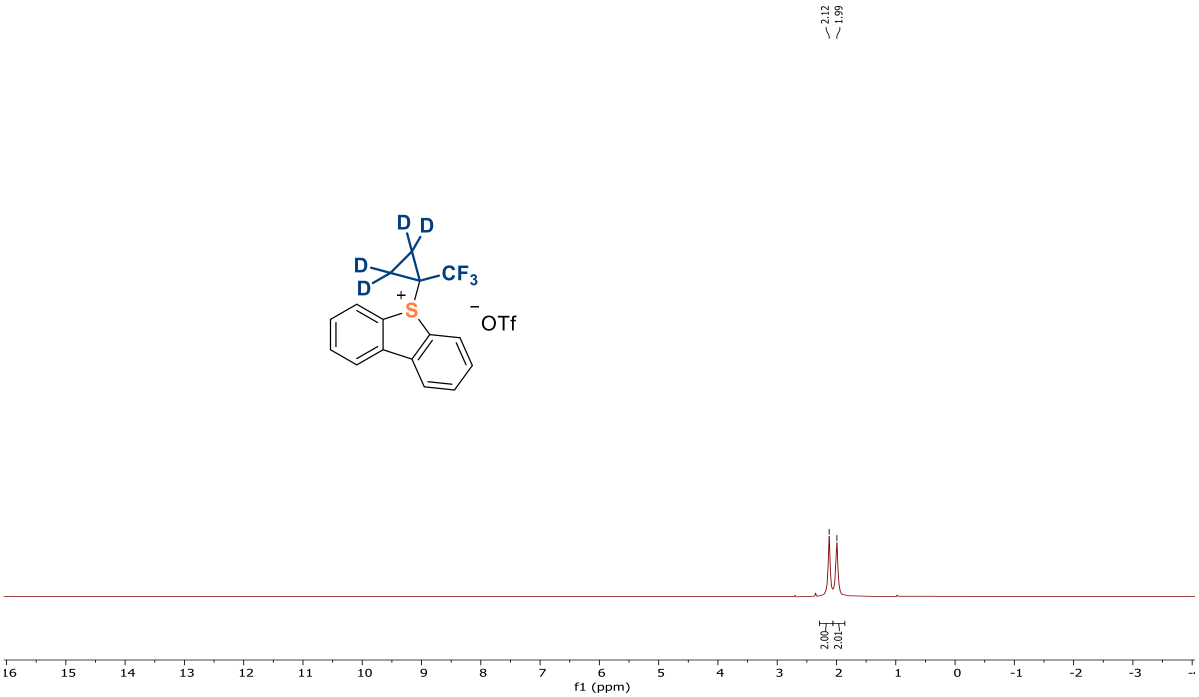


Compound **3d**:

$^{1}H$ NMR (400 MHz, $CDCl_3$)

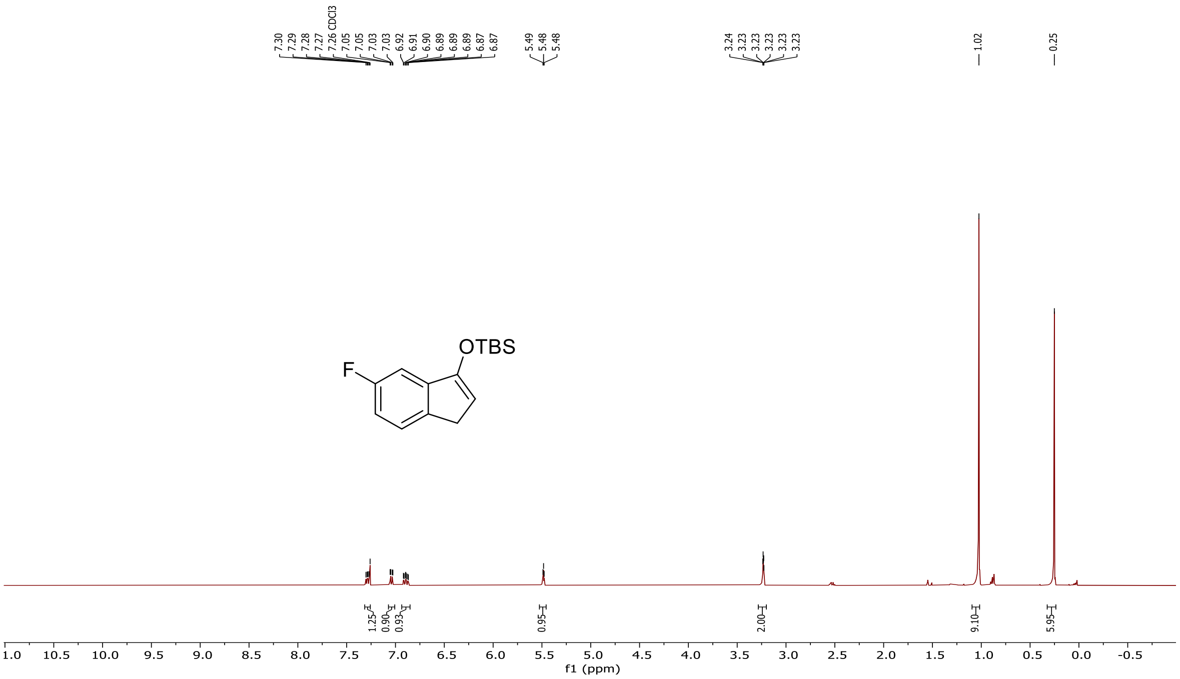

$^{13}C\{1H\}$ NMR (101 MHz, $CDCl_3$)

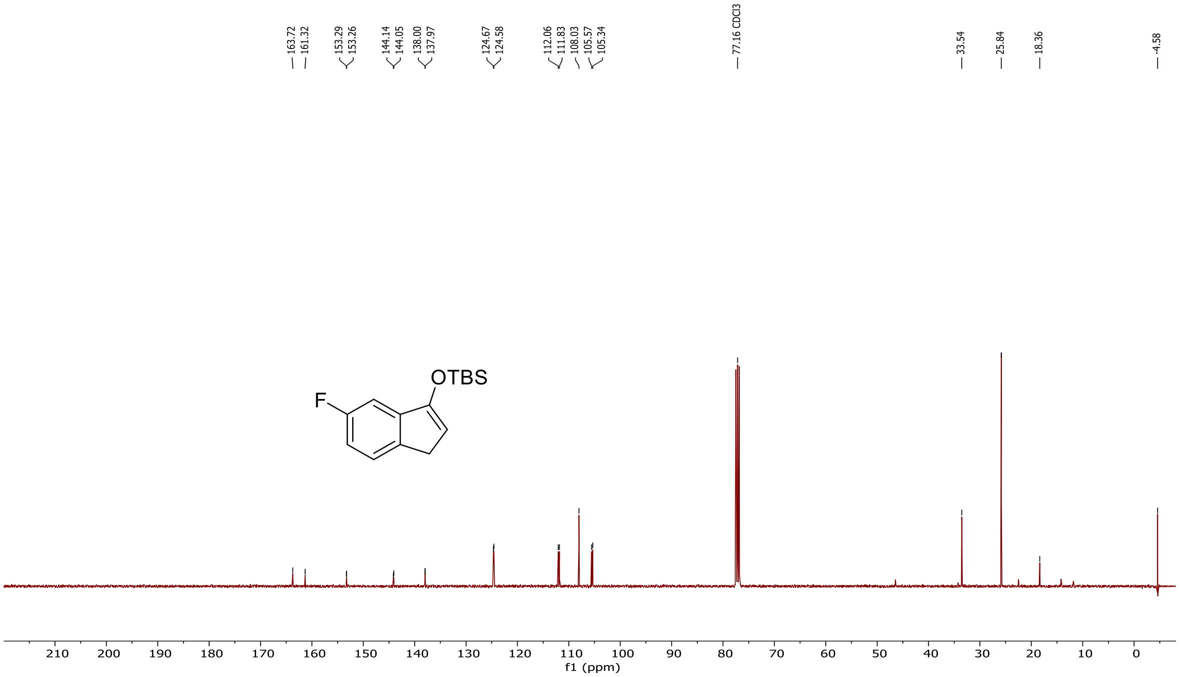


$^{19}F$ NMR (377 MHz, $CDCl_3$)

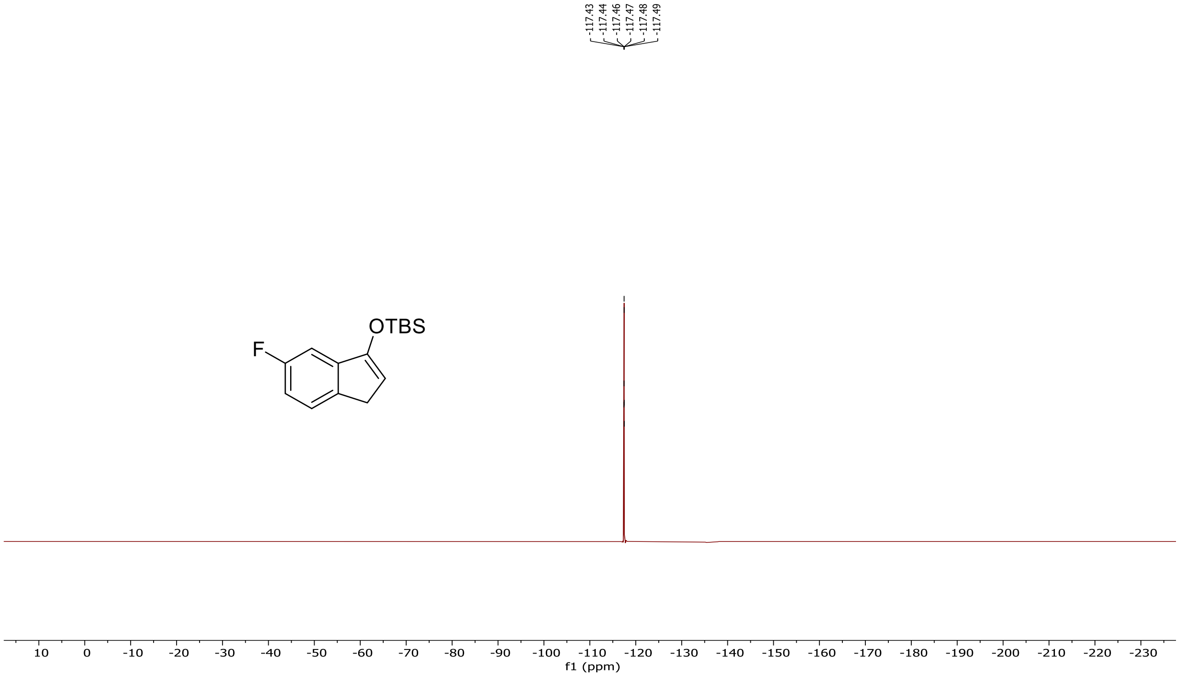

Compound **3e**:

$^{1}H$ NMR (300 MHz, $CDCl_3$)

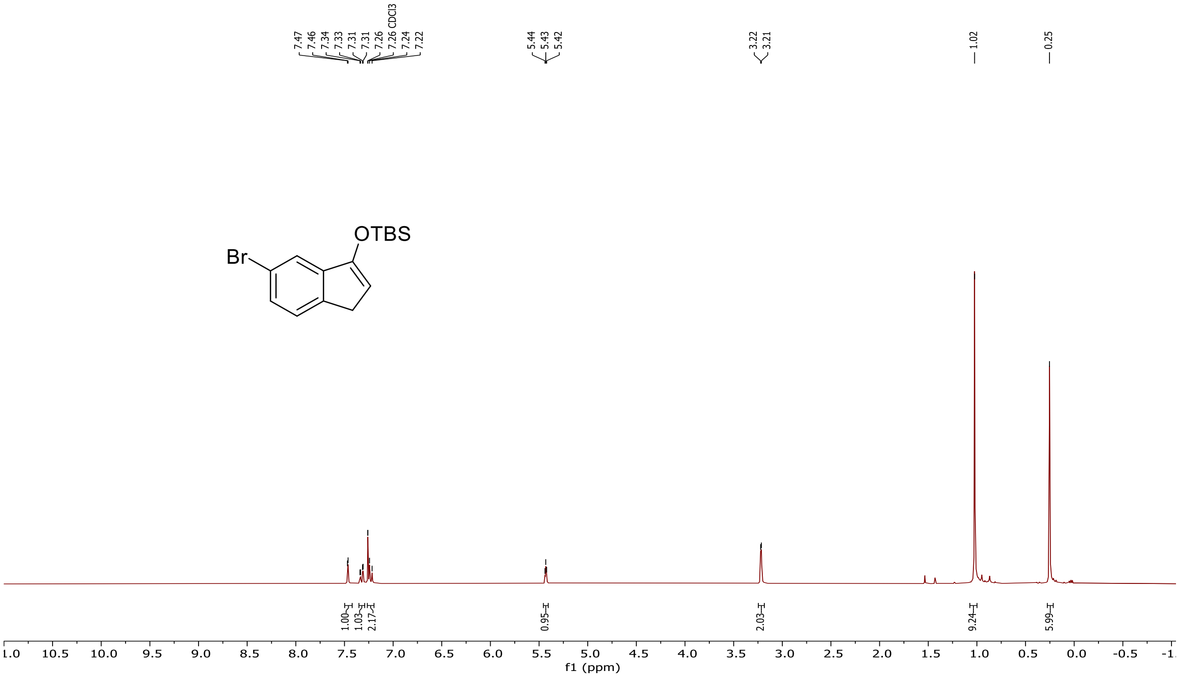


$^{13}C\{1H\}$ NMR (101 MHz, $CDCl_3$)

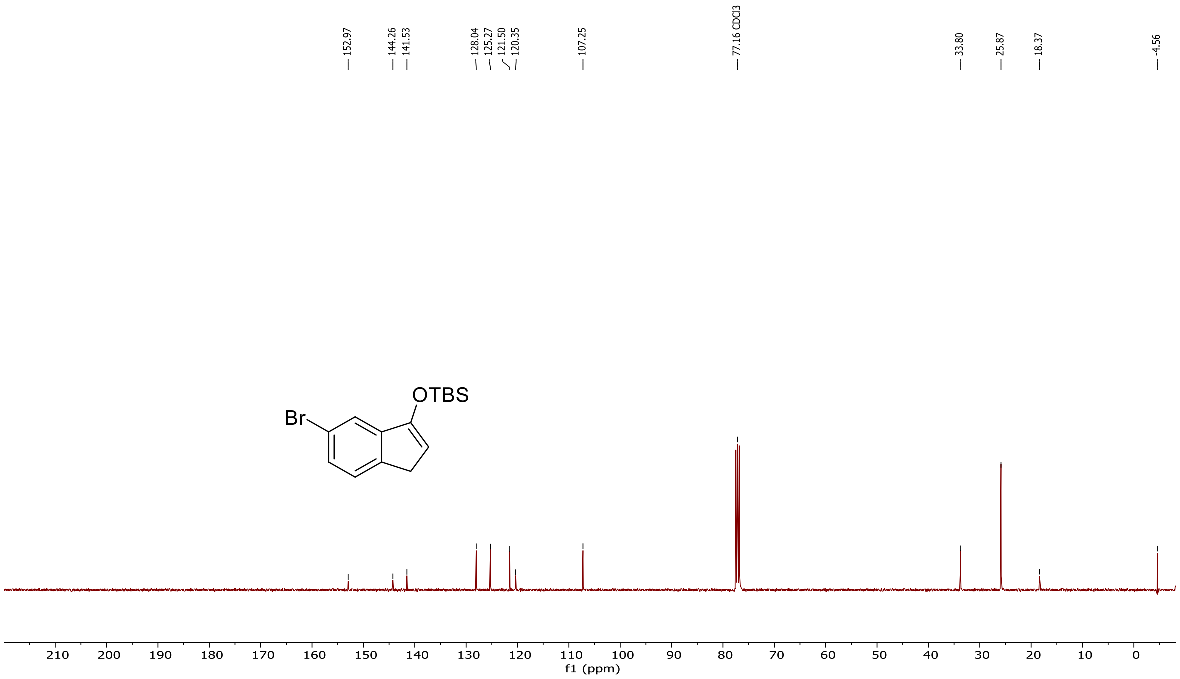

Compound **3f**:

$^{1}H$ NMR (300 MHz, $CDCl_3$)

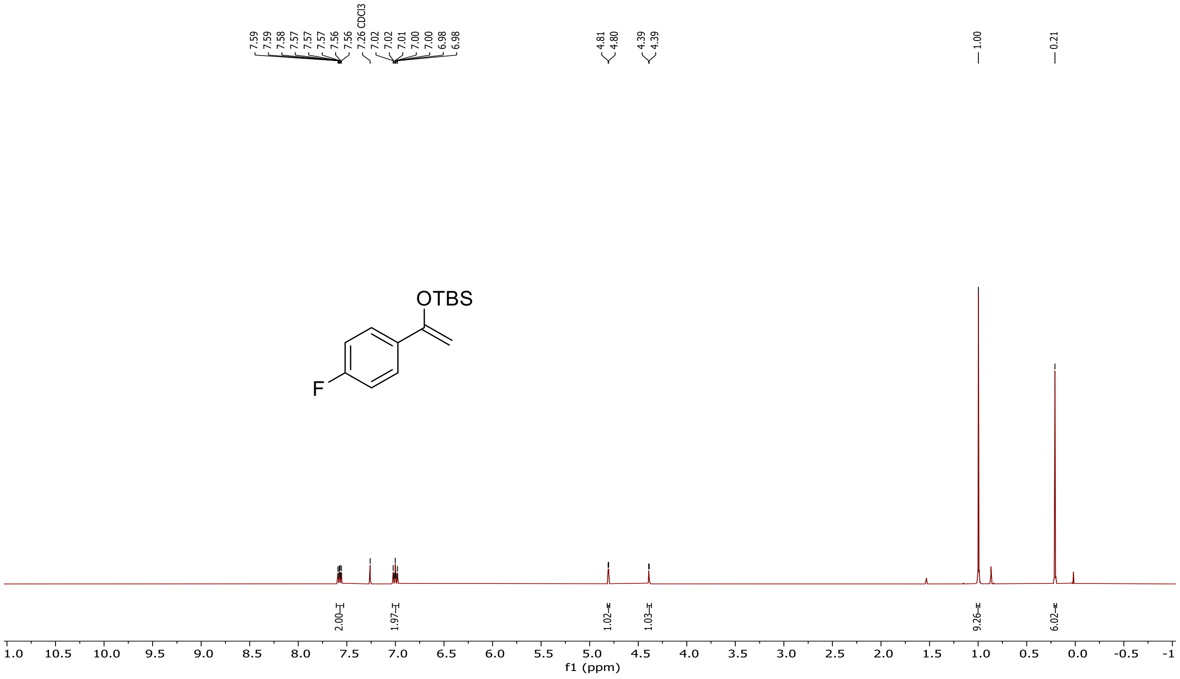


$^{13}C\{1H\}$ NMR (101 MHz, $CDCl_3$)

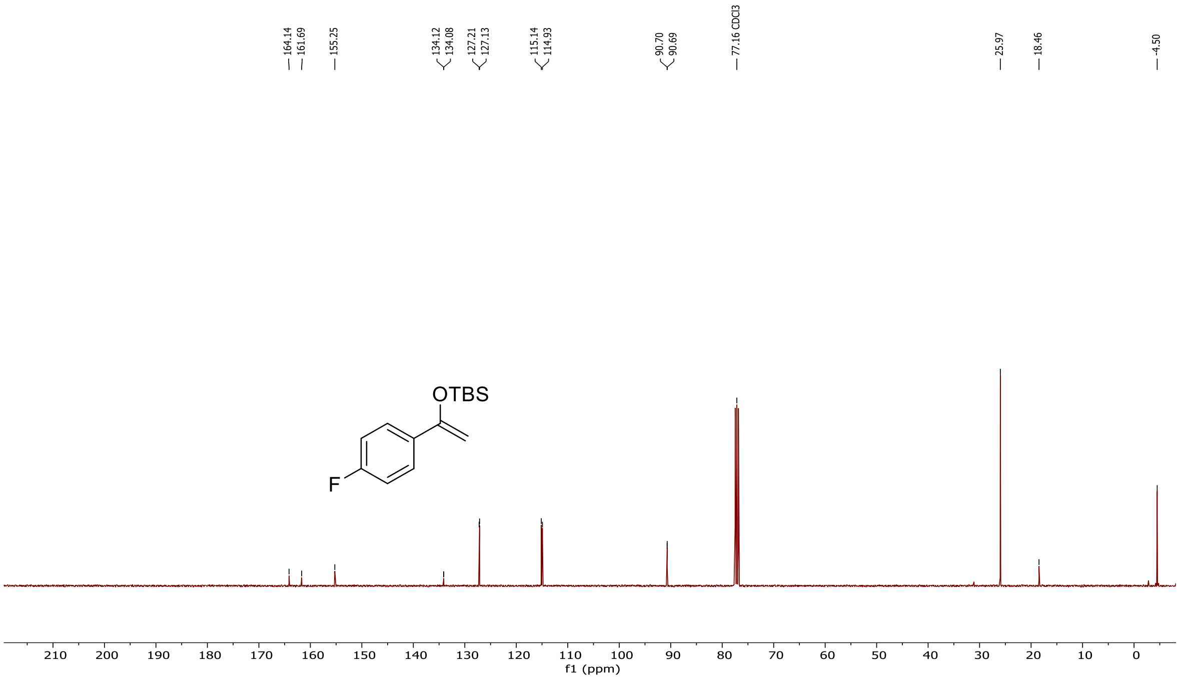

$^{19}$F NMR (282 MHz, $CDCl_3$)

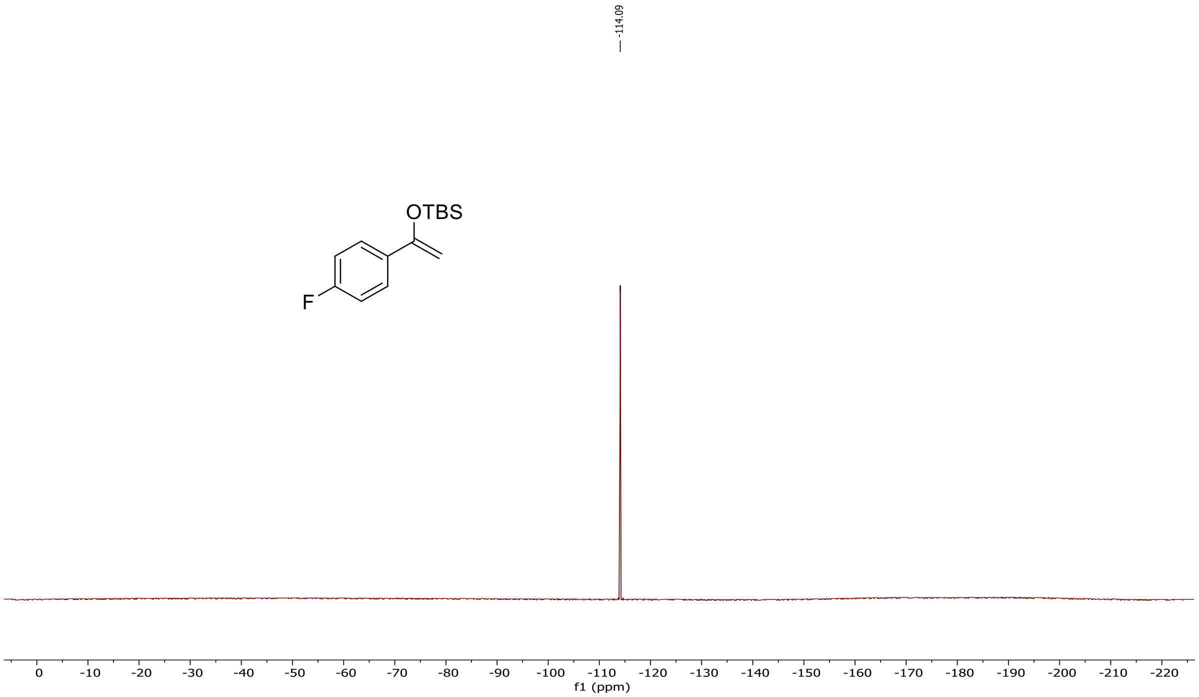


Compound **3i**:

$^{1}$H NMR (400 MHz, $CDCl_3$)

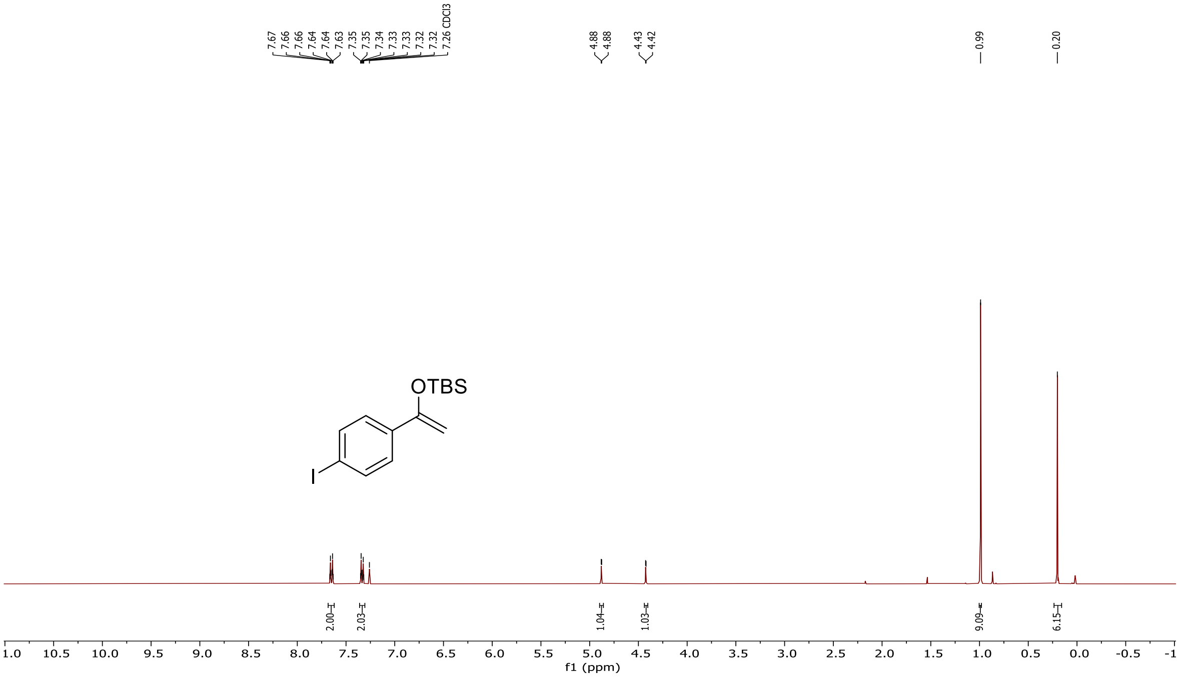

$^{13}C\{1H\}$ NMR (101 MHz, $CDCl_3$)

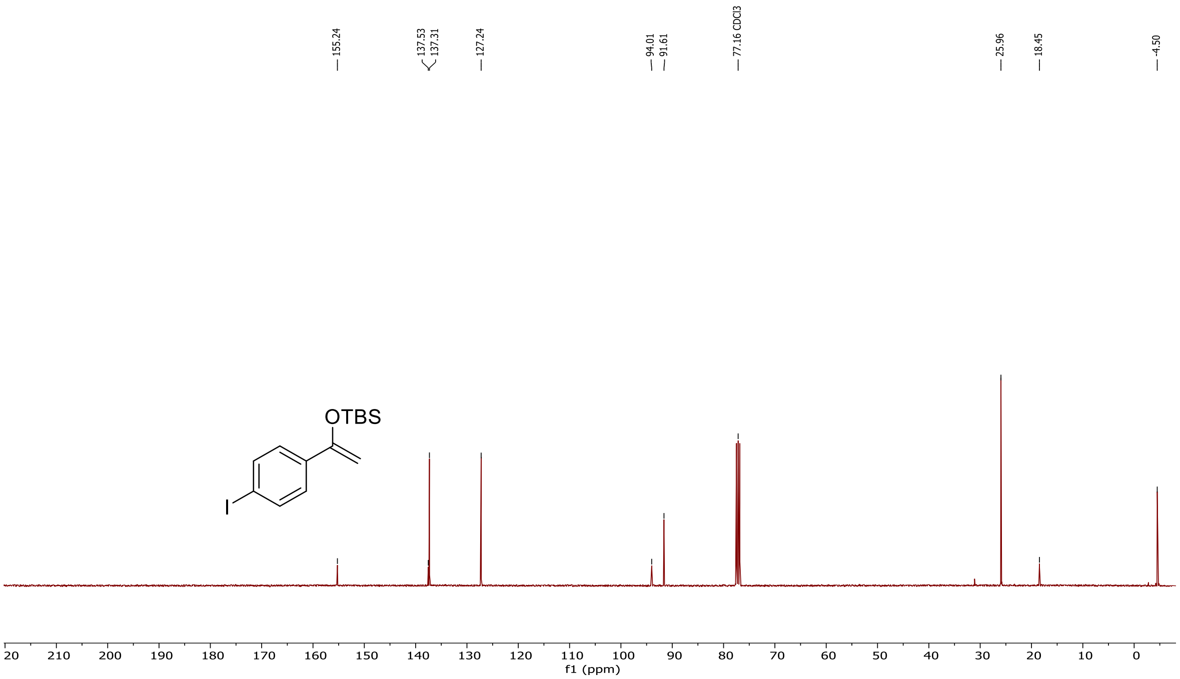


Compound **3m**:

$^{1}H$ NMR (300 MHz, $CDCl_3$)

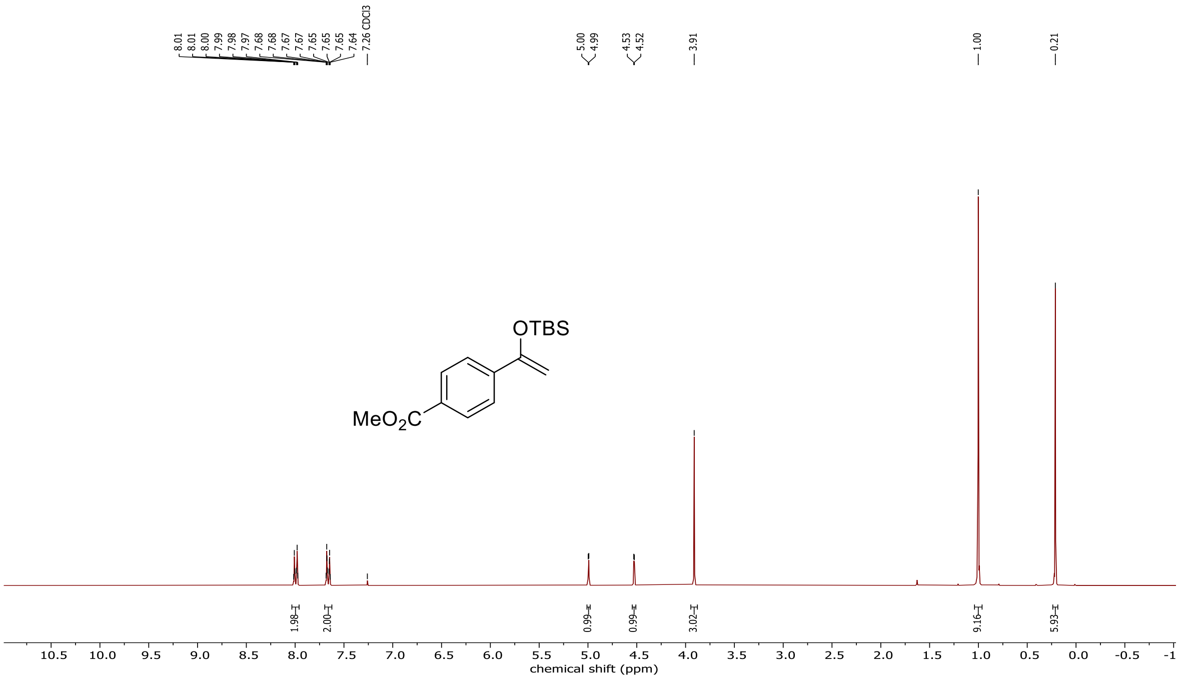

$^{13}C\{1H\}$ NMR (101 MHz, $CDCl_3$)

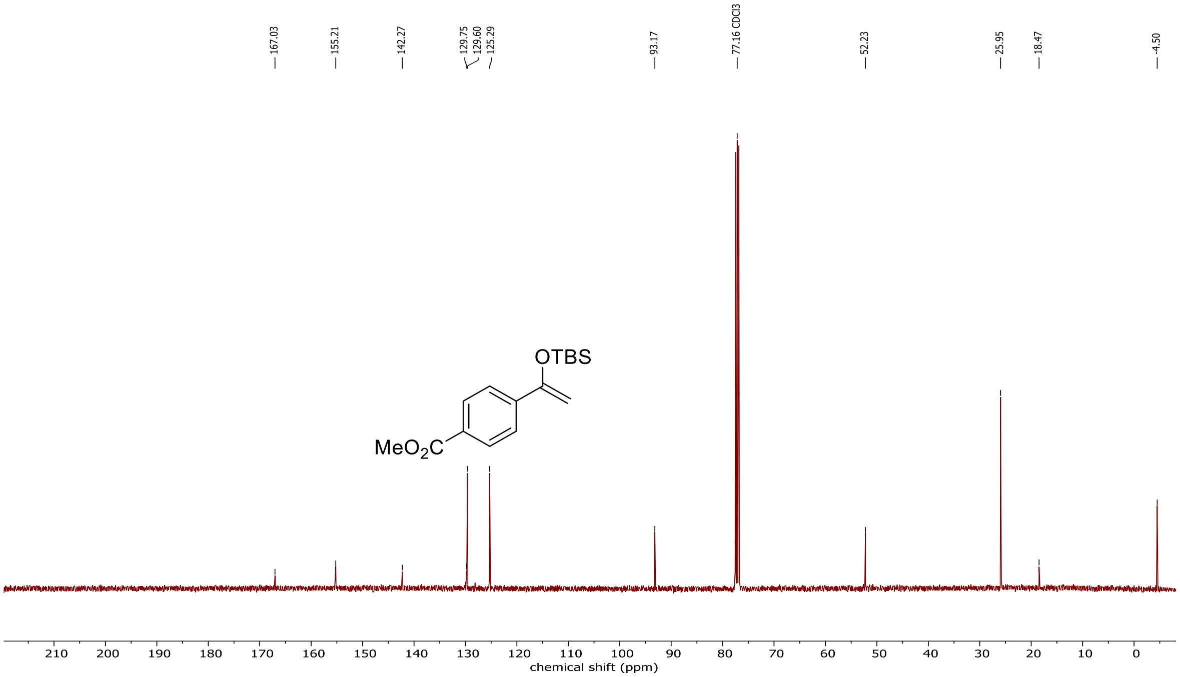


Compound **3n**:

$^{1}H$ NMR (300 MHz, $CDCl_3$)

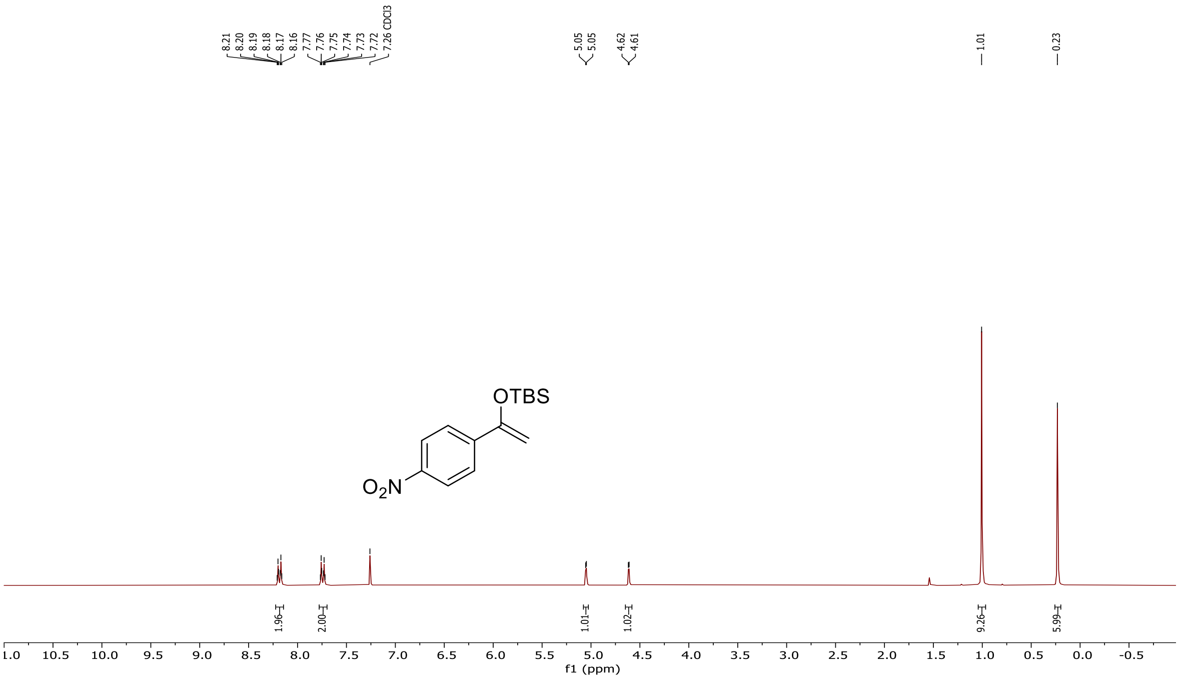

$^{13}C\{1H\}$ NMR (101 MHz, $CDCl_3$)

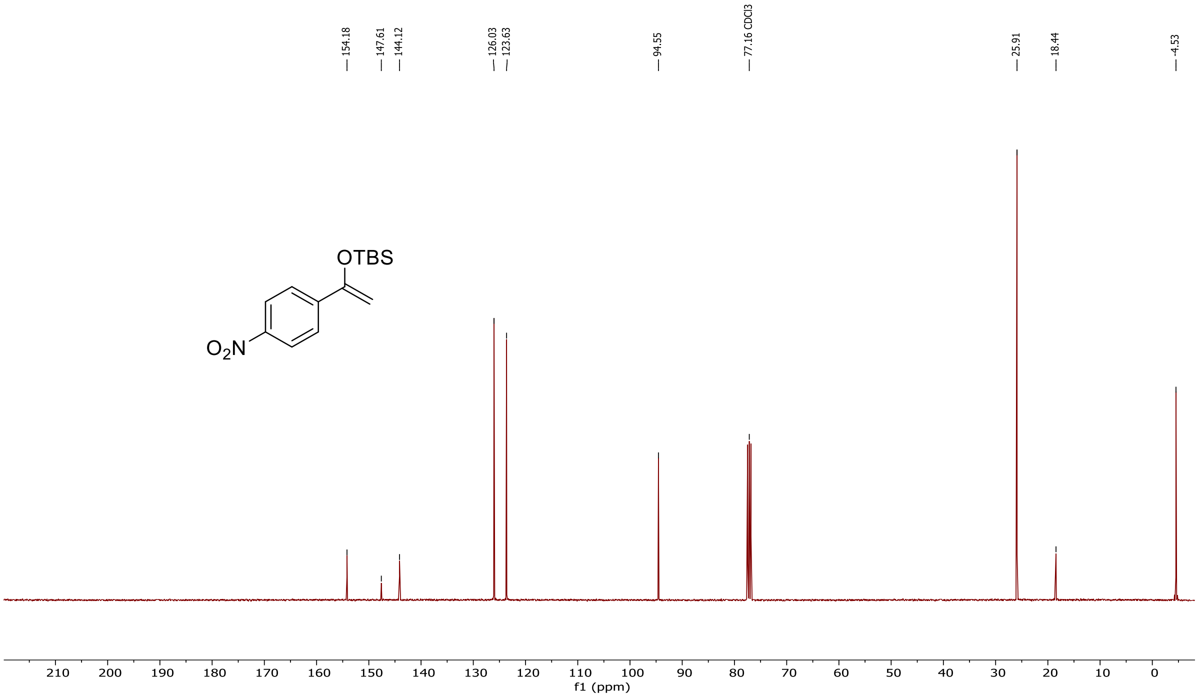


Compound **3q**:

$^1H$ NMR (400 MHz, $CDCl_3$)

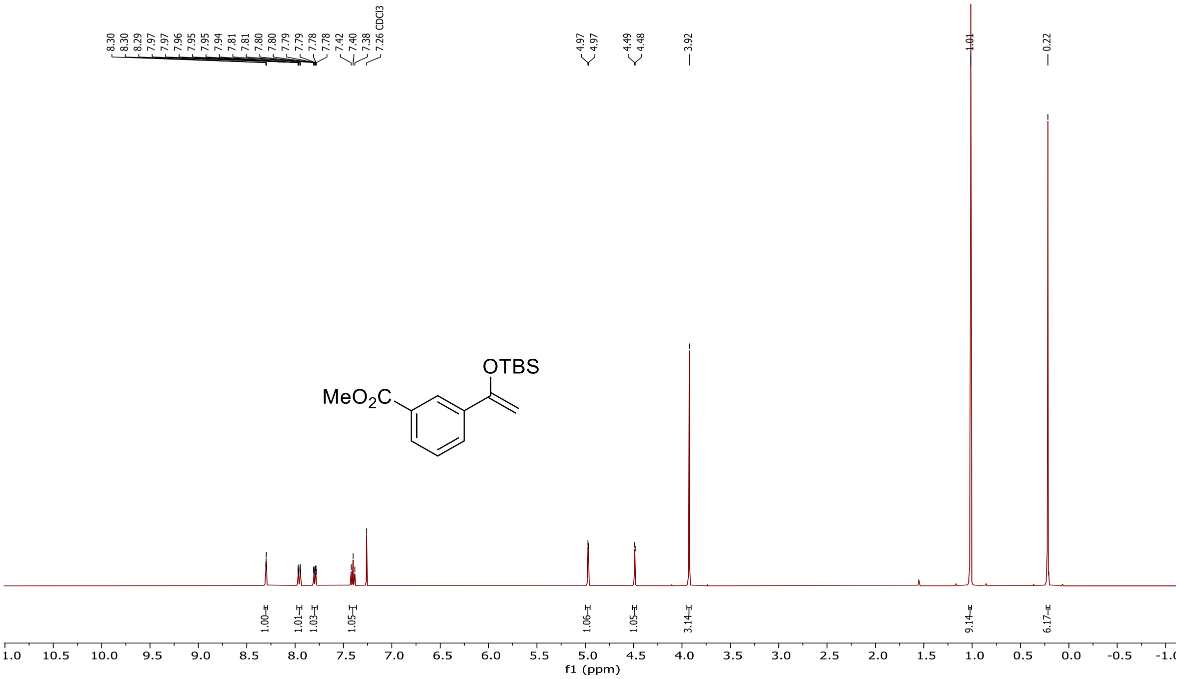

$^{13}$C{1H} NMR (101 MHz, $CDCl_3$)

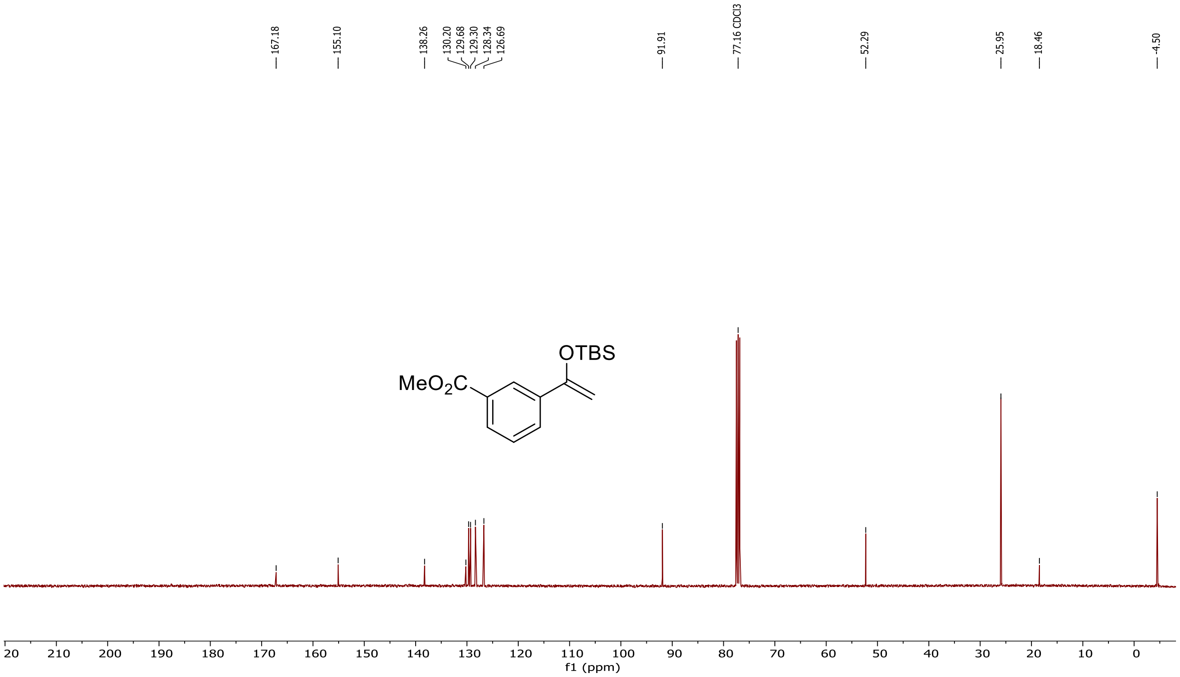


Compound **3u**:

$^{1}$H NMR (400 MHz, $CDCl_3$)

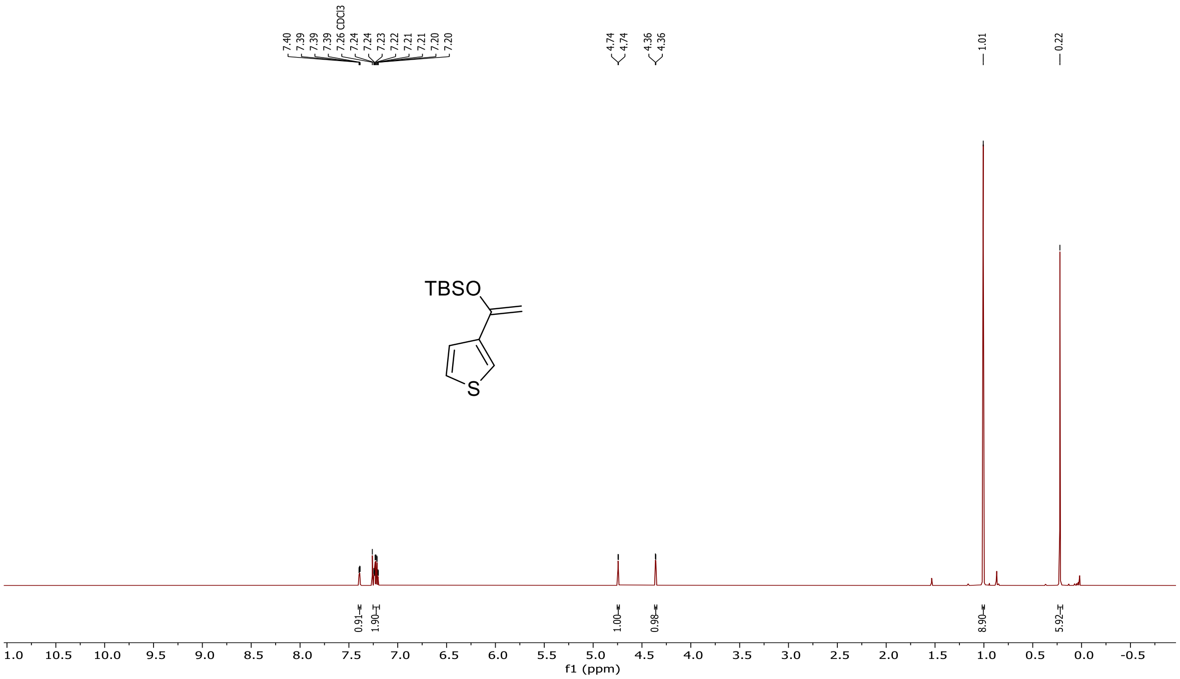

$^{13}C\{1H\}$ NMR (101 MHz, $CDCl_3$)

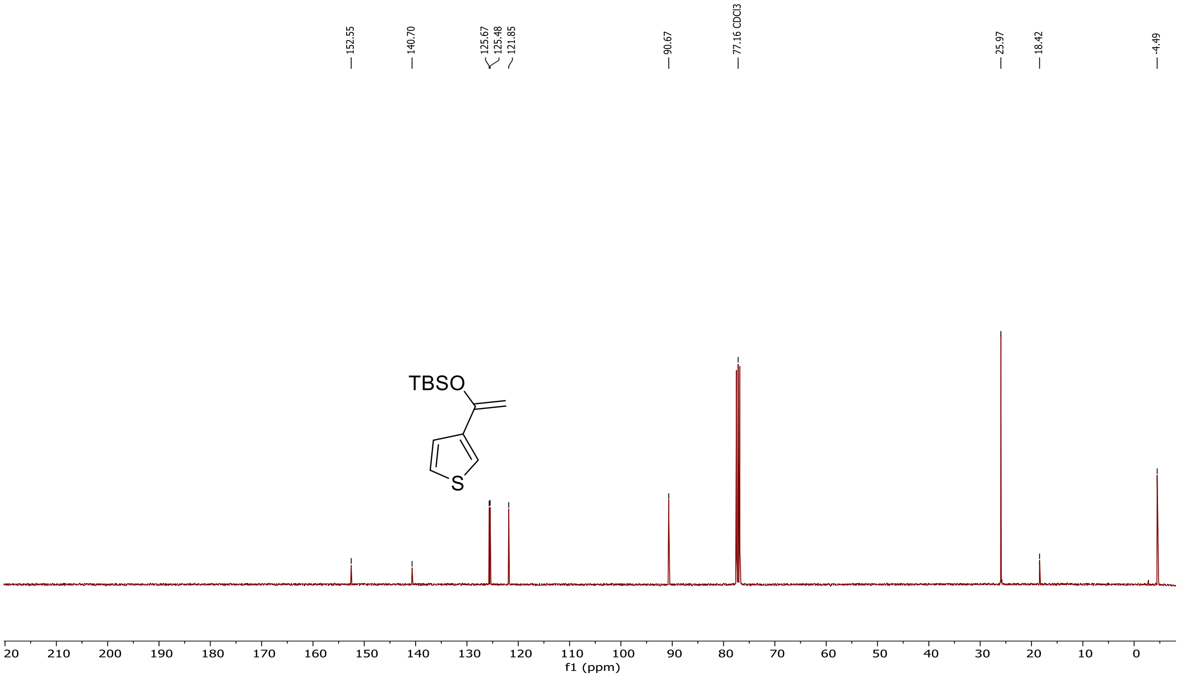


Compound **3v**:

$^1H$ NMR (300 MHz, $CDCl_3$)

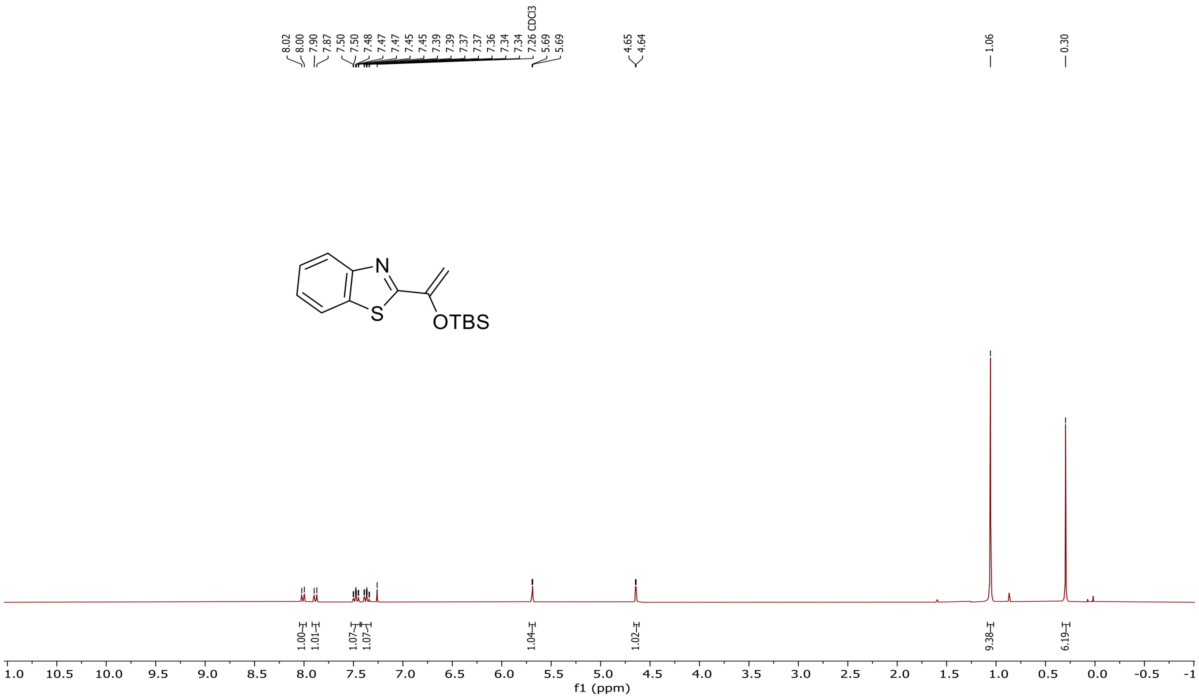

$^{13}C\{1H\}$ NMR (101 MHz, $CDCl_3$)

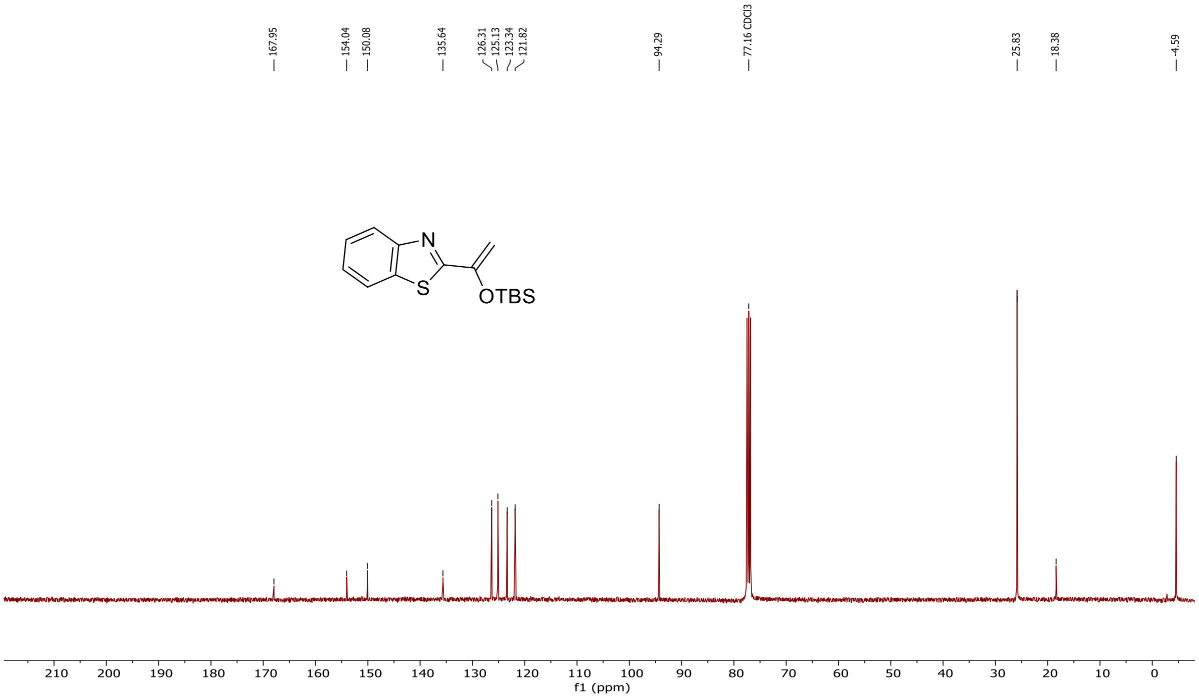


Compound **3w**:

$^{1}H$ NMR (400 MHz, $CDCl_3$)

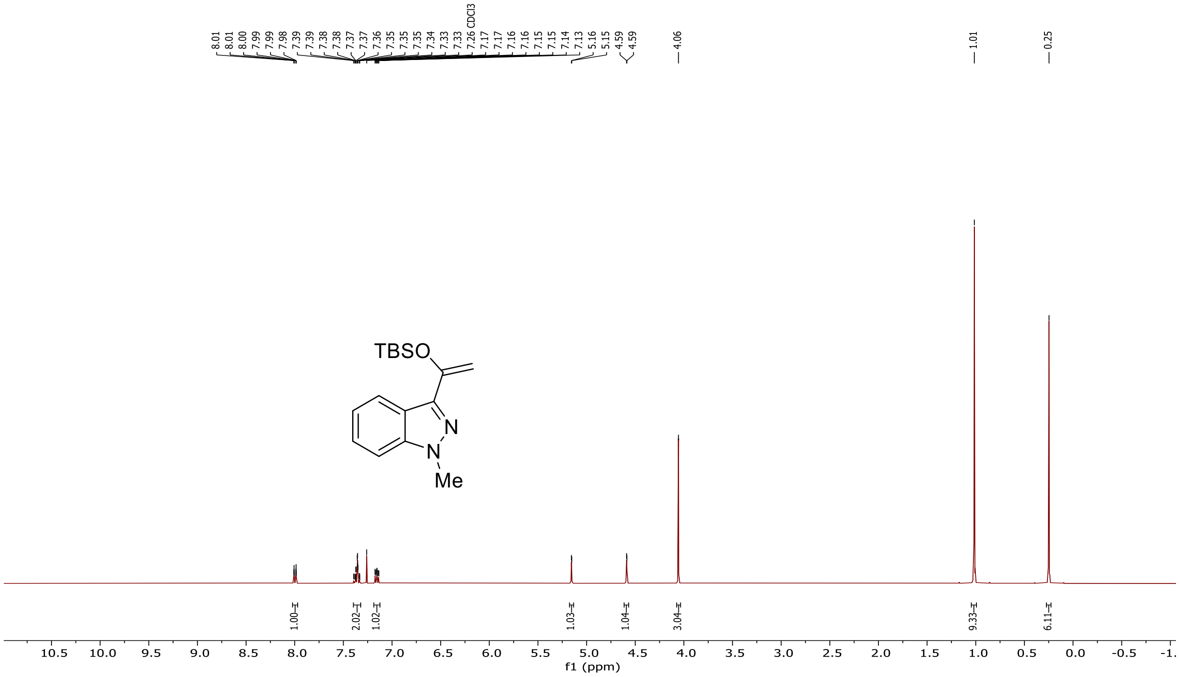

$^{13}C\{1H\}$ NMR (101 MHz, $CDCl_3$)

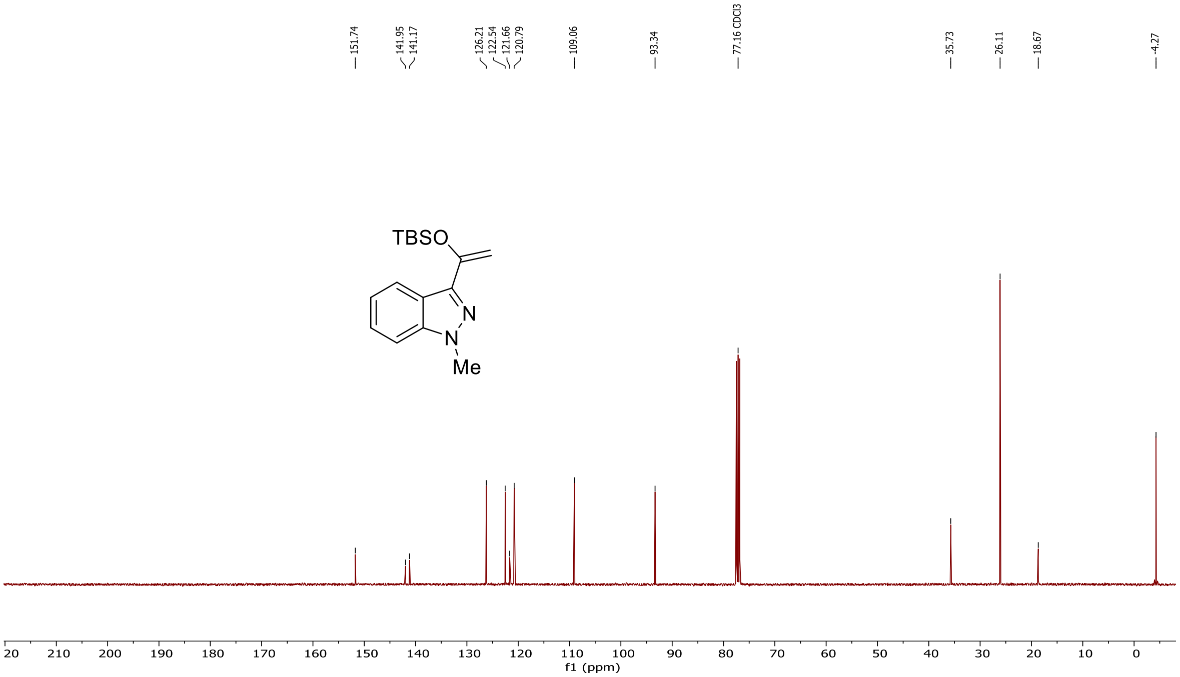


Compound **3x**:

$^{1}H$ NMR (300 MHz, $CDCl_3$)

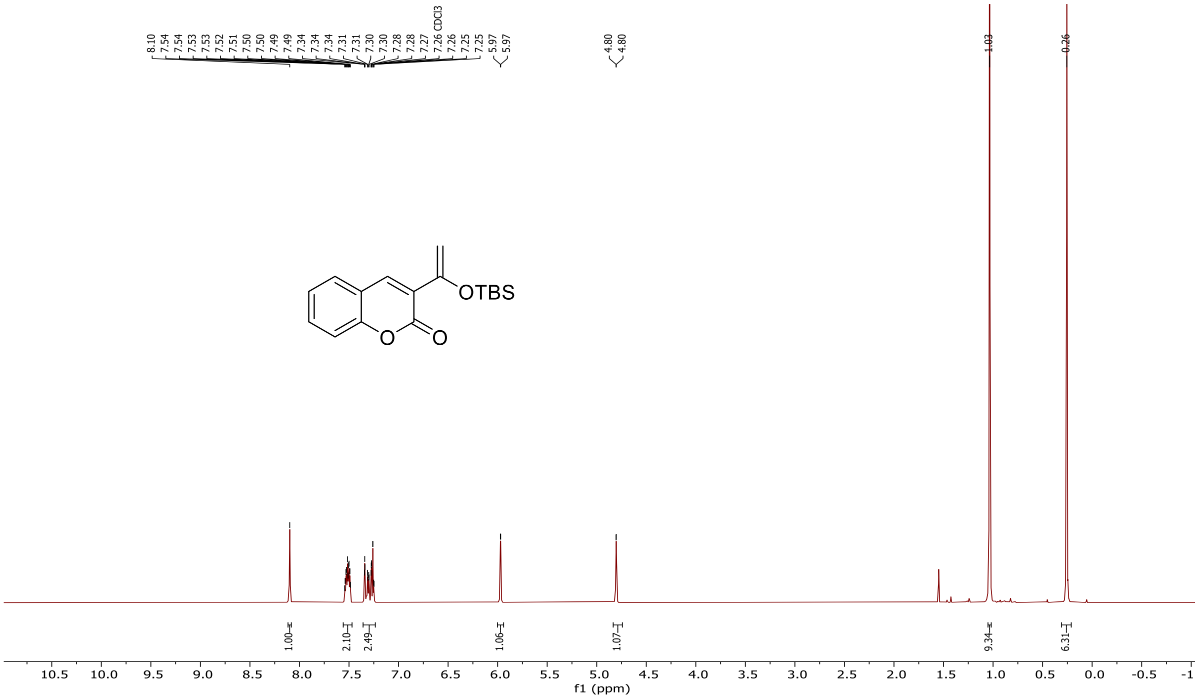

$^{13}C\{1H\}$ NMR (101 MHz, $CDCl_3$)

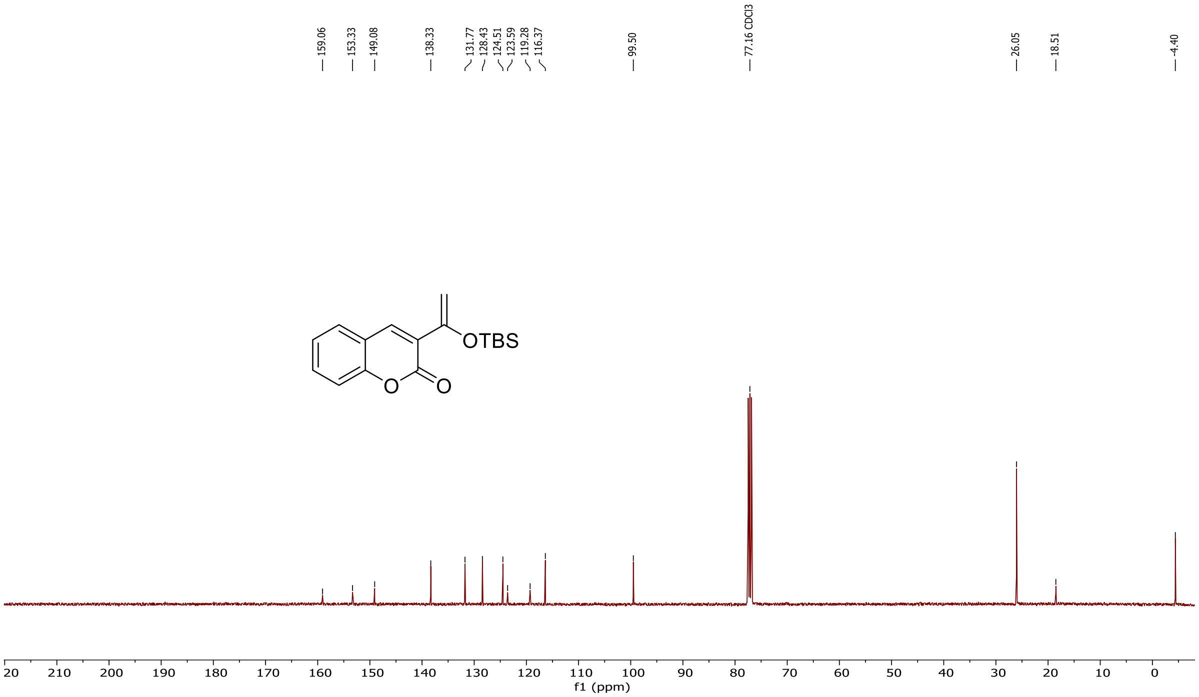


Compound **3y**:

$^{1}H$ NMR (400 MHz, $CDCl_3$)

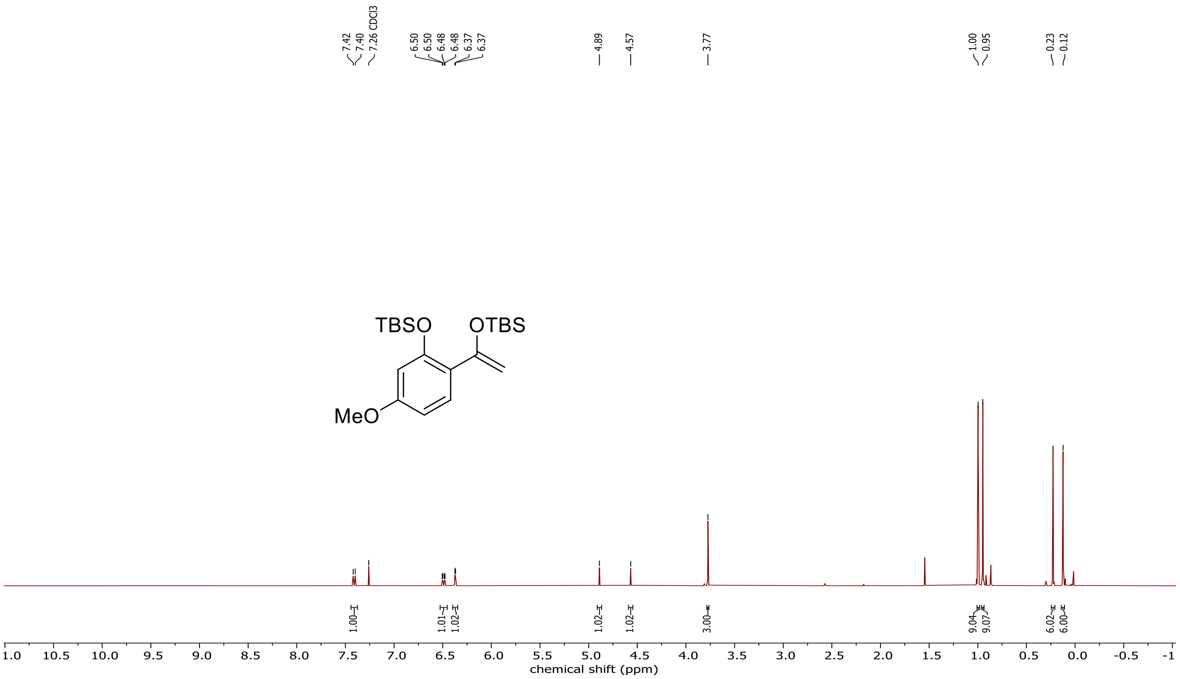

$^{13}C\{1H\}$ NMR (101 MHz, $CDCl_3$)

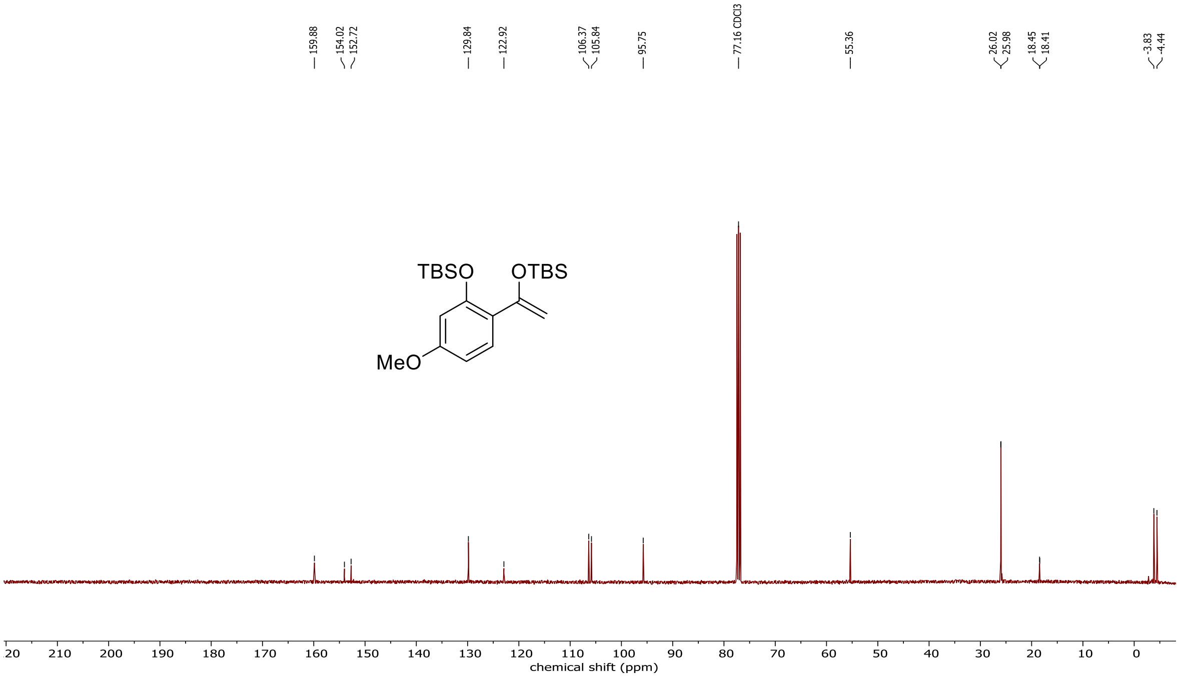


Compound **3z**:

$^{1}H$ NMR (400 MHz, $CDCl_3$)

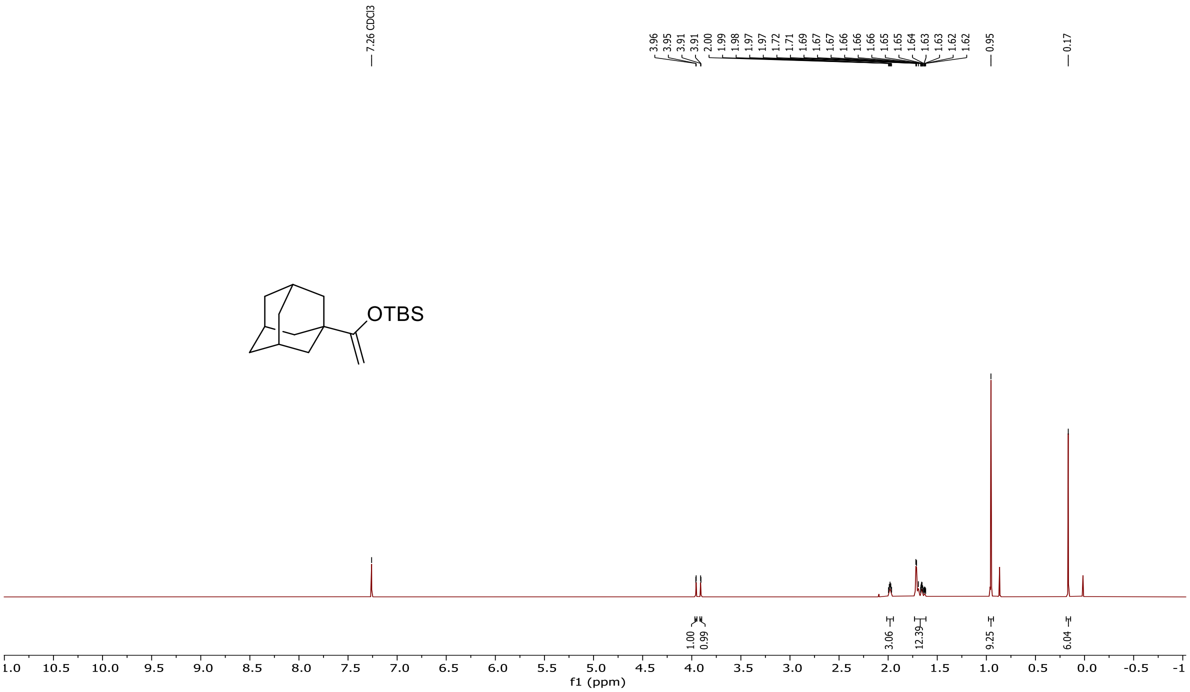

$^{13}C\{1H\}$ NMR (101 MHz, $CDCl_3$)

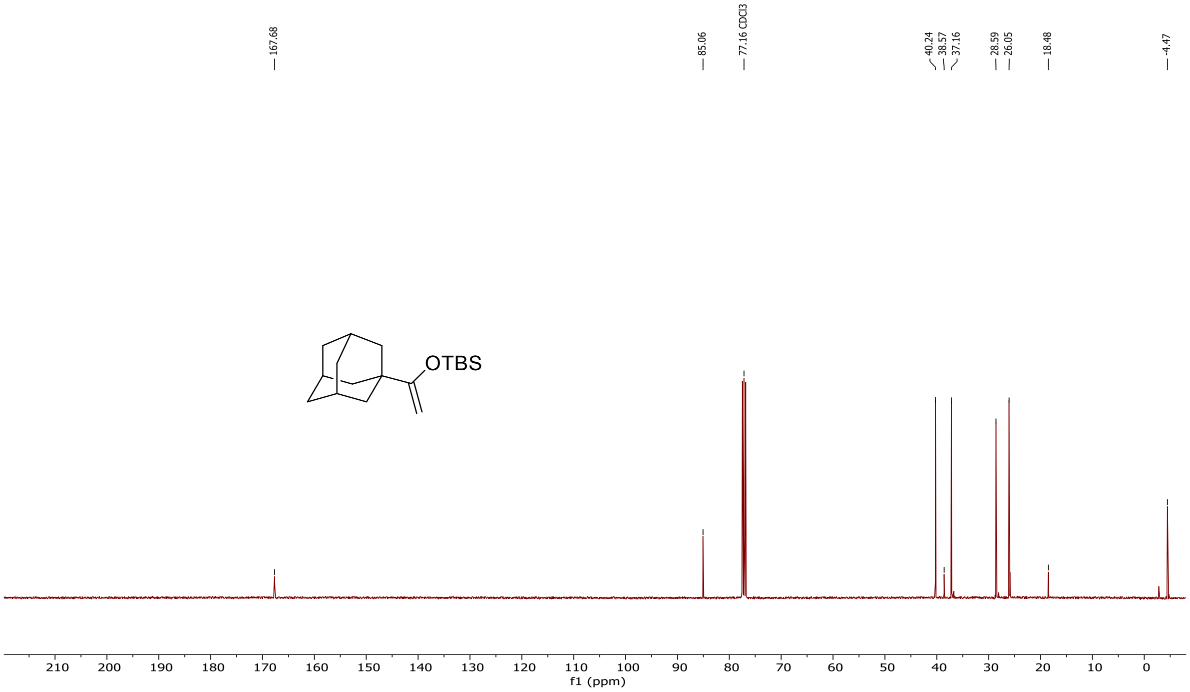


Compound **5w**:

$^1H$ NMR (400 MHz, $CDCl_3$)

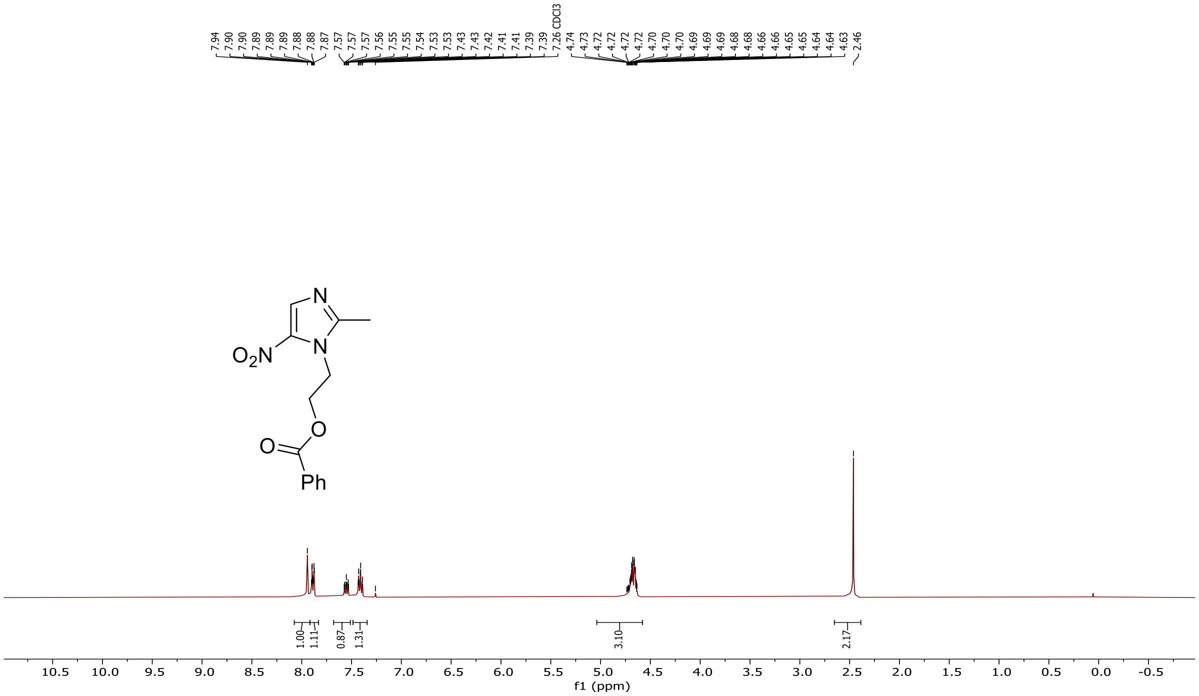

$^{13}C\{1H\}$ NMR (101 MHz, $CDCl_3$)

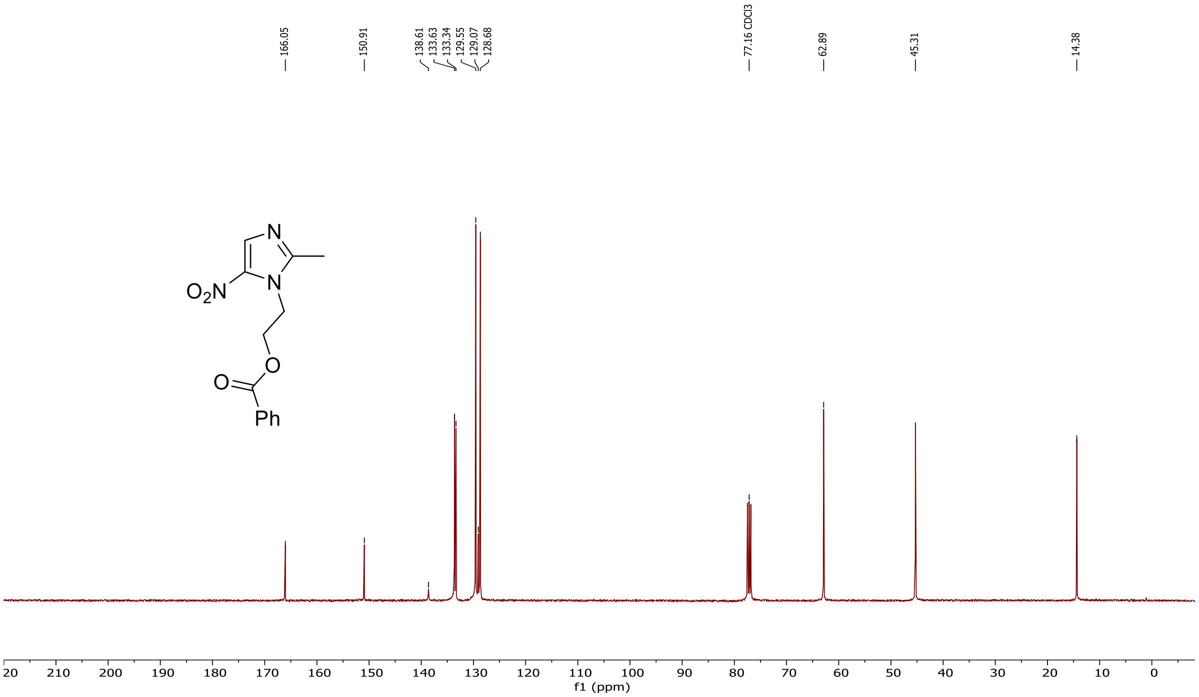


Compound **5ad**:

$^1H$ NMR (400 MHz, $CDCl_3$)

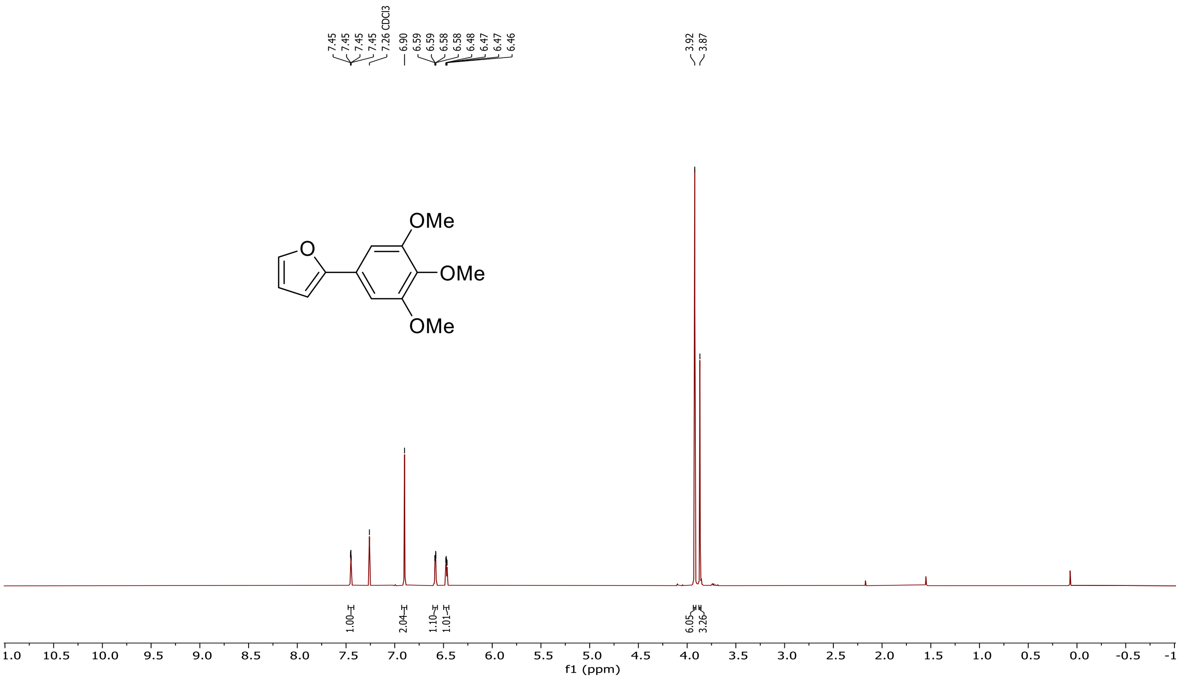

$^{13}C\{1H\}$ NMR (101 MHz, $CDCl_3$)

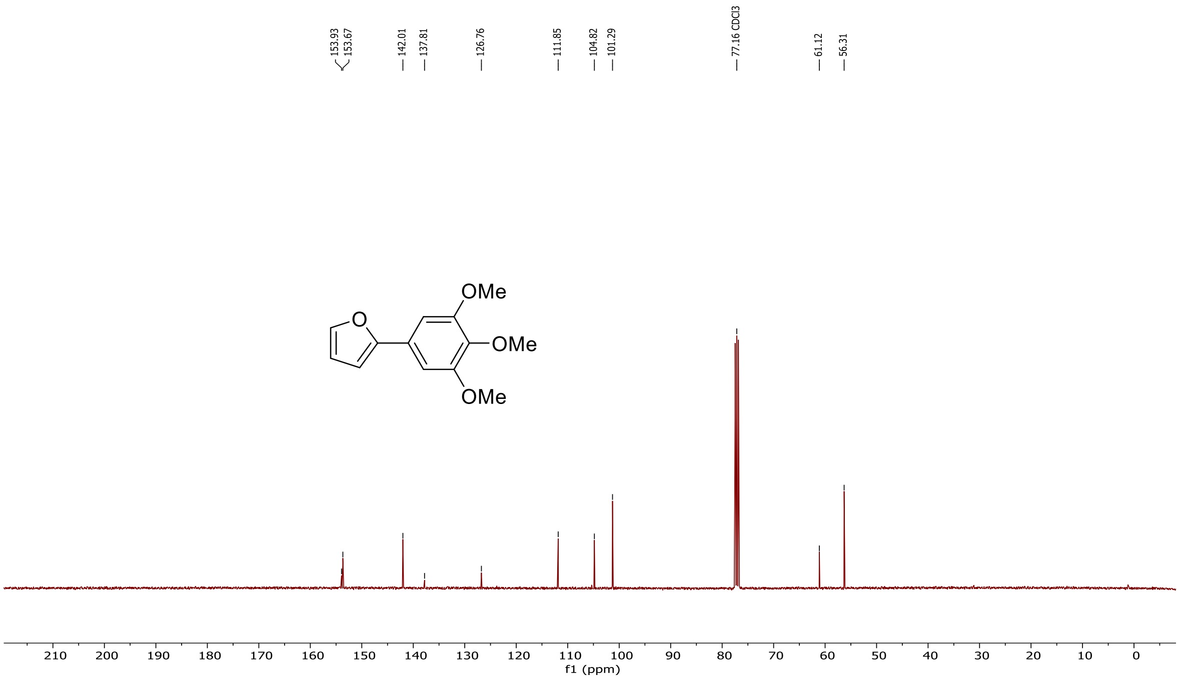


Compound **5ag**:

$^{1}H$ NMR (300 MHz, $CDCl_3$)

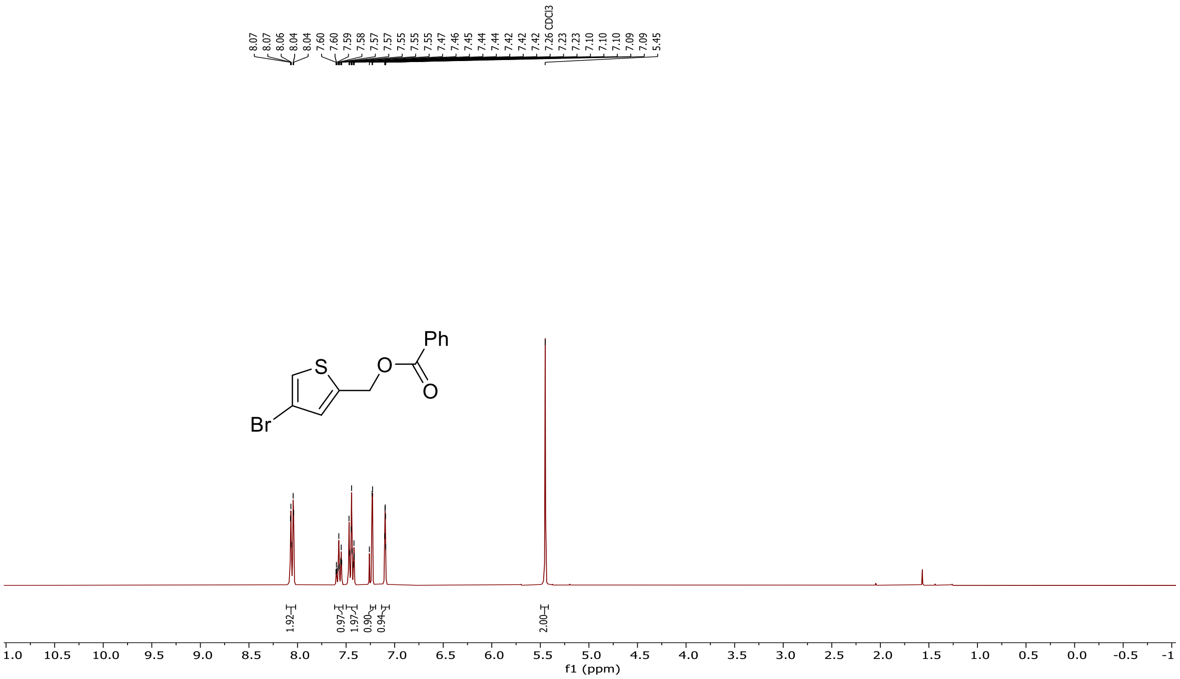

$^{13}C\{1H\}$ NMR (101 MHz, $CDCl_3$)

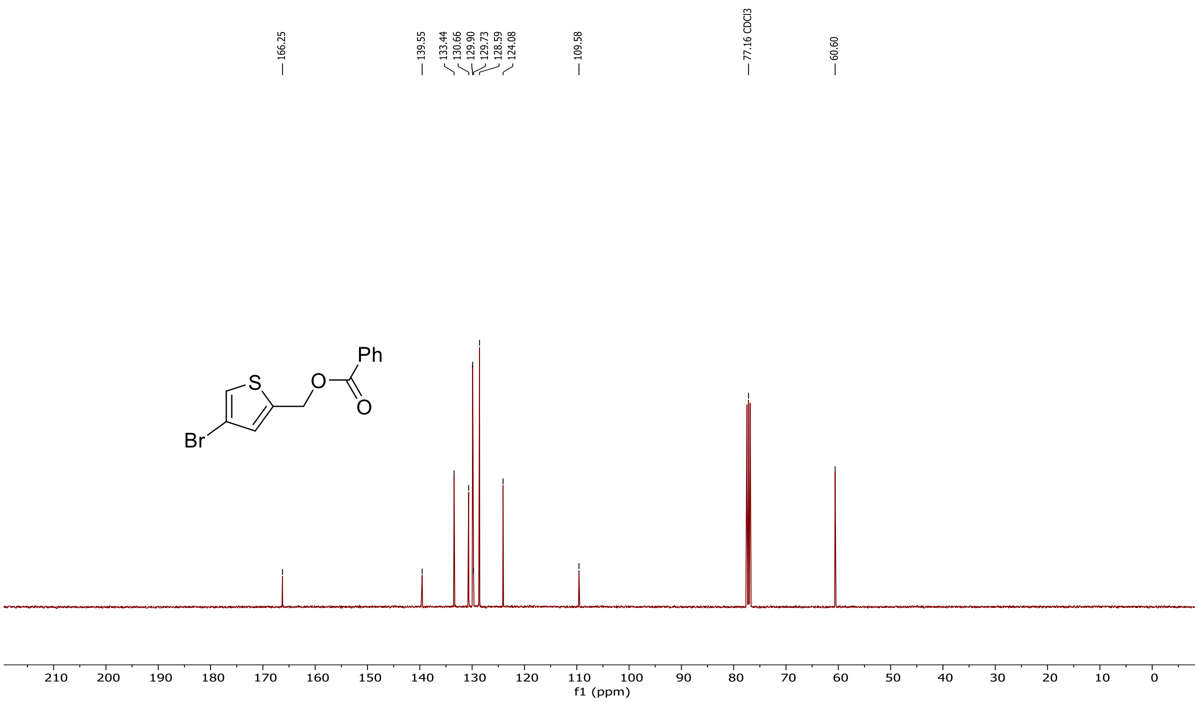


Compound **5ai**:

$^{1}H$ NMR (300 MHz, $CDCl_3$)

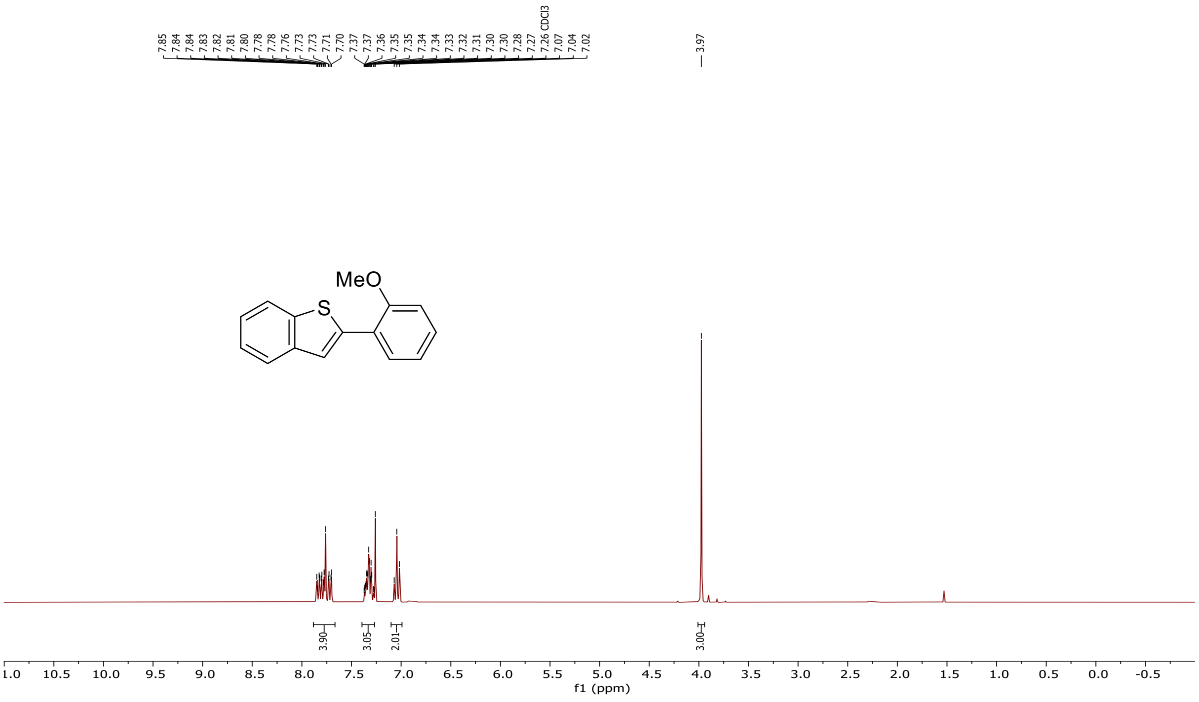

$^{13}C\{1H\}$ NMR (101 MHz, $CDCl_3$)

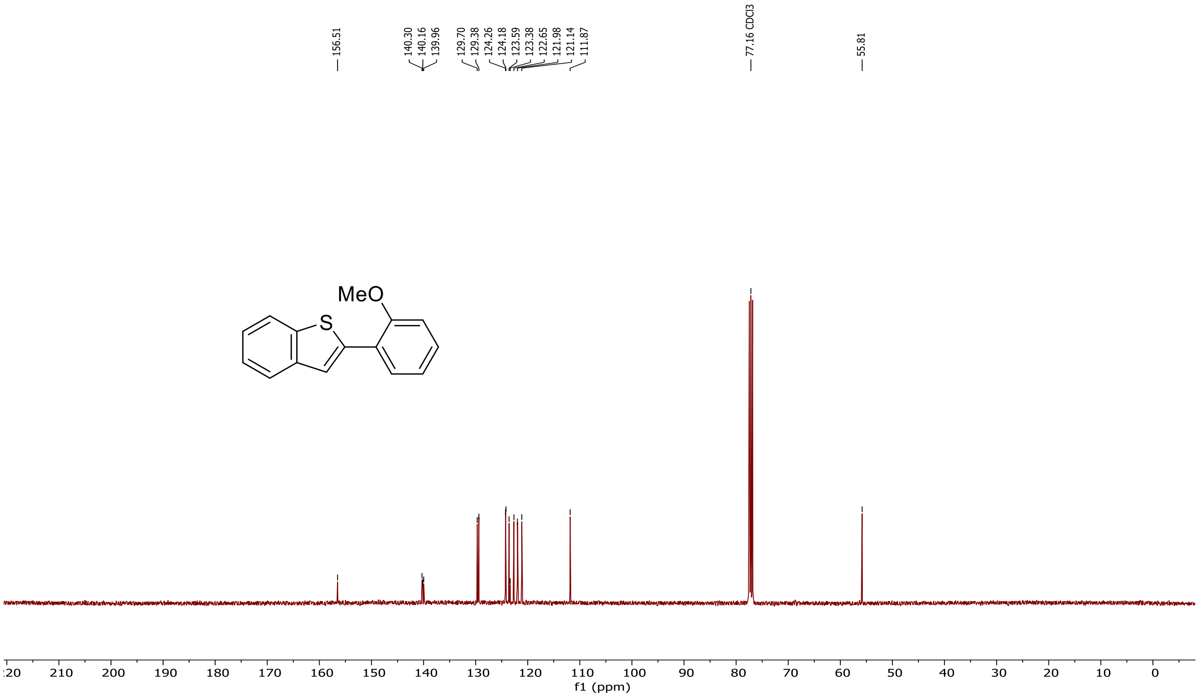


Compound **4a**:

$^1H$ NMR (300 MHz, $CDCl_3$)

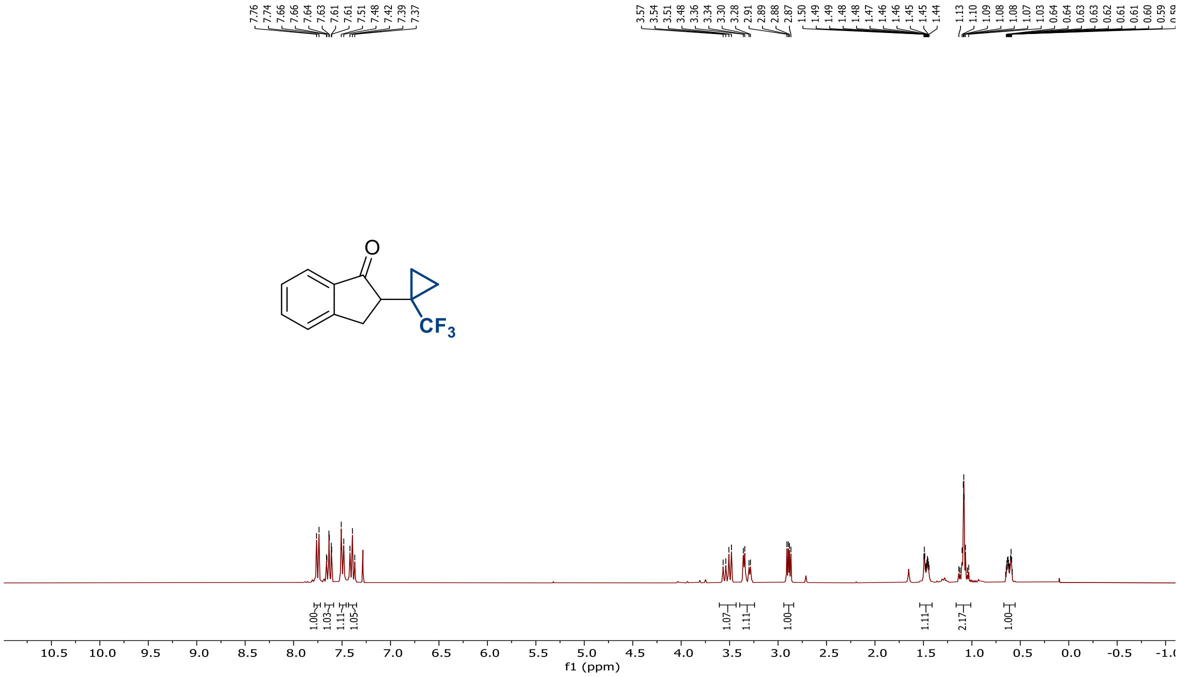

$^{13}C\{1H\}$ NMR (101 MHz, $CDCl_3$)

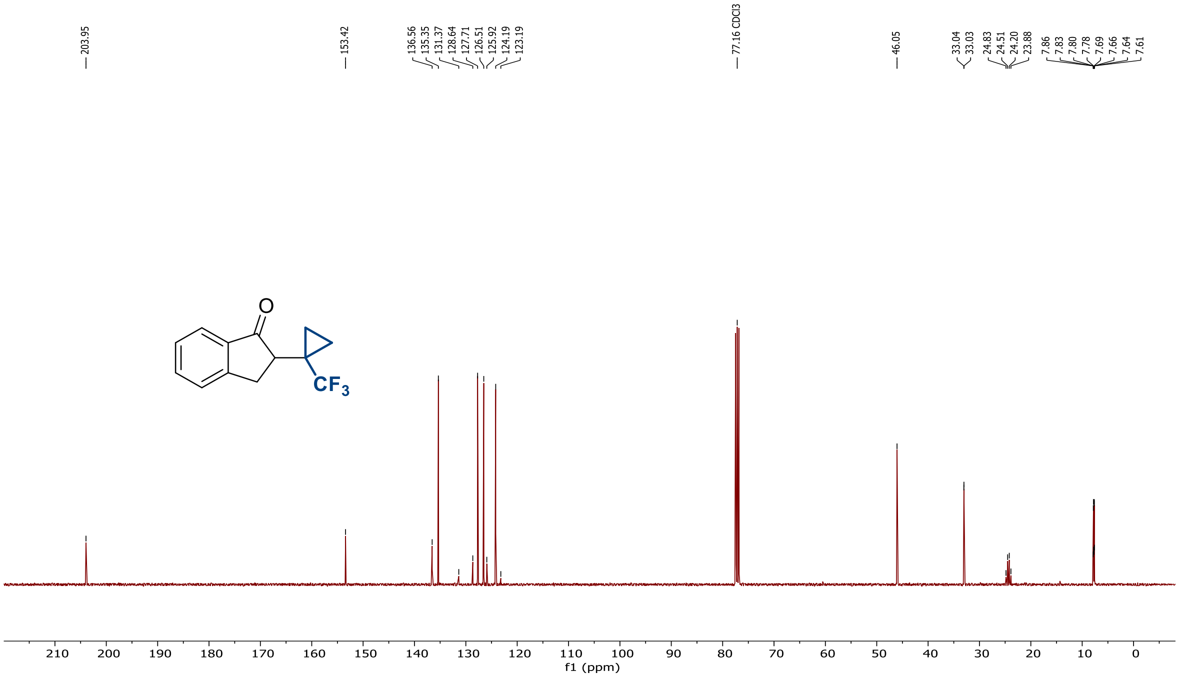


$^{19}F$ NMR (282 MHz, $CDCl_3$)

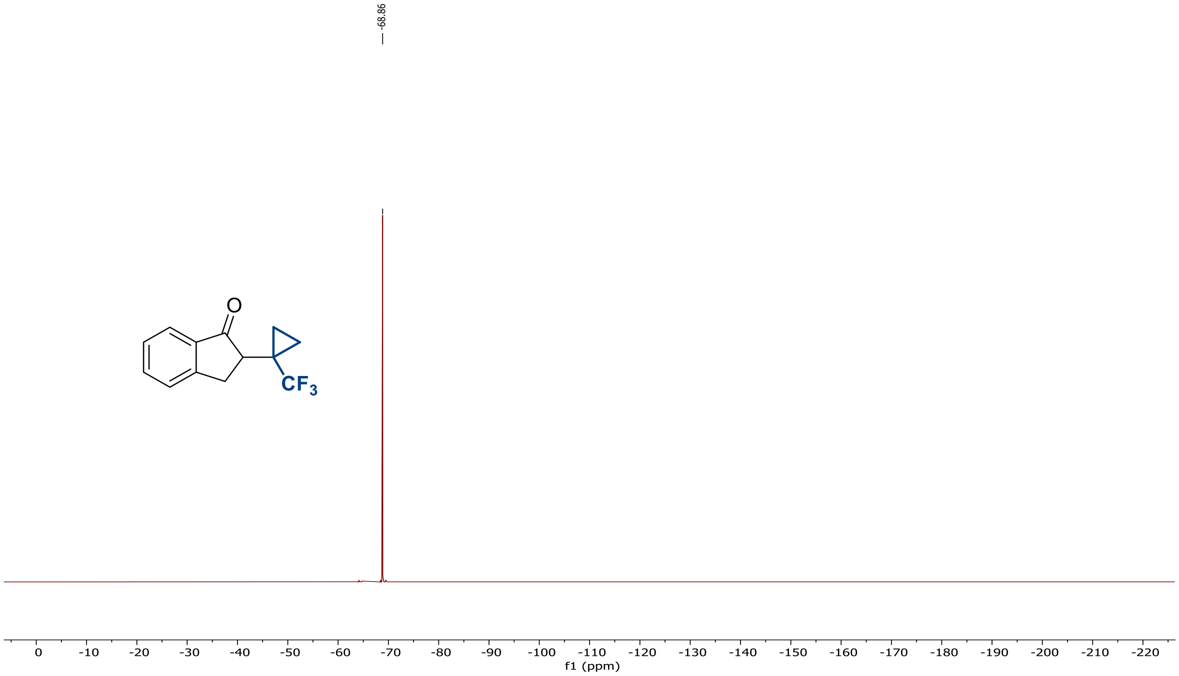

Compound **4c**:

$^{1}$H NMR (400 MHz, $CDCl_3$)

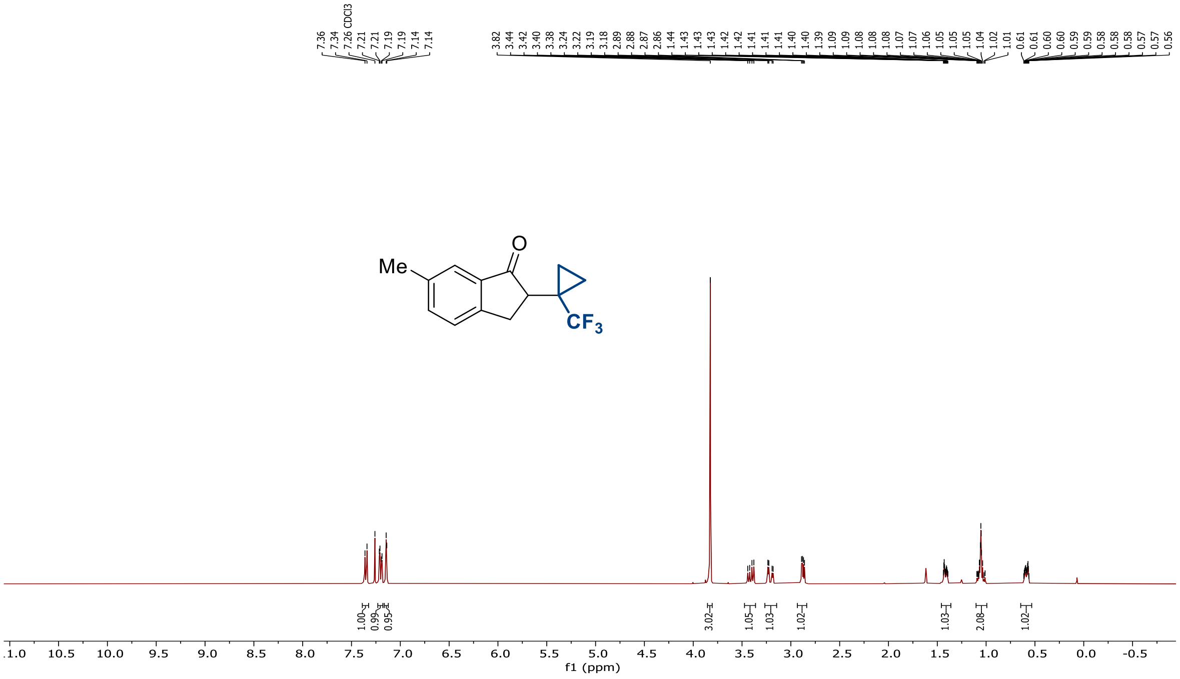


$^{13}$C{1H} NMR (101 MHz, $CDCl_3$)

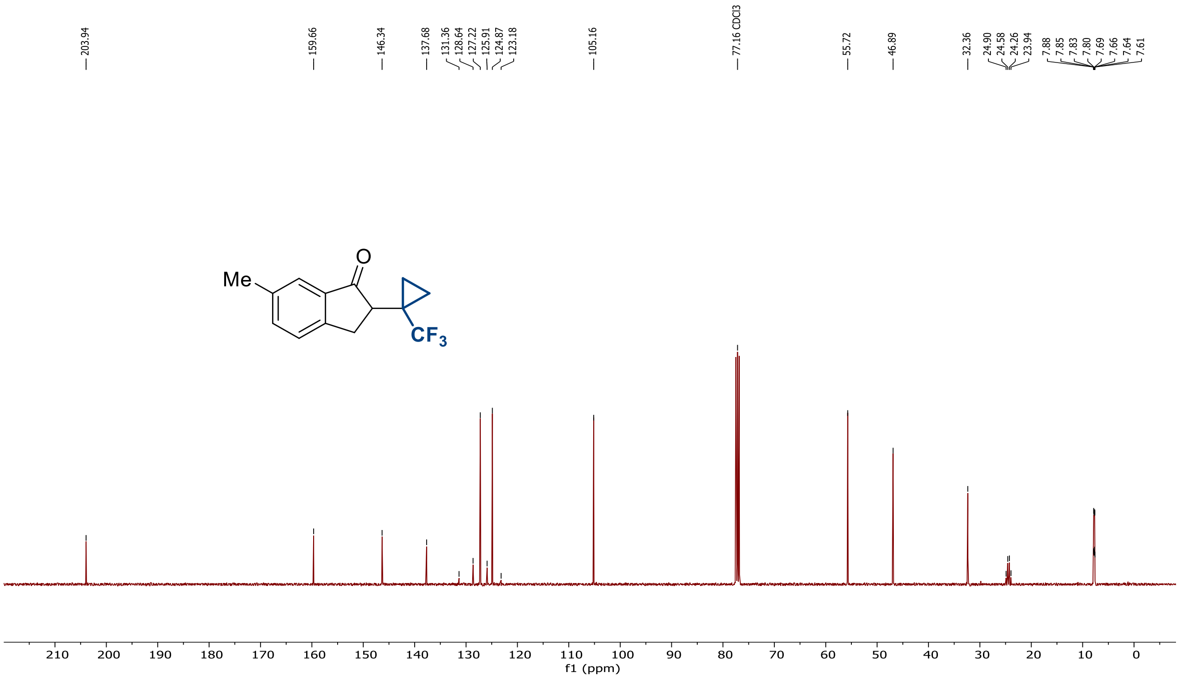

$^{19}F$ NMR (377 MHz, $CDCl_3$)

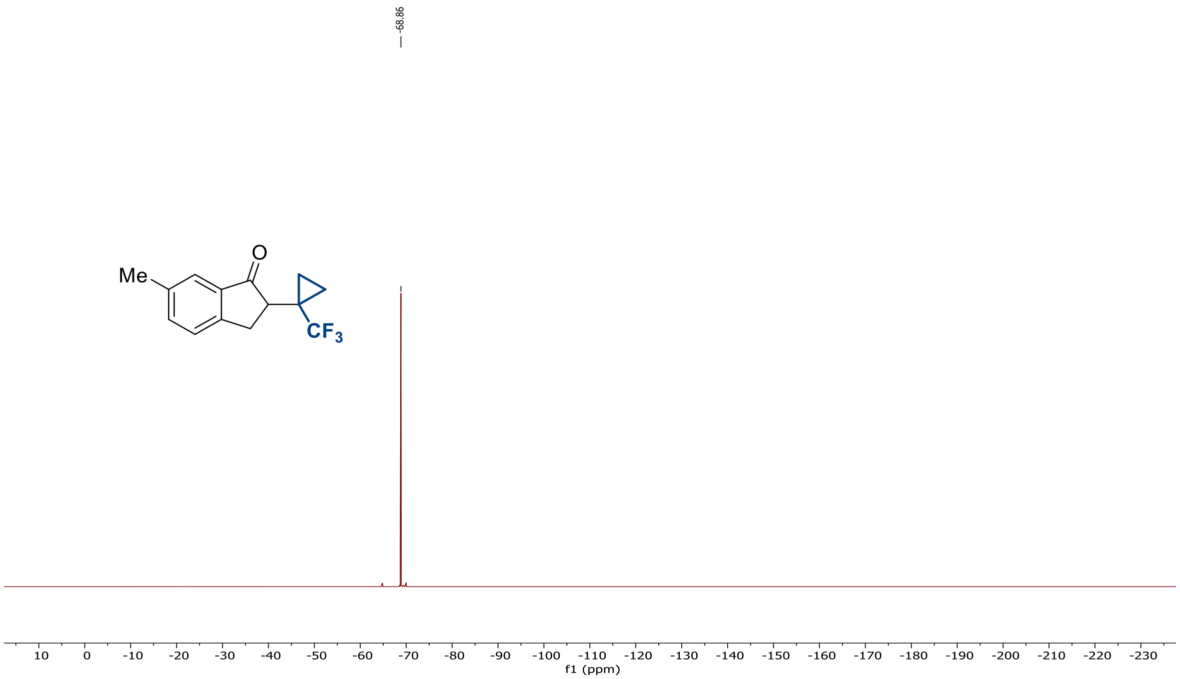


Compound **4d**:

$^{1}H$ NMR (400 MHz, $CDCl_3$)

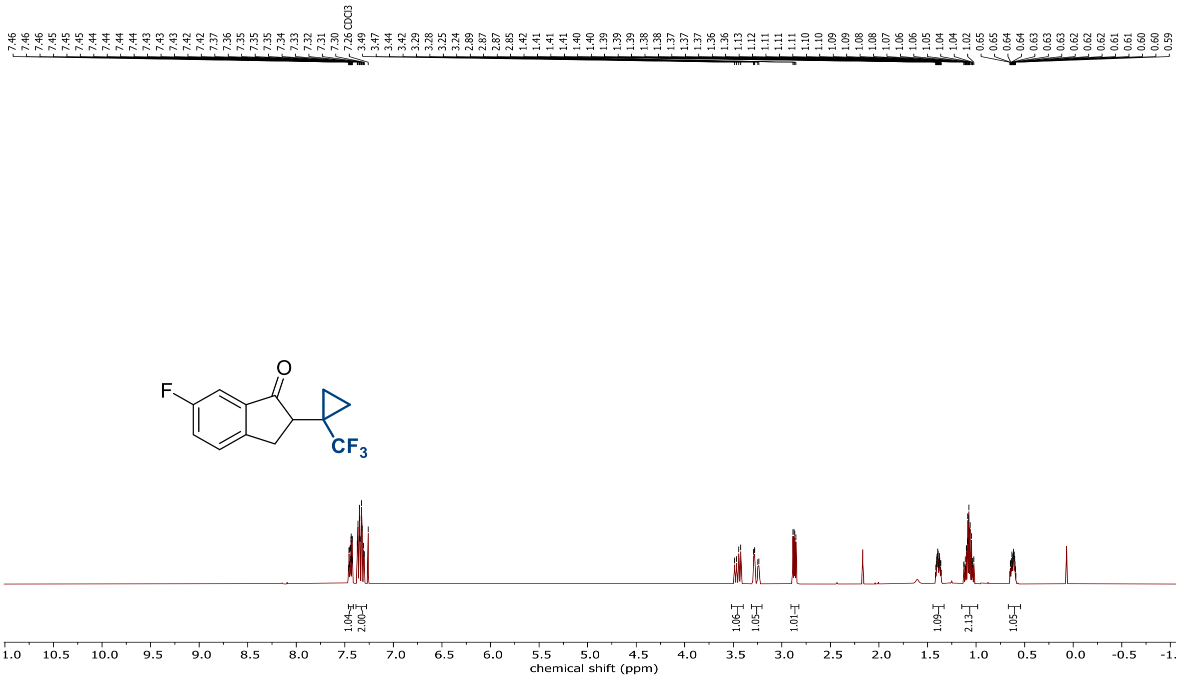

$^{13}C\{1H\}$ NMR (101 MHz, $CDCl_3$)

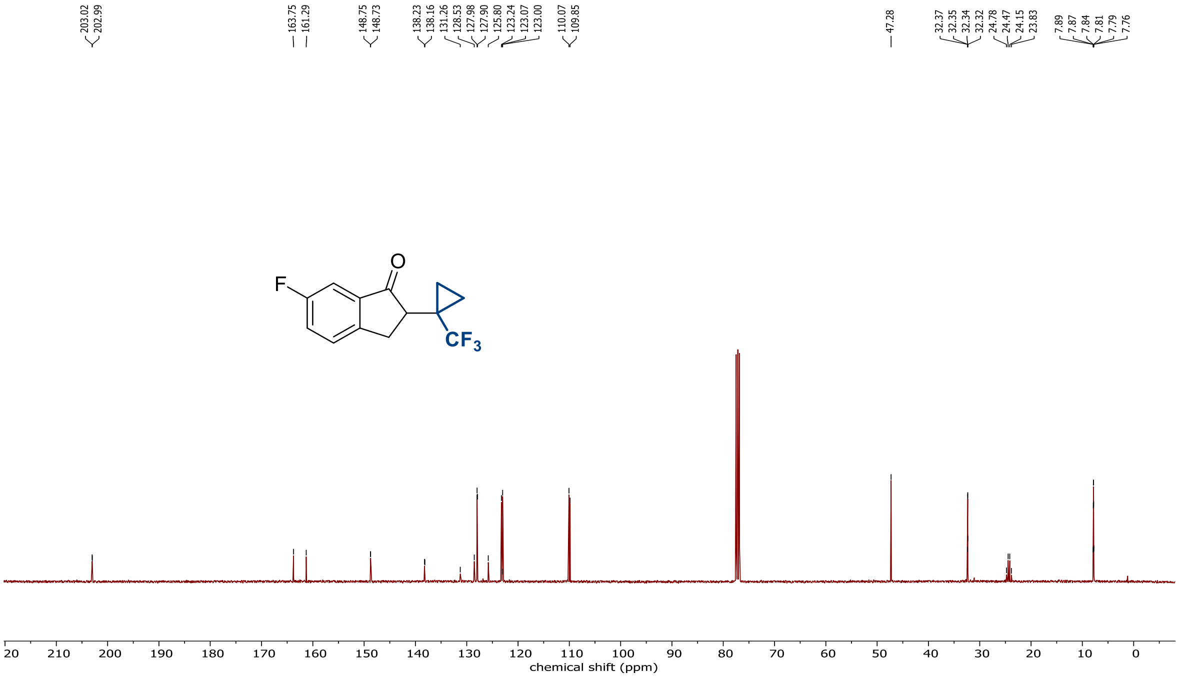


$^{19}F$ NMR (377 MHz, $CDCl_3$)

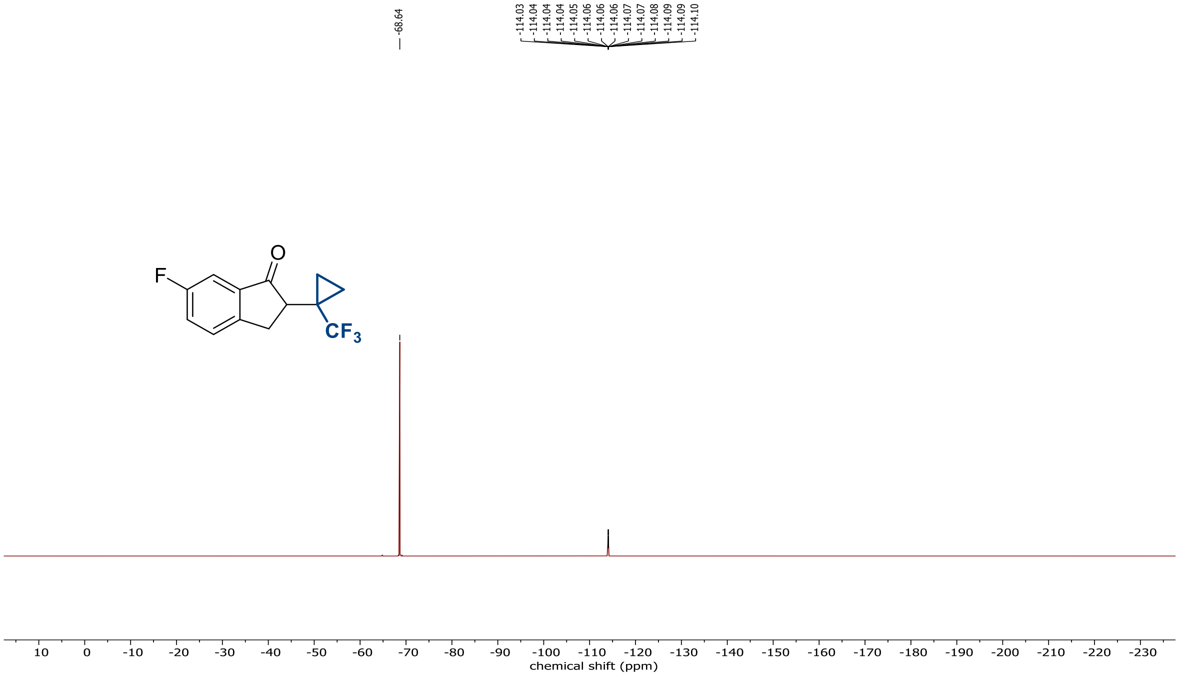

Compound **4e**:

$^{1}H$ NMR (300 MHz, $CDCl_3$)

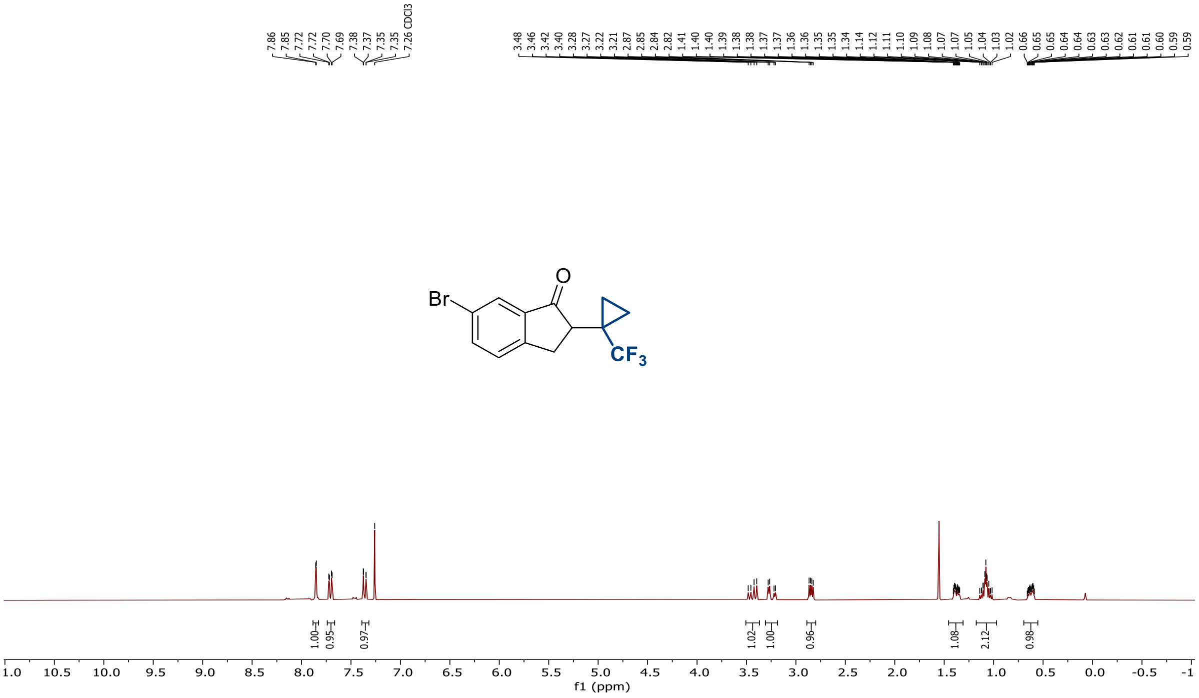


$^{13}C\{^{1}H\}$ NMR (101 MHz, $CDCl_3$)

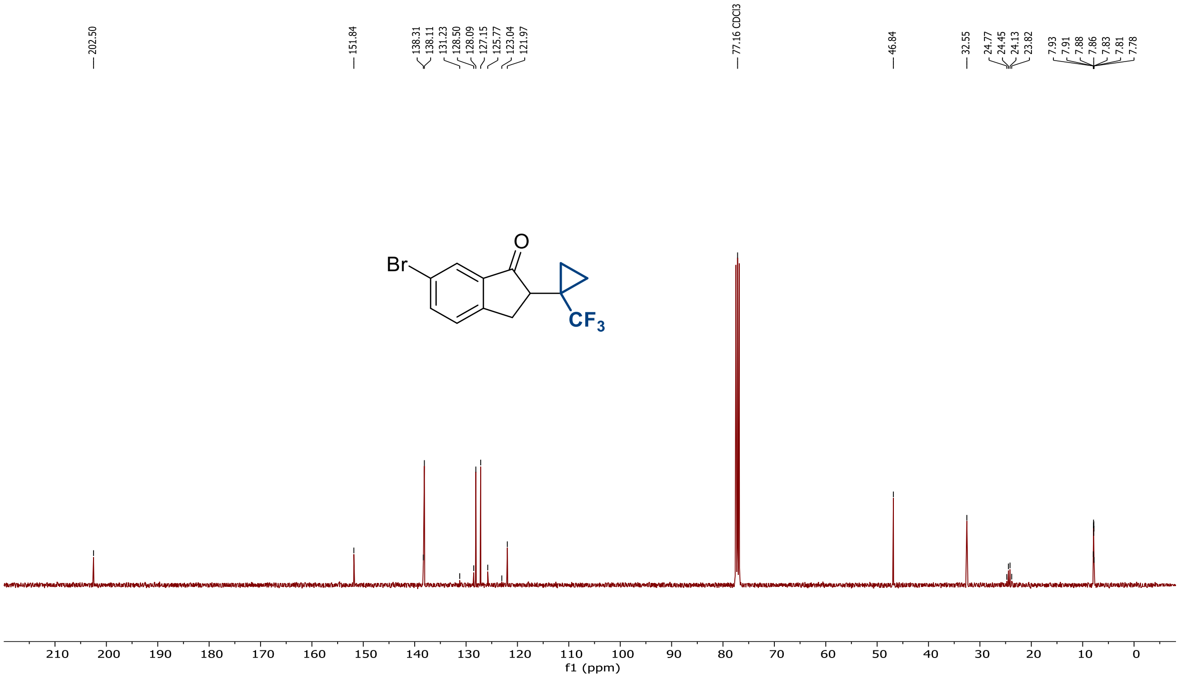

$^{19}F$ NMR (377 MHz, $CDCl_3$)

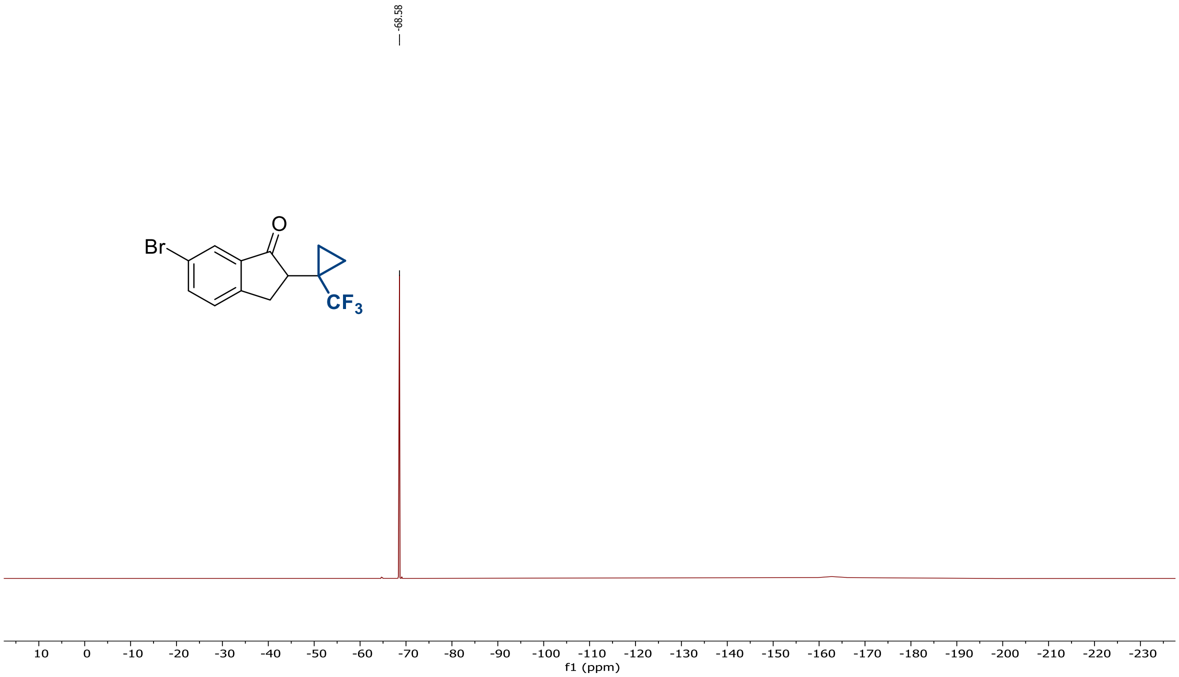


Compound **4f**:

$^{1}H$ NMR (400 MHz, $CDCl_3$)

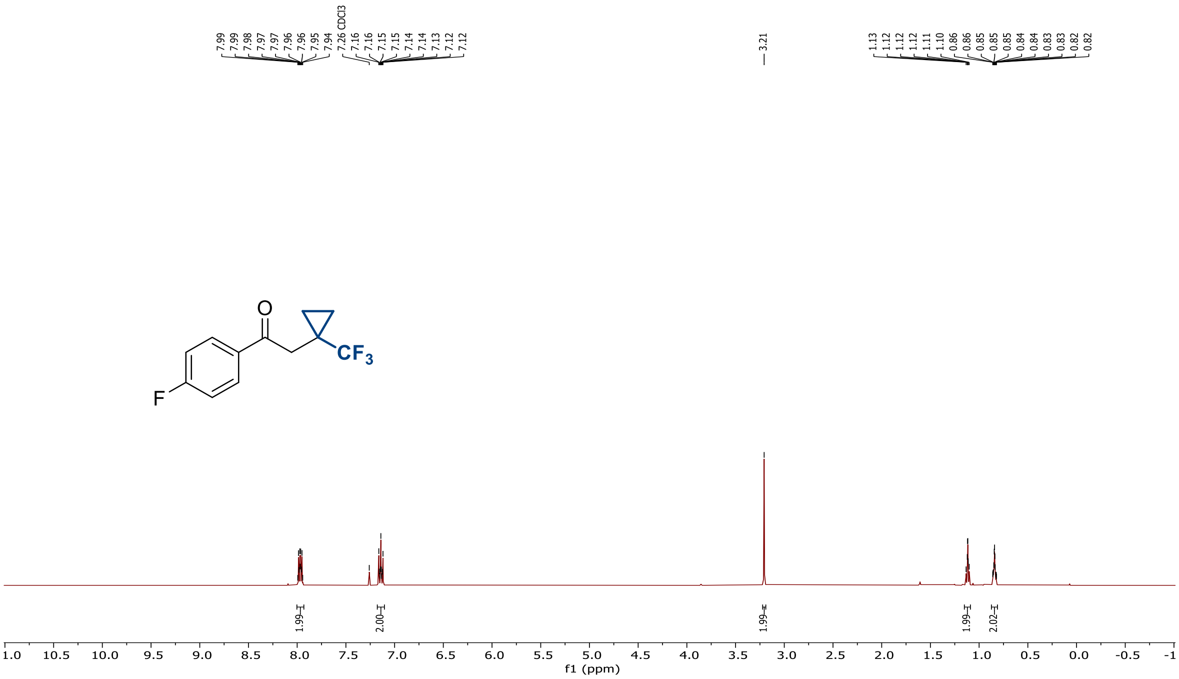

$^{13}C\{1H\}$ NMR (101 MHz, $CDCl_3$)

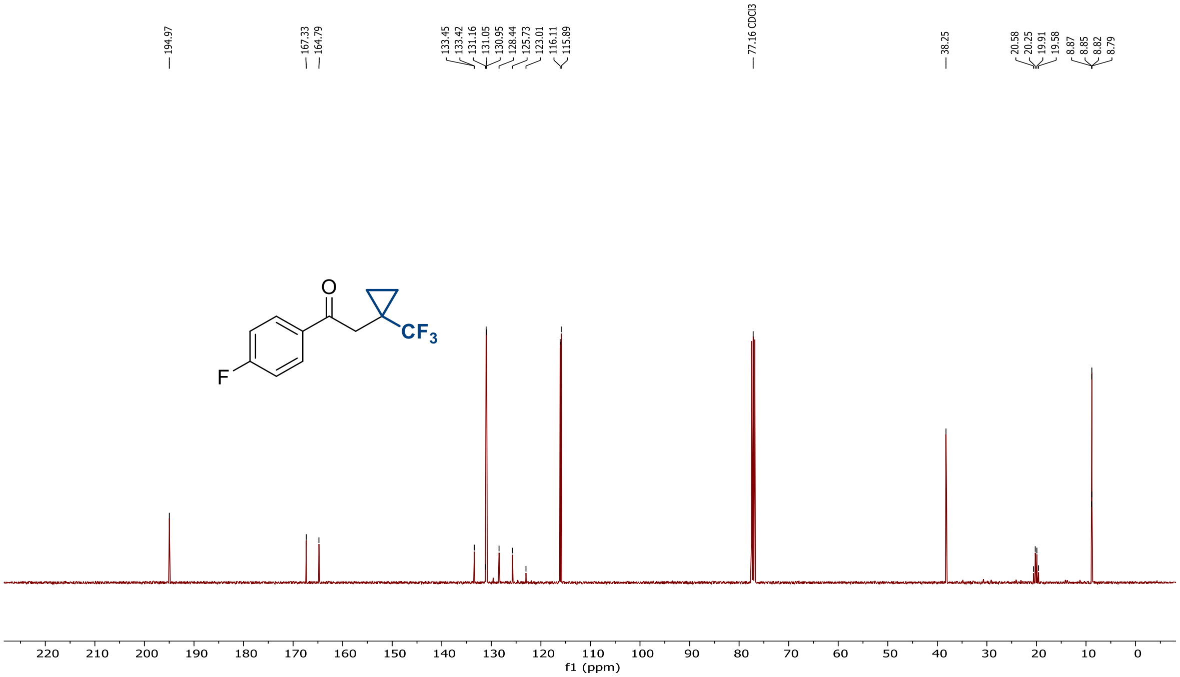


$^{19}F$ NMR (377 MHz, $CDCl_3$)

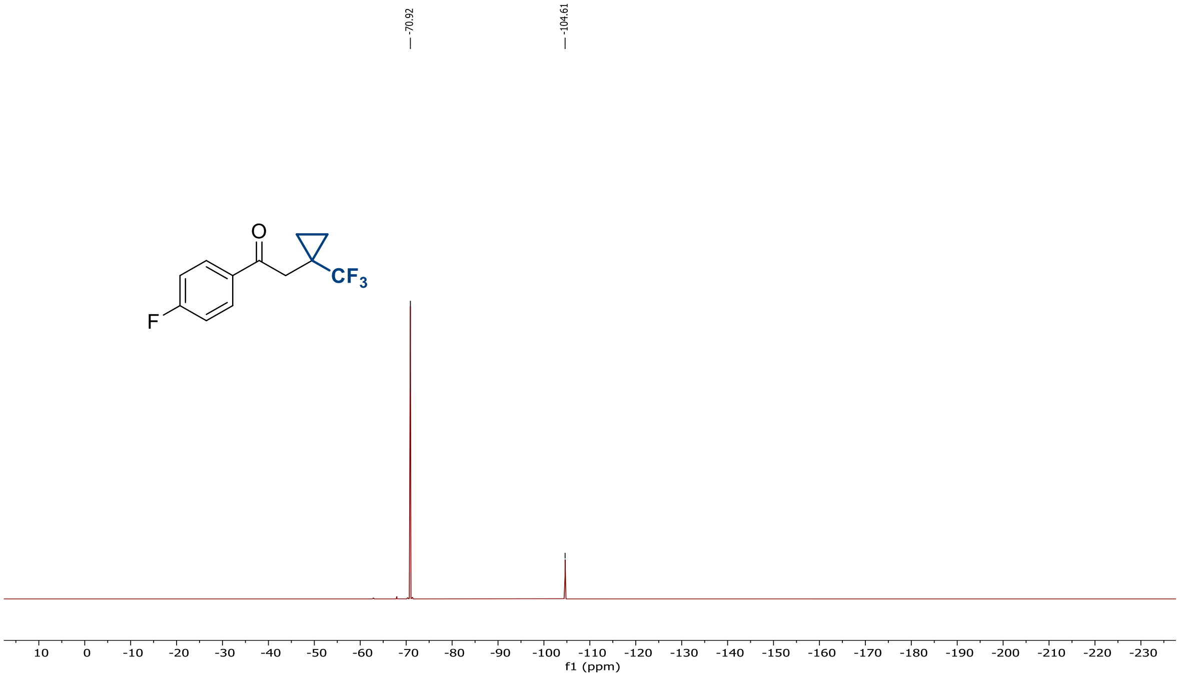

Compound **4g**:

$^{1}H$ NMR (300 MHz, $CDCl_3$)

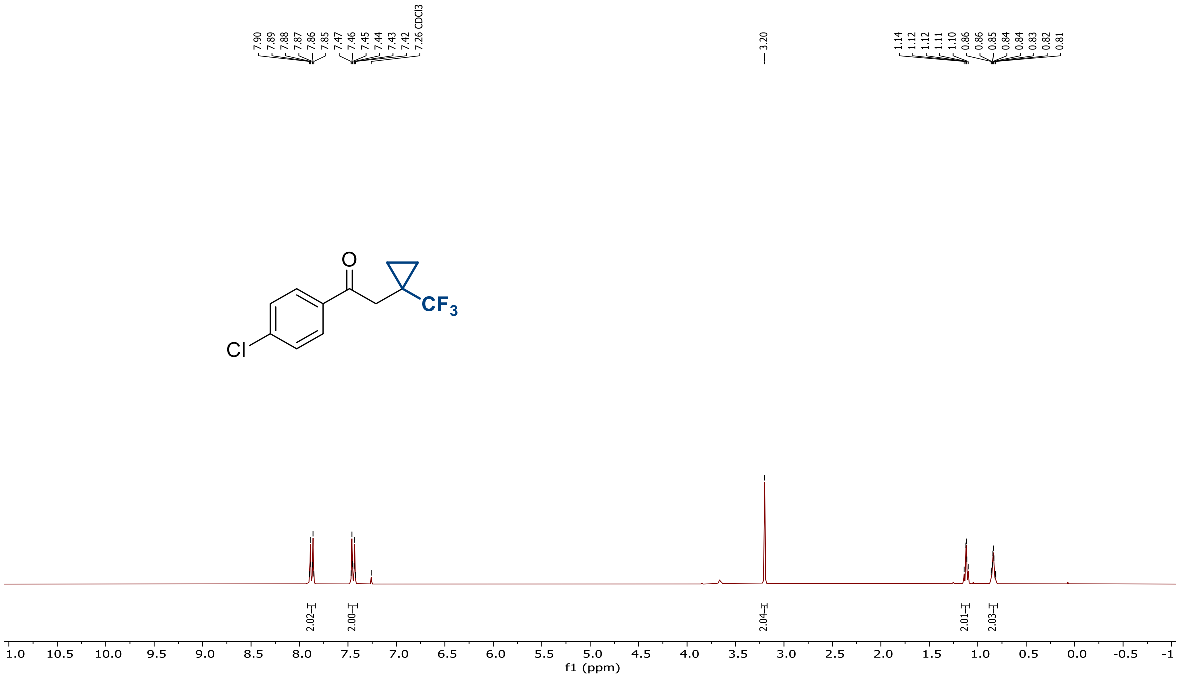


$^{13}C\{1H\}$ NMR (101 MHz, $CDCl_3$)

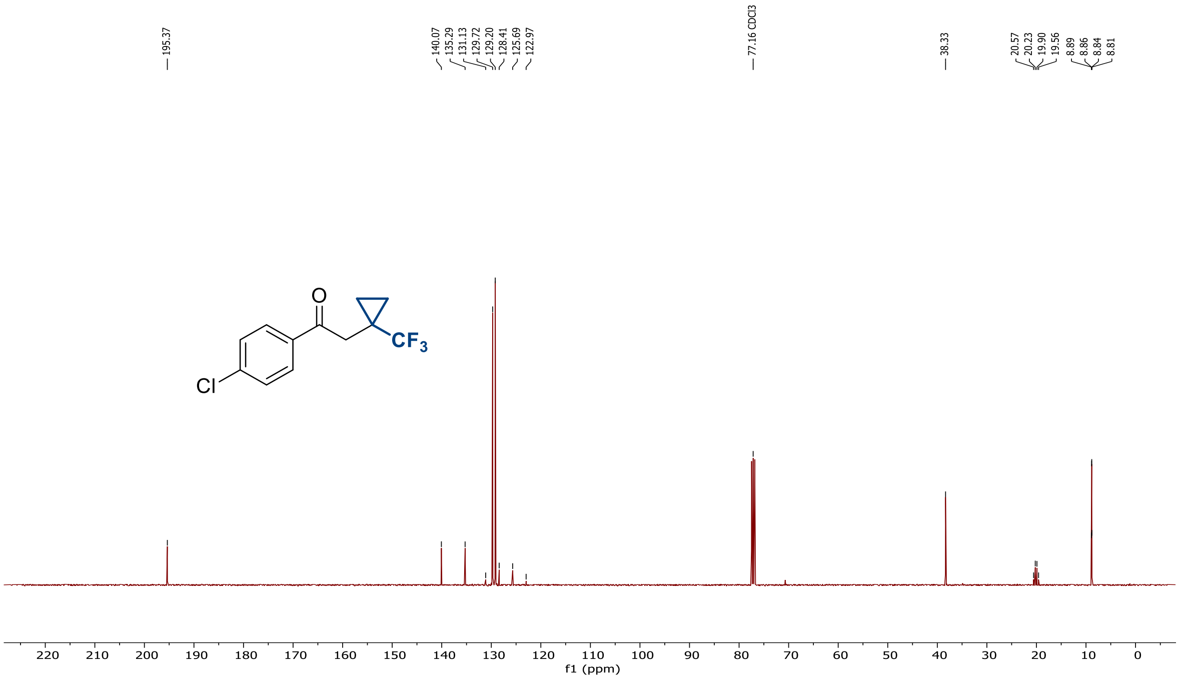

$^{19}$F NMR (282 MHz, $CDCl_3$)

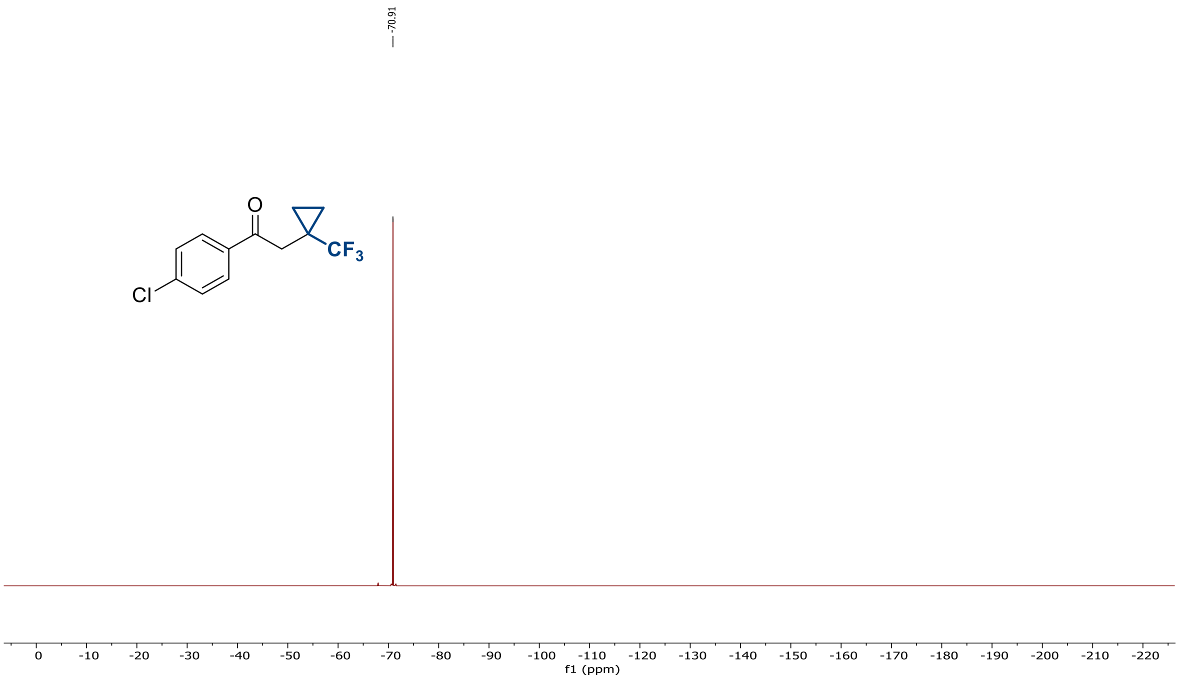


Compound **4h**:

$^{1}$H NMR (400 MHz, $CDCl_3$)

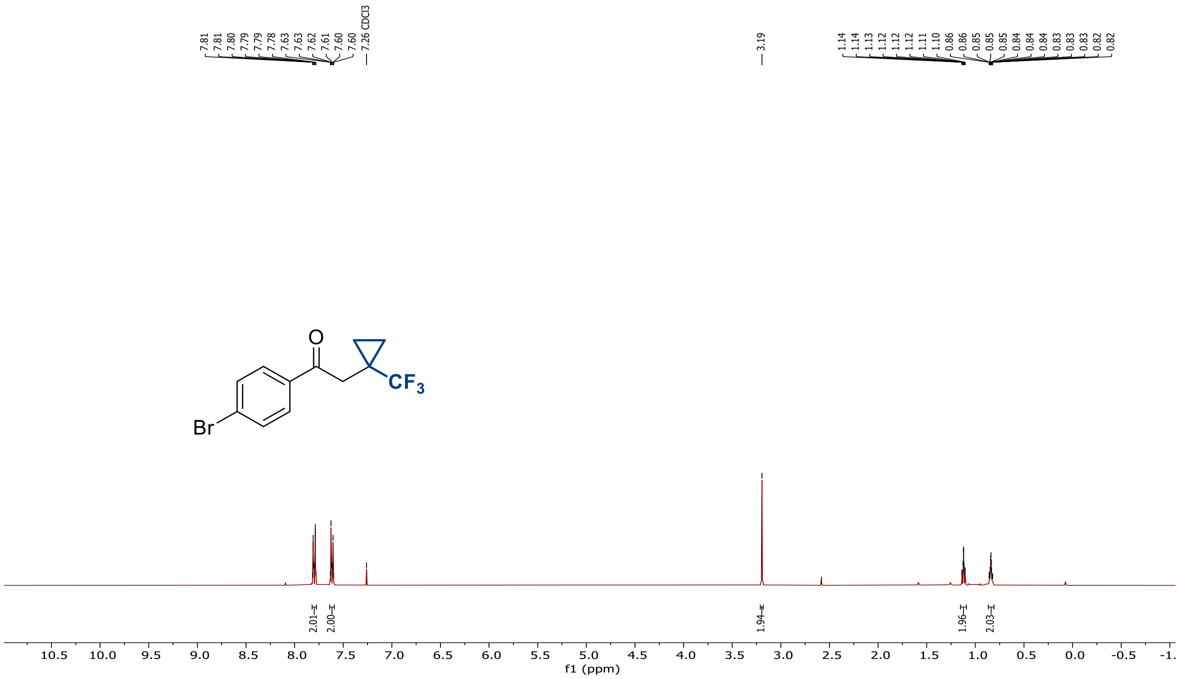

$^{13}C\{1H\}$ NMR (101 MHz, $CDCl_3$)

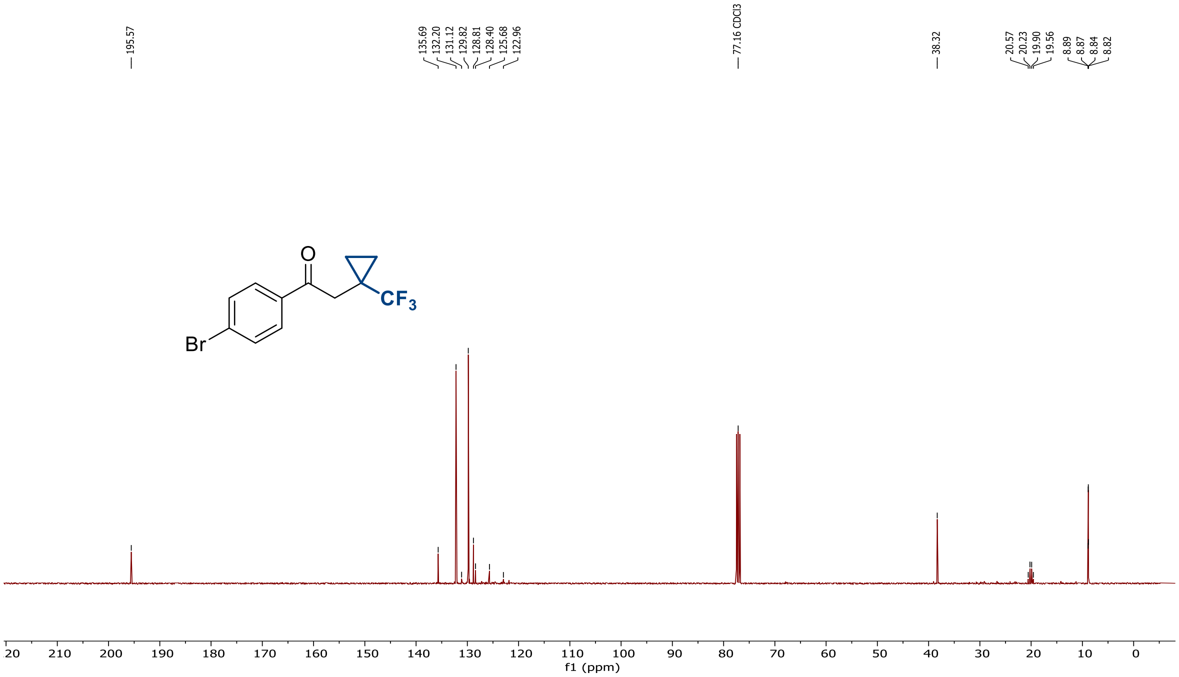


$^{19}F$ NMR (377 MHz, $CDCl_3$)

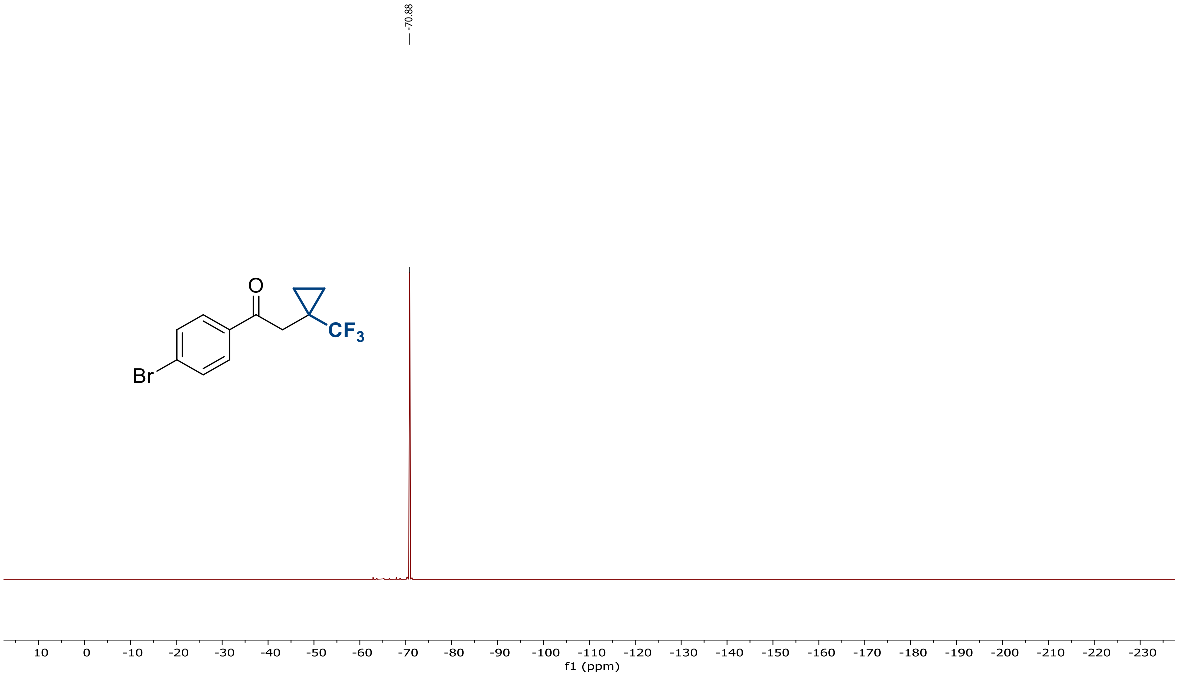

Compound **4i**:

$^1$H NMR (400 MHz, $CDCl_3$)

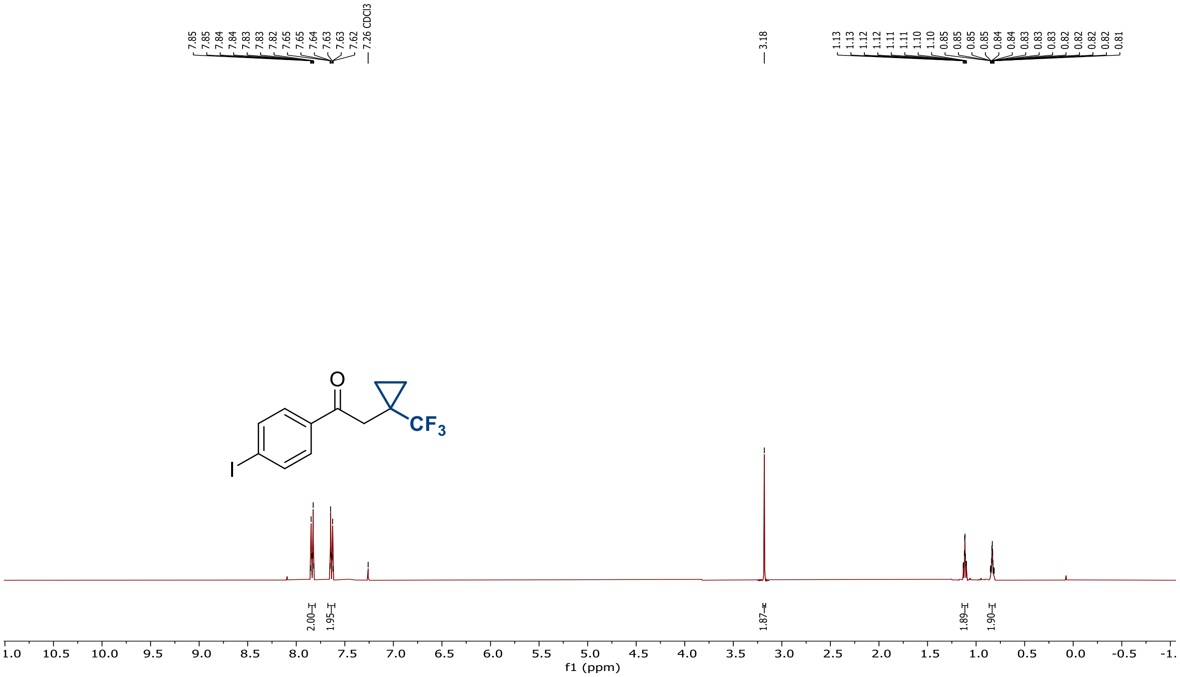


$^{13}$C{1H} NMR (101 MHz, $CDCl_3$)

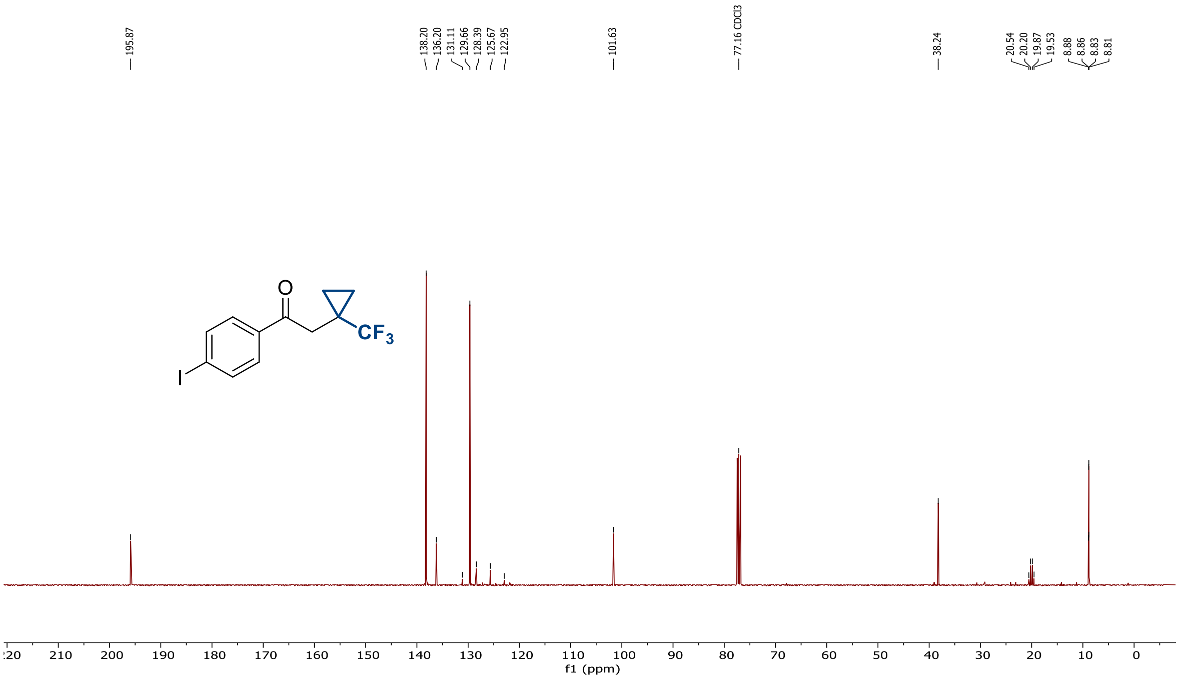

$^{19}F$ NMR (377 MHz, $CDCl_3$)

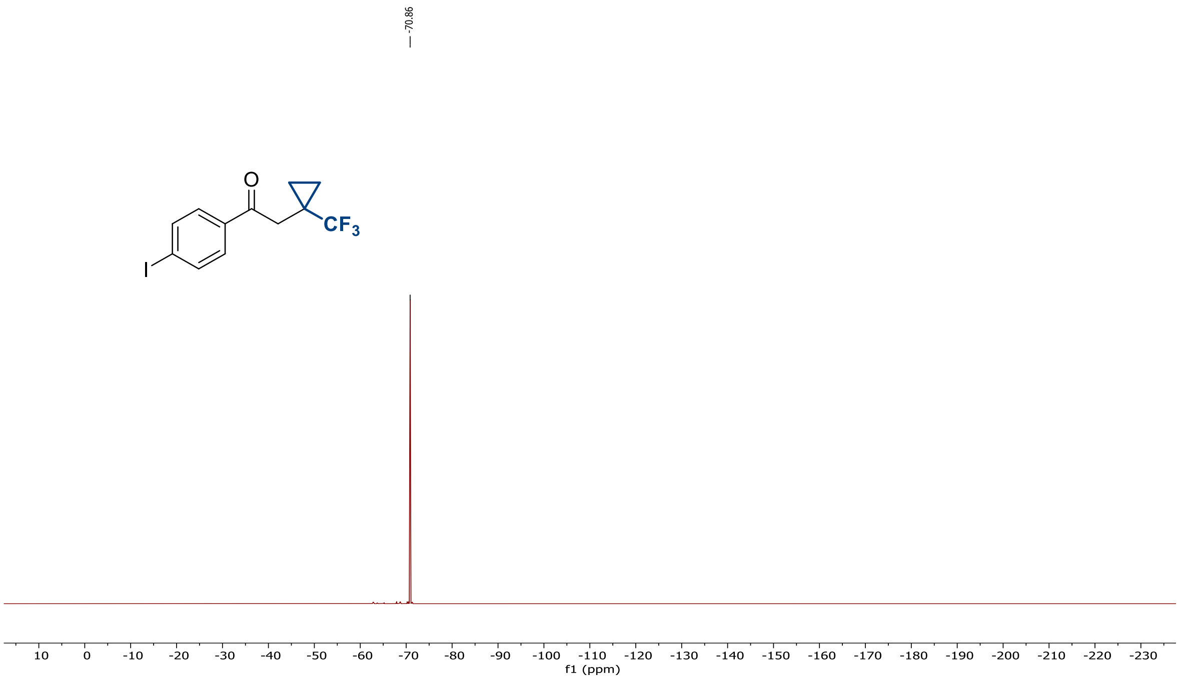


Compound **4j**:

$^{1}H$ NMR (400 MHz, $CDCl_3$)

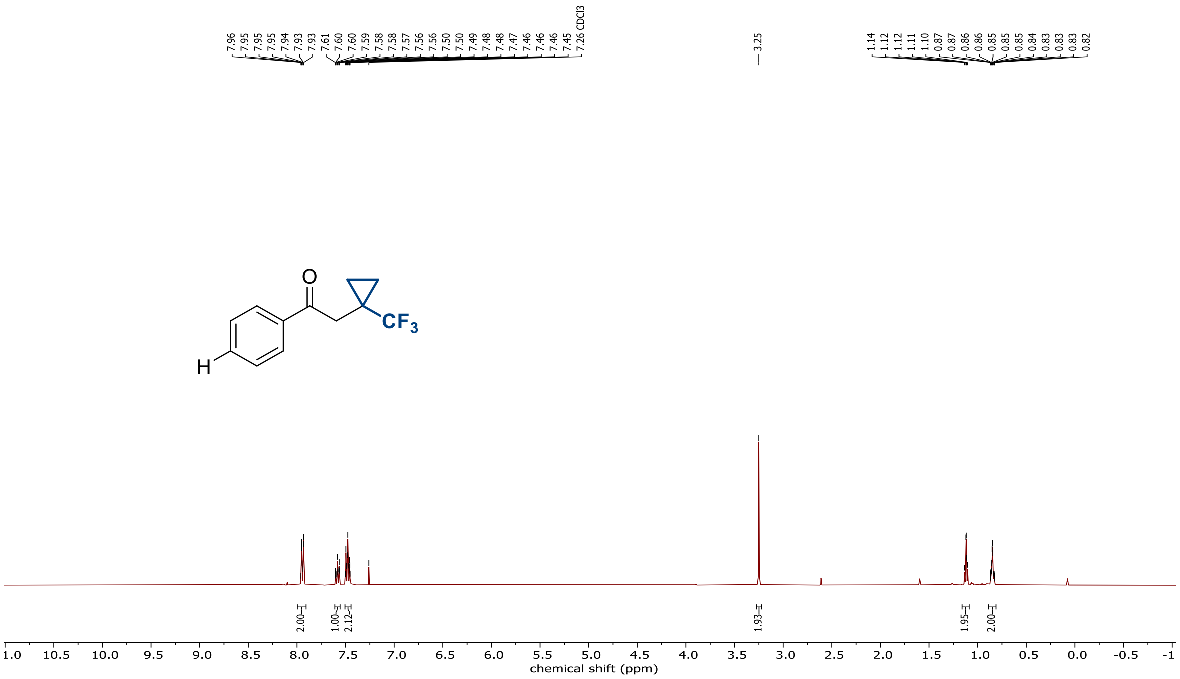

## $^{13}C\{1H\}$ NMR (101 MHz, $CDCl_3$)

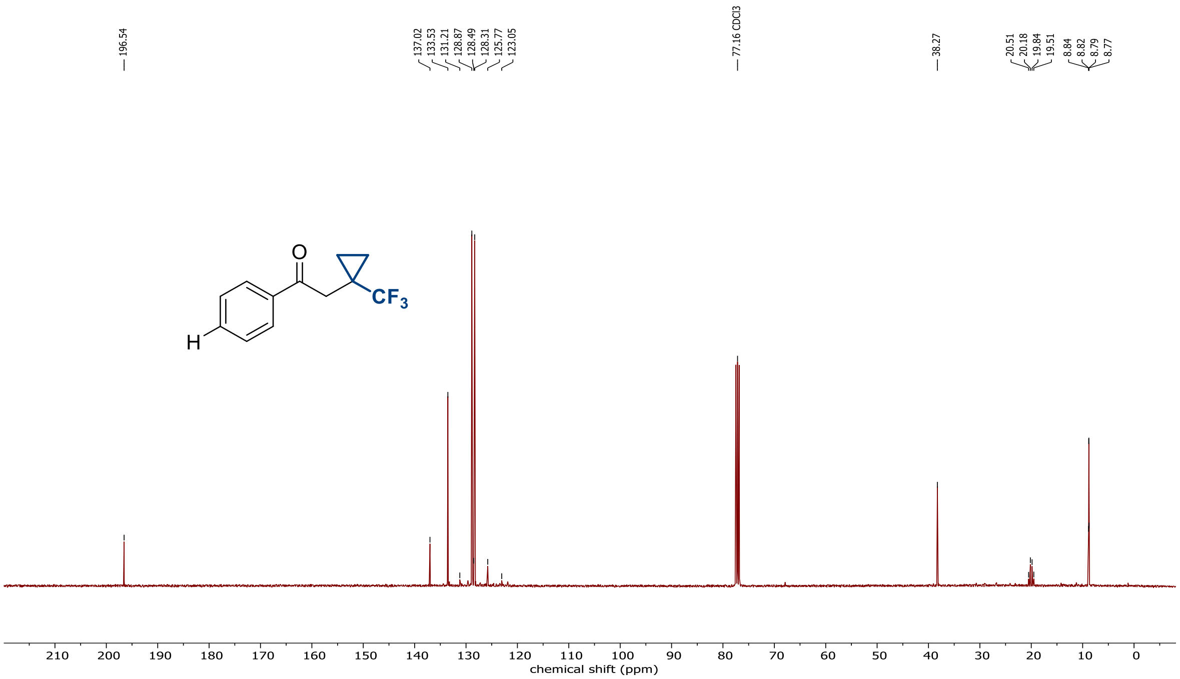


## $^{19}F$ NMR (377 MHz, $CDCl_3$)

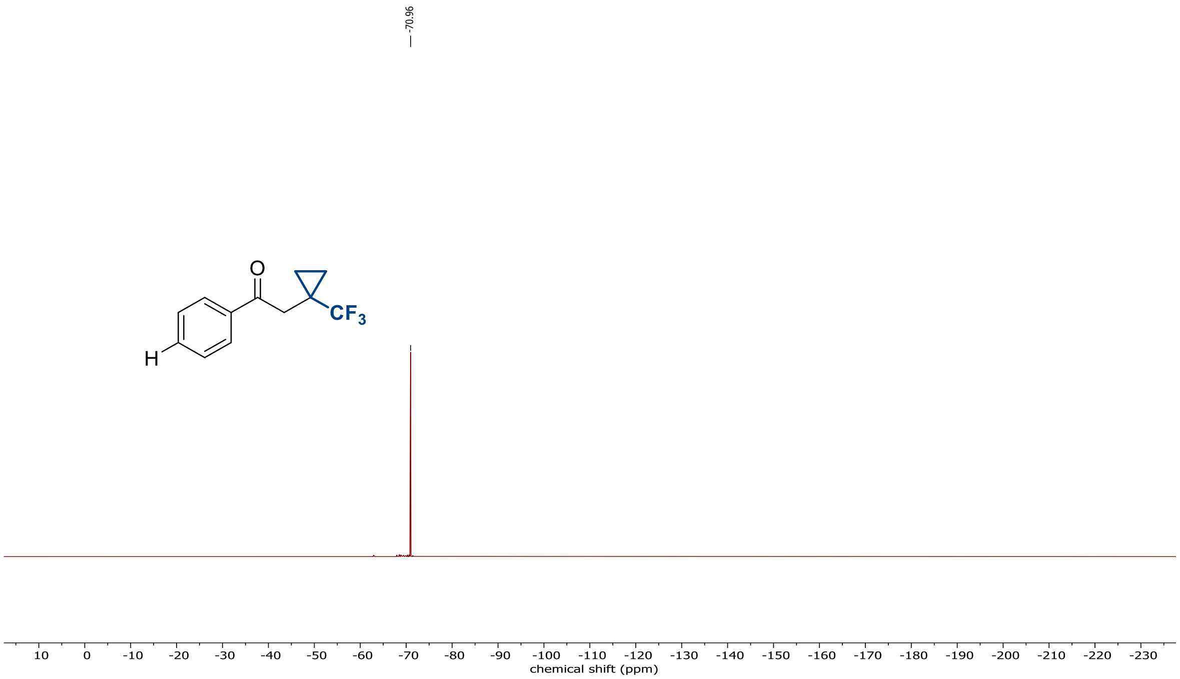

Compound **4k**:

$^{1}H$ NMR (400 MHz, $CDCl_3$)

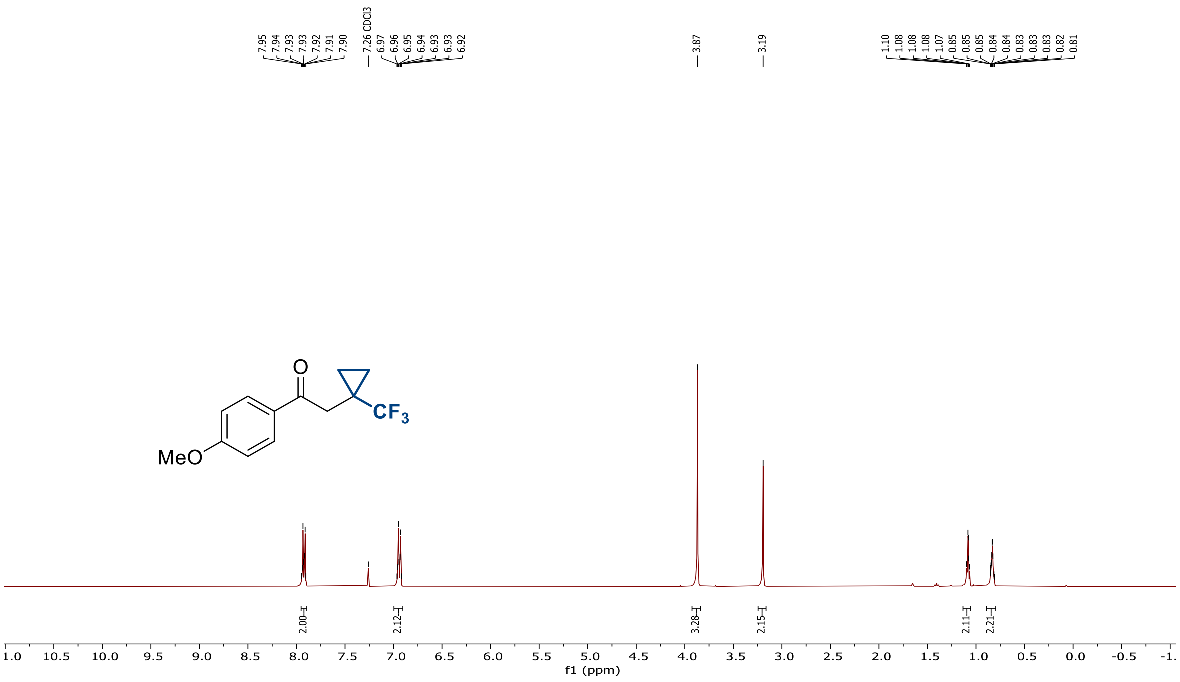

$^{13}C\{1H\}$ NMR (101 MHz, $CDCl_3$)

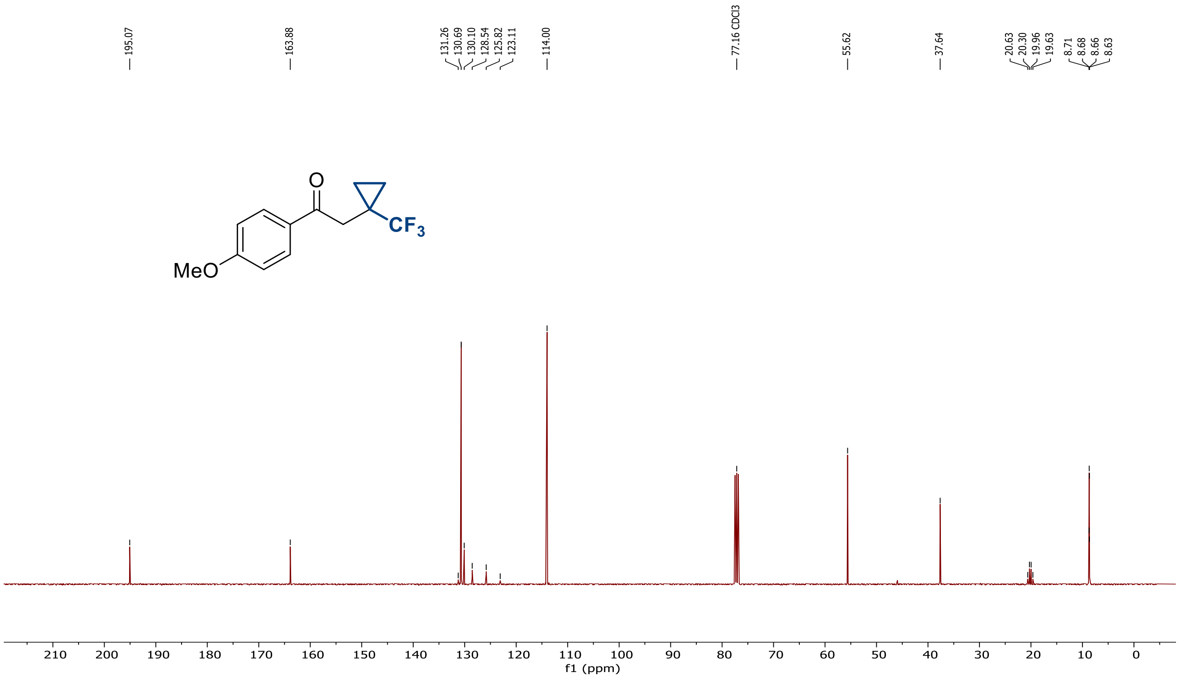

$^{19}F$ NMR (377 MHz, $CDCl_3$)

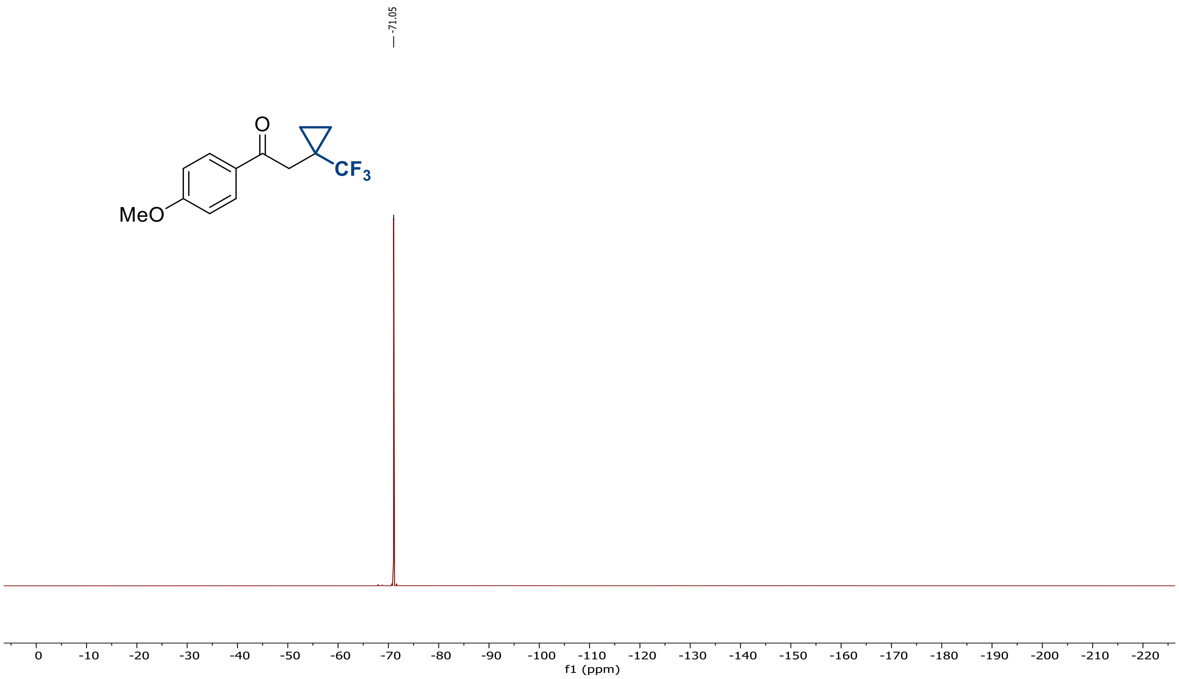


Compound **4l**:

$^{1}H$ NMR (400 MHz, $CDCl_3$)

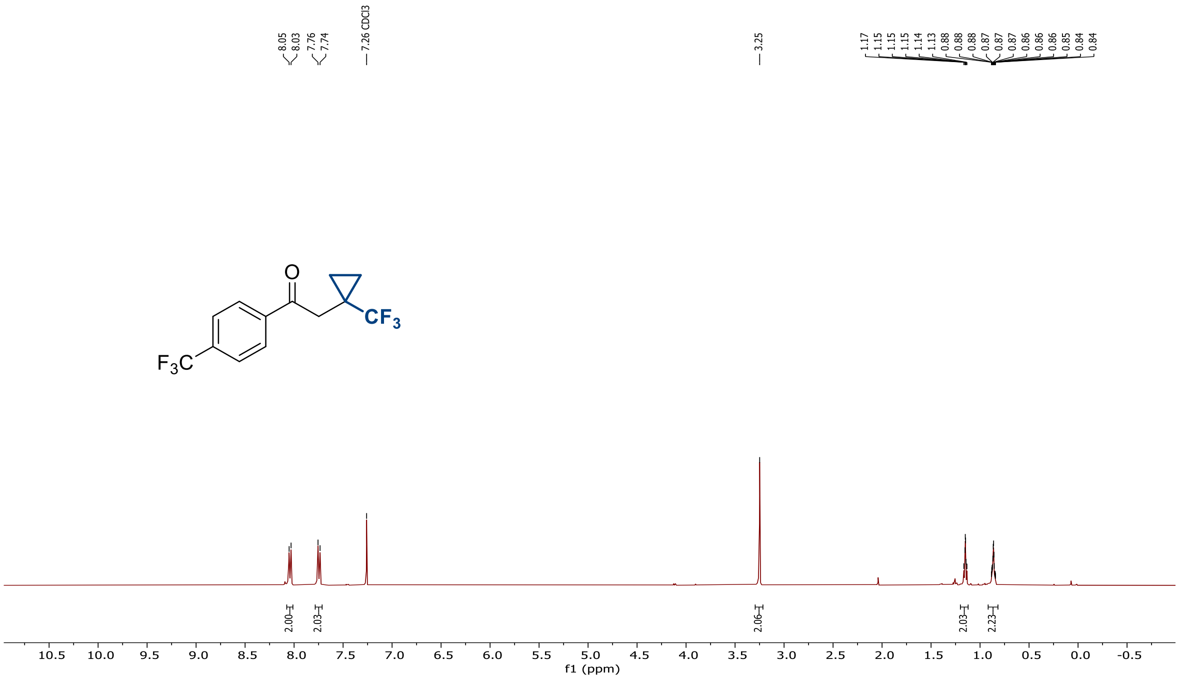

$^{13}C\{^1H\}$ NMR (101 MHz, $CDCl_3$)

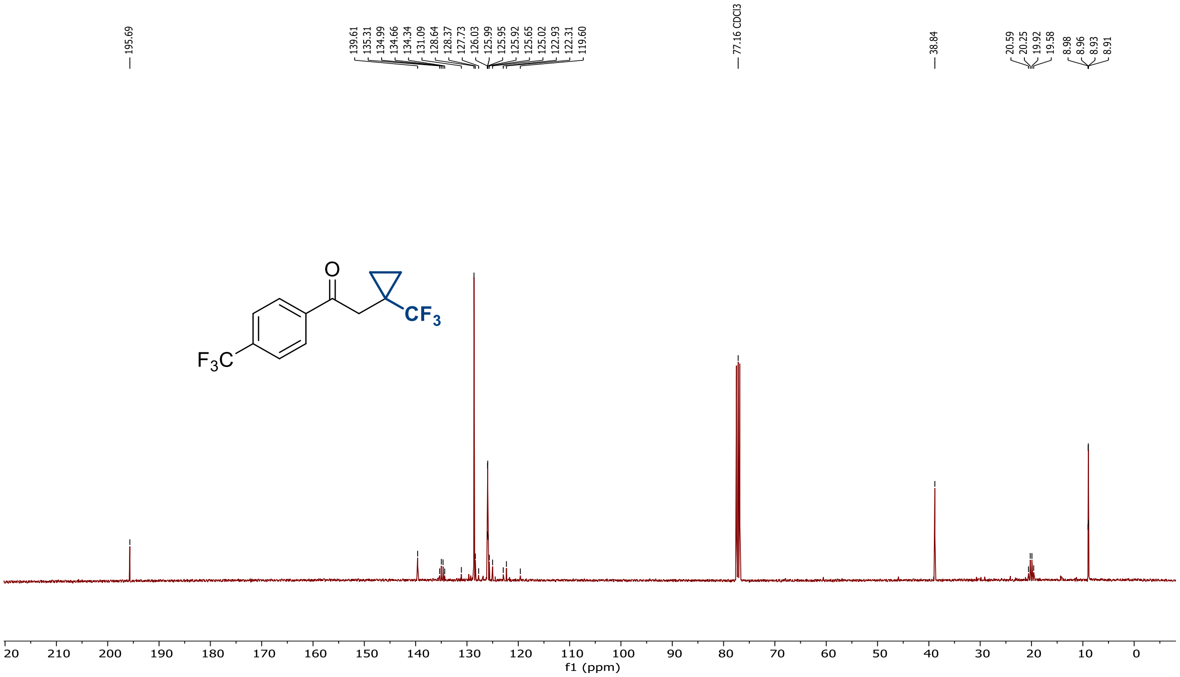

$^{19}F$ NMR (377 MHz, $CDCl_3$)

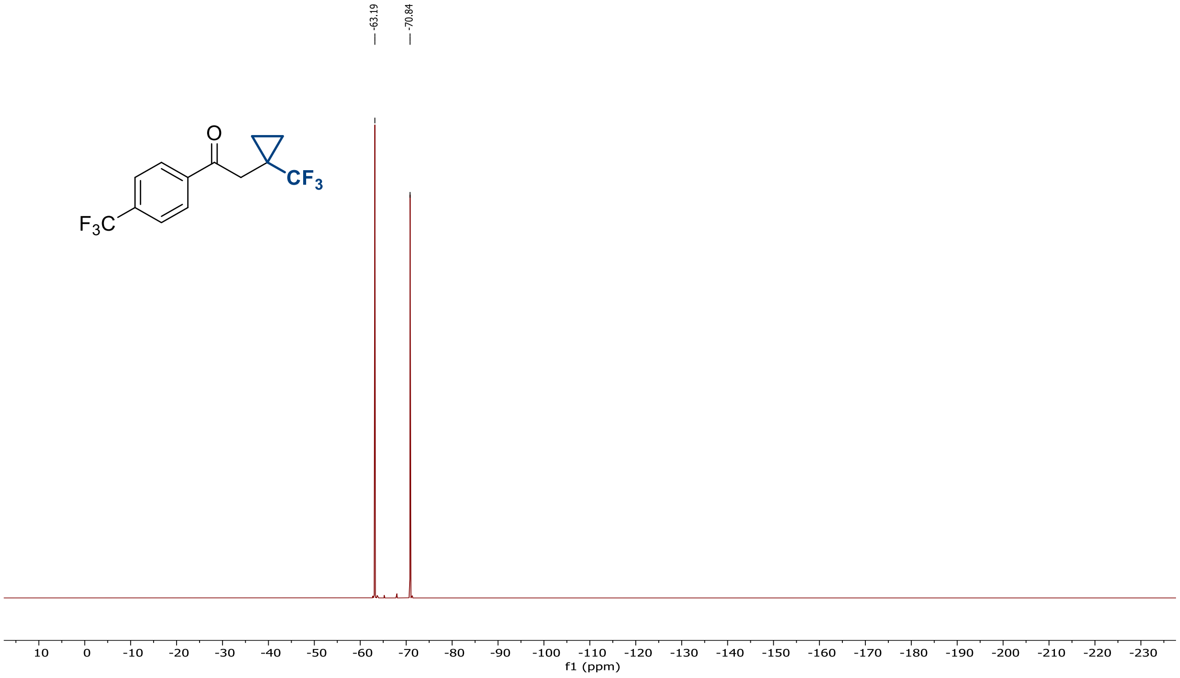

Compound **4m**:

$^{1}H$ NMR (400 MHz, $CDCl_3$)

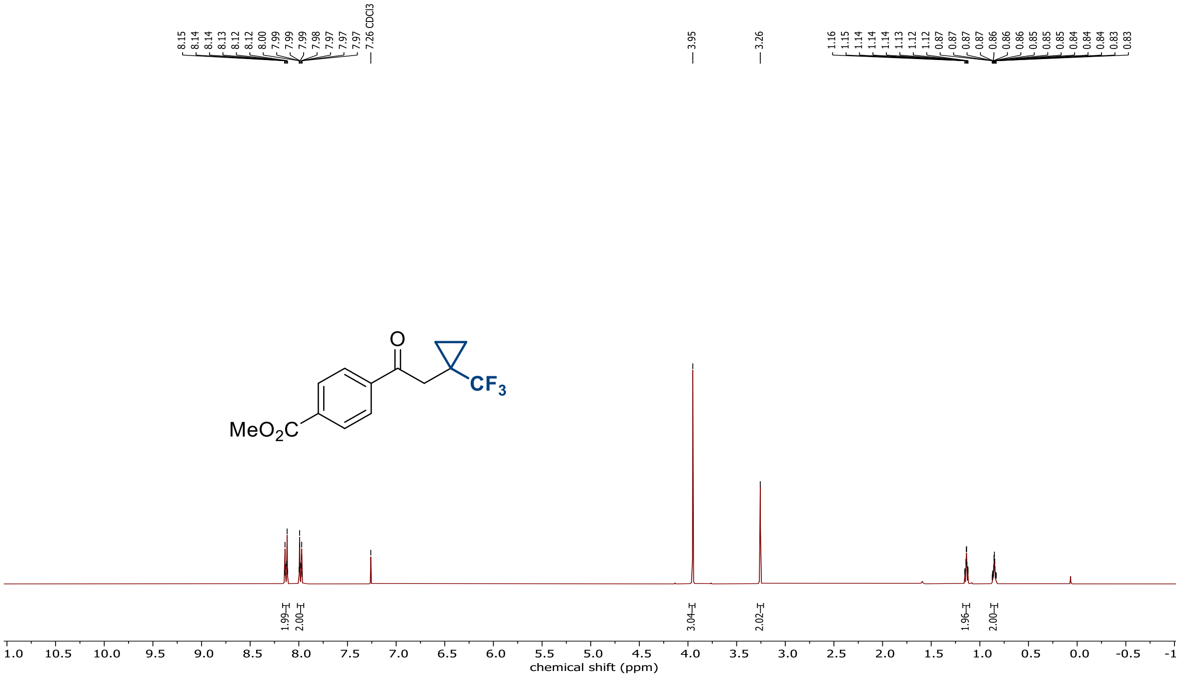


$^{13}C\{1H\}$ NMR (101 MHz, $CDCl_3$)

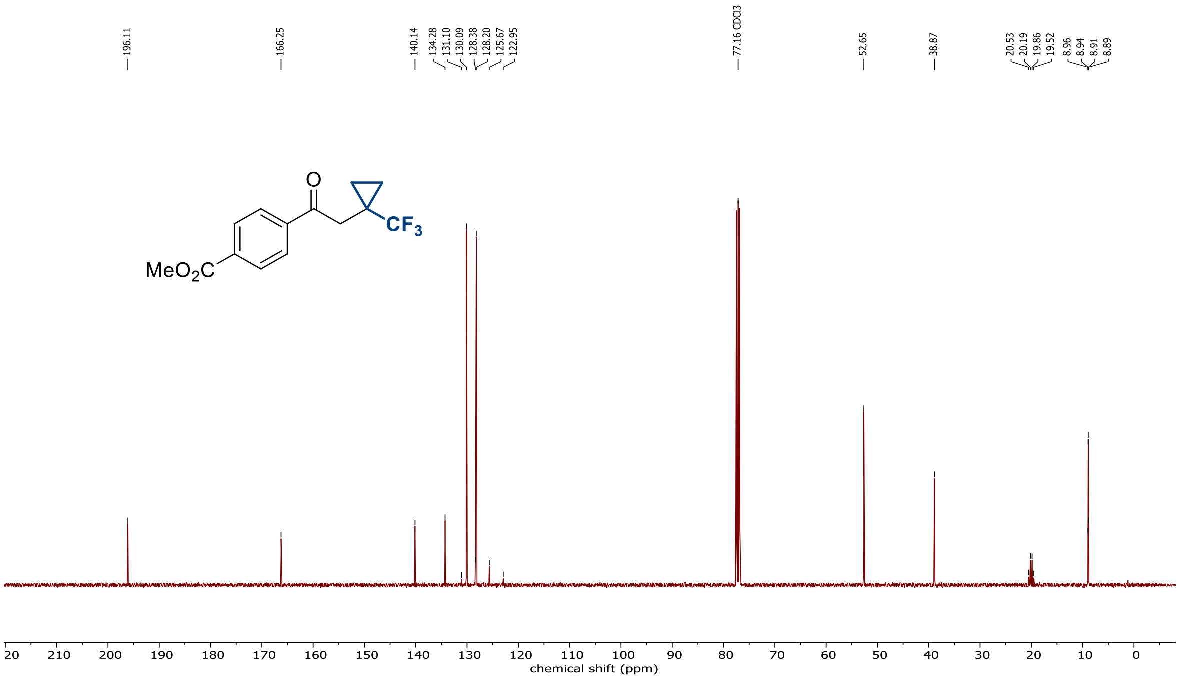

$^{19}F$ NMR (282 MHz, $CDCl_3$)

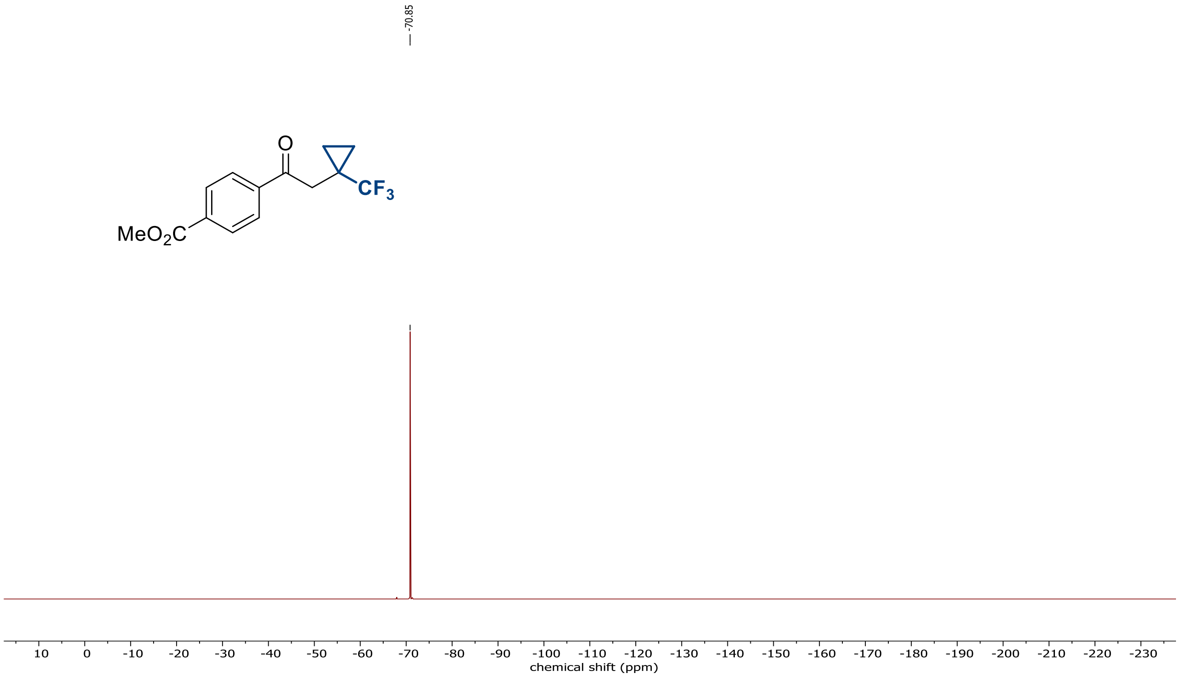


Compound **4n**:

$^{1}H$ NMR (300 MHz, $CDCl_3$)

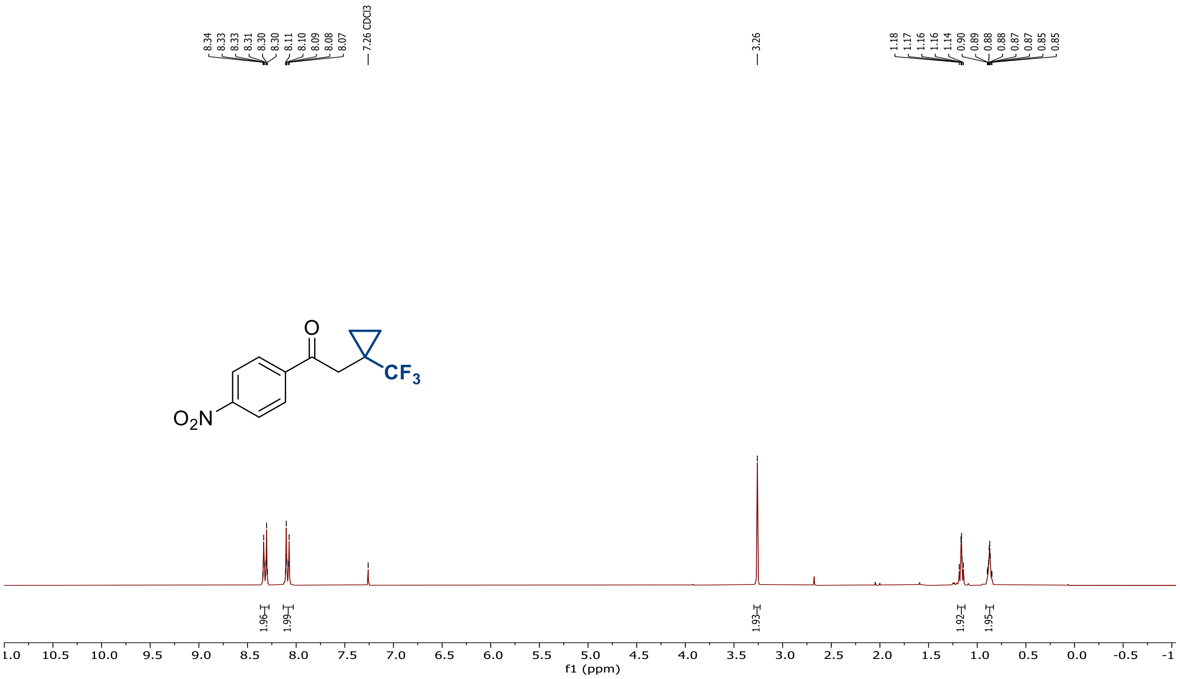

$^{13}C\{^1H\}$ NMR (101 MHz, $CDCl_3$)

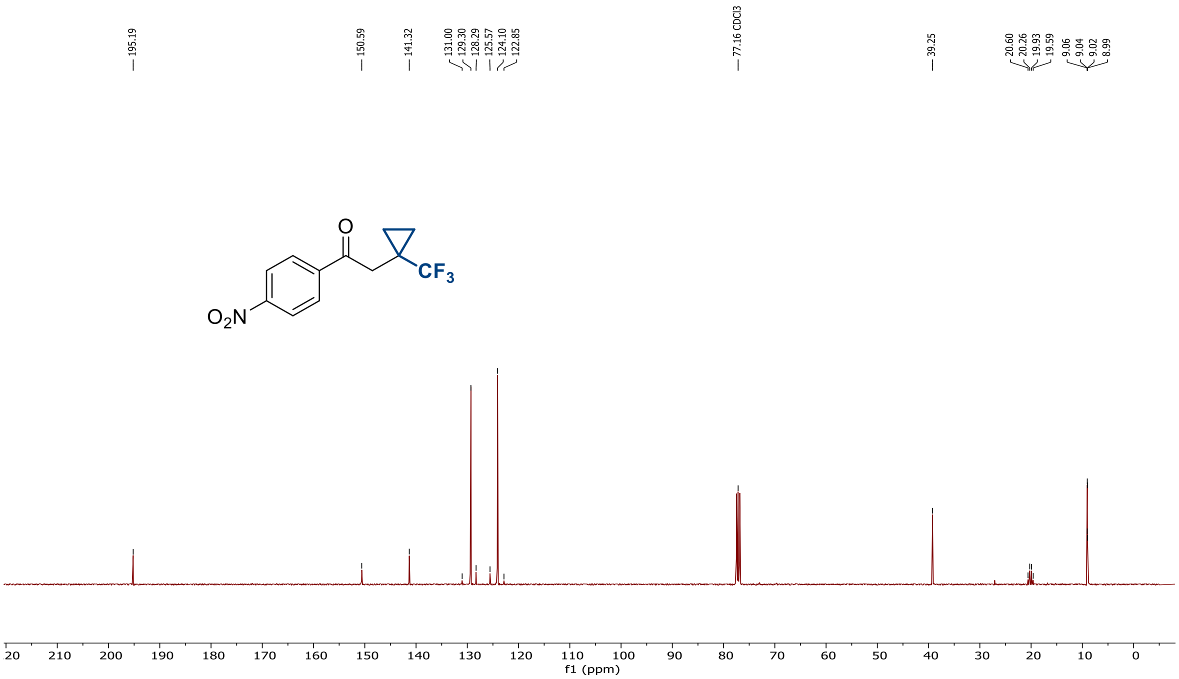


$^{19}F$ NMR (282 MHz, $CDCl_3$)

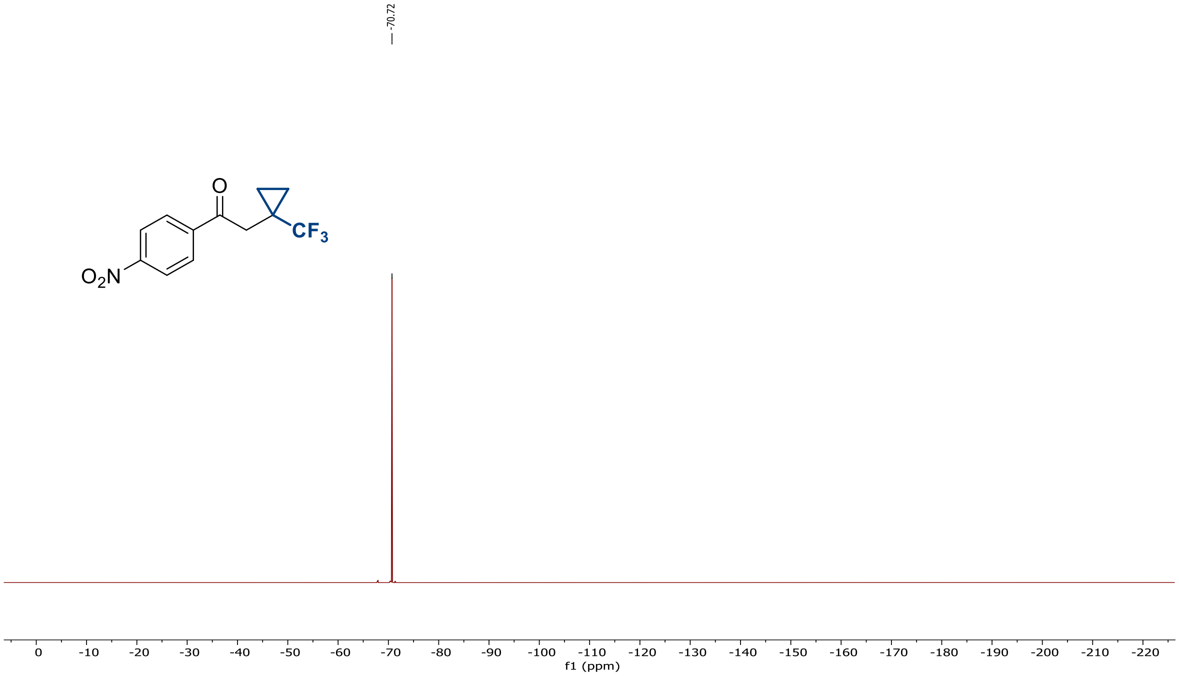

Compound **4o**:

$^{1}H$ NMR (400 MHz, $CDCl_3$)

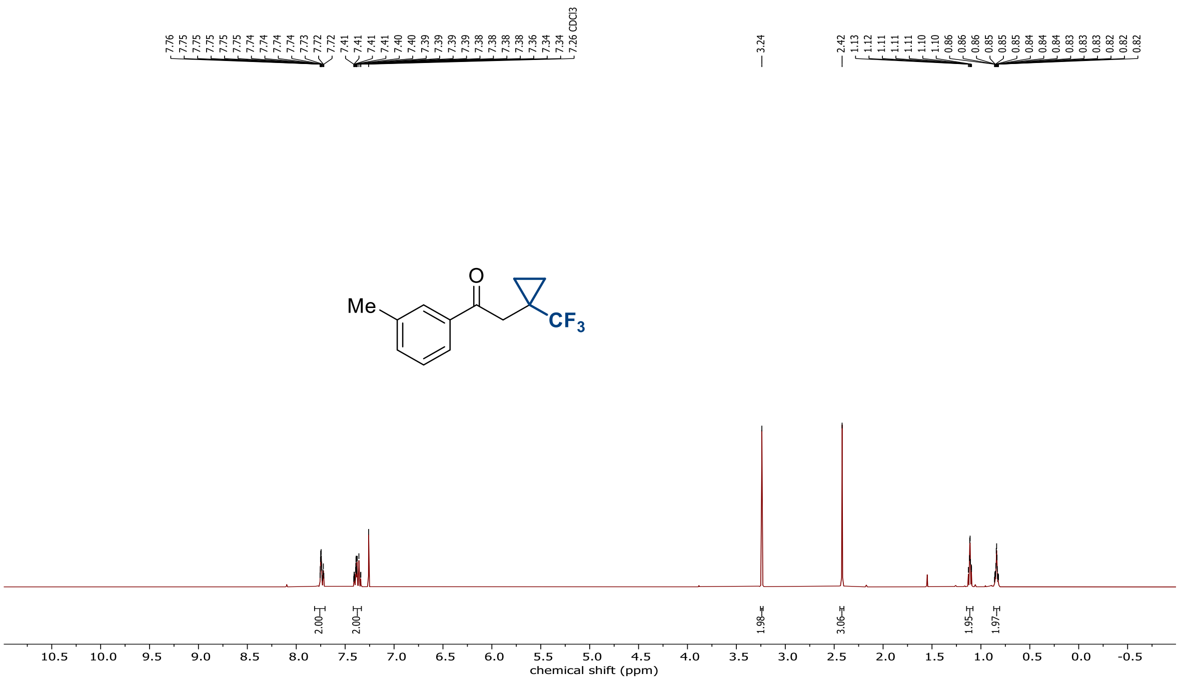


$^{13}C\{1H\}$ NMR (101 MHz, $CDCl_3$)

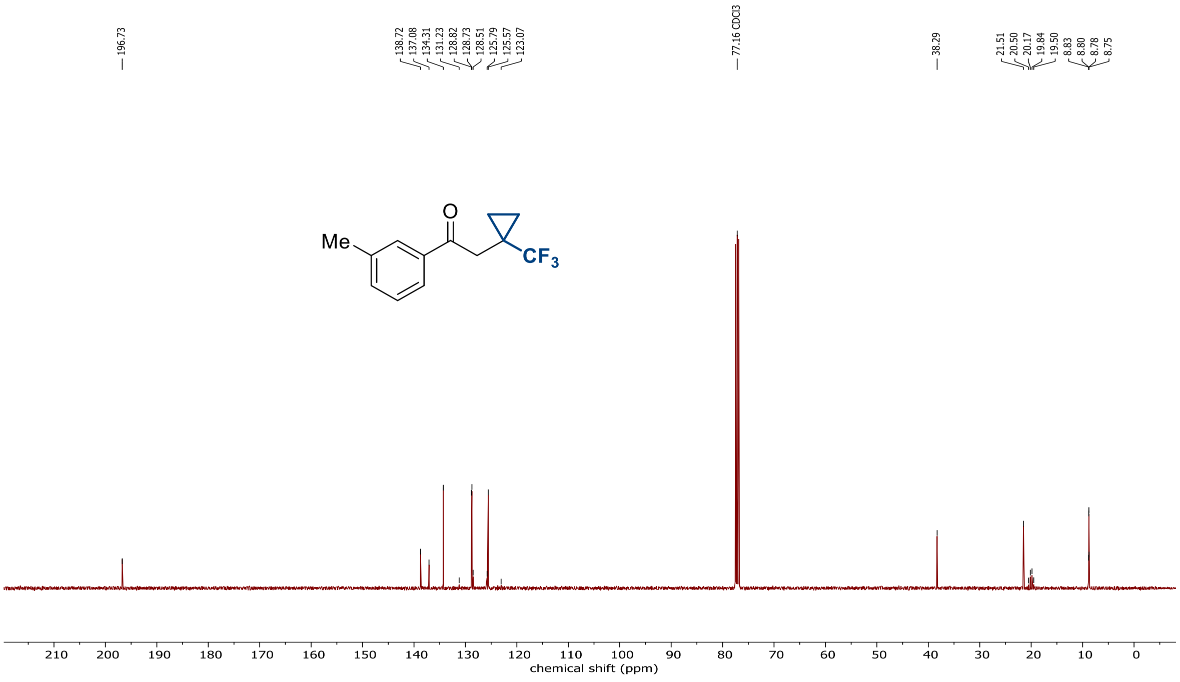

$^{19}F$ NMR (377 MHz, $CDCl_3$)

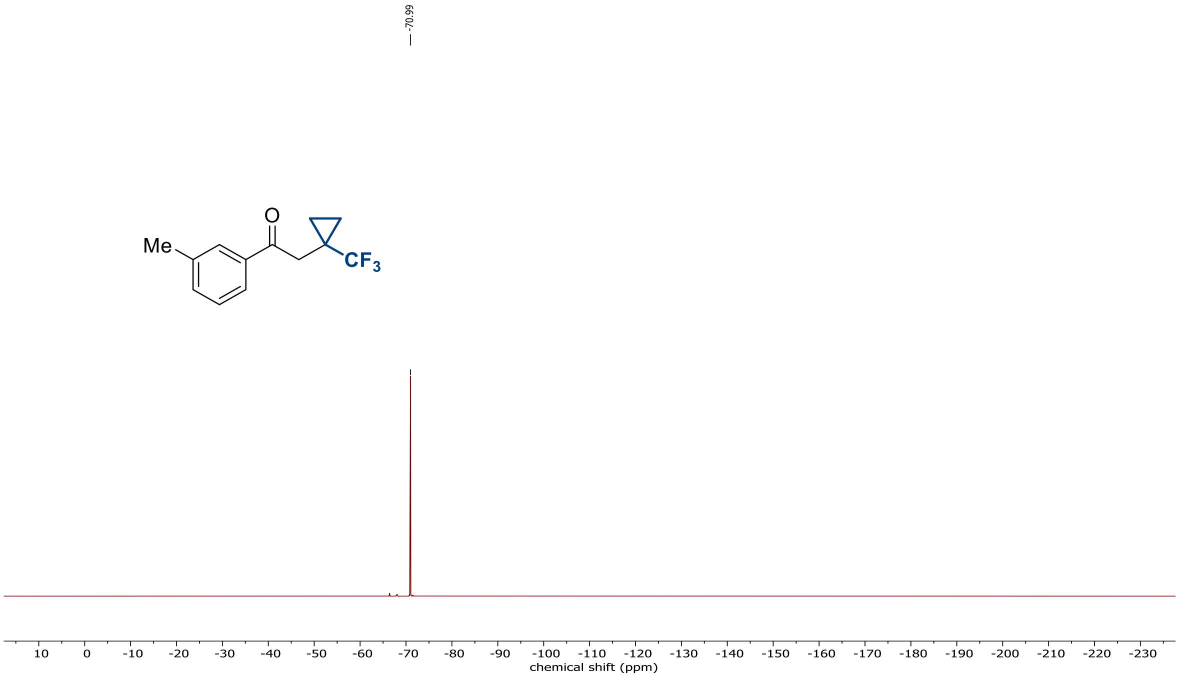


Compound **4p**:

$^{1}H$ NMR (400 MHz, $CDCl_3$)

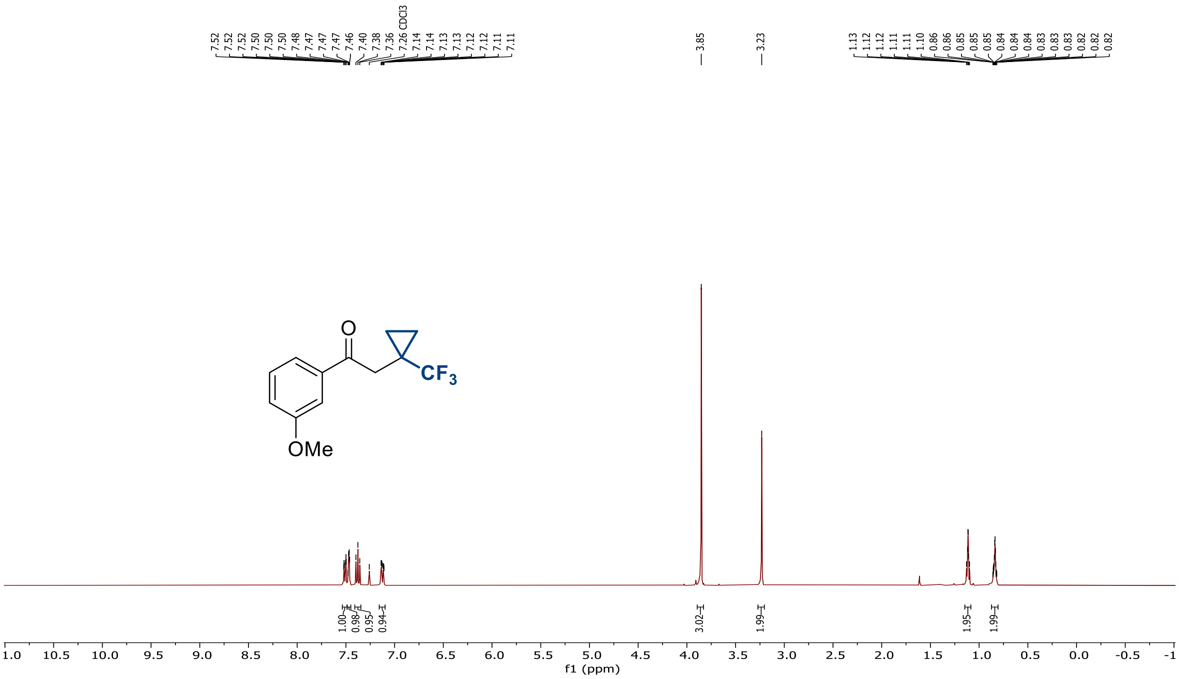

$^{13}C\{1H\}$ NMR (101 MHz, $CDCl_3$)

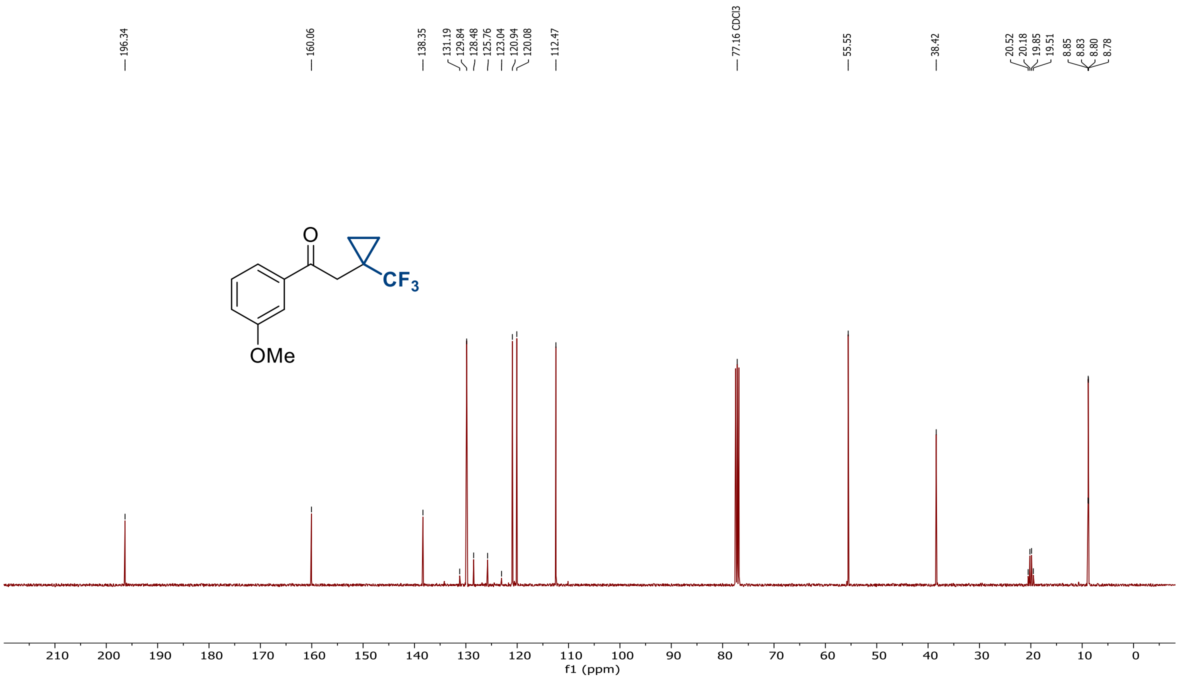


$^{19}F$ NMR (377 MHz, $CDCl_3$)

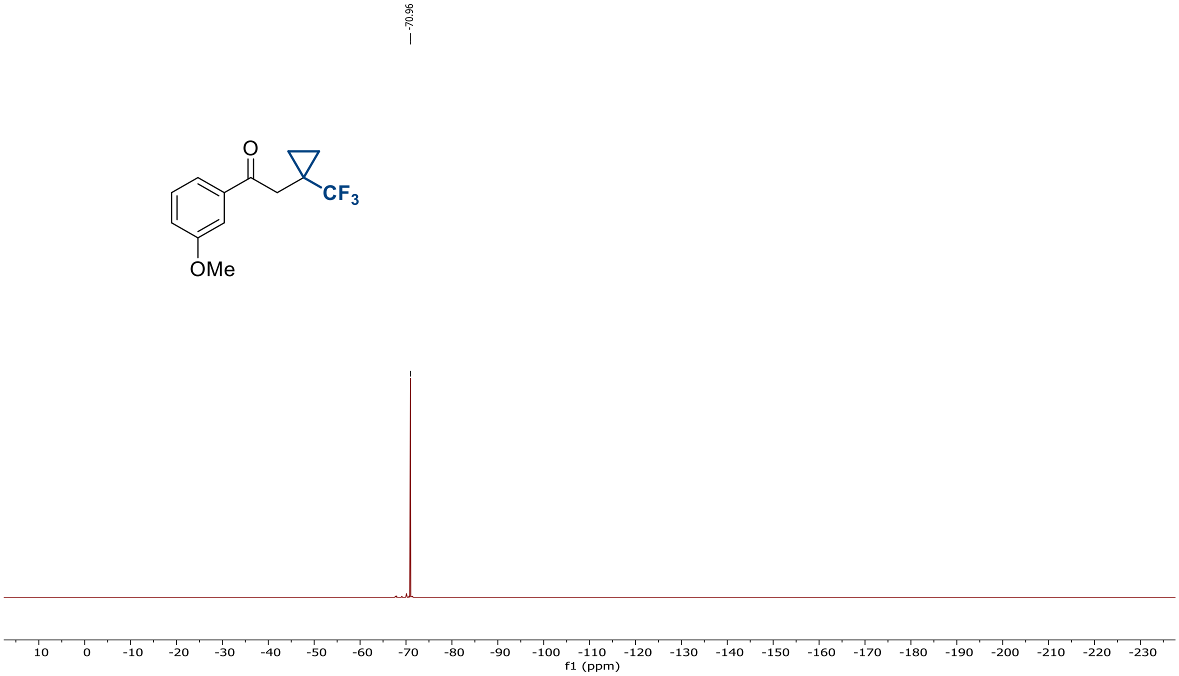

Compound **4q**:

$^1$H NMR (400 MHz, $CDCl_3$)

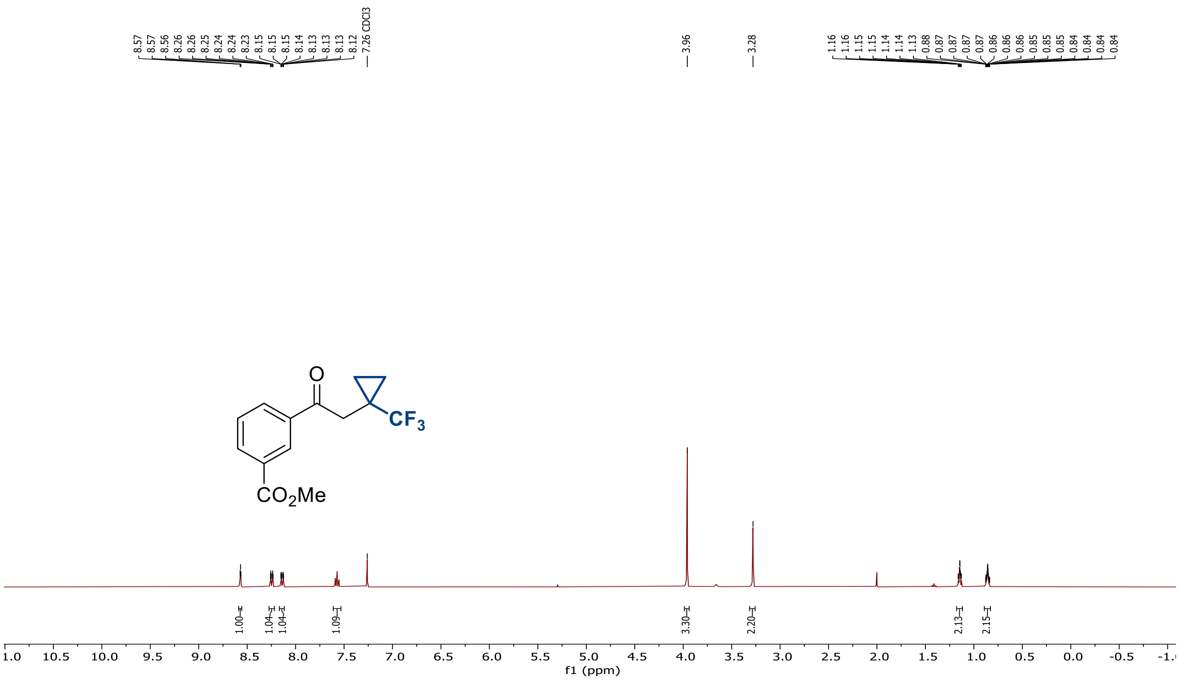


$^{13}$C{1H} NMR (101 MHz, $CDCl_3$)

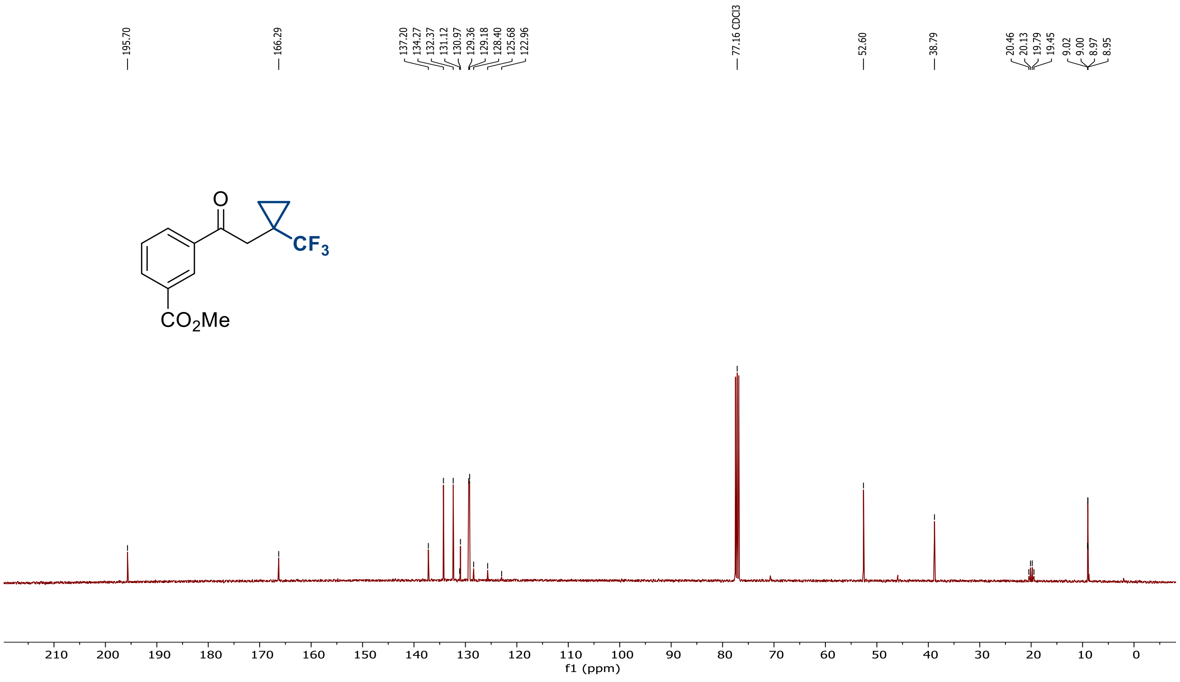

$^{19}F$ NMR (377 MHz, $CDCl_3$)

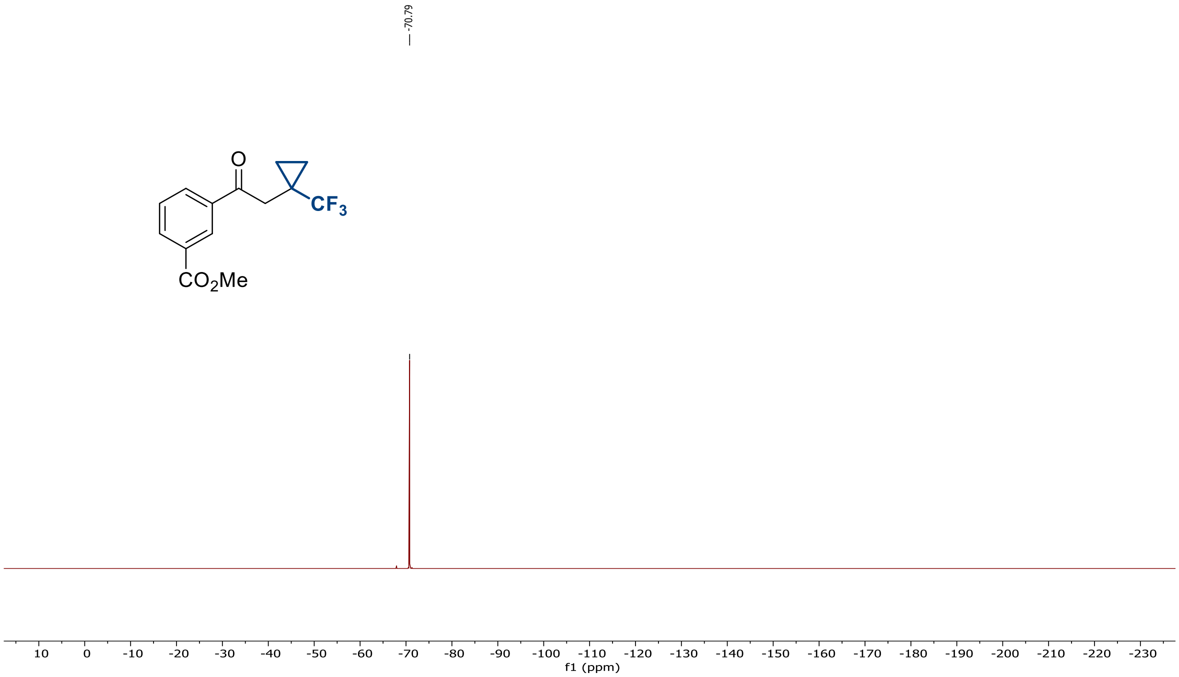


Compound **4r**:

$^{1}H$ NMR (300 MHz, $CDCl_3$)

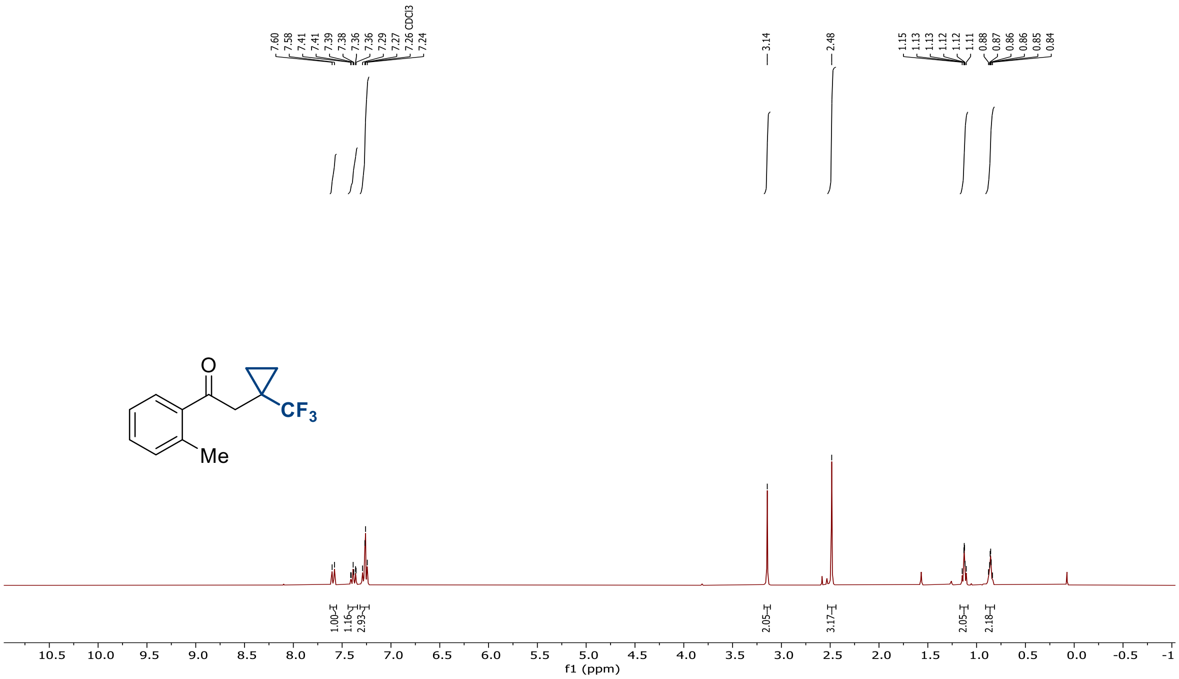

$^{13}C\{1H\}$ NMR (101 MHz, $CDCl_3$)

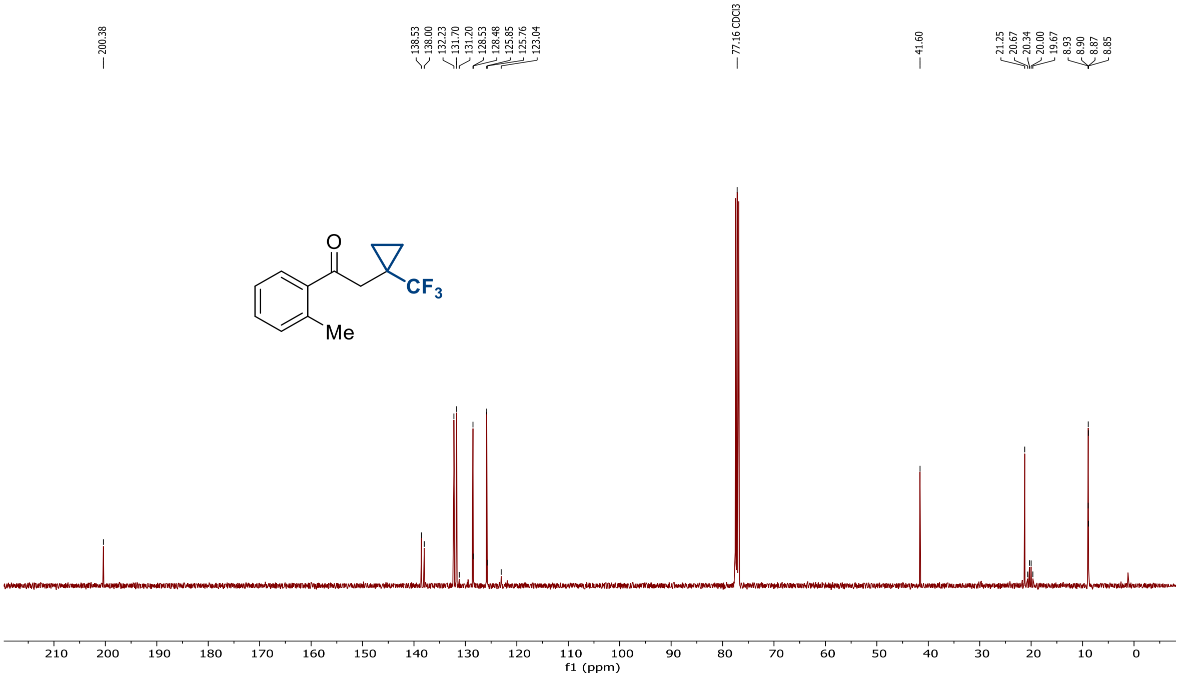

$^{19}F$ NMR (377 MHz, $CDCl_3$)

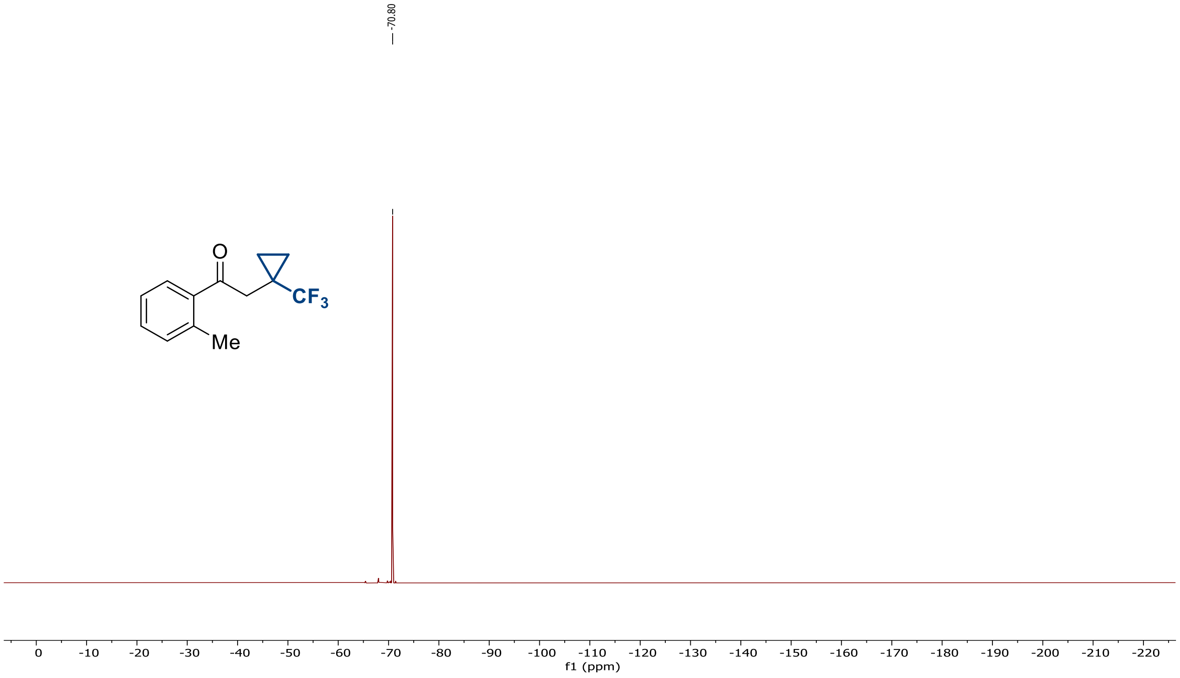

Compound **4s**:

$^{1}H$ NMR (300 MHz, $CDCl_3$)

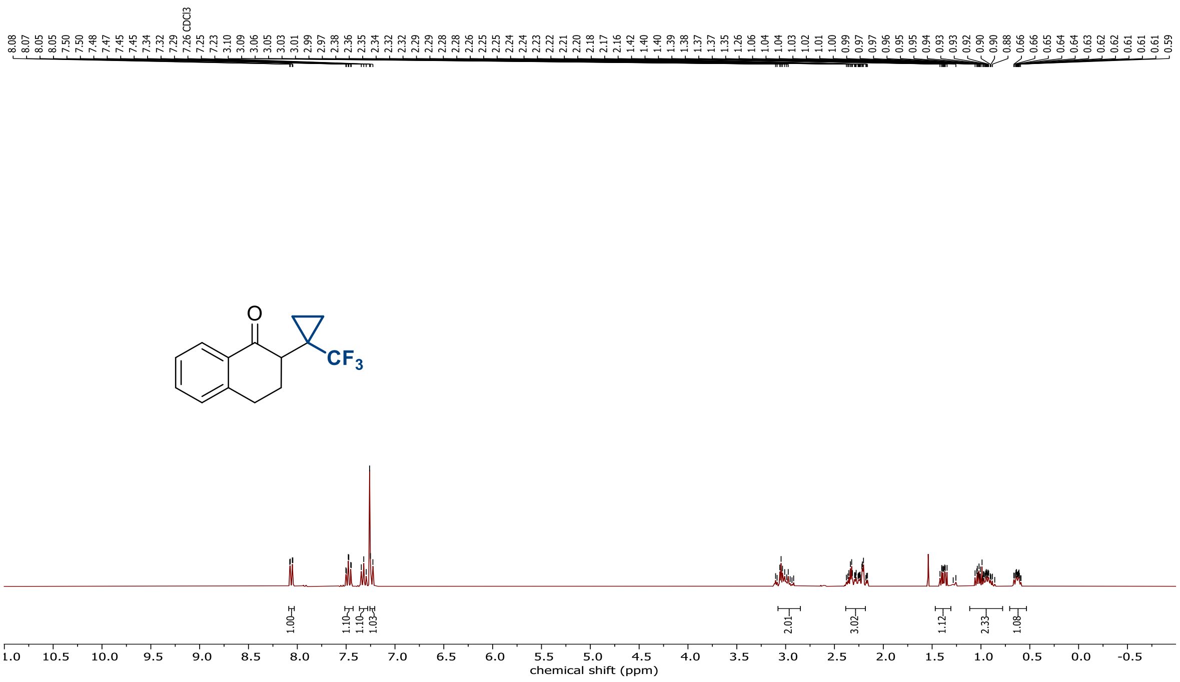


$^{13}C\{1H\}$ NMR (101 MHz, $CDCl_3$)

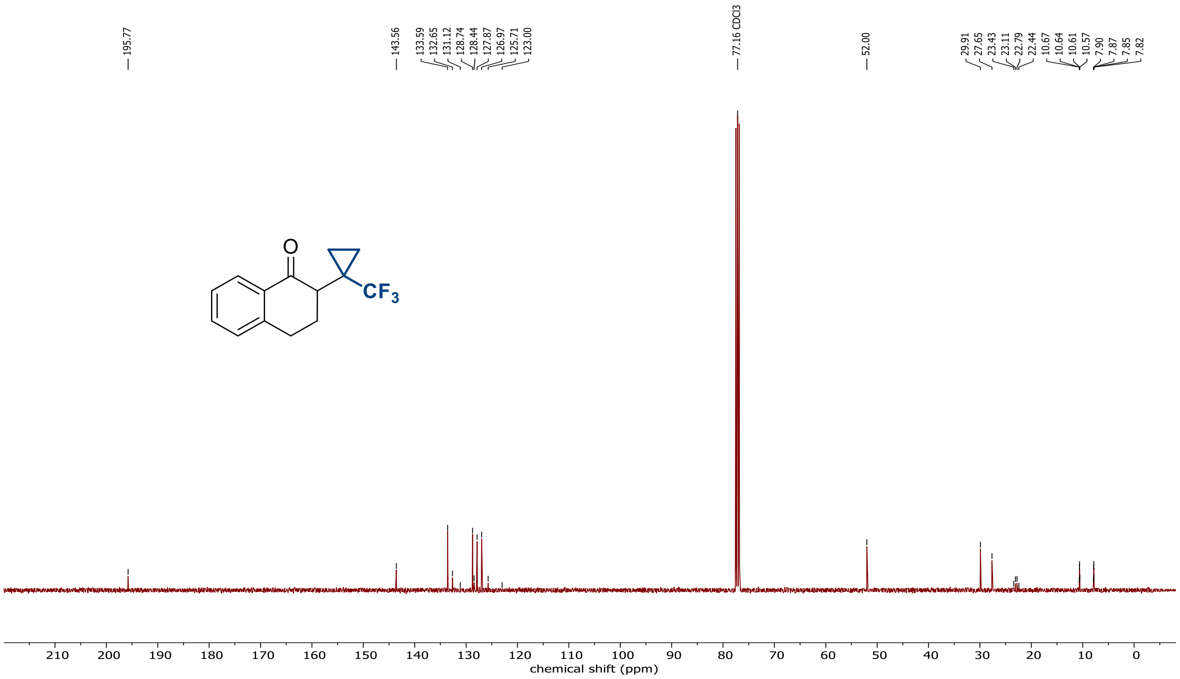

$^{19}F$ NMR (282 MHz, $CDCl_3$)

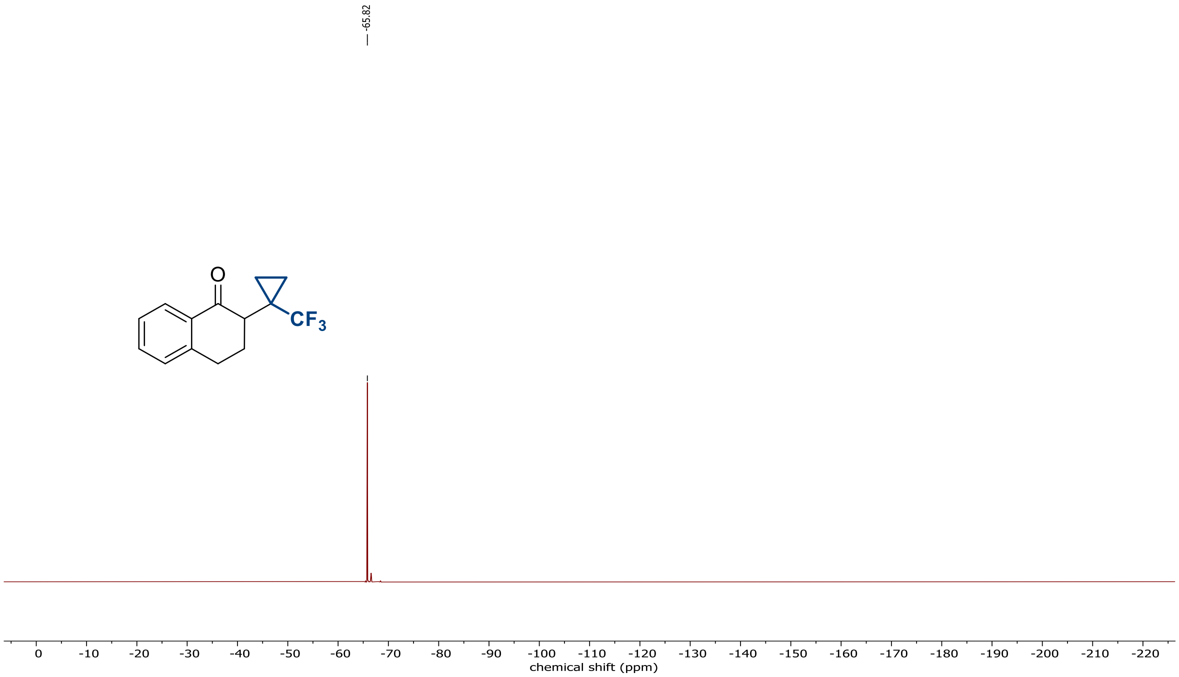


Compound **4t**:

$^{1}H$ NMR (400 MHz, $CDCl_3$)

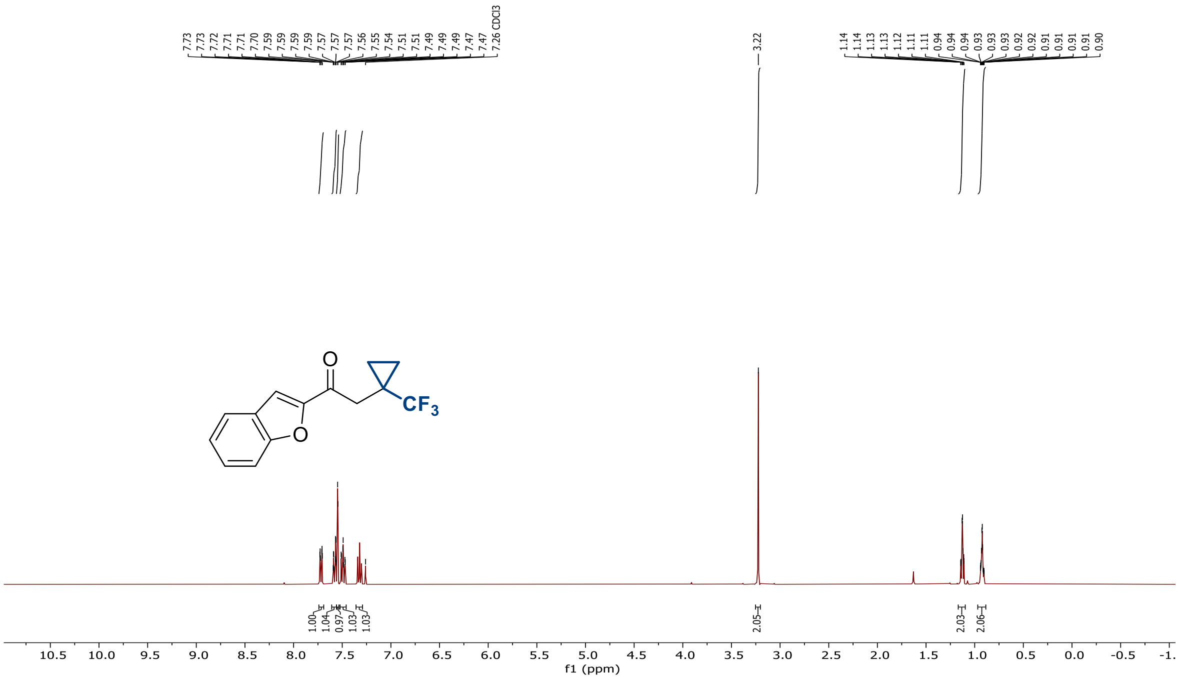

$^{13}C\{1H\}$ NMR (101 MHz, $CDCl_3$)

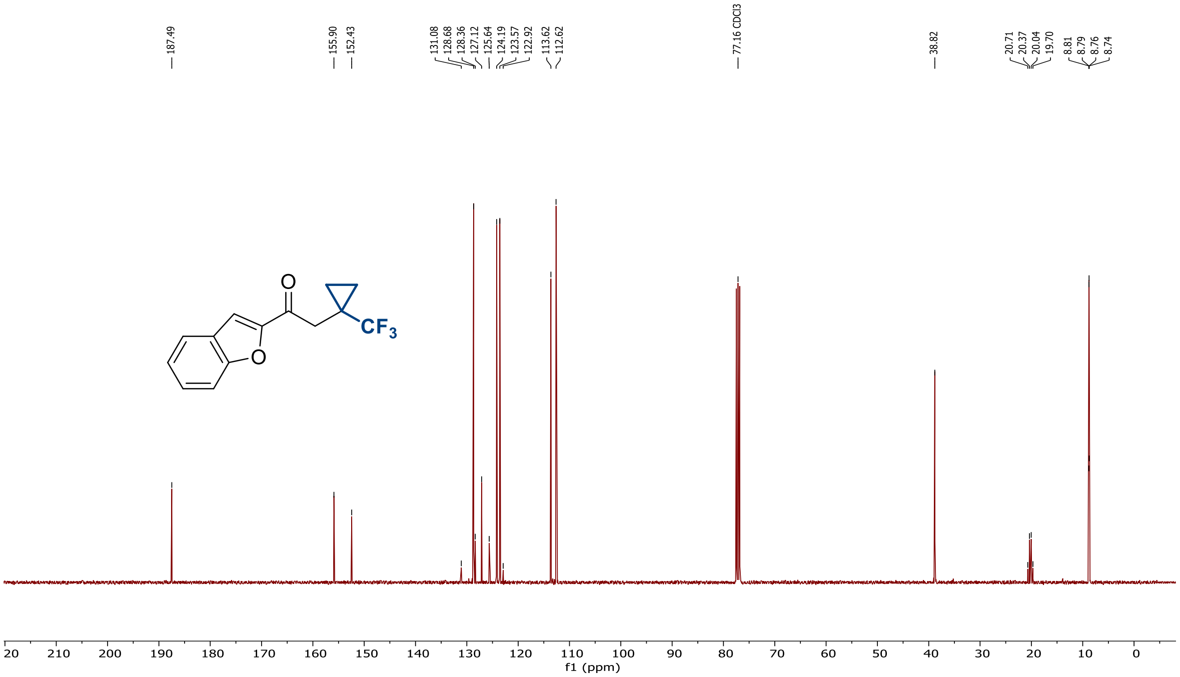


$^{19}F$ NMR (377 MHz, $CDCl_3$)

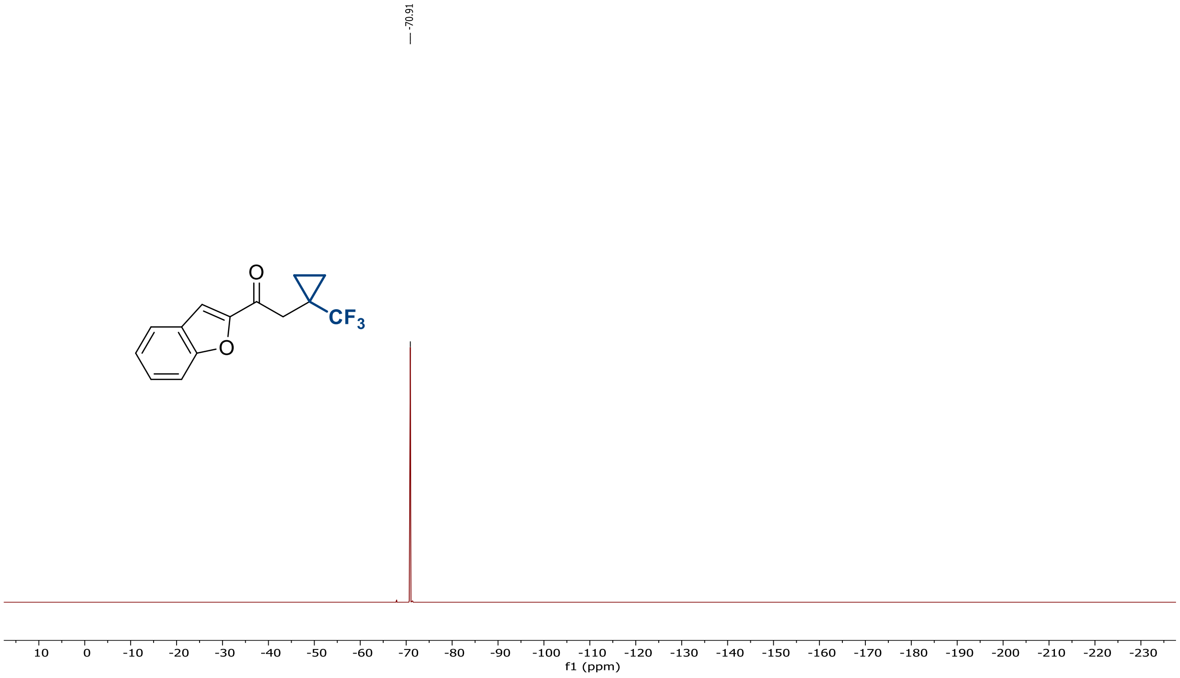

Compound **4u**:

$^{1}$H NMR (400 MHz, $CDCl_3$)

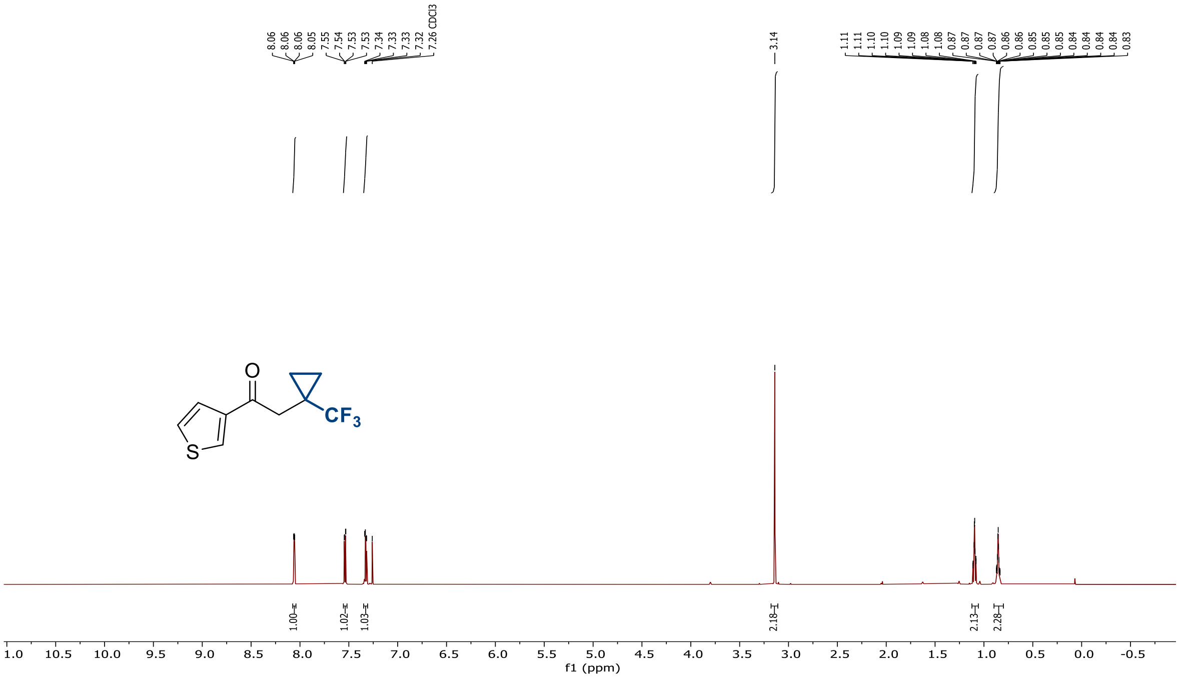


$^{13}$C{1H} NMR (101 MHz, $CDCl_3$)

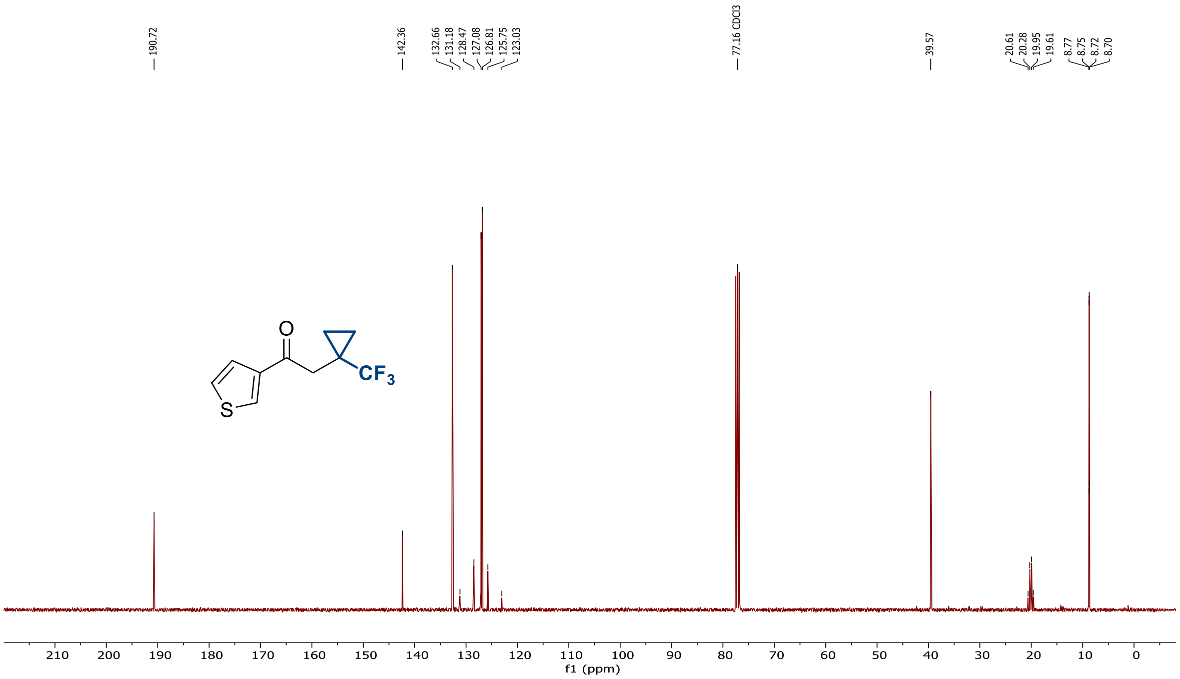

$^{19}$F NMR (377 MHz, $CDCl_3$)

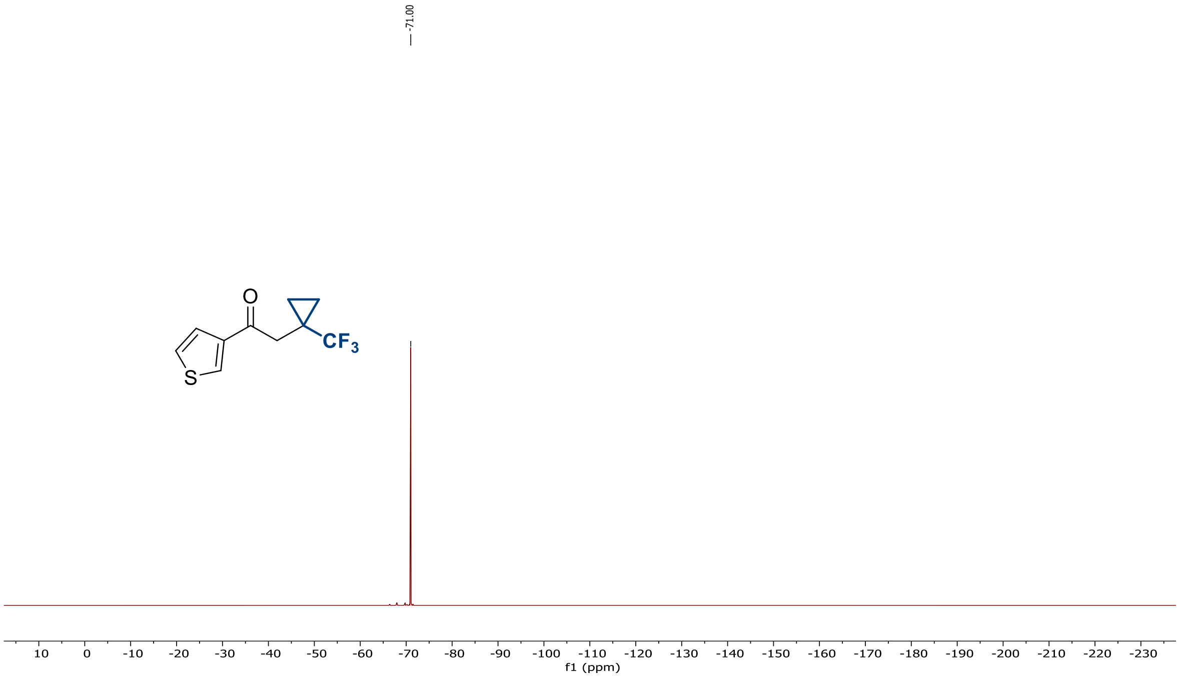

Compound **4v**:

$^{1}$H NMR (400 MHz, $CDCl_3$)

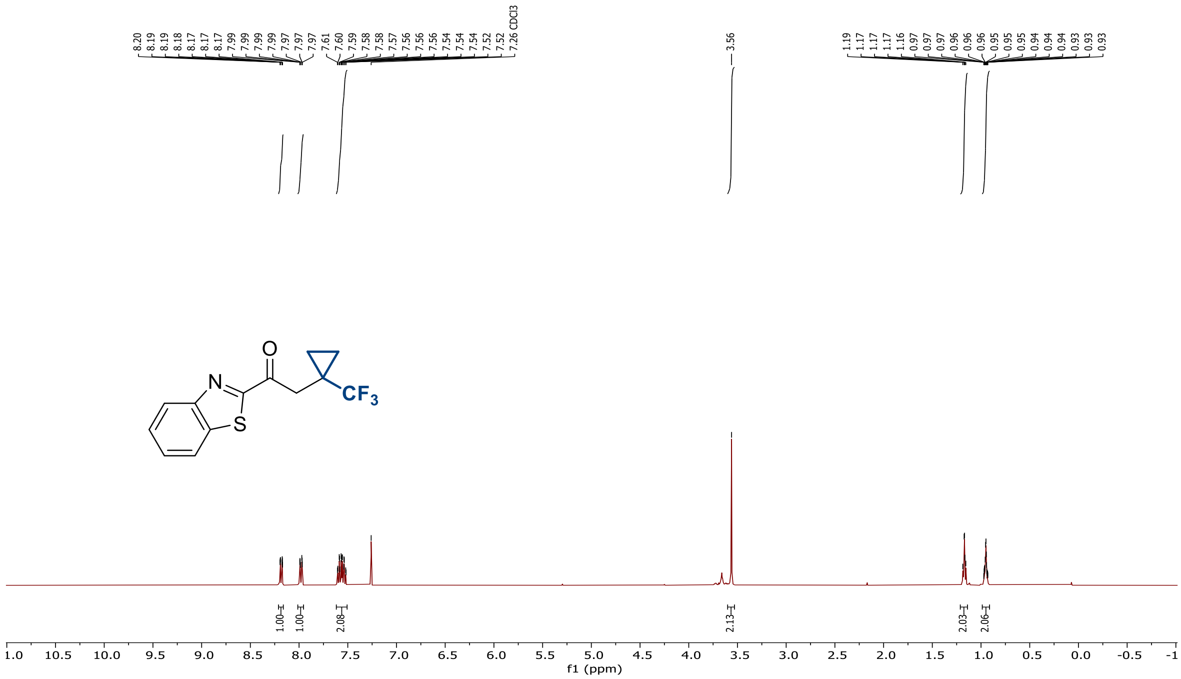

$^{13}C\{1H\}$ NMR (101 MHz, $CDCl_3$)

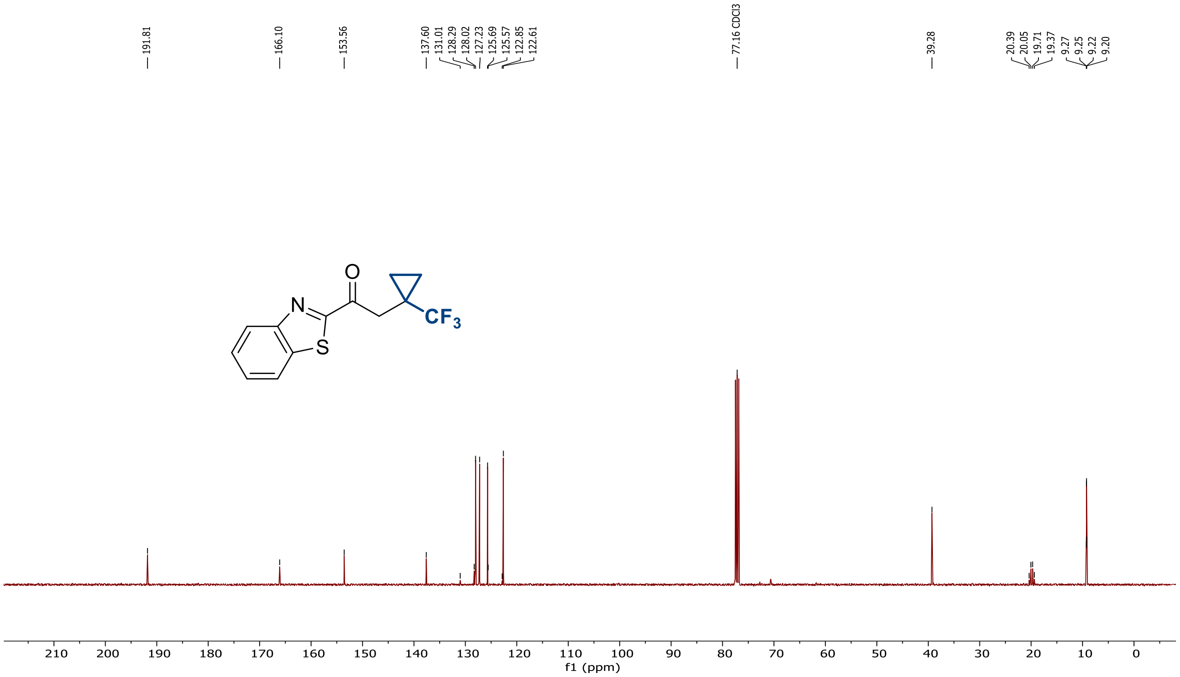


$^{19}F$ NMR (377 MHz, $CDCl_3$)

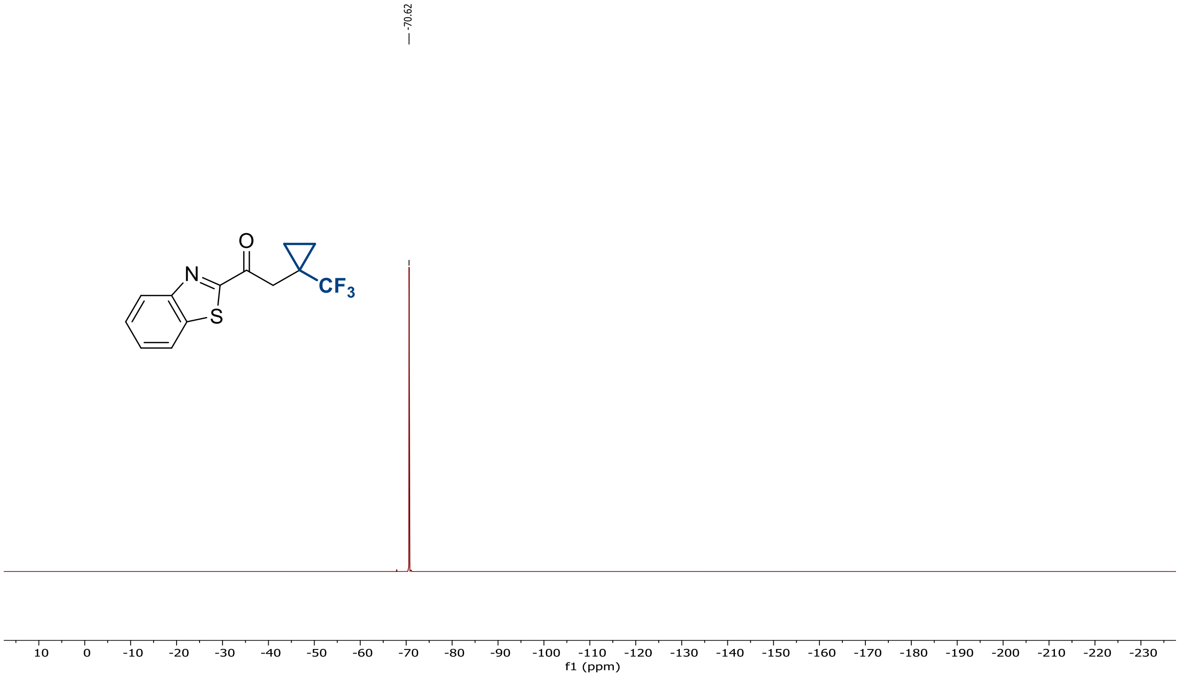

Compound **4w**:

$^{1}$H NMR (300 MHz, $CDCl_3$)

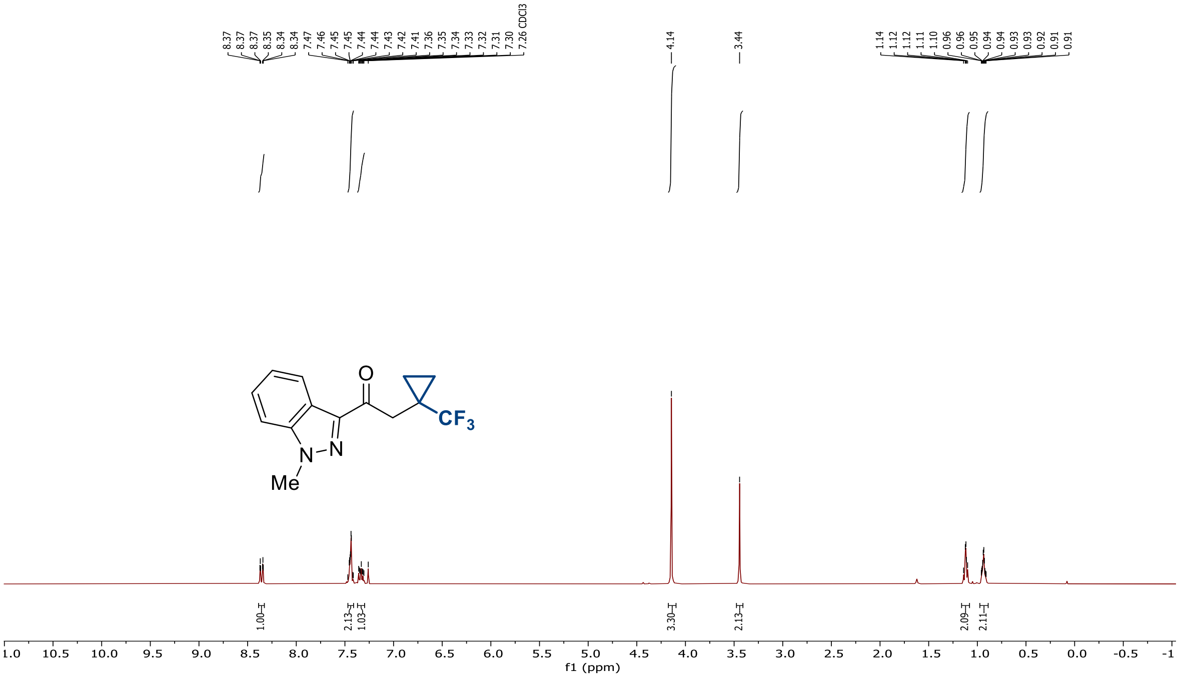


$^{13}$C{1H} NMR (101 MHz, $CDCl_3$)

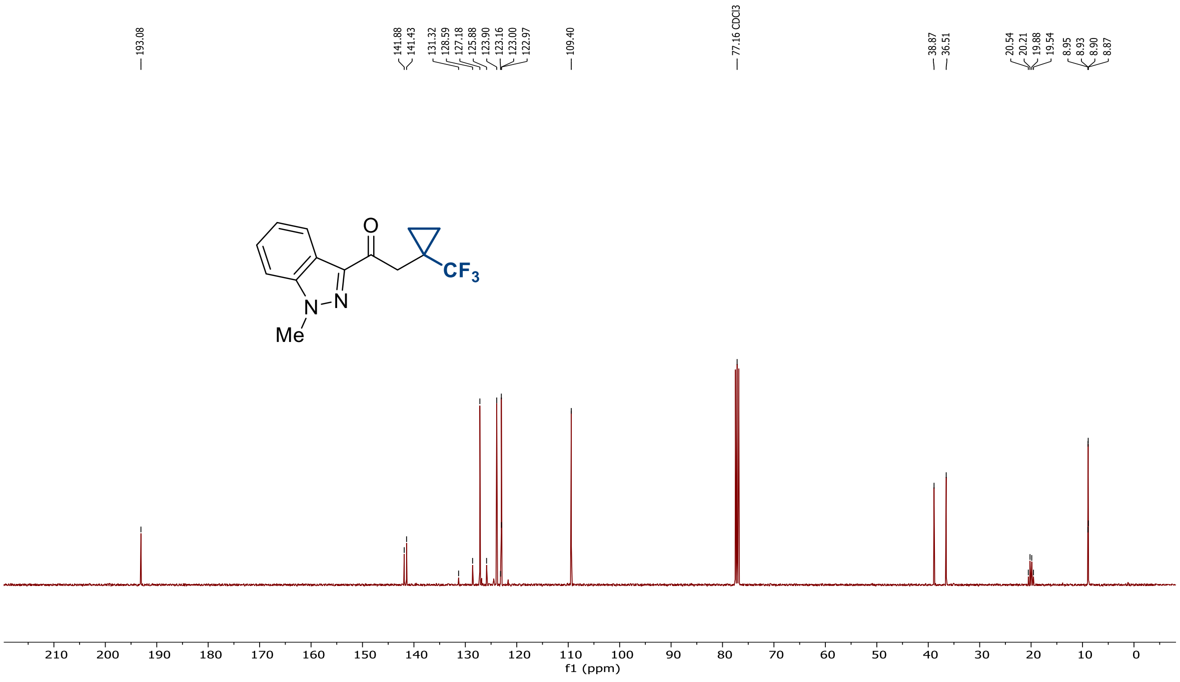

$^{19}F$ NMR (282 MHz, $CDCl_3$)

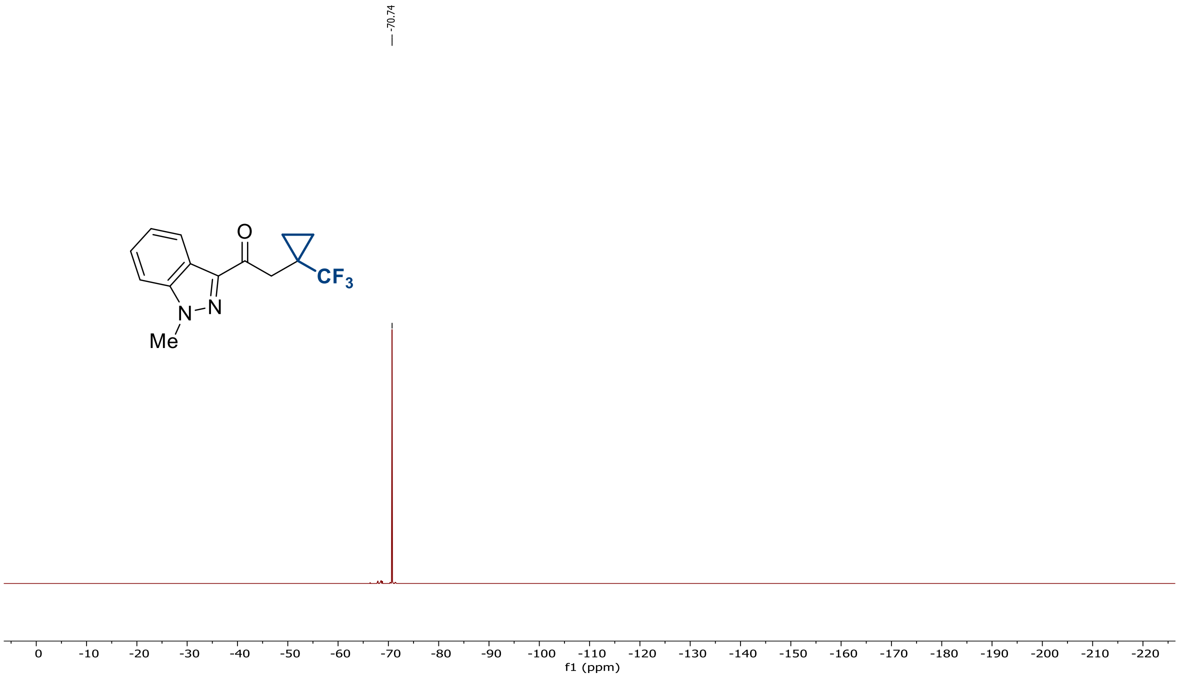


Compound **4x**:

$^{1}H$ NMR (300 MHz, $CDCl_3$)

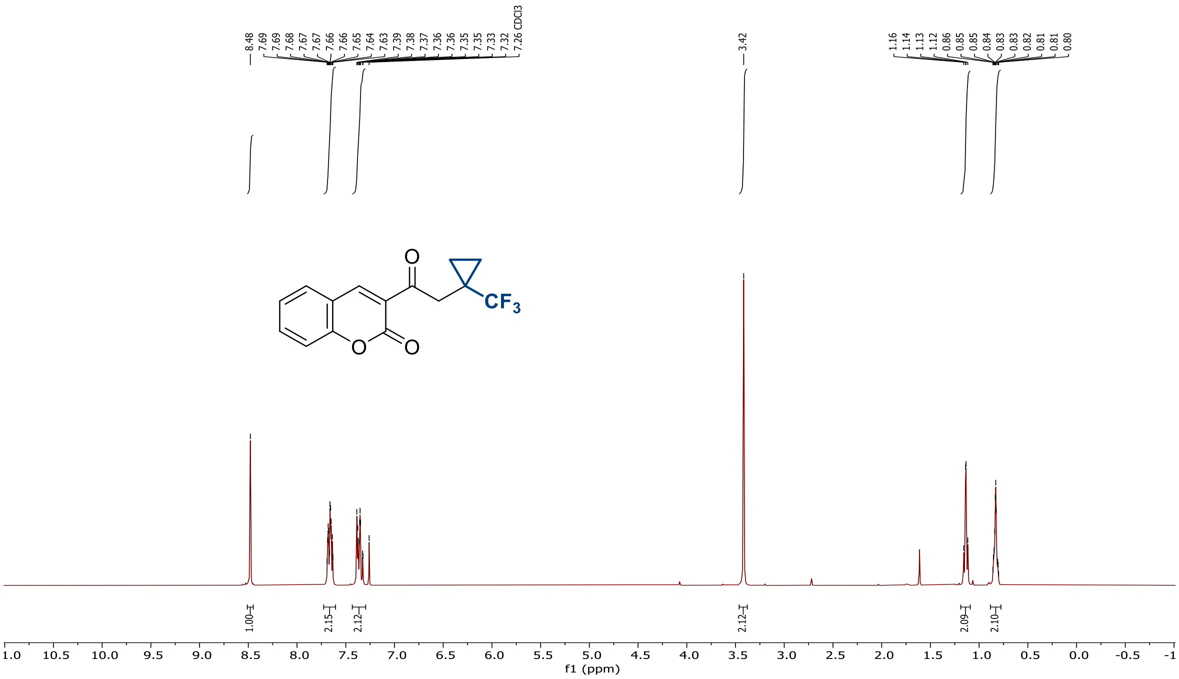

$^{13}C\{1H\}$ NMR (101 MHz, $CDCl_3$)

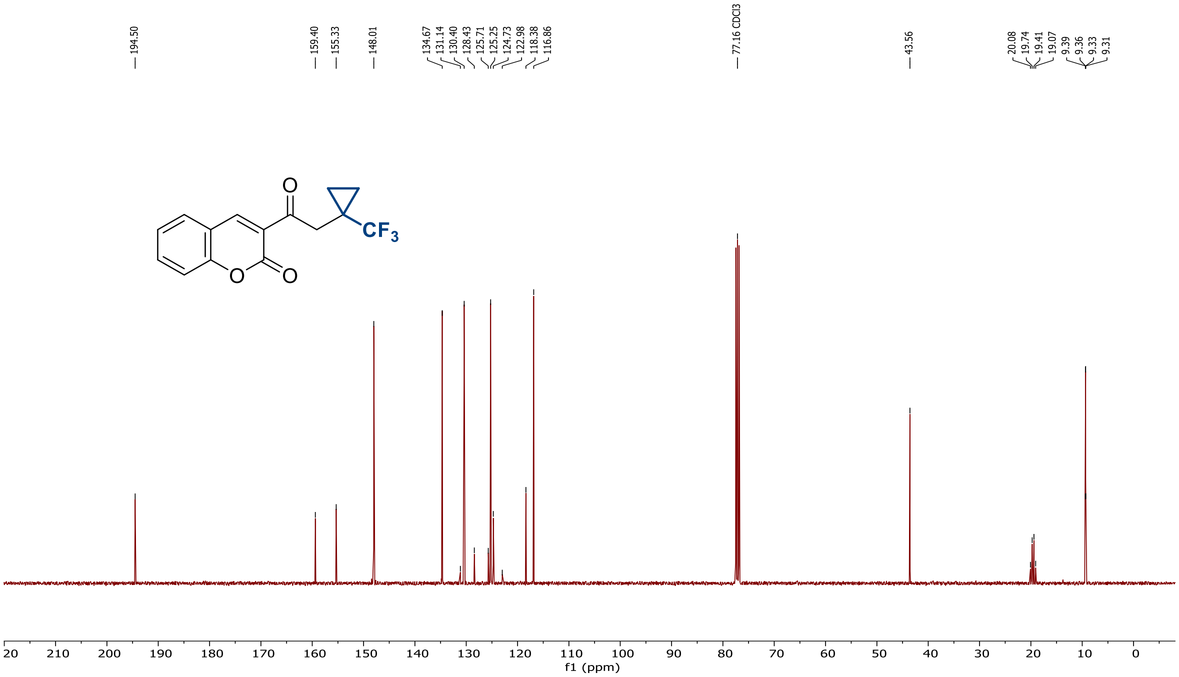

$^{19}F$ NMR (377 MHz, $CDCl_3$)

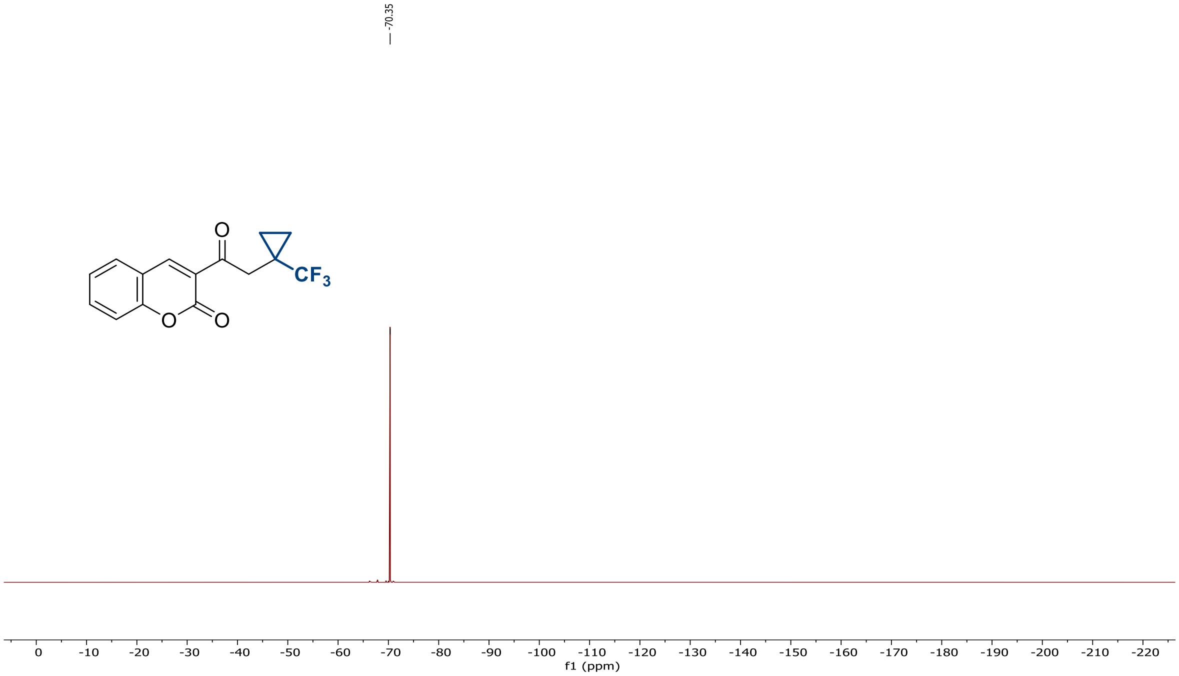

Compound **4y**:

$^{1}$H NMR (400 MHz, $CDCl_3$)

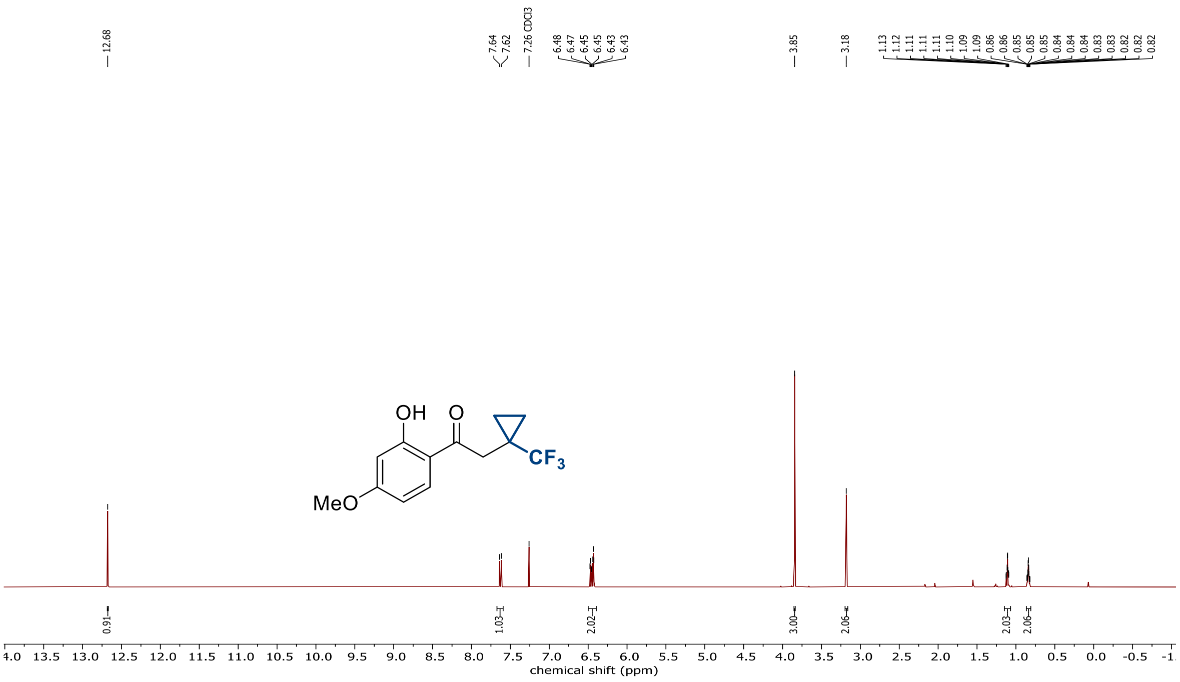


$^{13}$C{1H} NMR (101 MHz, $CDCl_3$)

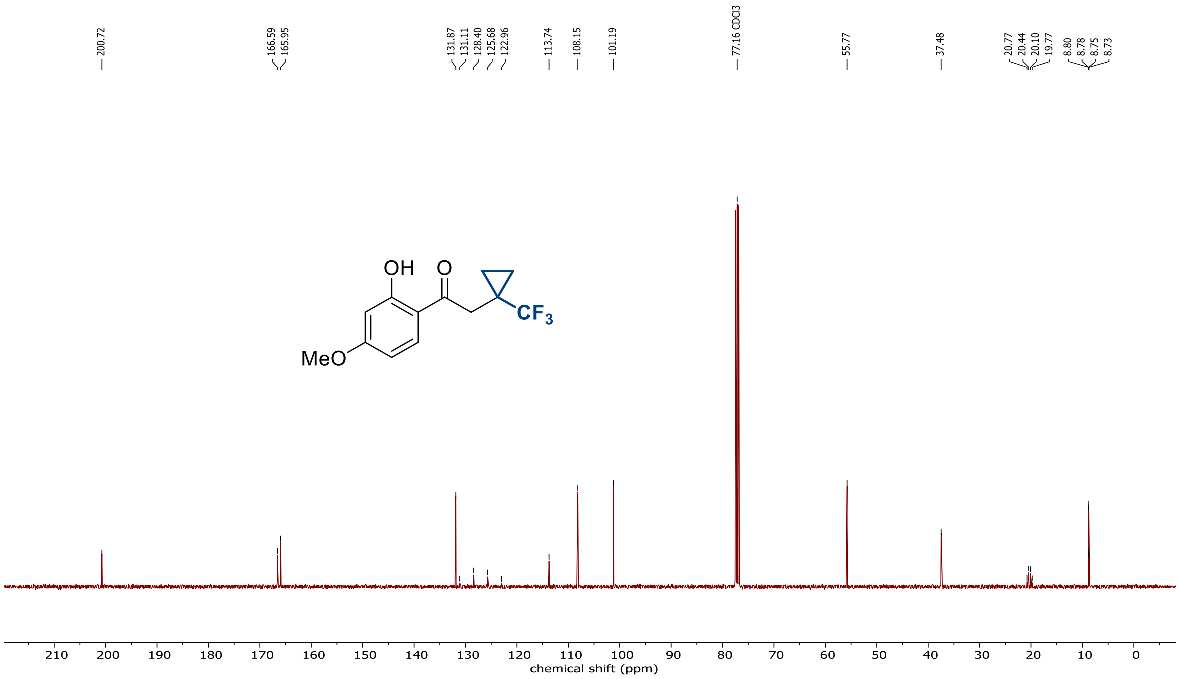

$^{19}$F NMR (377 MHz, $CDCl_3$)

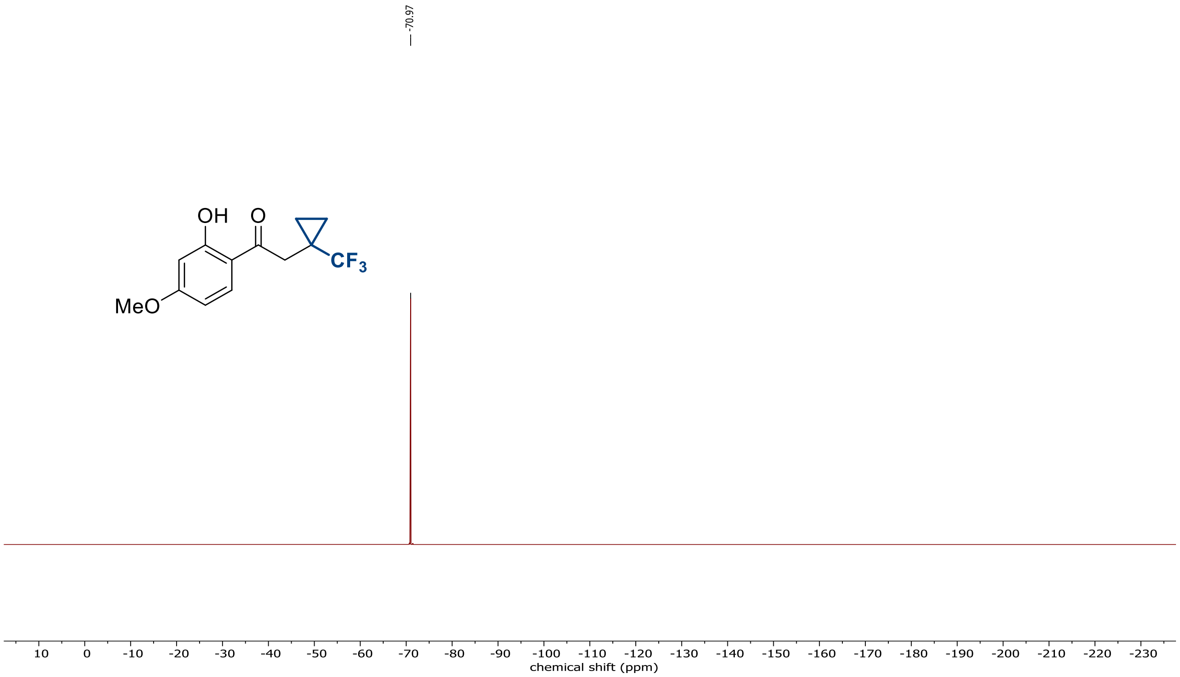


Compound **4z**:

$^{1}$H NMR (300 MHz, $CDCl_3$)

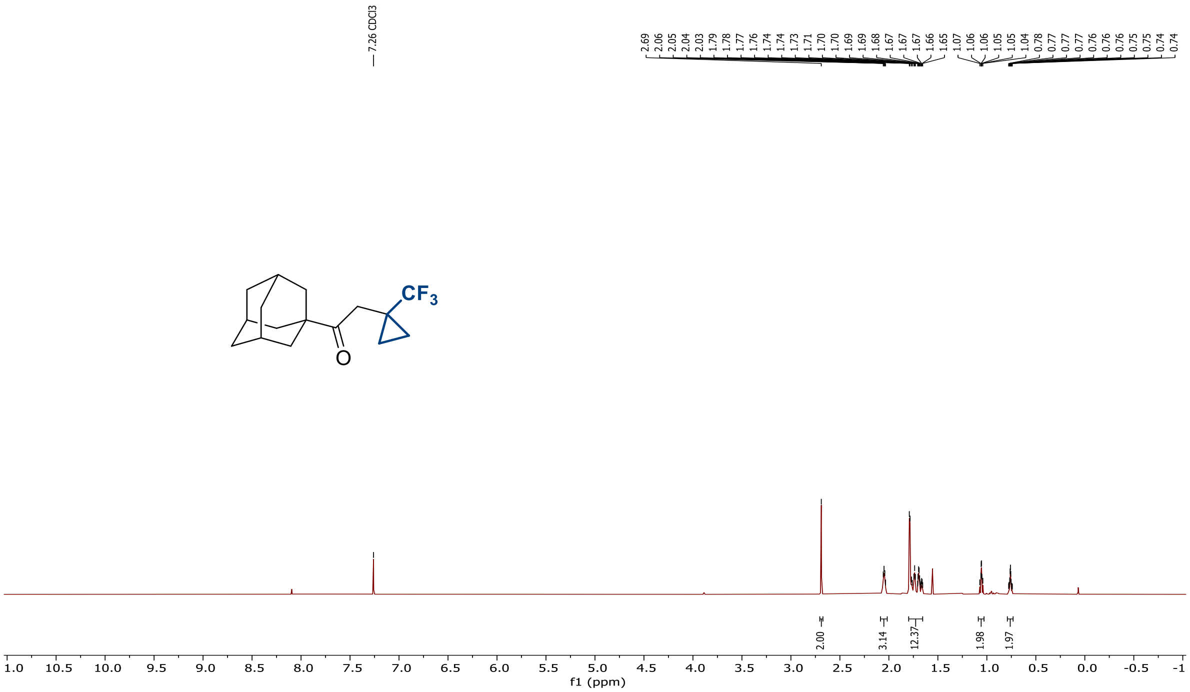

## $^{13}C\{1H\}$ NMR (101 MHz, $CDCl_3$)

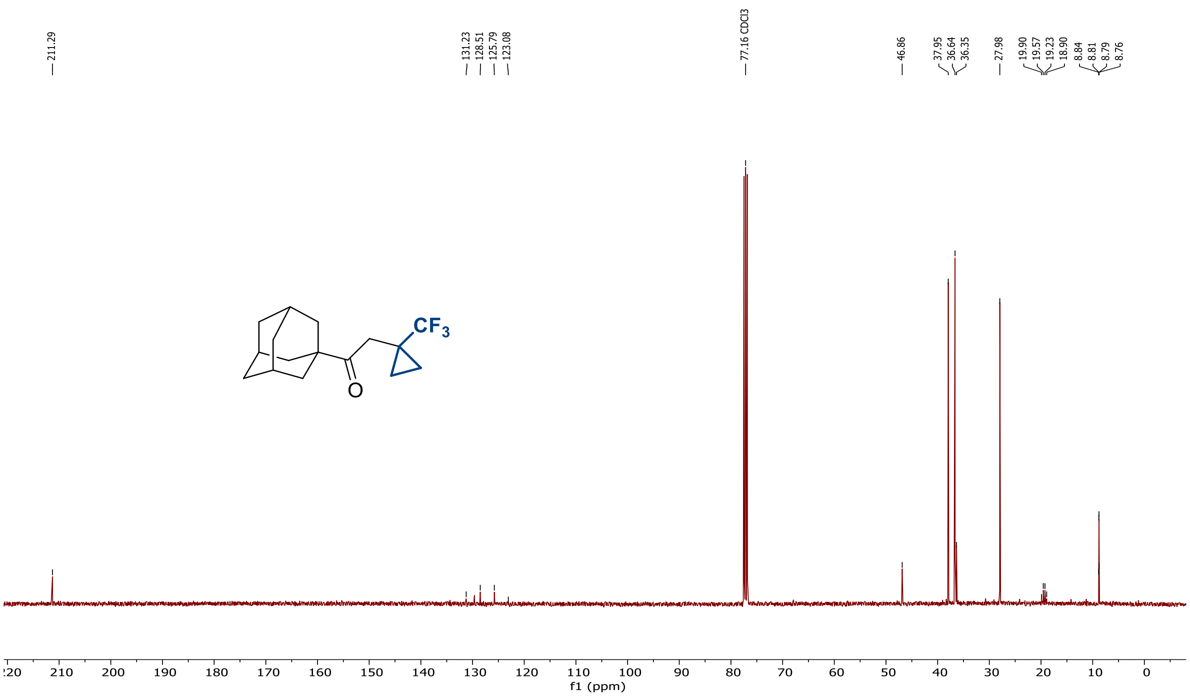


## $^{19}F$ NMR (282 MHz, $CDCl_3$)

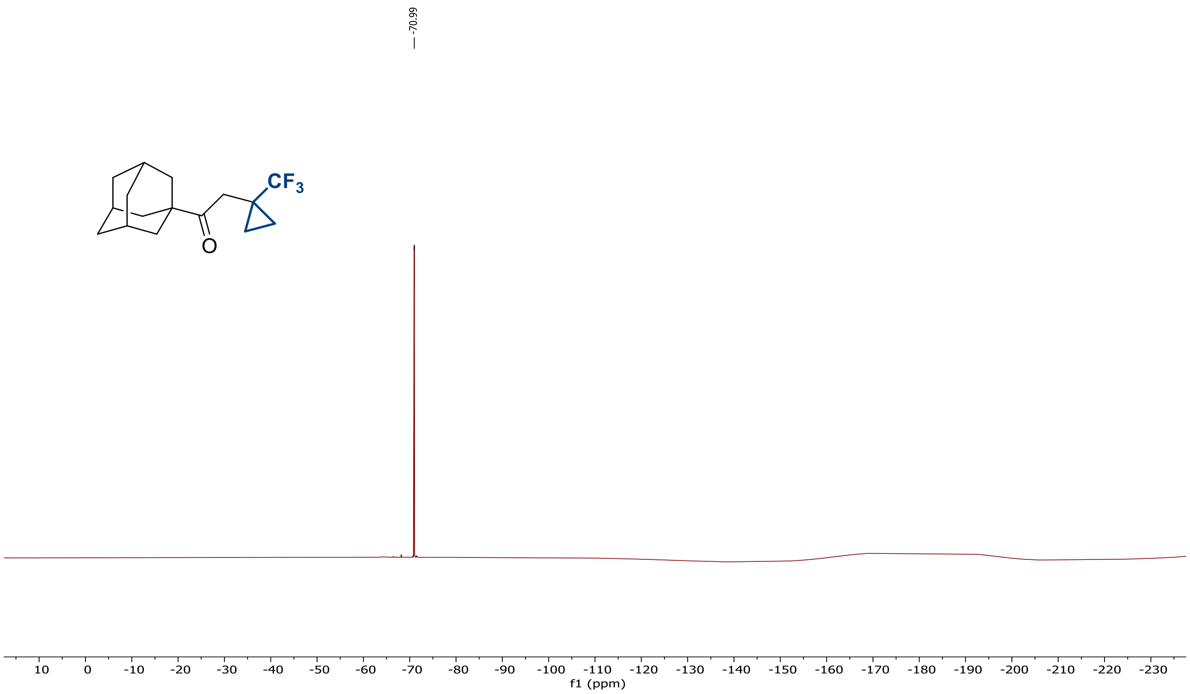

Compound **6a**:

$^1$H NMR (300 MHz, $CDCl_3$)

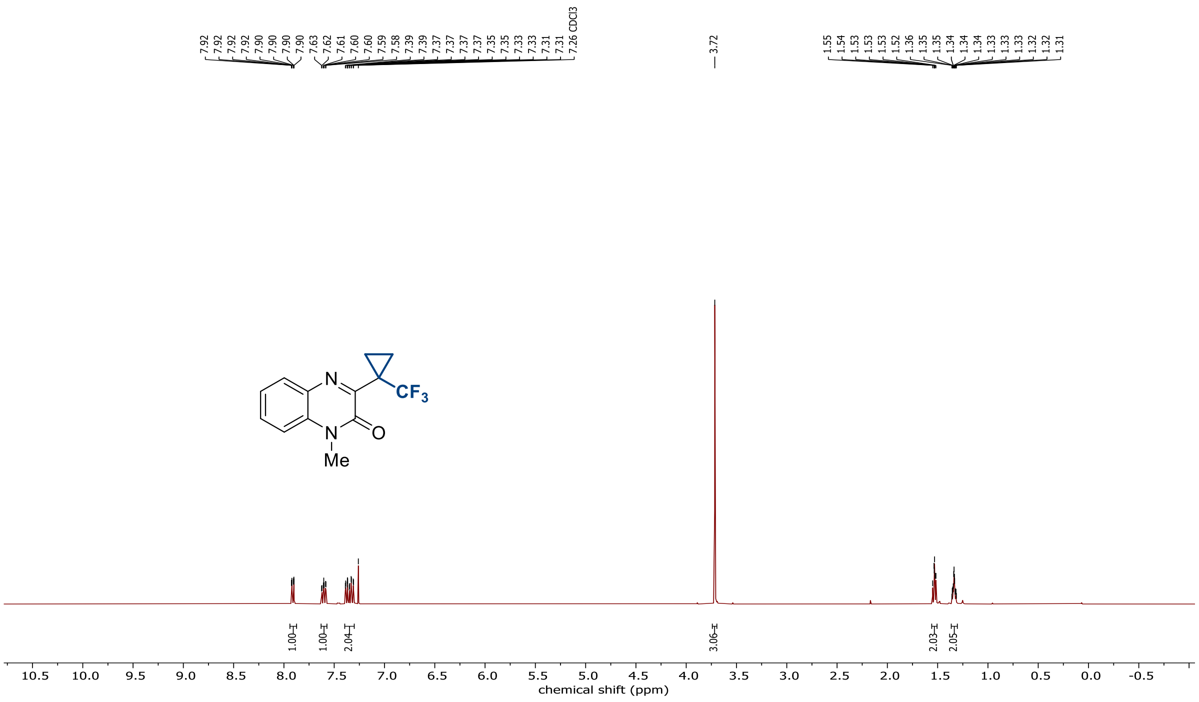


$^{13}$C{1H} NMR (101 MHz, $CDCl_3$)

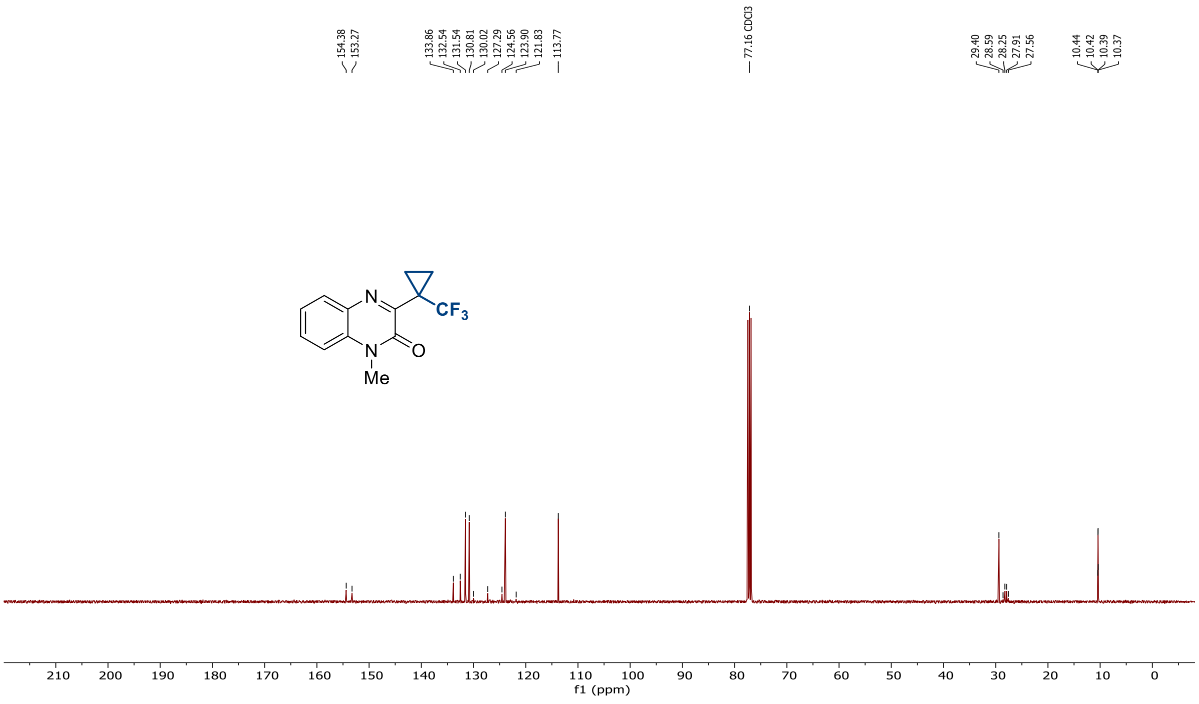

$^{19}$F NMR (282 MHz, $CDCl_3$)

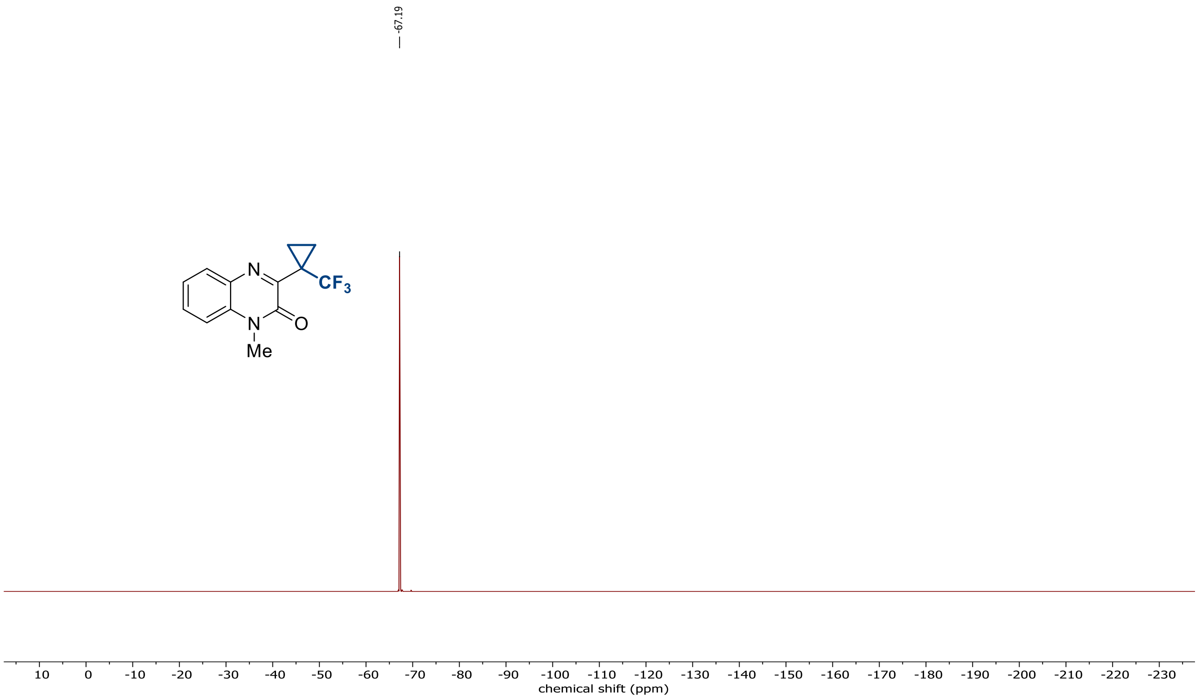


Compound **6b**:

$^{1}$H NMR (300 MHz, $CDCl_3$)

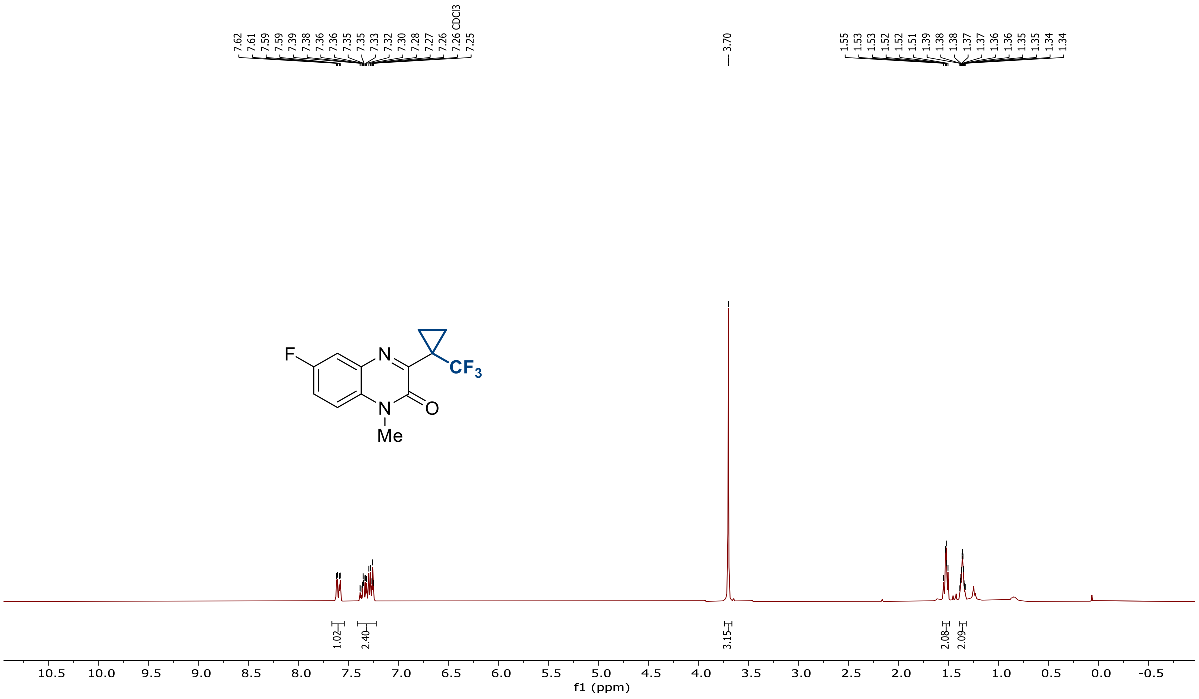

$^{13}C\{1H\}$ NMR (101 MHz, $CDCl_3$)

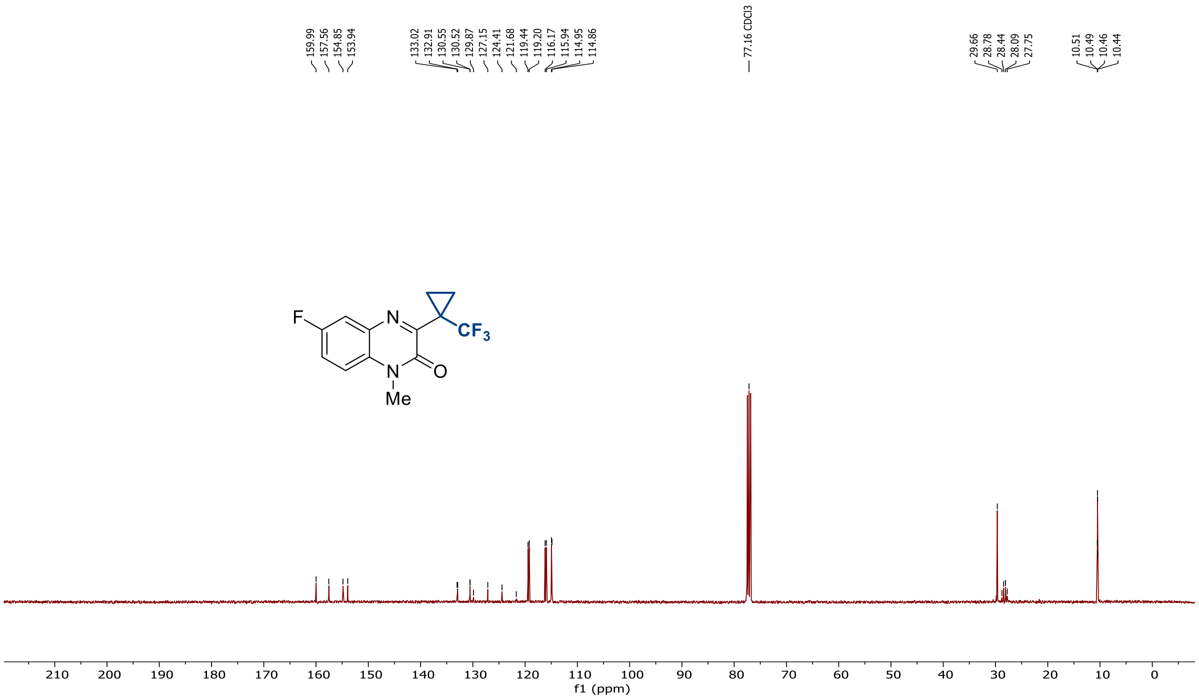

$^{19}F$ NMR (282 MHz, $CDCl_3$)

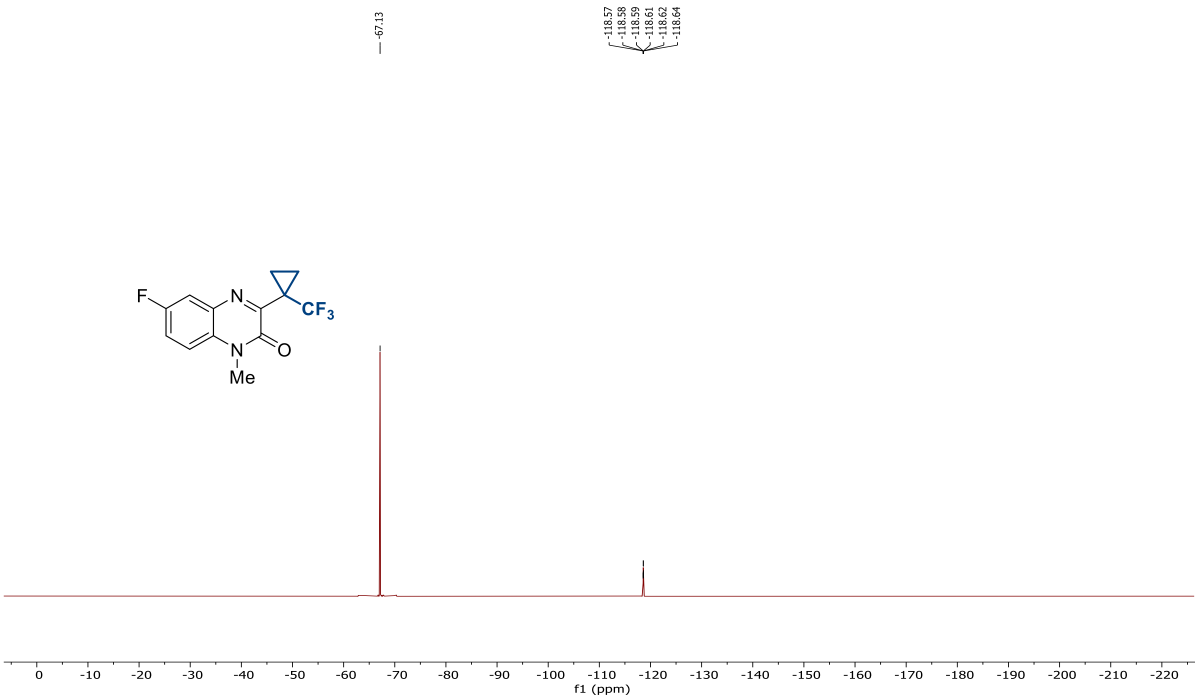

Compound **6c**:

$^{1}H$ NMR (300 MHz, $CDCl_3$)

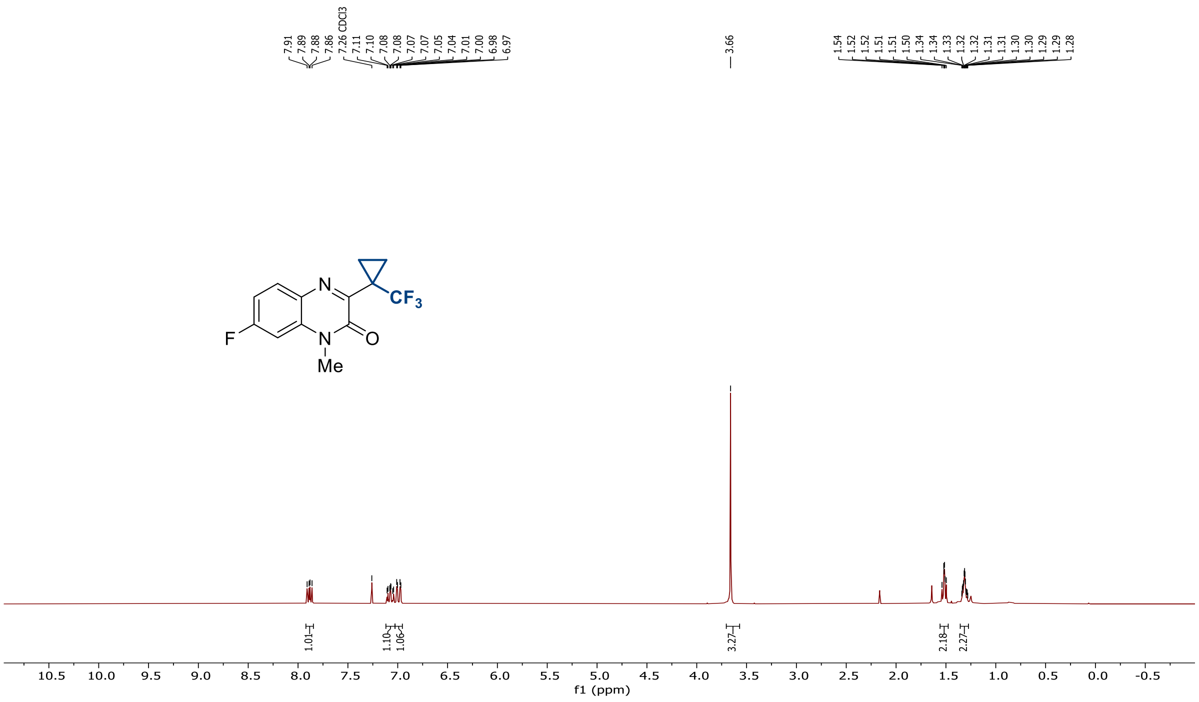


$^{13}C\{1H\}$ NMR (101 MHz, $CDCl_3$)

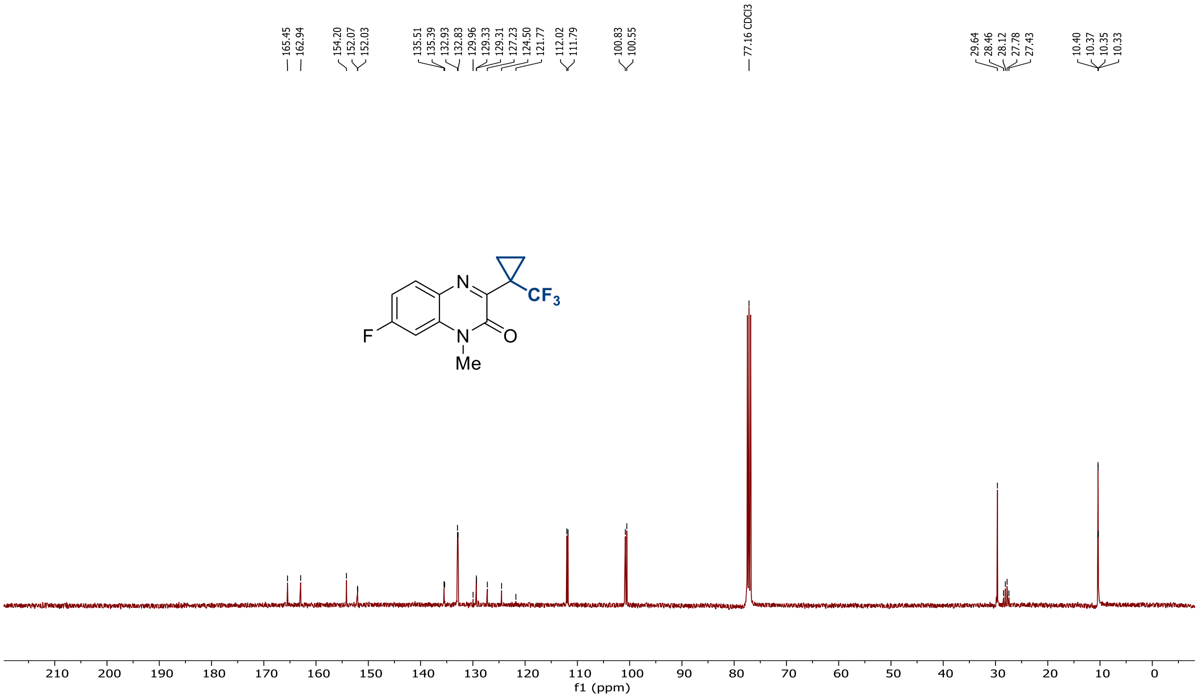

$^{19}F$ NMR (282 MHz, $CDCl_3$)

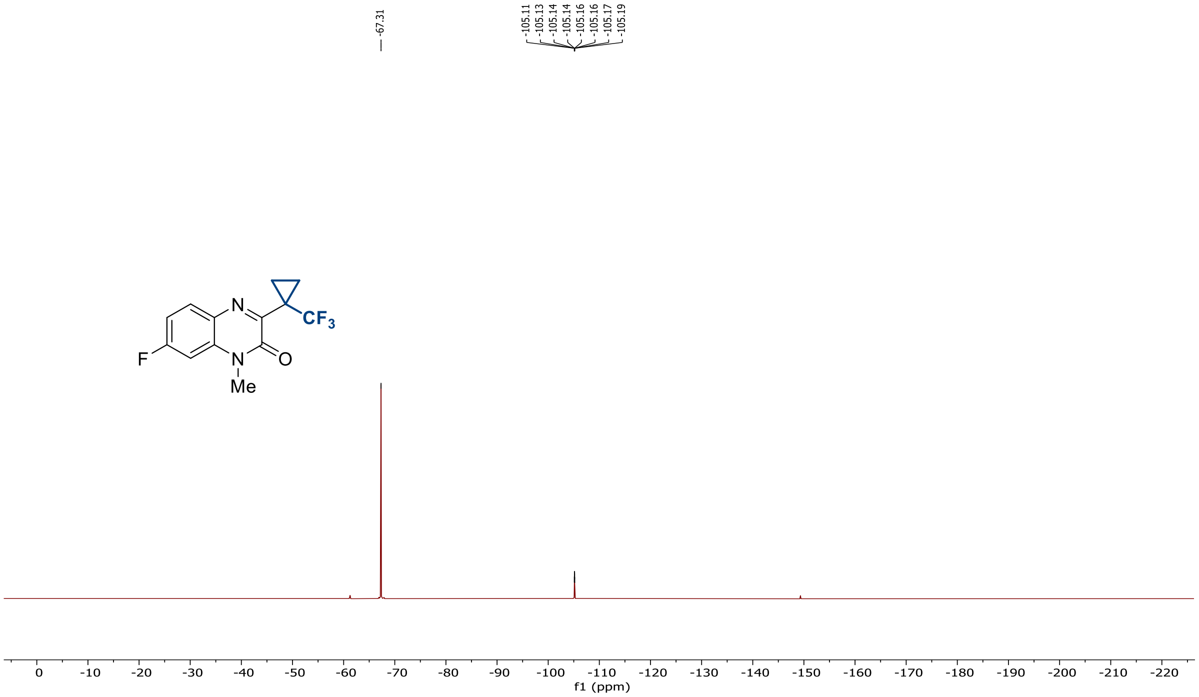


Compound **6d**:

$^{1}H$ NMR (300 MHz, $CDCl_3$)

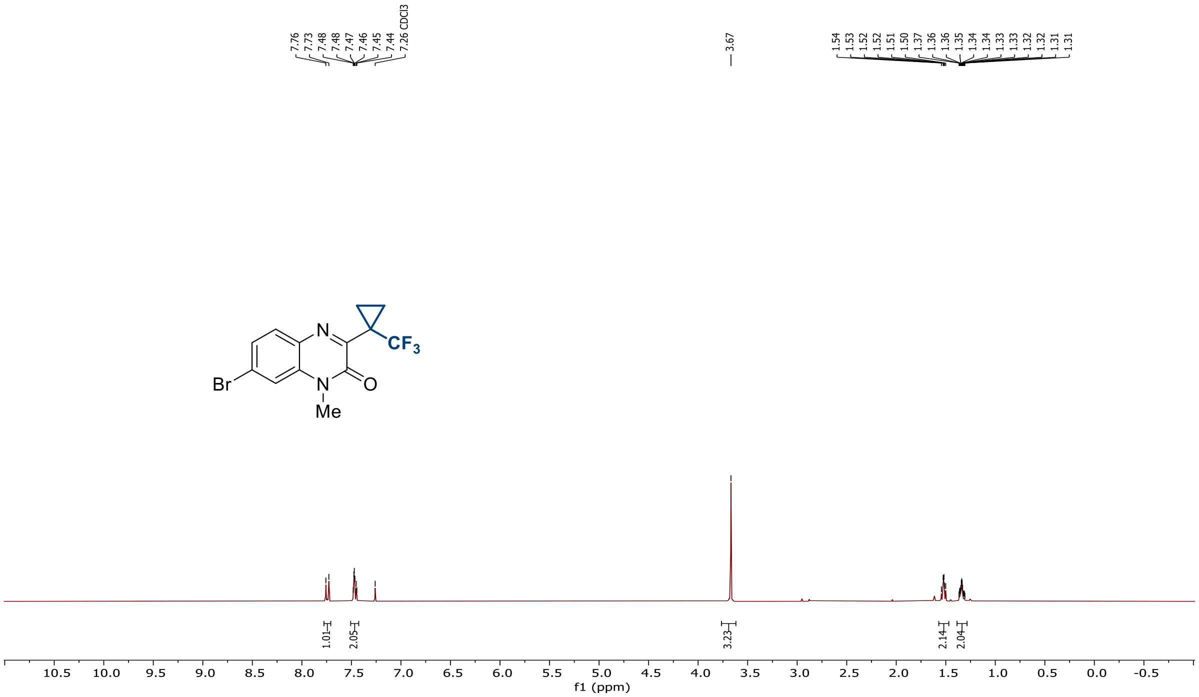

$^{13}C\{^1H\}$ NMR (101 MHz, $CDCl_3$)

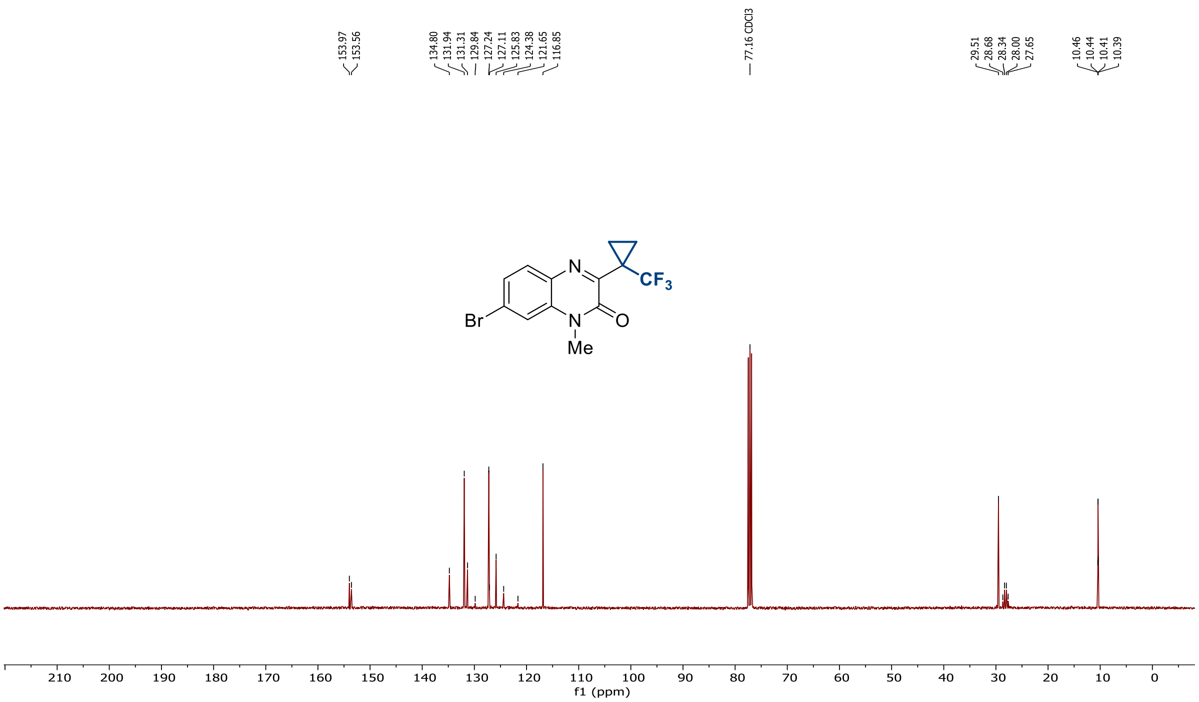


$^{19}F$ NMR (282 MHz, $CDCl_3$)

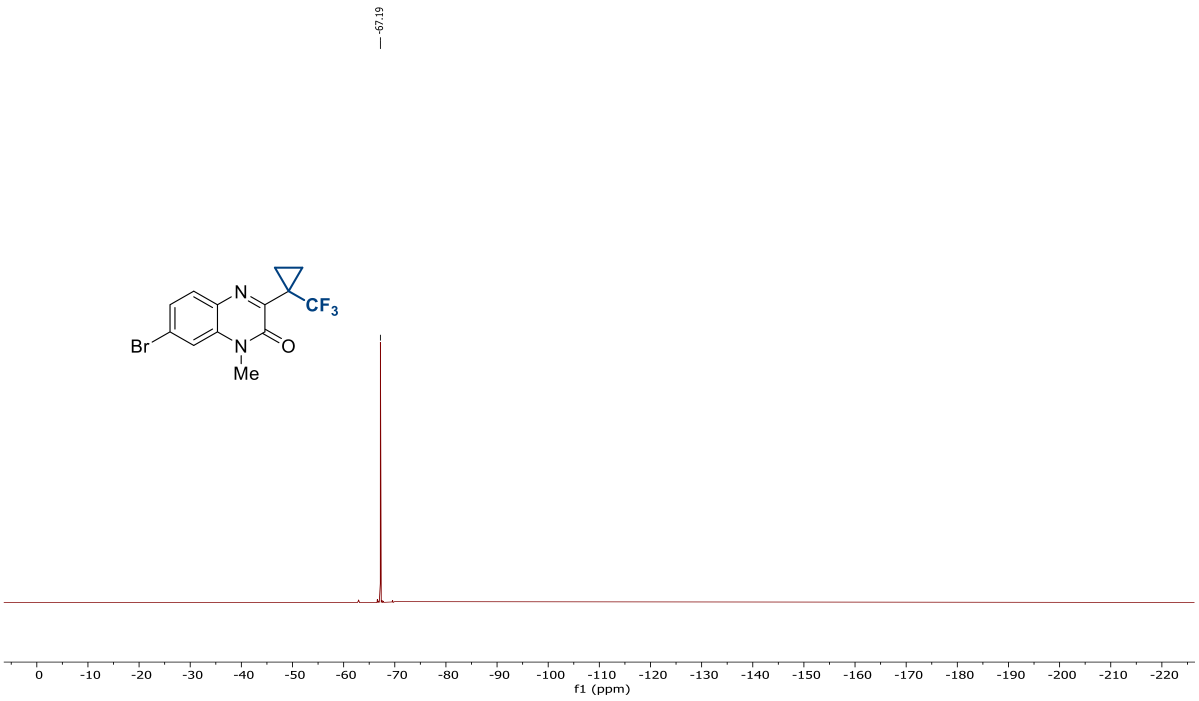

Compound **6e**:

$^1H$ NMR (300 MHz, $CDCl_3$)

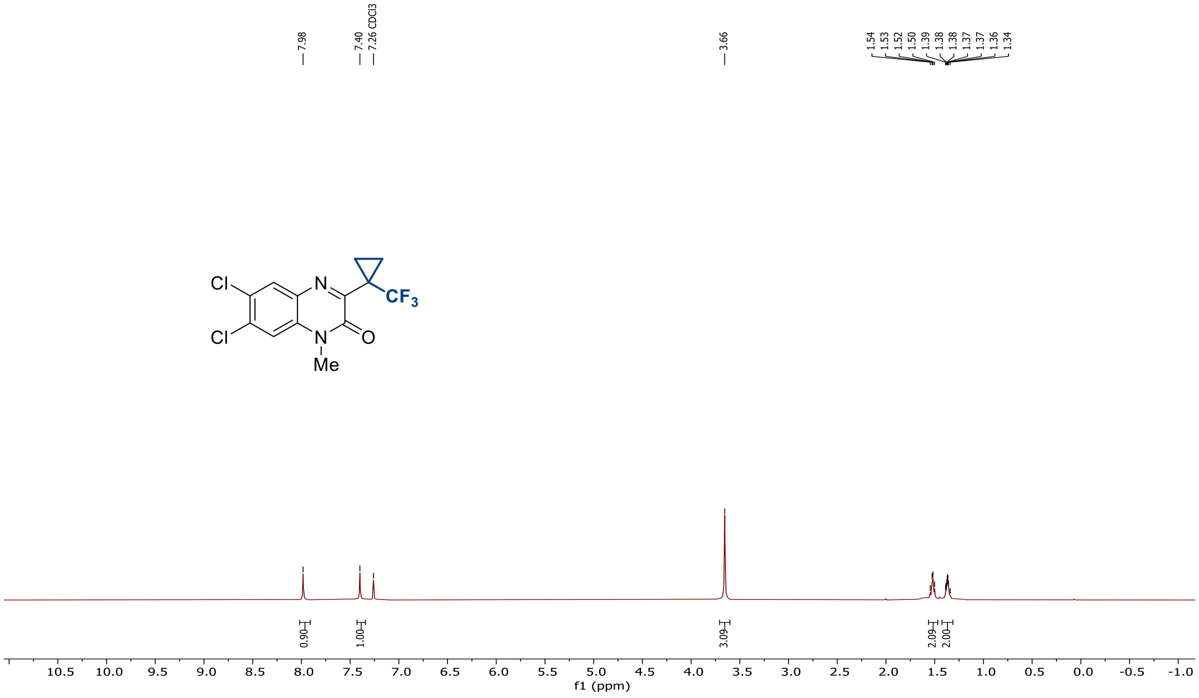


$^{13}C\{1H\}$ NMR (101 MHz, $CDCl_3$)

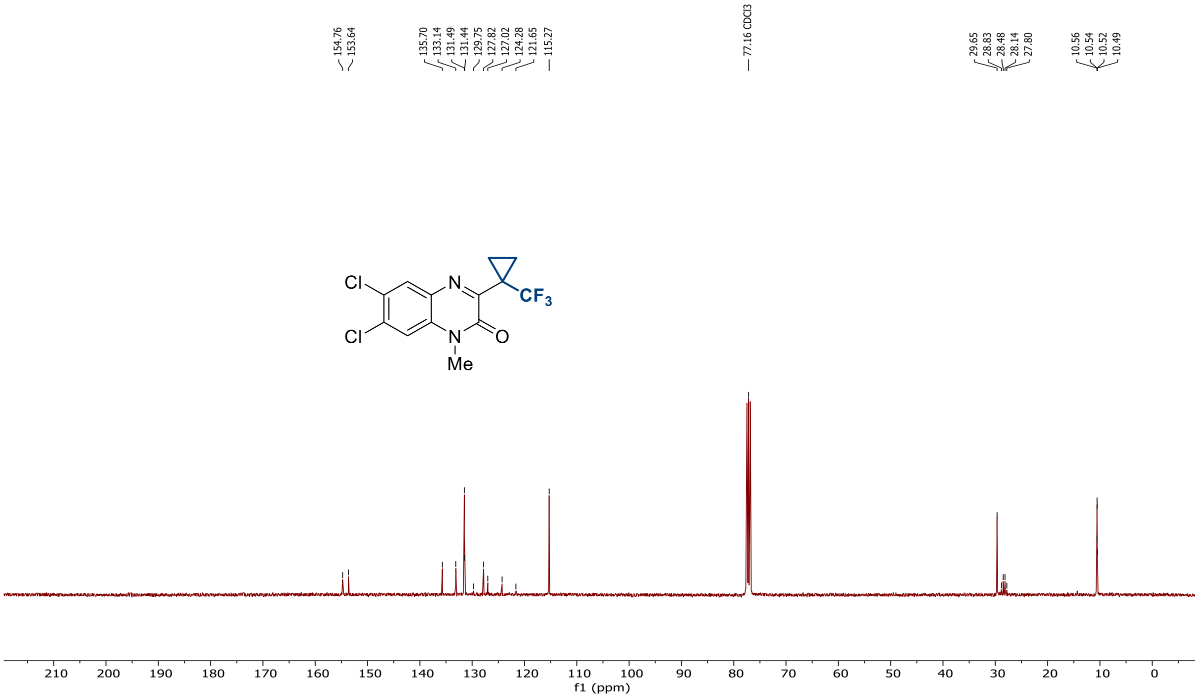

$^{19}F$ NMR (282 MHz, $CDCl_3$)

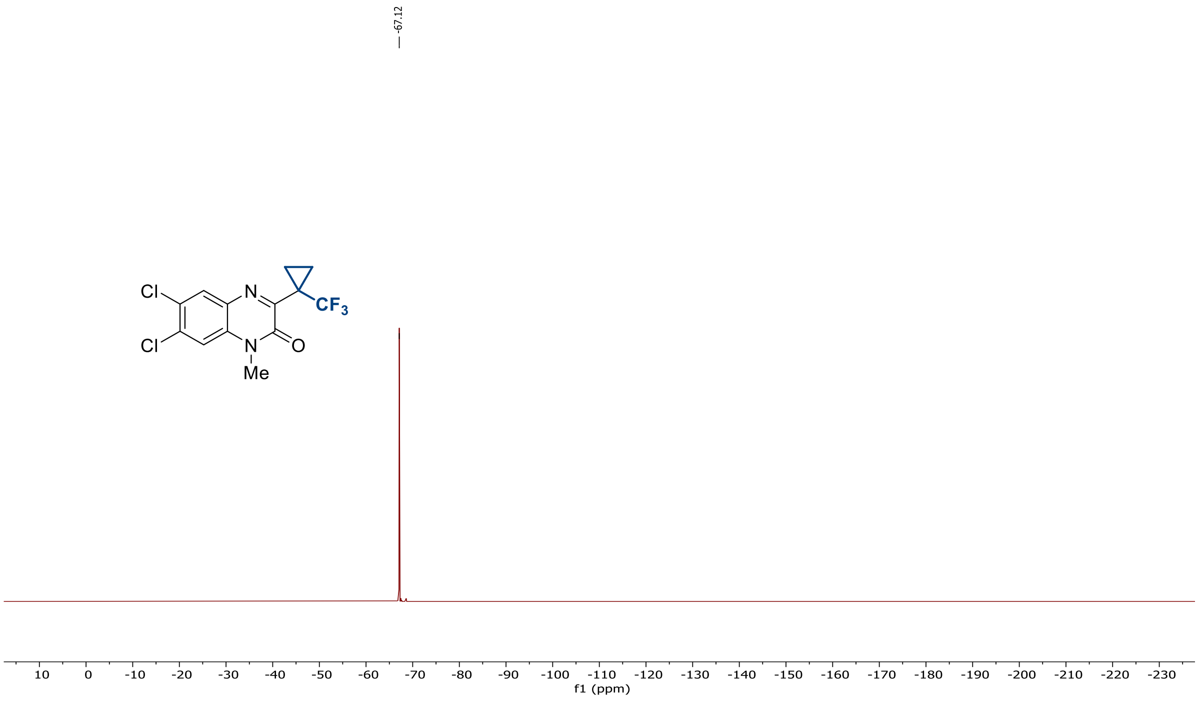


Compound **6f**:

$^{1}H$ NMR (300 MHz, $CDCl_3$)

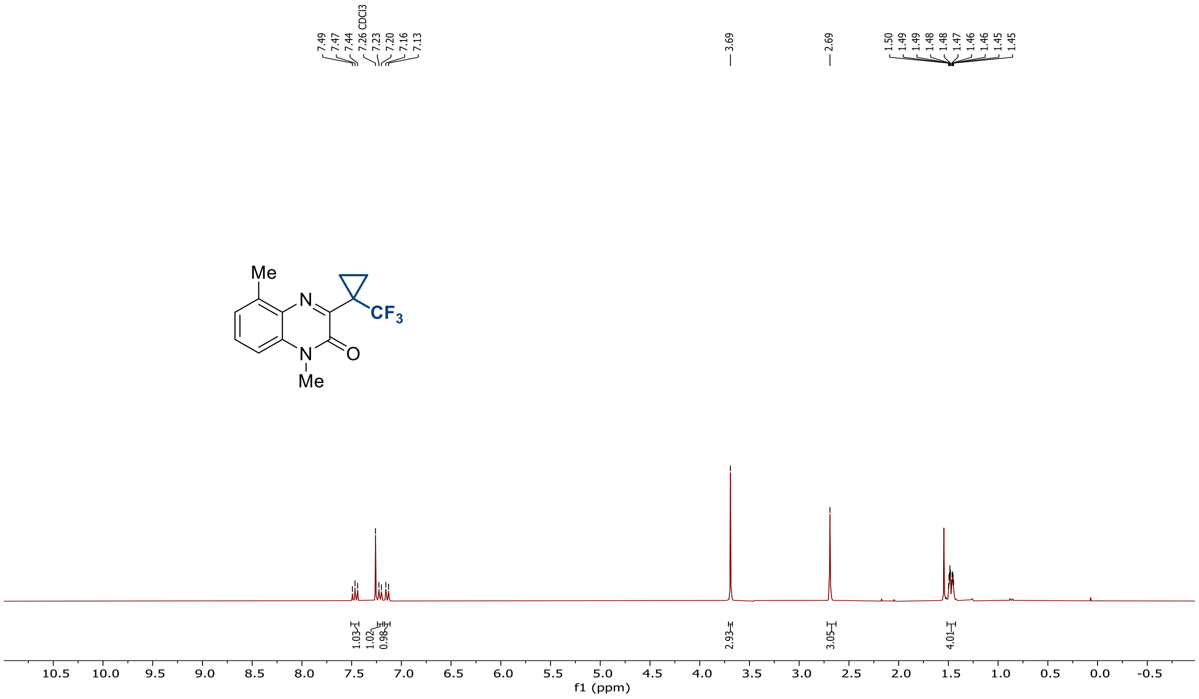

$^{13}C\{1H\}$ NMR (101 MHz, $CDCl_3$)

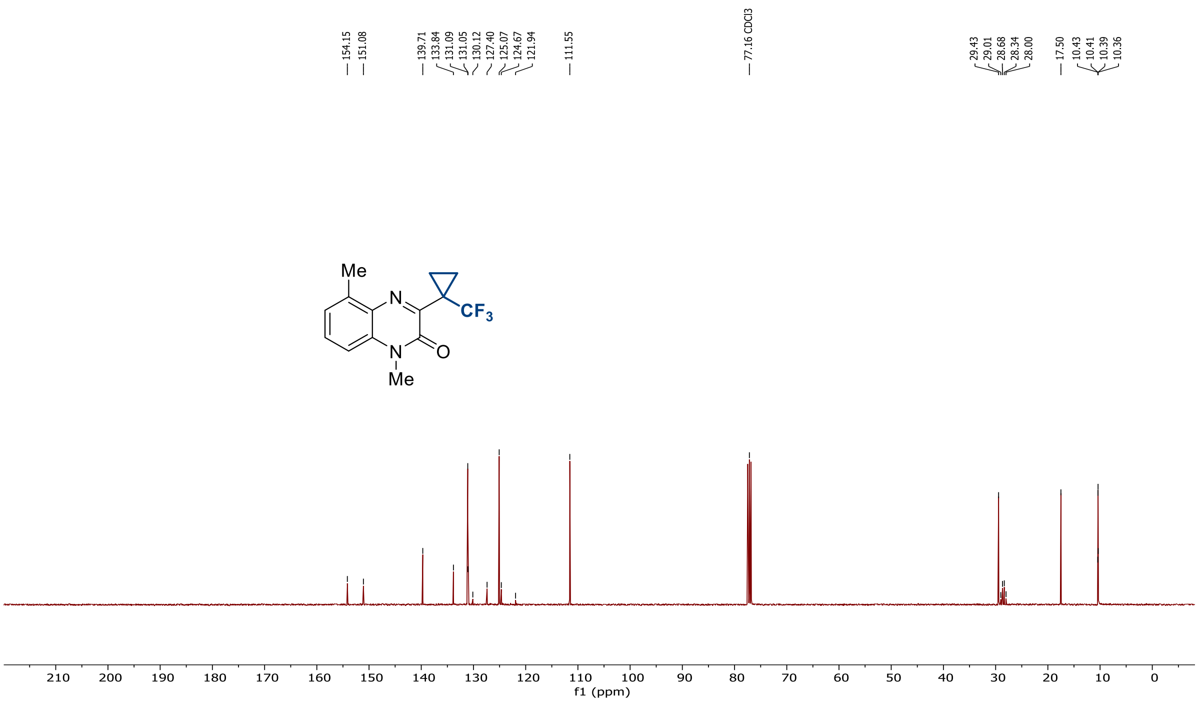


$^{19}F$ NMR (282 MHz, $CDCl_3$)

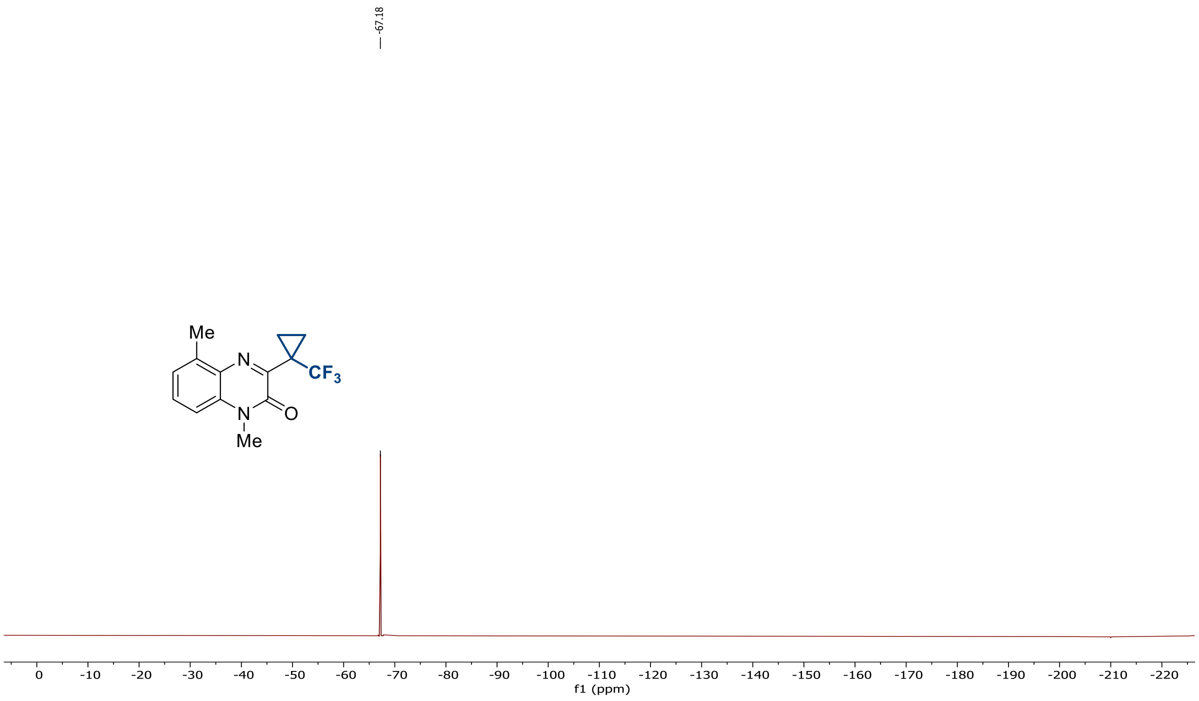

Compound **6g**:

$^1$H NMR (300 MHz, $CDCl_3$)

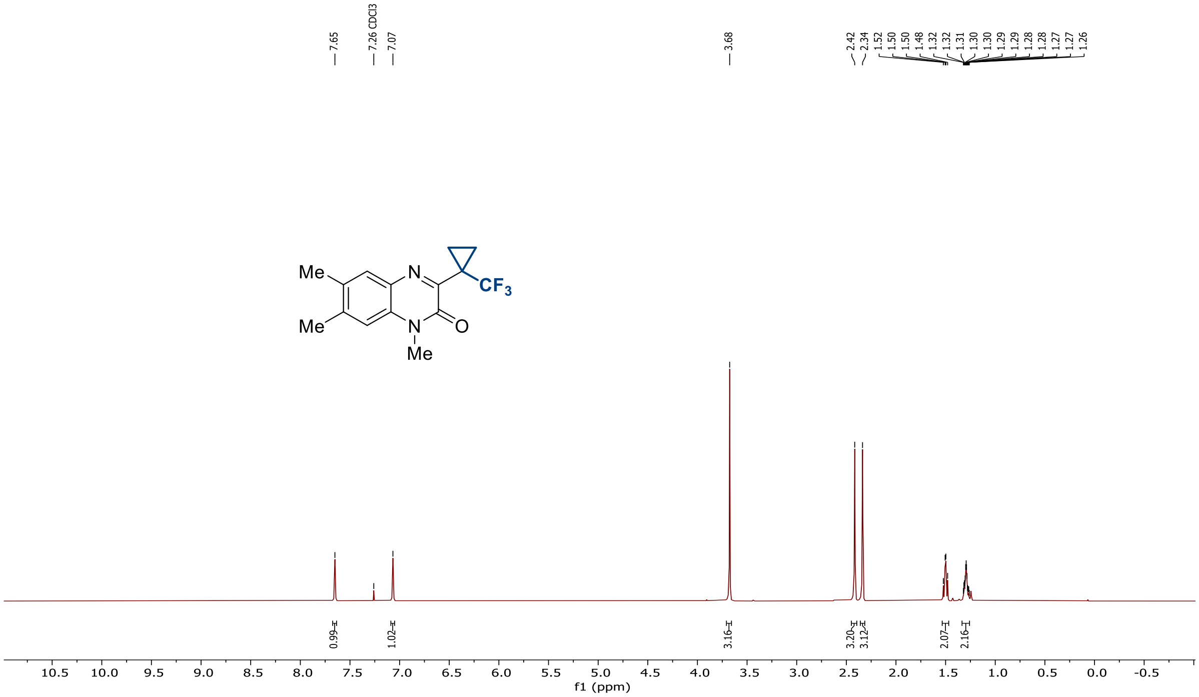


$^{13}$C{1H} NMR (101 MHz, $CDCl_3$)

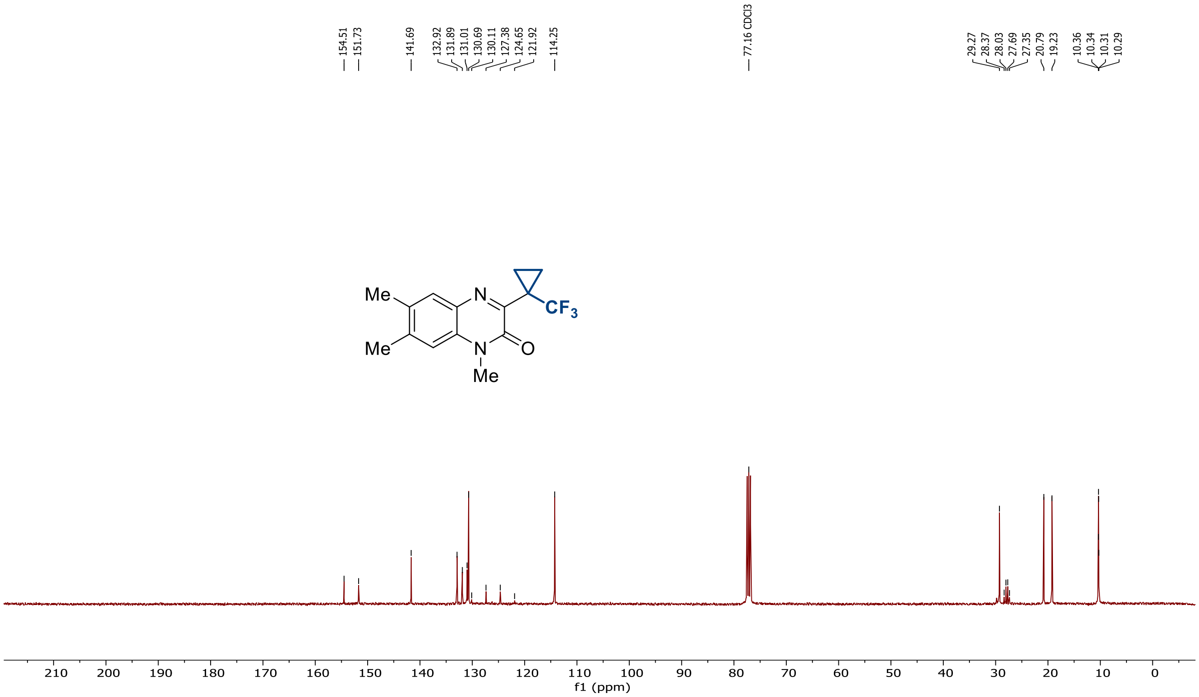

$^{19}F$ NMR (282 MHz, $CDCl_3$)

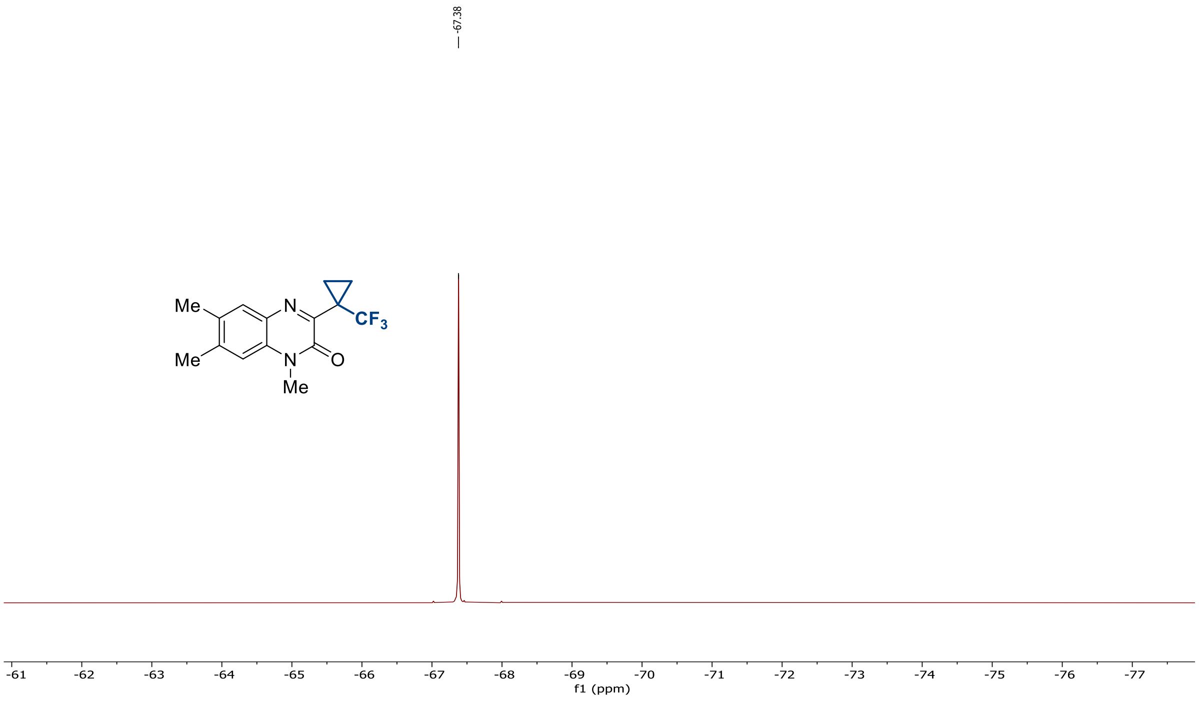


Compound **6h**:

$^{1}H$ NMR (300 MHz, $CDCl_3$)

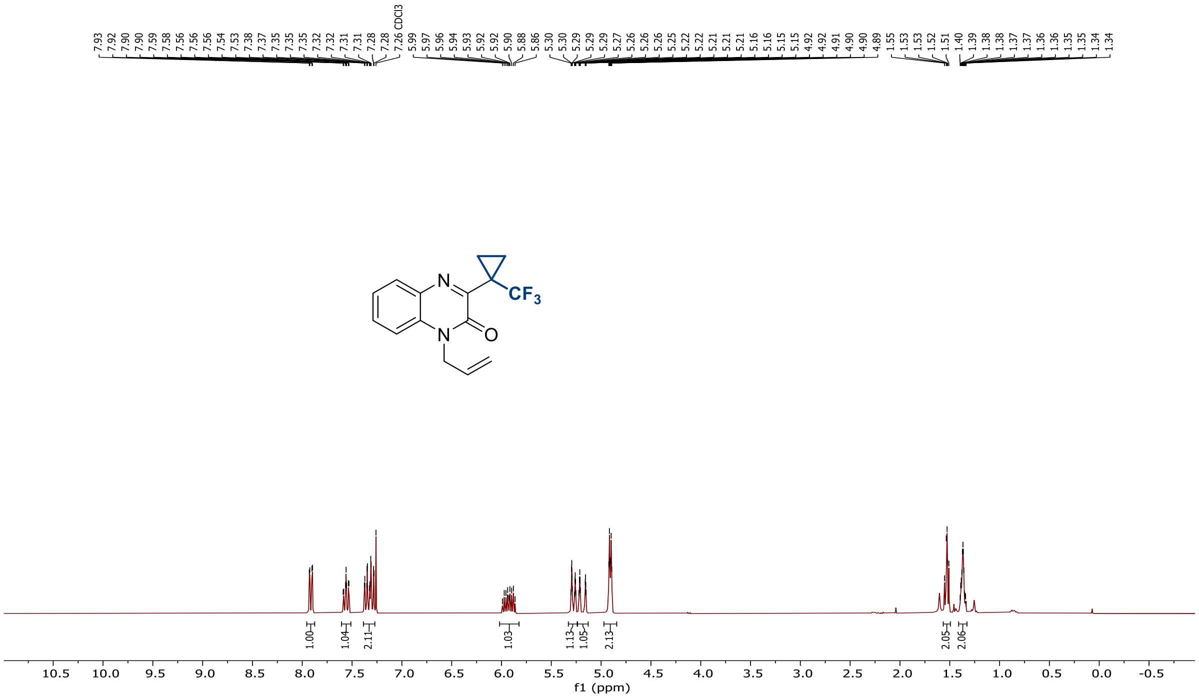

$^{13}C\{1H\}$ NMR (101 MHz, $CDCl_3$)

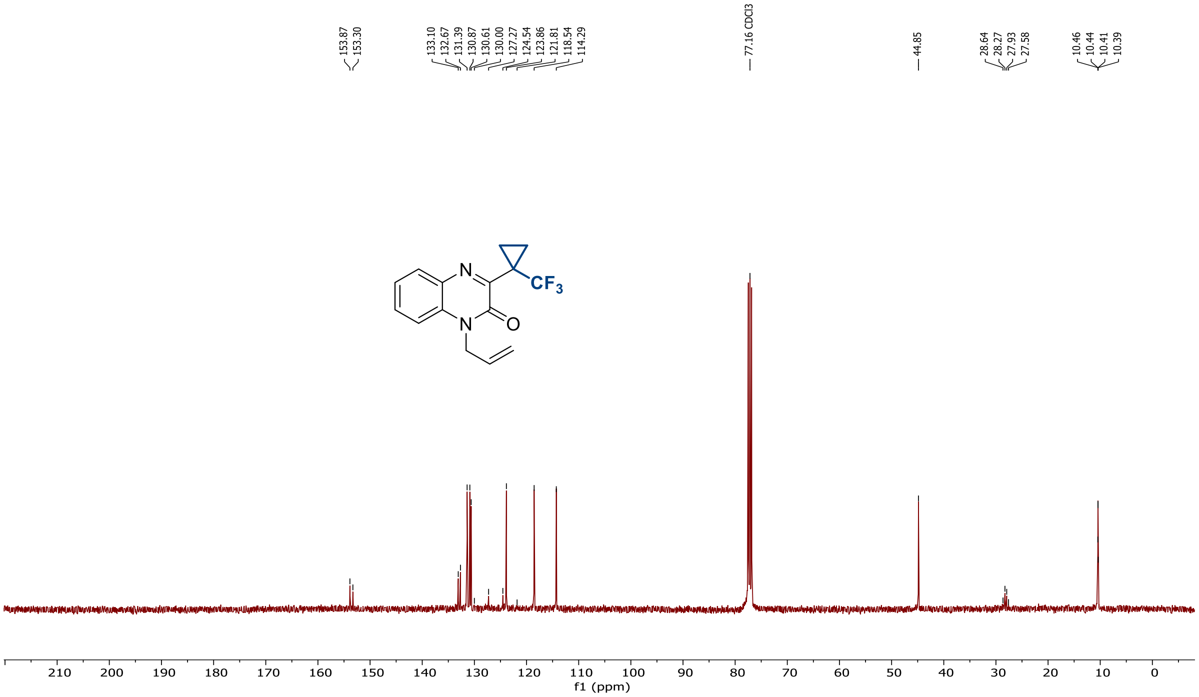


$^{19}F$ NMR (282 MHz, $CDCl_3$)

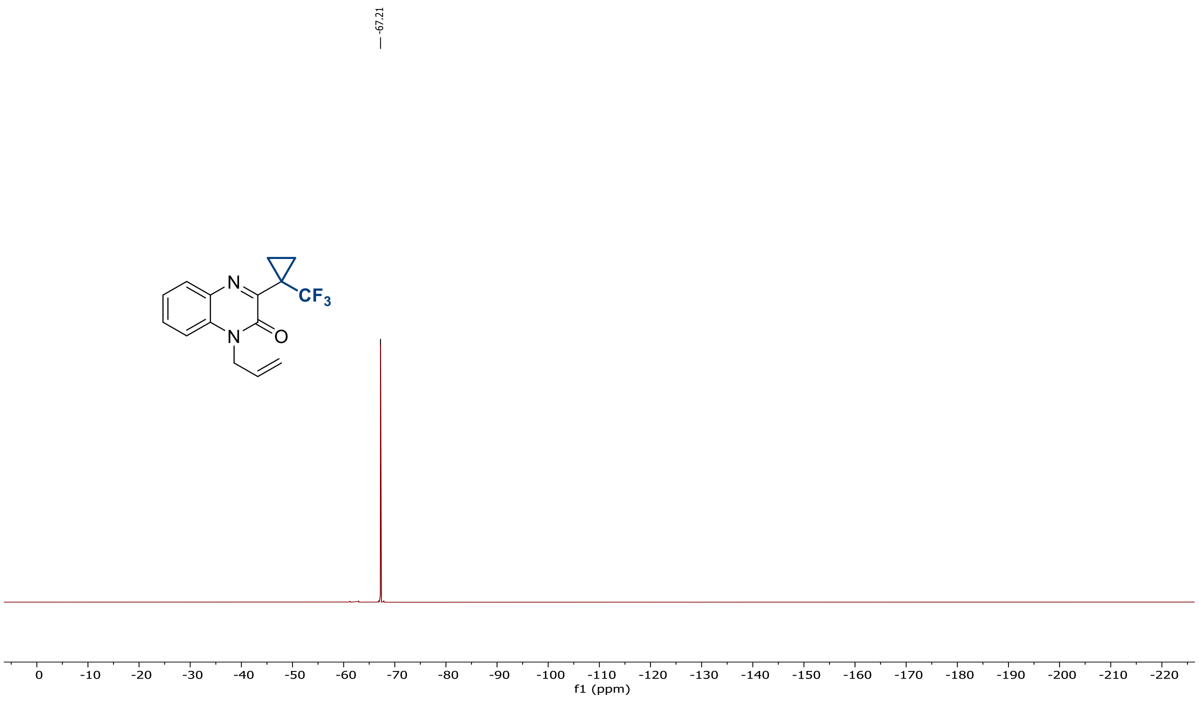

Compound **6i**:

$^{1}$H NMR (300 MHz, $CDCl_3$)

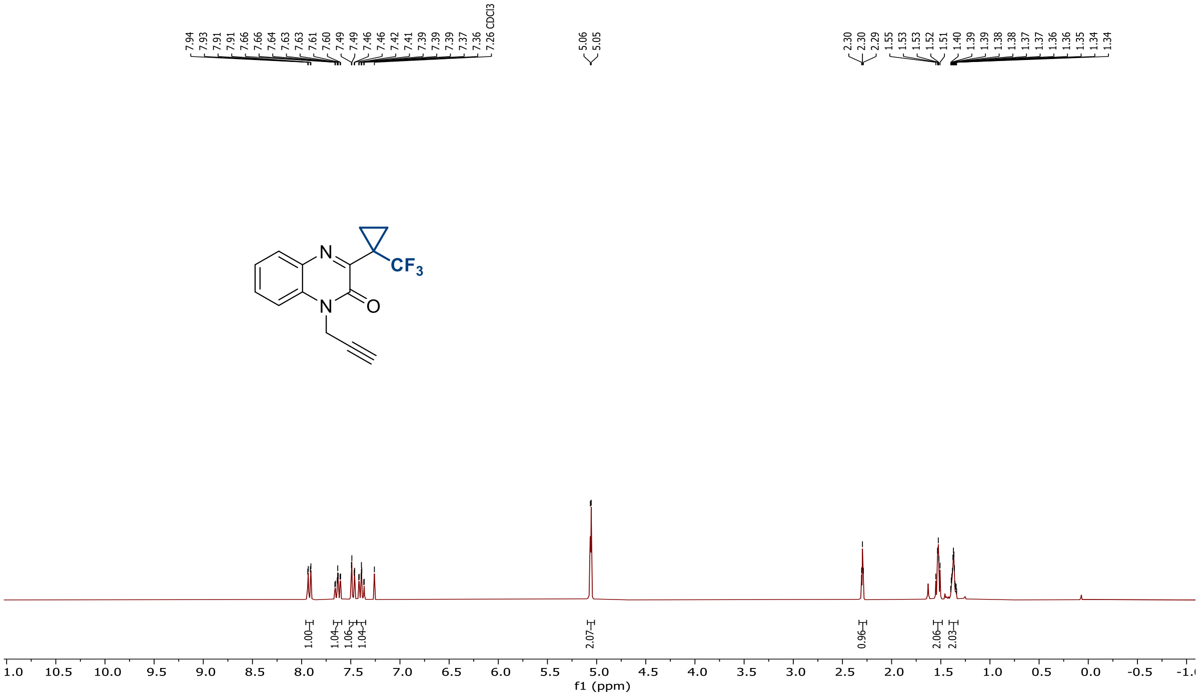


$^{13}$C{1H} NMR (101 MHz, $CDCl_3$)

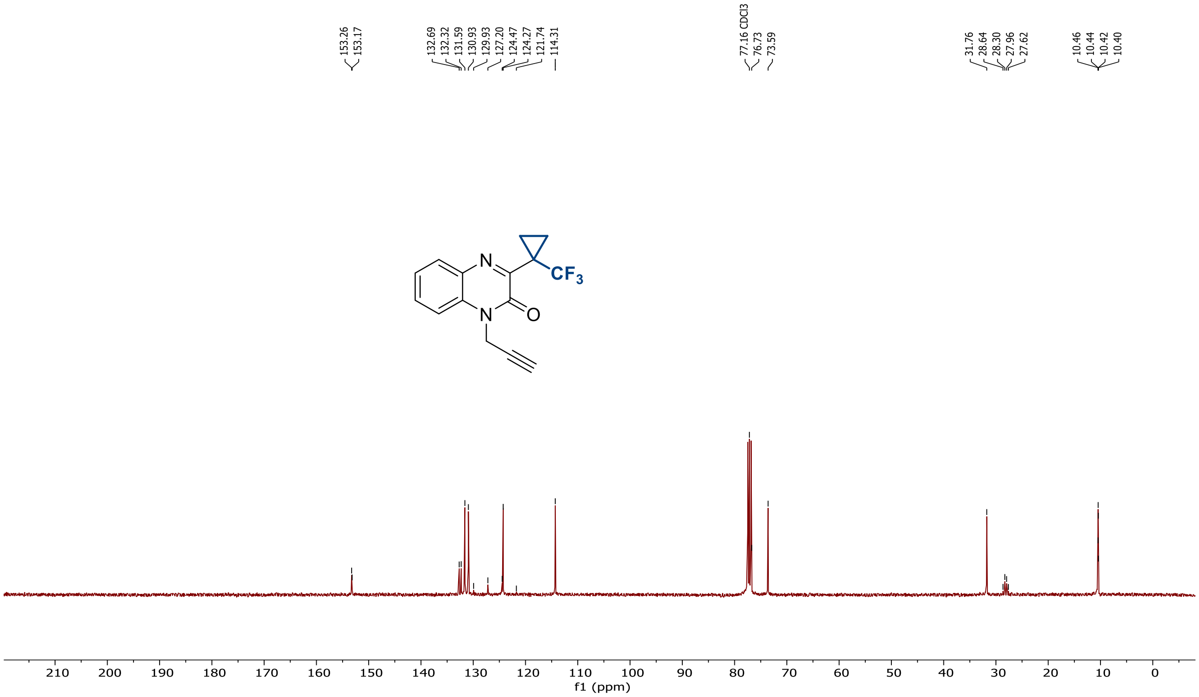

$^{19}F$ NMR (282 MHz, $CDCl_3$)

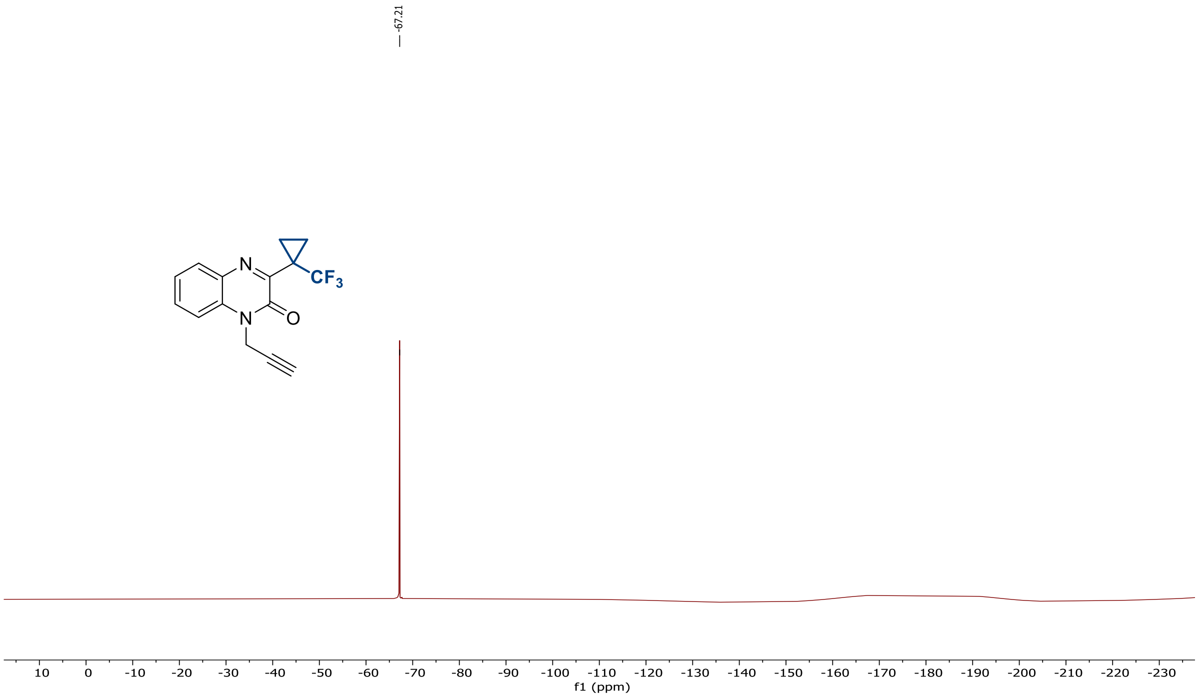


Compound **6j**:

$^{1}H$ NMR (400 MHz, $CDCl_3$)

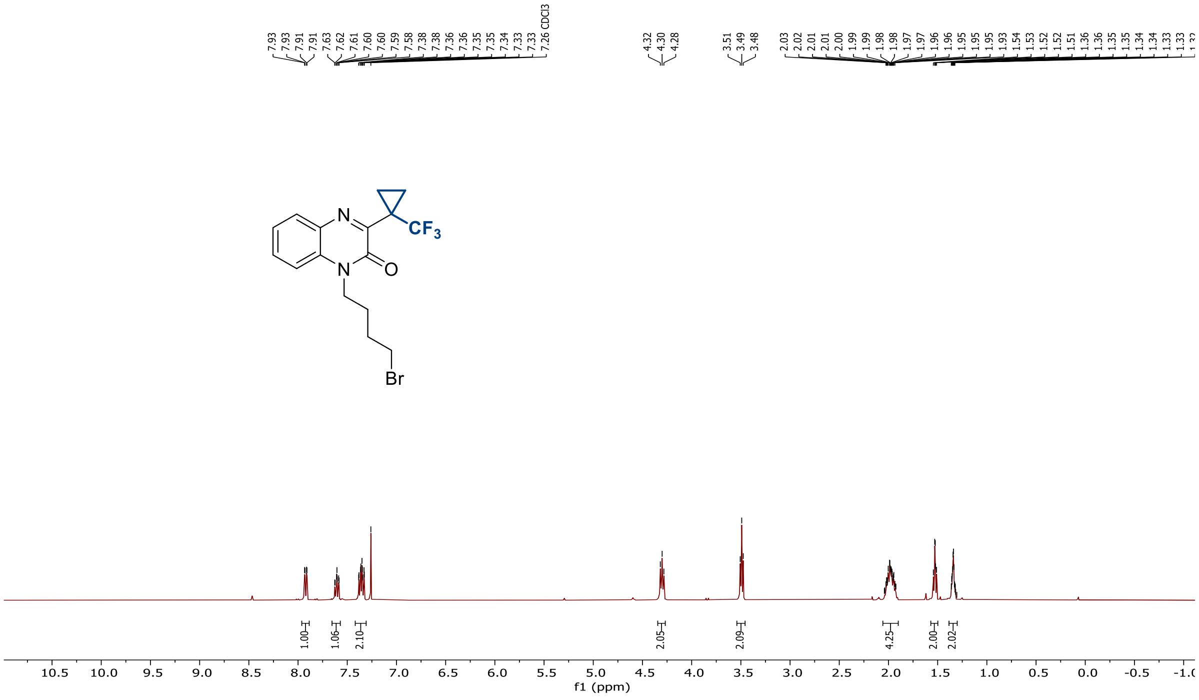

$^{13}$C{1H} NMR (101 MHz, $CDCl_3$)

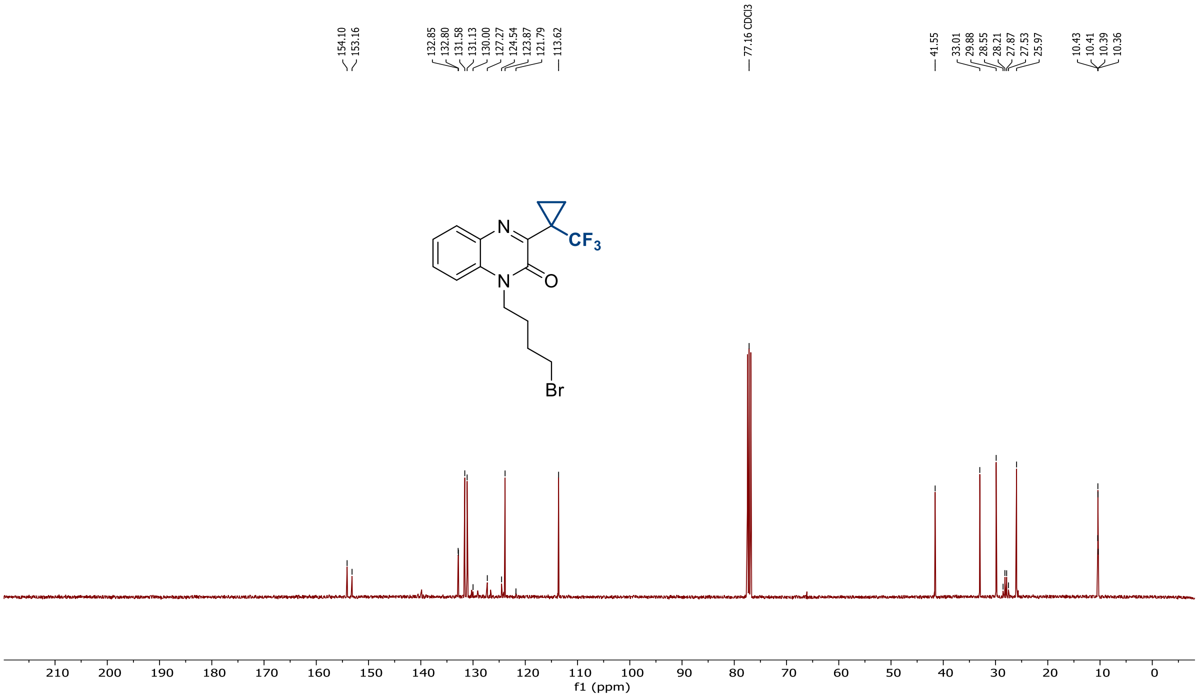


$^{19}$F NMR (377 MHz, $CDCl_3$)

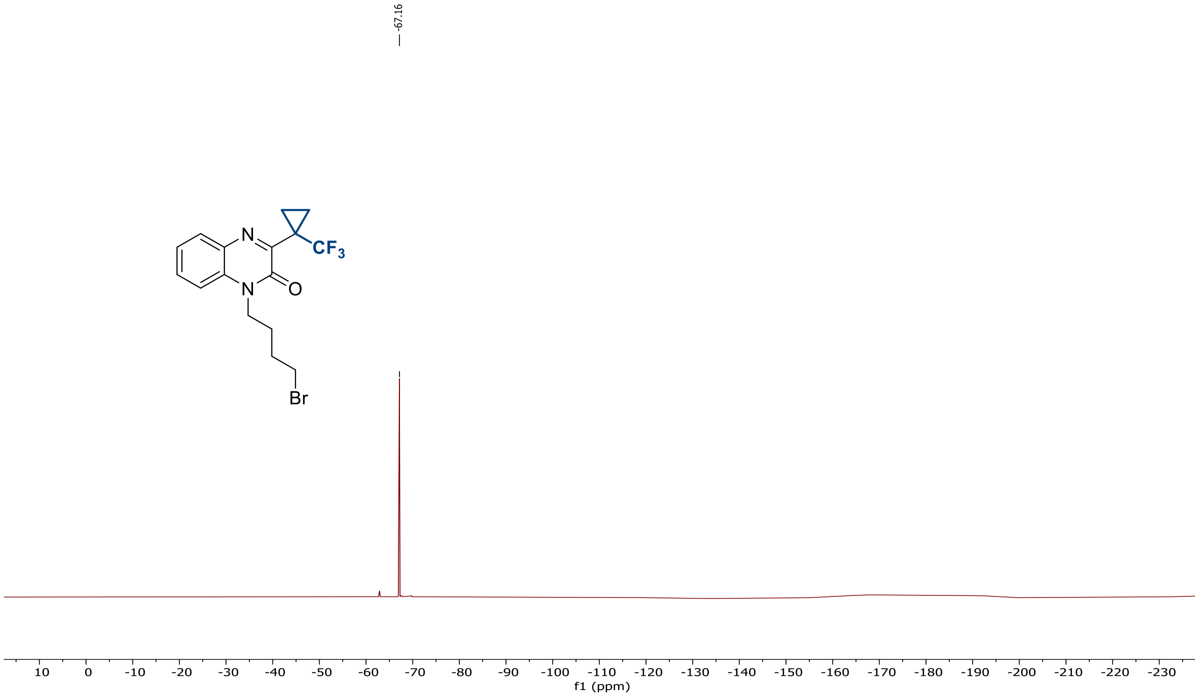

Compound **6k**:

$^1$H NMR (300 MHz, $CDCl_3$)

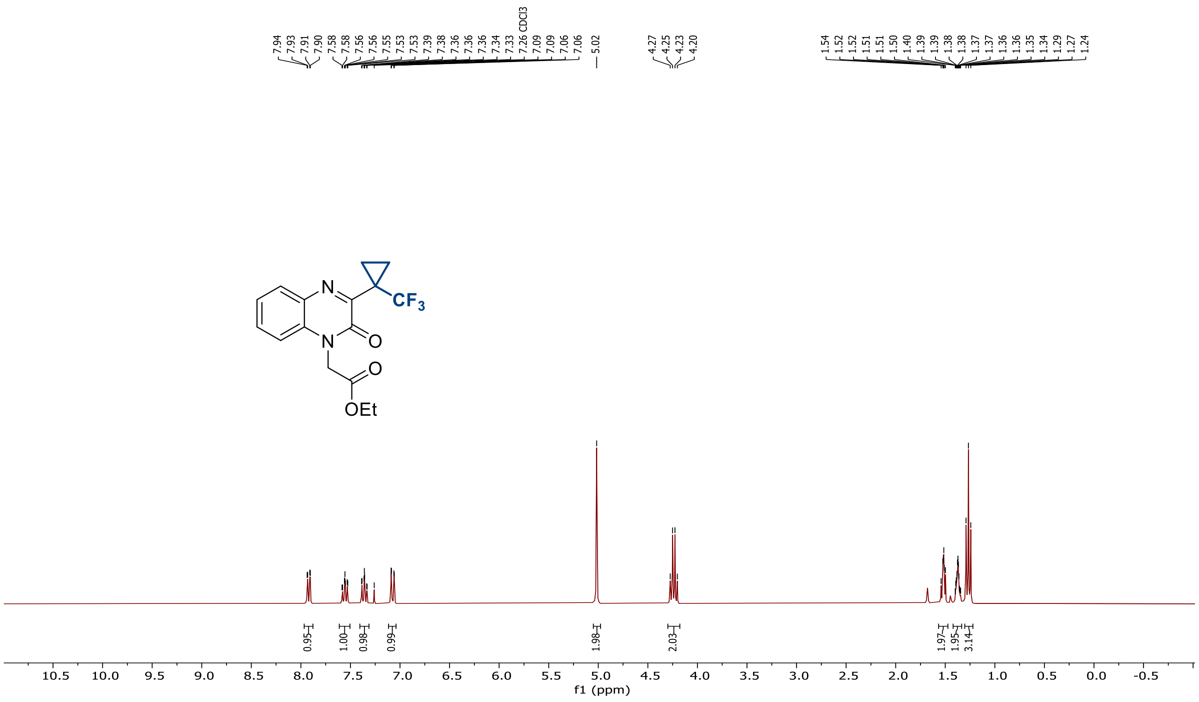


$^{13}$C{1H} NMR (101 MHz, $CDCl_3$)

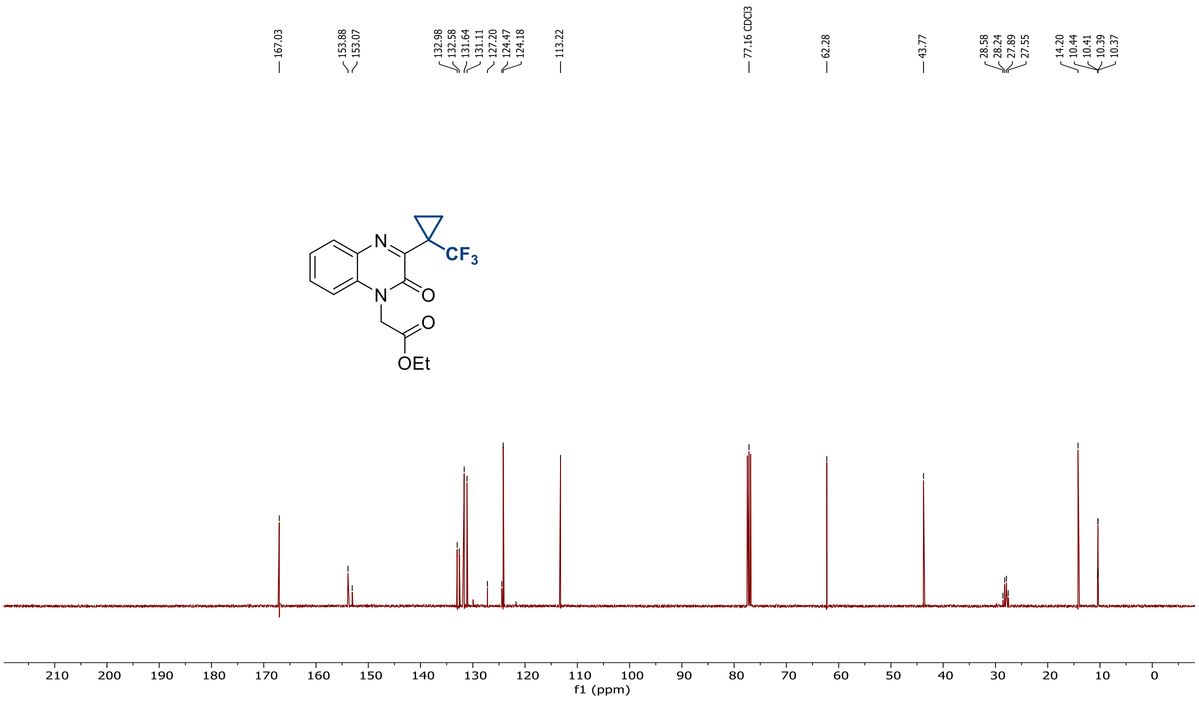

$^{19}F$ NMR (282 MHz, $CDCl_3$)

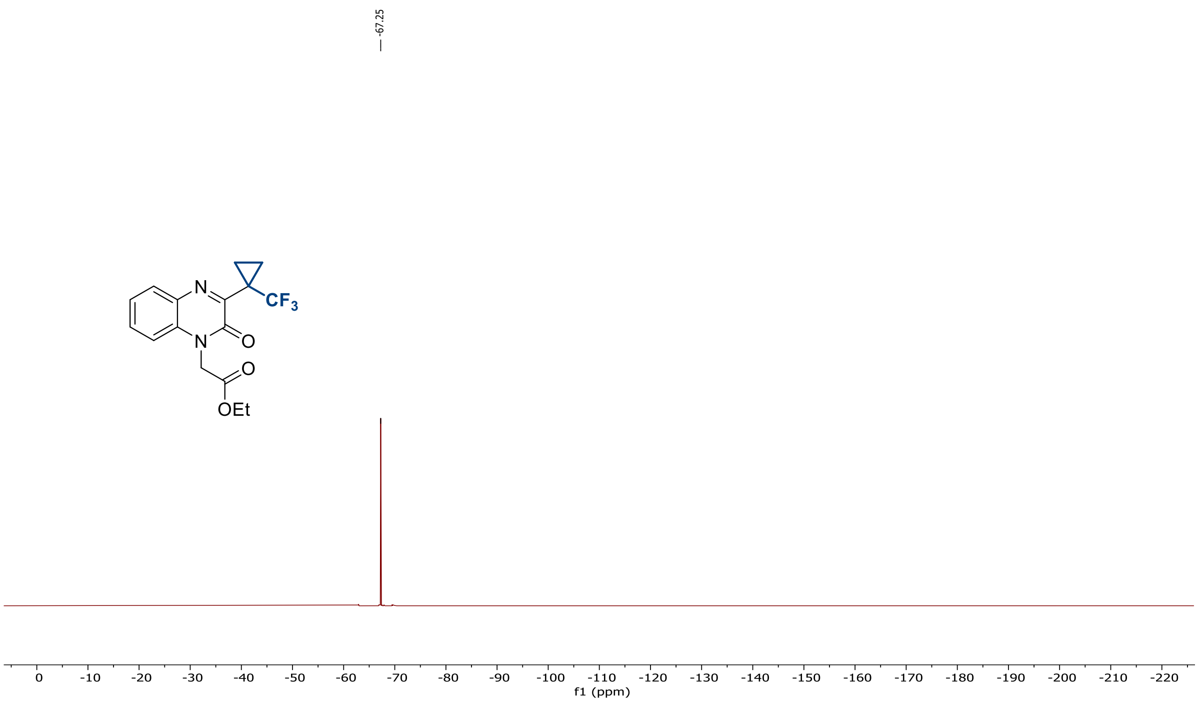


Compound **6l**:

$^{1}H$ NMR (300 MHz, $CDCl_3$)

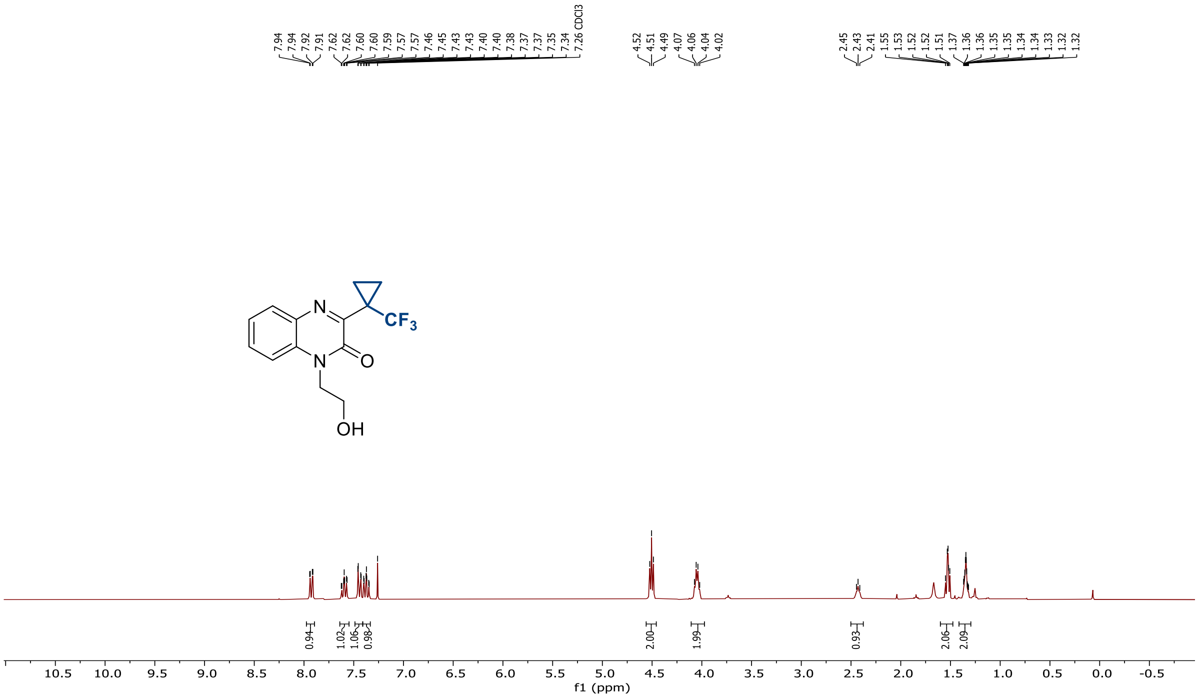

$^{13}C\{1H\}$ NMR (101 MHz, $CDCl_3$)

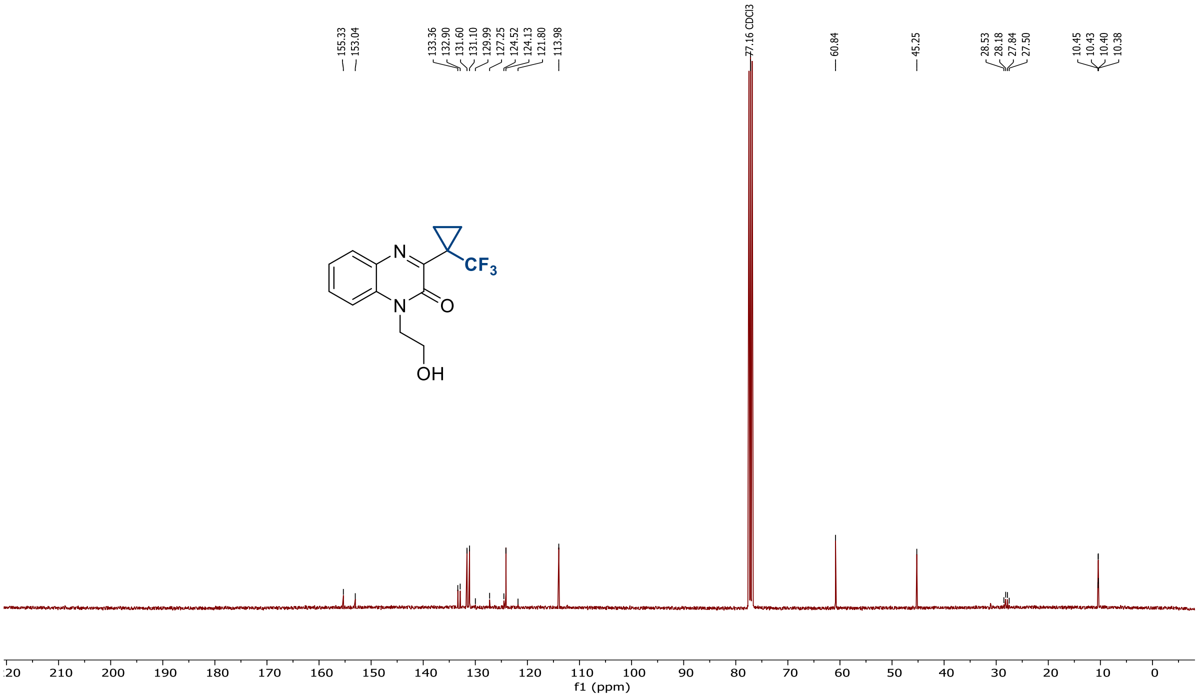


$^{19}F$ NMR (282 MHz, $CDCl_3$)

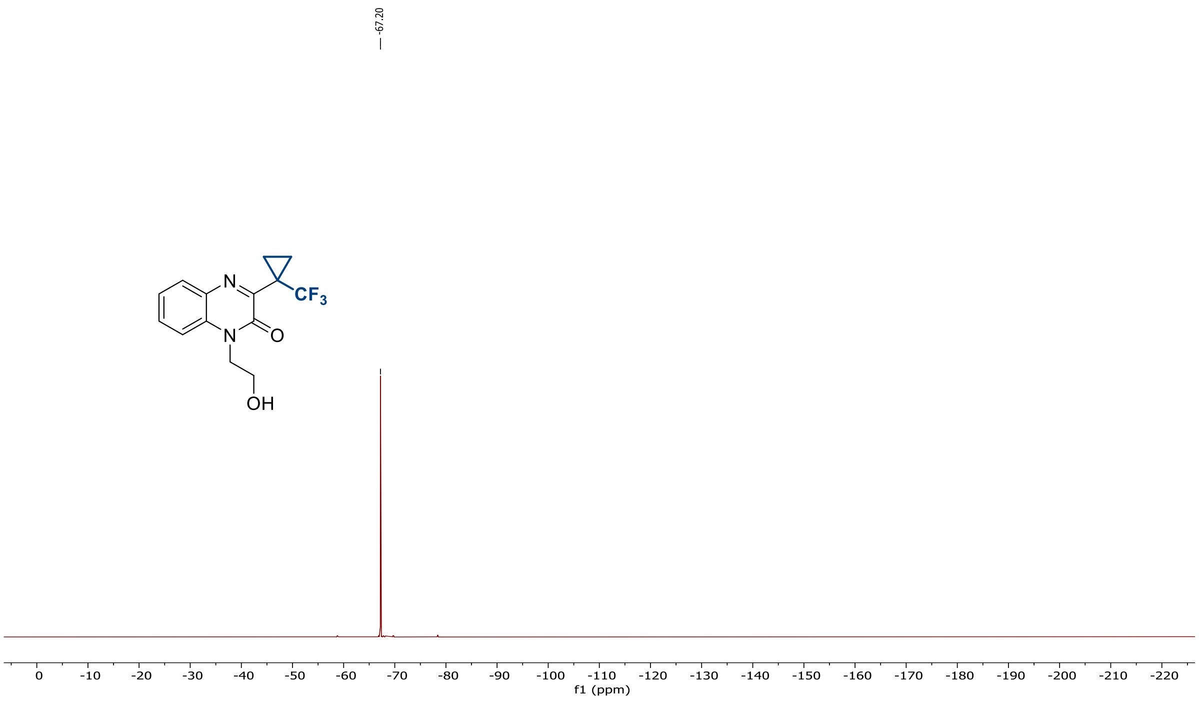

Compound **6m**:

$^{1}$H NMR (400 MHz, $CDCl_3$)

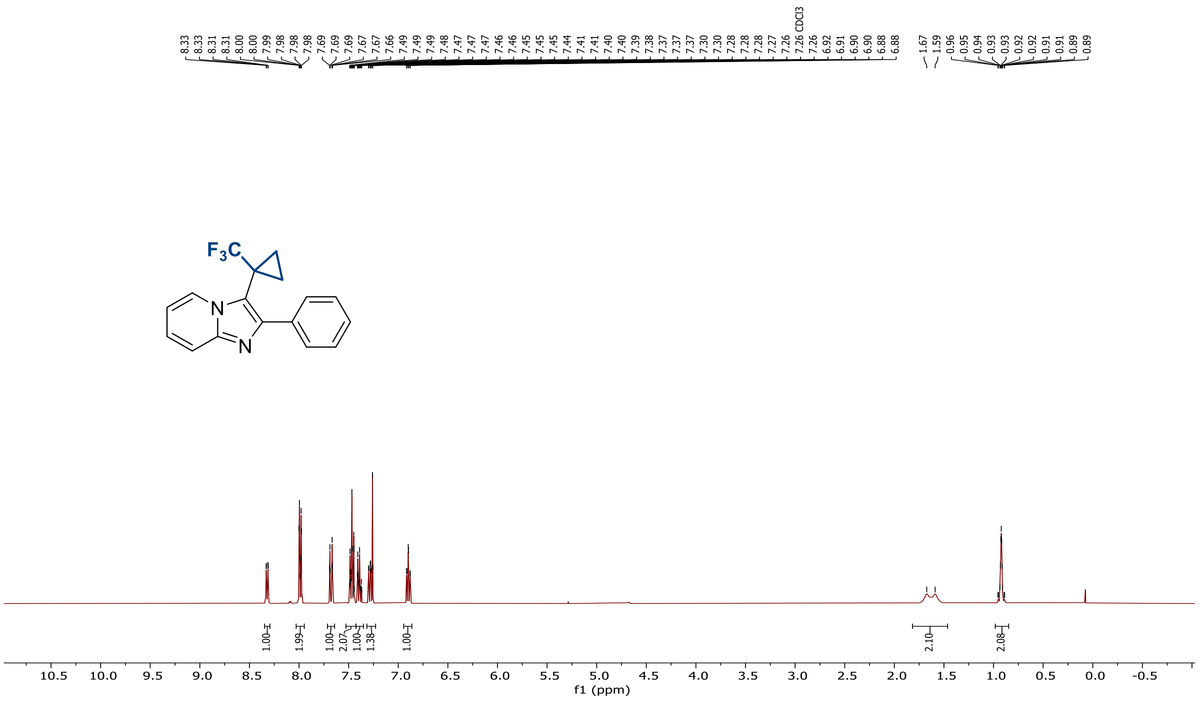


$^{13}$C{1H} NMR (101 MHz, $CDCl_3$)

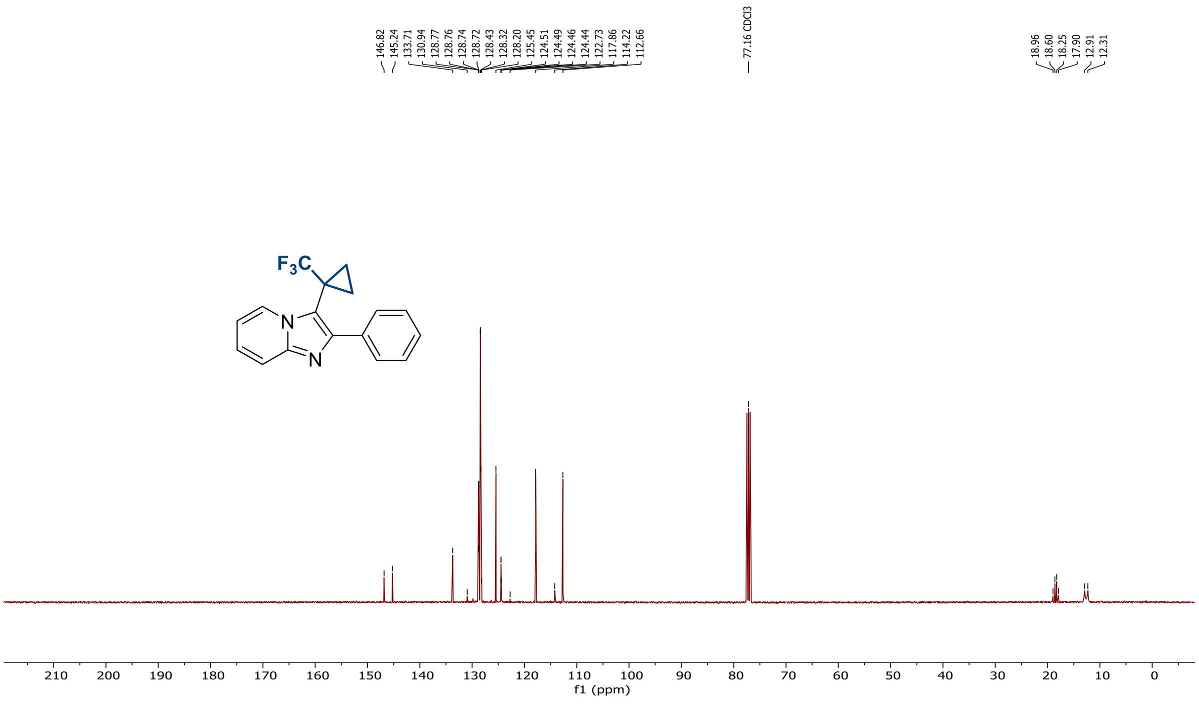

$^{19}F$ NMR (377 MHz, $CDCl_3$)

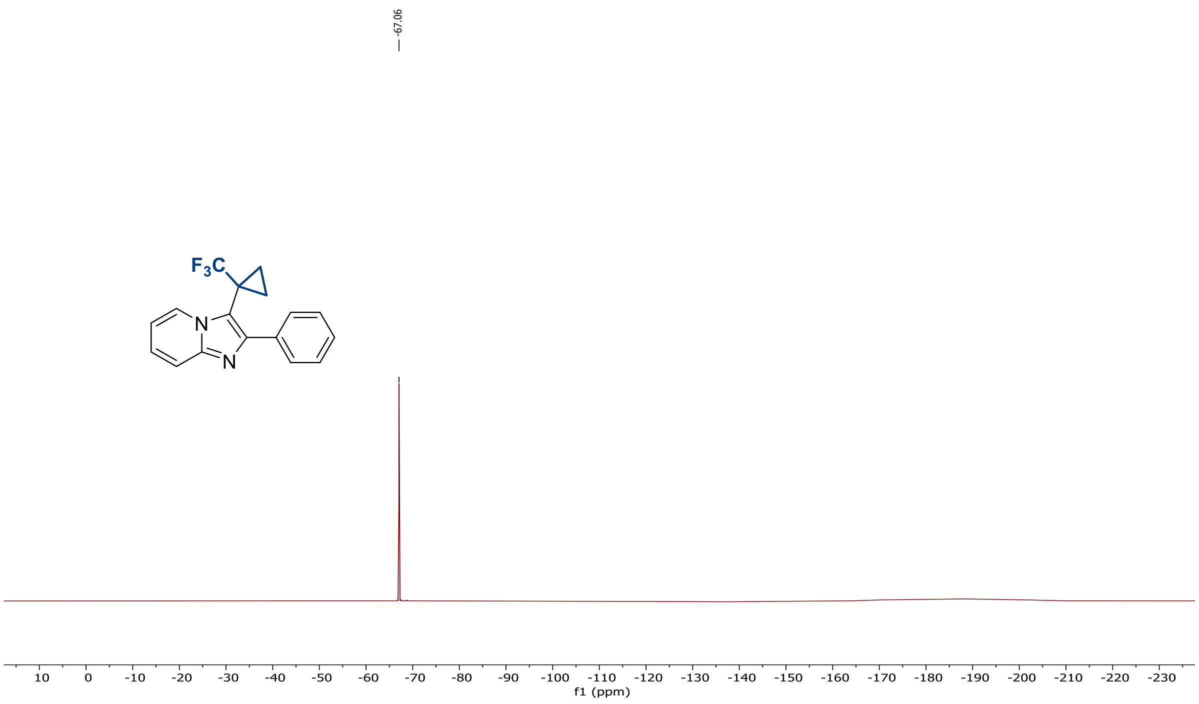


Compound **6n**:

$^{1}H$ NMR (300 MHz, $CDCl_3$)

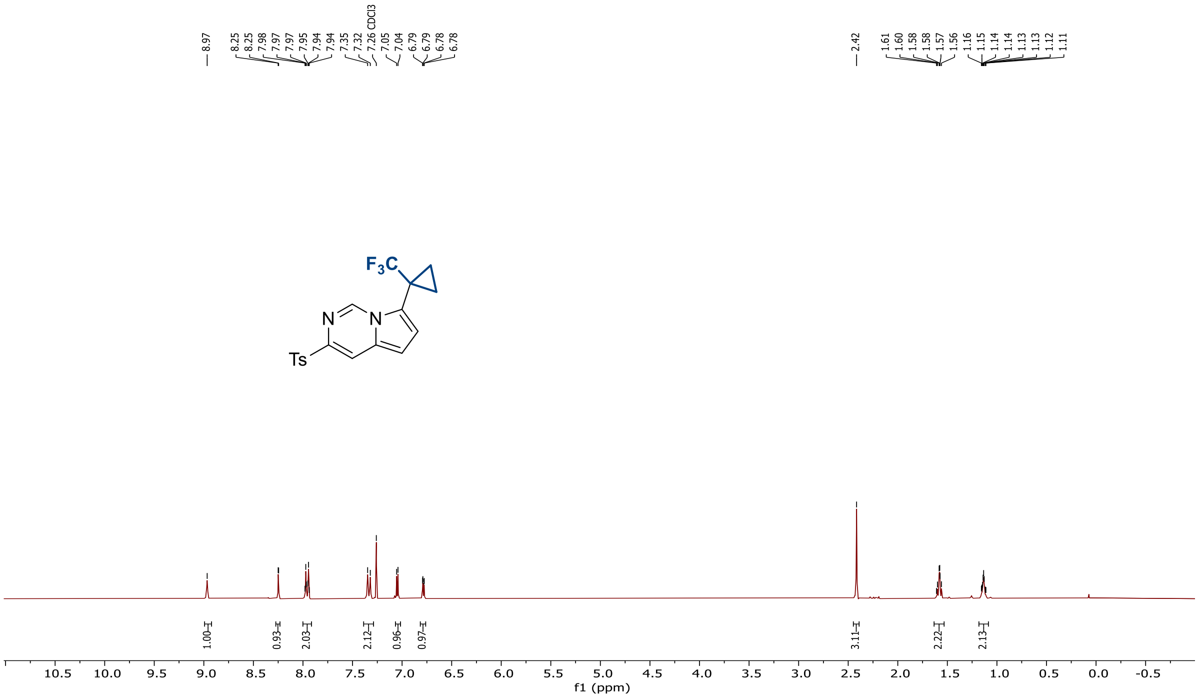

$^{13}C\{1H\}$ NMR (101 MHz, $CDCl_3$)

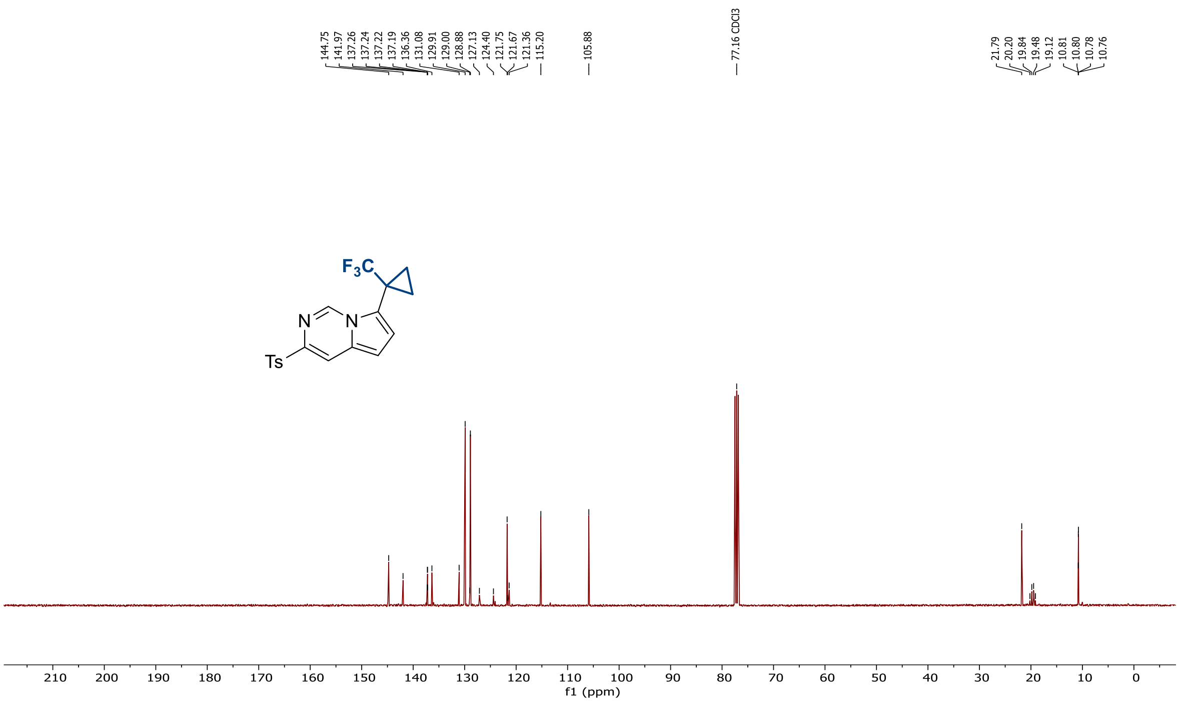


$^{19}F$ NMR (282 MHz, $CDCl_3$)

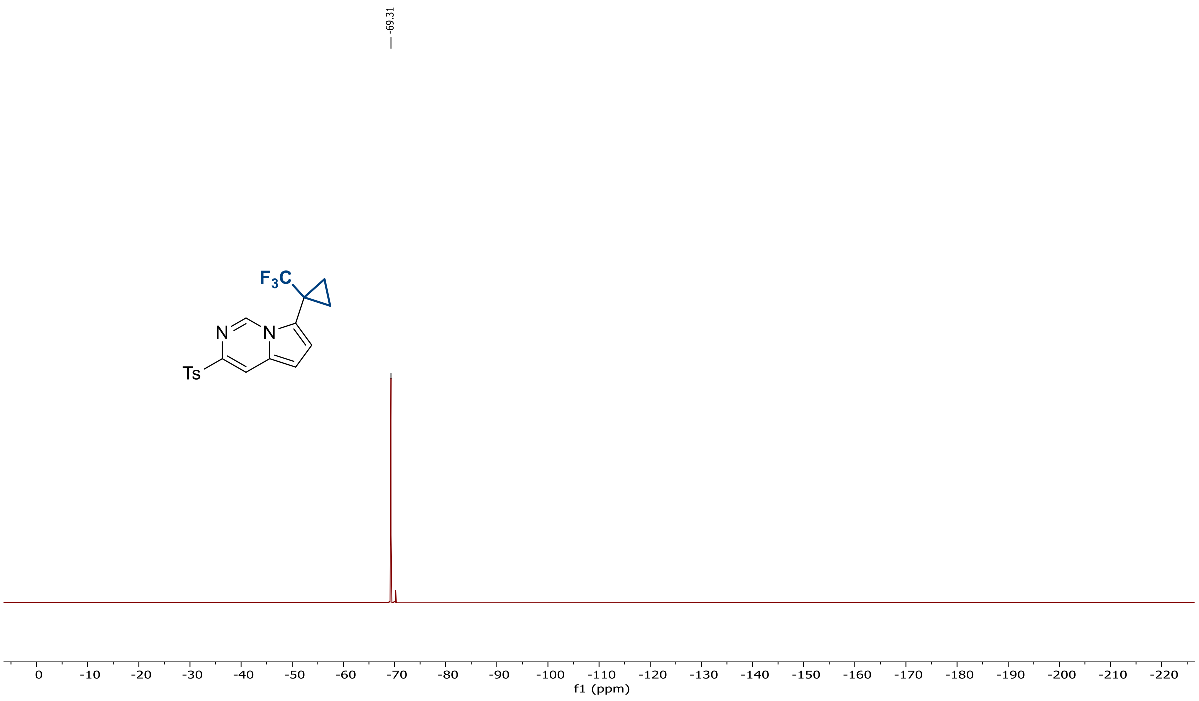

Compound **6o'**:

$^{1}H$ NMR (300 MHz, $CDCl_3$)

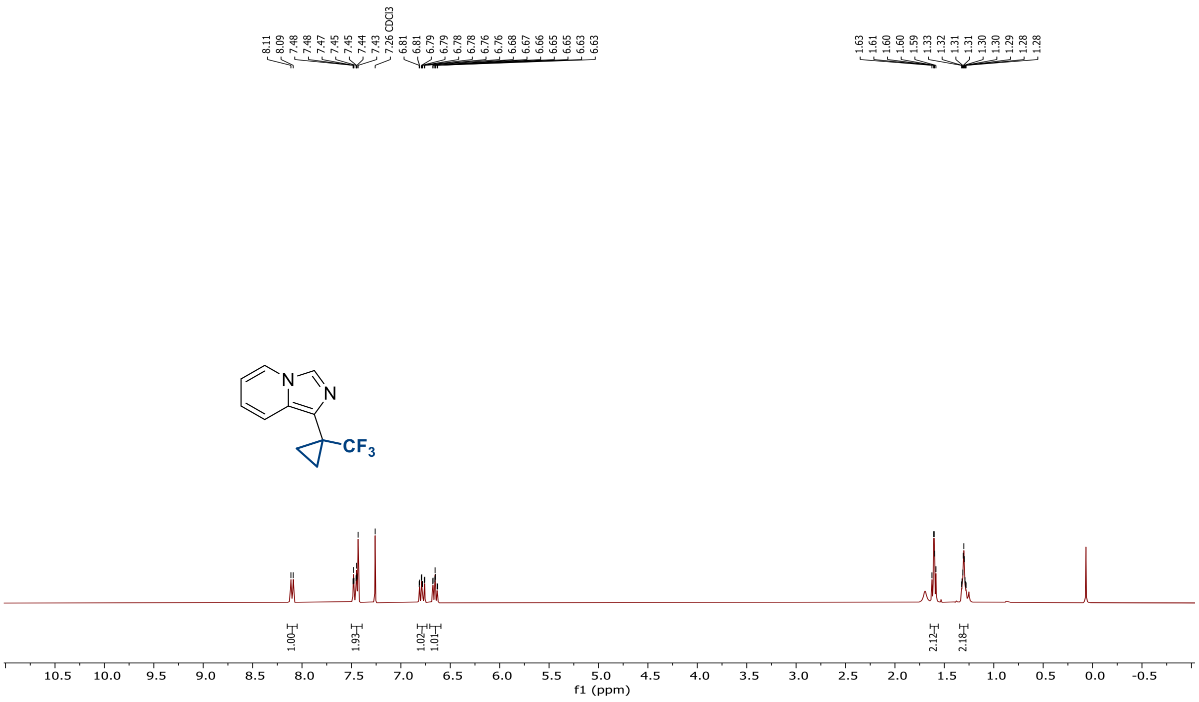

$^{13}C\{1H\}$ NMR (101 MHz, $CDCl_3$)

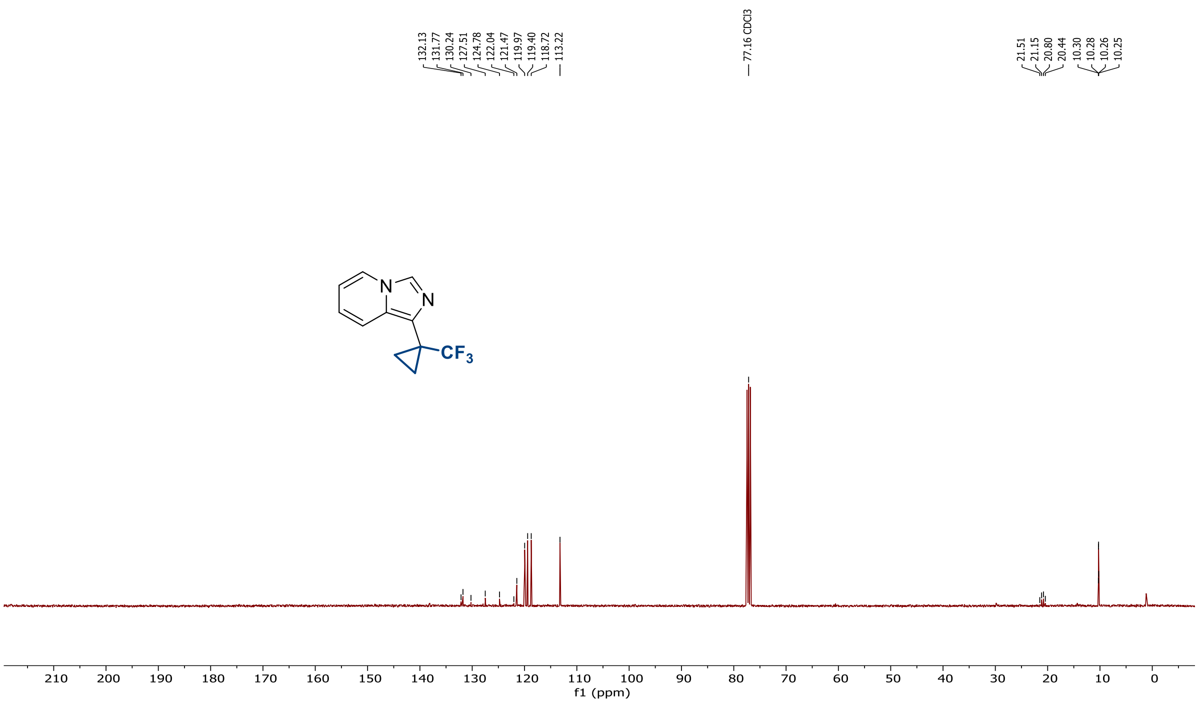

$^{19}F$ NMR (282 MHz, $CDCl_3$)

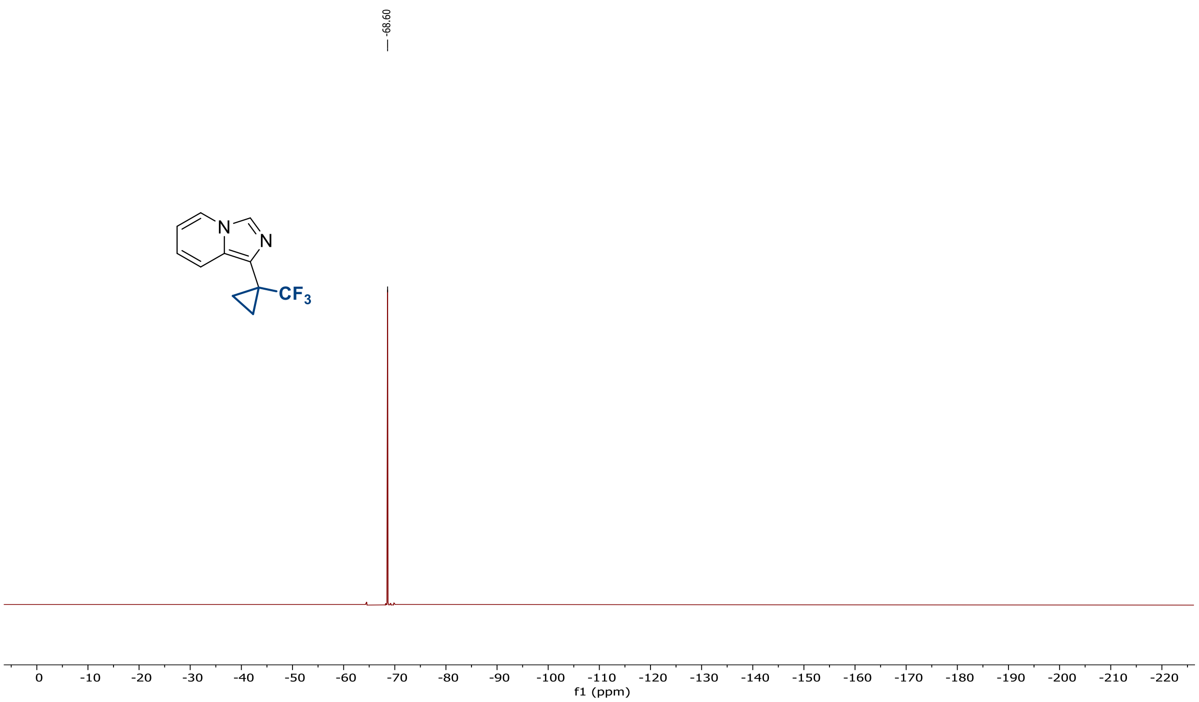


Compound **6p**:

$^{1}H$ NMR (400 MHz, $CDCl_3$)

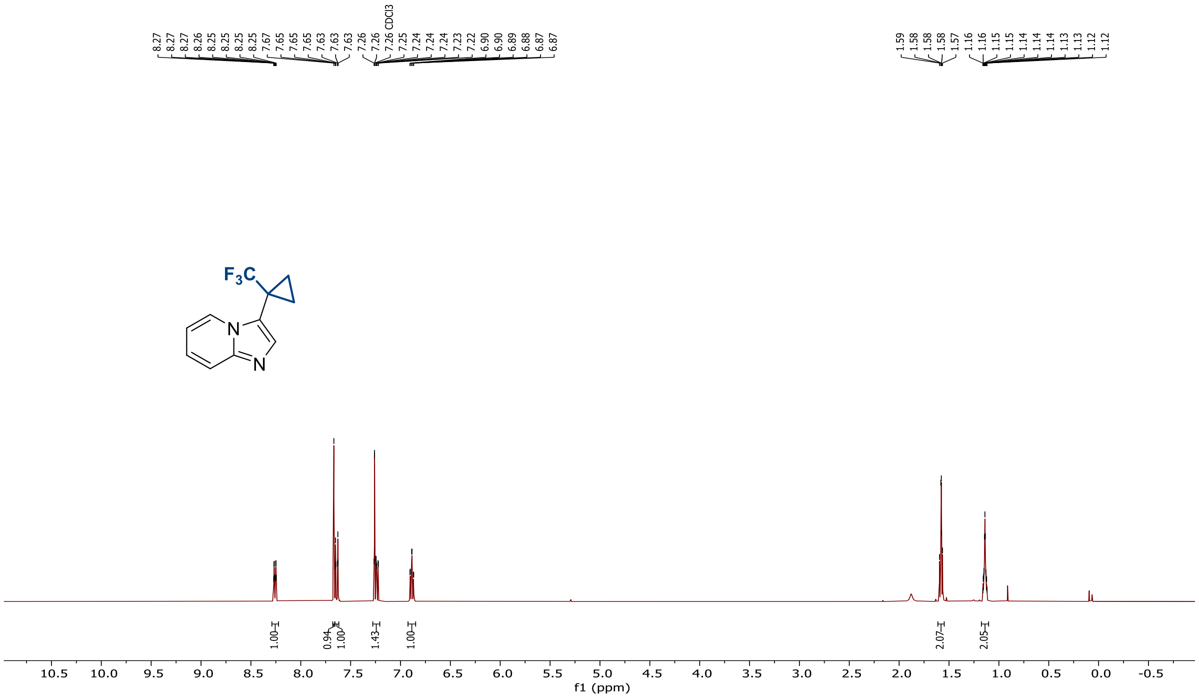

$^{13}C\{1H\}$ NMR (101 MHz, $CDCl_3$)

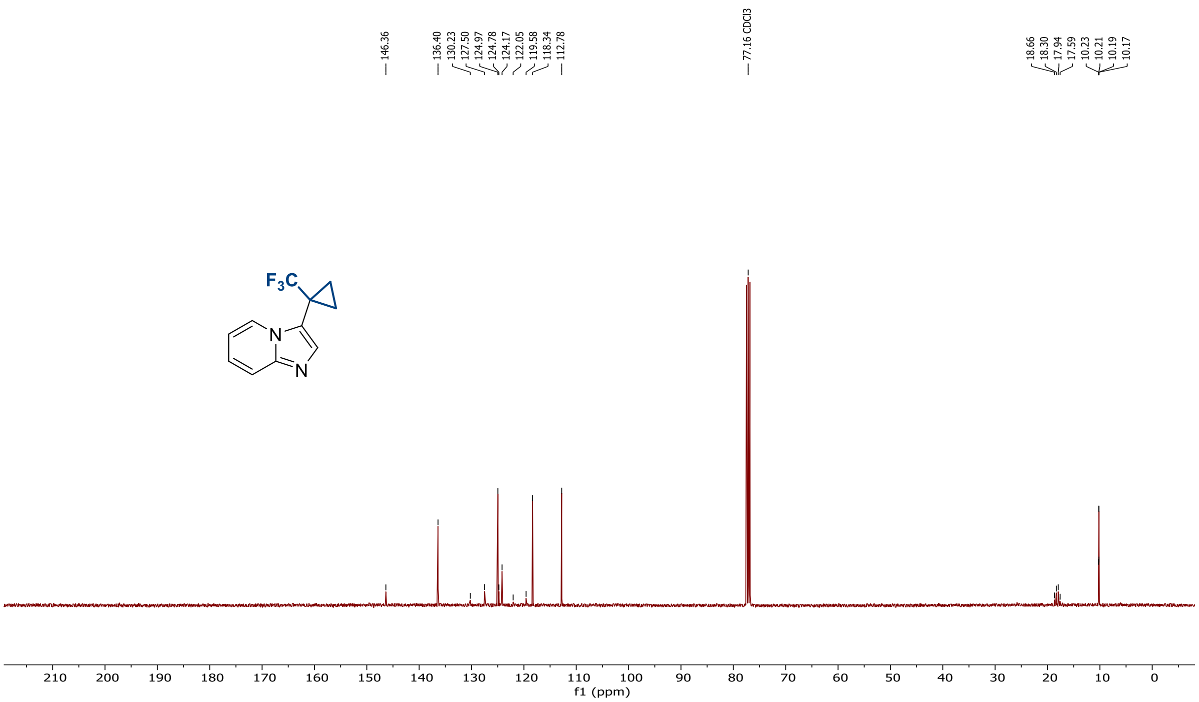


$^{19}F$ NMR (377 MHz, $CDCl_3$)

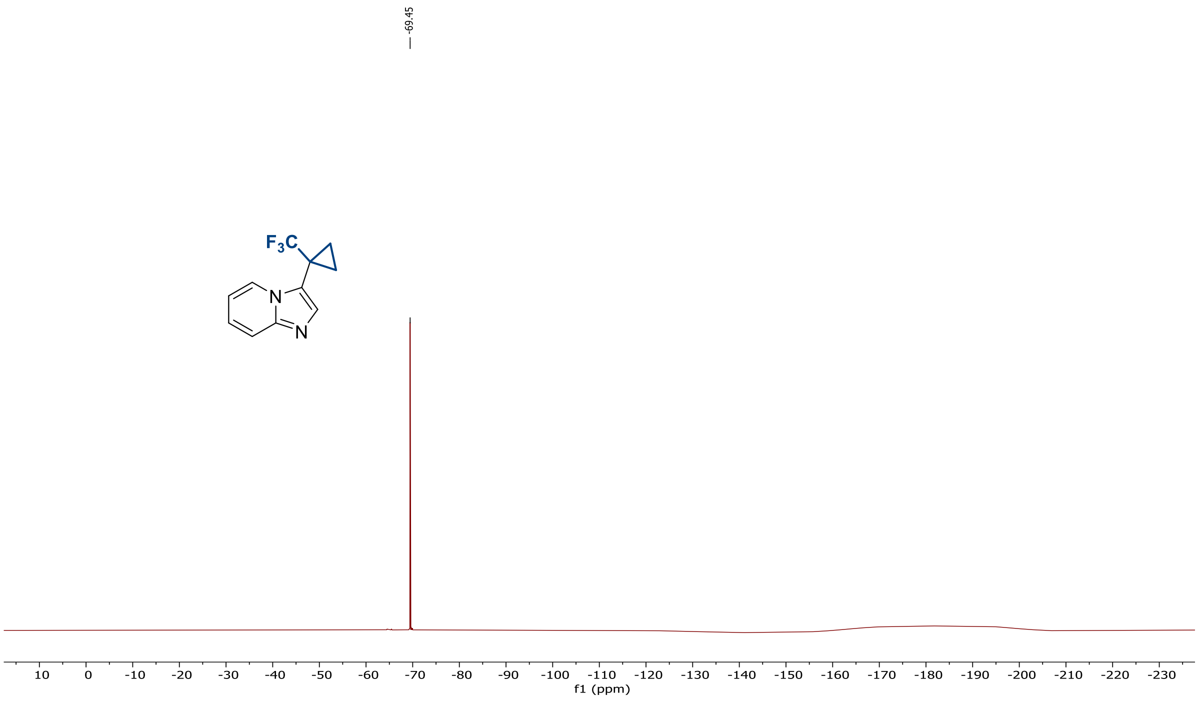

Compound **6q**:

$^{1}H$ NMR (300 MHz, $CDCl_3$)

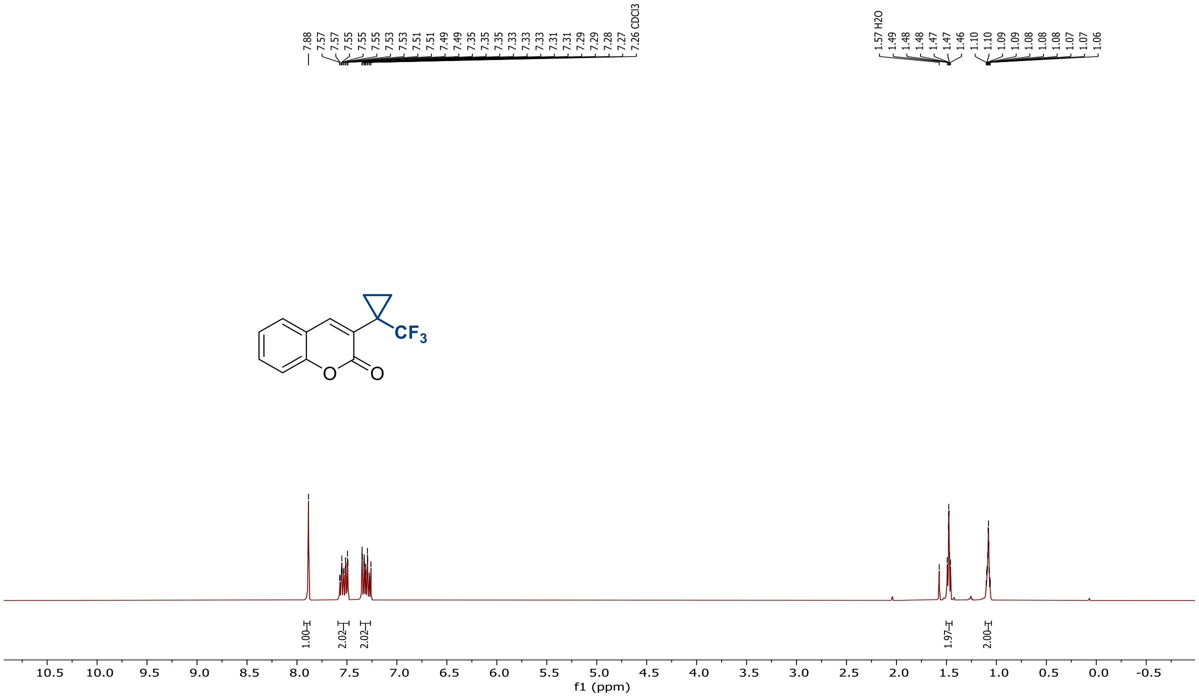


$^{13}C\{1H\}$ NMR (101 MHz, $CDCl_3$)

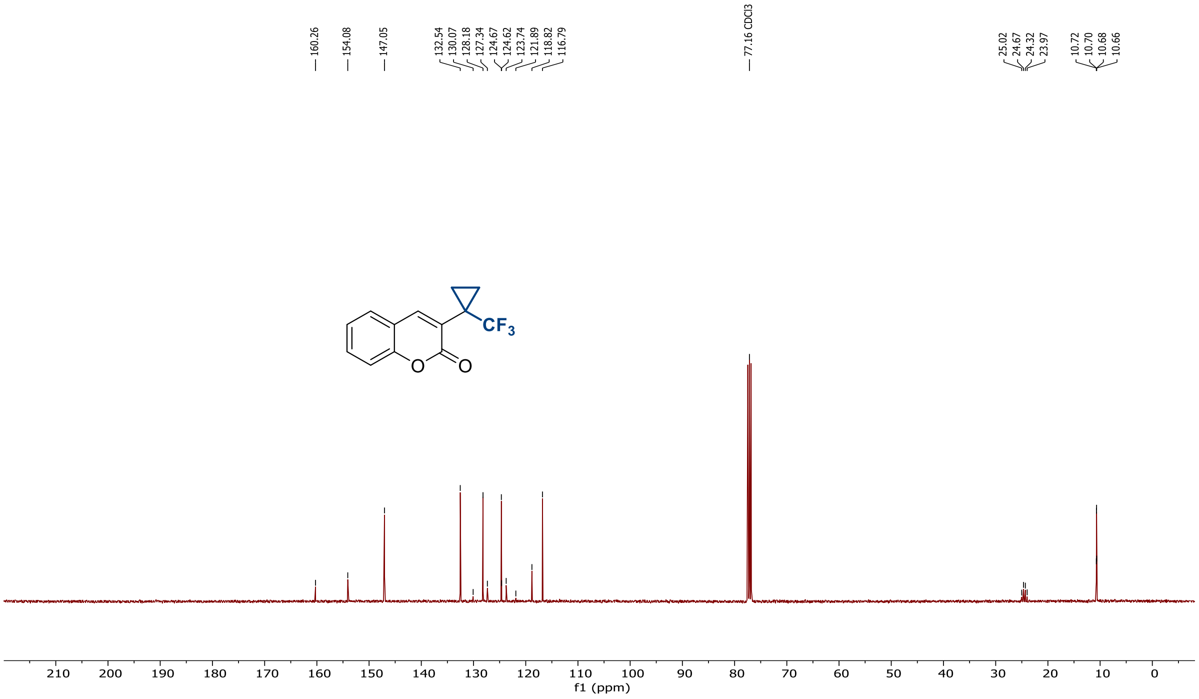

$^{19}F$ NMR (282 MHz, $CDCl_3$)

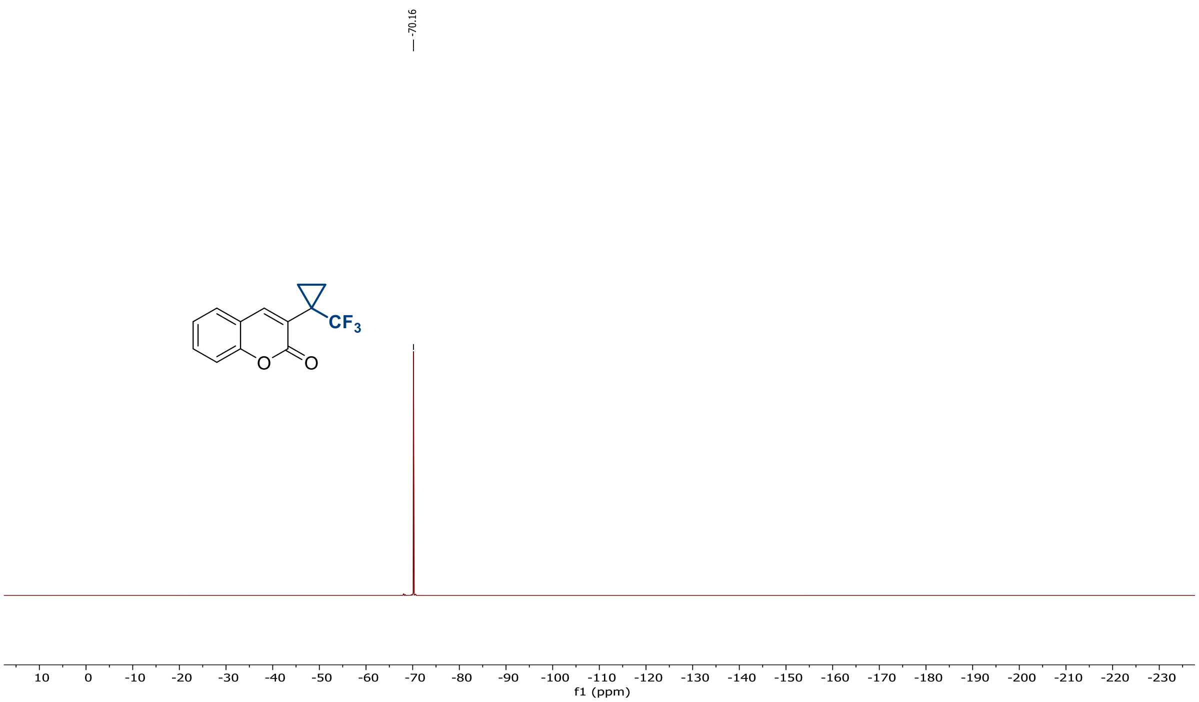


Compound **6r**:

$^{1}H$ NMR (400 MHz, $CD_3CN$)

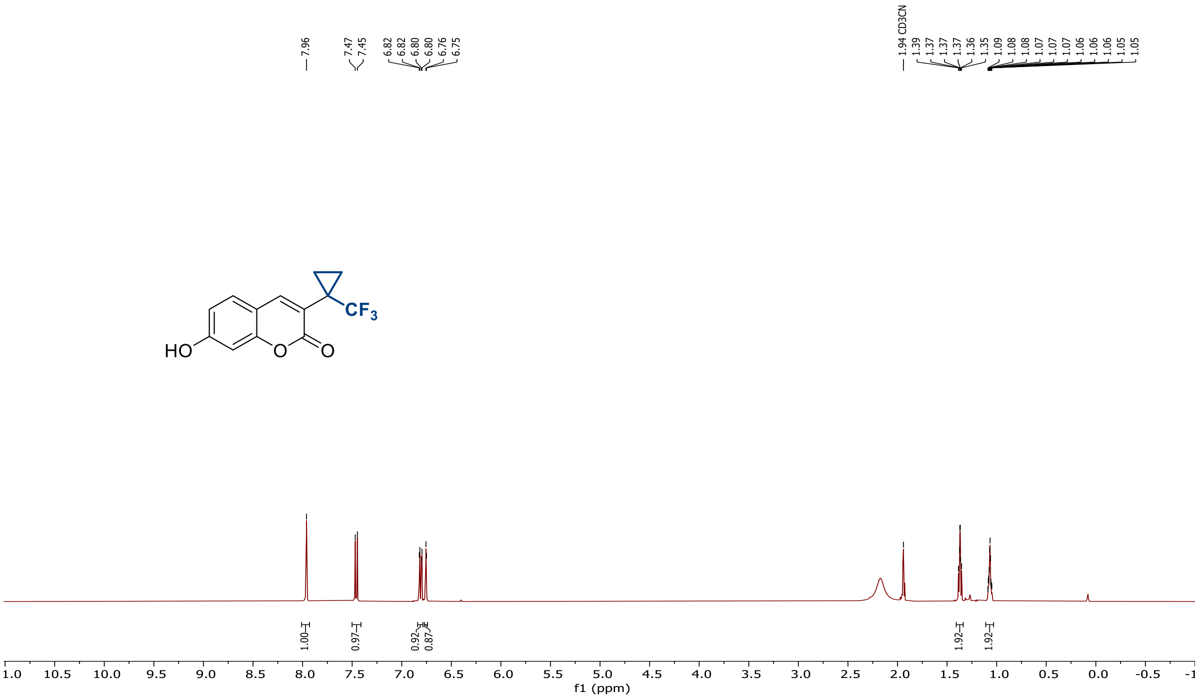

$^{13}C\{1H\}$ NMR (101 MHz, $CD_3CN$)

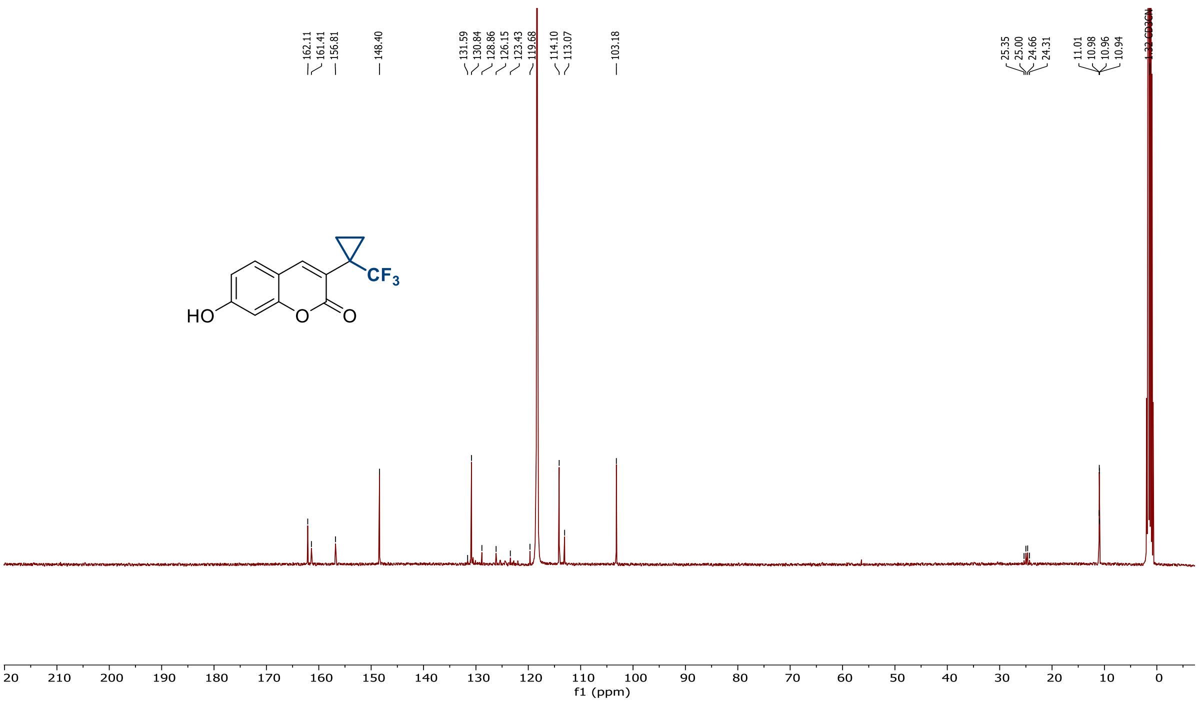

$^{19}F$ NMR (377 MHz, $CD_3CN$)

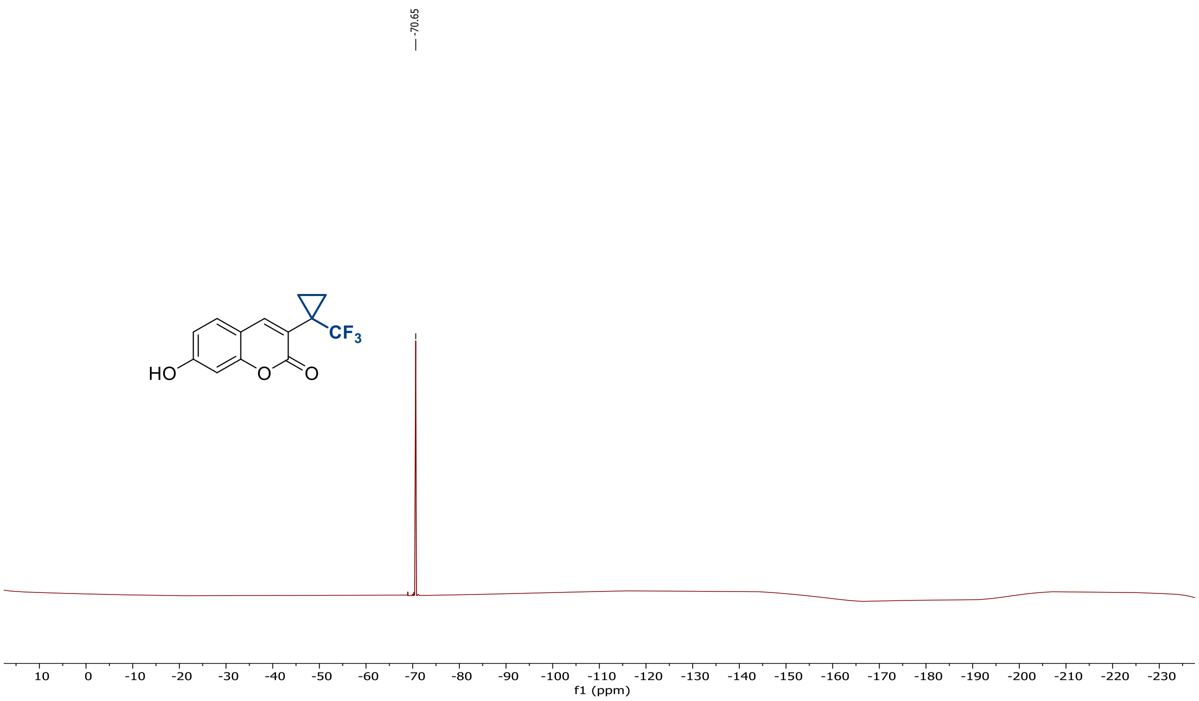

Compound **6s**:

$^1$H NMR (400 MHz, $CDCl_3$)

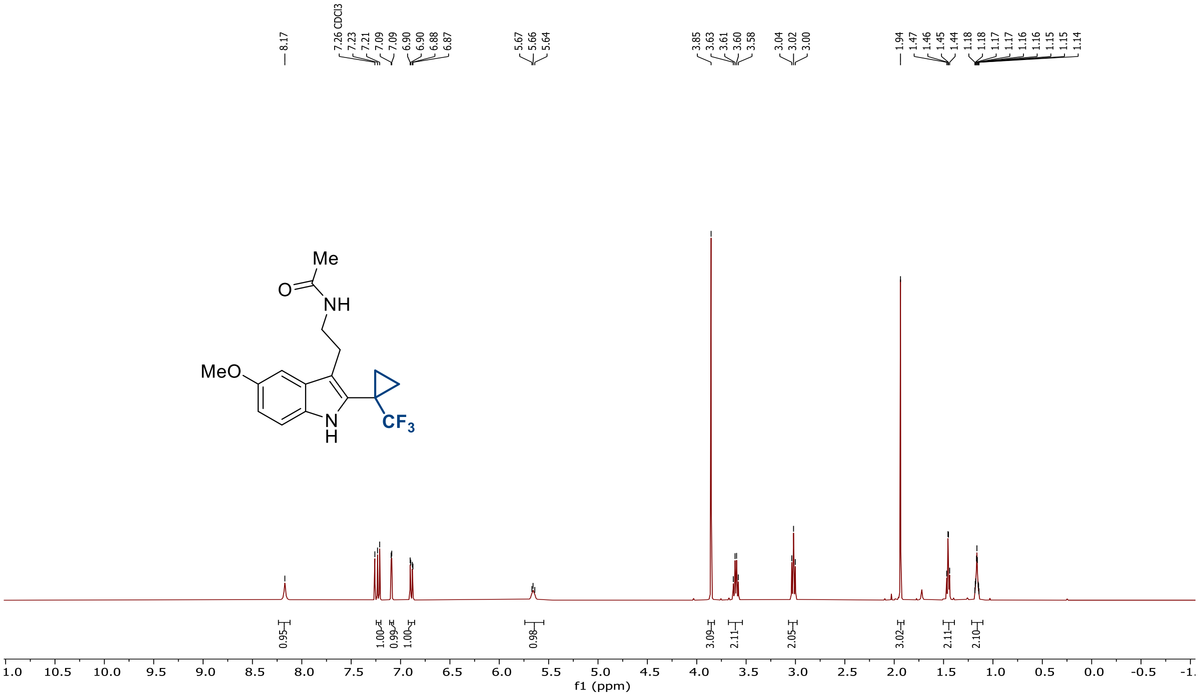


$^{13}$C{1H} NMR (101 MHz, $CDCl_3$)

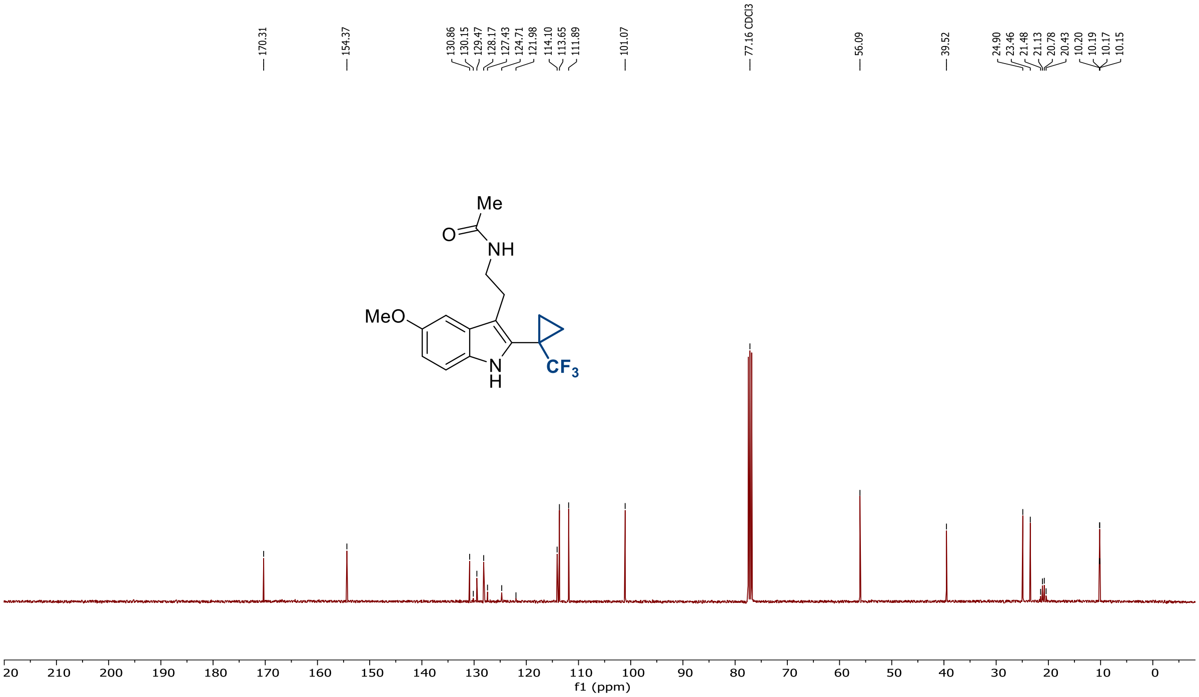

$^{19}$F NMR (377 MHz, $CDCl_3$)

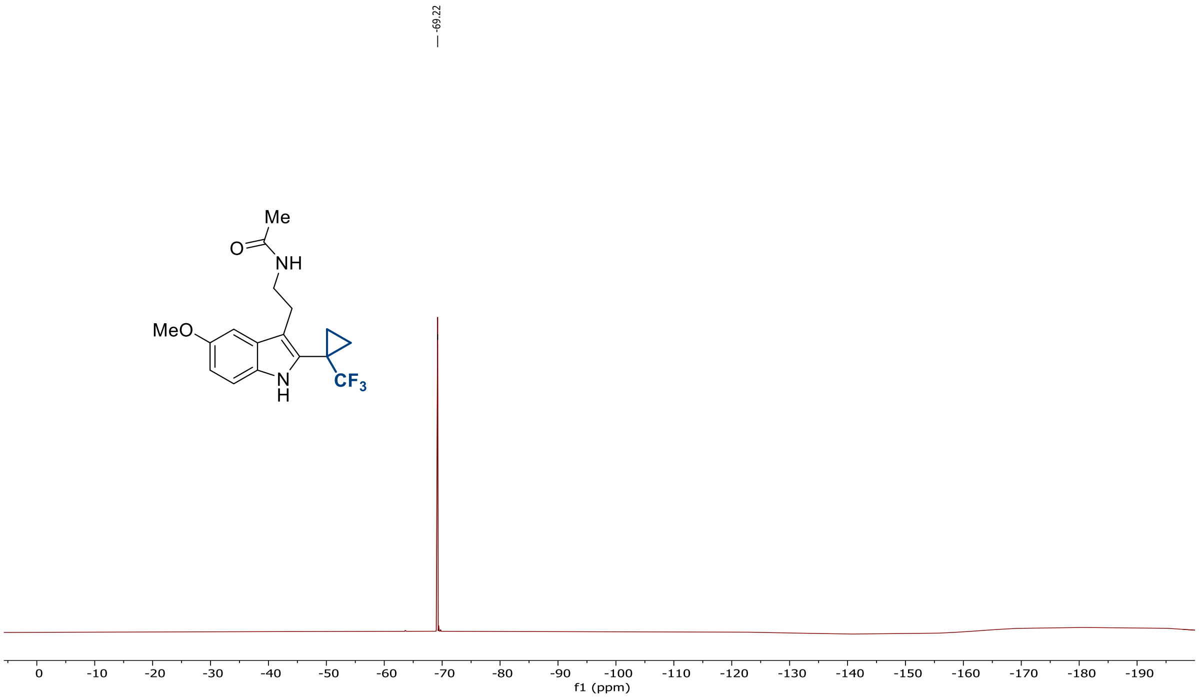


Compound **6t**:

$^{1}$H NMR (400 MHz, $CDCl_3$)

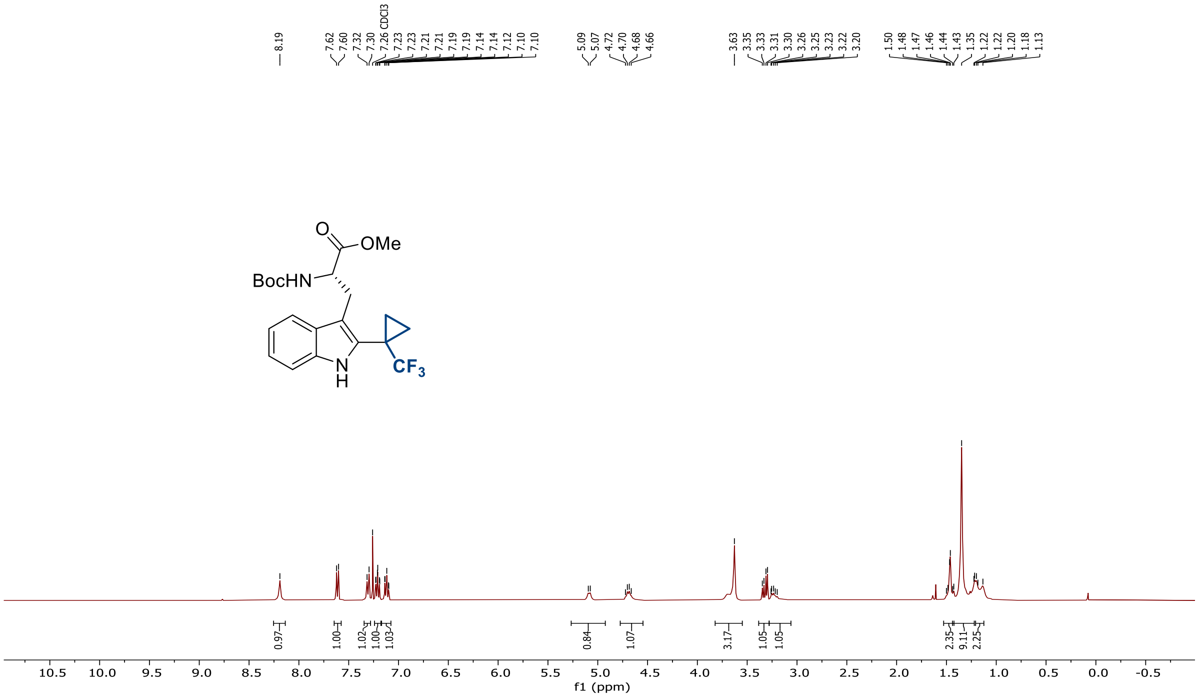

$^{13}C\{1H\}$ NMR (101 MHz, $CDCl_3$)

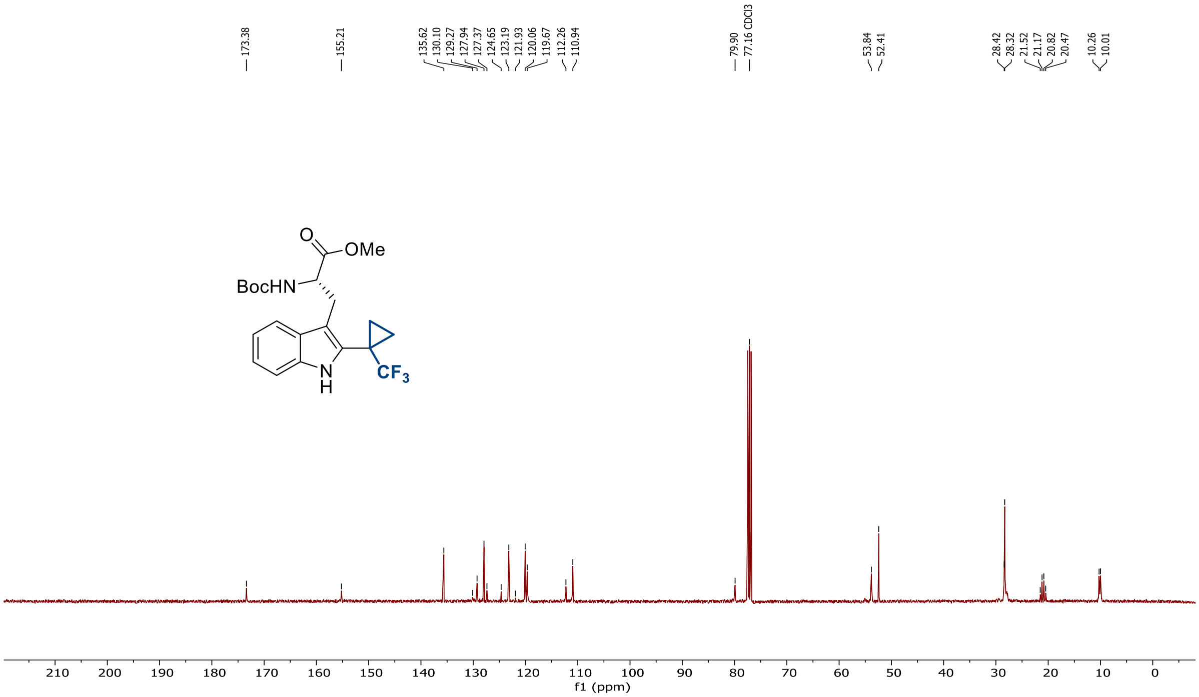


$^{19}F$ NMR (377 MHz, $CDCl_3$)

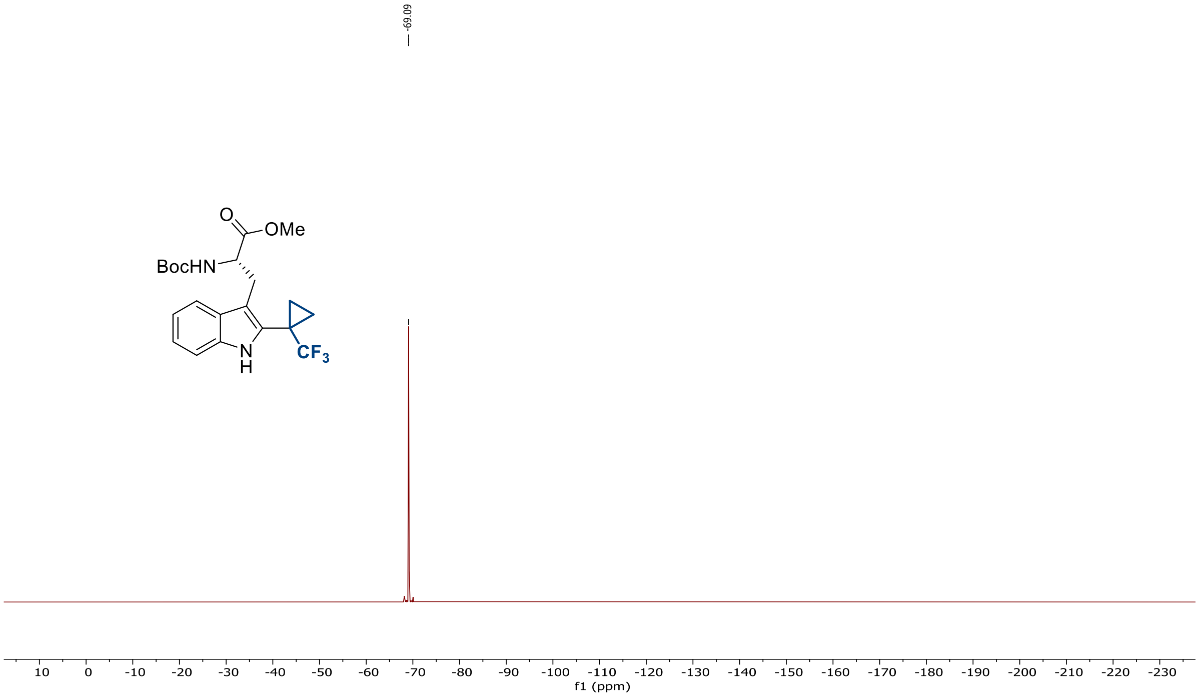

Compound **6u**:

$^{1}H$ NMR (400 MHz, $CDCl_3$)

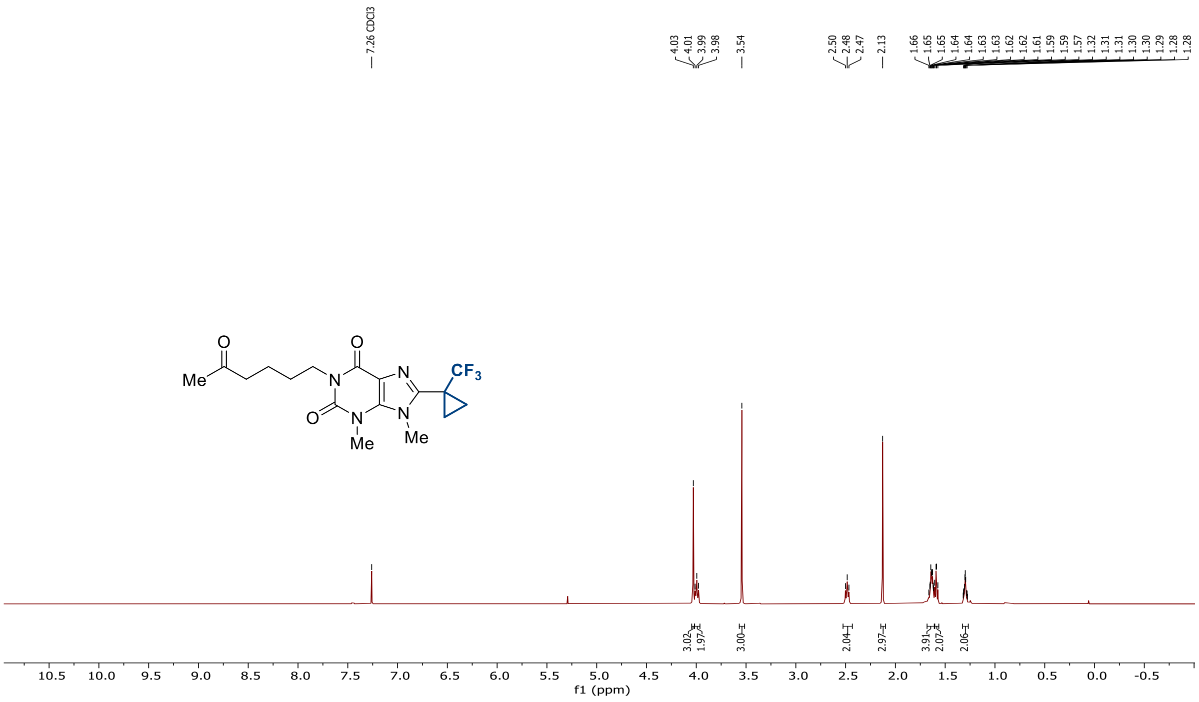


$^{13}C\{1H\}$ NMR (101 MHz, $CDCl_3$)

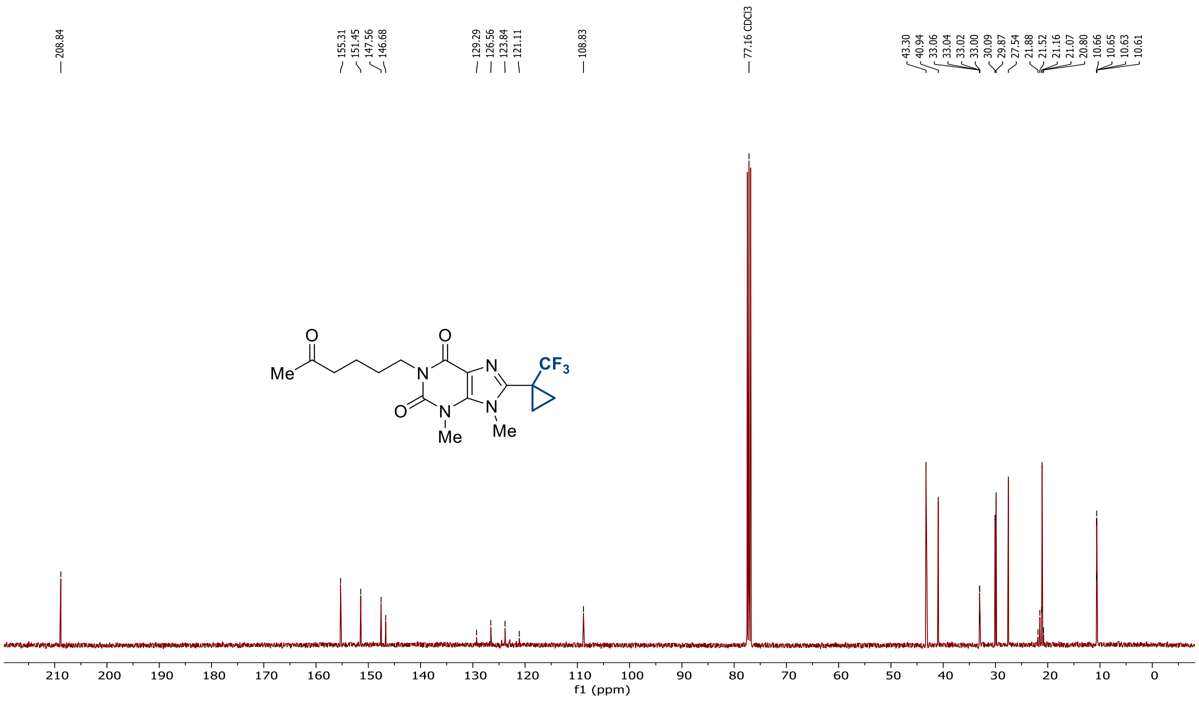

$^{19}$F NMR (377 MHz, $CDCl_3$)

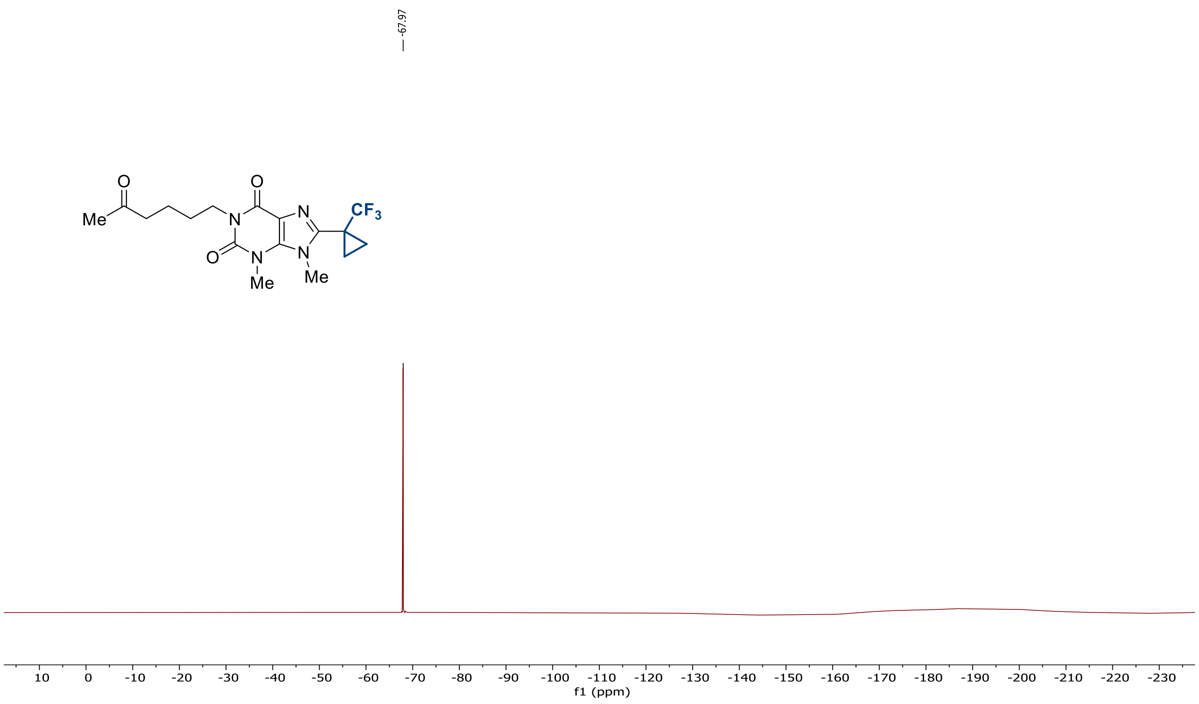


Compound **6v**:

$^{1}$H NMR (400 MHz, $CDCl_3$)

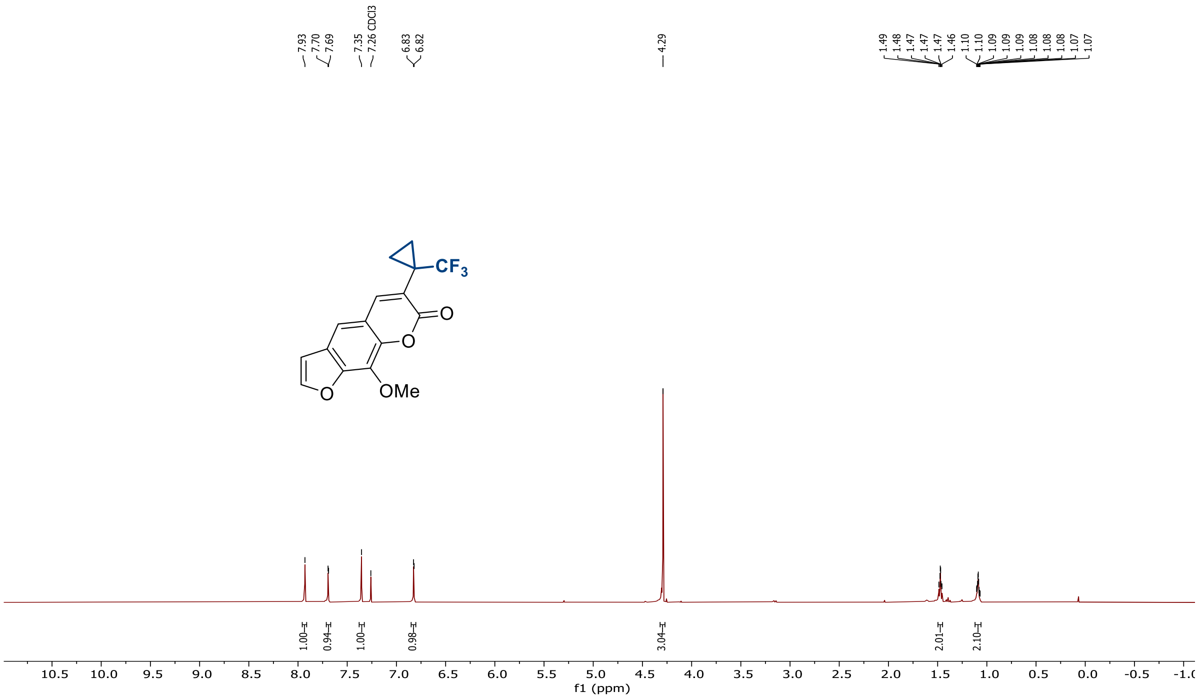

$^{13}C\{1H\}$ NMR (101 MHz, $CDCl_3$)

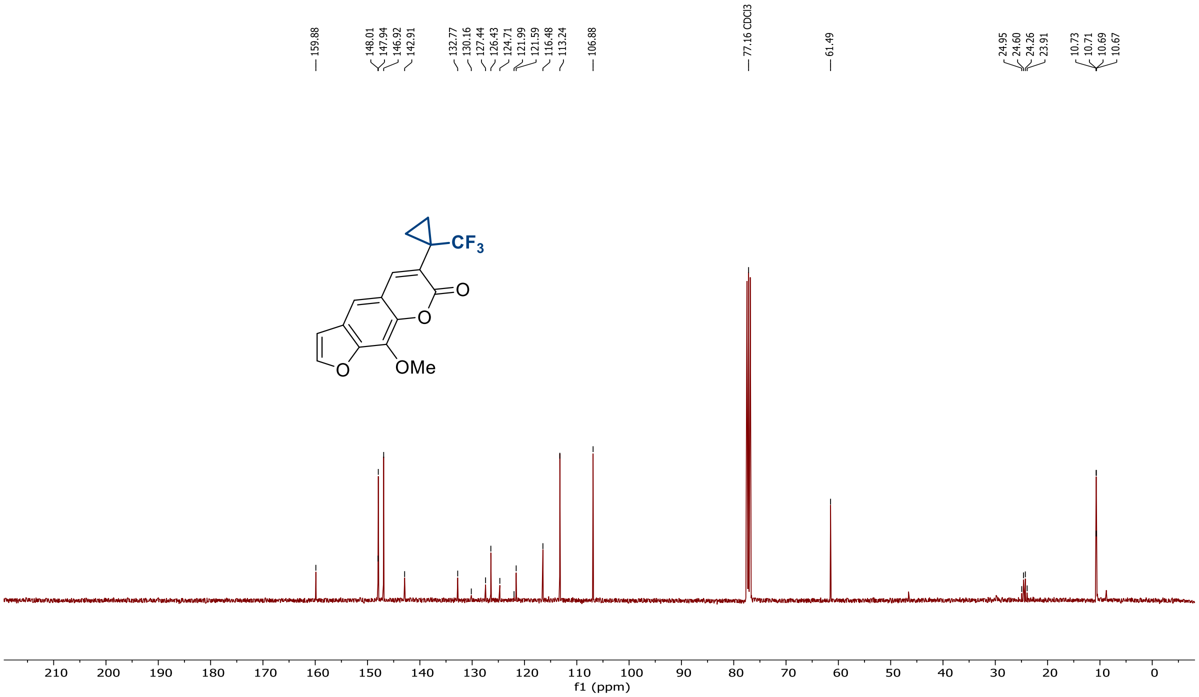


$^{19}F$ NMR (377 MHz, $CDCl_3$)

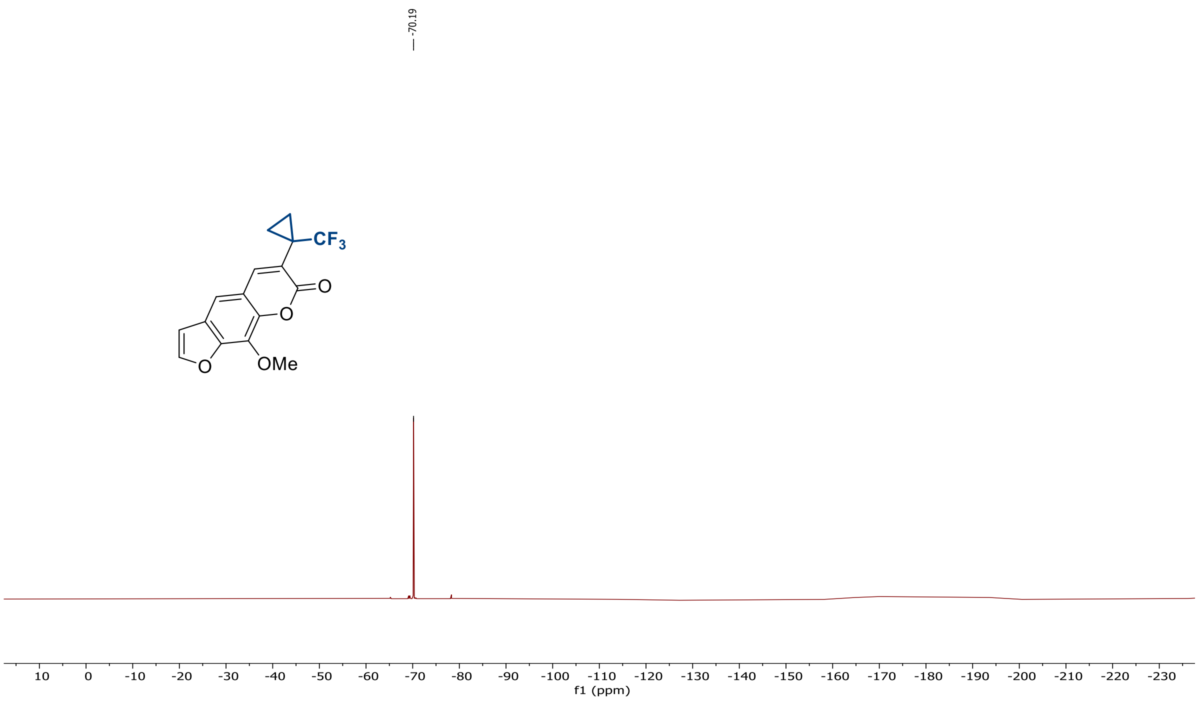

Compound **6v'**:

$^1$H NMR (400 MHz, $CDCl_3$)

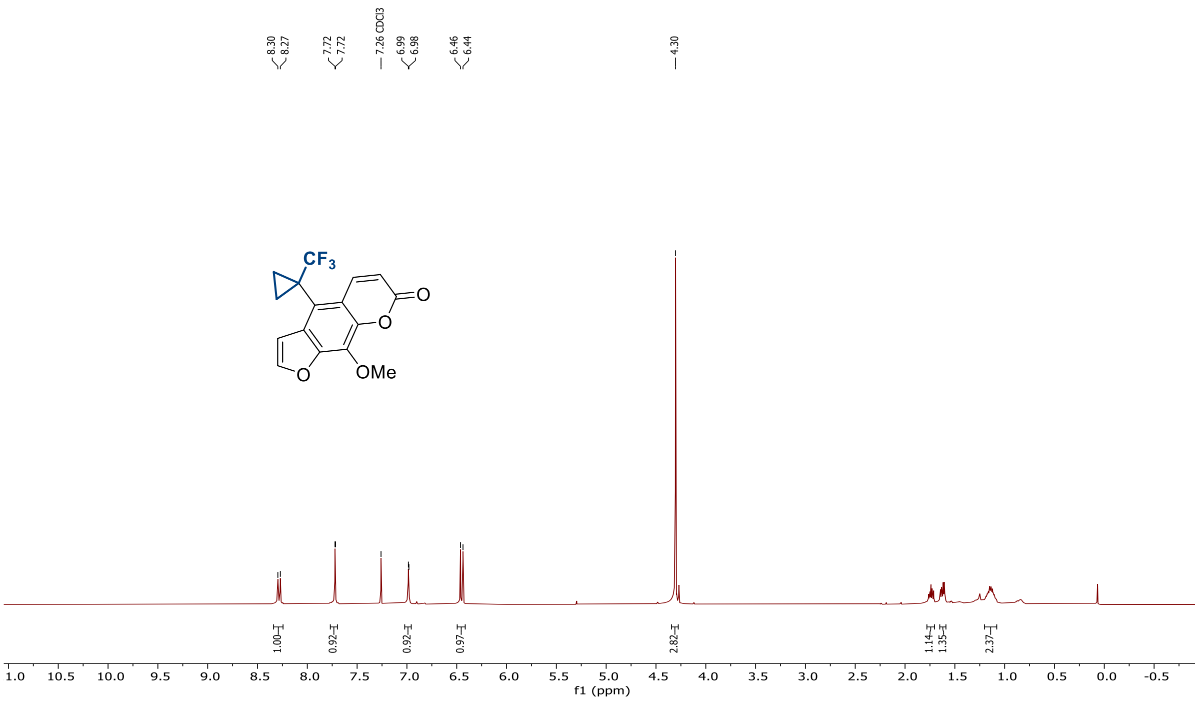


$^{13}$C{1H} NMR (101 MHz, $CDCl_3$)

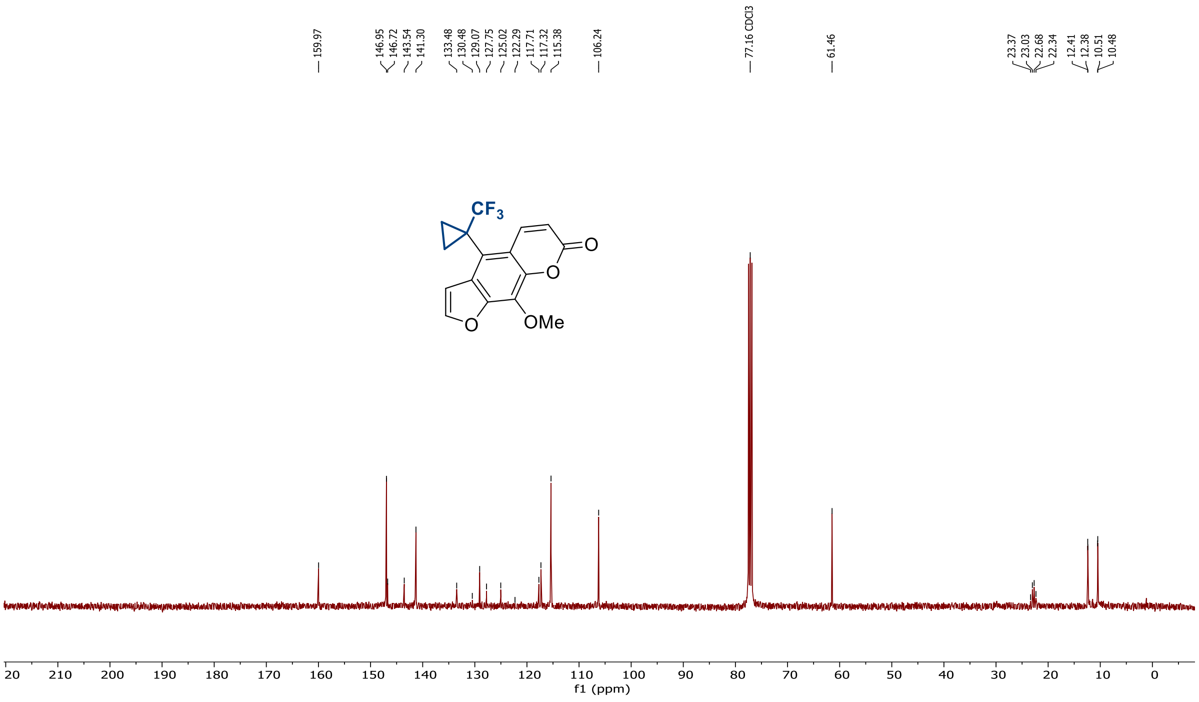

$^{19}F$ NMR (377 MHz, $CDCl_3$)

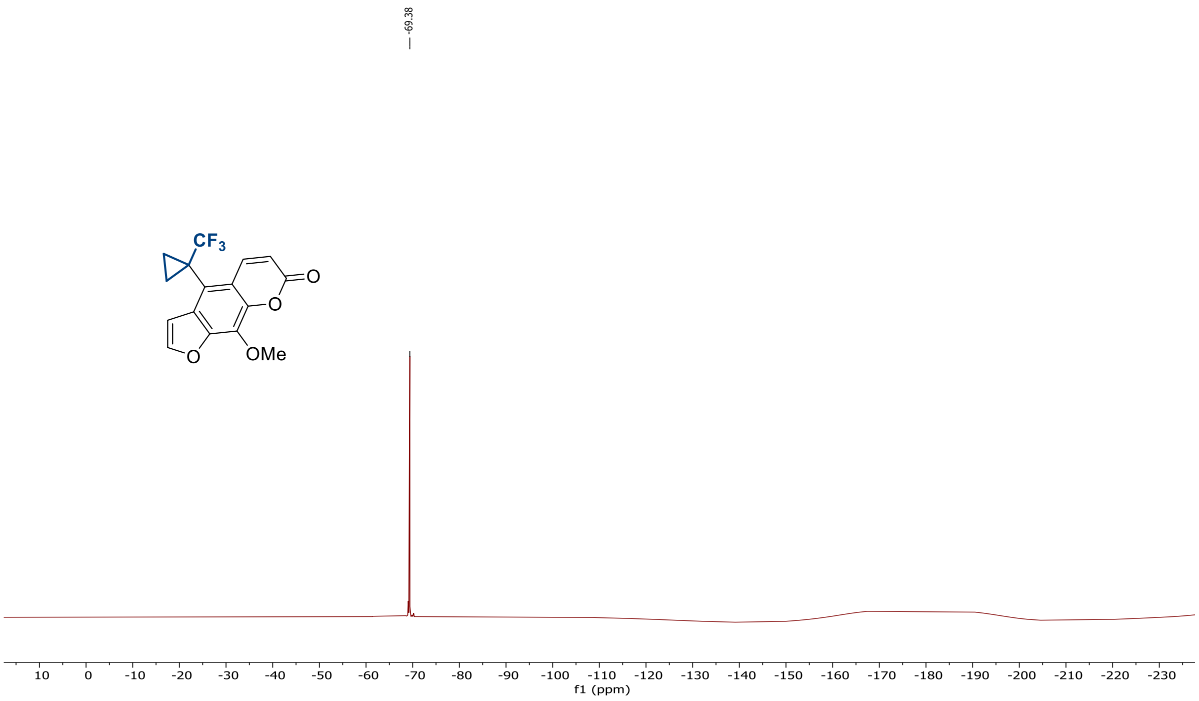


Compound **6v''**:

$^{1}H$ NMR (400 MHz, $CDCl_3$)

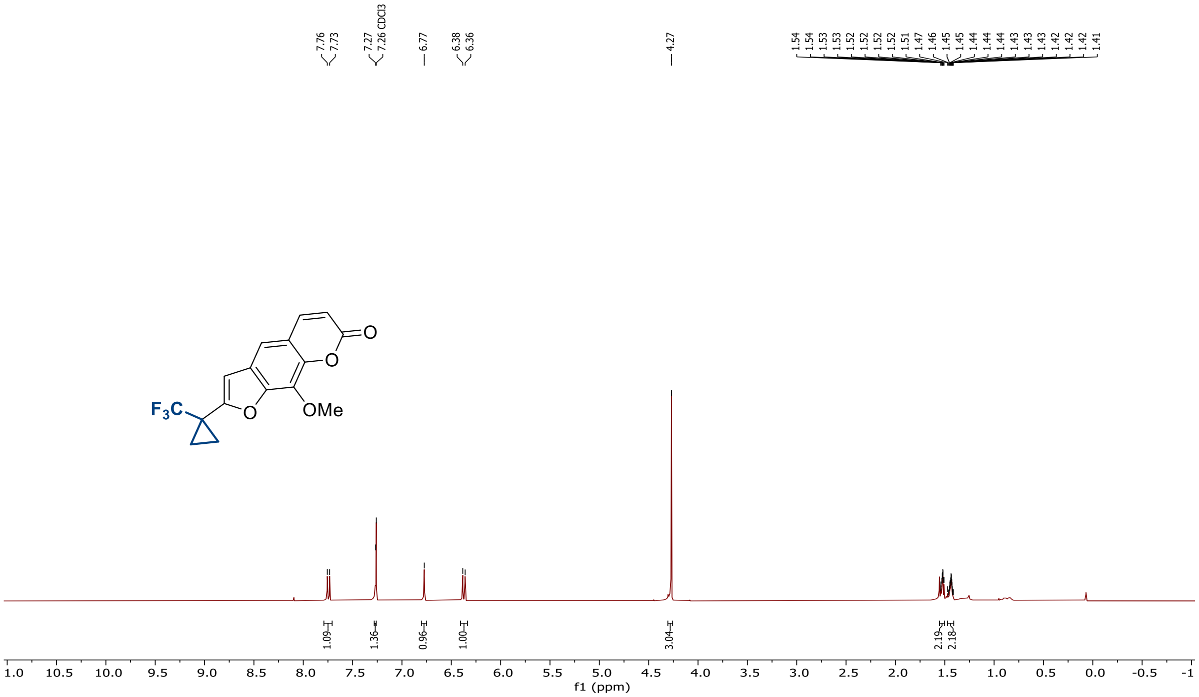

$^{13}C\{1H\}$ NMR (101 MHz, $CDCl_3$)

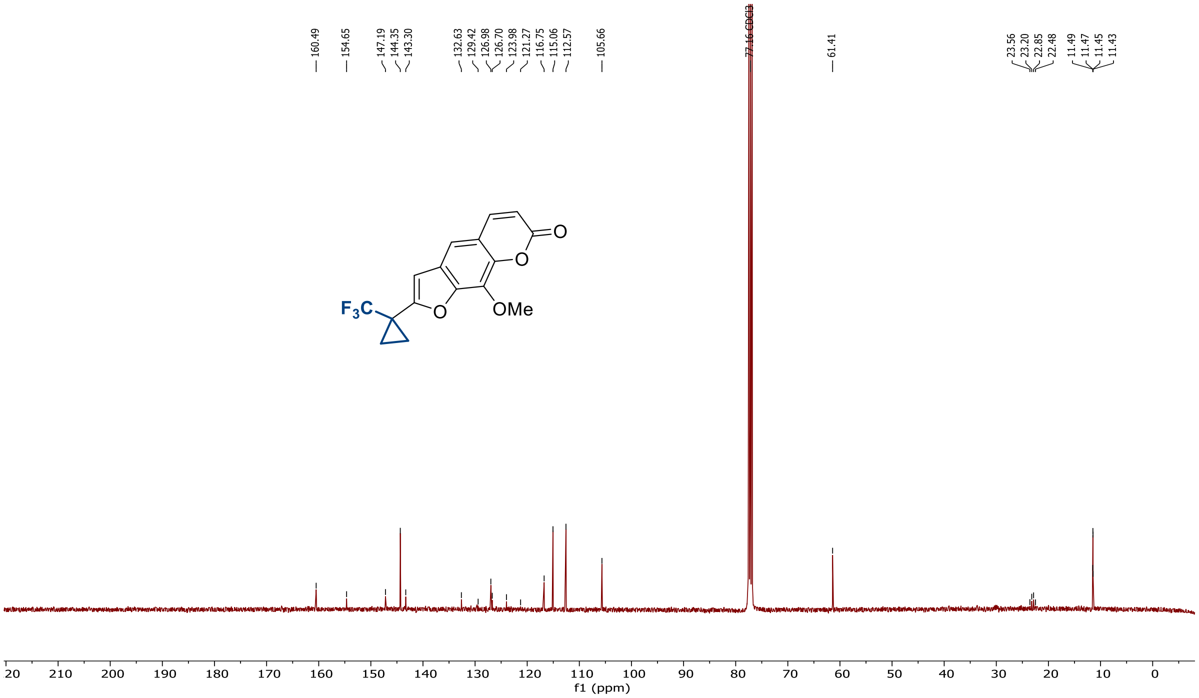


$^{19}F$ NMR (377 MHz, $CDCl_3$)

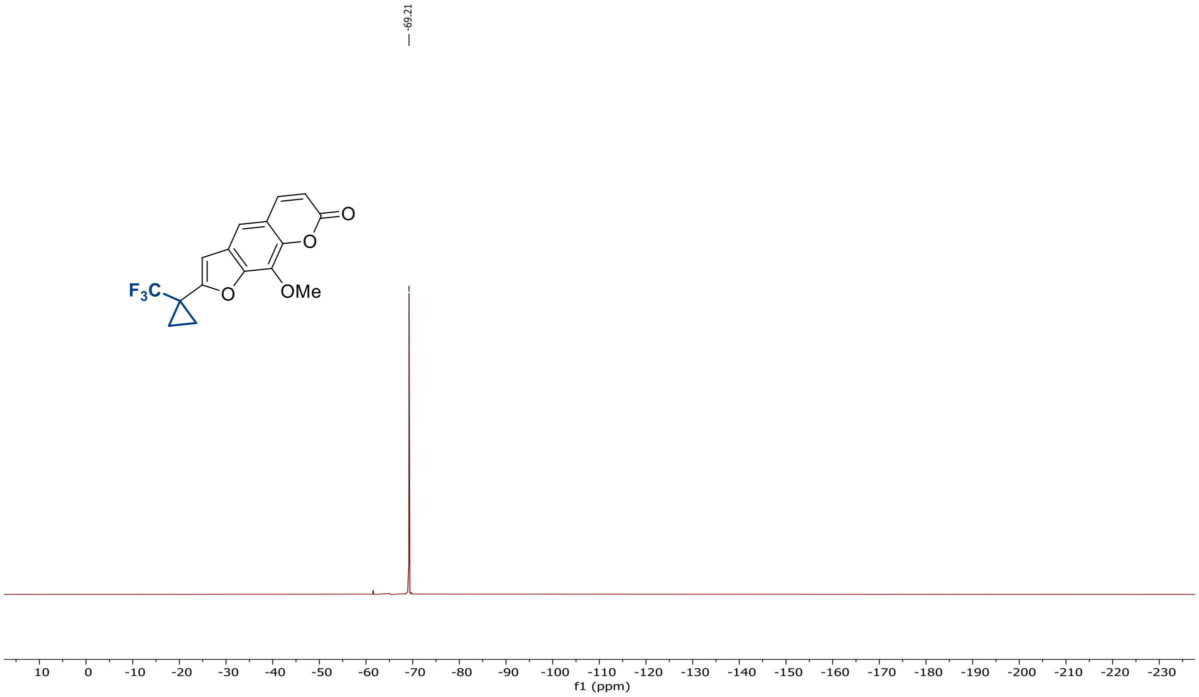

Compound **6w**:

$^{1}H$ NMR (400 MHz, $CDCl_3$)

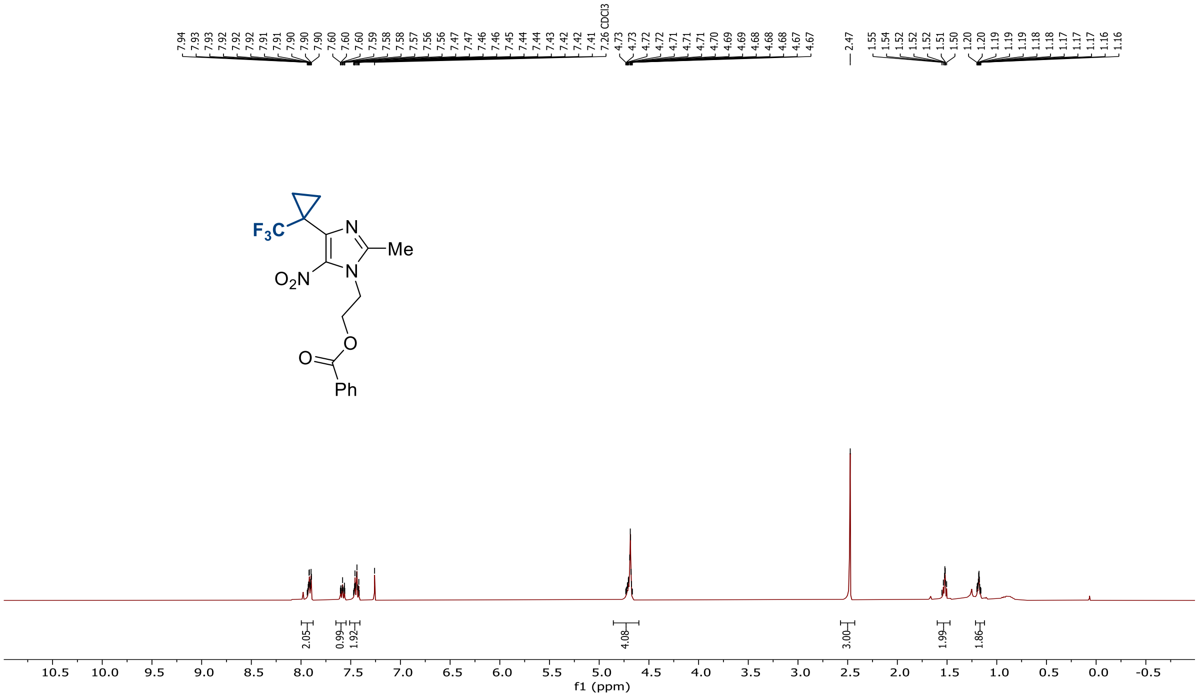


$^{13}C\{1H\}$ NMR (101 MHz, $CDCl_3$)

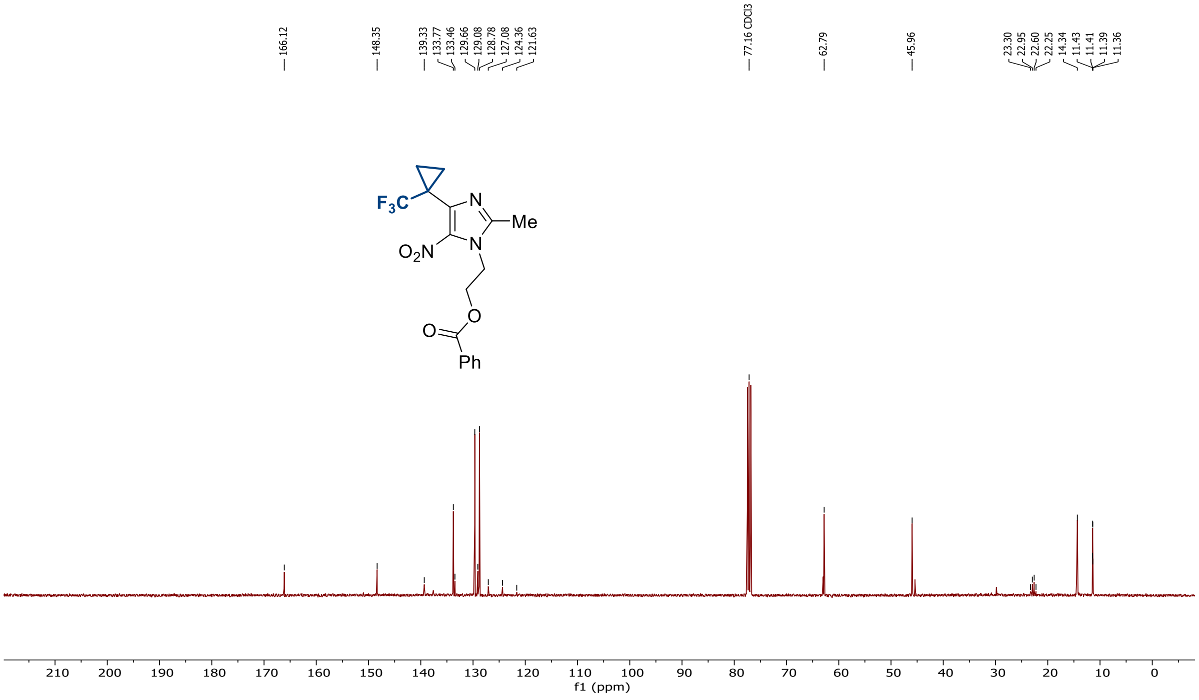

$^{19}F$ NMR (377 MHz, $CDCl_3$)

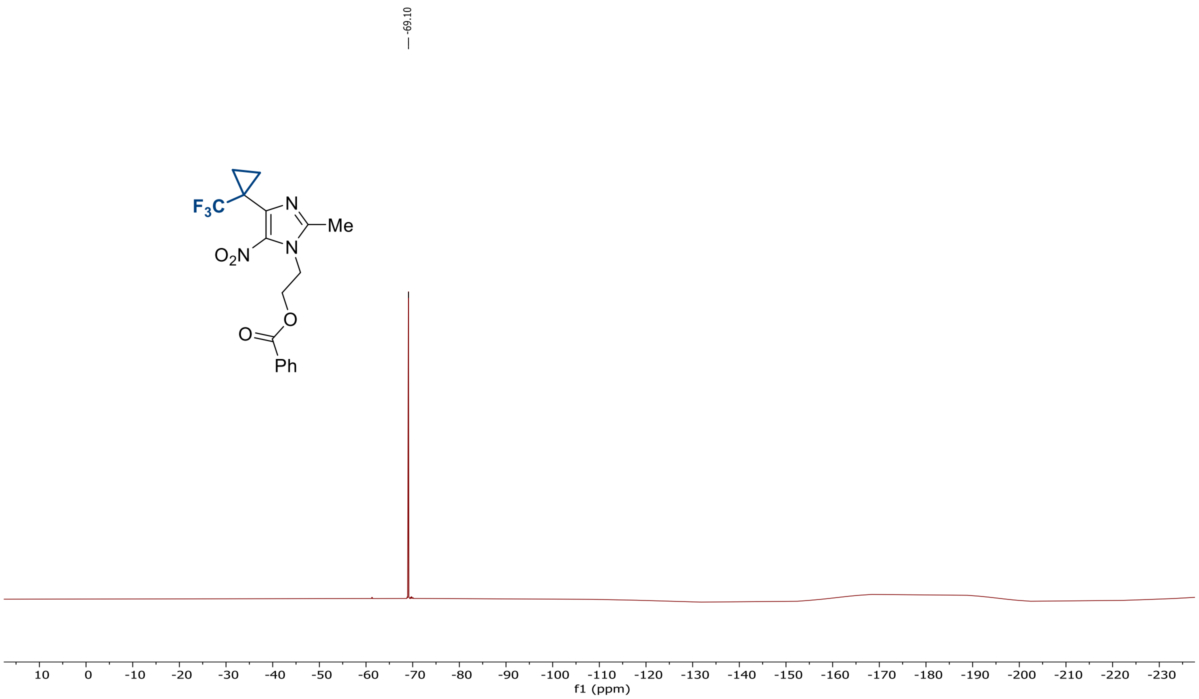


Compound **6x**:

$^{1}H$ NMR (400 MHz, $CDCl_3$)

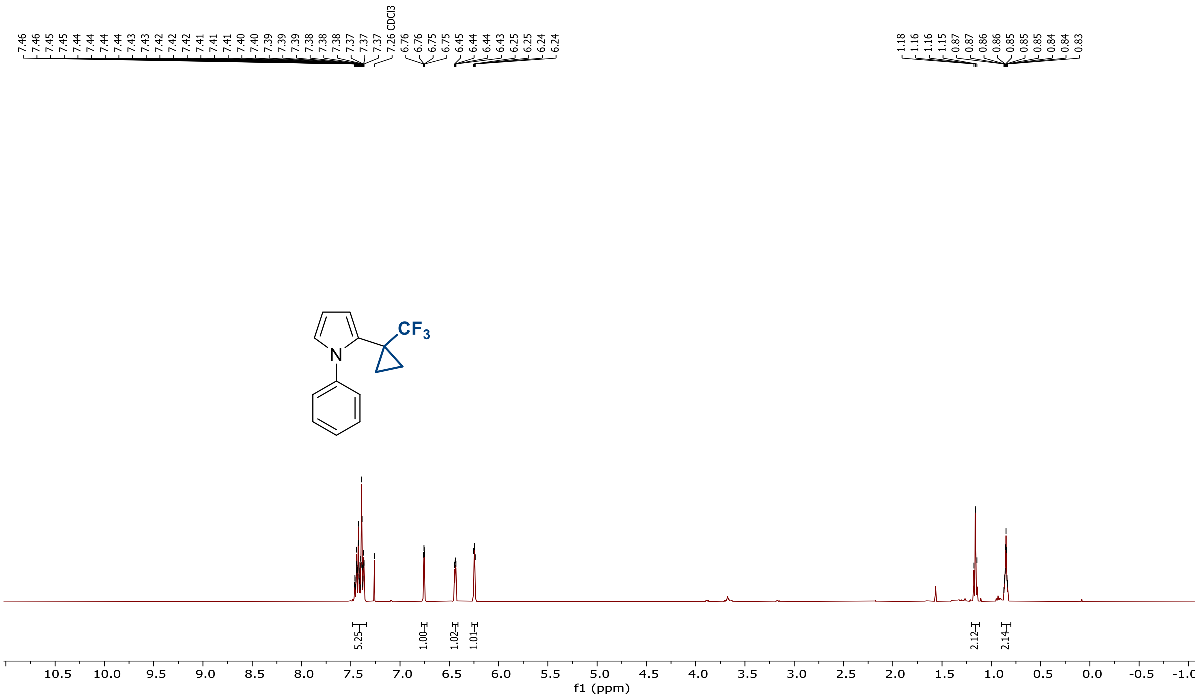

$^{13}C\{1H\}$ NMR (101 MHz, $CDCl_3$)

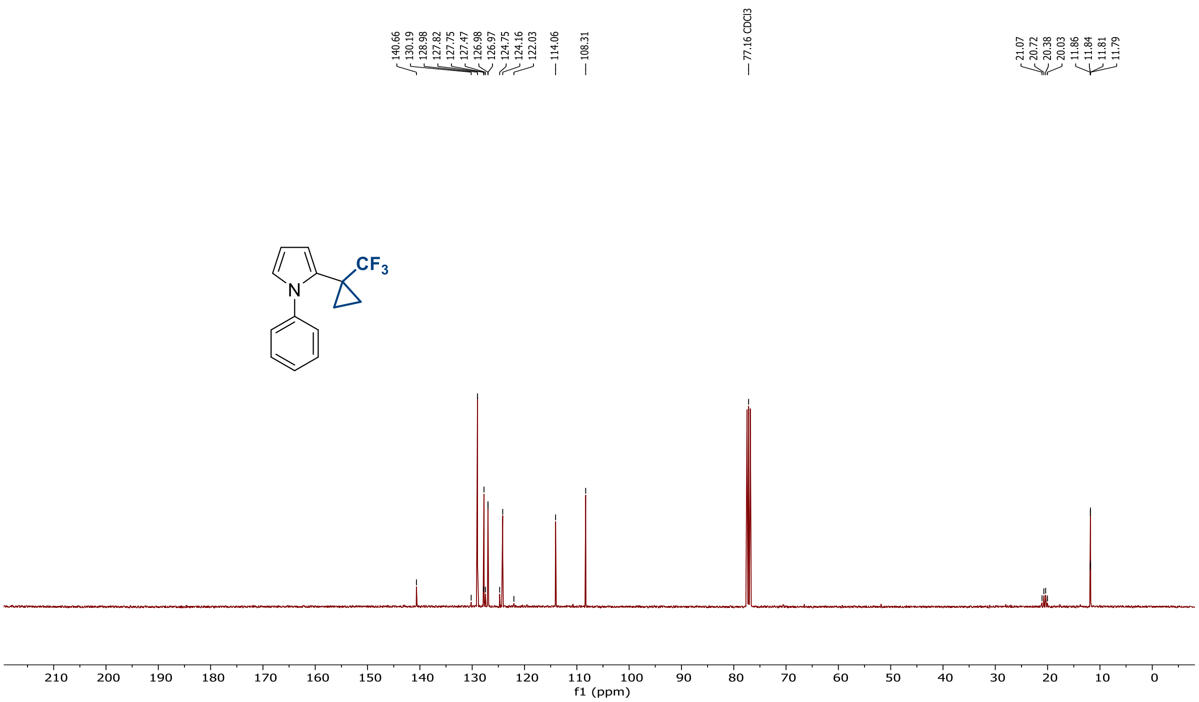


$^{19}F$ NMR (377 MHz, $CDCl_3$)

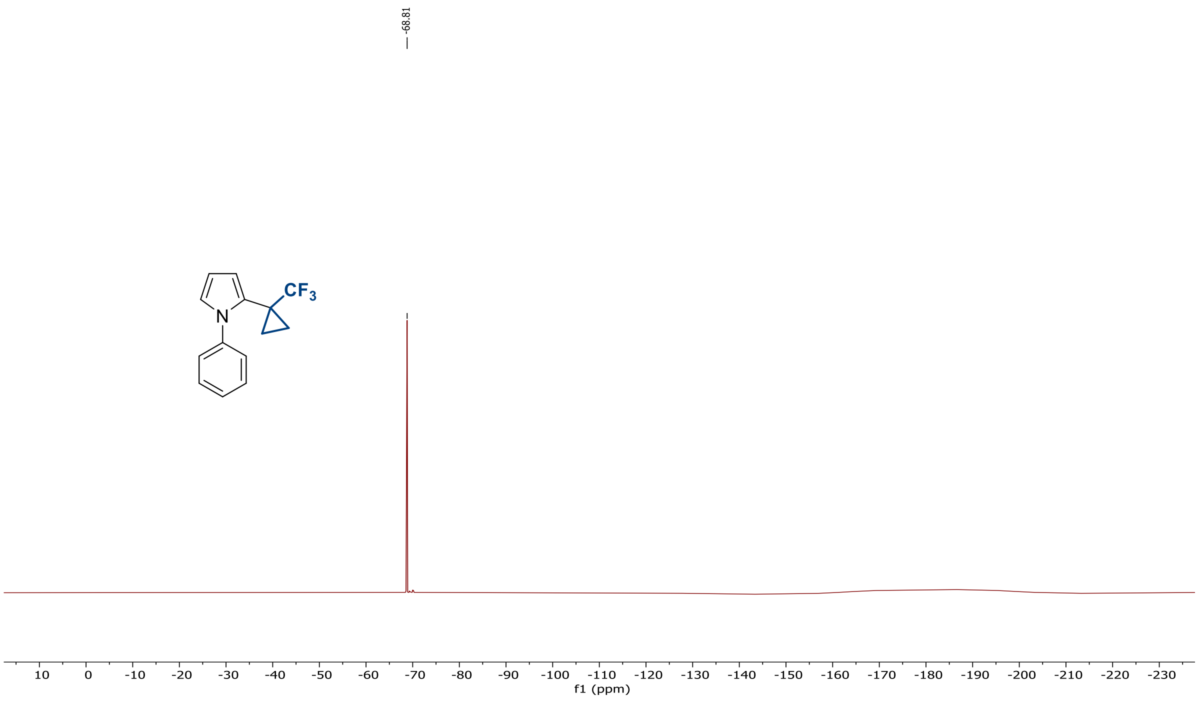

Compound **6y**:

$^{1}$H NMR (400 MHz, $CDCl_3$)

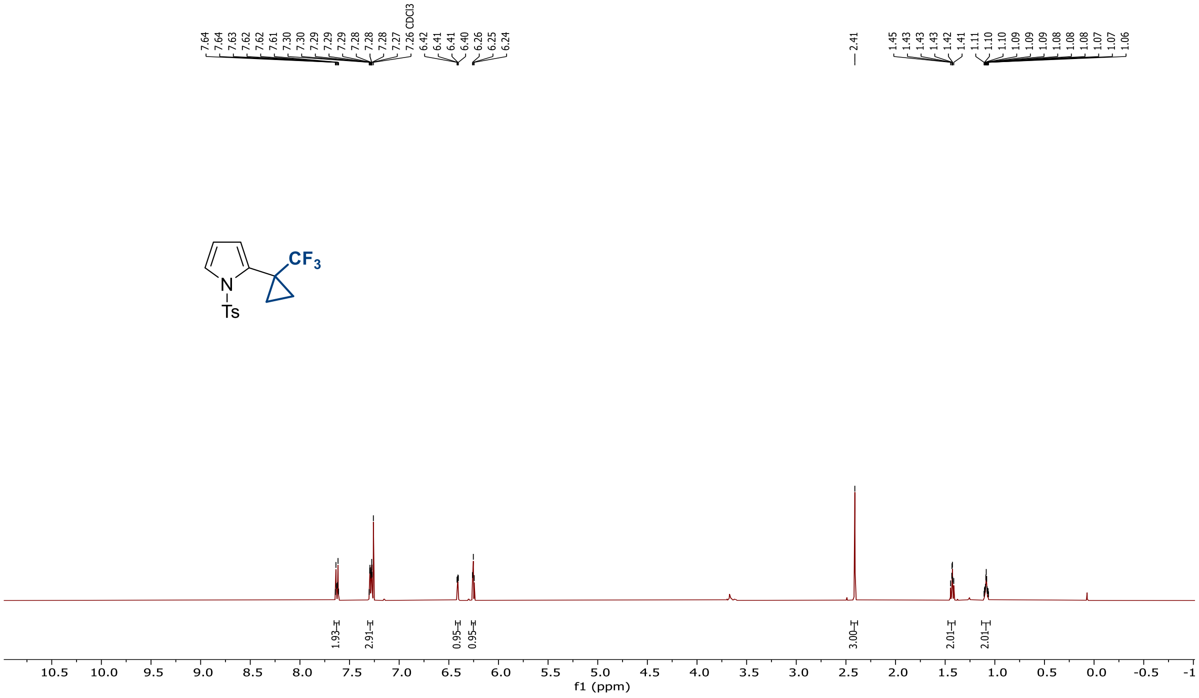


$^{13}$C{1H} NMR (101 MHz, $CDCl_3$)

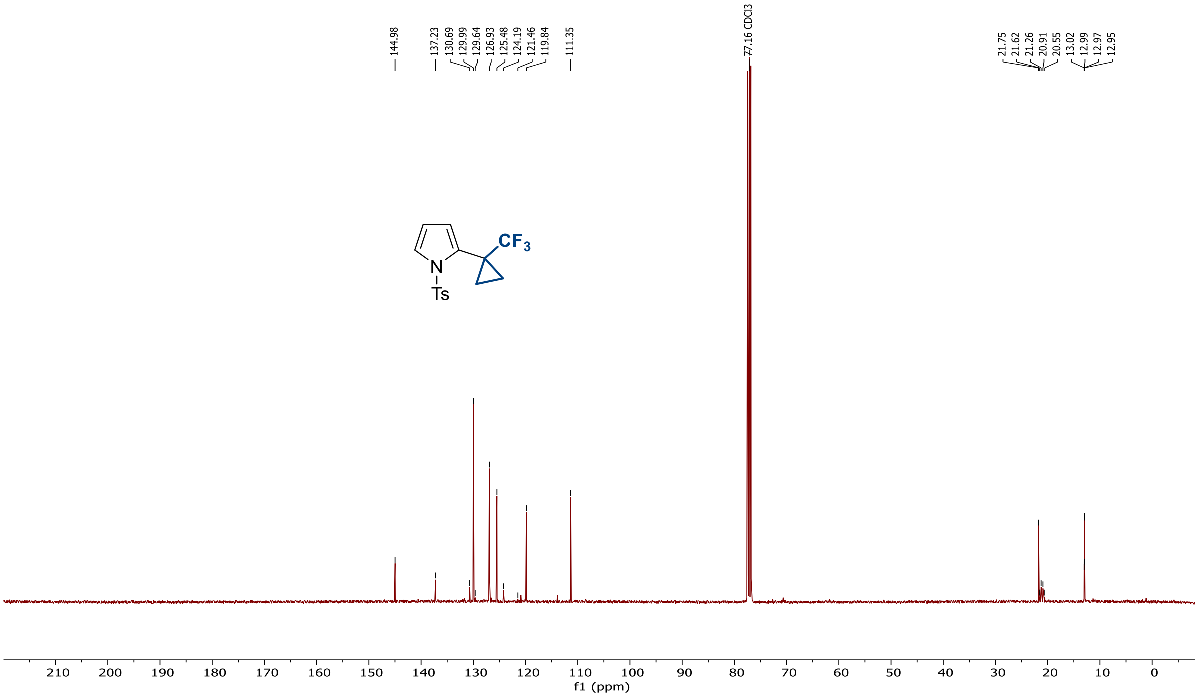

$^{19}F$ NMR (377 MHz, $CDCl_3$)

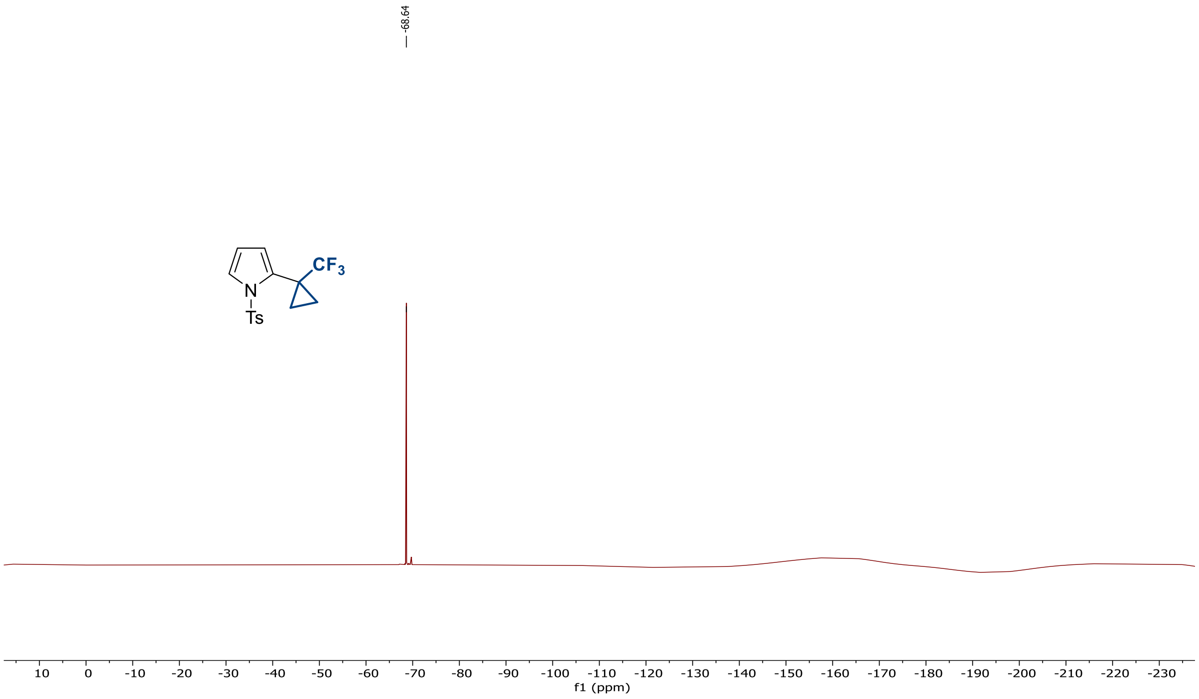


Compound **6z**:

$^{1}H$ NMR (400 MHz, $CDCl_3$)

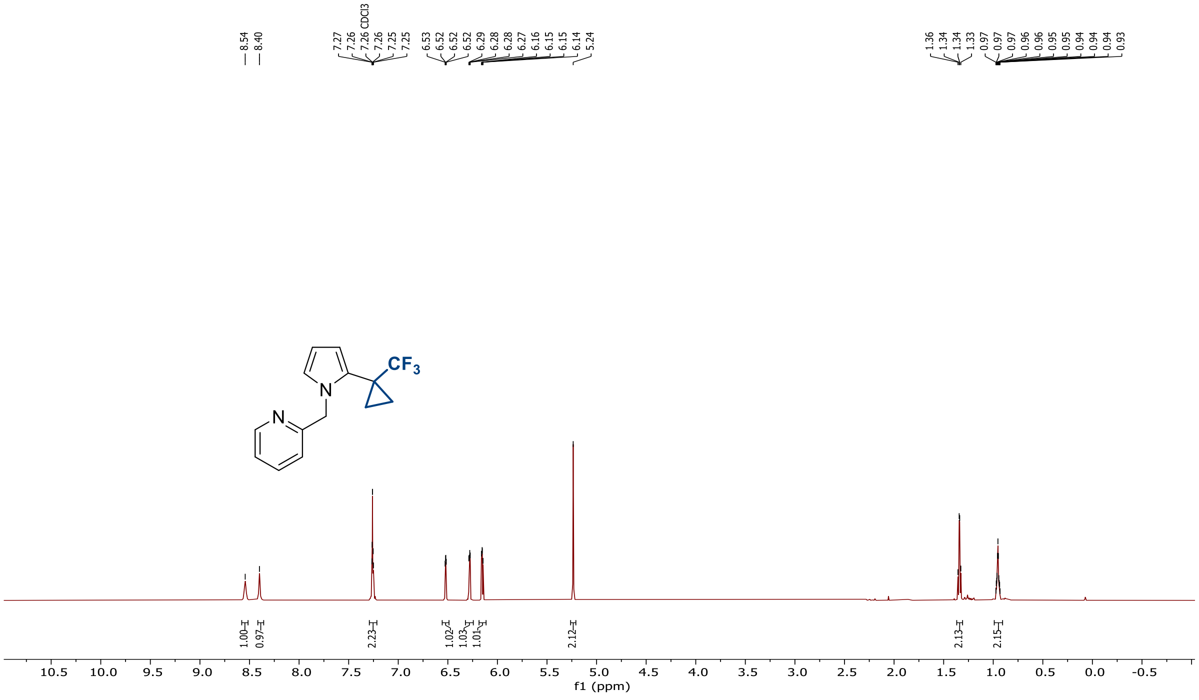

$^{13}C\{1H\}$ NMR (101 MHz, $CDCl_3$)

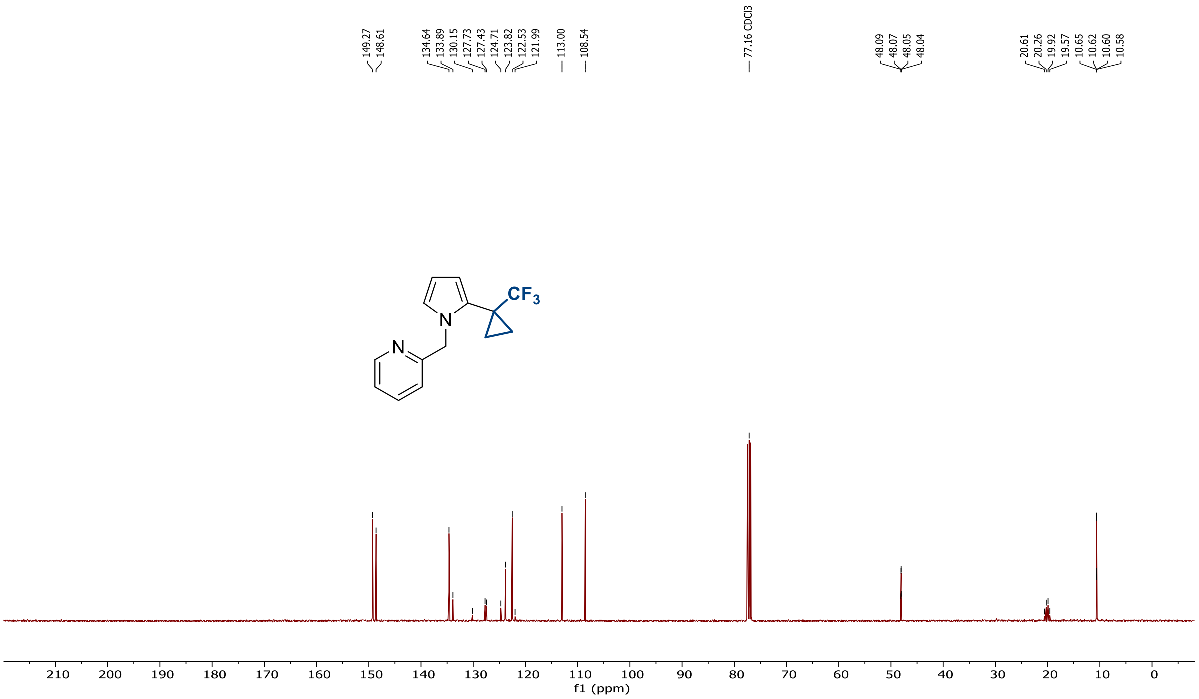


$^{19}F$ NMR (377 MHz, $CDCl_3$)

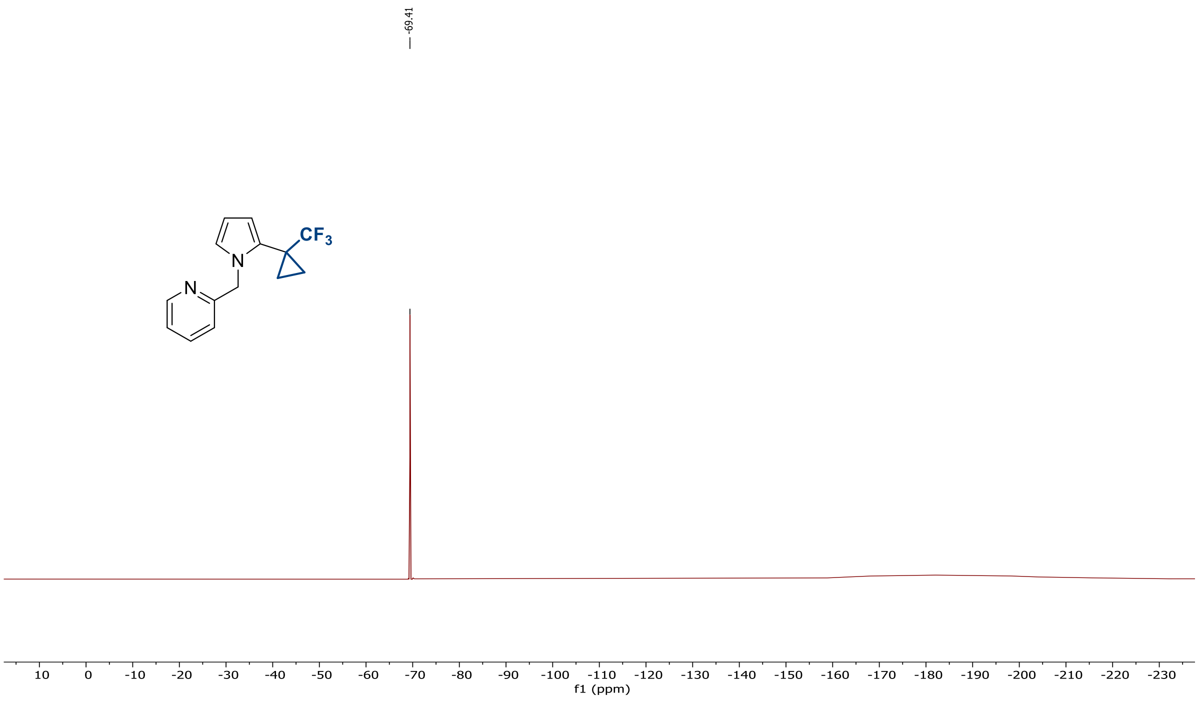

Compound **6aa**:

$^{1}H$ NMR (400 MHz, $CDCl_3$)

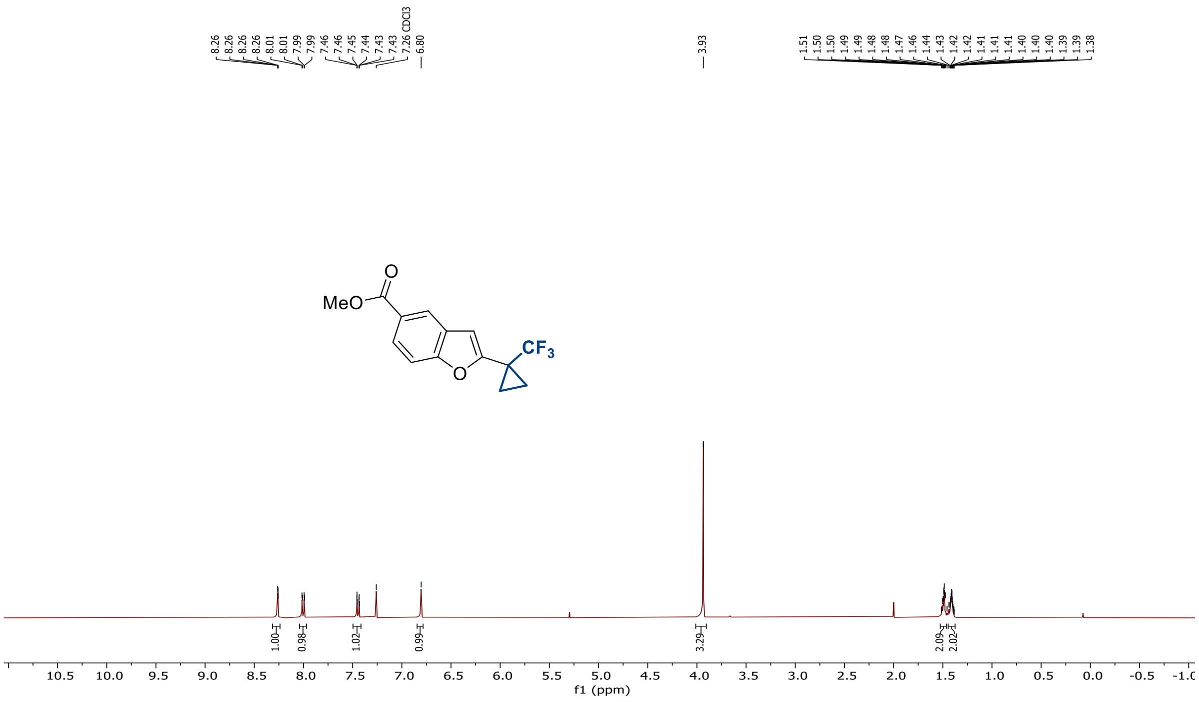


$^{13}C\{1H\}$ NMR (101 MHz, $CDCl_3$)

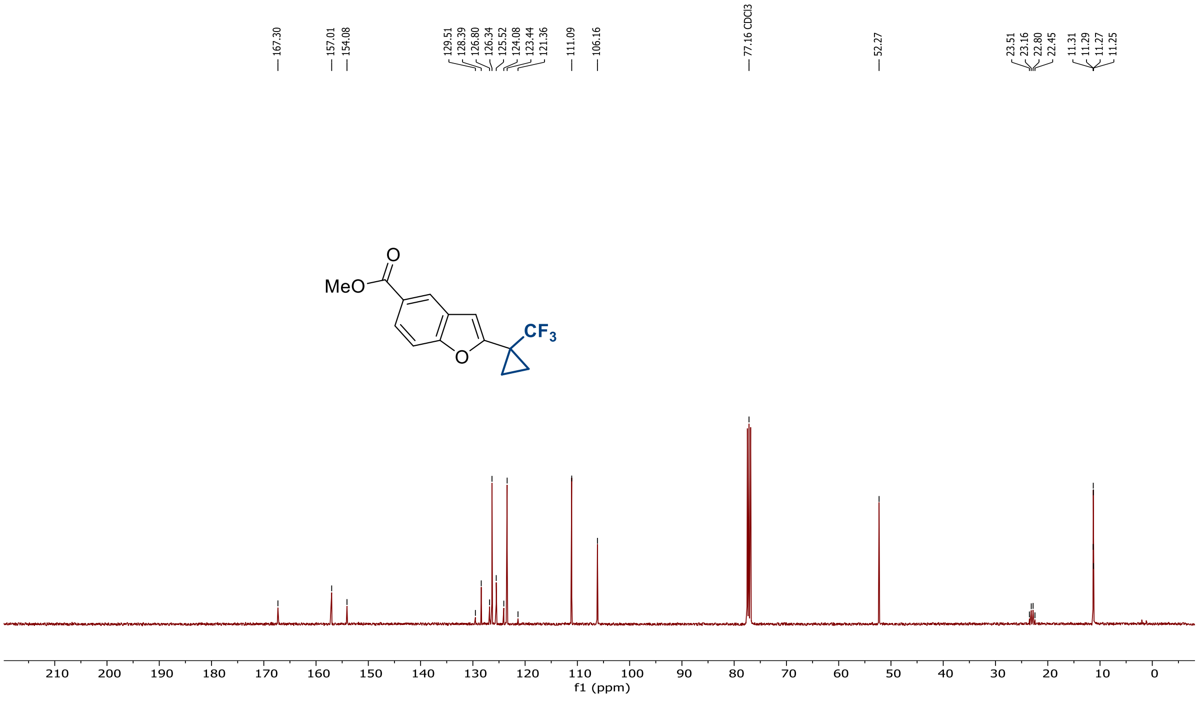

$^{19}F$ NMR (377 MHz, $CDCl_3$)

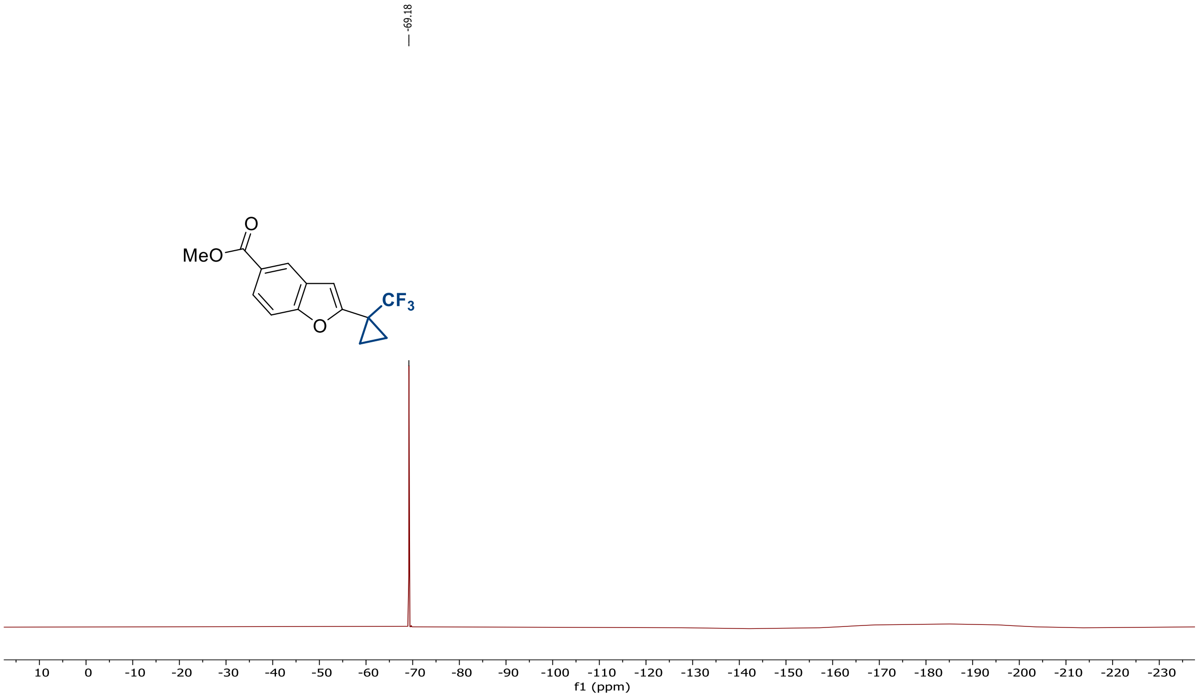


Compound **6ab**:

$^{1}H$ NMR (400 MHz, $CDCl_3$)

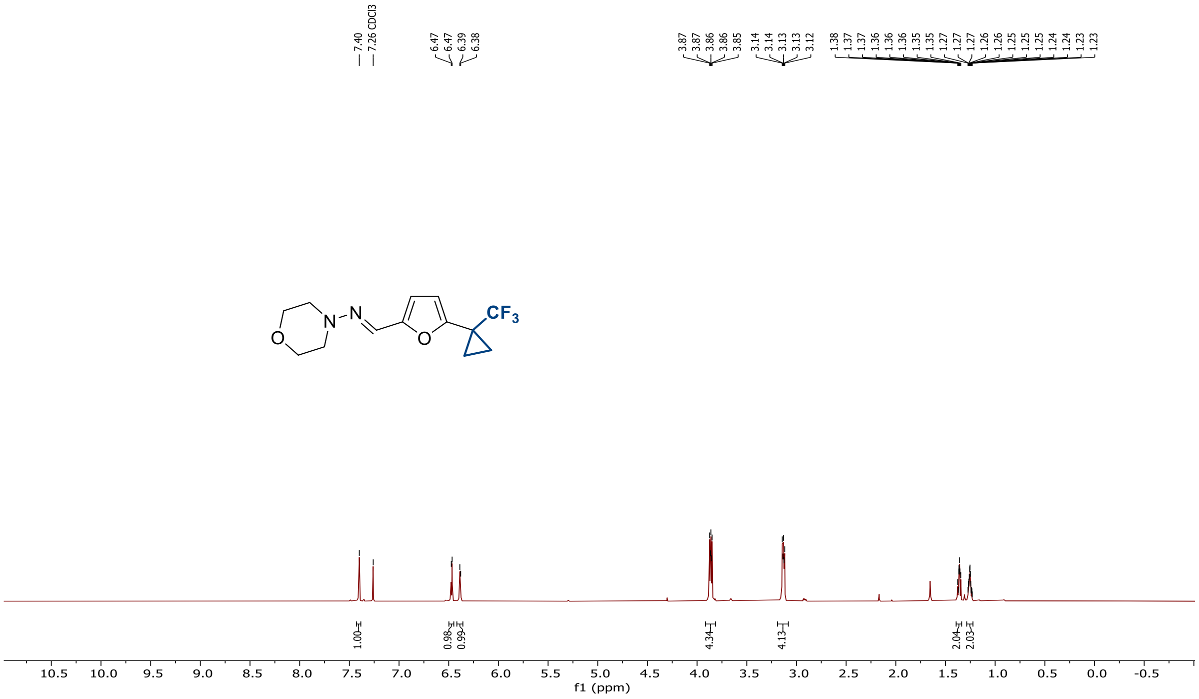

$^{13}C\{1H\}$ NMR (101 MHz, $CDCl_3$)

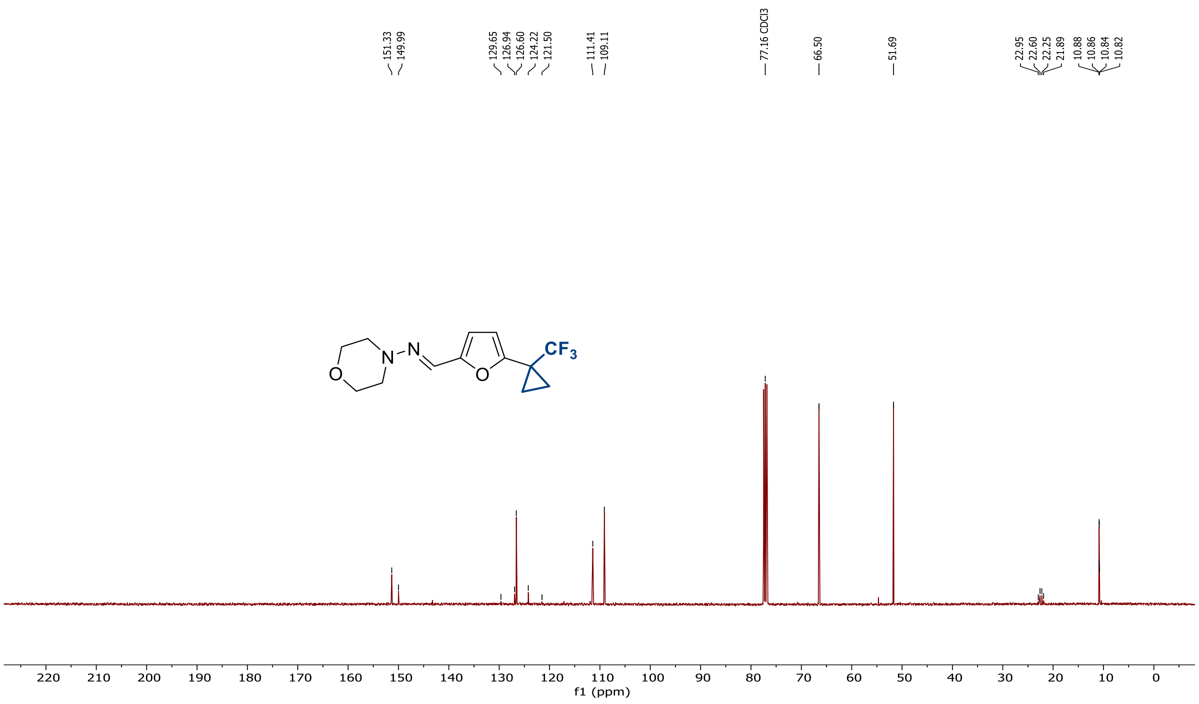


$^{19}F$ NMR (377 MHz, $CDCl_3$)

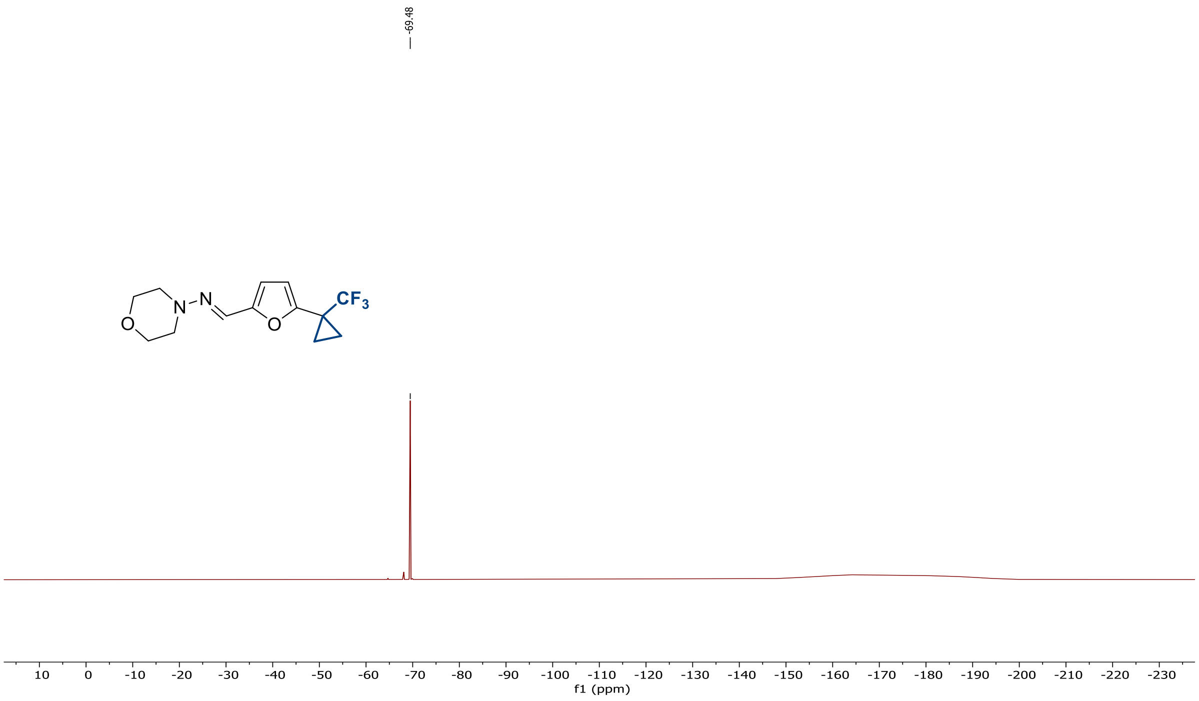

Compound **6ac**:

$^{1}H$ NMR (400 MHz, $CDCl_3$)

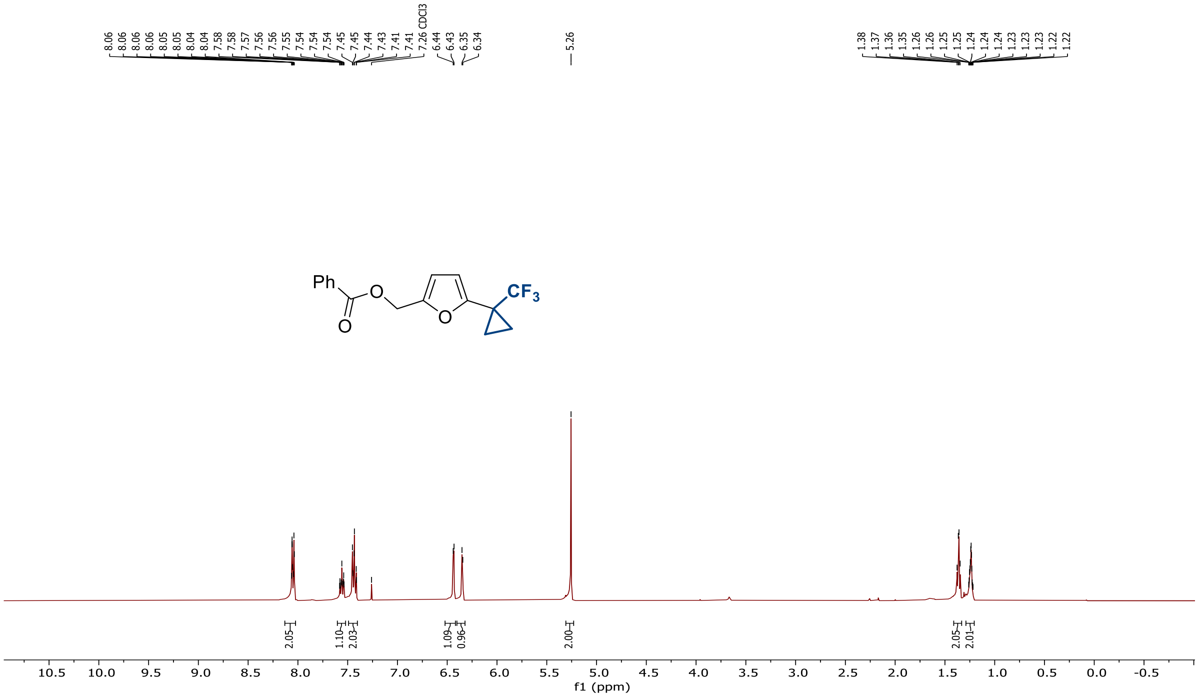


$^{13}C\{1H\}$ NMR (101 MHz, $CDCl_3$)

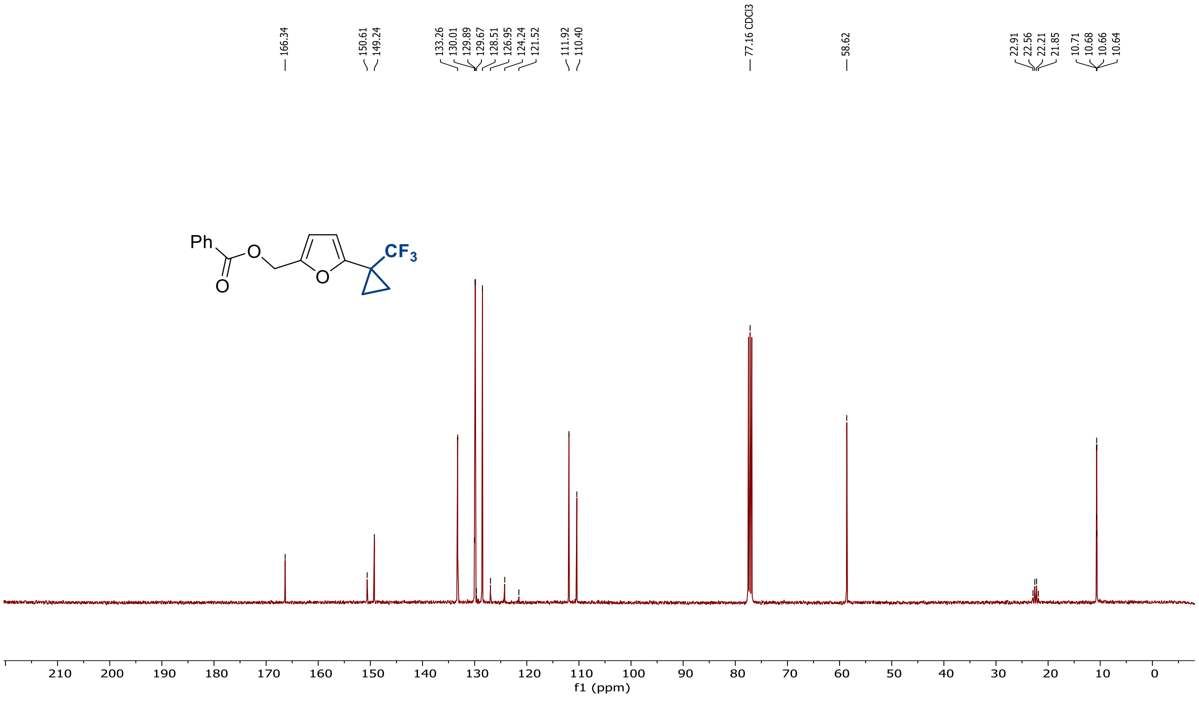

$^{19}$F NMR (377 MHz, $CDCl_3$)

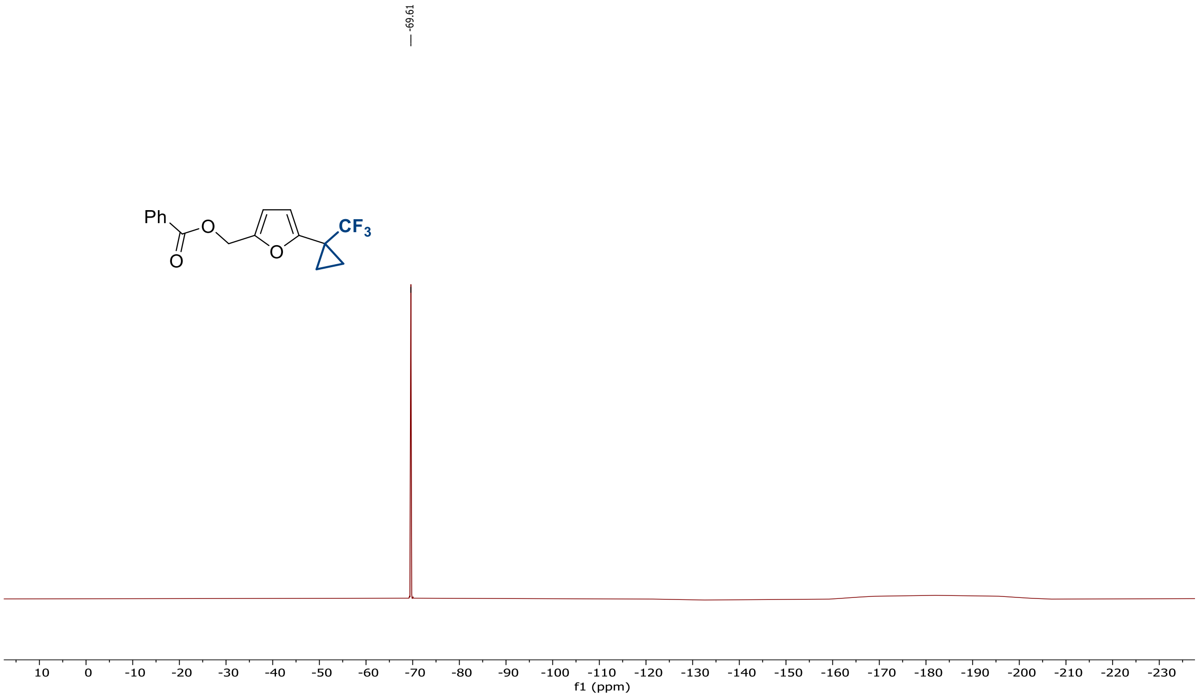


Compound **6ad**:

$^{1}$H NMR (400 MHz, $CDCl_3$)

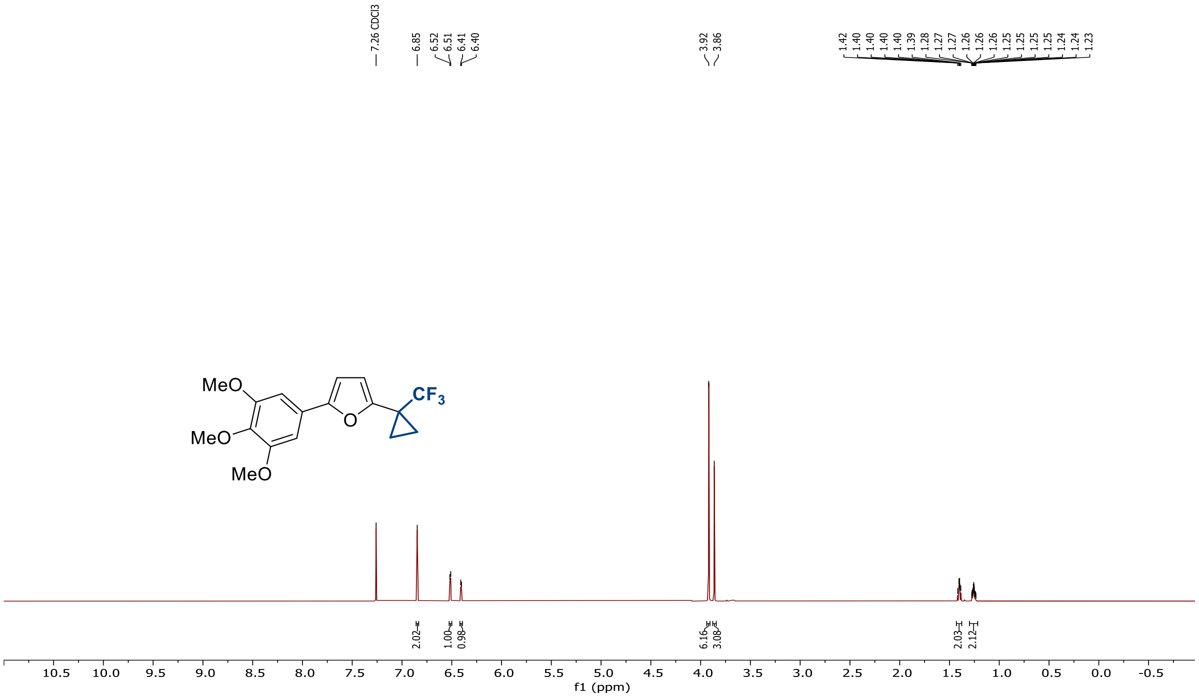

$^{13}C\{1H\}$ NMR (101 MHz, $CDCl_3$)

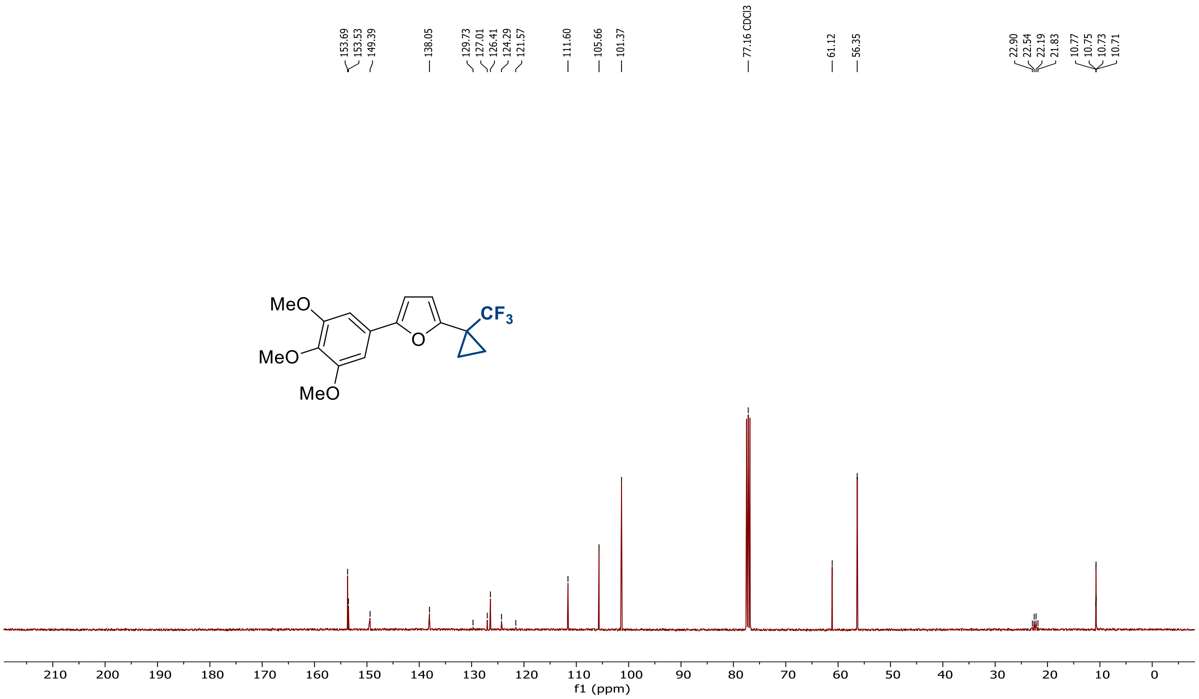


$^{19}F$ NMR (377 MHz, $CDCl_3$)

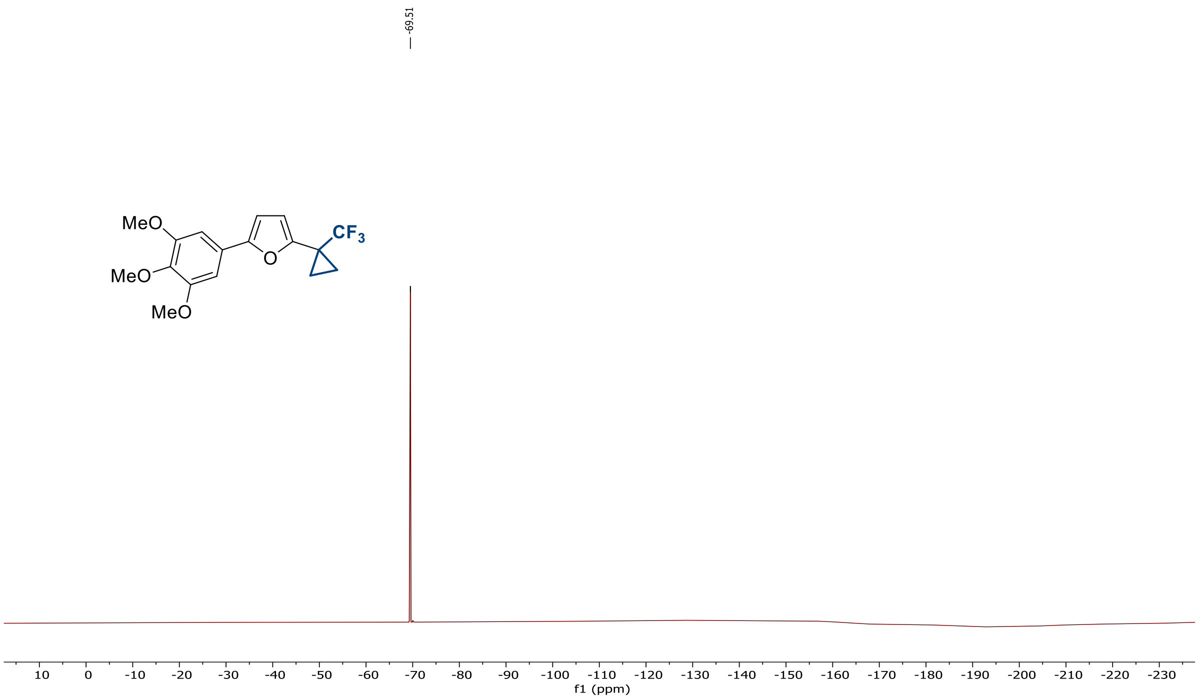

Compound **6ae**:

$^1$H NMR (300 MHz, $CDCl_3$)

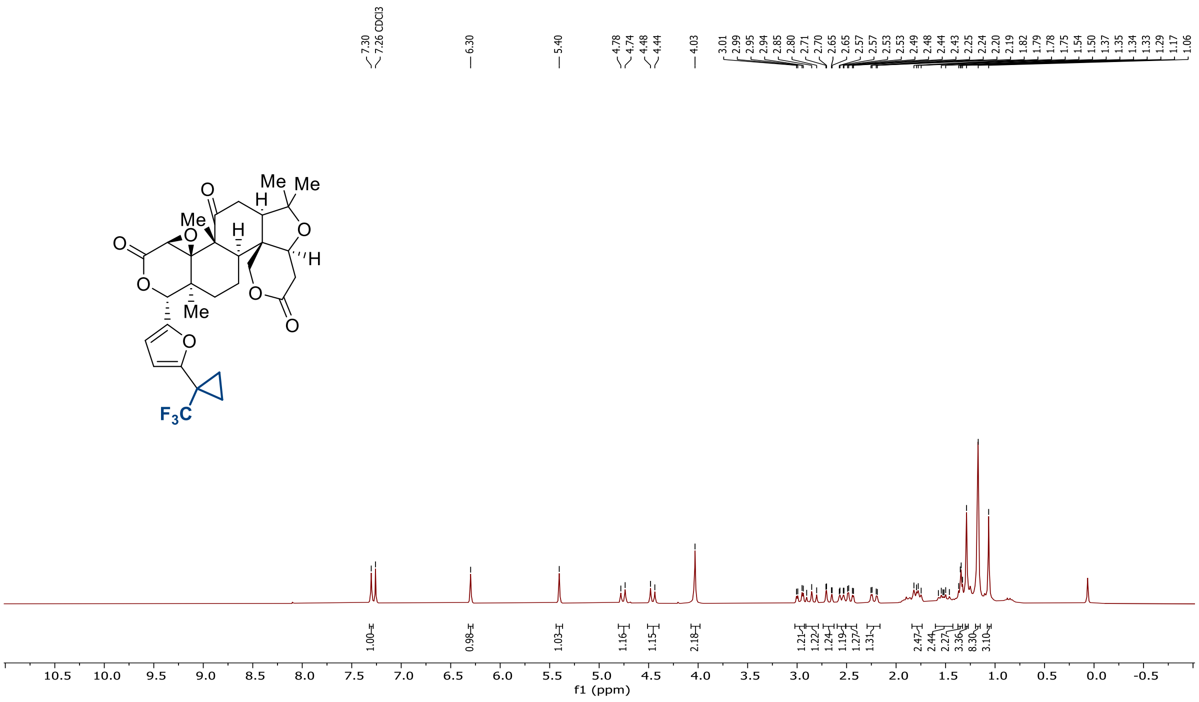


$^{13}$C{1H} NMR (101 MHz, $CDCl_3$)

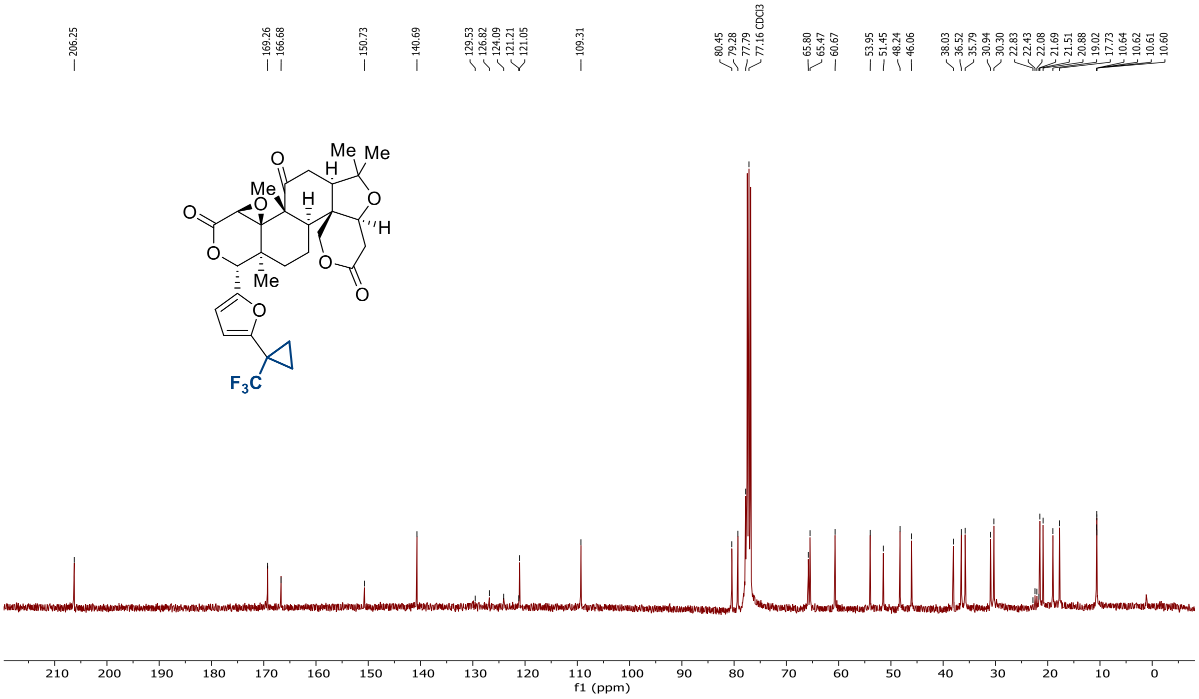

$^{19}F$ NMR (282 MHz, $CDCl_3$)

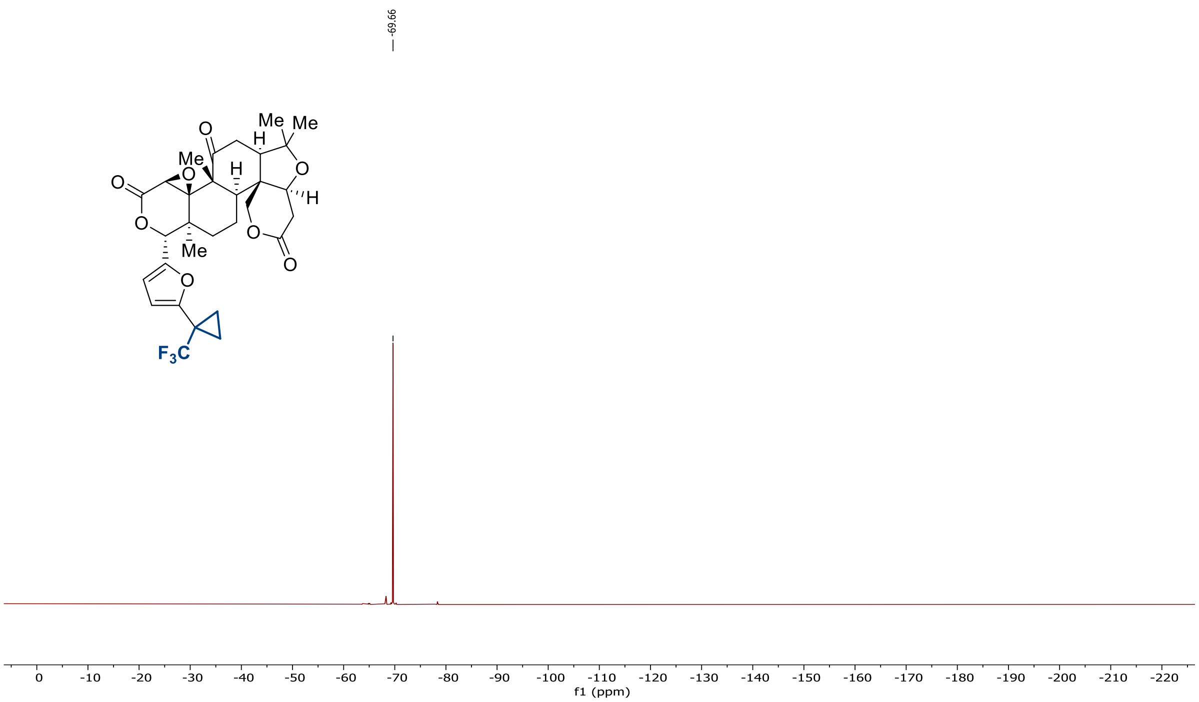


Compound **6af**:

$^{1}H$ NMR (400 MHz, $CDCl_3$)

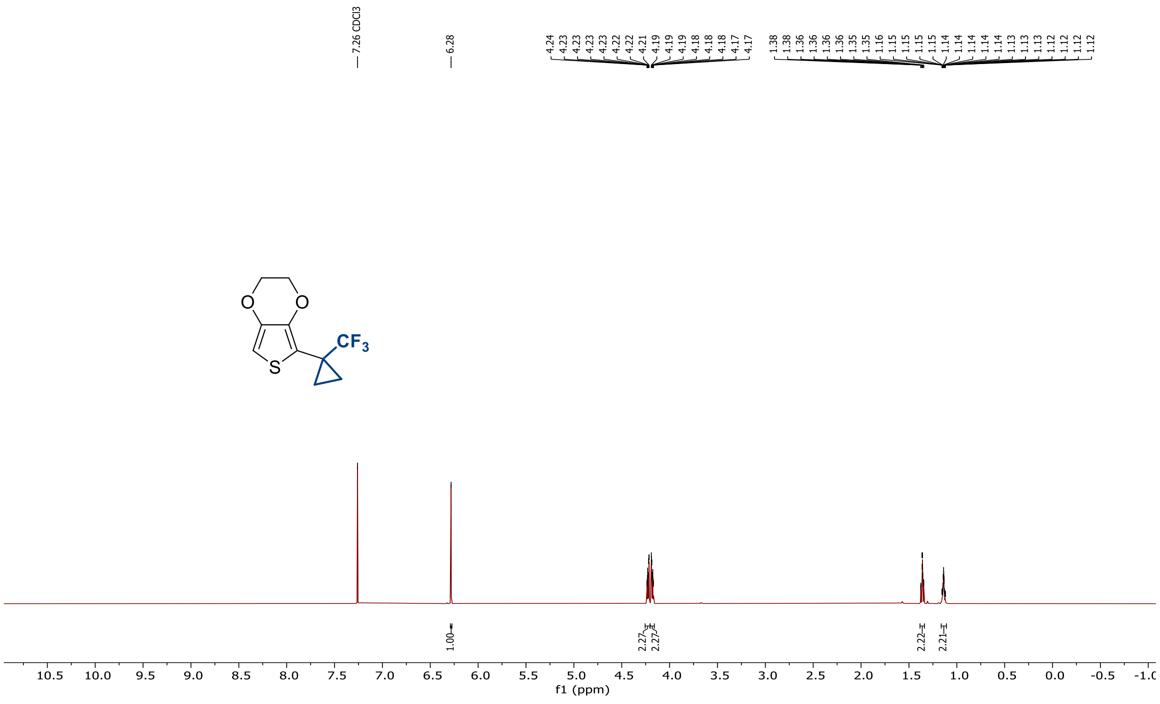

$^{13}C\{1H\}$ NMR (101 MHz, $CDCl_3$)

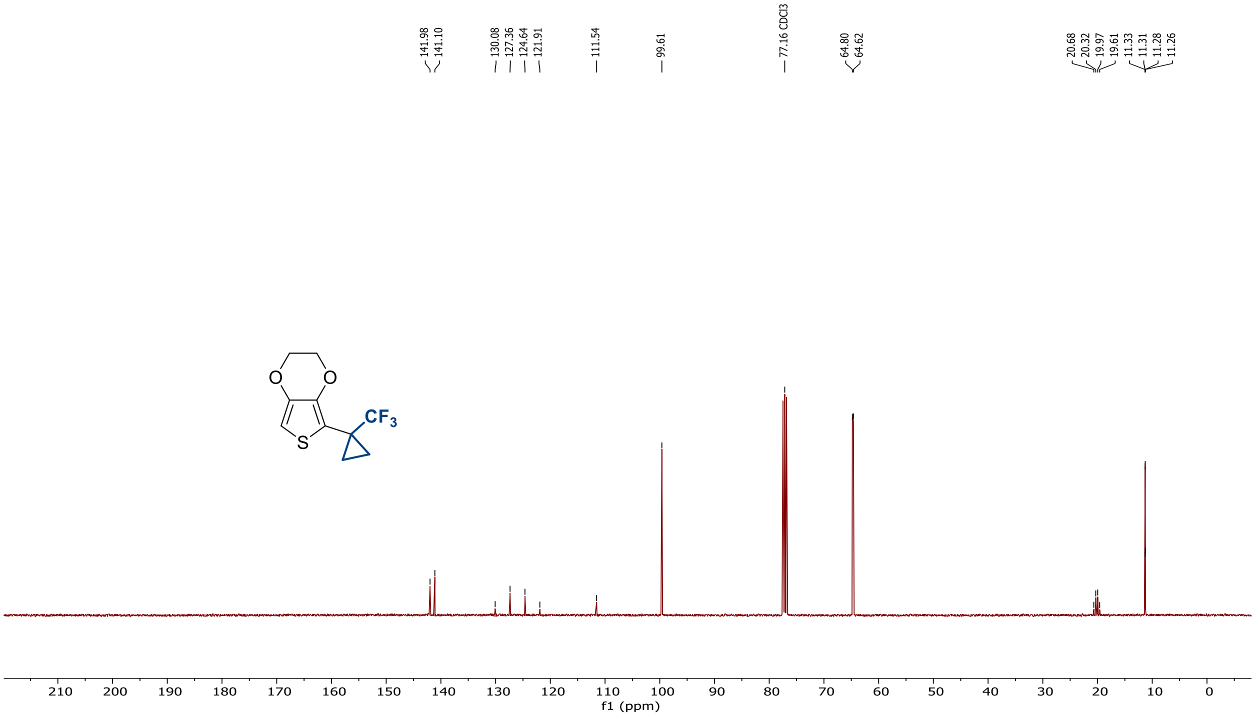


$^{19}F$ NMR (377 MHz, $CDCl_3$)

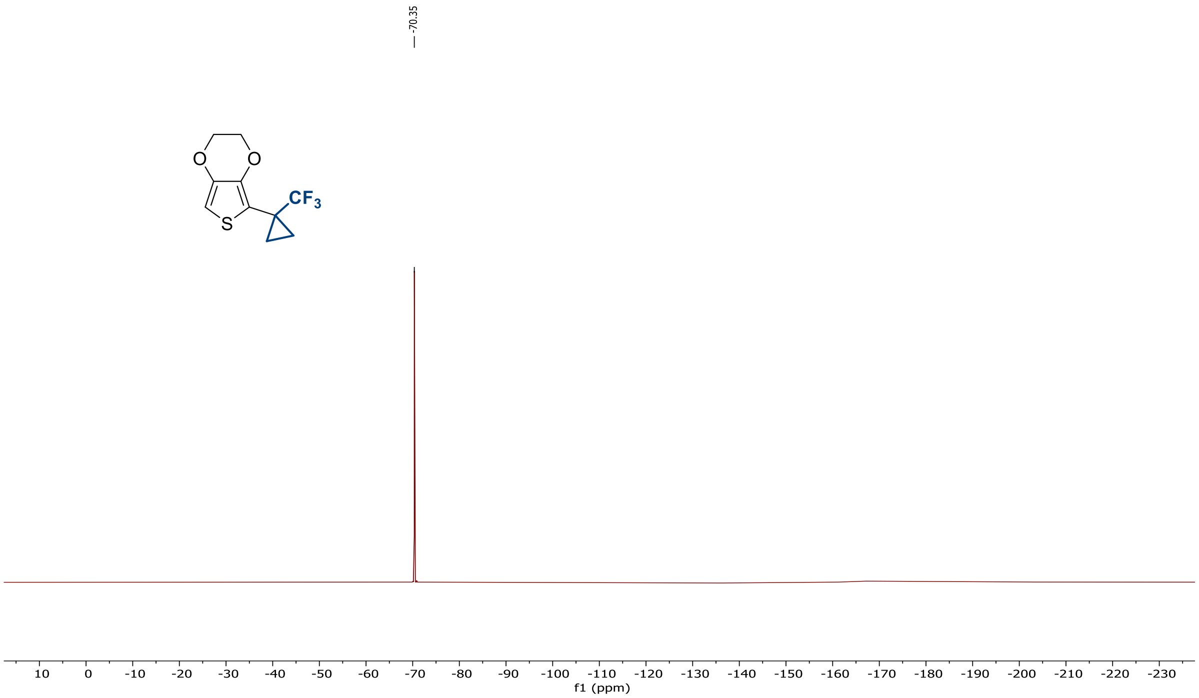

Compound **6ag**:

$^{1}$H NMR (400 MHz, $CDCl_3$)

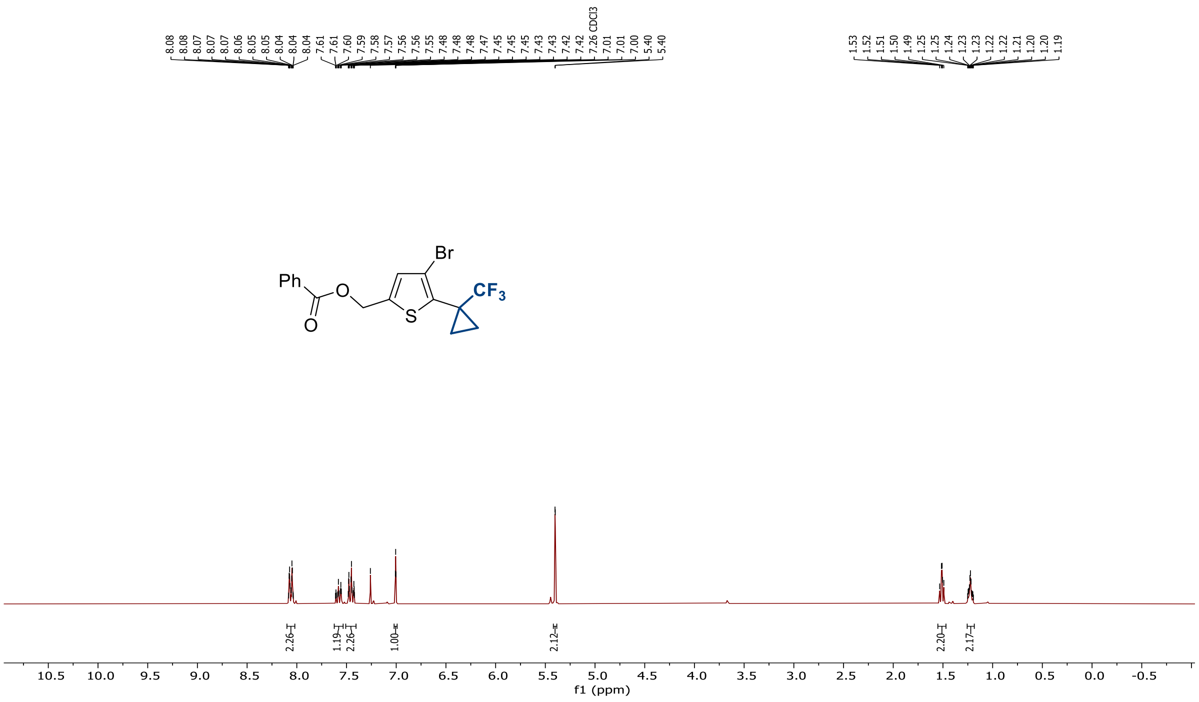


$^{13}$C{1H} NMR (101 MHz, $CDCl_3$)

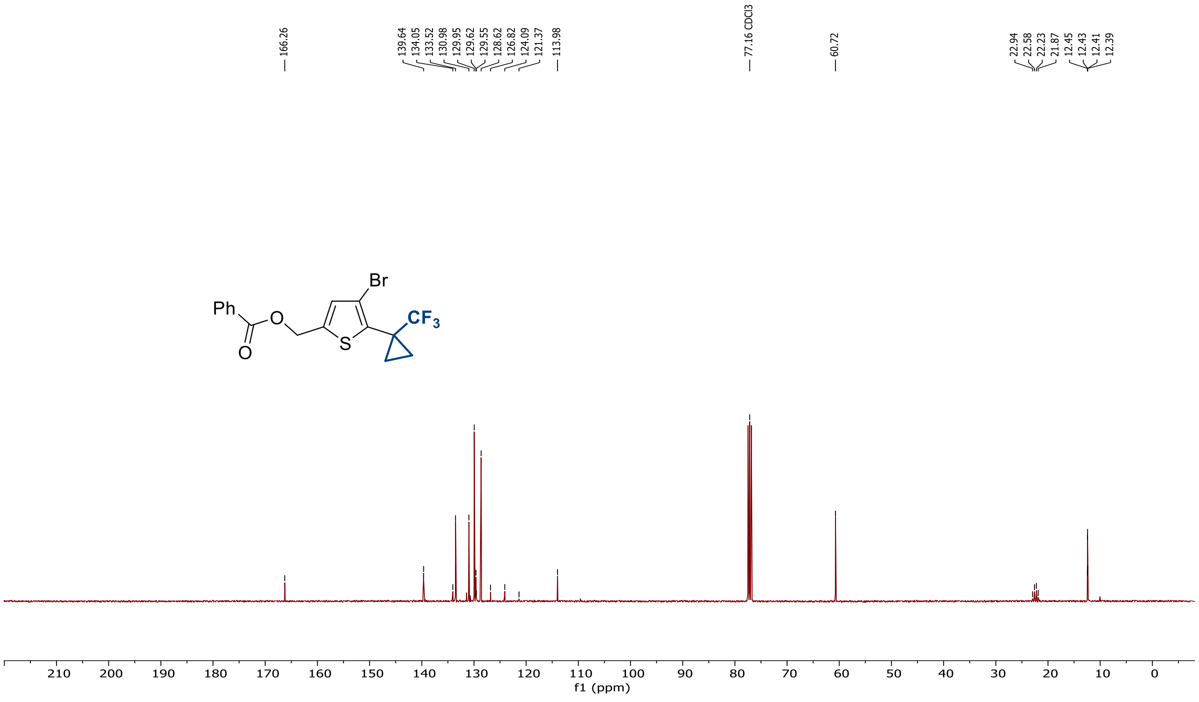

$^{19}F$ NMR (377 MHz, $CDCl_3$)

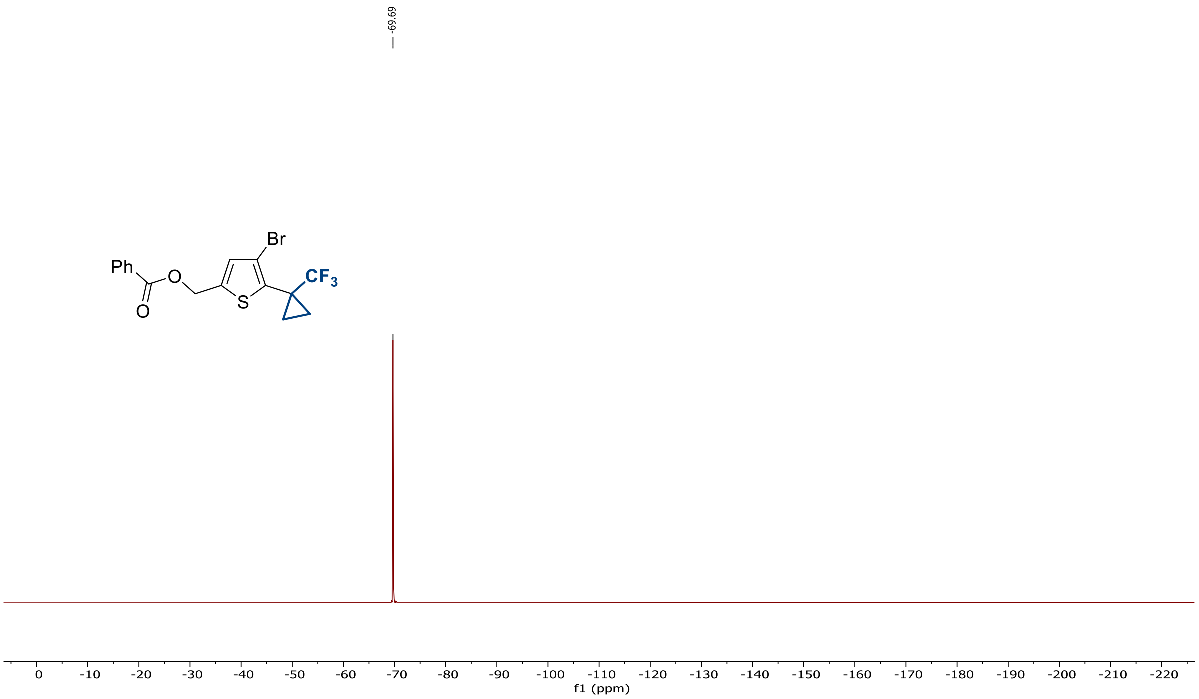


Compound **6ah**:

$^{1}H$ NMR (400 MHz, $CDCl_3$)

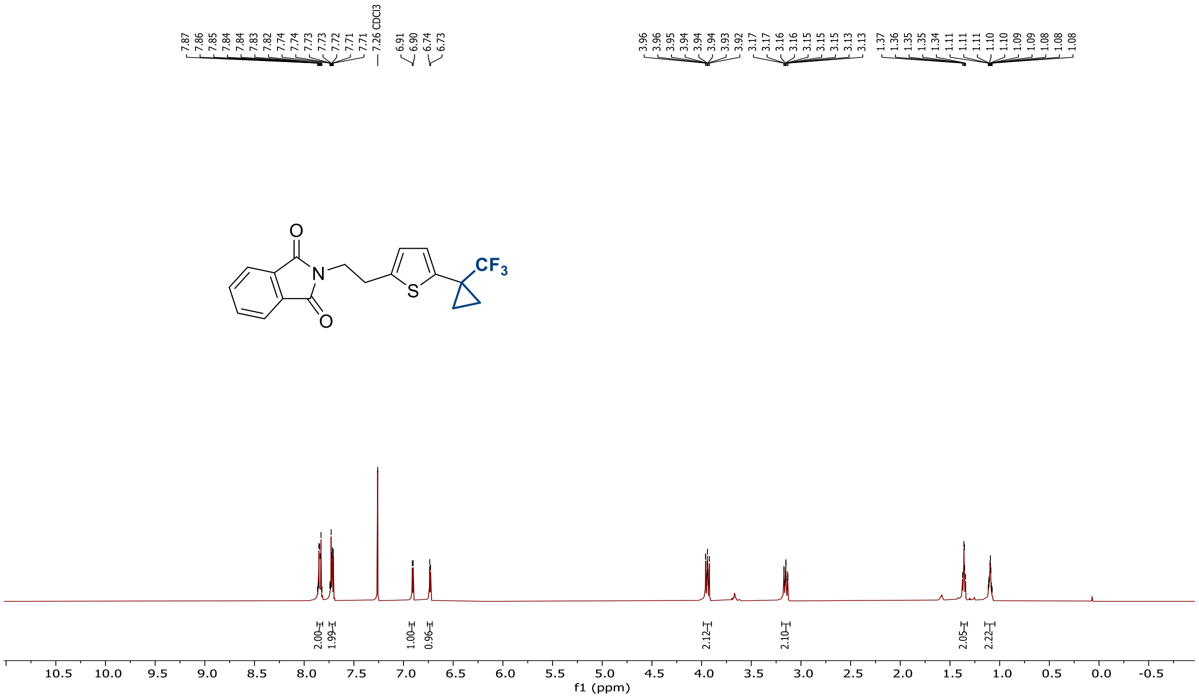

$^{13}C\{1H\}$ NMR (101 MHz, $CDCl_3$)

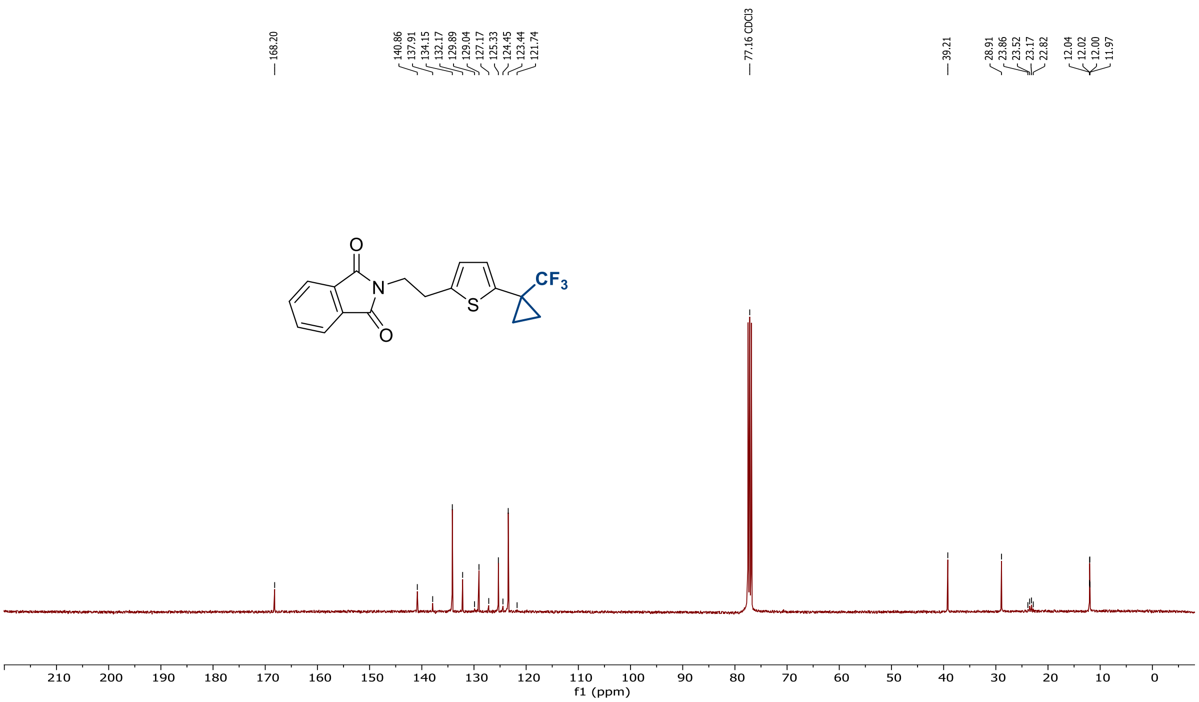


$^{19}F$ NMR (377 MHz, $CDCl_3$)

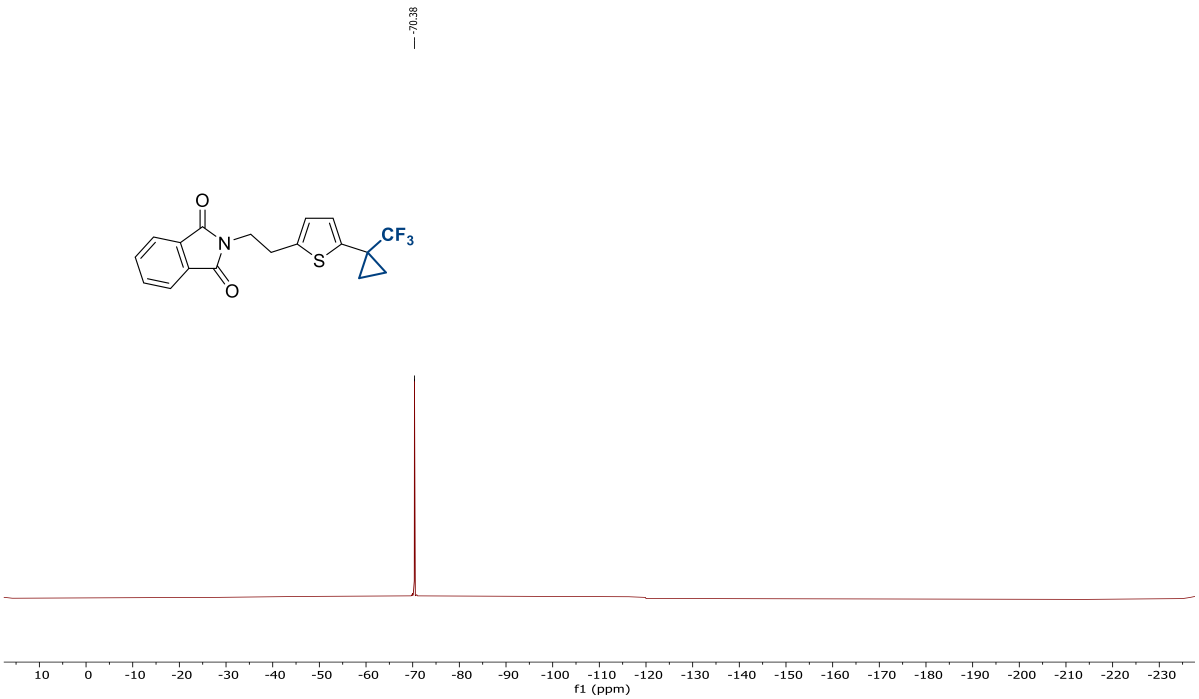

Compound **6ai**:

$^{1}H$ NMR (300 MHz, $CDCl_3$)

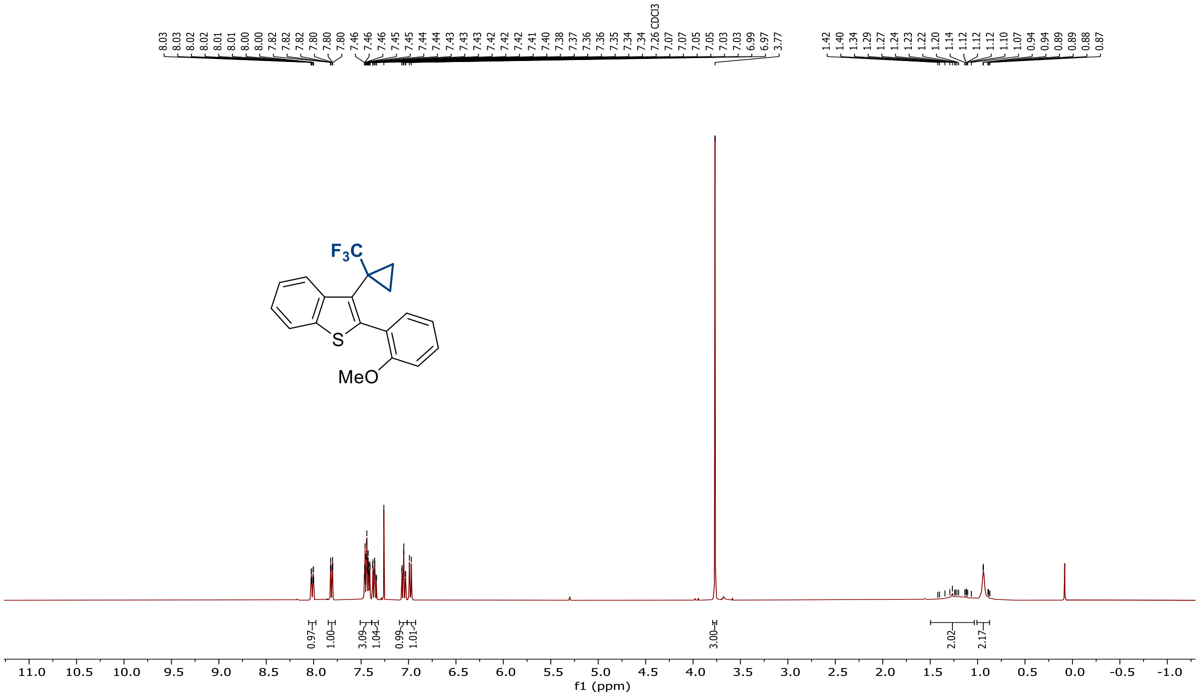


$^{13}C\{1H\}$ NMR (101 MHz, $CDCl_3$)

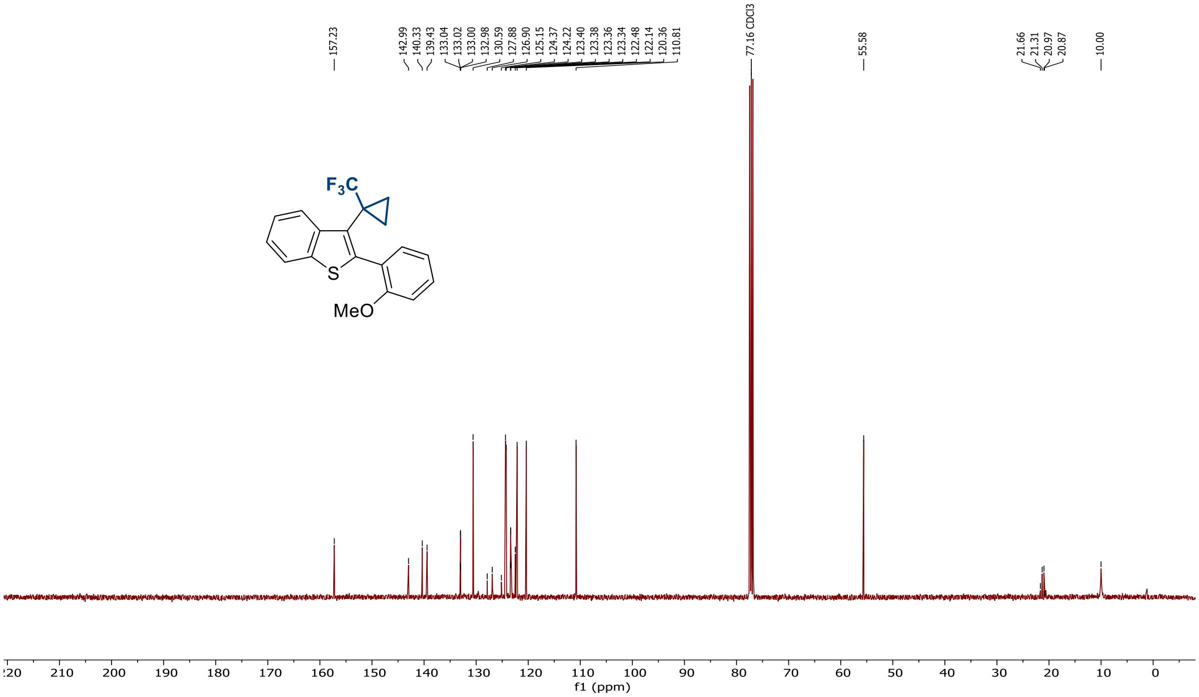

$^{19}$F NMR (377 MHz, $CDCl_3$)

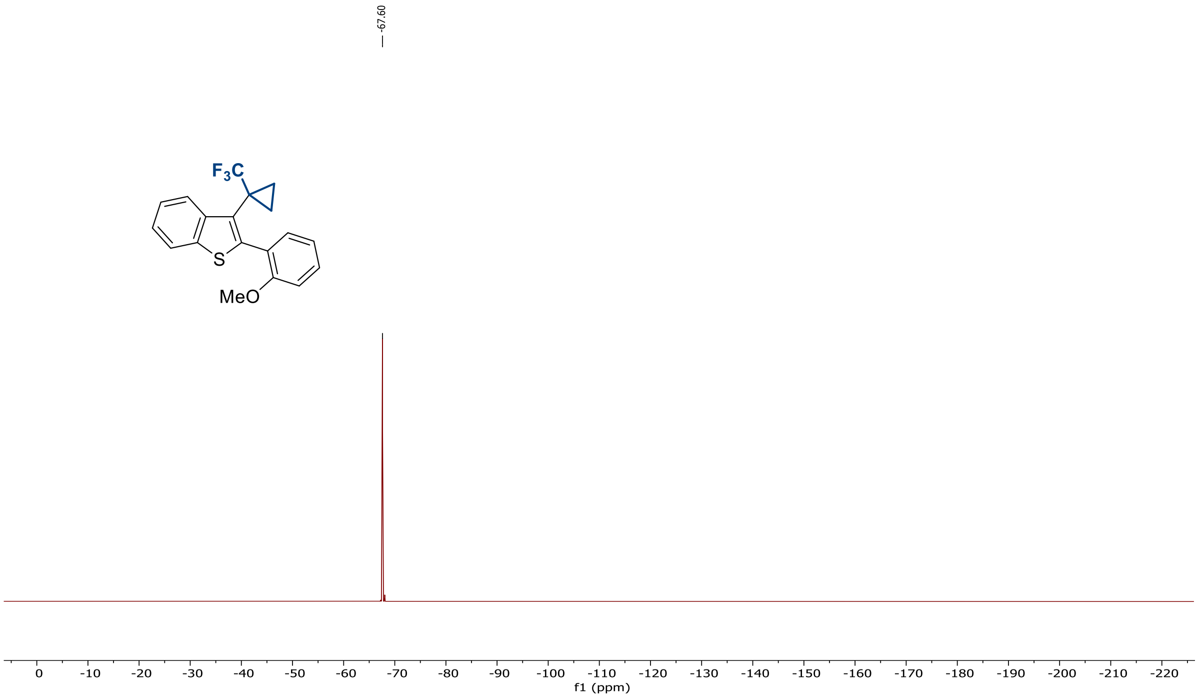


Compound **4w-d$_4$**:

$^{1}$H NMR (400 MHz, $CDCl_3$)

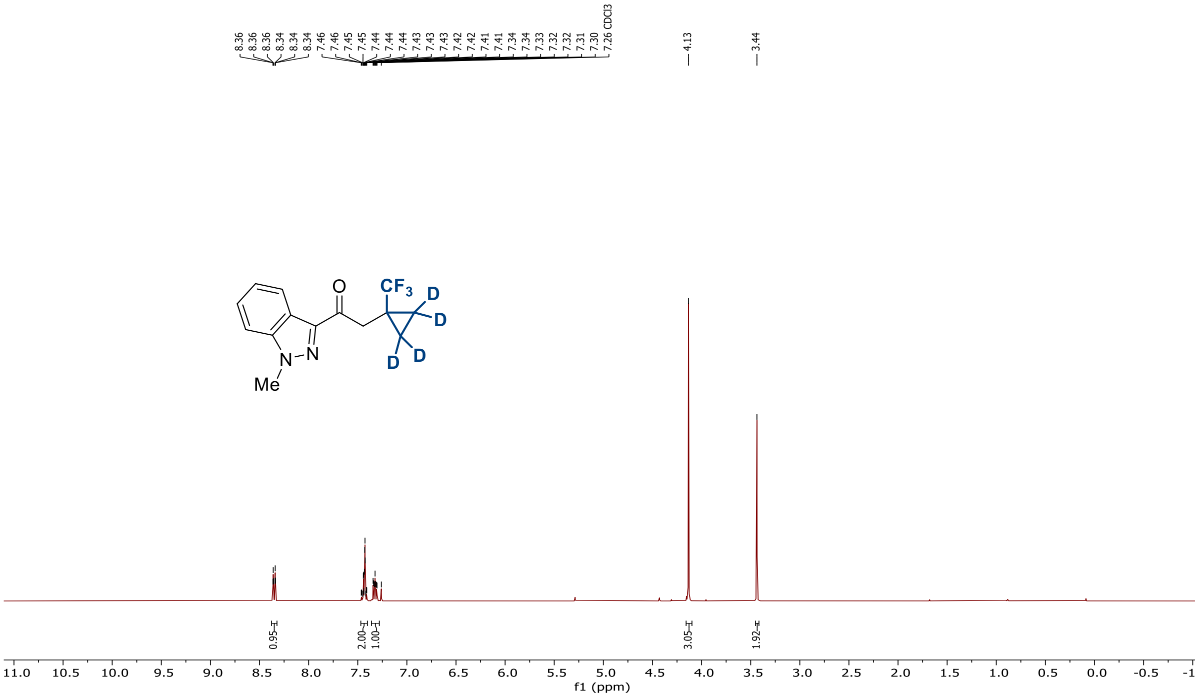

$^{13}C\{1H\}$ NMR (101 MHz, $CDCl_3$)

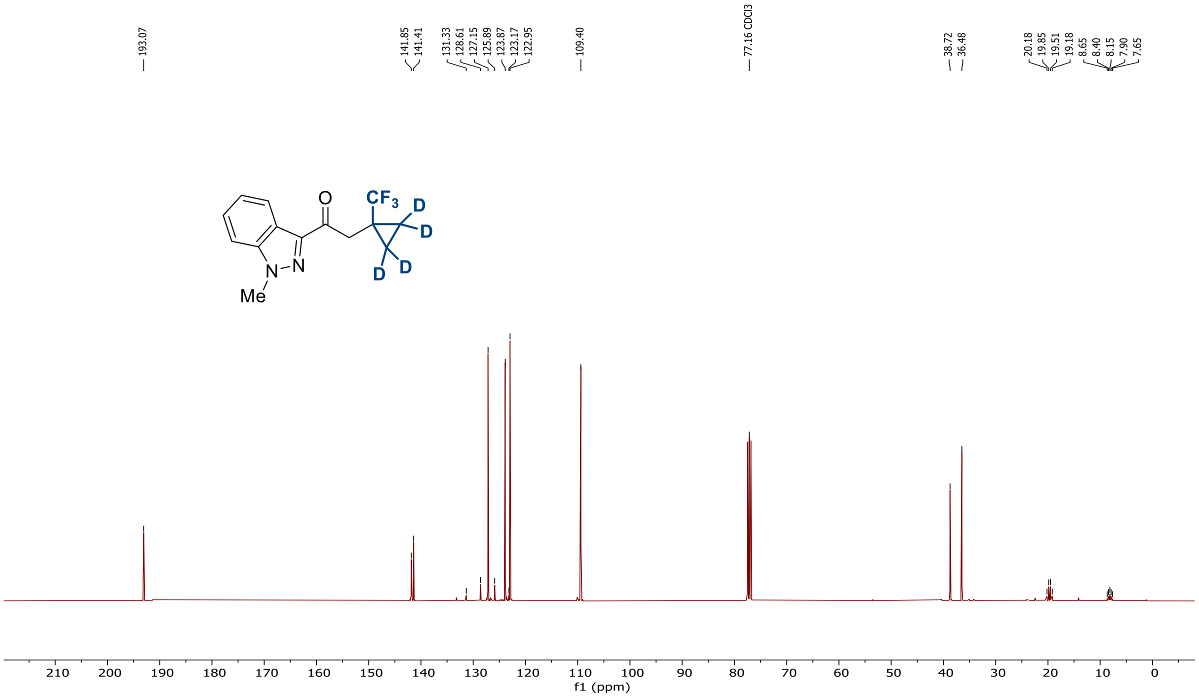


$^{19}F$ NMR (377 MHz, $CDCl_3$)

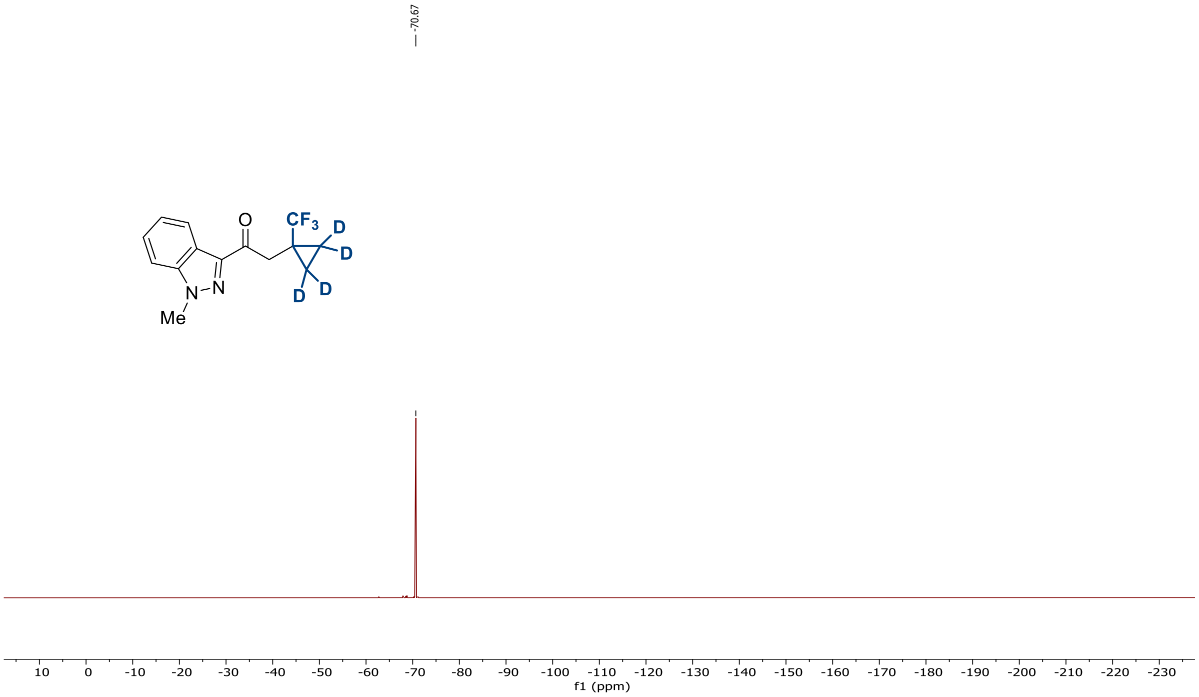

$^2H$ NMR (92 MHz, $CHCl_3$)

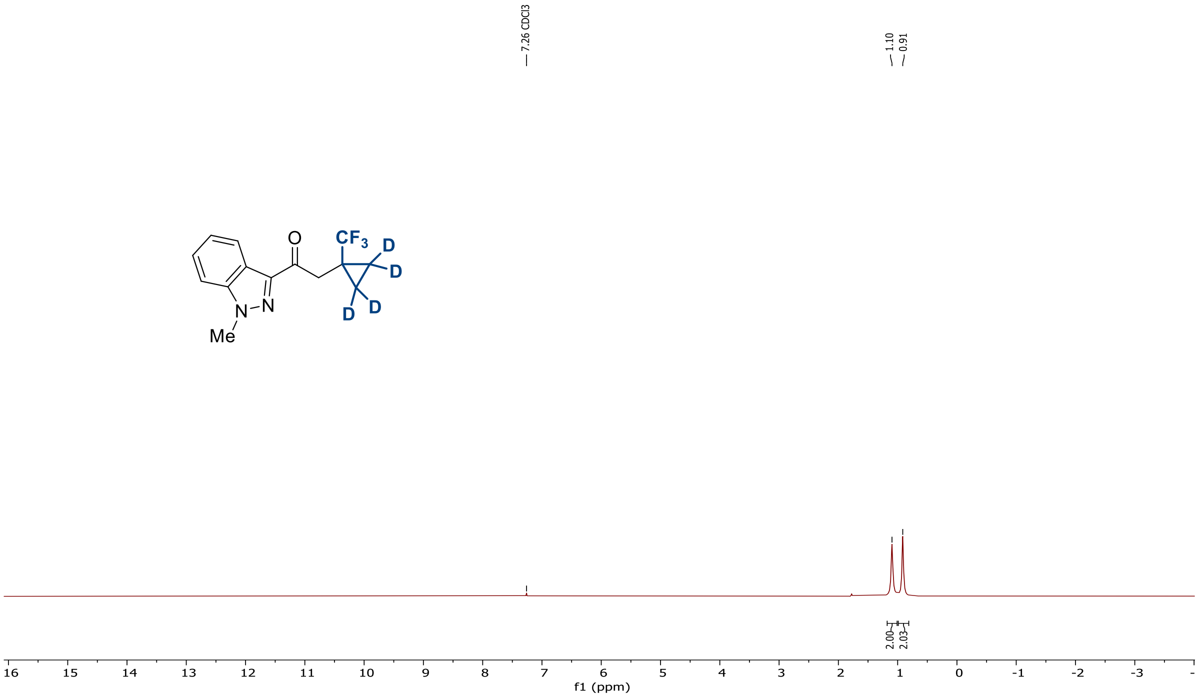


Compound **4u-d$_4$**:

$^1H$ NMR (300 MHz, $CDCl_3$)

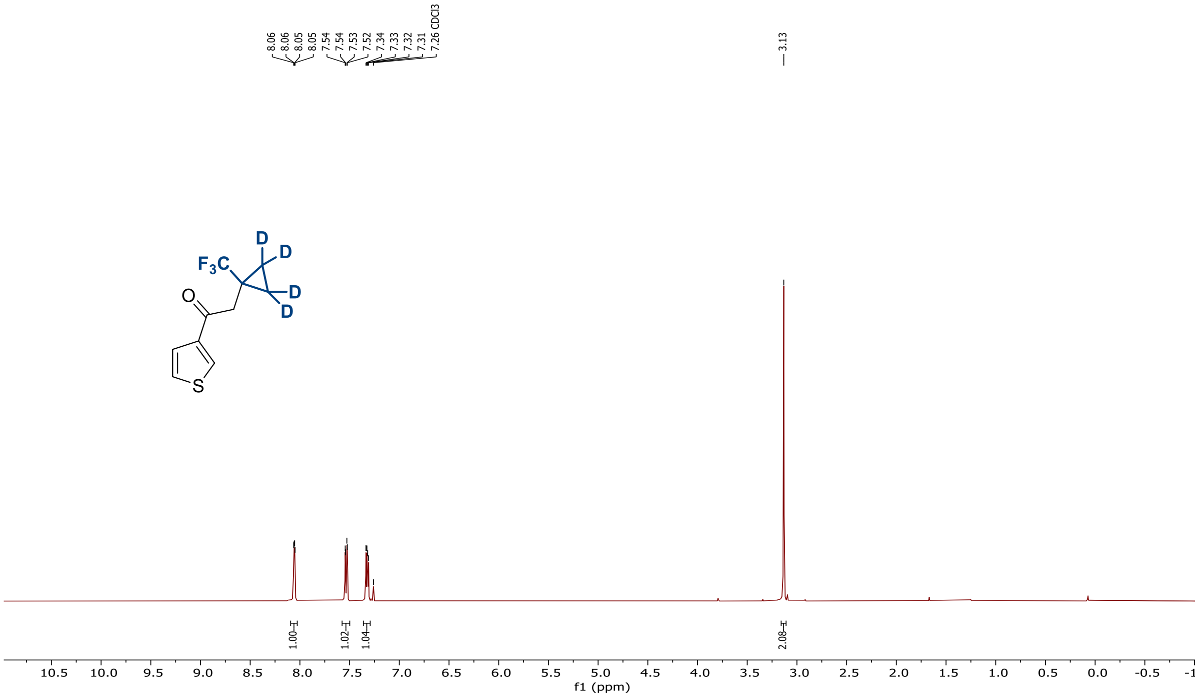

$^{13}C\{1H\}$ NMR (101 MHz, $CDCl_3$)

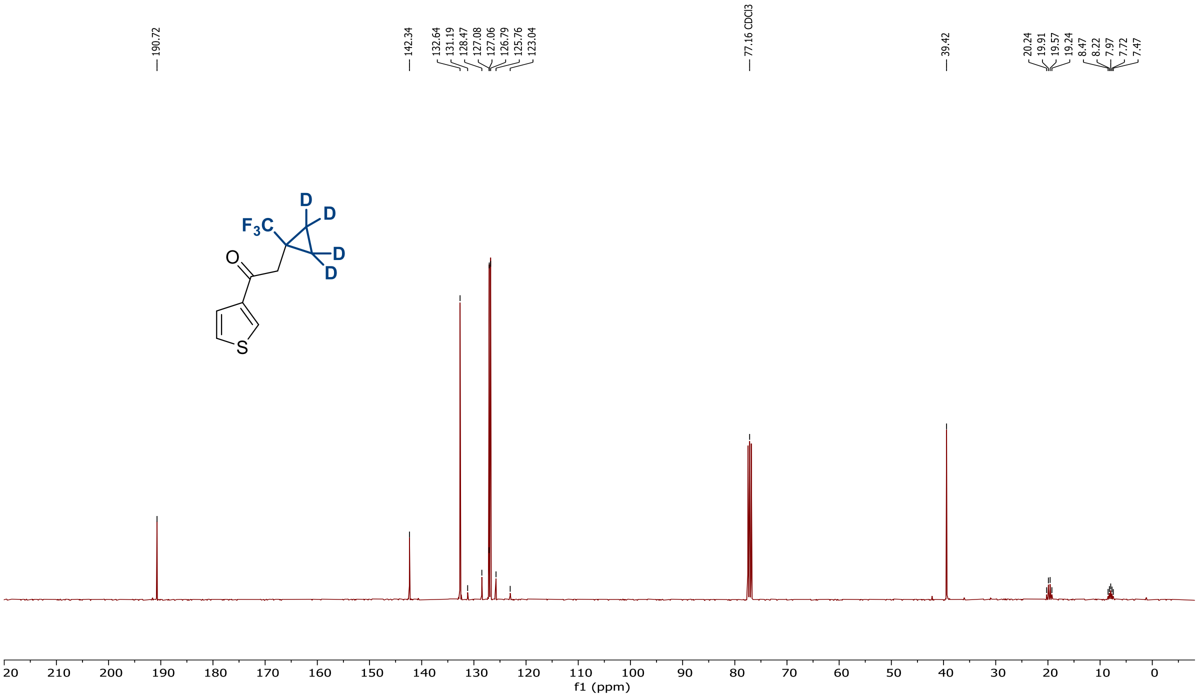


$^{19}F$ NMR (282 MHz, $CDCl_3$)

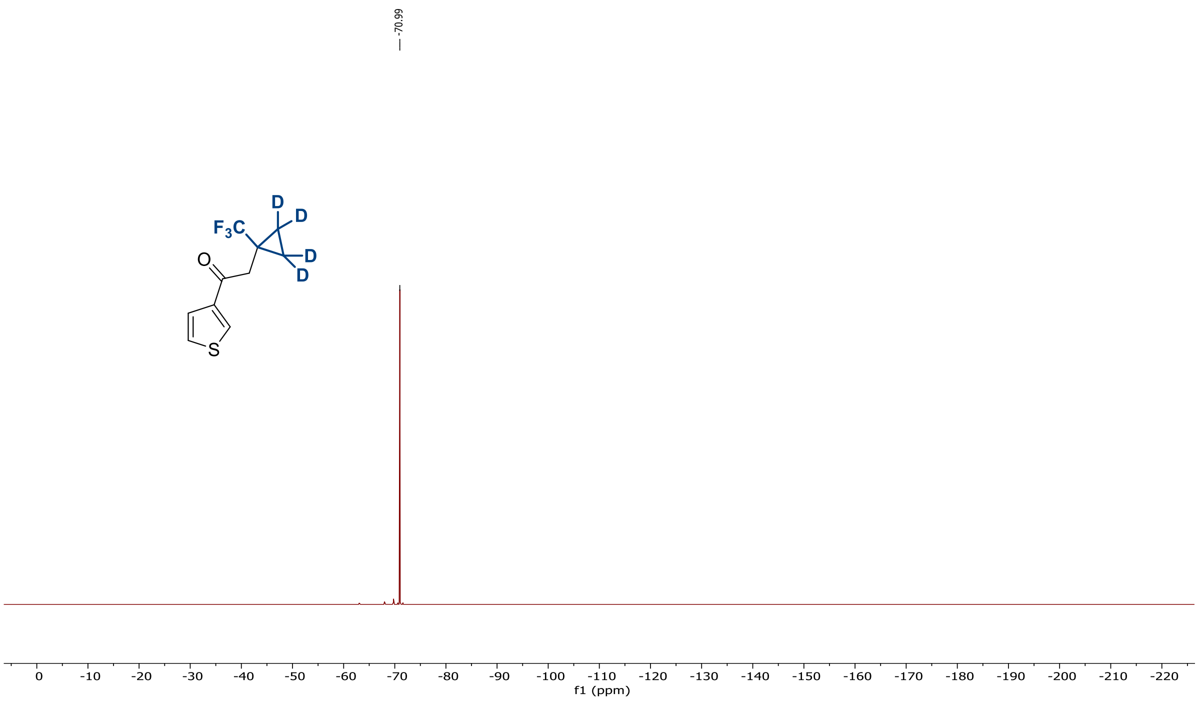

$^{2}H$ NMR (92 MHz, $CHCl_3$)

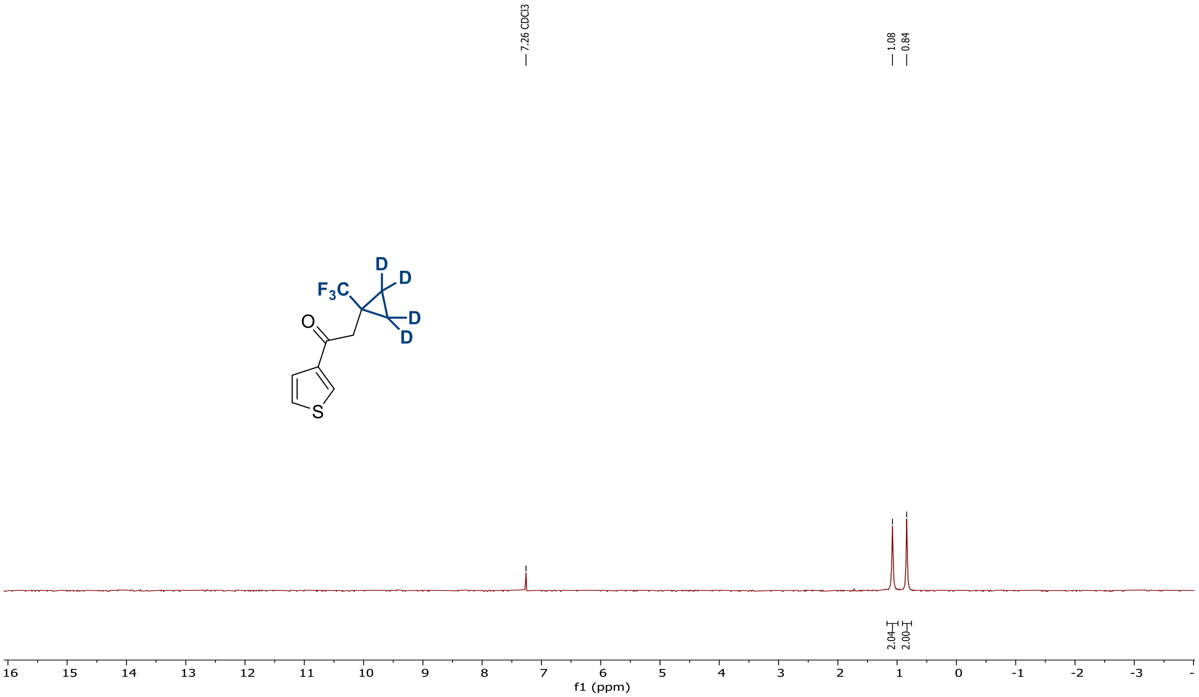


Compound **6a-d$_4$**:

$^{1}H$ NMR (400 MHz, $CDCl_3$)

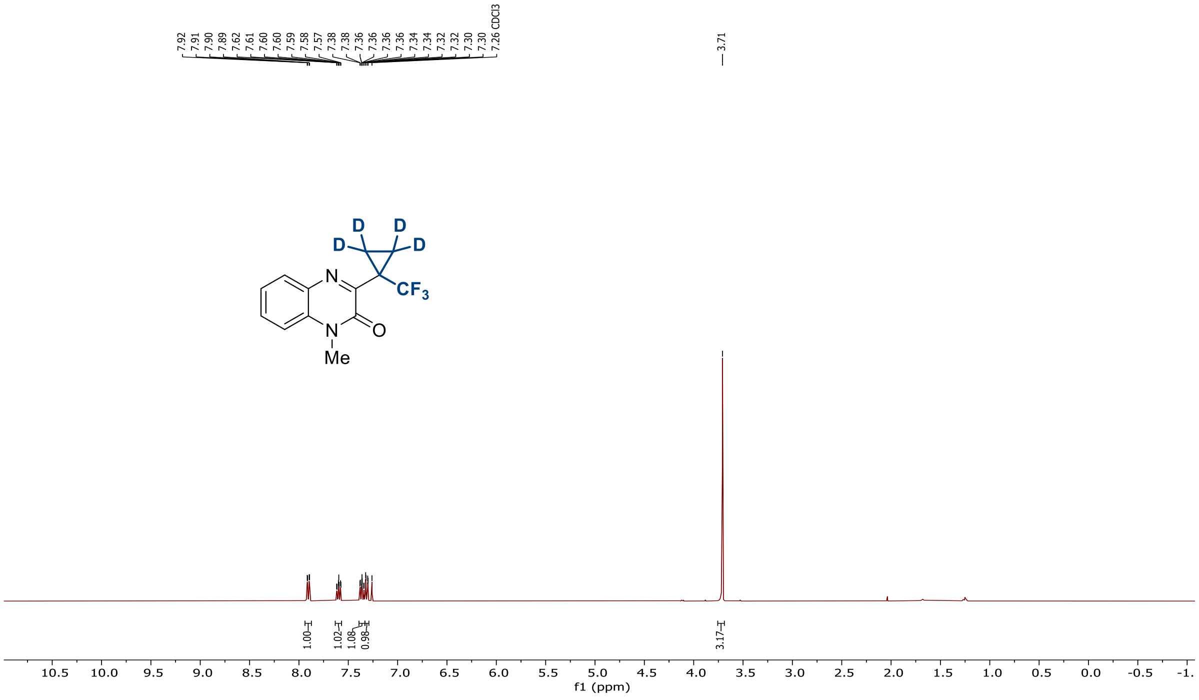

$^{13}C\{1H\}$ NMR (101 MHz, $CDCl_3$)

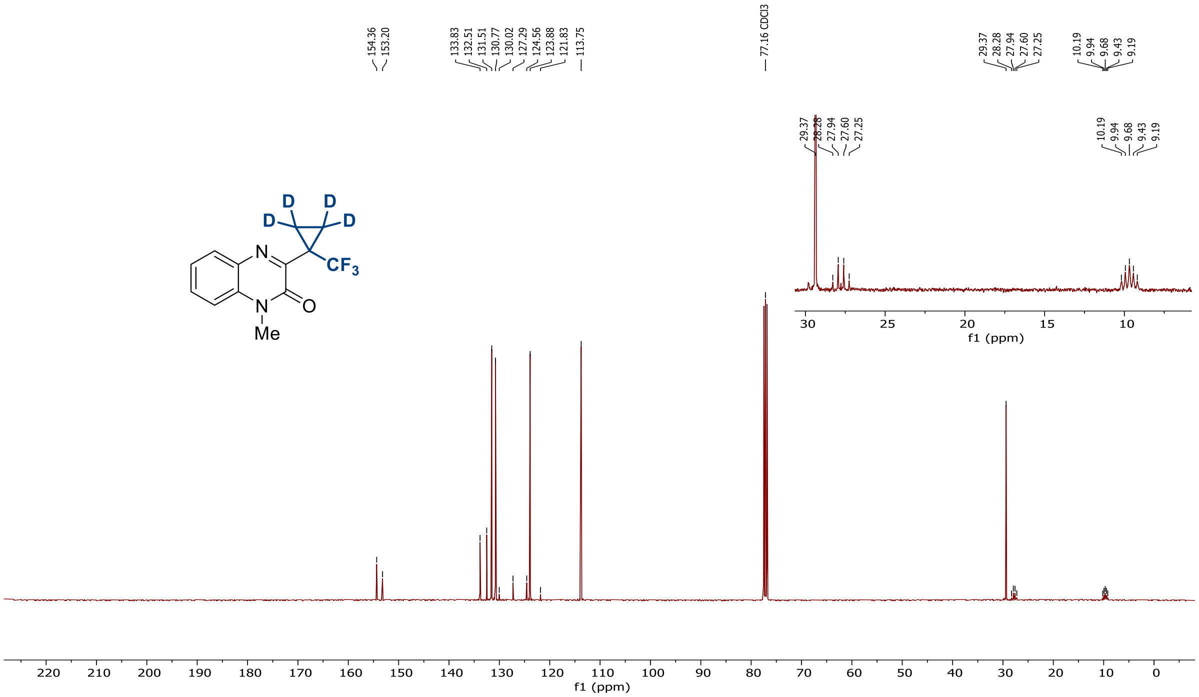


$^{19}F$ NMR (377 MHz, $CDCl_3$)

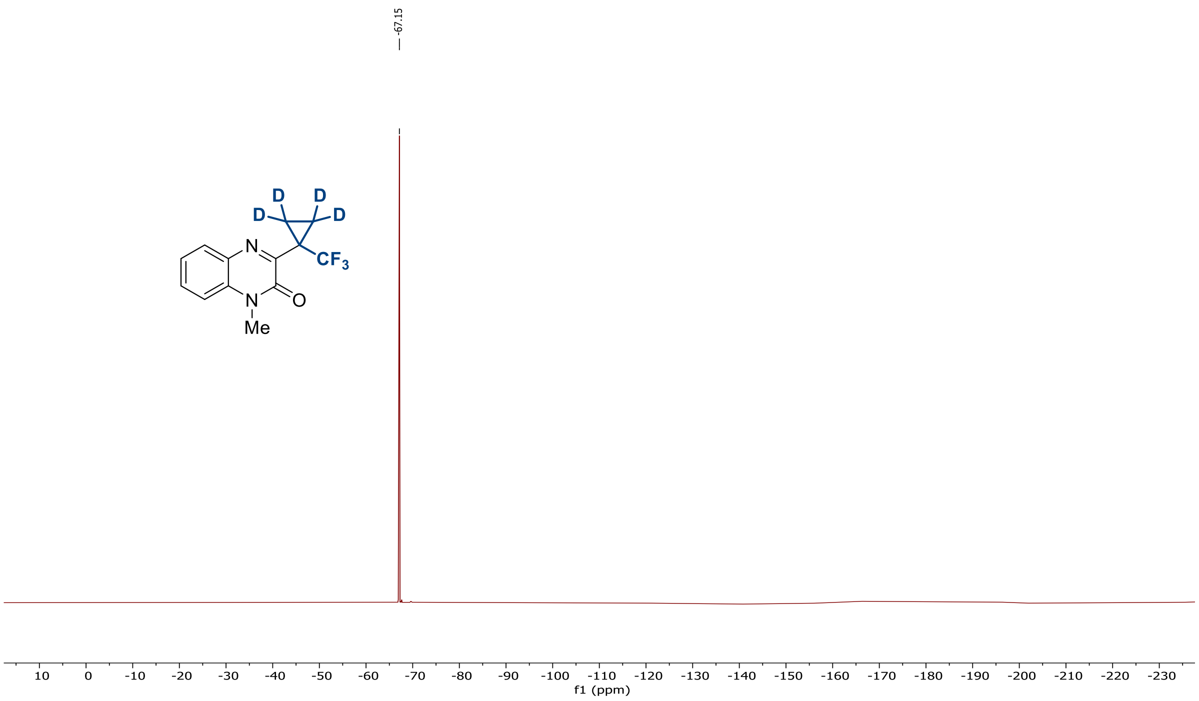

$^{2}$H NMR (92 MHz, $CHCl_3$)

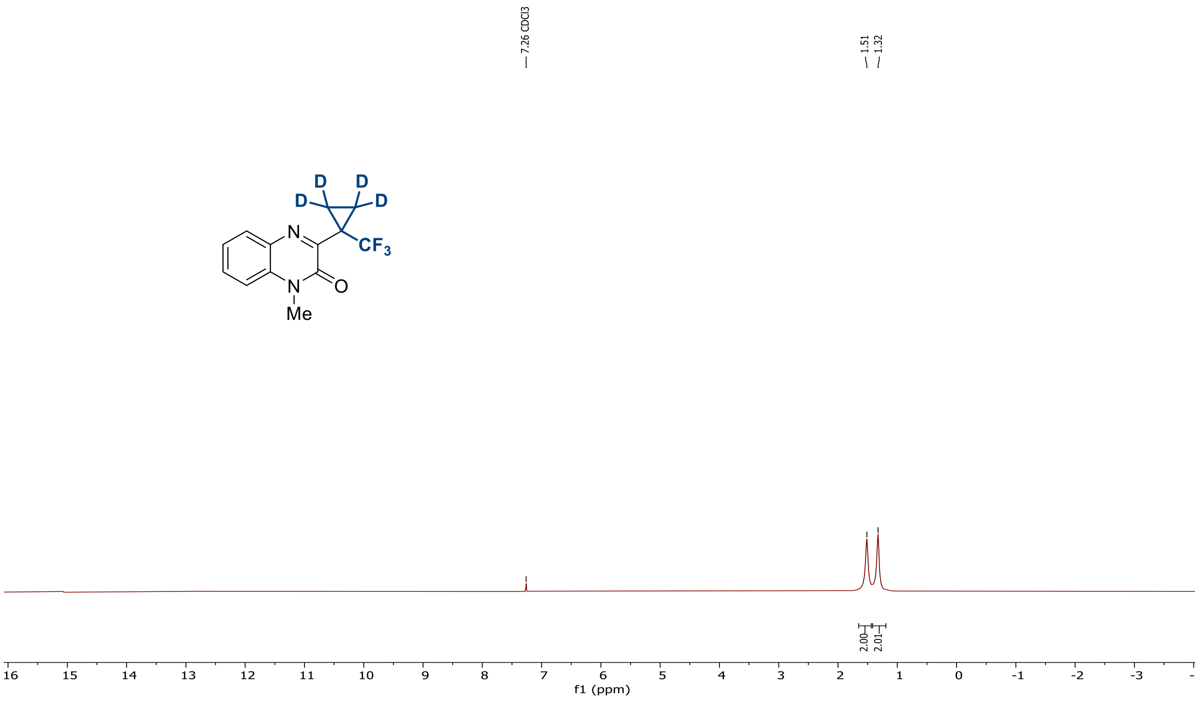


Compound **6m-d$_4$**:

$^{1}$H NMR (400 MHz, $CDCl_3$)

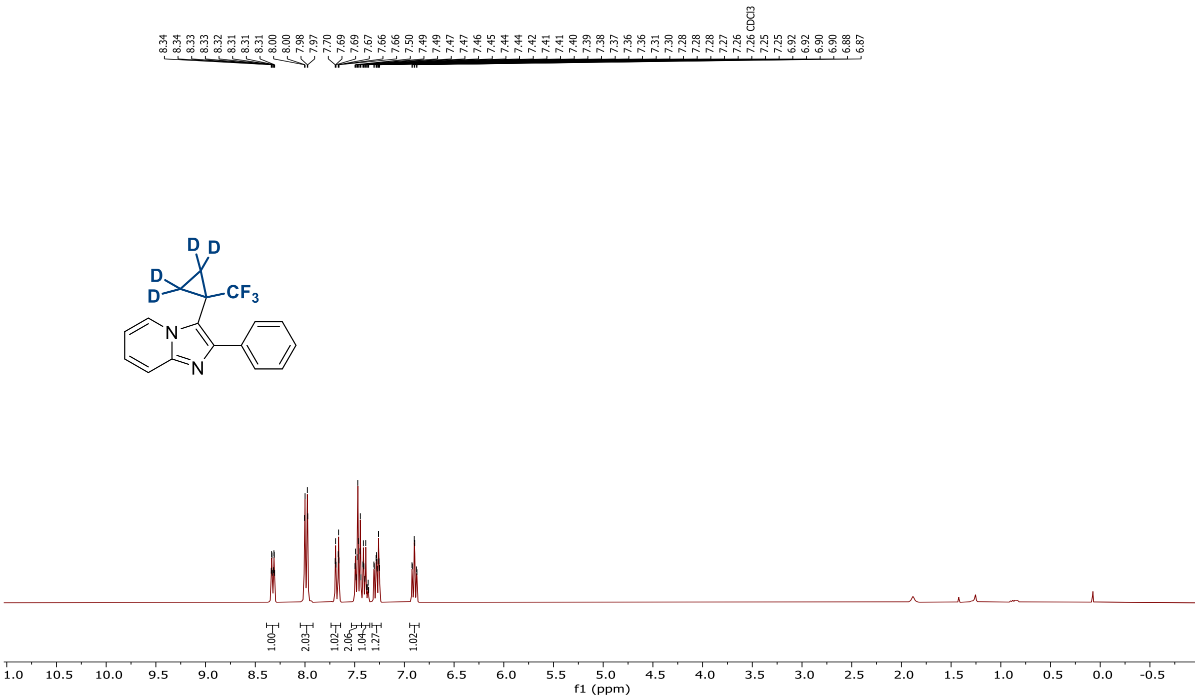

$^{13}C\{1H\}$ NMR (101 MHz, $CDCl_3$)

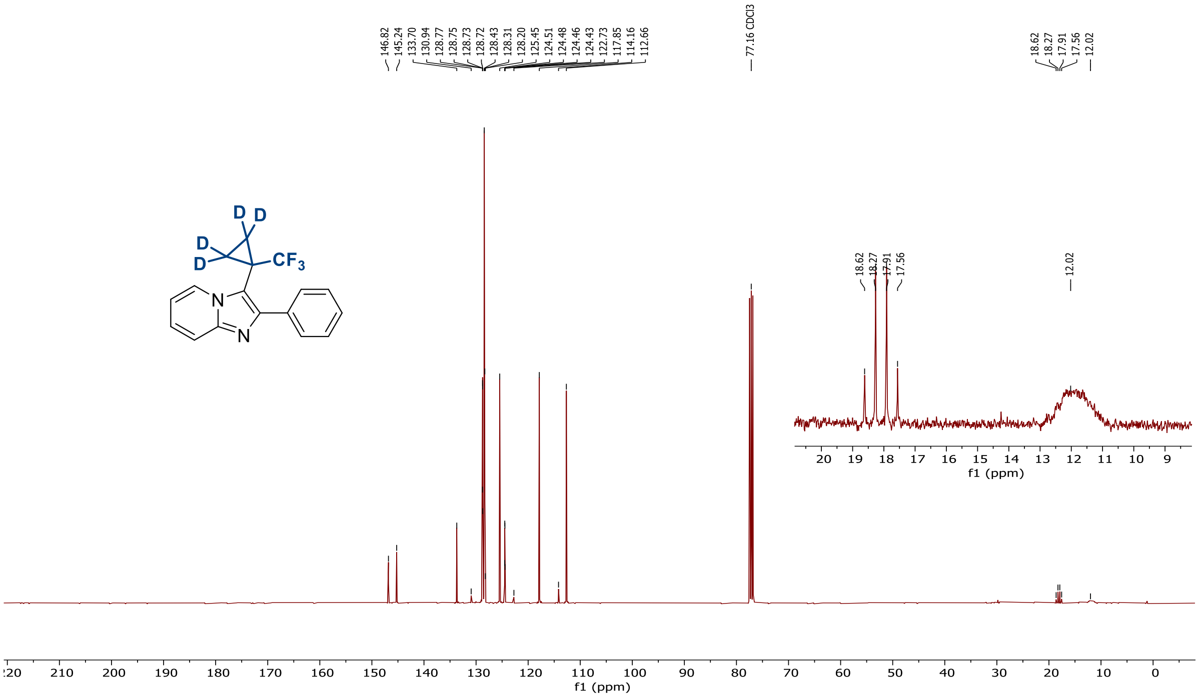


$^{19}F$ NMR (377 MHz, $CDCl_3$)

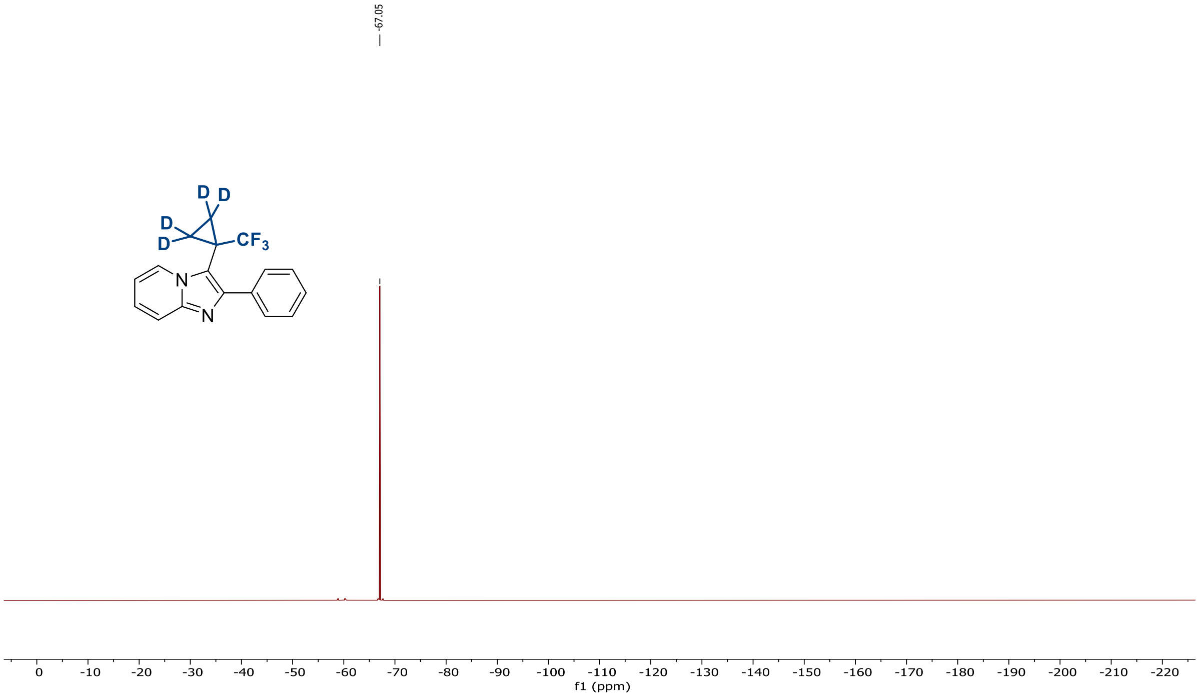

$^2$H NMR (92 MHz, $CHCl_3$)

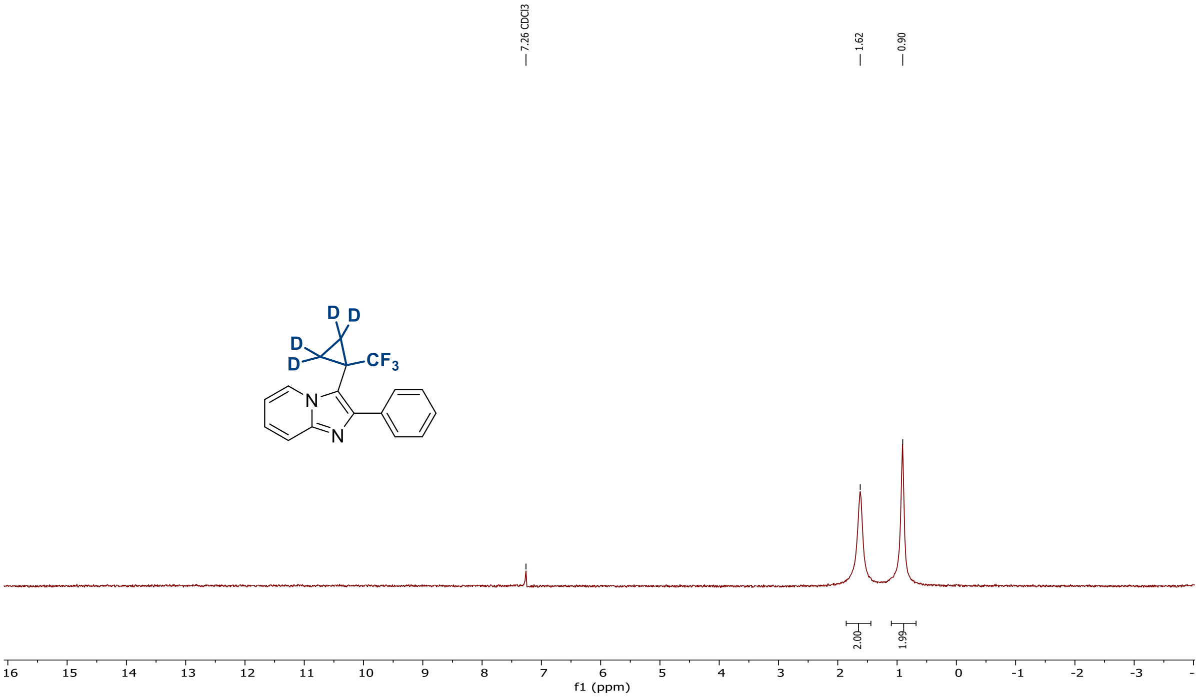


Compound **6q-d₄**:

$^1$H NMR (400 MHz, $CDCl_3$)

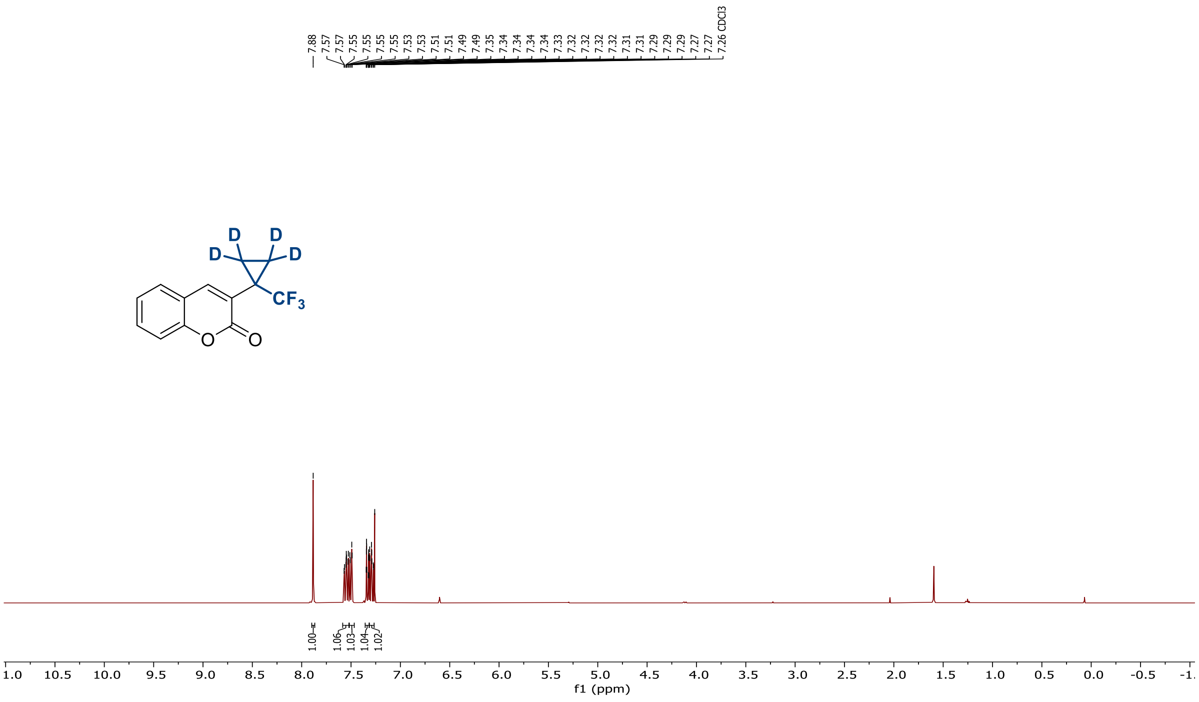

$^{13}C\{1H\}$ NMR (101 MHz, $CDCl_3$)

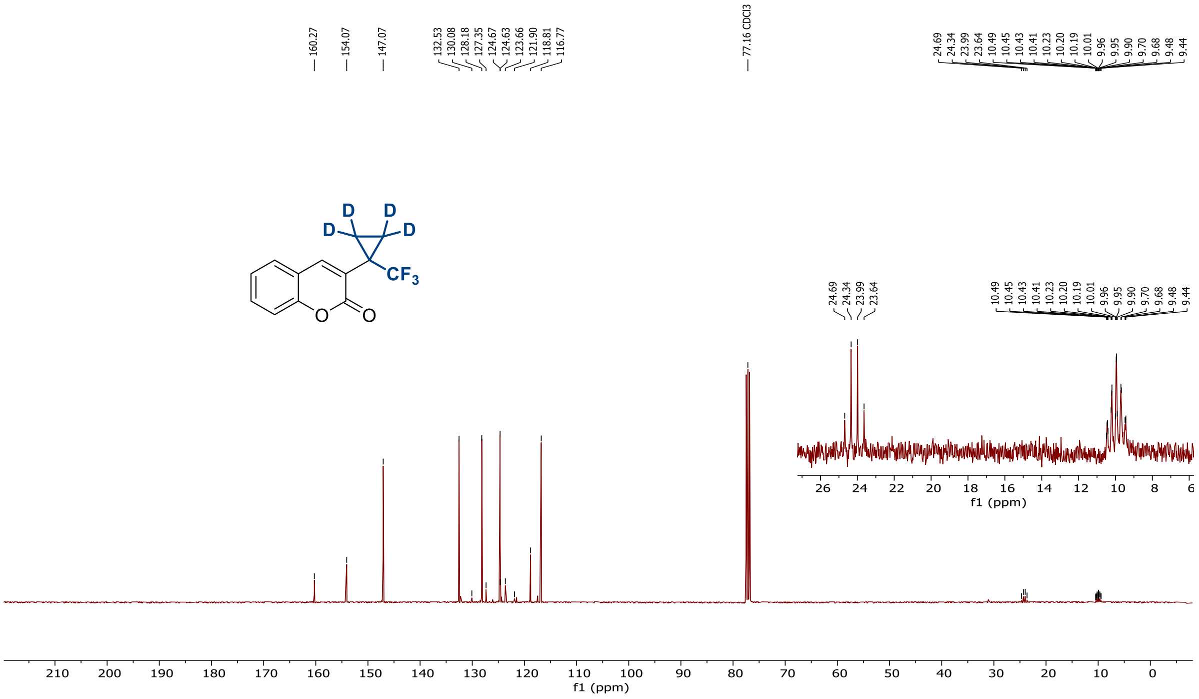


$^{19}F$ NMR (377 MHz, $CDCl_3$)

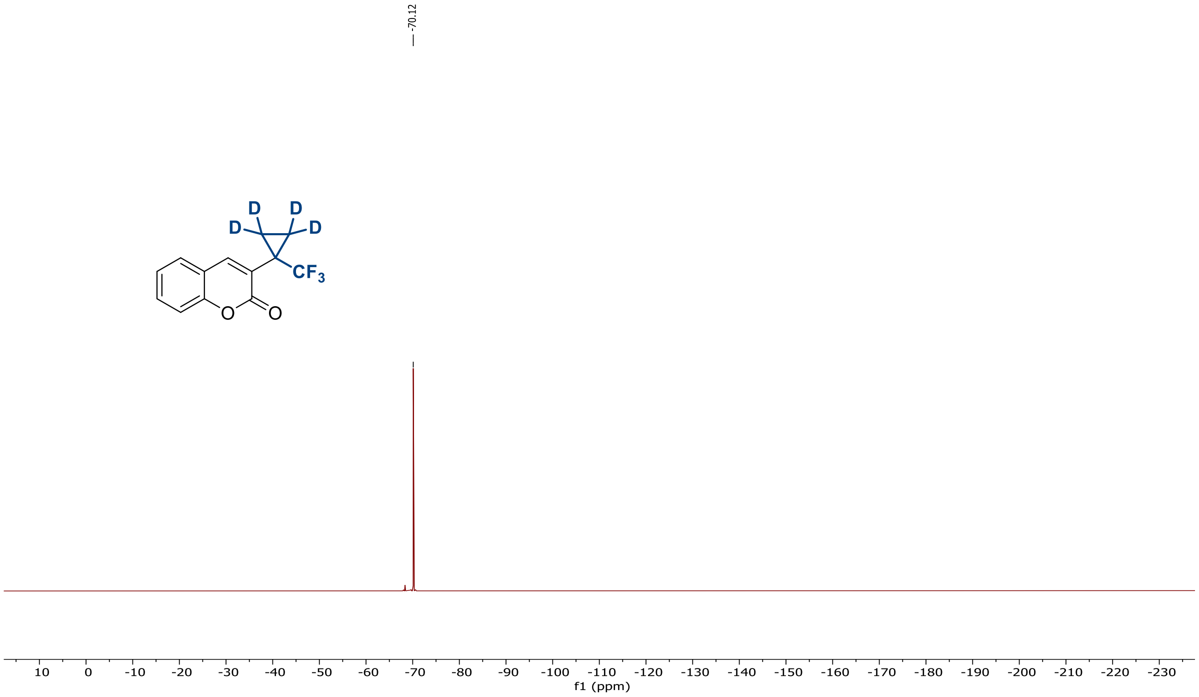

$^2H$ NMR (92 MHz, $CHCl_3$)

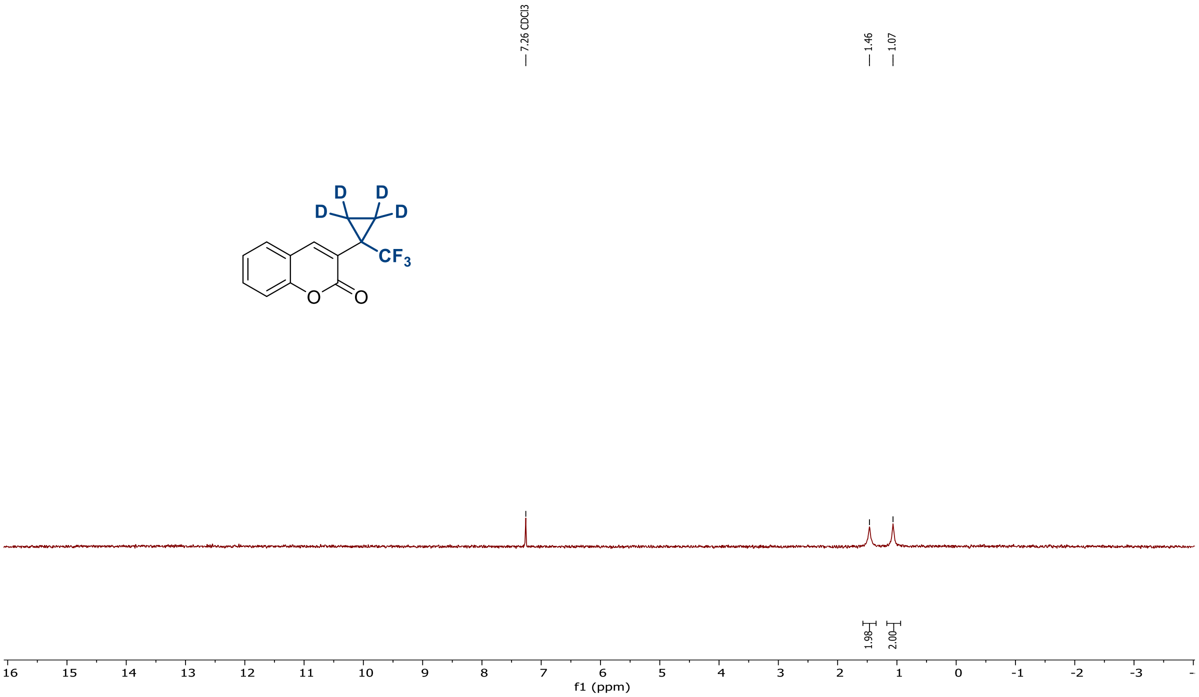


Compound **6ad-d$_4$**:

$^1H$ NMR (400 MHz, $CDCl_3$)

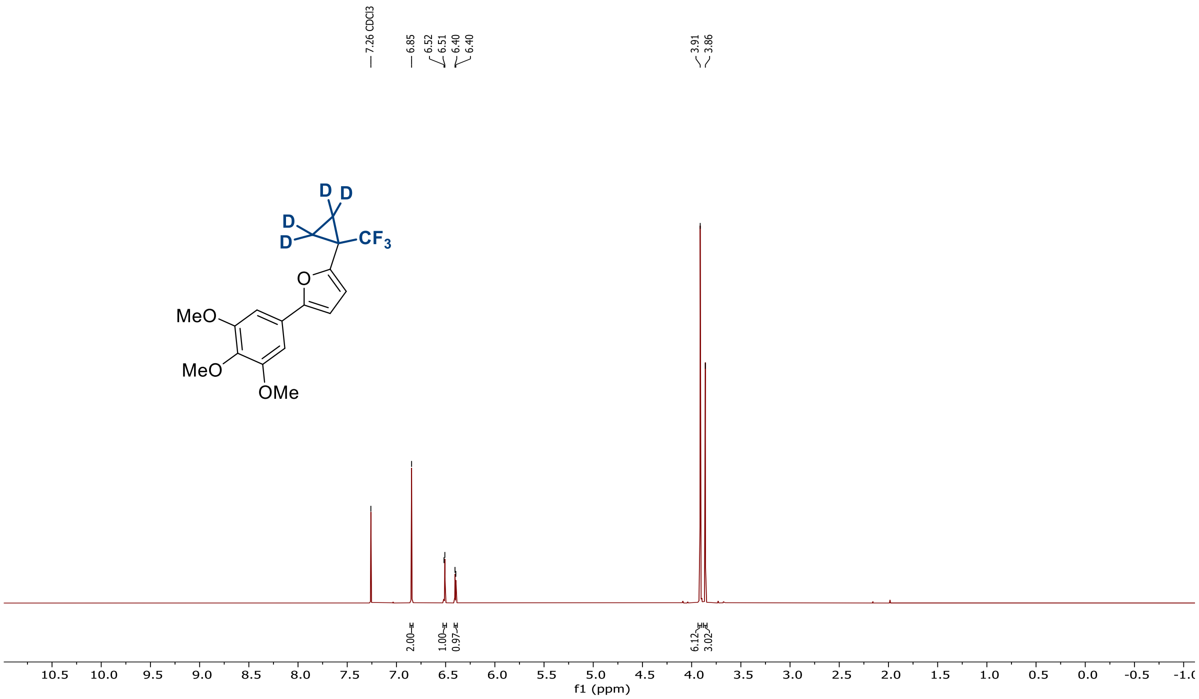

$^{13}C\{1H\}$ NMR (101 MHz, $CDCl_3$)

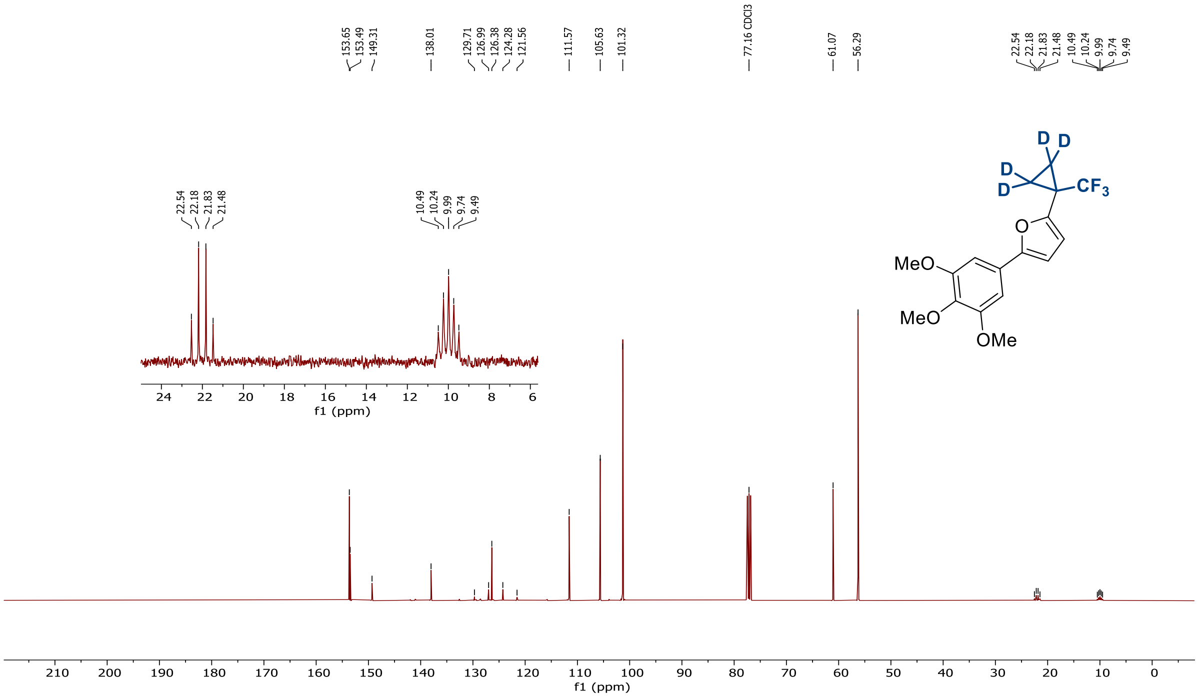


$^{19}F$ NMR (377 MHz, $CDCl_3$)

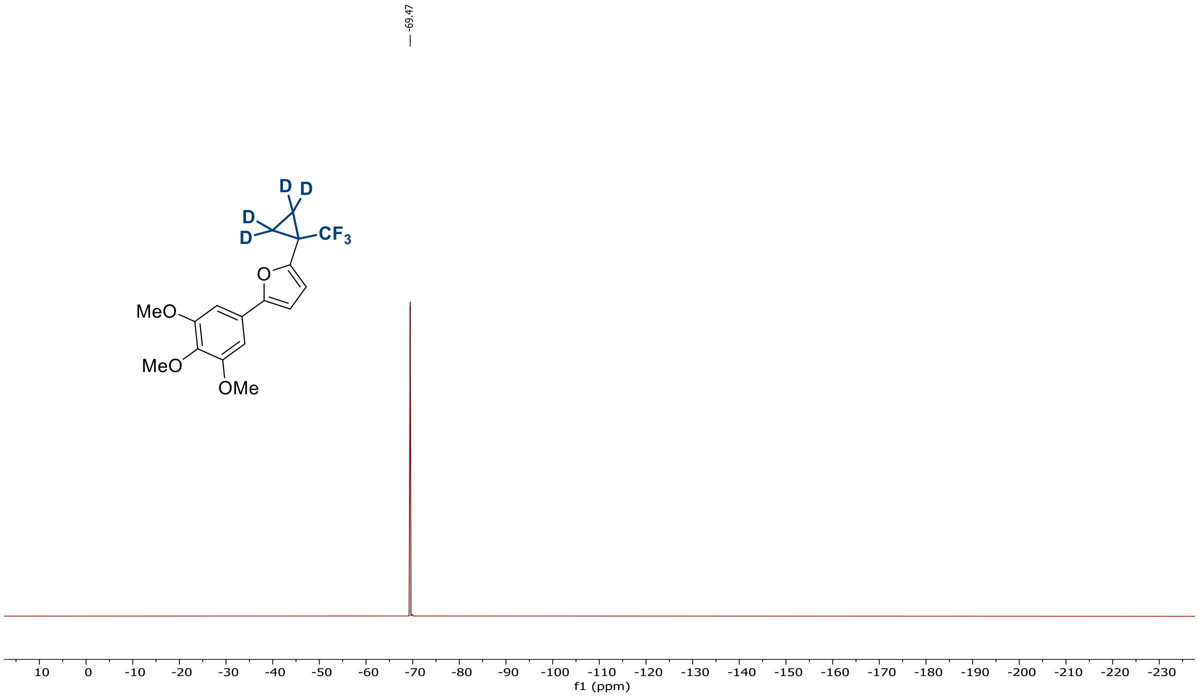

$^2$H NMR (92 MHz, $CHCl_3$)

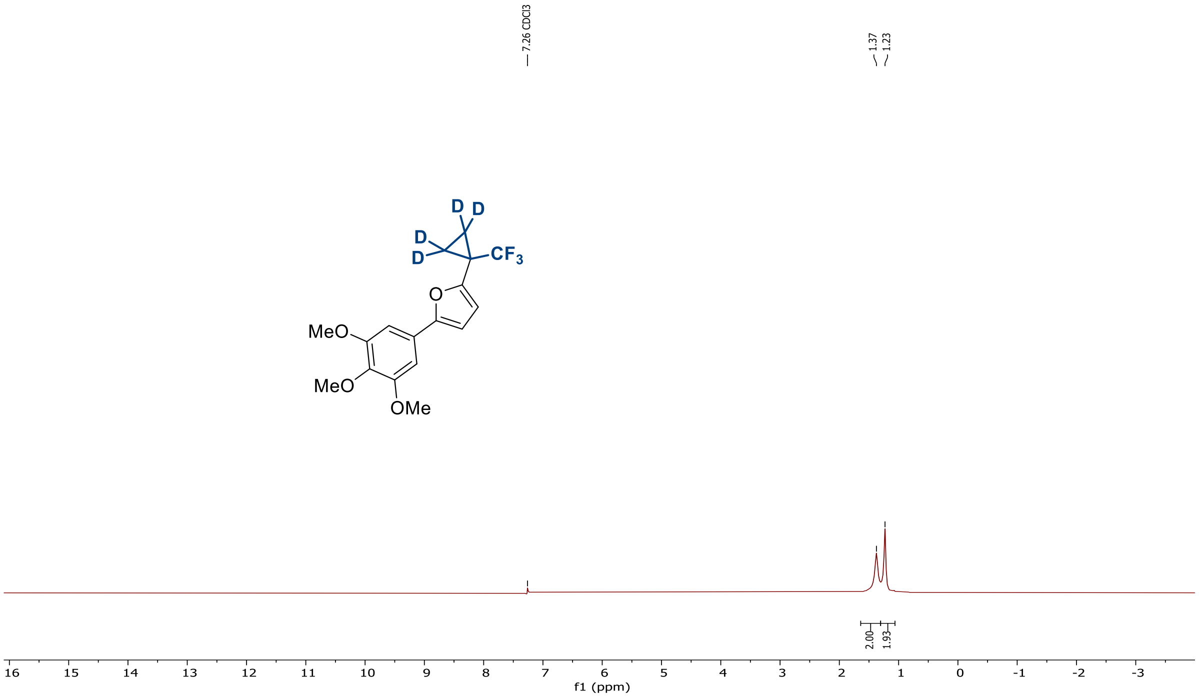


Compound **8**:

$^1$H NMR (300 MHz, $CDCl_3$)

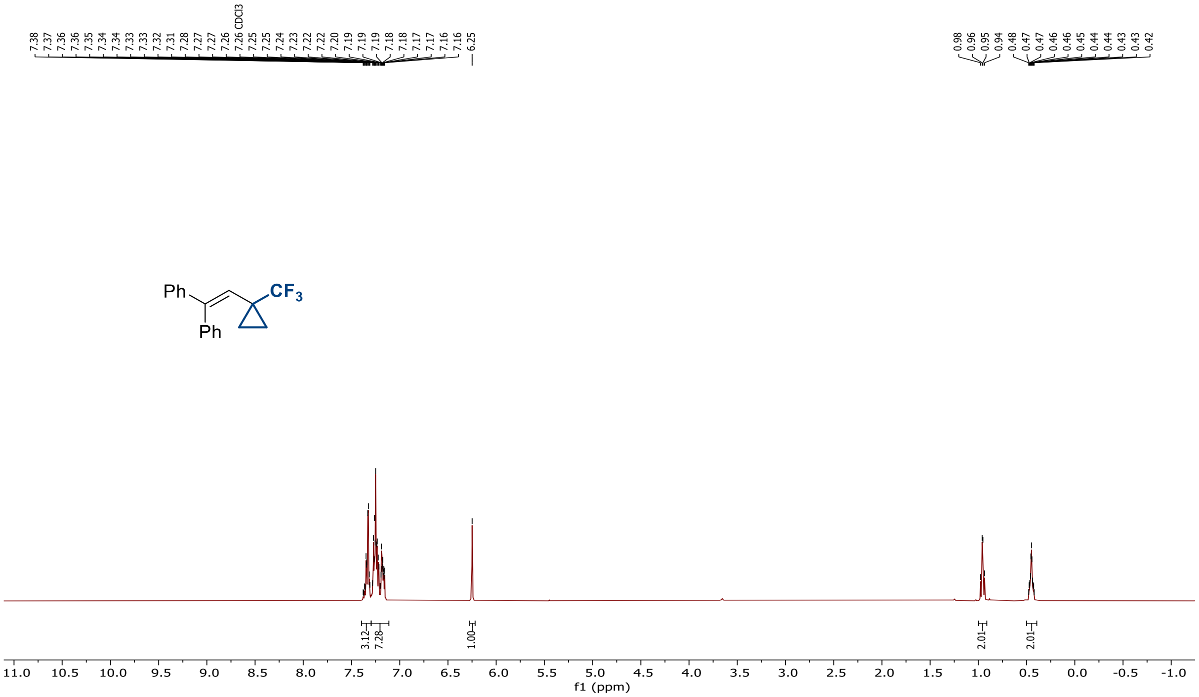

$^{13}C\{1H\}$ NMR (101 MHz, $CDCl_3$)

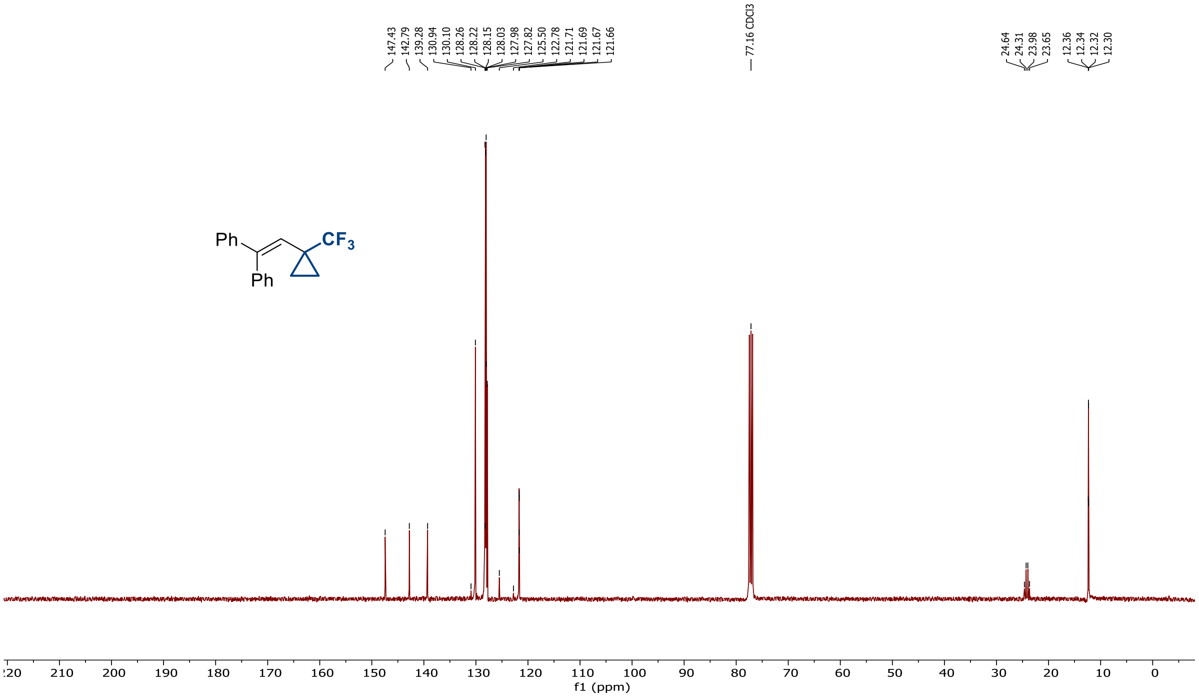


$^{19}F$ NMR (288 MHz, $CDCl_3$)

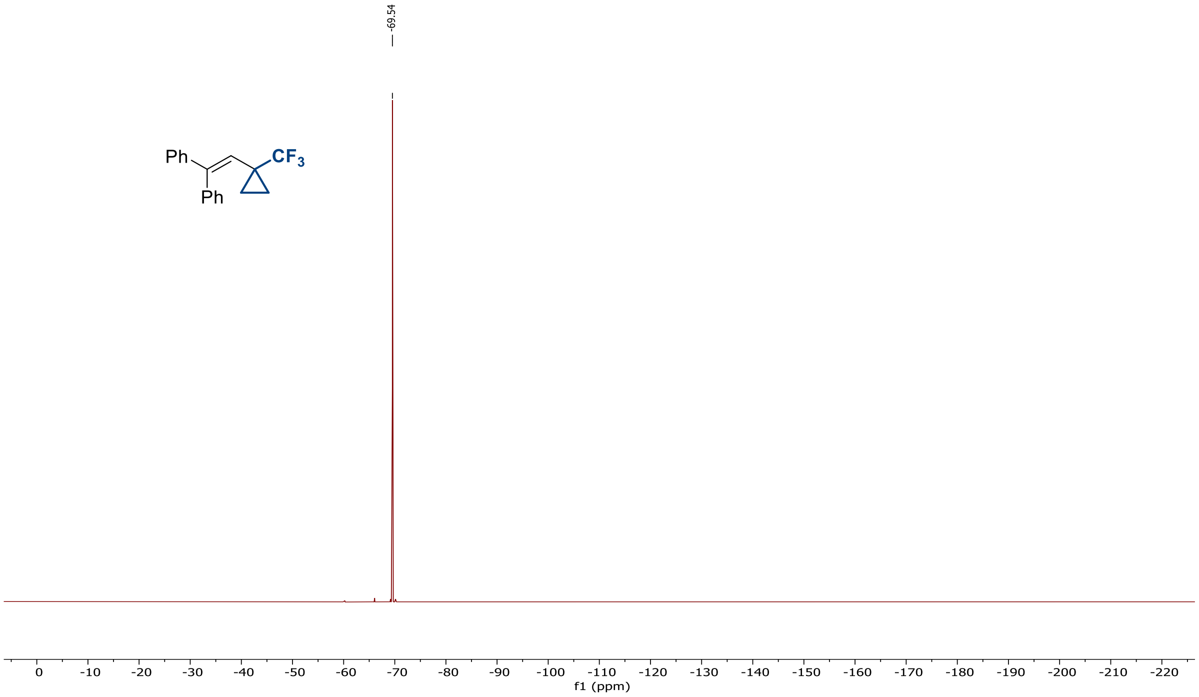

Compound **S3**:

$^{1}$H NMR (400 MHz, $CDCl_3$)

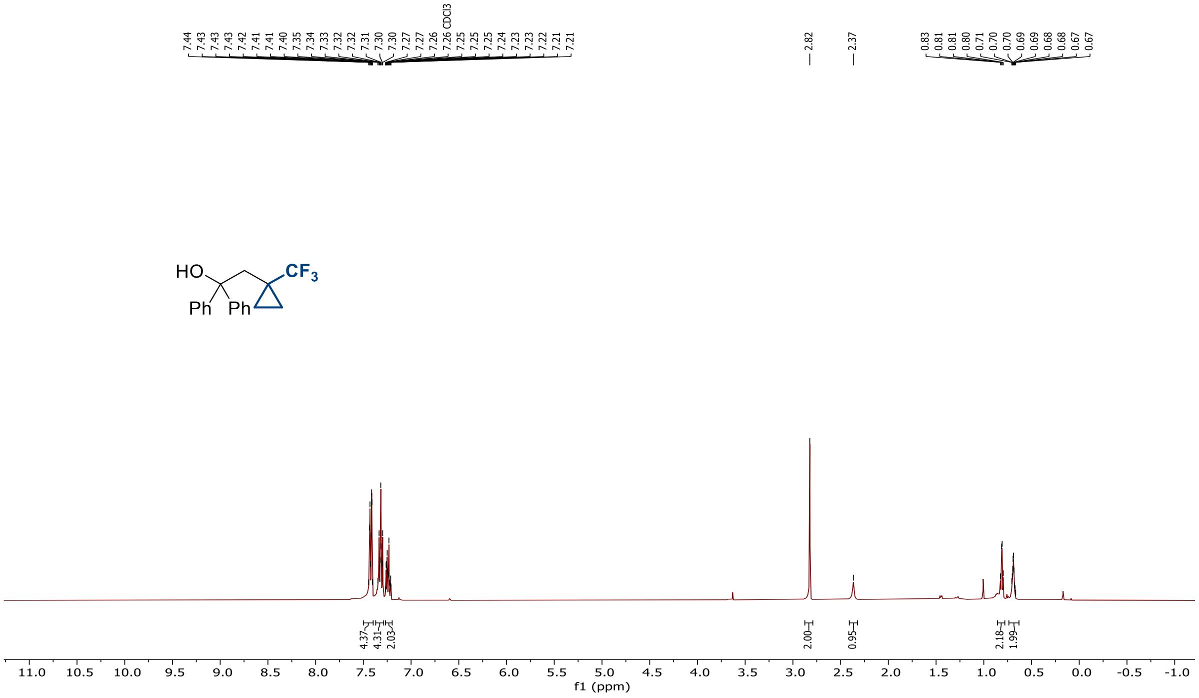


$^{13}$C{1H} NMR (101 MHz, $CDCl_3$)

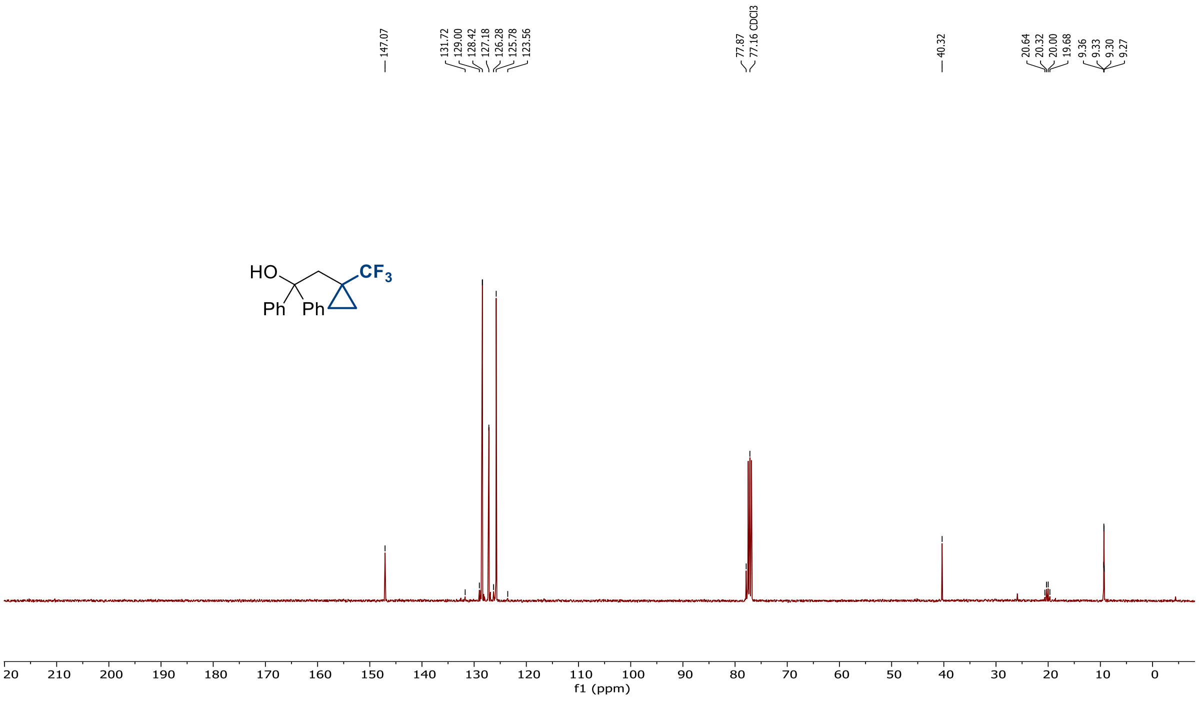

$^{19}F$ NMR (377 MHz, $CDCl_3$)

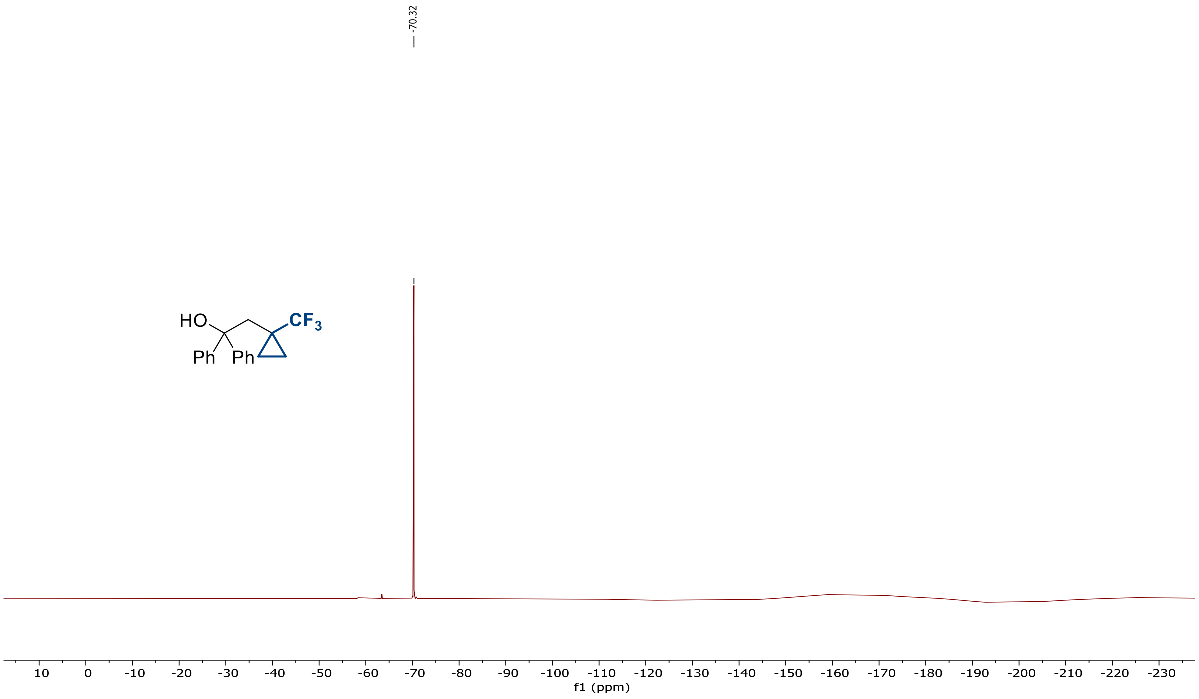


## COMPUTATIONAL STUDIES

Calculation of global electrophilicity index $\omega$ and nucleophilicity index $\omega^-$ for the TFCp radical were carried out according to literature.[29] The global electrophilicity index is defined as

$$\omega = \mu^2 / 2\eta \quad (1)$$

with $\mu$ being the chemical potential and $\eta$ the chemical hardness. Both values are calculated from the vertical ionization energy $I$ and the electron affinity $A$ following equation (2) and (3). These are directly calculated from the N, N+1 and N-1 electron single point energies.

$$\mu = -\frac{1}{2}(I + A) \quad (2)$$

$$\eta = (I + A) \quad (3)$$

The nucleophilicity index is defined as a scale relative to the fluorine radical. It is calculated according to equation (4):

$$\omega^- = {}^1/_2\, \eta_{TFCp} \frac{(\mu_{TFCp} - \mu_F)^2}{(\eta_{TFCp} + \eta_F)} \quad (4)$$

Here, the indexed $\mu$ and $\eta$ belong to the TFCp and fluorine radical.

Geometry optimization of the TFCp radical was carried out with Gaussian 16, Rev. A.03, and the output files was analyzed with GaussView 6.0.19.[30] The level of theory used was unrestricted B3LYP/def2-TZVP and D3 dispersion correction with Becke-Johnson damping.[31,32] Multiwfn 3.6 was used to generate the N, N+1 and N-1 input files which were then submitted to Gaussian for calculation. Subsequently, the electrophilic and nucleophilic indices were calculated by Multiwfn for the TFCp radical and the fluorine radical (see Table **S5**).[33]

**Table S5**: Calculated values for the TFCp and fluorine radical.

| | TFCp radical | | F radical | |
|---|---|---|---|---|
| | [Hartree] | [eV] | [Hartree] | [eV] |
| E(N) | -454.473278 | -12366.9 | -99.770151 | -2714.9 |
| E(N+1) | -454.121675 | -12357.3 | -98.98593 | -2693.6 |
| E(N-1) | -454.506121 | -12367.8 | -99.876334 | -2717.8 |
| E_HOMO(N) | -0.259093 | -7.1 | -0.447226 | -12.2 |
| E_HOMO(N+1) | -0.592312 | -16.1 | -1.102837 | -30.0 |
| E_HOMO(N-1) | 0.049456 | 1.3 | 0.058354 | 1.6 |
| Vertical I | -0.032844 | -0.9 | -0.106184 | -2.9 |
| Vertical A | -0.351602 | -9.6 | -0.78422 | -21.3 |
| Mulliken electronegativity | -0.192223 | -5.2 | -0.445202 | -12.1 |
| Chemical potential $\mu$ | 0.192223 | 5.2 | 0.445202 | 12.1 |
| Hardness $\eta$ | 0.318758 | 8.7 | 0.678037 | 18.5 |
| Softness | 3.137173 | 0.1 | 1.474846 | 0.1 |
| Electrophilicity index | 0.057959 | 1.6 | 0.146161 | 4.0 |
| Nucleophilicity index | 0.076105 | 2.1 | -0.112028 | -3.0 |

# REFERENCES


[1] X. Li, C. Golz, M. Alcarazo, *Angew. Chem. Int. Ed.* **2021**, *60*, 6943−6948.

[2] A. Aljarilla, R. Córdoba, A. G. Csaky, I. Fernández, F. López Ortiz, J. Plumet, G. Ruiz Gómez, *Eur. J. Org. Chem.* **2006**, *2006*, 3969−3976.

[3] H. Yang, N. Huo, P. Yang, H. Pei, H. Lv, X. Zhang, *Org. Lett.* **2015**, *17*, 4144−4147.

[4] E. V. V. Reddy, J. Ramanatham, N. Devanna, K. Srinivasa Reddy, D. Rajender, *J. Heterocycl. Chem.* **2013**, *50*, 221−227.

[5] G. M. Sheldrick, *Acta Crystallogr. A* **2008**, *64*, 112−122.

[6] F. Kleemiss, O. V. Dolomanov, M. Bodensteiner, N. Peyerimhoff, L. Midgley, L. J. Bourhis, A. Genoni, L. A. Malaspina, D. Jayatilaka, J. L. Spencer, F. White, B. Grundkötter-Stock, S. Steinhauer, D. Lentz, H. Puschmann, S. Grabowsky, *Chem. Sci.* **2020**, *12*, 1675−1692.

[7] O. V. Dolomanov, L. J. Bourhis, R. J. Gildea, J. A. K. Howard, H. Puschmann, *J. Appl. Cryst.* **2009**, *42*, 339−341.

[8] T. Kottke, D. Stalke, *J. Appl. Cryst.* **1993**, *26*, 615−619.

[9] T. Kang, W. Cao, L. Hou, Q. Tang, S. Zou, X. Liu, X. Feng, *Angew. Chem. Int. Ed.* **2019**, *58*, 2464−2468.

[10] N. S. Y. Loy, S. Choi, S. Kim, C.-M. Park, *Chem. Commun.* **2016**, *52*, 7336−7339.

[11] B. H. Lipshutz, R. Moser, K. R. Voigtritter, *Isr. J. Chem.* **2010**, *50*, 691−695.
[12] I. Khan, B. G. Reed-Berendt, R. L. Melen, L. C. Morrill, *Angew. Chem. Int. Ed.* **2018**, *57*, 12356−12359.
[13] W. Dong, Z. Ye, W. Zhao, *Angew. Chem. Int. Ed.* **2022**, *61*, e202117413.
[14] A. Carrër, J.-D. Brion, S. Messaoudi, M. Alami, *Org. Lett.* **2013**, *15*, 5606−5609.
[15] V. Hellgren, P. Singh, A. Kulkarni, N. Bagheri, J. Widengren, G. Manavalan, F. Almqvist, *J. Org. Chem.* **2024**, *89*, 11802−11810.
[16] S. Sau, S. Takizawa, H. Y. Kim, K. Oh, *Org. Lett.* **2024**, *26*, 8821−8826.
[17] P. Ghosh, Y. Byun, N. Y. Kwon, J. Y. Kang, N. K. Mishra, J. S. Park, I. S. Kim, *Cell Rep. Phys. Sci.* **2022**, *3*, 100819.
[18] a) S. Gupta, V. Srinivasu, D. Sureshkumar, *Org. Biomol. Chem.* **2023**, *21*, 8136−8140; b) H. Ni, Y. Li, J. Deng, X. Shi, Q. Pan, *New J. Chem.* **2021**, *45*, 22432−22436.
[19] J. M. Minguez, J. J. Vaquero, J. Alvarez-Builla, O. Castaño, J. L. Andrés, *J. Org. Chem.* **1999**, *64*, 7788−7801.
[20] a) P. García-Domínguez, A. R. de Lera, *J. Org. Chem.* **2022**, *87*, 12528−12546; b) A. Kasahara, R. Yamada, T. Hyodo, K. Yamaguchi, Y. Otani, S. Sumimoto, M. Okada, T. Ohwada, *J. Org. Chem.* **2025**, *90*, 623−635.
[21] H. Saito, S. Otsuka, K. Nogi, H. Yorimitsu, *J. Am. Chem. Soc.* **2016**, *138*, 15315−15318.
[22] Y. Shi, K. Wang, Y. Ding, Y. Xie, *Org. Biomol. Chem.* **2022**, *20*, 9362−9367.
[23] Y. Baba, N. Hirukawa, N. Tanohira, M. Sodeoka, *J. Am. Chem. Soc.* **2003**, *125*, 9740−9749.
[24] M. Othman, P. Pigeon, B. Decroix, *Tetrahedron* **1997**, *53*, 2495−2504.
[25] J. R. Lakowicz, *Principles of Fluorescence Spectroscopy*, Springer US, New York, **2006**.
[26] H. J. Kuhn, S. E. Braslavsky, R. Schmidt, *Pure Appl. Chem.* **2004**, *76*, 2105−2146.
[27] M. Montalti, A. Credi, L. Prodi, M. T. Gandolfi, *Handbook of Photochemistry* (CRC/Taylor & Francis, Boca Raton, Fl, Ed. 3, **2006**).
[28] B. Spingler, S. Schnidrig, T. Todorova, F. Wild, *CrystEngComm* **2012**, *14*, 751−757.
[29] F. de Vleeschouwer, V. van Speybroeck, M. Waroquier, P. Geerlings, F. de Proft, *Org. Lett.* **2007**, *9*, 2721−2724.
[30] Gaussian 16, Revision A.03, M. J. Frisch, G. W. Trucks, H. B. Schlegel, G. E. Scuseria, M. A. Robb, J. R. Cheeseman, G. Scalmani, V. Barone, G. A. Petersson, H. Nakatsuji, X. Li, M. Caricato, A. V. Marenich, J. Bloino, B. G. Janesko, R. Gomperts, B. Mennucci, H. P. Hratchian, J. V. Ortiz, A. F. Izmaylov, J. L. Sonnenberg, D. Williams-Young, F. Ding, F. Lipparini, F. Egidi, J. Goings, B. Peng, A. Petrone, T. Henderson, D. Ranasinghe, V. G. Zakrzewski, J. Gao, N. Rega, G. Zheng, W. Liang, M. Hada, M. Ehara, K. Toyota, R. Fukuda, J. Hasegawa, M. Ishida, T. Nakajima, Y. Honda, O. Kitao, H. Nakai, T. Vreven, K. Throssell, J. A. Montgomery, Jr., J. E. Peralta, F. Ogliaro, M. J. Bearpark, J. J. Heyd, E. N. Brothers, K. N. Kudin, V. N. Staroverov, T. A. Keith, R. Kobayashi, J. Normand, K. Raghavachari, A. P. Rendell, J. C. Burant, S. S. Iyengar, J. Tomasi, M. Cossi, J. M. Millam, M. Klene, C. Adamo, R. Cammi, J. W. Ochterski, R. L. Martin, K. Morokuma, O. Farkas, J. B. Foresman, and D. J. Fox, Gaussian, Inc., Wallingford CT, 2016.
[31] a) A. Schäfer, H. Horn, R. Ahlrichs, *J. Chem. Phys.* **1992**, *97*, 2571−2577; b) F. Weigend, R. Ahlrichs, *Phys. Chem. Chem. Phys.* **2005**, *7*, 3297−3305; c) F. Weigend, *Phys. Chem. Chem. Phys.* **2006**, *8*, 1057−1065.

[32] a) S. Grimme, J. Antony, S. Ehrlich, H. Krieg, *J. Chem. Phys.* **2010**, *132*, 154104; b) S. Grimme, S. Ehrlich, L. Goerigk, *J. Comput. Chem.* **2011**, *32*, 1456−1465.
[33] T. Lu, F. Chen, *J. Comput. Chem*. **2012**, *33*, 580-592.
